# hsa-miR-548v controls the viscoelastic properties of human cardiomyocytes and improves their relaxation rates

**DOI:** 10.1172/jci.insight.161356

**Published:** 2024-01-02

**Authors:** Eva Vermersch, Salomé Neuvendel, Charlène Jouve, Andrea Ruiz-Velasco, Céline Pereira, Magali Seguret, Marie-Elodie Cattin-Messaoudi, Sofia Lotfi, Thierry Dorval, Pascal Berson, Jean-Sébastien Hulot

**Affiliations:** 1 Université Paris Cité, Inserm, PARCC, F-75015 Paris, France.; 2 Institut de recherches Servier, In vitro Pharmacology unit, and; 3 Institut de recherches Servier, Cardiovascular and Metabolism Therapeutic Area, Croissy-sur-seine, France.; 4 CIC1418 and DMU CARTE, AP-HP, Hôpital Européen Georges-Pompidou, F-75015, Paris, France.

**Keywords:** Cardiology, Stem cells, Heart failure, iPS cells

## Abstract

The impairment of left ventricular (LV) diastolic function with an inadequate increase in myocardial relaxation velocity directly results in lower LV compliance, increased LV filling pressures, and heart failure symptoms. The development of agents facilitating the relaxation of human cardiomyocytes requires a better understanding of the underlying regulatory mechanisms. We performed a high-content microscopy-based screening in human induced pluripotent stem cell–derived cardiomyocytes (hiPSC-CMs) using a library of 2,565 human miRNA mimics and measured relaxation kinetics via high-computing analyses of motion movies. We identified hsa-miR-548v, a primate-specific miRNA, as the miRNA producing the largest increase in relaxation velocities. This positive lusitropic effect was reproduced in engineered cardiac tissues generated with healthy and *BRAF* T599R mutant hiPSC-CMs and was independent of changes in calcium transients. Consistent with improvements in viscoelastic responses to mechanical stretch, RNA-Seq showed that hsa-miR-548v downregulated multiple targets, especially components of the mechanosensing machinery. The exogenous administration of hsa-miR-548v in hiPSC-CMs notably resulted in a significant reduction of ANKRD1/CARP1 expression and localization at the sarcomeric I-band. This study suggests that the sarcomere I-band is a critical control center regulating the ability of cardiomyocytes to relax and is a target for improving relaxation and diastolic dysfunction.

## Abstract 2

<p>Hsa-miRNA-548v promotes an increase in relaxation velocities of human cardiomyocytes by down-regulating components of the mechano-sensing machinery.</p>

## Graphical abstract

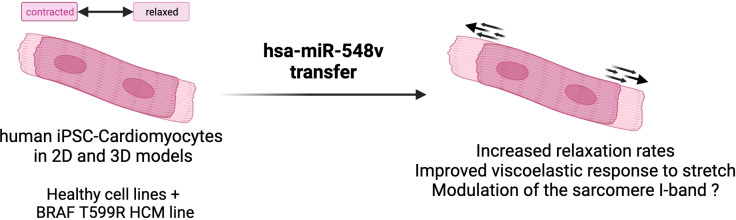

## Introduction

Left ventricular (LV) diastolic function plays an important role in cardiac performance and is mainly determined by the efficiency of myocardial relaxation. In the healthy human myocardium, the rate of myocardial relaxation directly influences the ability to fill the LV while keeping low filling pressure (1, 2). In response to a higher demand, such as during exercise, relaxation velocity is increased in order to accelerate diastolic LV filling, despite a shortening of the time available for ventricular filling with tachycardia (3, 4). Reciprocally, an impaired diastolic reserve, measured as an inadequate increase in myocardial relaxation velocity, is considered a hallmark of heart failure (especially for heart failure with preserved ejection fraction [HFpEF]) and is associated with a progressive decline in exercise capacity (2, 4–6). In theory, pharmacological agents that facilitate myocardial relaxation would improve LV compliance and would be ideal for the treatment of diastolic dysfunction. However, our understanding of the mechanisms regulating myocardial relaxation is limited, especially in humans.

Myocardial relaxation is a complex multicomponent process that depends, at least in part, on the ability of cardiomyocytes (CMs) to relax (i.e., lusitropy). After each contraction, CMs exhibit a nonlinear viscoelastic behavior as they rapidly return to their original configuration without any memory of the mechanical compaction induced by the contraction. In addition, the stretching of the CMs (within the LV walls) as the heart fills with blood during diastole invokes considerable viscoelastic forces (7, 8). In addition to the influence of calcium cycling, it has been proposed that the rapid elastic response of CMs depends on elements composing the myofilament and the cytoskeleton. For example, the giant protein titin is an important determinant of myofilament diastolic tension (9, 10) and a contributor to viscous forces (11). Changes in titin phosphorylation modifies its compliance, which is often altered in diseases with reduced diastolic compliance (12). Recent data also demonstrate the importance of the nonsarcomeric cytoskeleton (consisting of microtubules and desmin intermediate filaments) in CM viscoelasticity. Posttranslational detyrosination of microtubules influences the stability of the microtubule network and promotes its cross-linking with the myocyte cytoskeleton and intermediate filament network (13, 14). The *Z* disc myofilament is also thought to play a key role in controlling diastolic tension in CMs. Consistently, restrictive cardiomyopathy, primarily characterized by impaired relaxation and diastolic dysfunction, is found in patients with genetic mutations affecting *Z* disc proteins (15–17).

It is likely that the multiscale remodeling of these elements in heart disease (and particularly in HFpEF) leads to abnormal myocardial viscoelasticity and a disturbed ventricular compliance that directly impede diastolic filling. However, our understanding of myocardial relaxation and its regulation remains incomplete. Techniques and methods to characterize myocardial viscoelasticity at the tissue and the cell levels have only recently emerged, and the field is globally understudied, especially in human CMs. Similarly, the impact of the CM viscoelastic properties on the physiology of cardiac performance is not understood, especially in humans.

MicroRNAs (miRNAs) are endogenous 22-nucleotide single-stranded RNAs that can bind and suppress multiple mRNAs. It is estimated that miRNAs control almost every cellular process and 60% of the proteome (18). Hence, the miRNA library is an attractive tool in the identification of regulators of a specific phenotype within a phenotypic or functional screening strategy (19). Here we set out to systematically identify miRNAs that enhance CM relaxation using a synthetic miRNA library of human origin applied to human models based on human induced pluripotent stem cell–derived CMs (hiPSC-CMs). Our primary goal was to identify miRNAs that significantly improve relaxation rates and to gain further insight into the associated mechanisms that most strongly influence CM relaxation.

## Results

### Screening for miRNAs regulating CM relaxation velocities.

**Figure 1:**
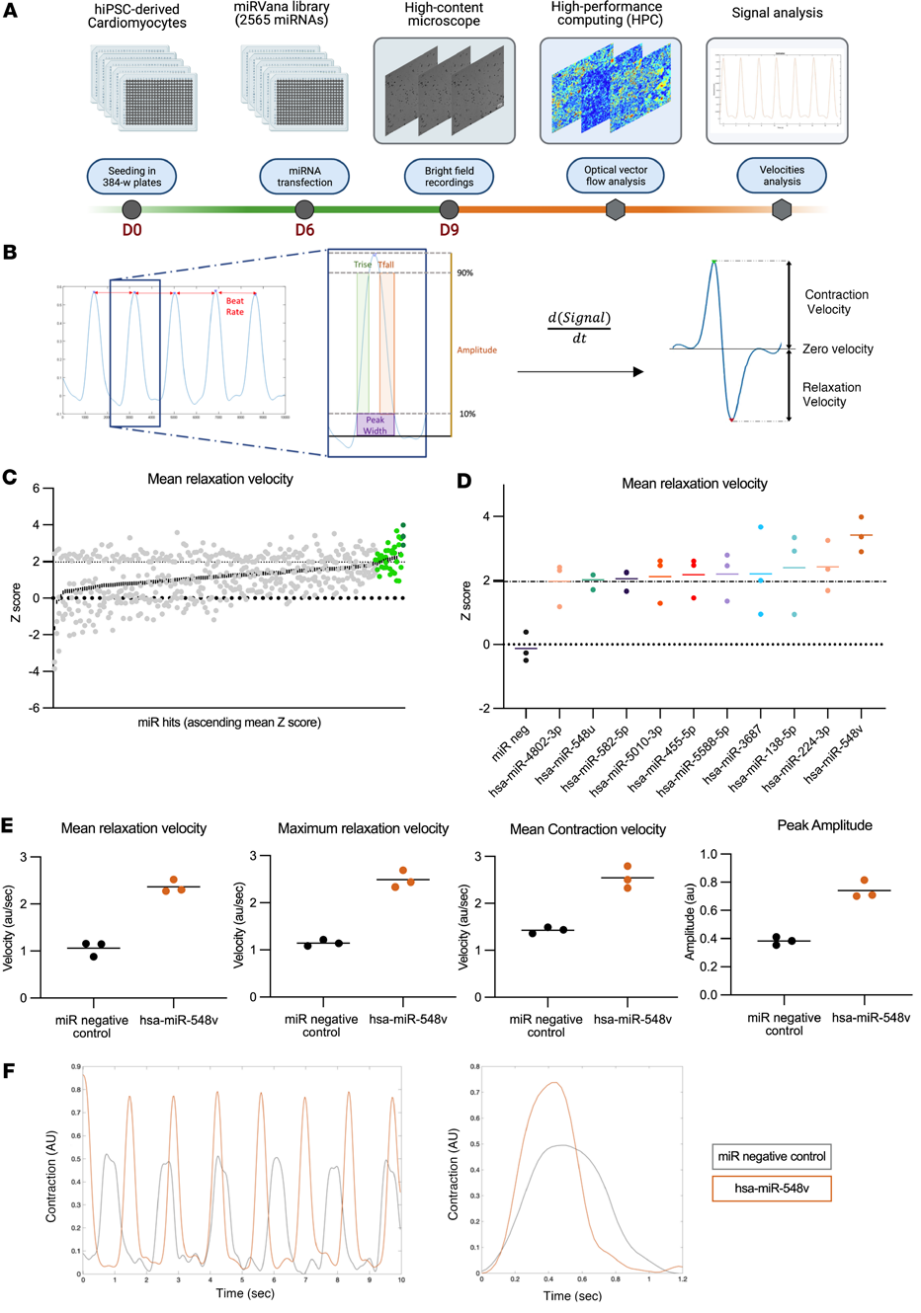
High-content screening identified miRNAs accelerating the relaxation phase of cardiomyocytes. (**A**) Screening workflow. The green line indicates the culture and transfection phase until microscope video recordings. The orange line indicates the postprocessing of videos to acquire and analyze motion parameters. (**B**) Signal analysis. Representative example of beat-to-beat motion signal analysis (left) and derivation of velocities from the integration of signal overtime (right). (**C**) miRNA Hits sorted by ascending *Z* score. Green dots indicate the 10 miRNAs with *Z* score ≥ 2 in at least 2 independent replicates. (**D**) Individual *Z* scores of miRNA hits in at least 2 independent replicates (*Z* score ≥ 2, *P* < 0.05) and miRNA negative control. (**E**) Mean relaxation velocity, maximum relaxation velocity, mean contraction velocity, and peak amplitude of motion in hiPSC-derived cardiomyocytes transfected with hsa-miR-548v or miRNA negative control. (**F**) Representative records of beat-to-beat motion (left) and averaged contraction/relaxation cycle (right) recorded from cardiomyocytes transfected with hsa-miR-548v (orange) or miRNA negative control (gray).

We performed a high-content, microscopy-based, high-throughput screening (HTS) in hiPSC-CM using a library of 2,565 human miRNA mimics (miRbase sequence database version 21) (Figure 1A). The miRNA mimics were transfected into the hiPSC-CM cultures (forward transfection), which presented as beating monolayers in 384-well plates. Three days later, we recorded high-speed videos of iPSC-CM beating monolayers in each well using an automated high-content screening microscope. The image sequences were then analyzed by optical vector flow analysis with a high-performance computer (HPC) in order to model the contractile movements of hiPSC-CMs and to measure the relaxation and contraction velocities (Figure 1B and Supplemental Videos 1 and 2; supplemental material available online with this article; https://doi.org/10.1172/jci.insight.161356DS1). The screening was performed in triplicate. In addition to the miRNA negative control, a random sequence that has been tested in human cell lines and shown to have no detectable effects, we included 3 miRNA mimics in each plate as quality controls. We also tested different miRNA concentrations to assess the effect of transfection on our readouts (Supplemental Figure 1A).

Compared with hiPSC-CMs treated with control miRNA, 163 miRNAs accelerated the mean relaxation velocity in at least 1 of the 3 independent screen replicates (*Z* score≥2, *P* < 0.05) (Figure 1C), but 10 miRNAs significantly increased the relaxation velocity in at least 2 independent replicates (Figure 1D). The largest and most reproducible changes in relaxation phase were observed with hsa-miR-548v, which significantly increased the relaxation velocities in the 3 independent screen replicates (Figure 1D). Similar results were obtained when considering the maximal relaxation velocities (Figure 1E). In addition to its effect on relaxation, hsa-miR-548v also increased contraction velocity, beating amplitude, and rate (Figure 1E), suggesting a global improvement in CM mechanics (Figure 1F).

hsa-miR-548v is part of the large primate-specific miR-548 family and is located on chromosome 8 (cytogenetic band 8p22). The miR-548 superfamily is the largest miRNA family in the human genome, with 74 miRNAs members. A downregulation of at least 10 miR-548 family members was identified by genome-wide analysis on peripheral blood mononuclear cells (PBMCs) from patients with heart failure with reduced ejection fraction (20). However, little is known about the implication of hsa-miR-548v in cardiovascular disorders. We used miRNATissueAtlas2 (21), a small noncoding RNA expression tissue atlas determined from humans, to explore hsa-miR-548v expression in human organs and found very low levels of expression in the 21 organs explored (Supplemental Figure 1B). Moreover, FANTOM5 (22) shows an enrichment of hsa-miR-548v in endothelial cells (Supplemental Figure 1C). These data suggest that there is either no or limited basal expression of hsa-miR-548v in CMs or fibroblasts, and we further confirmed the lack of expression of hsa-miR-548v in the different hiPSC-CMs included in this study.

The results of this HTS thus indicated hsa-miR-548v transfer as having interesting lusitropic effects, prompting us to further investigate its effect in CMs.

### hsa-miR-548v improves cardiac relaxation at the tissue level.

**Figure 2:**
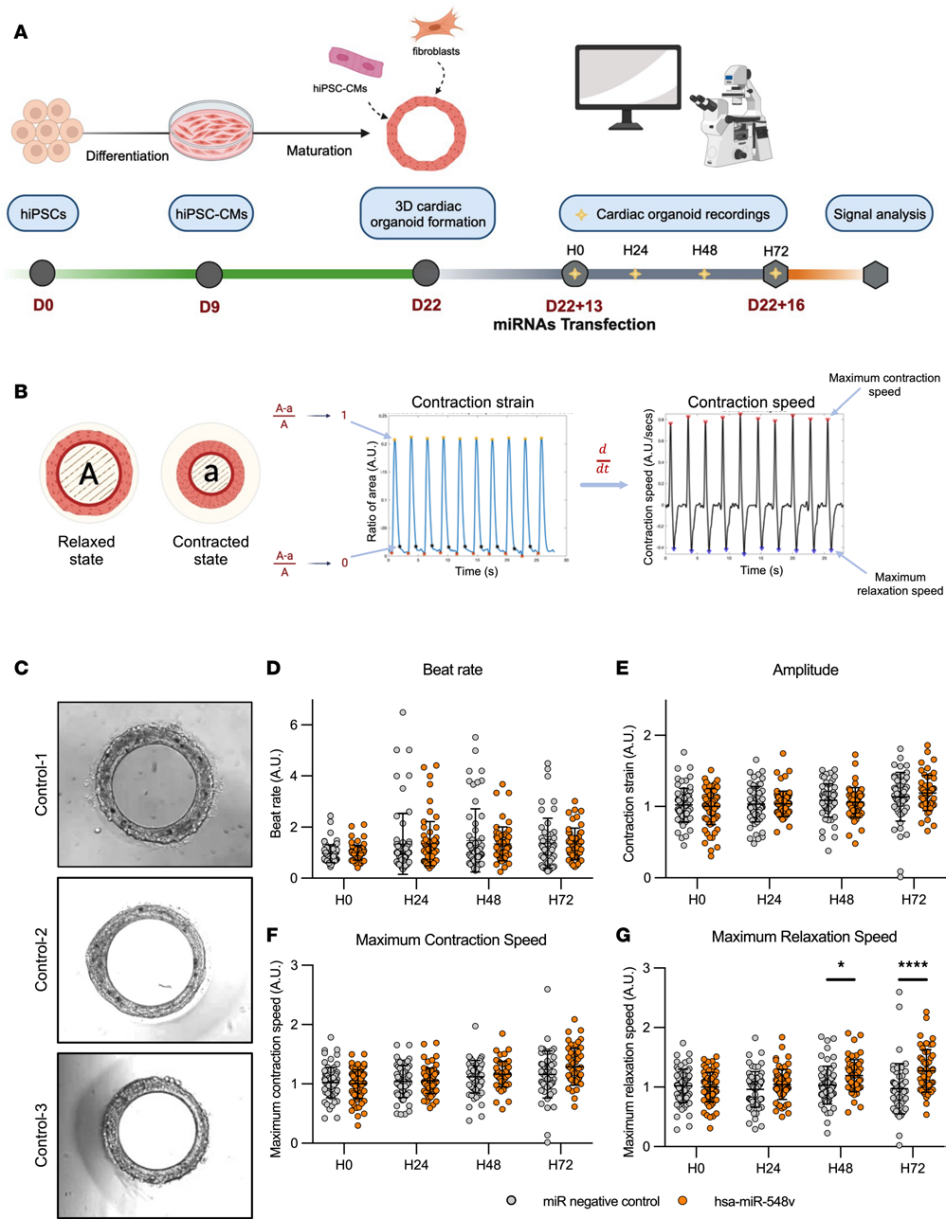
Generation and transfection of control cardiac organoids with hsa-miR-548v leads to increase relaxation rates. (**A**) Schematic overview of the cardiac differentiation and cardiac organoid generation protocol. Cardiac organoids were transfected and recorded after 2 weeks of culture in calcium enriched medium. miRNA transfection was performed 13 days after the generation of cardiac organoids, and movies were recorded daily up to hour 72 (H72)after transfection. (**B**) Schematic of representative graphs obtained after image analysis of the motion movies. (**C**) Representative bright-field images of tissues obtained from the 3 different control hiPS cell lines: Control-1, Control-2, and Control-3. (**D**–**G**) Beat rate (**D**), amplitude (**E**), maximum contraction speed (**F**), and maximum relaxation speed (**G**) of tissues measured at H0, H24, H48, and H72 after transfection. Control-1 (miR Neg, *n* = 22; hsa-miR-548v, *n* = 24) from 5 independent experiments each; Control-2 (miR Neg, *n* = 28; hsa-miR-548v, *n* = 31) from 3 independent experiments each, and Control-3 (miR Neg, *n* = 19; hsa-miR-548v, *n* = 15) from 2 independent experiments each. Values are given as mean ± SD. *P < 0.05, ****P < 0.0001 from Šídák post hoc comparisons between treatment groups, 2-way ANOVA.

The function of CMs depends on several parameters in their 3D environment, including the extracellular matrix and the multicellular interactions. Furthermore, hiPSC-CM display a more mature phenotype in 3D organoids as compared with 2D-monolayer culture (23). To further characterize the effects of hsa-miR-548v on cardiac function, we tested its effect on human engineered cardiac tissue (hECT) generated with different lines of hiPSC-CMs. The 3 additional iPSC lines were different from that used in the HTS (Supplemental Figure 2A) and were generated from 2 male and 1 female healthy subject as previously reported (24, 25). The differentiation protocol resulted in the production of more than 90% TNNT2^+^ cells for all lines (Supplemental Figure 2B) and CMs expressing typical sarcomere proteins (Supplemental Figure 2C). We used 2 different 3D platforms, as previously reported (26, 27), that used a mixture of hiPSC-CM/fibroblasts to either form a macrostructure (~1 cm) similar to a trabecular cardiac muscle (Supplemental Figure 3, A and B) or a microstructure (~400 μm) similar to a cardiac ring (Figure 2, A–C). After approximately 2 weeks of culture and just prior to miRNA transfection, hECT were imaged with a high-speed camera to capture their motion and assess their contractile movements (Supplemental Videos 3–6).

Consistent with predicted expression from public databases, we found no basal expression of hsa-miR-548v expression in hECT (Supplemental Figure 3C). Three days after transfection, hsa-miR-548v expression was highly increased in the transfected hECT with hsa-miR-548v, while hsa-miR-548v expression was absent in hECT transfected with miRNA negative control (Supplemental Figure 3C). hECT were imaged again in the following days (Figure 2A and Supplemental Figure 3A), and the posttransfection parameters were used to evaluate the relative changes in relaxation and contraction velocities. The beat rate or contractility parameters were not significantly changed after treatment with hsa-miR-548v (Figure 2, D–F). However, consistent with the HTS results, the relaxation velocities of the ring-shaped hECT increased progressively in the days following treatment with hsa-miR-548v, with a significant difference observed at 48 and 72 hours after transfection (Figure 2G), consistent in the 3 hiPSC lines. Similar results were found on the other 3D platform using macroscopic structures that were recorded under paced conditions (Supplemental Figure 3D). Supplemental Figure 3E shows representative signals 3 days after transfection. Relaxation velocity was significantly increased in hECT transfected with hsa-miR-548v as compared with hECT transfected with miRNA negative control (Supplemental Figure 3F), while there was a nonsignificant trend for a higher developed force in hECT transfected with hsa-miR-548v (Supplemental Figure 3G).

These data suggest that hsa-miR-548v transfer improves cardiac lusitropy in a multicellular environment formed at the tissue level, and they confirm its benefit on relaxation in different hiPSC-CM lines, generated from healthy subjects of both sexes, recorded under either spontaneous or paced conditions, and cultured in different multicellular formats.

### hsa-miR-548v does not change calcium transients.

**Figure 3:**
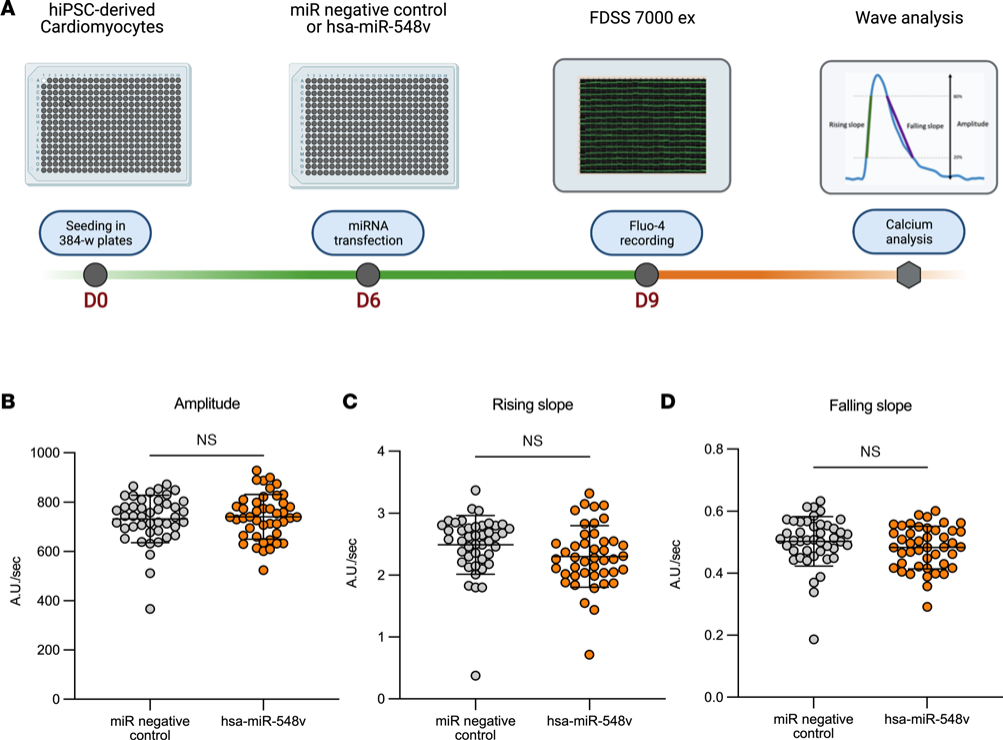
hsa-miR-548v does not affect calcium handling in hiPSC-CMs. (**A**) Assessment of calcium transients’ workflow. The green line represents hiPSC-CMs preparation, miRNA transfection, and loading with Fluo-4 to calcium transients recording (with FDSS). The orange line represents post-recording analysis (**B**–**D**) Amplitude, rising slope, and falling slope of the calcium transient measured in hiPSC-CM 3 days after transfection with hsa-miR-548v (*n* = 44) or miRNA negative control (*n* = 44). Student’s *t* test.

Since calcium is an important contributor to relaxation rate and force development in CMs, we next determined whether hsa-miR-548v affects calcium handling in hiPSC-CMs. We recorded the basal intracellular Ca^2+^ ([Ca^2+^]_i_) transients of iPSC-CMs treated with hsa-miR-548v or miRNA negative control and loaded 3 days later with Fluo-4, a Ca^2+^ indicator (Figure 3A). We found no significant changes in calcium transients’ amplitudes (Figure 3B and Supplemental Figure 4A) nor in release and reuptake kinetics parameters (Figure 3, C and D), whereas relaxation velocities were significantly improved in hsa-miR-548v–treated hiPSC-CMs concordant with our previous results (Supplemental Figure 4B). Overall, these results indicate that hsa-miR-548v significantly changed CMs’ lusitropy without involving changes in Ca^2+^ handling properties of hiPSC-CMS.

### hsa-miR-548v affects the internal distensibility properties of human iPSC-derived CMs at the single-cell level.

**Figure 4:**
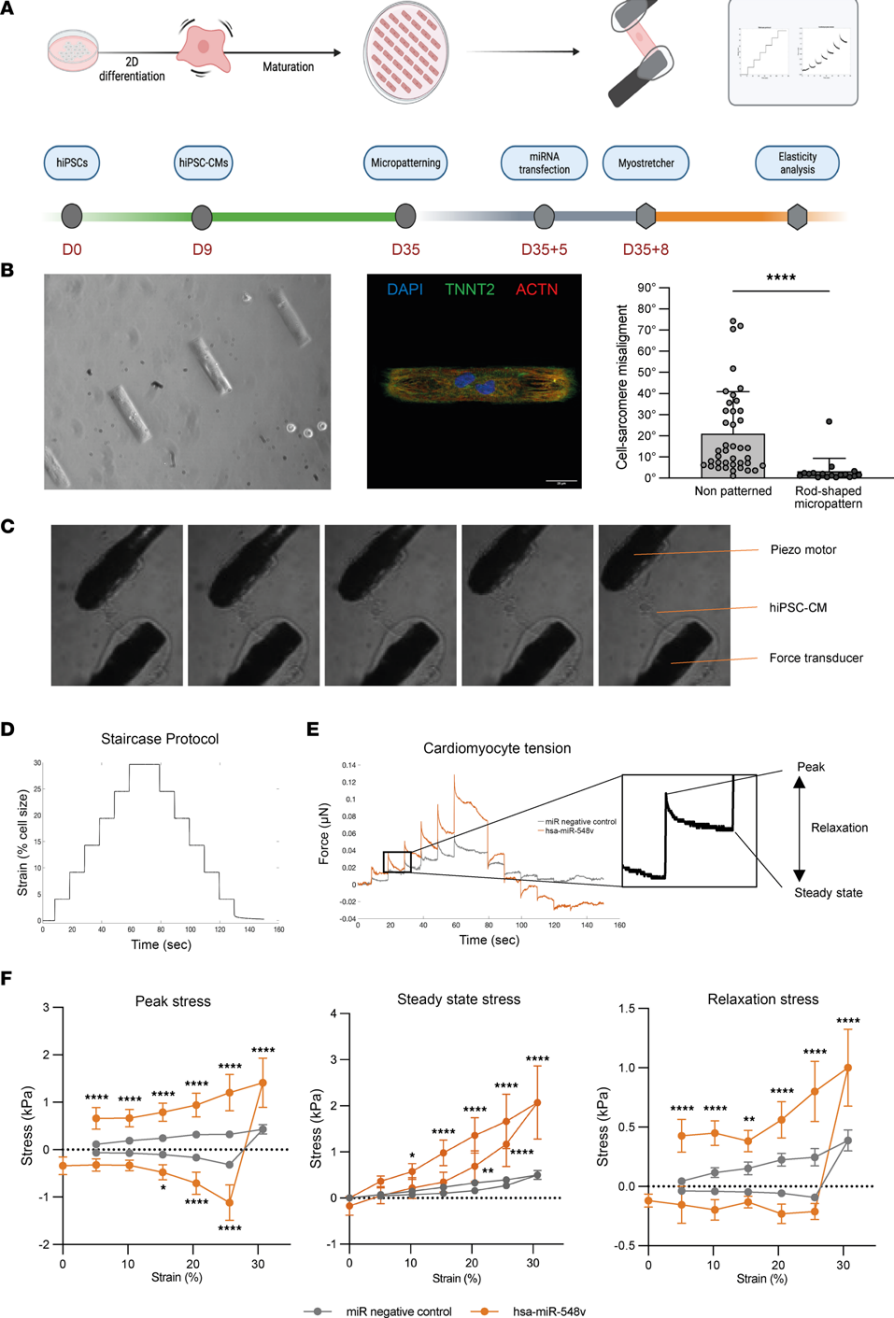
hsa-miR-548v modifies viscoelastic properties of cardiomyocyte in response to stretching. (**A**) Single-cell hiPSC-CM mechanical properties study protocol. The green line indicates the cells preparation up to culture on micropatterned rod-shape substrate. The gray line shows the miRNA transfection and MyoStretcher attachment and recordings. The orange line indicated the postprocessing of recordings. (**B**) Typical rod-shaped hiPSC-CM obtained on micropatterned culture plates. Optical image of micropatterned cells (objective, ×20) (left), and immunofluorescence images of a micropatterned cell expressing actin and TroponinT2 (Objective, ×63) (middle); sarcomere score alignment in nonpatterned (*n* = 40) versus rod-shape micropatterned hiPSC-CM (*n* = 17) (right). ****P < 0.0001. Mann-Whitney *U* test. (**C**) Typical images of different stretch increments of hiPSC-CM. (**D**) Staircase protocol; each increment represents a strain of 6 μm (5% stretch). (**E**) Force measurements at different stretch levels and derived parameters. (**F**) Mechanical response of hiPSC-CMs transfected with hsa-miR-548v (orange, *n* = 6) and miRNA negative control (gray, *n* = 8) to different stretch levels.Peak stress (viscous and elastic stress); steady state stress (elastic stress); relaxation stress (viscous response). *P < 0.05, **P = 0.0067, ****P < 0.0001. Two-way ANOVA with Šídák post hoc comparisons.

These results prompted us to investigate other processes that can regulate cardiac relaxation. We studied the mechanical properties of single-cell human hiPSC-derived CMs transfected with hsa-miR-548v or miRNA negative control (Figure 4A). The measure of mechanical properties requires typical rod-shape morphology as observed in isolated CMs from rodent and human adult hearts, whereas isolated hiPSC-CM appears to have a rounded morphology. We thus developed a protocol to generate hiPSC-CM on specifically designed micropatterned slides with a rod-shaped morphology (Figure 4, A and B). Briefly, the slides were stamped with Matrigel-coated rectangular islands (120 × 30 μm) surrounded with an antiadhesive agent. After seeding, hiPSC-CM grew in the predesigned areas and reached a rod-shaped morphology (Figure 4B). This method also imposed a constant size of CMs and a controlled background stiffness limiting variability (Figure 4B).

We used a similar protocol as Ballan and colleagues (28) to attach the cells and were able to successfully stretch the cells up to 40% of their initial length (Figure 4C). To assess length-tension relationship, we used a staircase protocol, in which a piezoelectric motor moved gradually for 6 μm with a 62.5 μm/sec stretch velocity, every 10 seconds (Figure 4D). Force measurements were held without any contraction inhibitors. Both CMs transfected with miRNA negative control or hsa-miR-548v showed a positive length-tension relationship as the forces increased with cell stretching (Frank-Starling Law) (Figure 4E). Viscous tension (relaxation stress) increased nonlinearly with stretch increasing. We observed a significant increase of effective (peak stress) and resting (steady state stress) tension of CMs transfected by hsa-miR-548v compared with the ones transfected by miRNA negative control (Figure 4, E and F), and these difference between groups strikingly grew as the stretch increased. Viscoelastic tension was increased during stretch of hsa-miR-548v–transfected CMs compared with miRNA negative control transfected CMs. hsa-miR-548v increased both elastic tension (*P* < 0.001) and a viscous tension (*P* = 0.0004) (Figure 4F). These data show that hsa-miR-548v affects viscoelastic response to stretch by increasing both its elastic and viscous tension.

### hsa-miR-548v changes the expression of intracellular components associated with mechanotransduction.

**Figure 5:**
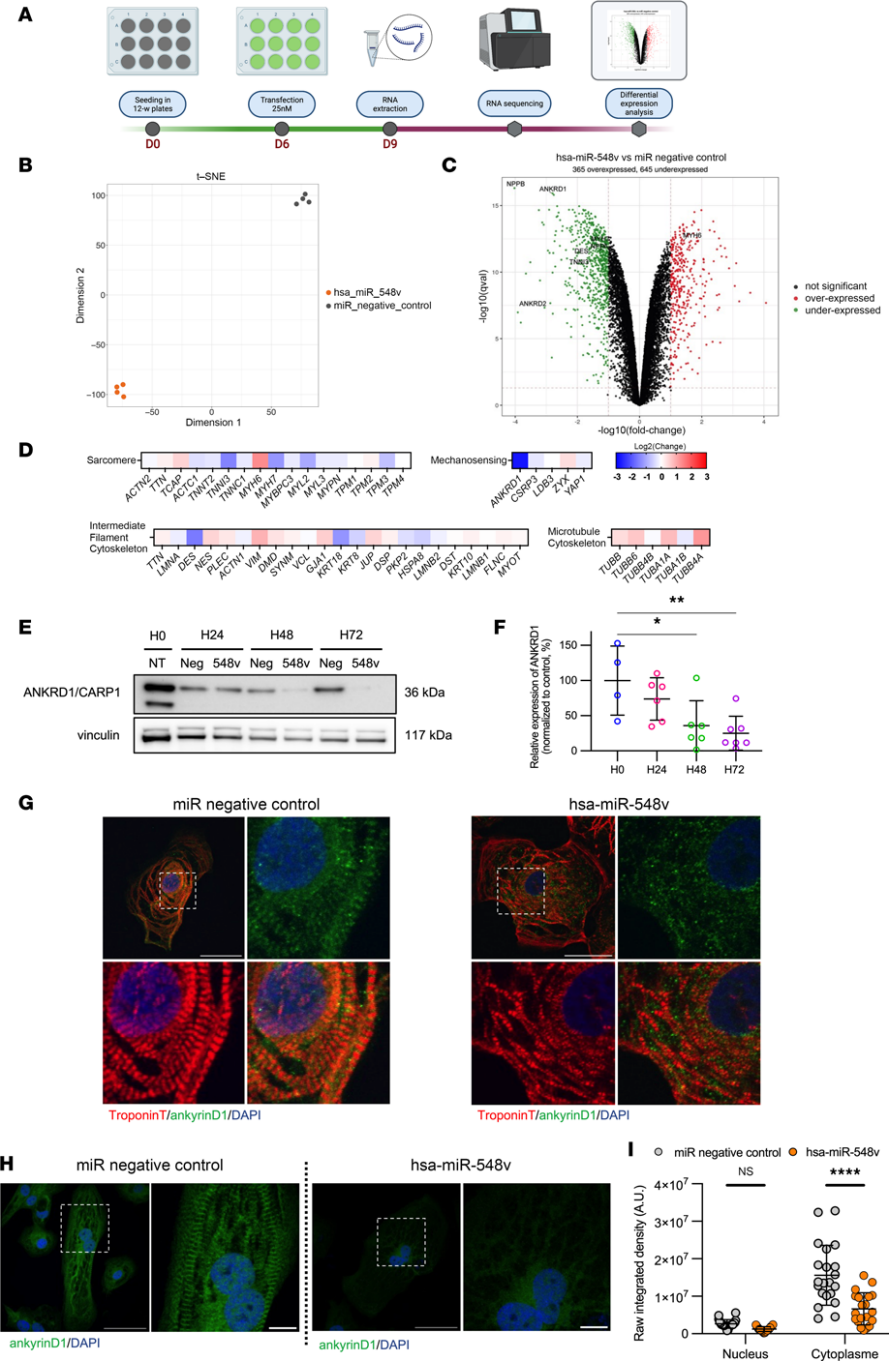
Transcriptomics analysis reveals downregulation of ankyrin repeat domain 1 protein in hiPSC-CMs transfected with hsa-miR-548v. (**A**) Schematic overview of RNA-sequencing experiment. (**B**) t-SNE plot of RNA sequencing data based on the 1000 most variant genes from hiPSC-CM transfected with hsa-miR-548v (orange, *n* = 4) or negative miRNA negative control (gray, *n* = 4). (**C**) Volcano plot of downregulated (green) and upregulated (red) genes in hiPSC-CMs transfected with hsa-miR 548v or miRNA negative control. NPPA: Natriuretic peptide A; NPPB: Natriuretic peptide B; ANKRD1: cardiac ankyrin protein 1; ANKRD2: cardiac ankyrin protein 2; DES: Desmin; TNNI3: Troponin I3, Cardiac; MYH6: alpha cardiac myosin heavy chain; MYH7: beta cardiac myosin heavy chain. (**D**) Log2-Fold change in expression of sarcomere components, mechano-sensing proteins, intermediate filament components and microtubules. (**E**) Representative western blot showing ankyrin repeat domain 1 protein (CARP1) expression in control cells before transfection (H0), and 24, 48 and 72 hours after transfection with miRNA negative control or with hsa-miR-548v. (**F**) Relative level of ankyrin repeat domain 1 protein expression normalized to the basal expression before transfection (*n* = 4) and expressed as ratio between hsa-miR-548v treated cells/miRNA negative control–treated cells at 24 hours (*n* = 6), 48 hours (*n* = 6), and 72 hours (*n* = 7) after transfection. *P < 0.05, **P < 0.01, ordinary 1-way ANOVA. (**G**) Representative images of control CM-hiPSCs transfected with miRNA negative control or hsa-miR-548v 72 hours after transfection, stained for troponinT (red) and ankyrin D1 (green) as well as DAPI (blue). Scale bar: 50 μm. (**H**) Representative images of control CM-hiPSCs transfected with miRNA negative control or hsa-miR-548v 72 hours after -transfection, stained for ankyrin D1 (green) and DAPI (blue). Scale bar: 50 μm. (**I**) Quantification of raw integrated density of ankydrin D1 protein expression in the nucleus and in the cytoplasm in hiPSC-CMs 72 hours after transfection with hsa-miR-548v or miRNA negative control.

We assessed global transcriptome changes by deep sequencing hiPSC-CMs RNA after transfection with hsa-miR-548v versus miRNA negative control (Figure 5A). The clustering analysis revealed distinct profiles between groups (Figure 5B and Supplemental Figure 5, A and B) with 645 downregulated transcripts (FPKM > 1 and > 2.0-fold downregulation) and 365 upregulated transcripts (FPKM > 1 and > 2.0-fold upregulation) (Figure 5C and Supplemental Tables 1–3). Metascape and gene set enrichment analysis (GSEA) revealed that the 645 most downregulated genes were enriched in cardiovascular networks as “heart development” or “circulatory system process” (Supplemental Figure 6A), in line with a previous in silico study indicating a significant association between members of miR-548 family and cardiovascular system development (20). We first observed that *NPPB* (encoding for the natriuretic peptide B, a well-known hormone secreted by cardiac ventricular myocytes in response to myocardial stretch) was the most downregulated gene in response to hsa-miR-548v (log_2_ fold change, –4.02; *q* value = 4.8 × 10^–17^; Figure 5C and Supplemental Figure 5D). We further analyzed the data sets of downregulated transcript to assess sets of genes encoding for CMs’ intracellular components that typically contribute to myocardial elasticity (i.e., calcium handling, microtubule network, filaments, and cytoskeletal proteins) (Figure 5D and Supplemental Figure 5, C–K). We found a significant downexpression of 2 important mechanosensors, cardiac ankyrin repeat proteins *ANKRD1*/CARP1 (log_2_ fold change, –2.76; *q* value = 1.6 × 10^–16^) and *ANKRD2*/CARP2 (log_2_ fold change, –3.06; *q* value = 4.6 × 10^–8^) (Figure 5C and Supplemental Figure 5, F and G), as well as *DESMIN* (log_2_ fold change, –1.57; *q* value = 3.2 × 10^–12^) (Supplemental Figure 5E), the predominant intermediate filament. CARPs are highly expressed in CMs, where they interact with the intermediate filament (desmin) proteins, acting as important regulators of the stretch-sensing machinery, consistent with our stretching experiments. ANKRD1/CARP1 was the predominant isoform expressed in hiPSC-CMs (Supplemental Figure 5, F and G). At the individual level, other components showed lower differences between groups (Figure 5, C and D, and Supplemental Figure 7). Further analyses for enriched canonical pathways revealed a downregulation of multiple members and regulators of the MAPK signaling cascade (Supplemental Figure 6B), which typically serve as specialized transducers of stress response.

Following these transcriptomics analyses, we assessed ANKRD1/CARP1 expression in hiPSC-CMs before and after treatment with hsa-miR-548v. ANKRD1/CARP1 expression was detectable in hiPSC-CMs under basal conditions but was significantly decreased in response to hsa-miR-548v, with a significant reduction in expression at 48 and 72 hours after treatment (Figure 5, E and F). Immunostaining experiments in hiPSC-CMs transfected with miRNA negative control revealed a dual subcellular localization of ANKRD1/CARP1, both in the nucleus and in the cytoplasm, with a striated pattern consistent with ANKRD1/CARP1 localization at the I-band of the sarcomere (Figure 5G), as previously reported (29). We further quantified the subcellular localization and found a significant decrease in ANKRD1/CARP1 localization in the cytoplasm after treatment with hsa-miR-548v, while its localization to the nucleus was maintained (Figure 5, H and I). While the hiPSC-CMs transfected with hsa-miR-548v shows striated staining of TroponinT (Figure 5G), the striated pattern of ANKRD1/CARP1 was strikingly reduced, suggesting a decreased localization of ANKRD1/CARP1 to the sarcomeres (Figure 5, G and H).

Overall, these data indicate that hsa-miR-548v dysregulated multiple targets, including structural components implicated in the transmission of mechanical forces and the resistance to cyclic deformation. The exogenous administration of hsa-miR-548v in CMs notably results in a reduction of ANKRD1/CARP1 expression and localization at the sarcomeric I-band, suggesting a lower binding to the sarcomere components and a more relaxed state of the myofibrils.

### hsa-miR-548v improves cardiac parameters in a hypertrophic cardiomyopathy model.

**Figure 6:**
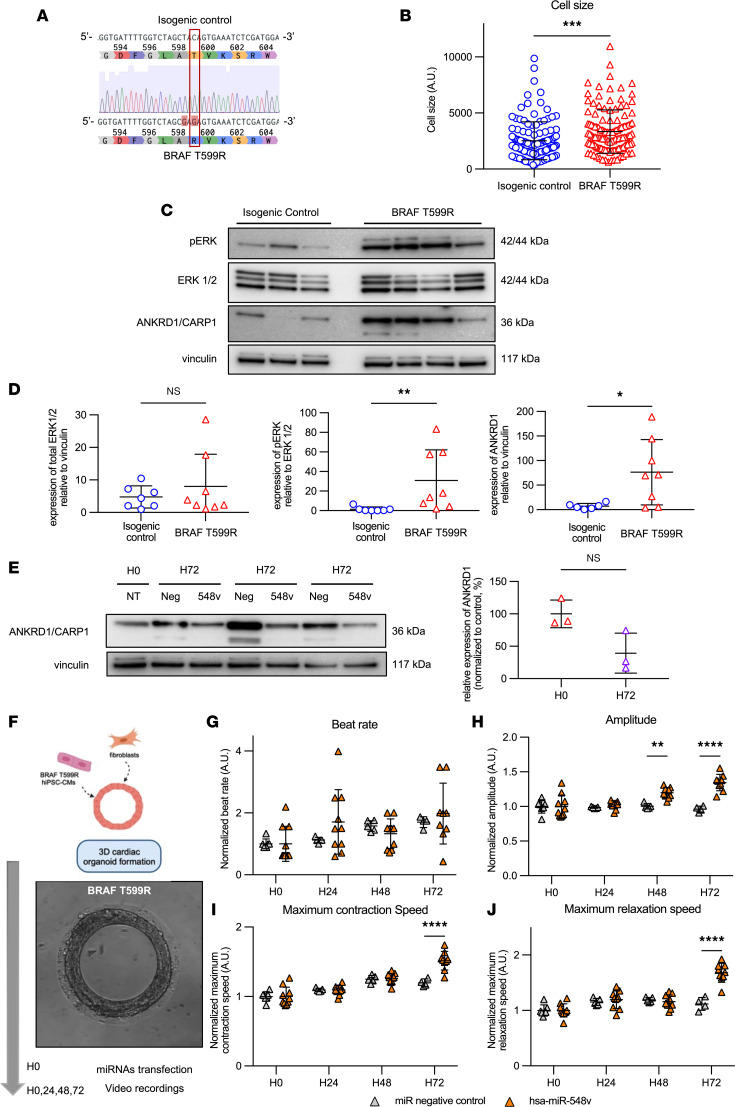
Effect on hsa-miR-548V on BRAF T599R mutated hiPSC-CMs. (**A**) Sequence alignment showing the *BRAF* 1796C>G nucleotide substitution leading to the nonsense T599R mutation (red square). Of note, the 1794T>G silent variant was introduced for the CRISPR/Cas9 processing. (**B**) Comparison of hiPSC-CMs between isogenic control (*n* = 124, 5 independent experiments) and BRAF T599R (*n* = 127, 6 independent experiments) cell lines. ***P = 0.0004, unpaired *t* test. (**C**) Representative Western blot for ERK phosphorylation (pERK) and ankyrin repeat domain 1 protein (CARP1) expression on hiPSC-CMs from BRAF T599R and its isogenic control. Vinculin was used as a loading control. (**D**) Quantification of total ERK1/2 normalized to vinculin (left) and quantification of pERK relative to ERK1/2 expression level (middle) in BRAF T599R (*n* = 8) cells and the isogenic control (*n* = 7). **P = 0.0012, Mann-Whitney *U* test. Quantification of ankyrin D1 level expression (right) in BRAF T599R (*n* = 8) and the isogenic control (*n* = 6) *P < 0.05, Mann-Whitney *U* test. (**E**) Western blot for ankyrin repeat domain 1 protein on BRAF T599R cells (*n* = 3) 72 hours after transfection with miRNA negative control or hsa-miR-548v. Quantification of ANKRD1 relative level expression in BRAF T599R cells 72 hours after transfection, normalized to the basal expression before transfection, and expressed as ratio between hsa-miR-548v treated cells/miRNA negative control–treated cells. *P* = 0.10, Mann-Whitney *U* test. (**F**) Schematic overview of hECT generation and representative bright-field image of tissues obtained using the BRAF mutant hiPSC-CMs. (**G**–**J**) Beat rate (**G**), amplitude (**H**), maximum contraction speed (**I**), and maximum relaxation speed (**J**) of BRAF T599R engineered cardiac tissues at 0, 24, 48, and 72 hours after transfection with miRNA negative control (*n* = 7) or hsa-miR-548v (*n* = 10), from 1 differentiation. **P < 0.01, ****P < 0.0001 from Šídák post hoc comparisons between treatment groups, 2-way ANOVA.

We finally evaluated the therapeutic potential of hsa-miR-548v in improving the relaxation kinetics of diseased human CMs. We used CRISPR/Cas9 genome editing to insert the T599R activating *BRAF* mutation into the Control-1 hiPSC line (Figure 6A). *BRAF* encodes a serine/threonine kinase that is a direct effector of Ras, and the T599R activating *BRAF* mutation is associated with aberrant activation of the Ras/MAPK pathway, resulting in a hypertrophic cardiomyopathy (HCM) phenotype. Consistently, the *BRAF* mutant hiPSC-CMs showed increased cell size in 2D monolayers (Figure 6B) and a hyperphosphorylation of the ERK1/2, the downstream effector of the MAPK pathway (Figure 6, C and D). We also found that ANKRD1/CARP1 was overexpressed in *BRAF* mutant hiPSC-CMs as compared with control hiPSC-CMs (Figure 6, C and D). We then evaluated the effect on hsa-miR-548v exogenous transfer in the diseased hiPSC-CMs and observed a nonsignificant trend for a reduction in ANKRD1/CARP1 expression in the BRAF mutant hiPSC-CMs treated with hsa-miR-548v (Figure 6E), getting closer to the expression observed in isogenic controls. We next evaluated the effect on hsa-miR-548v exogenous transfer into 3D ring-shaped hECT formed with BRAF mutant hiPSC-CMs (27), generated using the same experimental procedures as previously used with healthy hiPSC lines (Figure 2, A and B; Figure 6F; and Supplemental Video 7). We found that treatment with hsa-miR-548v induced a progressive and significant improvement in relaxation rate, as well as in contractile parameters (i.e., amplitude of contraction and contraction speed) in *BRAF* mutant hiPSC-CMs as compared with controls (Figure 6, G–J), thus reproducing the lusitropic effect of hsa-miR-548v in disease condition.

## Discussion

To the best of our knowledge, this is the first demonstration that the relaxation of human CMs can be stimulated and improved by the exogenous administration of miRNAs. Among the different candidates identified in the high-content miRNA screening, hsa-miR-548v provided strong and reproducible effects at the cellular and engineered tissue levels, in different healthy and diseased cell lines. These effects were primarily apparent as an increase in relaxation velocities with a consequent trend for higher developed force.

hsa-miR-548v is a poorly explored miRNA that is part of a large primate-specific miRNA family. This explains why its cardiac effects were not previously identified in small and nonprimate animal models of heart failure. The different miRNA expression analyses indicate a low expression in adult organs and the lack of expression in healthy adult human CMs, as confirmed in our hiPSC-CMs lines. hsa-miR-548v is likely expressed during development of the fetal heart (22, 30), and functional network analyses on targets of the miR-548 family have shown their possible involvement in the cardiovascular system development (20). Little is known about the role of miR-548 family in diseases, but a downregulation of at least 10 miR-548 family members was identified by genome-wide analysis on PBMCs from patients with heart failure with reduced ejection fraction (20). Our results suggest that hsa-miR-548v is likely a fetal miRNA that can exert a beneficial effect as reexpressed in adult human CMs.

As with other miRNAs, the significant effect on CM relaxation and viscoelastic properties induced after hsa-miR-548v transfer likely ensues from the sum of their multiple effects on multiple individual targets. Our investigations, however, delineate a potential mechanism that does not involve significant changes in calcium handling (at least under resting and basal conditions) but rather in the mechanical coupling at the levels of cytoskeletal networks. We observed a coherent reduction in different members of the mechanosensing machinery. *DESMIN* and CARPs (encoded by *ANKRD1* and *ANKRD2*) appeared as highly downregulated genes in response to hsa-miR-548v exogenous transfer. CARPs are highly expressed in CMs, where they interact with sarcomeric (titin and myopalladin) and intermediate filament (desmin) proteins, acting as important regulators of the stretch-sensing machinery. Overexpression of *ANKRD1* has recently been reported in the failing human myocardium (31), and CM-specific *Ankrd1* overexpression leads to progressive diastolic dysfunction in mice (32). Some *ANKRD1* mutations have been found in patients with HCM and diastolic dysfunction (29, 33). ANKRD1/CARP1 displays a dual subcellular localization (in the nucleus or in the cytoplasm at the sarcomeric I-band), and an increased localization to the cytoplasm involves higher binding to the cytoskeleton, changes in response to stretch, and activation of pathological stress-response pathways (29, 33). We strikingly found that hsa-miR-548v exogenous transfer resulted in a decrease in ANKRD1/CARP1 expression, associated with a reduced localization at the I-band. ANKRD1/CARP1 is able to bind Titin N2A in the I-band region (34) as well as Actin filament (35). CARP1 is likely regulating titin’s extension and stiffness (34), but the significance of the CARP1/titin/desmin interaction to CMs’ elasticity and, consequently, to diastolic tension is undetermined. Our results strongly suggest that the sarcomeric I-band is a mechanical controlling hub of CMs’ ability to relax and that disturbances in *Z* disc mechanosensitive proteins can be involved in impaired relaxation and diastolic dysfunction (17, 36).

The mechanical response to stretch of the myocardium is a critical determinant of cardiac performance in healthy and pathological hearts (36). Previous studies have demonstrated that viscous forces play a significant role in resisting diastolic filling and are considerably increased in multiple etiologies of human heart disease, especially in patients with LV hypertrophy, as frequently observed in HFpEF patients (7). Recent studies have reported an increase in the passive tension and the viscosity of failing CMs (8). While our measurement protocols are similar, the nonlinearity properties of the myocardium and CMs make the results difficult to compare. While most of previous studies have performed measures on isolated trabeculae, we here performed stretch-response measure on isolated rod-shaped hiPSC-CMs. Consequently, the range of stress developed is not comparable between studies, with lower levels recorded in our study (0.2–2 kPa), whereas stress recorded in human heart extracts varied from 10 kPa to more than 30 kPa for failing myocytes. This gap could be partly explained by the difference between the 2 models. In addition, the range of elongation is different between studies. In our study, cells were tolerant to a stretch up to 25% elongation, as adult CMs were typically stretched up to 12% elongation (8). Lastly, we performed measures in an original model based on CMs derived from healthy iPSC, and this might not exactly represent the mechanical properties from CMs isolated from elderly patients’ hearts.

Another important finding of our study remains the therapeutic potential of hsa-miR-548v exogenous administration to human CMs. Our study was designed as a functional screening approach using a library of miRNA mimics in order to decipher regulators of human CM relaxation. Our results reveal the strong effect of a specific miRNA that does not appear to be expressed in adult CMs but that robustly improved relaxation velocities after exogenous administration in both healthy and diseased hiPSC lines. hsa-miR-548v can, thus, appear as a promising positive lusitropic agent. The modeling of abnormal cardiac relaxation as seen in humans poses a major challenge for cardiac researchers. The use of small animals is limited by important differences in baseline heart rate, LV loading pressures, and consequent changes in molecular events (37). Our study was performed on the premise that human CM relaxation is best characterized using human-based models, and this may also have some limitations. The optimal conditions to model abnormal relaxation, or eventually HFpEF, with hiPSC-CMs remain to be defined. Therefore, we decided to use a genetic strategy and selected the T599R activating *BRAF* mutation as a prototypical cause of HCM, a condition that is associated with marked LV diastolic dysfunction. Recently, it has been reported that a subset of patients (~5%) with primary HCM develop advanced heart failure due to severe LV diastolic dysfunction, while the systolic function is preserved (38). The improvement of relaxation velocities in the BRAF mutant hiPSC-CMs suggests an interesting therapeutic potential for patients with HCM who have diastolic dysfunction, but this will require additional in vitro and in vivo experiments in order to establish the pharmacological profile of hsa-miR-548v, ensure its delivery to the CMs, and demonstrate its efficacy in vivo.

Our study has limitations that are mainly linked to the models used. In order to investigate CM relaxation in a human setting, we used hiPSC-CMs, which might not fully recapitulate the characteristics of isolated adult CMs from normal human hearts (an inaccessible resource for our HTS campaign). Nevertheless, our results were reproduced with different hiPSC-CM lines, from both sexes, and in different 2D and 3D configurations. We also developed original culture platforms (3D culture and micropatterning) to enhance CM maturation, and we were still able to recapitulate the effect of hsa-miR-548v identified in the 2D monolayer model. Other techniques and methods to characterize myocardial stretch response may compromise elements that contribute to viscoelasticity (e.g., skinned or permeabilized preparations), a limit that was not applicable to our experimental strategy. The myocardium is a nonlinear viscoelastic material, and it is also difficult to extrapolate the hemodynamical effect of hsa-miR548v on fully formed hearts. hsa-miR-548v is primate specific, thus also challenging its relevance in small-animal models as well as pharmacological investigations in preclinical models. Hence, the ongoing development of engineering heart chambers from human iPSC could extend our functional studies and assess the effect of the exogenous transfer of hsa-miR-548v on hemodynamics parameters (39).

In conclusion, our study uncovers a miRNA (i.e., hsa-miR-548v) that shows a strong and reproducible increase in relaxation rates in healthy and diseased hiPSC-CMs. This positive lusitropic effect results from the sum of multiple effects on different individual genes, with a more specific effect on proteins of the mechanical stretch-sensing system. In particular, the mechanism involves a decrease in ANKRD1/CARP1 expression, associated with its reduced localization at the I-band, which provides a favorable viscoelastic environment for relaxation and force generation of human CMs.

## Methods

### High-throughput imaging assays using hiPSC-CMs

We used commercially available hiPSC-CM from FUJIFILM Cellular Dynamics (iCell Cardiomyocytes²). Cells were thawed according to the manufacturer’s recommendations in 384-wells plates (PerkinElmer) on a 10 μg/mL fibronectin coating (F1141, MilliporeSigma). Media were changed 4 hours after seeding and every 2 days until transfection. Seeding and media changes were performed by a Zephyr Liquid Handler from Perkin Elmer.

Cells were transfected with the mirVana micro-RNA Mimic Library (Predefined Human v21, 4464074, Thermo Fisher Scientific) using Lipofectamine RNA iMax (13778-150, Thermo Fisher Scientific) in OptiMEM medium (11058-21, Thermo Fisher Scientific). This library is composed of 2,565 mimic human miRNAs. We used 25 nM as the final concentration of miRNAs. Twenty-four hours after transfection, medium was completely changed, and 72 hours after transfection, bright-field imaging of the cells was performed with an automated high-content screening system (CellVoyager CV8000, Yokogawa) with bright-field at 37°C and 5% CO_2_. A 10-second movie was recorded for each well at a framerate of 37 images per second using bright-field light microscopy with a binning of 2, generating 500 × 500 pixel images.

To analyze raw image sequences of hiPSC-CM contraction/relaxation, we implemented an optical vector flow script (40). The magnitude of each vector was then computed and integrated over the entire image, providing a total contraction amplitude per frame. The final signal was then created using the 357 amplitude values sequentially.

Plates’ optical vector flow signals were analyzed with a custom-made Matlab script (MatWorks). In this script, several readouts are extracted: the amplitude of contraction, the beating frequency, maximum and mean velocities, the contraction-time integral (AUC), the time to contract from 10% to 90% of amplitude, the time to relax from 90% to 10%, and the peak duration.

We analyzed plates results with the open access software HCS analyzer (41). The assay was performed in 3 independent replicates. Hits were selected according to their *Z* score on the 3 replicates: hits were validated when the *Z* score was above 2 in at least 2 replicates and the mean *Z* score of the 3 replicates above 2. *Z* score was calculated with the following equation:
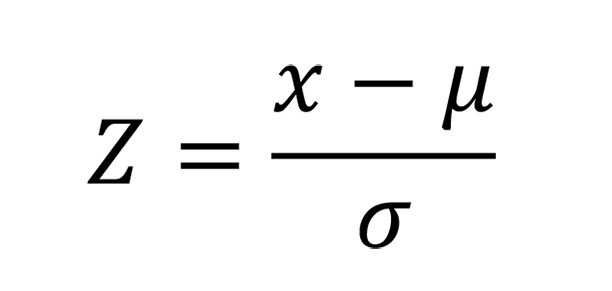


where μ is the mean of the mean relaxation velocity on the plate, x is the mean relaxation velocity of the miRNA, and σ is the SD of the plate.

### hiPSC-CM–based ECT

#### hiPSC culture and differentiation.

For 3D cultures, we used 3 hiPSC healthy lines: the SKiPSC-31.3 (Control-1), which was derived from human dermal fibroblasts from a healthy 45-year-old volunteer as previously published (42), and 2 lines (P11014 [Control-2] and P11007 [Control-3]), which were derived from healthy female and male donors, respectively (24). We also used a CRISPR/Cas9 generated BRAF T599R hiPSC line. hiPSCs cells were seeded on Matrigel and cultured in mTeSR1 medium (Stemcell Technologies). When hiPSCs reached a confluency of 70%–80%, cells were passaged in clumps by scraping with a pipette tip. A medium change was performed every 24 hours. Cultures were maintained at 37°C in a humidified incubator with 5% CO_2_. The hiPSC line used in this study was assessed for pluripotency and routinely tested for mycoplasma.

Total RNA was extracted using NucleoSpin RNA Kit (Macherey-Nagel), and cDNA was synthesized by SuperScript IV VILO kit (Thermo Fisher Scientific). Pluripotency gene expression was assessed by quantitative PCR (qPCR) and normalized to RPL32.

For immunostaining, hiPSCs cells were fixed in 4% paraformaldehyde for 10 minutes at room temperature, permeabilized in blocking solution (PBS 2% BSA, 0.5% Triton) for 1 hour, and stained with primary antibodies: Nanog (4903, Cell Signaling Technology; 1:200) and Tra1.60 (MAB4360, MilliporeSigma; 1:100) overnight at 4°C. Then, cells were washed 3 times with PBS and incubated 1 hour with secondary antibodies and DAPI for nuclear staining, at room temperature. Images were acquired with an EVOS imaging Systems (Thermo Fisher Scientific).

Once confluent, the hiPSC cells were differentiated into CMs using a small molecule–modulated differentiation and glucose starvation (43). Briefly, mTeSR1 medium (Stemcell Technologies) was changed by RPMI supplemented with B27 without insulin (Thermo Fisher Scientific) and 6 μM CHIR-99021 (Abcam), and it was maintained in a 37°C and 5% CO_2_ incubator for 48 hours. The medium was changed to RPMI-B27 without insulin for 24 hours and then to RPMI-B27 without insulin supplemented with 5 μM IWR-1 (MilliporeSigma) for 48 hours. On day 5, the medium was changed back to RPMI-B27 without insulin for 48 hours. From day 7 onward, cells were placed in RPMI-B27 with insulin and media change every 2 days. At day 11, the medium was changed to low-glucose medium for 3 days. CMs were then replated in RPMI-B27 with insulin. At day 15, medium was changed for a second glucose deprivation for 3 more days. Starting from day 18, medium was changed every 2 days with RPMI-B27 with insulin.

To assess the differentiation efficiency, at day 21, cells were strained with an APC anti–cardiac TroponinT (TNNT2) antibody (130-106-689, Miltenyi Biotec; 1:100) or APC isotype control (130-104-615, Miltenyi Biotec; 1:100) and analyzed by flow cytometry.

### Generation of T599R BRAF hiPSC line with CRISPR/Cas9

#### Preparation of the CRISPR/Cas9-BRAF sgRNA plasmid.

To introduce the *BRAF* (C1796G) mutation into the control line SKiPSC-31.3 (Control-1) with CRISPR/Cas9, we designed a homologous sgRNA and single-stranded oligo donor single-stranded oligodeoxynucleotides (ssODN) (Supplemental Table 3) that targets the DNA regions close to C1796 in *BRAF* exon 15. The sgRNA was cloned into pSpCas9(BB)-2A-Puro V2.0 (PX459, gift from Feng Zhang; Addgene plasmid #62988; http://n2t.net/addgene:62988; RRID:Addgene_62988). Homology directed repair (HDR) ssODN template was 127 pb long (Supplemental Table 3) and contained the mutated allele for mutation insertion together with an adjacent silent Ala598Ala substitution for recombinant events tracking and to minimize recutting of the recombinant allele.

#### hiPSC electroporation.

The hiPSC cells at approximately 80% confluency were dissociated with Accutase (Thermo Fisher Scientific) and washed 1× with PBS. Approximately 2 × 10^6^ hiPSCs were resuspended in 82 μL human stem cell nucleofector solution and 18 μL supplement 1 (Lonza). In total, 20 μg of sgRNA-Cas9 plasmid and 30 μg of ssODN were added, and the mixture was transferred into an electroporation cuvette and nucleofected using program A-013 on the Amaxa 2B nucleofector (Lonza). The electroporated cell suspension was dispensed into 6-well plates containing 2 mL of cloning medium (CloneR diluted into mTeSR1 media; Stemcell Technologies) supplemented with 10 μM of ROCK inhibitor Y-27632 (72304, Stemcell Technologies), in order to facilitate cell recovery.

The next day, cloning media were replaced with fresh mTeSR1 medium containing 0.5 μg/mL of puromycin (P9620, Sigma-Aldrich), which was maintained for 48–72 hours. Puromycin-resistant cells were maintained in culture with mTeSR1 medium and changed every 2 days until clonal colonies could be observed.

#### Clone isolation and screening.

After approximately 1 week, single rounded colonies were picked under a dissection microscope using sterile pipette tips to a new Matrigel-coated 4-well plate in mTeSR1 media; 50% of the cells were used for genome extracting and screened by sequencing (Eurofins genomic) of PCR products spanning the target site (Supplemental Table 3). Analyses of Sanger traces were done using SnapGene software. Briefly, sequences were aligned with WT sequences to confirm the successful edition to generate mutated clones. Positives clones were kept and amplified for further experiments. Genome integrity was confirmed by the iCS-digital PSC test (Stem Genomics) (44). We did not detect genomic abnormalities with these analyses.

#### Human fibroblasts culture.

We use a commercially available human fibroblast cell line from Lonza (CCC2511, lot 4888388). Fibroblasts were cultured in T75 flasks and maintained in DMEM supplemented with 10% FBS and 1% penicillin-streptomycin. Cells with low passage number (passage number < 7) were used.

#### hiPSC-CMs ring-shaped 3D cardiac organoids.

After 21 days of differentiation hiPSC-CMs were dissociated with enzymatic digestion (130-110-204, Miltenyi Biotec) and fibroblasts were collected after 5 days in culture with TrypLE Express Enzyme (12605028, Thermo Fisher Scientific). A ratio of 3:1 hiPSC-CMs/fibroblast were mixed and then seeded in molds provided from 4DCell (SmartHeart). After 2 weeks of culture in an enriched calcium medium (DMEM high glucose, 2.3 mM CaCl_2_,10%FBS, 0.1% penicillin-streptomycin), cardiac organoids were transfected with 25 nM of miRNA (miRNA negative control or hsa-miR-548v) in OptiMEM using Lipofectine RNAiMAX. Medium was then changed every day of recording. Cardiac organoids were recorded for 10 seconds in bright-field imaging with a high-speed CCD camera (PL-D672MU, Pixelink) mounted on a microscope (Primovert, Zeiss), at a 10× magnification. Analysis of cardiac organoids was performed thanks to a custom Matlab script (27). Each tissue raw data was normalized on the same tissue raw data before transfection. Data before transfection (0 hours) are normalized on the mean of all the data before transfection (24, 48, 72 hours).

#### hiPSC-CM–based engineered cardiac macrotissues.

To build ECT, we prepared a mix per tissue of 1.2 million hiPSC-CMs, 0.3 million fibroblasts, 2 mg/mL collagen I (354249, Corning), and 0.9 mg/mL Matrigel (356231, Corning) in a HEPES/MEM media. On day 22 of differentiation, CMs were dissociated with enzymatic digestion (130-110-204, Miltenyi Biotec) and fibroblasts with TrypLE Express Enzyme (12605028, Thermo Fisher Scientific).

The cell-matrix mix (100 μL/mold) was seeded in a flexible PDMS mold and placed at 37°C and 5% CO_2_ (26, 45, 46). After 2 hours, ECTs were fed with DMEM supplemented with 10% FBS, 1% penicillin-streptomycin, and a calcium concentration of 2.3 mM. Medium was changed every 2 days. After 13 days of culture, contractile forces were measured just before transfection. Forward transfection was performed using 25 nM of miRNA (miRNA negative control or hsa-miR-548v) in OptiMEM using Lipofectine RNAiMAX. Medium was changed 24 hours after transfection, and contractile forces were measured 72 hours after transfection.

Contractile force measurements were captured with a high-speed CCD camera (PL-D672MU, Pixelink) while custom LabVIEW software developed by K. Costa’s lab (46) tracked the centroid movement of the tips of the flexible posts. Force was converted from the deflection of the PDMS posts by an elastic beam-bending equation (46). A custom MATLAB script, similar to that developed for the HTS campaign, was used to extract several readouts, including the developed force and mean relaxation velocity.

#### qPCR.

RNA and miRNAs were extracted from ECTs using QIAzol lysis Reagent and purified with the miRNeasy mini kit (217004, Qiagen), as per the manufacturer’s instructions. Then, 10 ng of extracted RNA and miRNAs was subjected to reverse transcription using the miRCURY LNA RT Kit (339306, Qiagen) as per the manufacturer’s instructions. The resulting cDNA was subjected to qPCR using SYBR Select Master Mix (4472908, Applied Biosystems) on Quant Studio 3 Real-Time PCR system (Thermo Fisher Scientific) as per the following condition: 95°C for 2 minutes, 40 cycles of 95°C for 10 seconds and 56°C for 1 minute, followed by 95°C for 10 seconds and 60°C for 1 minute. The relative expression of hsa-miR-548v was calculated using the ΔCt method. The ΔCt was calculated by subtracting RNU1A1 Ct from hsa-miR-548v Ct, whereas ΔΔCt was obtained by subtracting the mean ΔCt of ECT transfected with miRNA negative control from ΔCt of the sample.

### hiPSC-CM single-cell distensibility measurements

#### hiPSC-CM micropatterning.

To promote rod-shaped CMs, we used micropatterning technology (4DCell). Thirty-five days after differentiation, CMs were seeded in micropatterned coverslips with a rectangular shape (custom-made, size: 120 × 30 μm). The micropatterned substrate allows cells to adhere only on micrometer-sized defined region. Cells were cultured for 5 days on micropatterned slides before forward transfection.

#### Distensibility measurements.

Seventy-two hours after transfection, cells were stretched in order to evaluate their distensibility properties, using the MyoStretcher system from IonOptix. This system is composed of 2 micromanipulators connected to an optical force transductor (OptiForce, IonOptix) and a piezoelectric length controller (motor). Cells were stretched to different lengths in a staircase protocol.

#### Cell attachment procedure.

Micropatterned cells were enzymatically dissociated with type II collagenase (50 U/mL) for 20 minutes at 37°C. The cell was glued on the MyoStretcher’s tips using a biological adhesive material (Myotak, IonOptix) at its 2 distal edges. To fully detach the cell from the slide, we used slight lateral movements as previously described (28) and lifted the cell to start staircase protocol.

### Transcriptome-wide analysis

To perform RNA-Seq, we used iCell CMs² from FCDI. After 6 days of culture, we performed forward transfection of miRNA and extract RNA 3 days after transfection. Mapping statistics are presented in Supplemental Figure 6.

#### Quantification of gene expression.

STAR was used to obtain the number of reads associated to each gene in the Gencode v31 annotation (restricted to protein-coding genes, antisense, and lincRNAs). Raw counts for each sample were imported into R statistical software. Extracted count matrix was normalized for library size and coding length of genes to compute FPKM expression levels.

#### Unsupervised analysis.

The Bioconductor edgeR package was used to import raw counts into R statistical software and compute normalized log_2_ counts per millions (CPM) of mapped reads using the weighted trimmed mean of M-values (TMM) as normalization procedure. The normalized expression matrix from the 1,000 most variant genes (based on SD) was used to classify the samples according to their gene expression patterns using principal component analysis (PCA) (Supplemental Figure 4A), hierarchical clustering and consensus clustering. PCA was performed by FactoMineR:PCA function with “ncp = 10, scale.unit = FALSE” parameters. Hierarchical clustering was performed by stats:hclust function (with euclidean distance and ward.D method). Consensus clustering was performed by ConsensusClusterPlus function to examine the stability of the clusters. We established consensus partitions of the data set in K clusters (for K = 2–8), on the basis of 1,000 resampling iterations (80% of genes, 80% of sample) of hierarchical clustering, with euclidean distance and ward.D method. Then, the cumulative distribution functions (CDFs) of the consensus matrices were used to determine the optimal number of clusters (K = 3 for instance), considering both the shape of the functions and the area under the CDF curves.

The t-distributed stochastic neighbor embedding (t-SNE) analysis was performed with the Bioconductor Rtsne package applied to the PCA object (theta = 0.0, perplexity = 2, maximal iterations [max_iter] = 1,000).

#### Differential expression analysis.

The Bioconductor edgeR package was used to import raw counts into R statistical software. Differential expression analysis was performed using the Bioconductor limma package and the voom transformation. To improve the statistical power of the analysis, only genes expressed in at least 1 sample (FPKM ≥ 1) were considered. A *q* value threshold of ≤ 0.05 and a minimum fold change of 2 were used to define differentially expressed genes. The enrichment for gene sets and canonical pathways (KEGG and GO terms) on significantly downexpressed genes was analyzed using the metascape web tool (47).

### Calcium transient analysis

After imaging cells with the HTS system, cells were loaded with Fluo-4 Direct Calcium Assay kit (F10471, Thermo Fisher Scientific, 0.5× final concentration). Then, cells were incubated 30 minutes at 37°C followed by 30 minutes at room temperature. Calcium imaging was performed using the functional drug screen system (FDSS) from Hamamatsu for 2 minutes. Signals analysis was then performed using Waveform Analysis Software from Hamamatsu.

### Immunostaining and imaging

After 7 days of culture, hiPSC-CMs were sequentially fixed between days 30 and 35 with 4% paraformaldehyde (PFA) (1573590, Electron Microscopy Sciences) for 10 minutes and then permeabilized and blocked with 0.5% Triton X-100 (T-8787, MilliporeSigma) and 2% BSA (001-000-162, Jackson ImmunoResearch) in PBS (blocking solution) for 1 hour. Subsequently, primary antibody incubation was performed overnight at 4°C in 1:10 diluted blocking solution: Cardiac-TroponinT (ab45932, Abcam; 1:500), α-actinin (A7811, Sigma Aldrich; 1:500), ANKRD1/CARP1 (sc-365056, Santa Cruz; 1:50). After washing, cells were incubated with secondary antibodies goat anti–mouse IgG conjugated to Alexa Fluor 488 (A10680, Thermo Fisher Scientific; 1:500) or anti–rabbit Alexa Fluor 546 (A11010, Thermo Fisher Scientific; 1:500), and DAPI (Thermo Fisher Scientific). After incubation, coverslips were mounted with Dako Faramount Aqueous Mounting (S3025, Agilent). Fluorescence images were captured on a Leica SPE confocal microscope at 63× objectives as appropriate. Image processing and analysis were performed using ImageJ (NIH).

### Microscopy and image analysis

#### Cell size analysis.

Cells were stained with WGA (FL-1021, Vector Laboratories) and fluorescence images were captured on a Leica SP8 confocal microscope at 40× objective. Image processing and analysis were performed using ImageJ. Cell size measurements were done using 1 stack per cells; cell membranes were then cut out manually and saved as ROI to extract their area.

#### ANKRD1/CARP1 signal quantification.

After acquisition with a Leica SPE confocal microscope at 40× objective, images from hiPSC-CMs were processed and analyzed using ImageJ. A Weka segmentation has been used to segment the DAPI signal, corresponding to the nucleus area and the ANKRD1/CARP1 signal corresponding to the whole cell signal. To obtain the signal of ANKRD1/CARP1 in the cytoplasm, nucleus area was removed from the image and a new WEKA segmentation was performed. Raw integrated density was then measured for each hiPSC-CM and each segment.

### Western blot

hiPSC-CMs were cultured in monolayers until 28 days of differentiation. At day 28, cells were transfected with miRNA negative control or hsa-miR-548v (25 nM of miRNA in OptiMEM using Lipofectine RNAiMAX). After 24, 48, or 72 hours, cells were dissociated with enzymatic digestion (130-110-204, Miltenyi Biotec), and dry pellets were then stocked at –80°C until protein extraction. See complete unedited blots in the supplemental material.

Total proteins were extracted using RIPA buffer supplemented with a protease and phosphatase inhibitor cocktail (MilliporeSigma), followed by BCA quantification (23227, Thermo Fisher Scientific). In total, 10 μg of each sample was subjected to electrophoresis on NuPAGE 4-12% Bis-Tris gradient gels (NP0335BOX, Invitrogen), and protein was transferred to nitrocellulose membranes using wet transfer (Bio-Rad). After drying, protein amounts were controlled with a Ponceau S staining. After blocking with 5% nonfat milk, the membranes were incubated overnight at 4°C with primary antibodies: mus musculus (msc) anti-ANKRD1/CARP1 (sc-365056, Santa Cruz Biotechnology Inc.; 1:500), msc anti-pERK (9106S, Cell Signaling Technology; 1:2,000), and rabbit (Rb) anti-ERK 1/2 (9102S, Cell Signaling Technology; 1: 1,000). Vinculin (V9131, MilliporeSigma; 1: 5,000) was used as loading control protein. Secondary antibodies used were HRP anti-mouse (715-035-150, Jackson ImmunoResearch), HRP anti-rabbit (111-035-144, Jackson ImmunoResearch). Membranes were incubated with chemiluminescent substrate (West Pico PLUS or West Femto, Thermo Fisher Scientific) for 5 minutes, and luminescence was detected using the Amersham ImageQuant 800. Densiometric analysis of the immunoblots was performed using Image Lab software.

### Statistics

Statistical analysis was performed using GraphPad Prism 10.0.2 and the R environment. Data are presented as mean ± SD. Differences between experimental groups were analyzed with the appropriate statistical tests, according to data distribution and experimental design, as specified in each figure legend. *P* < 0.05 were considered statistically significant. Comparisons were made using Mann-Whitney *U* test or Student 2-tailed *t* test for comparison between 2 groups as appropriate. Two-way ANOVA was used for comparison between > 2 groups or with mixed effect models in case of repeated measures, with post hoc comparisons (Šídák method). Statistical analyses were performed with GraphPad Prism software (Version 10).

### Study approval

Samples from donors were collected in subjects who gave their written informed consent prior to the participation in the study. The corresponding studies were reviewed and approved by IRBs (IRB of Staten Island University Hospital, Staten Island, New York, USA, for the first donor and IRB CPP Ile de France XI 11-015, Paris, France, for the 2 other donors).

### Data availability

The RNA-Seq data are available in the NCBI GEO, accession no. GSE250164. All supporting data for each figure panel are reported in the Supporting Data Values file. Any additional information is available from the corresponding author upon request.

## Author contributions

JSH designed research studies and supervised the work with the help of PB; EV conducted the screening experiments with the help of SL and with supervision by MECM and TD; EV and SN conducted the functional experiments in iPSC with the help of CJ, ARV, and CP; motion analyses were performed by TD, with assistance of EV; SN, MS, and EV acquired data on ECT; EV, SN, and JSH analyzed data; and JSH and EV wrote the manuscript with the assistance of PB and SN and the contribution of all coauthors.

## Supplementary Material

Supplemental data

Supplemental tables 1-3

Supplemental video 1

Supplemental video 2

Supplemental video 3

Supplemental video 4

Supplemental video 5

Supplemental video 6

Supplemental video 7

Supporting data values

## Version 1 01/02/2024

In-Press Preview

## Version 2 02/08/2024

Electronic publication
