# Supplementary material for: hsa-miR-548v controls the viscoelastic properties of human cardiomyocytes and improves their relaxation rates
Source: JCI Insight. 2024 Jan 2;9(3):e161356. doi: 10.1172/jci.insight.161356 (PMC11143964; doi:10.1172/jci.insight.161356)

Supplemental Figures, videos and tables

hsa-miRNA-548v controls the viscoelastic properties of human cardiomyocytes and improves their relaxation rates

Eva Vermersch[1,2] PhD, Salomé Neuvendel[1], Charlene Jouve[1], Andrea Ruiz Velasco[1], Céline Pereira[1], Magali Seguret [1], Marie-Elodie Cattin-Messaoudi[2] PhD, Sofia Lotfi[2], Thierry Dorval[2] PhD, Pascal Berson[3] PhD, and Jean-Sébastien Hulot[1,4*] MD PhD.

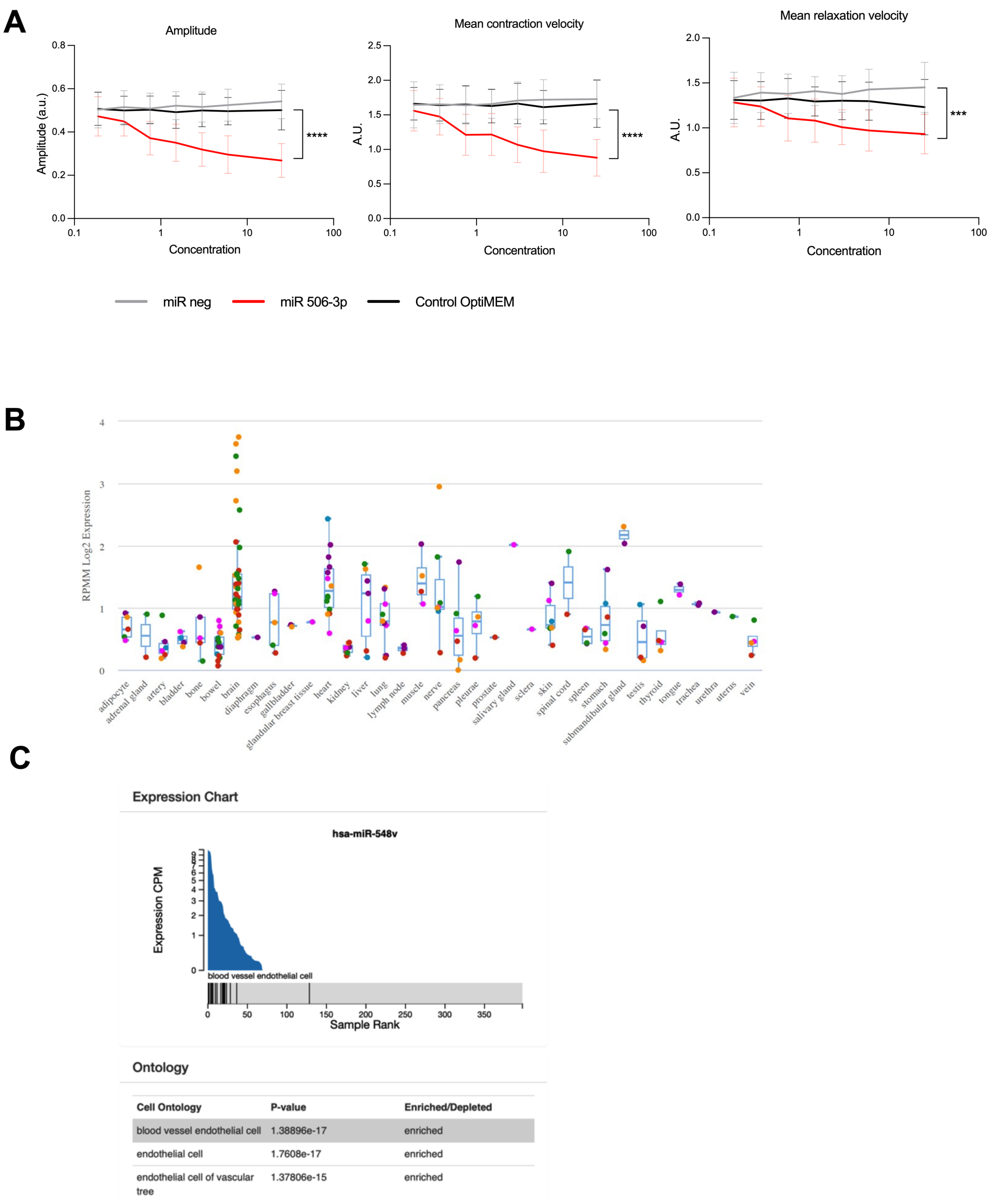

Figure Supplemental 1: **HTS quality control and expression profile of hsa-miR-548V**
A HTS Quality control: impact of different concentrations of miR negative control (grey) compared to the addition of transfection media only (black), or miR-506-3p (red) on amplitude, mean contraction velocity and mean relaxation velocity. N=3 screens. ***p<0.001, ****<p<0.0001 for miR-506-3p as compared to controls, ANOVA with post-hoc comparisons. **B** MiRNATissueAtlas2 results for hsa-miR-548v. **C** Fantom5 results for hsa-miR-548v showing an exclusive expression in endothelial cells

A

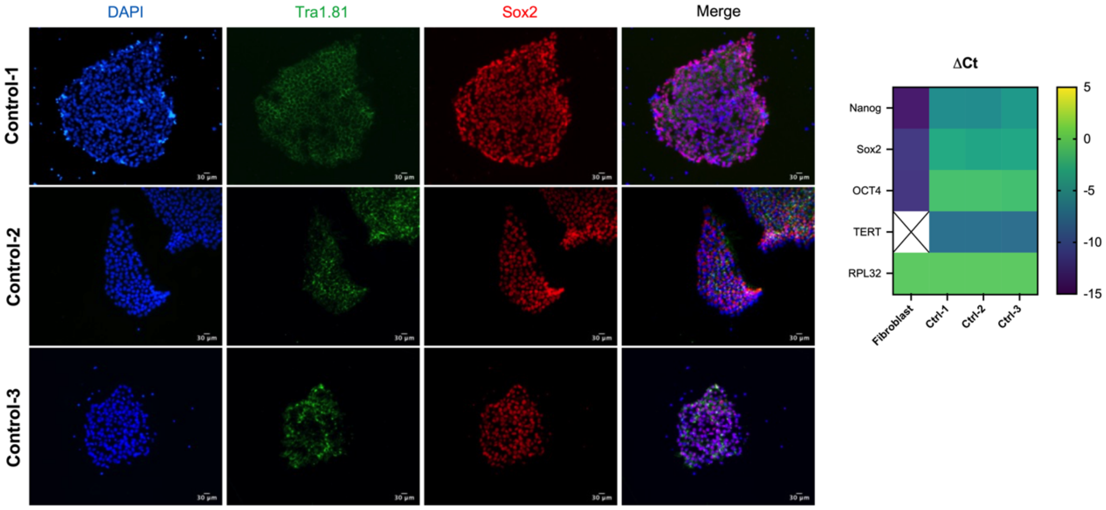

B

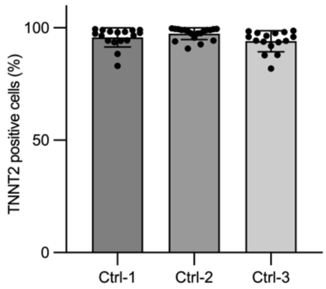

C

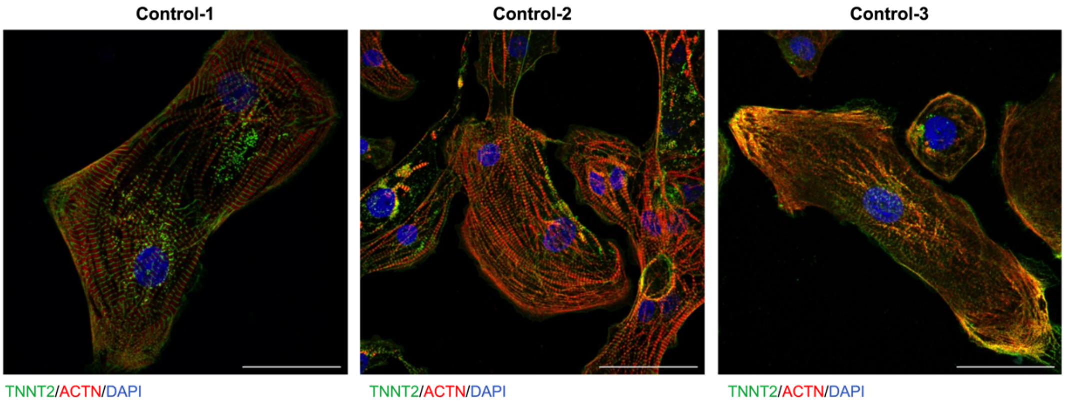

Figure Supplemental 2: **Quality control of hiPS control cell lines:**

A Control-1, Control-2 and Control-3 pluripotency evaluation by immunofluorescence (left) and qPCR (right). **B** Quality control of TroponinT2 positive cells by flow cytometry for Control-1 (n=17), Control-2 (n=19) and Control-3 (n=16). **C** Representative images of control cardiomyocytes stained for TroponinT2 (Red), Actinin (Green) and DAPI (Blue). Scale bar = 30 µm.

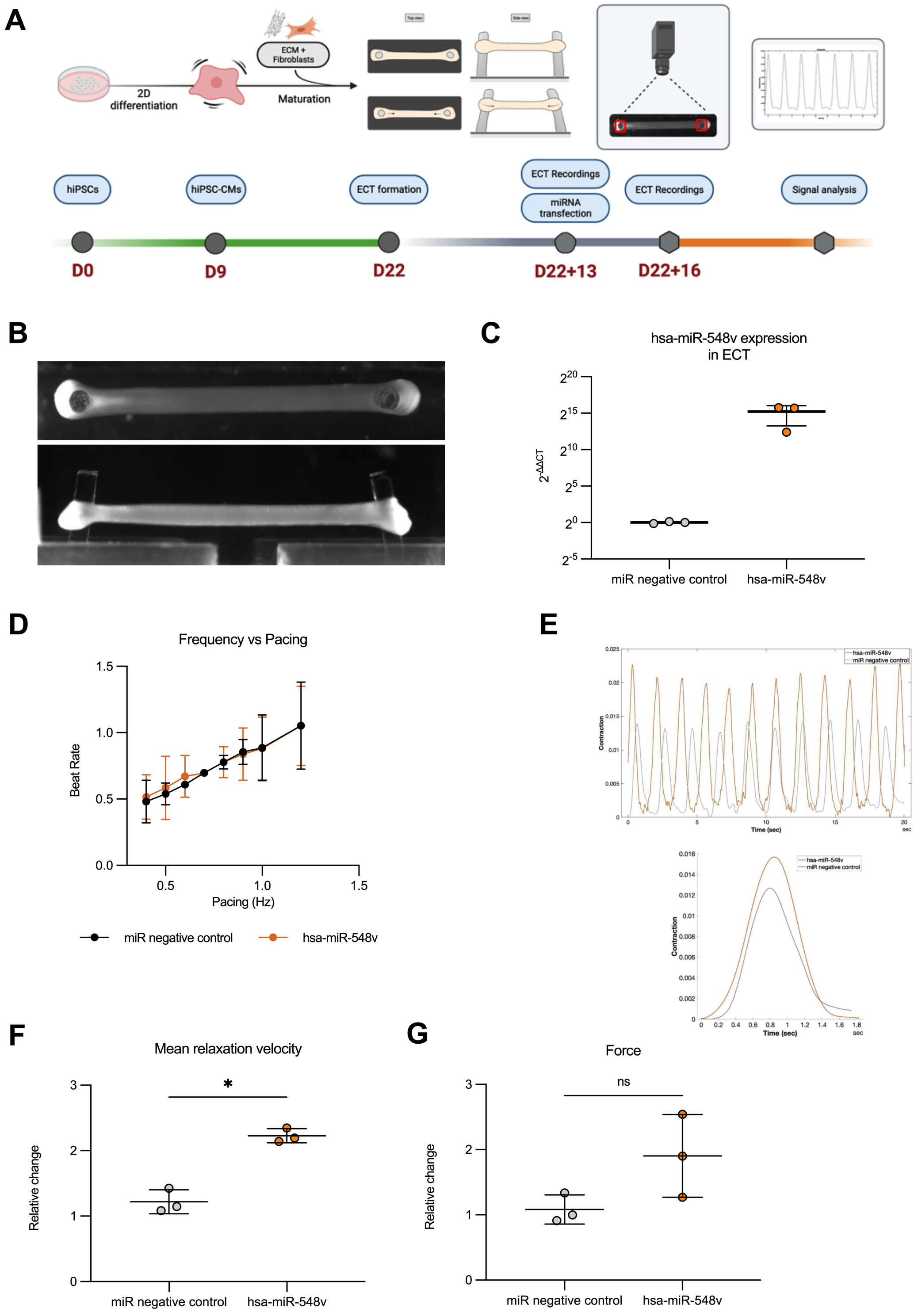

Figure Supplemental 3: hsa-miR-548v effect in hiPSC-CM-based engineered cardiac tissues (hECT)
A Experimental workflow. The green arrow represents the experiments until hECT formation. The grey arrow corresponds to baseline and 3 days post transfection hECTs' video recordings. The orange arrow corresponds to the post-processing of videos to acquire and analyze motion parameters. **B** Representative top and side view of hECT. **C** Evaluation of hsa-miR-548v expression levels 3 days after transfection (n=3 per groups). **D** Average beat rate according to pacing. (n=5 per group). **E** Representative recordings of beat-to-beat motion from hECTs transfected with hsa-miR-548v (orange) or miR negative control (grey). Averaged contraction/relaxation cycle recorded from ECTs transfected with hsa-miR-548v (orange) or miR negative control (grey). **F** Relative mean relaxation velocities in hECT 3 days after transfection with hsa-miR-548v (n=3) or miR negative control (n=3). (*, $p < 0.05$). **G** Relative developed force in hECT 3 days post transfection with hsa-miR-548v (n=3) or miR negative control (n=3).

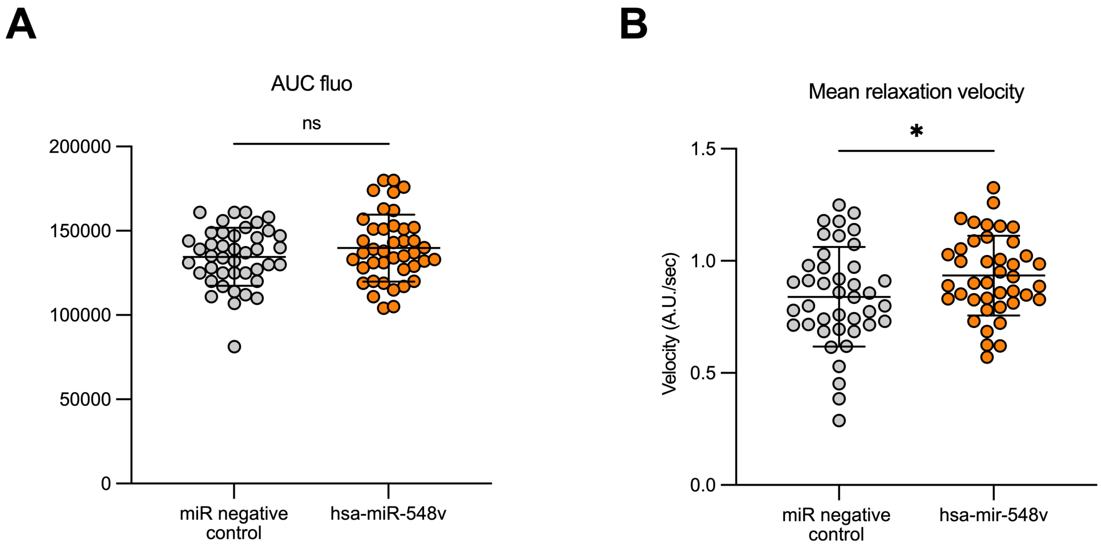

Figure Supplemental 4: Calcium imaging in hiPSC-CMs monolayers

A Area under the curve – calcium transient analysis in fluo4 loaded hiPSC-CMs transfected with miR negative control (n=42) or hsa-miR-548v (n=41).

B Mean relaxation velocity extracted from bright field records of hiPSC-CMs transfected with miR negative control (n=41) or hsa-miR-548v (n=41). *, P<0.05, T test.

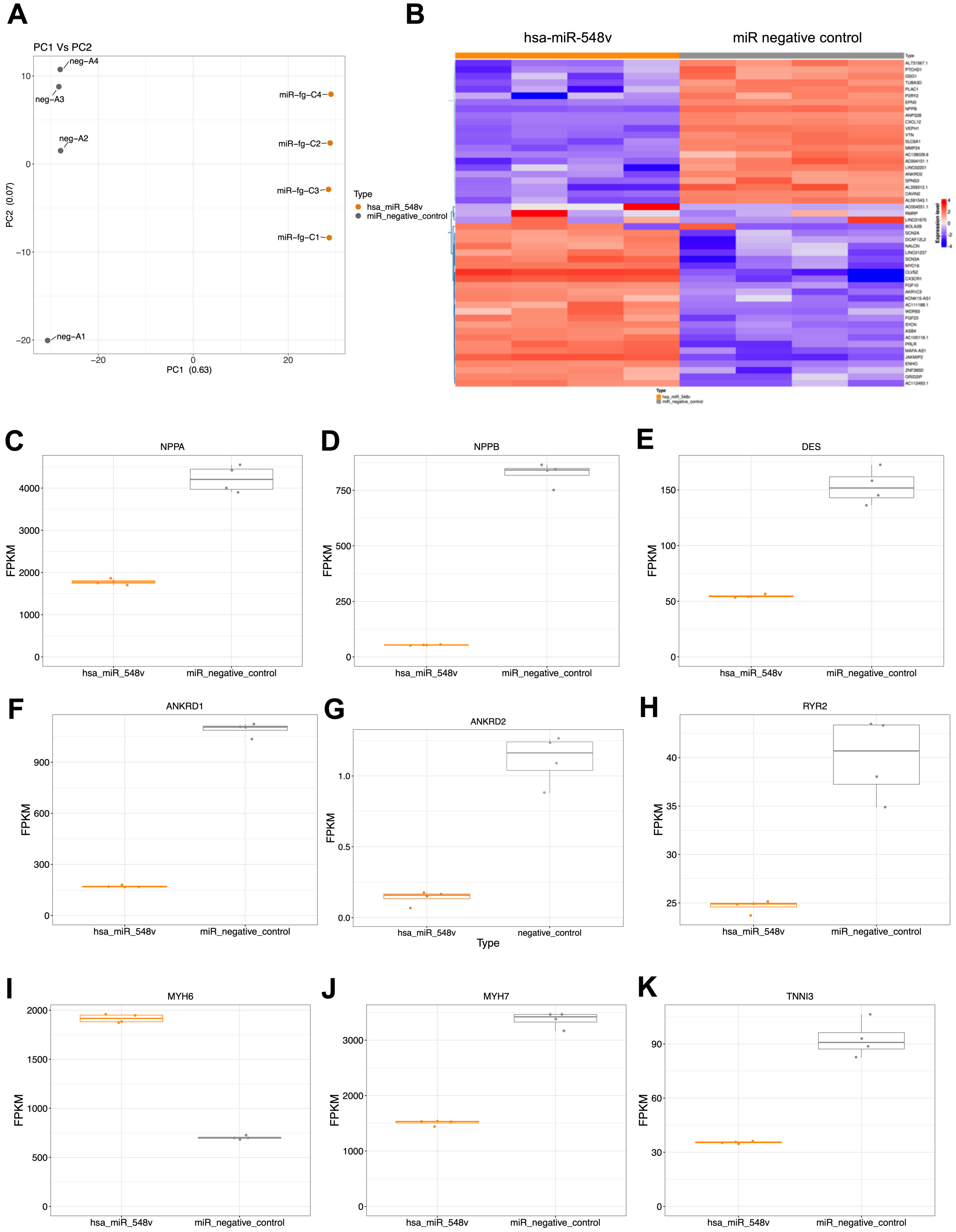

Figure Supplemental 5: Analysis of RNA-sequencing between control and hsa-miR-548v transfected hiPSC-CMs. **A** Principal component analysis based on the 1000 most variant genes. **B** Top 50 differentially expressed genes in hsa-miR-548v vs miR negative control. **C-K** level of expression of **C** *NNPA,* **D** *NNPB,* **E** *DES,* **F** *ANKDR1,* **G** *ANKDR2,* **H** *RYR2,* **I** *MYH6,* **J** *MYH7,* **K** *TNNI3*

A

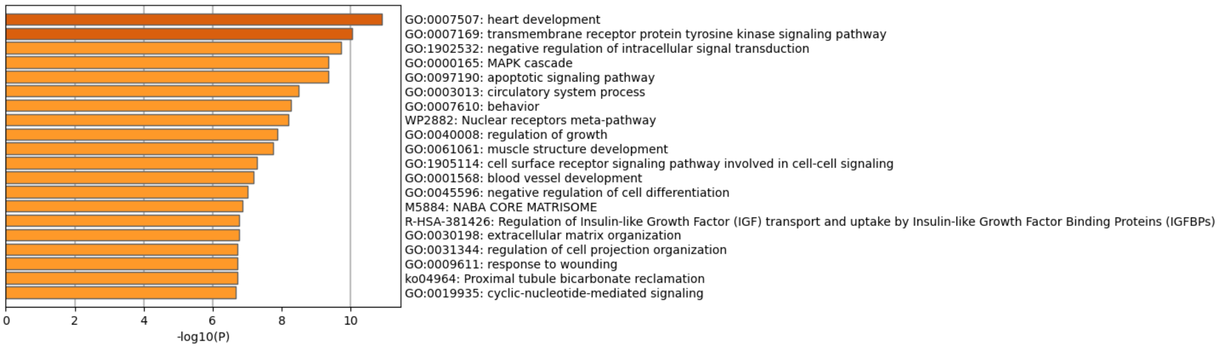

B

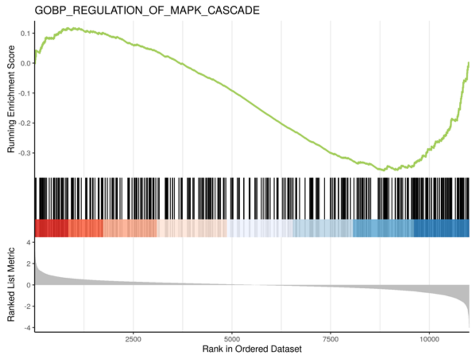

Figure Supplemental 6: Pathway analysis of RNA-sequencing between control and hsa-miR-548v transfected hiPSC-CMs.

A GSEA enriched pathways.

B Down-regulation of the MAPK cascade in response to hsa-miR-548v transfer.

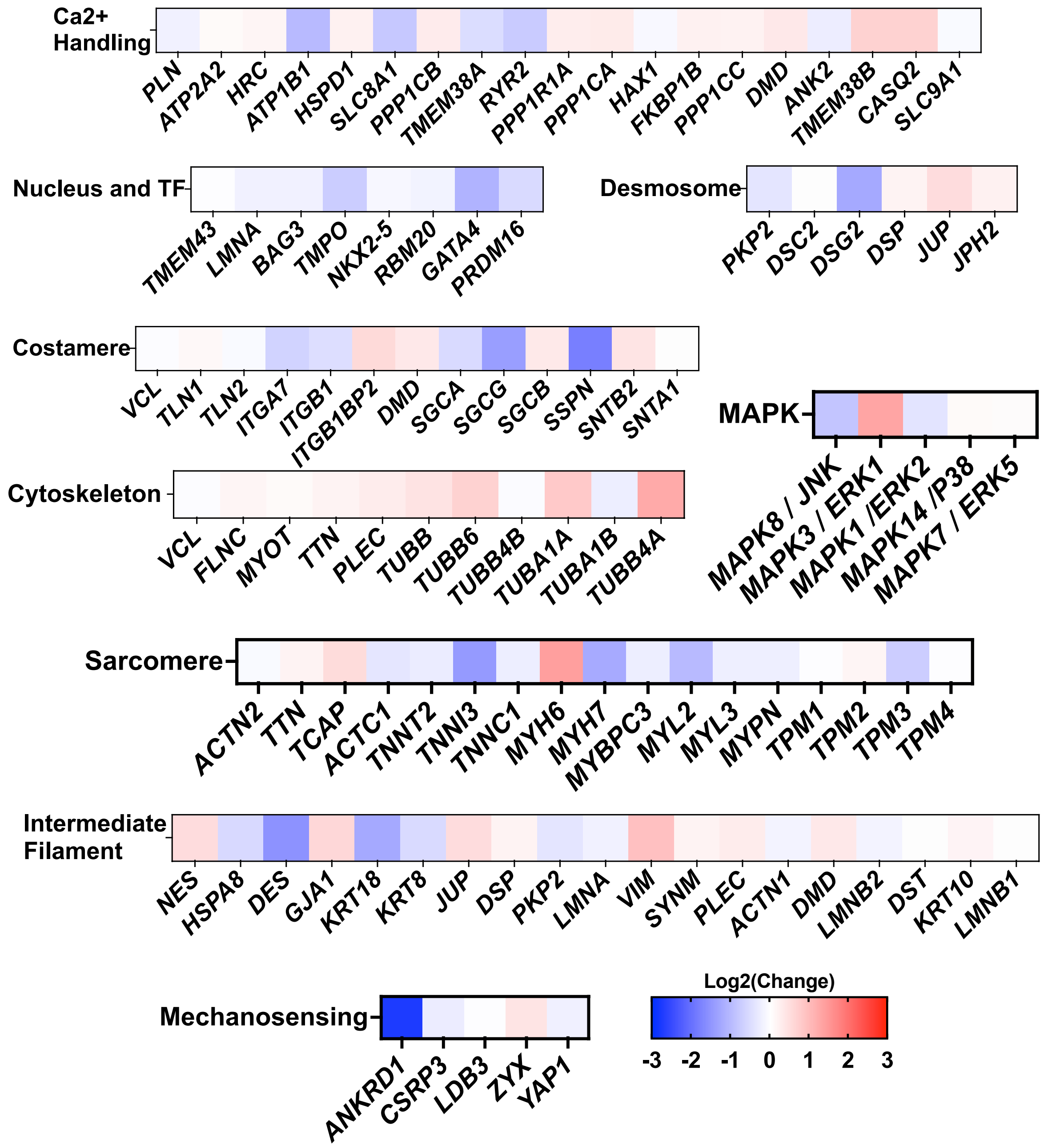

Figure Supplemental 7: Differential analysis of key genes of cardiomyocytes architecture

Supplemental Video V1: Bright field (BF) recording during HTS
Supplemental Video V2: Vector flow analysis of BF analysis during HTS
Supplemental Video V3: Beating ECT generated as linear macrostructure imaged by high-speed microscopy
Supplemental Video V4-V6: Beating ring-shaped ECT generated with the three control hiPS cell lines.
Supplemental Video V7: Beating ring-shaped ECT generated with the BRAF T599R mutated hiPSC-CMs

Supplemental Table 1: Overexpressed genes in hsa-miR-548v transfected cells compared to miR negative control transfected cells
Supplemental Table 2: Underexpressed genes in hsa-miR-548v transfected cells compared to miR negative control transfected cells
Supplemental Table 3: List of oligonucleotides used for CRISPR-Cas9 experiments

Figure 5E full unedited gels

ANKRD1/CARP1

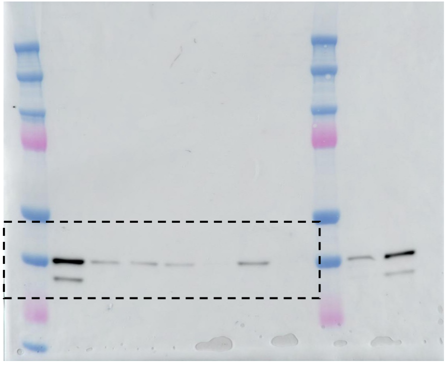

Vinculin

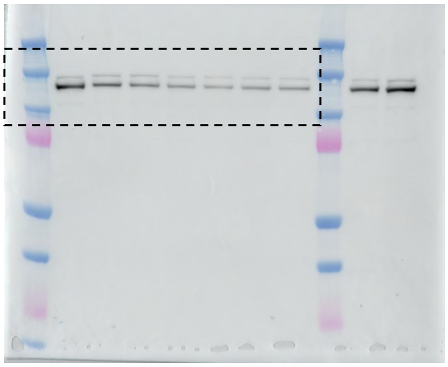

Figure 6C full unedited gels

6C

ANKRD1/CARP1

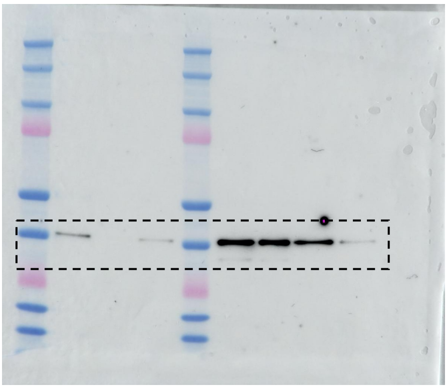

Vinculin

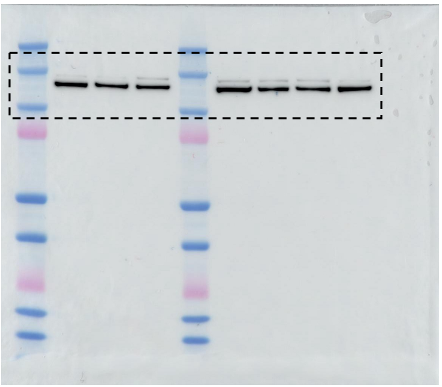

6D

pERK

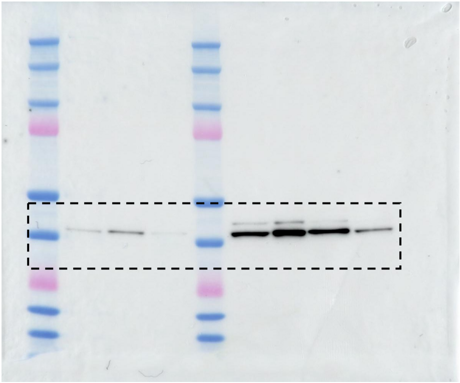

ERK 1/2

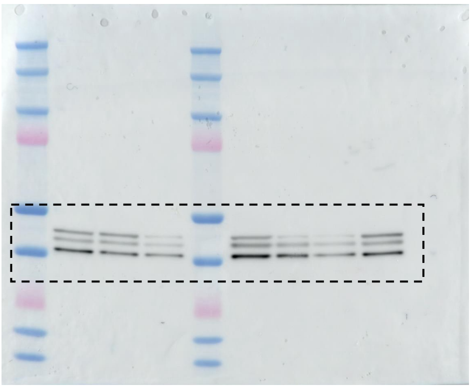

Figure 6D full unedited gels

ANKRD1/CARP1

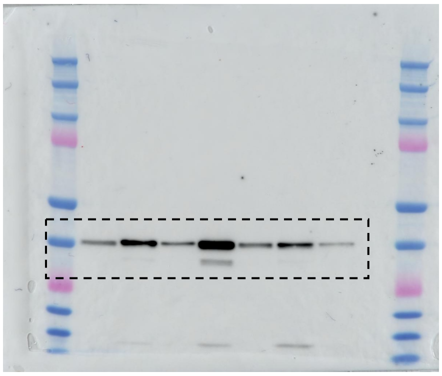

Vinculin

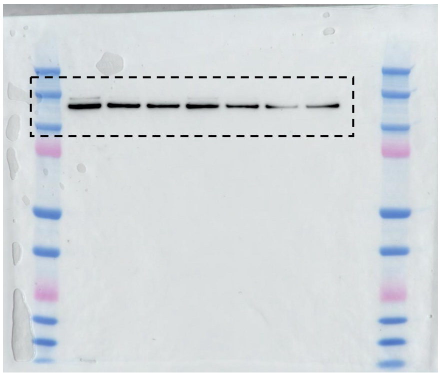

Supplement: Supplemental data [file jciinsight-9-161356-s008.pdf]
