# Supplementary material for: hsa-miR-548v controls the viscoelastic properties of human cardiomyocytes and improves their relaxation rates
Source: JCI Insight. 2024 Jan 2;9(3):e161356. doi: 10.1172/jci.insight.161356 (PMC11143964; doi:10.1172/jci.insight.161356)
Supplement: Supplemental tables 1-3 [file jciinsight-9-161356-s009.pdf]

Supplemental Table 1: Overexpressed genes in hsa-miR-548v transfected cells compared to miR negative transfected cells

Gene_Name	Description	Gene_biotype	Gene_position	CumulativeLengthExon	FPKM.miR negative control	FPKM.Hsa-mir-548v	log2FC	pval	qval
PDIA4	protein disulfide isomerase family A meml	protein_coding	chr7:149003062-1	3298	8.663359486	36.52205951	1.988677	2E-18	2E-15
TMEM65	transmembrane protein 65	protein_coding	chr8:124306189-1	9029	6.39270292	23.06597591	1.765124	4E-18	3E-15
CCND3	cyclin D3	protein_coding	chr6:41934933-42	5802	3.551127796	12.56177756	1.737059	5E-17	2E-14
SNCA	synuclein alpha	protein_coding	chr4:89724099-89	4014	2.515301081	13.75441229	2.369086	5E-17	2E-14
BRINP3	BMP/retinoic acid inducible neural specif	protein_coding	chr1:190097658-1	4273	3.772869407	13.60204712	1.762238	7E-17	2E-14
KALRN	kalirin RhoGEF kinase	protein_coding	chr3:124080023-1	24490	2.525051576	7.062496112	1.397722	1E-16	3E-14
CALU	calumenin	protein_coding	chr7:128739292-1	6342	22.19578388	61.90069026	1.393866	2E-16	3E-14
EPB41L1	erythrocyte membrane protein band 4.1 l	protein_coding	chr20:36091504-3	9676	2.416367742	8.260619741	1.68858	2E-16	3E-14
HSPA5	heat shock protein family A (Hsp70) meml	protein_coding	chr9:125234853-1	3921	88.15947024	229.2646018	1.292835	2E-16	4E-14
MLF1	myeloid leukemia factor 1	protein_coding	chr3:158571163-1	3735	31.69237796	96.08433845	1.514275	3E-16	4E-14
SEPT10	septin 10	protein_coding	chr2:109542799-1	4251	11.35450987	29.46105501	1.288688	3E-16	5E-14
PRSS35	serine protease 35	protein_coding	chr6:83512534-83	2444	71.65432186	202.9033864	1.415555	4E-16	6E-14
LDOC1	LDOC1 regulator of NFKB signaling	protein_coding	chrX:141111605-1	4979	2.094843233	8.307526588	1.897474	5E-16	7E-14
MYLK	myosin light chain kinase	protein_coding	chr3:123610049-1	22167	0.310800961	1.58200751	2.256298	5E-16	7E-14
KATNAL1	katanin catalytic subunit A1 like 1	protein_coding	chr13:30202630-3	8172	4.430191698	12.4735505	1.408751	6E-16	7E-14
KLHDC8B	kelch domain containing 8B	protein_coding	chr3:49171598-49	2171	69.55512134	181.4486072	1.296156	8E-16	9E-14
RTN4RL1	reticulon 4 receptor like 1	protein_coding	chr17:1934677-20	3614	5.702771202	16.40053799	1.438579	8E-16	9E-14
KCNV1	potassium voltage-gated channel modifie	protein_coding	chr8:109963636-1	6912	1.119796483	6.289795838	2.405966	8E-16	9E-14
STAG2	stromal antigen 2	protein_coding	chrX:123960212-1	8187	5.908070909	13.85876578	1.143642	9E-16	1E-13
CYP2U1	cytochrome P450 family 2 subfamily U m	protein_coding	chr4:107931549-1	5920	1.554698085	5.486477279	1.734868	1E-15	1E-13
SEC24D	SEC24 homolog D, COPII coat complex co	protein_coding	chr4:118722823-1	9920	1.924269166	5.412901936	1.405995	1E-15	1E-13
SORCS2	sortilin related VPS10 domain containing	protein_coding	chr4:7192538-774	6317	3.638695369	11.54901278	1.583625	1E-15	1E-13
TMEM263	transmembrane protein 263	protein_coding	chr12:106955719-	4768	19.05069334	44.81714026	1.147866	2E-15	1E-13
CDC34	cell division cycle 34	protein_coding	chr19:531760-542	1894	50.32152411	130.6899242	1.290045	2E-15	2E-13
OAT	ornithine aminotransferase	protein_coding	chr10:124397303-	3388	4.152510156	12.78181313	1.536845	2E-15	2E-13
JPH1	junctophilin 1	protein_coding	chr8:74234700-74	4590	8.608447921	20.2987129	1.151751	3E-15	2E-13
HACE1	HECT domain and ankyrin repeat containi	protein_coding	chr6:104728094-1	9237	1.927156047	5.095105219	1.31893	3E-15	2E-13
PURPL	p53 upregulated regulator of p53 levels	lncRNA	chr5:27217714-27	5603	9.891451116	22.06442186	1.070989	3E-15	3E-13
TNFRSF10D	TNF receptor superfamily member 10d	protein_coding	chr8:23135588-23	3535	3.037625306	11.58514131	1.843246	3E-15	3E-13
SEL1L	SEL1L adaptor subunit of ERAD E3 ubiquit	protein_coding	chr14:81471547-8	9553	4.996776997	11.84862586	1.158817	4E-15	3E-13
MYOF	myoferlin	protein_coding	chr10:93306429-9	8490	0.570328106	3.563760365	2.556016	4E-15	3E-13
MYH6	myosin heavy chain 6	protein_coding	chr14:23381982-2	7281	701.4864046	1916.247544	1.362946	5E-15	3E-13
PYGB	glycogen phosphorylase B	protein_coding	chr20:25248085-2	4456	157.0628202	350.4805812	1.072067	5E-15	3E-13
PRKAR2B	protein kinase cAMP-dependent type II re	protein_coding	chr7:107044705-1	4262	24.491097	56.12389875	1.109324	5E-15	4E-13
OSBPL8	oxysterol binding protein like 8	protein_coding	chr12:76351797-7	9198	9.99157983	21.41450675	1.013342	6E-15	4E-13
LONRF1	LON peptidase N-terminal domain and rin	protein_coding	chr8:12721906-12	5709	3.394200652	9.612769576	1.417459	7E-15	4E-13
B4GALT6	beta-1,4-galactosyltransferase 6	protein_coding	chr18:31622247-3	5306	2.752704574	7.42520652	1.343879	7E-15	4E-13
ERO1B	endoplasmic reticulum oxidoreductase 1	protein_coding	chr1:236215555-2	5361	1.093997335	4.284656536	1.880887	7E-15	4E-13
DSEL	dermatan sulfate epimerase like	protein_coding	chr18:67506587-6	9266	1.590317528	5.321193955	1.654989	8E-15	5E-13
RASD2	RASD family member 2	protein_coding	chr22:35540831-3	3447	2.541258865	9.656986787	1.840674	8E-15	5E-13
ART3	ADP-ribosyltransferase 3	protein_coding	chr4:76011184-76	3278	2.095235023	8.188542204	1.876421	8E-15	5E-13
PGRMC1	progesterone receptor membrane compo	protein_coding	chrX:119236245-1	1919	22.90348005	49.82033642	1.034665	8E-15	5E-13
PJA2	praja ring finger ubiquitin ligase 2	protein_coding	chr5:109334713-1	5193	27.77111176	54.28572885	0.880713	1E-14	5E-13
PI4K2B	phosphatidylinositol 4-kinase type 2 beta	protein_coding	chr4:25160663-25	3685	2.166194217	6.992573837	1.60408	1E-14	6E-13
NRAS	NRAS proto-oncogene, GTPase	protein_coding	chr1:114704469-1	4326	8.549702438	19.78742796	1.125413	1E-14	6E-13
KLHL13	kelch like family member 13	protein_coding	chrX:117897813-1	6983	13.79928074	28.22590695	0.945756	1E-14	6E-13
TWF1	twinfilin actin binding protein 1	protein_coding	chr12:43793723-4	3932	6.060723542	16.26455097	1.33834	1E-14	6E-13
AC087289.1	tripartite motif containing 47	protein_coding	chr17:75874164-7	3145	3.892104193	10.40070361	1.333858	1E-14	7E-13
CLU	clusterin	protein_coding	chr8:27596917-27	6616	5.26278398	15.06777424	1.431388	1E-14	7E-13
DYNLT3	dynein light chain Tctex-type 3	protein_coding	chrX:37836757-37	3120	3.61117165	13.32015849	1.798808	2E-14	8E-13
ALDH18A1	aldehyde dehydrogenase 18 family memb	protein_coding	chr10:95605941-9	3759	6.863258817	15.10679236	1.05188	2E-14	8E-13
ZDHHC14	zinc finger DHHC-type containing 14	protein_coding	chr6:157381133-1	11132	1.594169256	3.924853414	1.216746	2E-14	8E-13
ME1	malic enzyme 1	protein_coding	chr6:83210402-83	3338	9.230599268	20.07863785	1.03337	2E-14	8E-13
WDR1	WD repeat domain 1	protein_coding	chr4:10074339-10	7827	22.44324904	43.86967197	0.880436	2E-14	8E-13
DPYSL3	dihydropyrimidinase like 3	protein_coding	chr5:147390808-1	7179	20.9166866	42.25240854	0.928447	2E-14	8E-13
BANF1	barrier to autointegration factor 1	protein_coding	chr11:66002228-6	1496	32.85717338	90.62956144	1.380578	2E-14	8E-13
PYGO2	pygopus family PHD finger 2	protein_coding	chr1:154957026-1	3664	7.699041543	17.43717363	1.092228	2E-14	9E-13
GRAMD2B	GRAM domain containing 2B	protein_coding	chr5:126360132-1	5051	5.194590015	11.27556035	1.032631	2E-14	9E-13
SELENOM	selenoprotein M	protein_coding	chr22:31104772-3	3187	9.998223602	32.56423342	1.620174	2E-14	1E-12
CLIP2	CAP-Gly domain containing linker protein	protein_coding	chr7:74289475-74	7315	0.290529845	1.882844825	2.607525	2E-14	1E-12
CAV1	caveolin 1	protein_coding	chr7:116525001-1	3866	33.16068093	74.04893827	1.073353	3E-14	1E-12
DAB1	DAB adaptor protein 1	protein_coding	chr1:56994778-58	7548	2.825055352	6.77200029	1.174997	3E-14	1E-12
SOCS6	suppressor of cytokine signaling 6	protein_coding	chr18:70289045-7	5839	13.5309434	28.31273335	0.979435	3E-14	1E-12
CRMP1	collapsin response mediator protein 1	protein_coding	chr4:5748084-589	5823	7.11769365	13.98106376	0.887287	3E-14	1E-12
TRPM7	transient receptor potential cation chann	protein_coding	chr15:50552473-5	12380	6.835209987	14.44838818	0.99427	3E-14	1E-12
MMD	monocyte to macrophage differentiation	protein_coding	chr17:55392622-5	3193	6.895954587	17.46550289	1.252857	3E-14	1E-12
RFK	riboflavin kinase	protein_coding	chr9:76385526-76	3868	4.681670923	10.48503325	1.075937	3E-14	1E-12
MFAP2	microfibril associated protein 2	protein_coding	chr1:16974502-16	2915	3.809518981	12.16289919	1.585797	3E-14	1E-12
GRK5	G protein-coupled receptor kinase 5	protein_coding	chr10:119207589-	7000	2.133635183	5.577331643	1.302367	3E-14	1E-12
GPR180	G protein-coupled receptor 180	protein_coding	chr13:94601857-9	8884	0.88719571	3.056356908	1.694149	4E-14	1E-12
SPAST	spastin	protein_coding	chr2:32063547-32	9490	2.293271463	5.082688761	1.061348	4E-14	2E-12
MET	MET proto-oncogene, receptor tyrosine ki	protein_coding	chr7:116672196-1	7233	0.312129247	2.23629832	2.746724	4E-14	2E-12
BOD1	biorientation of chromosomes in cell divi	protein_coding	chr5:173607514-1	2018	10.21622653	21.92351637	1.015007	4E-14	2E-12
VIM	vimentin	protein_coding	chr10:17228241-1	5176	47.74524494	94.71228681	0.901984	4E-14	2E-12
ZMAT4	zinc finger matrin-type 4	protein_coding	chr8:40530590-40	3691	3.498930501	8.351419809	1.169201	5E-14	2E-12
AKAP12	A-kinase anchoring protein 12	protein_coding	chr6:151239967-1	8775	1.676228279	4.518903562	1.344462	5E-14	2E-12
DNAJB1	DnaJ heat shock protein family (Hsp40) m	protein_coding	chr19:14514769-1	3650	21.21442181	39.40144384	0.806573	5E-14	2E-12
STX12	syntaxin 12	protein_coding	chr1:27773219-27	3801	5.803992598	15.45027695	1.327027	5E-14	2E-12
GRB14	growth factor receptor bound protein 14	protein_coding	chr2:164492812-1	2958	0.700678327	4.199082681	2.49068	5E-14	2E-12
PTGER3	prostaglandin E receptor 3	protein_coding	chr1:70852353-71	9653	0.910354661	2.804899537	1.538477	5E-14	2E-12
MEX3D	mex-3 RNA binding family member D	protein_coding	chr19:1554669-15	2850	4.040869782	10.81090195	1.333013	5E-14	2E-12
PLPP1	phospholipid phosphatase 1	protein_coding	chr5:55424854-55	1935	12.19522733	27.2124442	1.07046	6E-14	2E-12
MBTPS1	membrane bound transcription factor pe	protein_coding	chr16:84053761-8	10274	7.949535409	15.69494409	0.894902	6E-14	2E-12
CLIP3	CAP-Gly domain containing linker protein	protein_coding	chr19:36014660-3	3765	3.122269288	10.0688643	1.607379	6E-14	2E-12
LMOD1	leiomodin 1	protein_coding	chr1:201896456-2	3967	15.2960165	33.08734718	1.025289	6E-14	2E-12
FGF18	fibroblast growth factor 18	protein_coding	chr5:171419647-1	1998	8.964048325	22.25192346	1.225497	7E-14	2E-12
OXCT1	3-oxoacid CoA-transferase 1	protein_coding	chr5:41730065-41	4100	29.9452769	54.65353179	0.7816	8E-14	3E-12
KRAS	KRAS proto-oncogene, GTPase	protein_coding	chr12:25204789-2	7302	5.195609901	10.48599007	0.926282	8E-14	3E-12
GNPTAB	N-acetylglucosamine-1-phosphate transfe	protein_coding	chr12:101745499-	9774	1.660246355	3.963451105	1.17109	9E-14	3E-12
BMT2	base methyltransferase of 25S rRNA 2 hor	protein_coding	chr7:112819147-1	4097	41.24414857	77.65306536	0.826957	1E-13	3E-12
EPOR	erythropoietin receptor	protein_coding	chr19:11377207-1	3077	14.56629722	29.20567686	0.91902	1E-13	3E-12
SMAP1	small ArfGAP 1	protein_coding	chr6:70667776-70	4161	11.50151847	24.11374242	0.982826	1E-13	3E-12
SMARCD2	SWI/SNF related, matrix associated, actin	protein_coding	chr17:63832081-6	3734	4.15799473	11.28993114	1.351175	1E-13	3E-12
LRRC4B	leucine rich repeat containing 4B	protein_coding	chr19:50516892-5	3511	7.212238764	19.33455803	1.336644	1E-13	4E-12
TMEM254	transmembrane protein 254	protein_coding	chr10:80078646-8	2996	2.057205415	5.611335295	1.360772	1E-13	4E-12
MANEA	mannosidase endo-alpha	protein_coding	chr6:95577535-95	5190	3.350256592	8.592053184	1.270724	1E-13	4E-12
PRKCI	protein kinase C iota	protein_coding	chr3:170222424-1	5778	6.949704519	13.06345477	0.825255	1E-13	4E-12
ECHDC2	enoyl-CoA hydratase domain containing 2	protein_coding	chr1:52895910-52	11572	3.24559567	6.875754469	0.997335	1E-13	4E-12
MAPK3	mitogen-activated protein kinase 3	protein_coding	chr16:30114105-3	3669	4.400551327	11.48784531	1.300966	1E-13	4E-12
TCEA3	transcription elongation factor A3	protein_coding	chr1:23381061-23	3515	16.50939481	31.48419412	0.845467	1E-13	4E-12
KLHL9	kelch like family member 9	protein_coding	chr9:21329671-21	5710	5.534548645	11.06277416	0.912712	2E-13	4E-12
ATP9A	ATPase phospholipid transporting 9A (put	protein_coding	chr20:51596514-5	8317	3.808958241	9.945489939	1.301615	2E-13	5E-12
HIST1H2AC	histone cluster 1 H2A family member c	protein_coding	chr6:26124145-26	1668	11.69303878	24.70098546	0.992008	2E-13	5E-12
PLEKHO1	pleckstrin homology domain containing (	protein_coding	chr1:150149183-1	6629	8.414463538	17.56315854	0.973602	2E-13	5E-12
UNC119B	unc-119 lipid binding chaperone B	protein_coding	chr12:120710458-	5172	4.463382864	9.781847793	1.043124	2E-13	5E-12
PPIP5K2	diphosphoinositol pentakisphosphate kin	protein_coding	chr5:103120149-1	17270	2.857516926	5.597202023	0.884843	2E-13	5E-12
PLIN2	perilipin 2	protein_coding	chr9:19108375-19	5011	7.128246313	14.87424352	0.976434	2E-13	5E-12

ZHX1	zinc fingers and homeoboxes 1	protein_coding	chr8:123248451-1	6733	4.205924243	8.400166132	0.910567	2E-13	5E-12
ARL5B	ADP ribosylation factor like GTPase 5B	protein_coding	chr10:18659431-1	7170	14.92367431	28.14297339	0.828833	2E-13	5E-12
SELENBP1	selenium binding protein 1	protein_coding	chr1:151364304-1	3476	1.808854893	5.080574289	1.399675	2E-13	5E-12
GJA3	gap junction protein alpha 3	protein_coding	chr13:20138255-2	5214	26.87877517	57.24146486	1.005822	2E-13	5E-12
CNN2	calponin 2	protein_coding	chr19:1026586-10	3640	30.20556443	64.41672476	1.005894	2E-13	6E-12
PABPC1L	poly(A) binding protein cytoplasmic 1 like	protein_coding	chr20:44910062-4	4984	2.366197753	5.886435163	1.231374	2E-13	6E-12
DNAJC3	DnaJ heat shock protein family (Hsp40) m	protein_coding	chr13:95677139-9	5590	4.485226677	9.331759105	0.969266	2E-13	6E-12
MTPN	myotrophin	protein_coding	chr7:135926760-1	3782	52.46494783	91.34755714	0.713561	2E-13	6E-12
KHNYN	KH and NYN domain containing	protein_coding	chr14:24429286-2	8024	6.42918355	15.32910765	1.169604	2E-13	6E-12
CAMSAP2	calmodulin regulated spectrin associated	protein_coding	chr1:200739558-2	7777	7.064751175	14.59094016	0.961838	2E-13	6E-12
CIB2	calcium and integrin binding family memb	protein_coding	chr15:78104606-7	2146	19.2003563	36.80332174	0.852749	2E-13	6E-12
INPP4A	inositol polyphosphate-4-phosphatase typ	protein_coding	chr2:98444854-98	13187	1.52470191	3.711134739	1.194526	2E-13	6E-12
ETV1	ETS variant 1	protein_coding	chr7:13891229-13	9615	7.929624904	17.20040246	1.031451	2E-13	6E-12
ACTG1	actin gamma 1	protein_coding	chr17:81509971-8	2920	92.24832879	172.3735049	0.816112	2E-13	6E-12
IFT81	intraflagellar transport 81	protein_coding	chr12:110124335-	7001	6.991231168	12.74434335	0.779545	2E-13	6E-12
SCAMP1	secretory carrier membrane protein 1	protein_coding	chr5:78360611-78	6746	10.17377618	18.03751973	0.739449	2E-13	6E-12
SSBP4	single stranded DNA binding protein 4	protein_coding	chr19:18418864-1	2974	18.37460238	35.57369639	0.865929	2E-13	6E-12
PNPLA6	patatin like phospholipase domain contai	protein_coding	chr19:7534004-75	7172	3.502374983	6.752353898	0.860739	2E-13	6E-12
GREB1L	GREB1 like retinoic acid receptor coactiva	protein_coding	chr18:21242242-2	10572	1.964439449	5.363581158	1.36388	2E-13	6E-12
ENTPD6	ectonucleoside triphosphate diphosphoh	protein_coding	chr20:25195693-2	5327	16.9475547	33.23780197	0.886307	3E-13	7E-12
SPINT1	serine peptidase inhibitor, Kunitz type 1	protein_coding	chr15:40844018-4	3542	0.700855334	4.61798433	2.638939	3E-13	7E-12
NSMF	NMDA receptor synaptonuclear signaling	protein_coding	chr9:137447573-1	4912	11.63847287	20.98647402	0.763833	3E-13	7E-12
PKN2	protein kinase N2	protein_coding	chr1:88684222-88	6897	17.34263132	30.48829791	0.727614	3E-13	7E-12
POSTN	periostin	protein_coding	chr13:37562583-3	5464	0.374347469	2.76907604	2.791077	3E-13	7E-12
SEMA3A	semaphorin 3A	protein_coding	chr7:83955777-84	9259	1.250081301	2.744760958	1.048981	3E-13	8E-12
RBM3	RNA binding motif protein 3	protein_coding	chrX:48574449-48	5827	13.96177086	25.56459253	0.786616	3E-13	8E-12
PIAS3	protein inhibitor of activated STAT 3	protein_coding	chr1:145848522-1	4806	5.796512561	11.04767432	0.843856	3E-13	8E-12
ISOC1	isochorismatase domain containing 1	protein_coding	chr5:129094749-1	1942	37.14270174	74.75022407	0.922991	4E-13	9E-12
LRRC8B	leucine rich repeat containing 8 VRAC sub	protein_coding	chr1:89524836-89	8213	1.377253178	3.49824482	1.263578	4E-13	9E-12
GNB5	G protein subunit beta 5	protein_coding	chr15:52115105-5	13302	2.233894473	4.04217221	0.768461	4E-13	9E-12
SKP2	S-phase kinase associated protein 2	protein_coding	chr5:36151989-36	3837	5.613785626	10.83699327	0.863648	4E-13	1E-11
ELL2	elongation factor for RNA polymerase II 2	protein_coding	chr5:95885098-95	7108	2.682852584	6.226460415	1.124391	4E-13	1E-11
RAB39B	RAB39B, member RAS oncogene family	protein_coding	chrX:155258235-1	3413	0.683377941	3.02545464	2.053833	4E-13	1E-11
TRERF1	transcriptional regulating factor 1	protein_coding	chr6:42224931-42	7732	1.048473116	3.123840761	1.487133	5E-13	1E-11
PAQR7	progestin and adipoQ receptor family mem	protein_coding	chr1:25861210-25	3297	1.967436886	5.292745225	1.345054	5E-13	1E-11
LRFN1	leucine rich repeat and fibronectin type II	protein_coding	chr19:39306568-3	3381	4.960522998	9.961148397	0.91861	5E-13	1E-11
KAZALD1	Kazal type serine peptidase inhibitor dom	protein_coding	chr10:101061841-	3613	6.53558349	14.61917416	1.075057	5E-13	1E-11
SGPP1	sphingosine-1-phosphate phosphatase 1	protein_coding	chr14:63684216-6	3336	2.60151229	6.057524003	1.131216	5E-13	1E-11
TMX1	thioredoxin related transmembrane prote	protein_coding	chr14:51240162-5	4608	3.107826724	7.296541947	1.142916	5E-13	1E-11
SLC24A3	solute carrier family 24 member 3	protein_coding	chr20:19212646-1	3933	0.668621745	2.943709998	2.061609	5E-13	1E-11
SPHKAP	SPHK1 interactor, AKAP domain containin	protein_coding	chr2:227979955-2	7046	60.62307028	111.3822182	0.791612	5E-13	1E-11
SCAF1	SR-related CTD associated factor 1	protein_coding	chr19:49642209-4	4464	20.45485908	35.21390033	0.697268	5E-13	1E-11
STK38	serine/threonine kinase 38	protein_coding	chr6:36493892-36	3585	13.20234294	23.93579067	0.771724	6E-13	1E-11
GCA	grancalcin	protein_coding	chr2:162318840-1	5323	3.536923514	6.914143379	0.881464	6E-13	1E-11
LIPA	lipase A, lysosomal acid type	protein_coding	chr10:89213569-8	4891	0.776822624	2.780649317	1.751873	6E-13	1E-11
PLPP5	phospholipid phosphatase 5	protein_coding	chr8:38263130-38	4102	2.455855926	6.393132031	1.291543	6E-13	1E-11
CLYBL	citrate lyase beta like	protein_coding	chr13:99606669-9	7227	1.927296928	5.383803905	1.404436	6E-13	1E-11
IL1RAP	interleukin 1 receptor accessory protein	protein_coding	chr3:190514051-1	11558	1.918642576	5.000162067	1.295253	6E-13	1E-11
TADA3	transcriptional adaptor 3	protein_coding	chr3:9779860-979	4234	27.79502589	50.80687571	0.7848	6E-13	1E-11
NLGN2	neuroligin 2	protein_coding	chr17:7404874-74	5717	3.278416099	8.612031183	1.309605	7E-13	1E-11
CITED4	Cbp/p300 interacting transactivator with	protein_coding	chr1:40861051-40	1316	89.8907162	216.3395884	1.179157	7E-13	1E-11
PIP4K2A	phosphatidylinositol-5-phosphate 4-kina	protein_coding	chr10:22534854-2	5106	2.281510133	6.425521482	1.418431	7E-13	1E-11
CLEC2D	C-type lectin domain family 2 member D	protein_coding	chr12:9664969-96	6955	0.966882446	3.544937639	1.784115	7E-13	1E-11
PRSS16	serine protease 16	protein_coding	chr6:27247701-27	5290	0.261232517	1.449051618	2.384191	8E-13	2E-11
CBR1	carbonyl reductase 1	protein_coding	chr21:36069941-3	2450	4.461562469	10.21179548	1.108838	8E-13	2E-11
BRMS1L	BRMS1 like transcriptional repressor	protein_coding	chr14:35826338-3	3877	6.549902672	13.41119805	0.947231	8E-13	2E-11
HAPLN3	hyaluronan and proteoglycan link protein	protein_coding	chr15:88877288-8	3524	3.295710359	9.550923944	1.446272	8E-13	2E-11
PLEKHB1	pleckstrin homology domain containing E	protein_coding	chr11:73646178-7	4933	1.104022394	3.418387717	1.547549	8E-13	2E-11
AC004854.2	novel transcript, antisense to PURB	lncRNA	chr7:44884953-44	1441	5.839448188	15.93908033	1.356806	9E-13	2E-11
CALR	calreticulin	protein_coding	chr19:12938578-1	2714	139.3222979	243.9719174	0.72243	1E-12	2E-11
RTL5	retrotransposon Gag like 5	protein_coding	chrX:72127110-72	4779	5.896640695	14.62063439	1.225623	1E-12	2E-11
TMEM14A	transmembrane protein 14A	protein_coding	chr6:52671113-52	988	2.079748528	9.297492421	2.064908	1E-12	2E-11
TRMT10A	tRNA methyltransferase 10A	protein_coding	chr4:99546709-99	4386	1.008001717	2.923844422	1.447225	1E-12	2E-11
LACTB	lactamase beta	protein_coding	chr15:63121833-6	3939	5.87466178	11.51652355	0.886584	1E-12	2E-11
ANP32E	acidic nuclear phosphoprotein 32 family	protein_coding	chr1:150218417-1	3605	23.98112453	42.23155732	0.730892	1E-12	2E-11
FAM114A1	family with sequence similarity 114 mem	protein_coding	chr4:38867677-38	4871	2.906096635	5.918309019	0.939179	1E-12	2E-11
SCRN1	secernin 1	protein_coding	chr7:29920104-29	6048	13.34227627	24.3067356	0.779409	1E-12	2E-11
TMEM159	transmembrane protein 159	protein_coding	chr16:21158377-2	2558	8.719892896	18.70151658	1.013048	1E-12	2E-11
NRBF2	nuclear receptor binding factor 2	protein_coding	chr10:63133247-6	1901	4.499865073	10.66786791	1.157034	1E-12	2E-11
TRUB1	TruB pseudouridine synthase family mem	protein_coding	chr10:114938195-	3599	6.02079131	11.64180477	0.865271	1E-12	2E-11
H3F3A	H3 histone family member 3A	protein_coding	chr1:226061851-2	3814	8.589410928	17.1720499	0.916813	1E-12	2E-11
NIN	ninein	protein_coding	chr14:50719763-5	13683	5.204120747	10.45266129	0.921773	1E-12	2E-11
ENHO	energy homeostasis associated	protein_coding	chr9:34521043-34	1028	1.229168773	11.27082922	3.108755	1E-12	2E-11
SLC12A6	solute carrier family 12 member 6	protein_coding	chr15:34229996-3	10056	4.108596252	8.32339322	0.933314	1E-12	2E-11
PPM1J	protein phosphatase, Mg2+/Mn2+ depend	protein_coding	chr1:112709994-1	2874	1.343802935	4.810452525	1.755296	1E-12	2E-11
LMOD3	leiomodin 3	protein_coding	chr3:69106872-69	4276	3.553924789	9.578149025	1.347224	1E-12	2E-11
SESN1	sestrin 1	protein_coding	chr6:108986437-1	5121	8.73587208	16.91660386	0.869857	1E-12	2E-11
GOPC	golgi associated PDZ and coiled-coil motif	protein_coding	chr6:117560269-1	4628	10.6355485	19.17858051	0.765038	1E-12	2E-11
GALNT11	polypeptide N-acetylgalactosaminyltrans	protein_coding	chr7:152025674-1	8288	2.786008448	5.369286558	0.860358	1E-12	2E-11
MYO5A	myosin VA	protein_coding	chr15:52307283-5	12988	2.947434782	6.895407448	1.141767	1E-12	2E-11
KCNJ12	potassium voltage-gated channel subfami	protein_coding	chr17:21376357-2	5455	1.860572146	4.621815153	1.235043	1E-12	2E-11
RRBP1	ribosome binding protein 1	protein_coding	chr20:17613678-1	6980	3.16299364	6.503246734	0.95383	1E-12	2E-11
FBXO44	F-box protein 44	protein_coding	chr1:11654375-11	3527	11.08758734	19.47256681	0.725764	1E-12	2E-11
HCN1	hyperpolarization activated cyclic nucleo	protein_coding	chr5:45254948-45	13706	0.313932341	1.115850772	1.741705	1E-12	3E-11
MCRS1	microspherule protein 1	protein_coding	chr12:49556544-4	3261	24.84113988	44.00263007	0.737994	1E-12	3E-11
KIF3A	kinesin family member 3A	protein_coding	chr5:132692628-1	6860	1.769298599	3.746638327	0.995796	1E-12	3E-11
CADPS2	calcium dependent secretion activator 2	protein_coding	chr7:122318411-1	6365	3.655028129	7.368457752	0.925682	2E-12	3E-11
HID1	HID1 domain containing	protein_coding	chr17:74950742-7	6197	1.256619817	3.115102837	1.223422	2E-12	3E-11
ANKMY2	ankyrin repeat and MYND domain contain	protein_coding	chr7:16599779-16	2851	19.35929151	35.7118073	0.796976	2E-12	3E-11
KLHL42	kelch like family member 42	protein_coding	chr12:27780048-2	7594	7.690489544	14.43887328	0.82214	2E-12	3E-11
VCAN	versican	protein_coding	chr5:83471618-83	14678	26.0850369	50.02937796	0.853513	2E-12	3E-11
TOMM20	translocase of outer mitochondrial memb	protein_coding	chr1:235109341-2	3438	23.98513205	40.33051096	0.663941	2E-12	3E-11
TMEM8B	transmembrane protein 8B	protein_coding	chr9:35814451-35	16221	2.240256613	4.725033073	0.991193	2E-12	3E-11
MAP2K4	mitogen-activated protein kinase kinase 4	protein_coding	chr17:12020829-1	5709	6.916581378	12.99409753	0.823724	2E-12	3E-11
MBOAT2	membrane bound O-acyltransferase doma	protein_coding	chr2:8852690-900	8694	6.148343141	11.5188942	0.820476	2E-12	3E-11
NORAD	non-coding RNA activated by DNA damage	lncRNA	chr20:36045618-3	5401	50.2217323	88.9351094	0.738938	2E-12	3E-11
HSP90B1	heat shock protein 90 beta family membe	protein_coding	chr12:103930107-	4695	55.56175688	93.39483951	0.663233	2E-12	3E-11
KBTBD8	kelch repeat and BTB domain containing 8	protein_coding	chr3:66998307-67	4736	0.985498841	2.81058054	1.420267	2E-12	3E-11
GARNL3	GTPase activating Rap/RanGAP domain lik	protein_coding	chr9:127224265-1	9695	1.476284534	3.017610191	0.944645	2E-12	3E-11
VIRMA	vir like m6A methyltransferase associated	protein_coding	chr8:94487689-94	10775	5.370620767	8.984079037	0.656143	2E-12	3E-11
MT1E	metallothionein 1E	protein_coding	chr16:56625475-5	1052	5.156115215	14.7442327	1.42974	2E-12	3E-11
PGM5	phosphoglucomutase 5	protein_coding	chr9:68328308-68	7714	24.44502184	42.47382714	0.710736	2E-12	3E-11
PPP3CB	protein phosphatase 3 catalytic subunit b	protein_coding	chr10:73436428-7	5094	8.277508719	16.60689974	0.919557	2E-12	3E-11
SMYD2	SET and MYND domain containing 2	protein_coding	chr1:214281102-2	4889	29.05026036	47.40494758	0.619841	2E-12	3E-11
GAS2L1	growth arrest specific 2 like 1	protein_coding	chr22:29306582-2	4481	5.482612551	10.61887343	0.866892	2E-12	4E-11
HADH	hydroxyacyl-CoA dehydrogenase	protein_coding	chr4:107989714-1	12086	6.374266663	11.35024136	0.74789	2E-12	4E-11
ACAP2	ArfGAP with coiled-coil, ankyrin repeat ar	protein_coding	chr3:195274745-1	11096	4.122290821	7.079914749	0.693323	2E-12	4E-11
GSE1	Gse1 coiled-coil protein	protein_coding	chr16:85169525-8	13376	11.69262486	20.49495219	0.72401	2E-12	4E-11
DHTKD1	dehydrogenase E1 and transketolase doma	protein_coding	chr10:12068954-1	5295	8.367016902	14.93997796	0.750974	2E-12	4E-11
BMP5	bone morphogenetic protein 5	protein_coding	chr6:55753653-55	3970	57.60547328	95.27999873	0.639729	2E-12	4E-11
SMCO1	single-pass membrane protein with coilec	protein_coding	chr3:196506879-1	1846	6.666978808	13.52682444	0.932583	2E-12	4E-11

A2M	alpha-2-macroglobulin	protein_coding	chr12:9067664-91	6384	24.71912867	64.78722782	1.300054	2E-12	4E-11
TUBA1A	tubulin alpha 1a	protein_coding	chr12:49184795-4	3178	49.15184867	89.76570684	0.783329	2E-12	4E-11
CAMK2A	calcium/calmodulin dependent protein k	protein_coding	chr5:150219491-1	6150	16.58803633	26.69826912	0.600343	2E-12	4E-11
ENPP4	ectonucleotide pyrophosphatase/phosph	protein_coding	chr6:46129989-46	4644	5.538415862	10.34399254	0.812698	2E-12	4E-11
SLC2A4RG	SLC2A4 regulator	protein_coding	chr20:63739776-6	2874	19.20021302	31.46550976	0.626085	2E-12	4E-11
SPTLC2	serine palmitoyltransferase long chain ba	protein_coding	chr14:77505997-7	9143	3.949048117	7.01814675	0.742649	3E-12	4E-11
USP45	ubiquitin specific peptidase 45	protein_coding	chr6:99432379-99	8051	2.309201687	4.501865034	0.878412	3E-12	4E-11
SORCS1	sortilin related VPS10 domain containing	protein_coding	chr10:106573663-	9964	0.94581087	2.857277385	1.517471	3E-12	4E-11
MEX3C	mex-3 RNA binding family member C	protein_coding	chr18:51174550-5	7842	4.865379706	9.671640627	0.903385	3E-12	4E-11
LAMP2	lysosomal associated membrane protein 2	protein_coding	chrX:120427827-1	5526	14.41192292	23.25254769	0.603717	3E-12	4E-11
ARL1	ADP ribosylation factor like GTPase 1	protein_coding	chr12:101393116-	4935	12.47233984	23.83726101	0.849506	3E-12	4E-11
C11orf58	chromosome 11 open reading frame 58	protein_coding	chr11:16613132-1	10342	13.87508386	22.89646132	0.636617	3E-12	4E-11
MMAA	metabolism of cobalamin associated A	protein_coding	chr4:145599042-1	8158	0.645761174	1.76812534	1.369485	3E-12	4E-11
FAM49B	family with sequence similarity 49 memb	protein_coding	chr8:129839593-1	9132	3.55450403	6.619133508	0.810775	3E-12	4E-11
AP3S2	adaptor related protein complex 3 subuni	protein_coding	chr15:89830599-8	9237	1.760391093	3.334138225	0.837219	3E-12	5E-11
ZNF117	zinc finger protein 117	protein_coding	chr7:64971776-65	10691	1.455699341	2.905123357	0.910339	3E-12	5E-11
SBNO1	strawberry notch homolog 1	protein_coding	chr12:123289109-	11128	4.188471649	7.203034752	0.696847	3E-12	5E-11
TMEM87B	transmembrane protein 87B	protein_coding	chr2:112055269-1	5321	1.519079551	3.418700425	1.080731	3E-12	5E-11
DDX18	DEAD-box helicase 18	protein_coding	chr2:117814691-1	7808	4.612241754	7.8776411	0.68596	3E-12	5E-11
LRRC58	leucine rich repeat containing 58	protein_coding	chr3:120324509-1	7918	3.561614526	6.539812668	0.791598	3E-12	5E-11
FKBP10	FKBP prolyl isomerase 10	protein_coding	chr17:41812680-4	4273	20.10385212	33.63260095	0.655999	3E-12	5E-11
EFHD1	EF-hand domain family member D1	protein_coding	chr2:232606057-2	2675	6.455725793	14.52131882	1.083327	3E-12	5E-11
FBXL22	F-box and leucine rich repeat protein 22	protein_coding	chr15:63597353-6	3614	41.32313264	67.67045094	0.625508	3E-12	5E-11
SERP1	stress associated endoplasmic reticulum	protein_coding	chr3:150541998-1	5743	7.367762811	14.11131261	0.848453	3E-12	5E-11
HEATR5B	HEAT repeat containing 5B	protein_coding	chr2:36968383-37	8238	4.859190592	8.907899587	0.789197	3E-12	5E-11
ATP6AP2	ATPase H+ transporting accessory protein	protein_coding	chrX:40579372-40	9560	6.616286445	11.39127624	0.698652	3E-12	5E-11
IGSF11	immunoglobulin superfamily member 11	protein_coding	chr3:118900557-1	4899	0.144157252	1.13670179	2.879858	4E-12	5E-11
RPRD1A	regulation of nuclear pre-mRNA domain c	protein_coding	chr18:35984387-3	8442	5.176303175	8.784250877	0.676689	4E-12	5E-11
ESRRG	estrogen related receptor gamma	protein_coding	chr1:216503246-2	10912	4.045312557	6.818344715	0.667853	4E-12	5E-11
LDAH	lipid droplet associated hydrolase	protein_coding	chr2:20684014-20	4433	4.072962179	7.944202844	0.876036	4E-12	5E-11
CSMD1	CUB and Sushi multiple domains 1	protein_coding	chr8:2935353-499	18705	0.468590899	1.570944285	1.663718	4E-12	5E-11
ME2	malic enzyme 2	protein_coding	chr18:50879080-5	15897	5.860521504	11.06404699	0.83173	4E-12	6E-11
STIM1	stromal interaction molecule 1	protein_coding	chr11:3854527-40	7130	3.495502775	6.914077183	0.897303	4E-12	6E-11
SUSD2	sushi domain containing 2	protein_coding	chr22:24181487-2	5047	0.501827557	2.168269464	2.037759	4E-12	6E-11
TOM1L1	target of myb1 like 1 membrane traffickin	protein_coding	chr17:54899387-5	7275	2.10240163	3.911940194	0.808966	4E-12	6E-11
MARCH11	membrane associated ring-CH-type finger	protein_coding	chr5:16067139-16	1877	6.785137574	17.10558451	1.249505	4E-12	6E-11
ABRA	actin binding Rho activating protein	protein_coding	chr8:106759483-1	2754	3.206302352	8.412199528	1.300667	4E-12	6E-11
PTBP2	polypyrimidine tract binding protein 2	protein_coding	chr1:96721665-96	12937	1.795170649	3.569430542	0.907558	4E-12	6E-11
ARFGAP3	ADP ribosylation factor GTPase activating	protein_coding	chr22:42796502-4	3273	9.396147304	16.84906571	0.757725	4E-12	6E-11
PPP1R3A	protein phosphatase 1 regulatory subunit	protein_coding	chr7:113876777-1	4679	13.46426673	26.15247226	0.87143	5E-12	6E-11
FAM13B	family with sequence similarity 13 memb	protein_coding	chr5:137937960-1	6808	8.2805453	14.71423767	0.743459	5E-12	6E-11
NF1	neurofibromin 1	protein_coding	chr17:31094927-3	27130	4.248444919	8.083786559	0.843742	5E-12	6E-11
VIPR2	vasoactive intestinal peptide receptor 2	protein_coding	chr7:159028175-1	5132	4.161203861	7.909458321	0.840836	5E-12	6E-11
KIF3C	kinesin family member 3C	protein_coding	chr2:25926598-25	6401	0.342666338	1.532661344	2.070647	5E-12	7E-11
EARS2	glutamyl-tRNA synthetase 2, mitochondri	protein_coding	chr16:23522014-2	5730	3.495669985	6.86501544	0.884643	5E-12	7E-11
MBOAT1	membrane bound O-acyltransferase doma	protein_coding	chr6:20102145-20	1794	3.310904331	8.103894852	1.203647	5E-12	7E-11
CEP126	centrosomal protein 126	protein_coding	chr11:101915012-	8194	0.352797517	1.161793139	1.630341	5E-12	7E-11
CCND2-AS1	CCND2 antisense RNA 1	lncRNA	chr12:4247981-42	4397	1.431660286	3.984858788	1.39758	5E-12	7E-11
KBTBD4	kelch repeat and BTB domain containing 4	protein_coding	chr11:47572197-4	3263	4.958986148	9.594774677	0.863149	5E-12	7E-11
ZNF524	zinc finger protein 524	protein_coding	chr19:55600022-5	1441	7.117298142	14.74827303	0.962819	5E-12	7E-11
CARMN	cardiac mesoderm enhancer-associated n	lncRNA	chr5:149406689-1	18004	1.528640813	2.898728836	0.835468	5E-12	7E-11
HNRNPA1	heterogeneous nuclear ribonucleoprotein	protein_coding	chr12:54280193-5	5093	88.28541974	145.8777515	0.638963	5E-12	7E-11
PRMT2	protein arginine methyltransferase 2	protein_coding	chr21:46635595-4	7603	7.459958607	12.39973244	0.647727	5E-12	7E-11
KLHL15	kelch like family member 15	protein_coding	chrX:23983720-24	6272	3.592772354	7.013198614	0.878077	5E-12	7E-11
TMEM30A	transmembrane protein 30A	protein_coding	chr6:75252924-75	5103	19.71567784	33.241424	0.666519	5E-12	7E-11
BAIAP2-DT	BAIAP2 divergent transcript	lncRNA	chr17:81029130-8	4773	5.329204297	12.77019811	1.178158	6E-12	8E-11
MFSD6	major facilitator superfamily domain con	protein_coding	chr2:190408355-1	6031	1.33189198	3.512388262	1.306673	6E-12	8E-11
CADM1	cell adhesion molecule 1	protein_coding	chr11:115169218-	11017	7.800950028	12.71863838	0.619401	6E-12	8E-11
CCT2	chaperonin containing TCP1 subunit 2	protein_coding	chr12:69585426-6	4230	26.32928884	45.62299768	0.706118	6E-12	8E-11
CTDSP2	CTD small phosphatase 2	protein_coding	chr12:57819927-5	5432	26.29321712	46.48431282	0.737057	6E-12	8E-11
MCCC2	methylcrotonoyl-CoA carboxylase 2	protein_coding	chr5:71587288-71	6036	12.24194653	20.1230667	0.631022	6E-12	8E-11
AC008969.1	novel transcript, antisense to ZNF606	lncRNA	chr19:58002061-5	9172	1.998046681	4.397942341	1.048757	6E-12	8E-11
BCO2	beta-carotene oxygenase 2	protein_coding	chr11:112175467-	6789	3.068956298	6.555176237	1.007617	6E-12	8E-11
DCAF4	DDB1 and CUL4 associated factor 4	protein_coding	chr14:72926332-7	5581	0.972056254	2.43436213	1.232571	6E-12	9E-11
TRMT61A	tRNA methyltransferase 61A	protein_coding	chr14:103529196-	3217	3.256320547	6.704271703	0.956241	7E-12	9E-11
TM7SF3	transmembrane 7 superfamily member 3	protein_coding	chr12:26971579-2	5504	5.605838768	9.940830706	0.739147	7E-12	9E-11
ST6GALNAC3	ST6 N-acetylgalactosaminide alpha-2,6-si	protein_coding	chr1:76074746-76	7212	1.287410658	3.028275872	1.146839	7E-12	9E-11
EMB	embigin	protein_coding	chr5:50396192-50	5240	1.089384696	3.09284041	1.421246	7E-12	9E-11
DOK1	docking protein 1	protein_coding	chr2:74549026-74	3049	1.074699313	3.567893574	1.6343	7E-12	9E-11
GABPB1-AS1	GABPB1 antisense RNA 1	lncRNA	chr15:50354959-5	17179	2.645821273	4.985721582	0.827026	7E-12	9E-11
RIOK3	RIO kinase 3	protein_coding	chr18:23453287-2	4856	18.10371637	29.03051755	0.595325	7E-12	9E-11
DCTN4	dynactin subunit 4	protein_coding	chr5:150708440-1	6417	12.46651716	20.08709154	0.602873	7E-12	9E-11
SMIM10L2A	small integral membrane protein 10 like 2	protein_coding	chrX:135421944-1	5230	2.257178054	4.755994946	0.991727	7E-12	1E-10
BMF	Bcl2 modifying factor	protein_coding	chr15:40087890-4	5447	7.49385165	12.48696881	0.650667	8E-12	1E-10
GPCPD1	glycerophosphocholine phosphodiesteras	protein_coding	chr20:5544404-56	7527	5.786398424	9.904371882	0.690058	8E-12	1E-10
SNHG7	small nucleolar RNA host gene 7	lncRNA	chr9:136721366-1	4891	4.208019548	7.697645112	0.783172	8E-12	1E-10
SYNPO	synaptopodin	protein_coding	chr5:150601080-1	10243	9.72484139	15.15094857	0.55342	8E-12	1E-10
BIVM	basic, immunoglobulin-like variable moti	protein_coding	chr13:102799119-	7952	2.66433065	4.791990564	0.761123	8E-12	1E-10
RPA1	replication protein A1	protein_coding	chr17:1829702-19	5492	12.12168821	19.64592788	0.611034	8E-12	1E-10
GABPA	GA binding protein transcription factor s	protein_coding	chr21:25734570-2	5429	4.690166546	8.752171737	0.812135	8E-12	1E-10
C15orf41	chromosome 15 open reading frame 41	protein_coding	chr15:36579626-3	9278	1.999098639	3.718676825	0.809216	8E-12	1E-10
SUZ12	SUZ12 polycomb repressive complex 2 su	protein_coding	chr17:31937007-3	6551	9.371729022	16.02818802	0.688564	9E-12	1.06147
PGAP3	post-GPI attachment to proteins 3	protein_coding	chr17:39671122-3	3619	6.406465809	11.52705804	0.760729	9E-12	1.13052
FAM102B	family with sequence similarity 102 mem	protein_coding	chr1:108560089-1	9350	1.437399278	2.800571875	0.874733	9E-12	1.14029
SCAMP1-AS1	SCAMP1 antisense RNA 1	lncRNA	chr5:78342365-78	5028	1.321752606	2.979362656	1.082799	1E-11	1.25596
NLGN4X	neuroligin 4 X-linked	protein_coding	chrX:5840637-622	7439	0.237362091	1.175352068	2.231463	1E-11	1.26881
MGAT3	mannosyl (beta-1,4-)-glycoprotein beta-1,	protein_coding	chr22:39457012-3	5789	5.315882686	11.70622862	1.053201	1E-11	1.31949
MAP1B	microtubule associated protein 1B	protein_coding	chr5:72107234-72	12540	14.52425531	25.71918427	0.738083	1E-11	1.32348
ARFGEF1	ADP ribosylation factor guanine nucleotic	protein_coding	chr8:67173511-67	11059	5.21327498	8.70933555	0.654622	1E-11	1.32667
PER3	period circadian regulator 3	protein_coding	chr1:7784320-784	8099	3.069197244	5.244242849	0.686963	1E-11	1.34592
SEMA3C	semaphorin 3C	protein_coding	chr7:80742538-80	6100	2.682779647	5.461931733	0.938543	1E-11	1.34669
CA11	carbonic anhydrase 11	protein_coding	chr19:48637946-4	1946	4.529097804	9.995550778	1.050679	1E-11	1.45085
ABRAXAS1	abraxas 1, BRCA1 A complex subunit	protein_coding	chr4:83459517-83	7699	1.671101802	3.32573522	0.905472	1E-11	1.47343
CAMK2G	calcium/calmodulin dependent protein k	protein_coding	chr10:73812501-7	6780	2.715094912	5.322143068	0.885443	1E-11	1.48637
TEX261	testis expressed 261	protein_coding	chr2:70985942-70	4184	10.43387066	17.14643318	0.630628	1E-11	1.49199
LZIC	leucine zipper and CTNNBIP1 domain con	protein_coding	chr1:9922113-994	5459	8.190282687	13.47913013	0.631622	1E-11	1.50555
PAPSS1	3'-phosphoadenosine 5'-phosphosulfate s	protein_coding	chr4:107590276-1	3260	4.164005769	8.623737644	0.960322	1E-11	1.50555
ZFYVE19	zinc finger FYVE-type containing 19	protein_coding	chr15:40807086-4	4779	4.745611805	8.572304585	0.767831	1E-11	1.50604
TNFRSF19	TNF receptor superfamily member 19	protein_coding	chr13:23570370-2	5113	9.499715618	17.03590877	0.756111	1E-11	1.51752
ROCK2	Rho associated coiled-coil containing pro	protein_coding	chr2:11179759-11	10198	6.175962716	9.849356754	0.587213	1E-11	1.53418
MKRN1	makorin ring finger protein 1	protein_coding	chr7:140453033-1	5843	31.85058739	48.66846469	0.525218	1E-11	1.53748
DOCK4	dedicator of cytokinesis 4	protein_coding	chr7:111726110-1	11931	1.8188904	3.174029969	0.715503	1E-11	1.54643
C12orf57	chromosome 12 open reading frame 57	protein_coding	chr12:6942978-69	1806	12.75302491	27.15985333	1.002992	1E-11	1.54882
ZNF561	zinc finger protein 561	protein_coding	chr19:9604680-96	6304	6.029156258	11.1648859	0.805168	1E-11	1.56083
HSDL2	hydroxysteroid dehydrogenase like 2	protein_coding	chr9:112380080-1	3307	48.84238551	73.23205075	0.498025	1E-11	1.62184
TMCC1-AS1	TMCC1 antisense RNA 1 (head to head)	lncRNA	chr3:129893811-1	7702	0.364692428	1.260891963	1.697301	1E-11	1.62657
STIM2	stromal interaction molecule 2	protein_coding	chr4:26857601-27	8386	1.539308085	3.004812781	0.876547	1E-11	1.65083
MIR133A1HG	MIR133A1 host gene	lncRNA	chr18:21825487-2	5825	7.092210318	12.50182259	0.730252	1E-11	1.70686
MBNL1	muscleblind like splicing regulator 1	protein_coding	chr3:152243828-1	9989	9.614801698	15.4650787	0.599372	1E-11	1.71055
EPDR1	ependymin related 1	protein_coding	chr7:37683843-37	2780	21.41130997	34.80436134	0.613277	1E-11	1.71305
GRWD1	glutamate rich WD repeat containing 1	protein_coding	chr19:48445773-4	5763	3.103857923	5.587576188	0.761334	2E-11	1.77729

MTUS2	microtubule associated scaffold protein 2	protein_coding	chr13:28820348-2	8445	22.65814884	35.68455713	0.569284	2E-11	1.78710
SUSD1	sushi domain containing 1	protein_coding	chr9:112040785-1	4183	2.737539741	5.704412383	0.972944	2E-11	1.79714
SSX2IP	SSX family member 2 interacting protein	protein_coding	chr1:84643707-84	8639	4.522822402	7.724950392	0.685202	2E-11	1.80584
COPG2	coatomer protein complex subunit gamm	protein_coding	chr7:130506238-1	4139	5.936012879	11.39481114	0.857339	2E-11	1.80766
SNHG8	small nucleolar RNA host gene 8	lncRNA	chr4:118278709-1	6608	1.443696268	2.884920727	0.910704	2E-11	1.87306
TCTA	T cell leukemia translocation altered	protein_coding	chr3:49412206-49	2747	8.230108668	14.9051264	0.76715	2E-11	1.87351
UNC119	unc-119 lipid binding chaperone	protein_coding	chr17:28546707-2	5128	5.513557105	9.592185609	0.712607	2E-11	1.88078
NLN	neurolysin	protein_coding	chr5:65722205-65	10939	1.5561706	2.838492955	0.780848	2E-11	1.91990
KPNA3	karyopherin subunit alpha 3	protein_coding	chr13:49699320-4	4222	22.9874511	36.5312473	0.582666	2E-11	1.92628
SPACA6	sperm acrosome associated 6	protein_coding	chr19:51689128-5	4103	3.54538978	7.631454217	1.02452	2E-11	1.95579
CRIP2	cysteine rich protein 2	protein_coding	chr14:105472962-	4837	298.881069	489.2465729	0.625464	2E-11	2.00710
SRSF12	serine and arginine rich splicing factor 12	protein_coding	chr6:89095959-89	4538	0.976784646	2.43994993	1.232693	2E-11	2.01476
GPR183	G protein-coupled receptor 183	protein_coding	chr13:99294539-9	1685	0.97667224	4.017139619	1.961315	2E-11	2.02097
DNAJA1	DnaJ heat shock protein family (Hsp40) m	protein_coding	chr9:33025273-33	2668	46.30428903	76.94409412	0.646492	2E-11	2.02229
PDSS2	decaprenyl diphosphate synthase subunit	protein_coding	chr6:107152562-1	3921	6.158776739	11.1241878	0.764293	2E-11	2.07088
TUBB6	tubulin beta 6 class V	protein_coding	chr18:12307669-1	4117	33.58155102	56.21883107	0.657817	2E-11	2.08762
RXYLT1	ribitol xylosyltransferase 1	protein_coding	chr12:63779803-6	7804	1.626556949	3.193417467	0.885935	2E-11	2.08814
ID1	inhibitor of DNA binding 1, HLH protein	protein_coding	chr20:31605283-3	1233	3.455887612	10.59494518	1.527062	2E-11	2.08943
PAQR3	progestin and adipoQ receptor family mer	protein_coding	chr4:78887127-78	12403	2.141863995	3.643522971	0.680583	2E-11	2.10546
EDEM1	ER degradation enhancing alpha-mannosi	protein_coding	chr3:5187646-521	6737	2.620817468	5.024315014	0.851528	2E-11	2.10579
FARP2	FERM, ARH/RhoGEF and pleckstrin domai	protein_coding	chr2:241356285-2	14371	1.221582505	2.429748049	0.904437	2E-11	2.12081
JAK1	Janus kinase 1	protein_coding	chr1:64833229-64	6362	16.74677091	25.39835991	0.514525	2E-11	2.12802
SELENON	selenoprotein N	protein_coding	chr1:25800176-25	4421	23.54815606	40.67919233	0.703468	2E-11	2.12906
C2orf69	chromosome 2 open reading frame 69	protein_coding	chr2:199911293-1	4259	4.314070773	7.764754927	0.764463	2E-11	2.14773
UTRN	utrophin	protein_coding	chr6:144285701-1	14604	9.152733299	17.91829595	0.886092	2E-11	2.17162
UBA6	ubiquitin like modifier activating enzyme	protein_coding	chr4:67612652-67	11408	2.488128669	4.079506186	0.626737	2E-11	2.19561
STX2	syntaxin 2	protein_coding	chr12:130789600-	3723	6.579872096	12.34744108	0.821265	2E-11	2.24374
MAP3K12	mitogen-activated protein kinase kinase k	protein_coding	chr12:53479669-5	7971	1.749702238	3.83460543	1.047947	2E-11	2.35129
ABHD10	abhydrolase domain containing 10	protein_coding	chr3:111979010-1	3026	5.679296106	10.82002104	0.846984	2E-11	2.39843
FUBP3	far upstream element binding protein 3	protein_coding	chr9:130578965-1	6603	12.74276396	19.79591557	0.548932	2E-11	2.39909
KDM1B	lysine demethylase 1B	protein_coding	chr6:18155329-18	5626	3.066881505	5.375048376	0.722207	2E-11	2.41613
LINC01569	long intergenic non-protein coding RNA 1	lncRNA	chr16:4243943-42	5237	1.35896056	3.764902138	1.378839	2E-11	2.42422
GATC	glutamyl-tRNA amidotransferase subunit	protein_coding	chr12:120446444-	4416	3.50484867	6.738206413	0.856491	2E-11	2.46526
CPEB2	cytoplasmic polyadenylation element bin	protein_coding	chr4:15002674-15	7177	6.767126813	10.54116249	0.552392	2E-11	2.47281
AKAP11	A-kinase anchoring protein 11	protein_coding	chr13:42272152-4	9915	8.669557463	14.37135121	0.64368	2E-11	2.47310
ABHD14B	abhydrolase domain containing 14B	protein_coding	chr3:51968510-51	3161	2.490383676	5.662143438	1.097133	2E-11	2.49174
RAB33B	RAB33B, member RAS oncogene family	protein_coding	chr4:139453232-1	5028	1.644912552	3.422932479	0.968911	2E-11	2.51802
SLC27A1	solute carrier family 27 member 1	protein_coding	chr19:17468769-1	8204	2.422332169	4.807282443	0.903812	2E-11	2.54481
AC068733.3	novel transcript, antisense to PRKCDBP	lncRNA	chr11:6319140-63	4764	3.509276033	7.252645302	0.959933	2E-11	2.56961
ZBTB46	zinc finger and BTB domain containing 46	protein_coding	chr20:63743666-6	7068	5.753631019	10.03122069	0.716266	2E-11	2.56961
AP4M1	adaptor related protein complex 4 subun	protein_coding	chr7:100101549-1	4722	3.499420915	6.225164693	0.744793	2E-11	2.57623
LSM14B	LSM family member 14B	protein_coding	chr20:62122461-6	3377	17.96014723	28.19499529	0.564688	3E-11	2.63869
SMAD9	SMAD family member 9	protein_coding	chr13:36844831-3	5723	3.294078989	7.350590596	1.076889	3E-11	2.69713
RAPGEFL1	Rap guanine nucleotide exchange factor li	protein_coding	chr17:40177010-4	5711	2.462850057	4.684331773	0.843244	3E-11	2.70835
CUL7	cullin 7	protein_coding	chr6:43037617-43	5628	9.234924443	15.9800768	0.7066	3E-11	2.76279
NAP1L2	nucleosome assembly protein 1 like 2	protein_coding	chrX:73212299-73	2550	2.433479292	5.208863684	1.011938	3E-11	2.76279
TMEM38B	transmembrane protein 38B	protein_coding	chr9:105694541-1	4531	10.6617727	17.80879156	0.653711	3E-11	2.76279
HSPA1B	heat shock protein family A (Hsp70) meml	protein_coding	chr6:31827738-31	2517	14.47580319	24.90792348	0.694644	3E-11	2.79710
PQLC3	PQ loop repeat containing 3	protein_coding	chr2:11155198-11	2271	1.02753195	3.275109043	1.589342	3E-11	2.88086
KDM2B	lysine demethylase 2B	protein_coding	chr12:121429096-	13632	1.774464917	3.044840545	0.691369	3E-11	2.91851
C5orf30	chromosome 5 open reading frame 30	protein_coding	chr5:103258763-1	3507	2.929153309	5.867101442	0.91935	3E-11	2.92113
PPP1R9B	protein phosphatase 1 regulatory subunit	protein_coding	chr17:50133737-5	4343	14.1088391	24.30144383	0.697657	3E-11	2.92113
QSOX1	quiescin sulfhydryl oxidase 1	protein_coding	chr1:180154869-1	9276	4.783369216	8.422829815	0.729972	3E-11	2.92113
MINK1	misshapen like kinase 1	protein_coding	chr17:4833340-48	7865	8.055611772	13.99155785	0.710962	3E-11	2.95944
BLMH	bleomycin hydrolase	protein_coding	chr17:30248203-3	5119	4.762162499	9.645603862	0.928462	3E-11	3.01826
PRR36	proline rich 36	protein_coding	chr19:7868719-78	4467	3.409686333	6.380301907	0.81957	3E-11	3.03267
ZFAND3	zinc finger AN1-type containing 3	protein_coding	chr6:37819727-38	3808	24.18120554	36.77213816	0.518556	3E-11	3.09658
ATG12	autophagy related 12	protein_coding	chr5:115828200-1	8867	3.017182149	5.13435815	0.682304	3E-11	3.11257
AMZ2	archaelysin family metallopeptidase 2	protein_coding	chr17:68247930-6	3858	13.53117139	21.55861127	0.585708	3E-11	3.11757
JRKL	JRK like	protein_coding	chr11:96389989-9	3815	1.545071205	3.689032333	1.165958	3E-11	3.11757
LITAF	lipopolysaccharide induced TNF factor	protein_coding	chr16:11547722-1 5060		1.605971752	3.676951634	1.110648	3E-11	3.12412
PWWP3A	PWWP domain containing 3A, DNA repair	protein_coding	chr19:1354711-13	9876	3.640894242	6.401698232	0.727026	3E-11	3.12412
VWA1	von Willebrand factor A domain containi	protein_coding	chr1:1434861-144	5065	0.445875325	1.582227489	1.743693	3E-11	3.12602
DTWD1	DTW domain containing 1	protein_coding	chr15:49621037-4	14996	2.369920555	4.26036487	0.760874	3E-11	3.32691
CASQ2	calsequestrin 2	protein_coding	chr1:115700007-1	2769	9.822532256	16.63348412	0.671445	3E-11	3.35931
SAMD8	sterile alpha motif domain containing 8	protein_coding	chr10:75099586-7	7163	2.766578708	5.218349016	0.827278	3E-11	3.35931
EIF2AK4	eukaryotic translation initiation factor 2 a	protein_coding	chr15:39934115-4	11988	6.533875977	10.00940278	0.529061	3E-11	3.40606
ZNF575	zinc finger protein 575	protein_coding	chr19:43525497-4	3222	0.231071202	1.234730452	2.309965	3E-11	3.40606
ACOT2	acyl-CoA thioesterase 2	protein_coding	chr14:73567620-7	2393	46.02926196	77.20387974	0.658657	3E-11	3.43316
SUCLG2	succinate-CoA ligase GDP-forming beta su	protein_coding	chr3:67360460-67 3090		11.65758788	19.37869581	0.647803	4E-11	3.50472
MARCKSL1	MARCKS like 1	protein_coding	chr1:32333839-32	1546	47.05919885	72.83026227	0.543476	4E-11	3.51065
RCN2	reticulocalbin 2	protein_coding	chr15:76931738-7	9901	5.444860818	8.856796685	0.61465	4E-11	3.54152
PLP2	proteolipid protein 2	protein_coding	chrX:49171898-49	1303	23.03833755	45.30768271	0.893315	4E-11	3.55056
PNMA1	PNMA family member 1	protein_coding	chr14:73711783-7	2590	37.76961821	56.94750651	0.506433	4E-11	3.58089
TSEN34	tRNA splicing endonuclease subunit 34	protein_coding	chr19:54189938-5	3542	10.9459907	17.3577479	0.578046	4E-11	3.64676
GRPEL2	GrpE like 2, mitochondrial	protein_coding	chr5:149345430-1	4419	2.07419068	4.057908552	0.882959	4E-11	3.68491
MORC3	MORC family CW-type zinc finger 3	protein_coding	chr21:36320189-3	6408	3.533621996	5.914153297	0.656682	4E-11	3.68493
EHD4	EH domain containing 4	protein_coding	chr15:41895933-4	7864	18.52578925	28.5784828	0.539366	4E-11	3.71699
SLC2A3	solute carrier family 2 member 3	protein_coding	chr12:7919230-79	6071	13.01538001	21.33062623	0.627624	4E-11	3.76324
BAX	BCL2 associated X, apoptosis regulator	protein_coding	chr19:48954815-4	2587	18.33975738	29.00602829	0.574332	4E-11	3.77514
ZNF467	zinc finger protein 467	protein_coding	chr7:149764182-1	2843	5.269085486	10.45070495	0.902757	4E-11	3.78582
ARHGEF3	Rho guanine nucleotide exchange factor 3	protein_coding	chr3:56727418-57	6594	4.210594745	6.847113565	0.6152	4E-11	3.90651
ZNHIT6	zinc finger HIT-type containing 6	protein_coding	chr1:85649417-85	6203	1.507300124	3.143443588	0.976407	4E-11	3.96403
CHML	CHM like Rab escort protein	protein_coding	chr1:241628853-2	7661	4.559858678	7.669491686	0.664837	4E-11	4.03952
CD276	CD276 molecule	protein_coding	chr15:73683966-7	5482	11.59919371	18.06029623	0.552852	4E-11	4.04937
CKAP4	cytoskeleton associated protein 4	protein_coding	chr12:106237881-	3666	32.63960755	51.38624025	0.568158	4E-11	4.06585
ICA1L	islet cell autoantigen 1 like	protein_coding	chr2:202773150-2	10598	0.60618002	1.390212982	1.114023	4E-11	4.08909
ACOT1	acyl-CoA thioesterase 1	protein_coding	chr14:73537143-7	1686	9.594141084	17.68369335	0.794061	4E-11	4.11733
PDCL3	phosducin like 3	protein_coding	chr2:100562993-1	1532	9.983222749	18.37756753	0.791662	4E-11	4.14438
ANKEF1	ankyrin repeat and EF-hand domain conta	protein_coding	chr20:9986126-10	5869	0.955034013	2.497921429	1.315324	4E-11	4.16485
CDK2AP2	cyclin dependent kinase 2 associated prot	protein_coding	chr11:67506497-6	2019	15.61684878	30.83672073	0.898738	4E-11	4.29897
CNOT7	CCR4-NOT transcription complex subunit	protein_coding	chr8:17224966-17	10941	4.499063542	6.962985561	0.543566	5E-11	4.37590
CARS2	cysteinyl-tRNA synthetase 2, mitochondri	protein_coding	chr13:110641412-	10436	4.547474293	7.133688874	0.563502	5E-11	4.43807
HLCS	holocarboxylase synthetase	protein_coding	chr21:36750888-3	7889	2.595127073	4.704020109	0.770435	5E-11	4.45401
SLC25A24	solute carrier family 25 member 24	protein_coding	chr1:108134043-1	6726	4.216438487	7.469596264	0.736517	5E-11	4.46211
ANKRD13C	ankyrin repeat domain 13C	protein_coding	chr1:70258999-70	6184	4.32690418	7.139402313	0.634294	5E-11	4.48026
SERPINI1	serpin family I member 1	protein_coding	chr3:167735243-1	2418	5.508018882	10.74507086	0.882202	5E-11	4.48026
ADM	adrenomedullin	protein_coding	chr11:10305073-1	2325	34.2620583	57.49248147	0.660519	5E-11	4.51099
SVBP	small vasohibin binding protein	protein_coding	chr1:42807052-42	1353	6.508546937	13.05595232	0.918068	5E-11	4.53070
B3GNTL1	UDP-GlcNAc:betaGal beta-1,3-N-acetylglu	protein_coding	chr17:82942155-8	6891	0.657853776	1.725997402	1.296472	5E-11	4.58360
MBOAT7	membrane bound O-acyltransferase doma	protein_coding	chr19:54173412-5	5292	6.120289262	10.14719951	0.644814	5E-11	4.58360
CERS5	ceramide synthase 5	protein_coding	chr12:50129306-5	6749	3.790901378	6.168417805	0.614583	5E-11	4.60432
MAP3K11	mitogen-activated protein kinase kinase k	protein_coding	chr11:65597756-6	10563	3.599429977	6.188687715	0.695719	5E-11	4.60432
SNAPC2	small nuclear RNA activating complex pol	protein_coding	chr19:7920338-79	1978	5.740493826	11.91246103	0.972441	5E-11	4.62147
KLHL5	kelch like family member 5	protein_coding	chr4:39045039-39	5144	3.942652705	6.823587181	0.705006	5E-11	4.62602
CNOT11	CCR4-NOT transcription complex subunit	protein_coding	chr2:101252886-1	2702	96.49995311	141.3573986	0.464646	5E-11	4.78925
ZBED5-AS1	ZBED5 antisense RNA 1	lncRNA	chr11:10858179-1	4599	2.548363966	4.900733014	0.857666	5E-11	4.80467
EEA1	early endosome antigen 1	protein_coding	chr12:92770637-9 10150		4.873741452	7.893182287	0.609978	5E-11	4.87641
MAGED1	MAGE family member D1	protein_coding	chrX:51803007-51	6016	56.68295315	85.87530554	0.513324	5E-11	4.89597
HSD3B7	hydroxy-delta-5-steroid dehydrogenase, 3	protein_coding	chr16:30985207-3	2490	0.72731908	2.612424391	1.760149	5E-11	4.97763
SERTAD3	SERTA domain containing 3	protein_coding	chr19:40440844-4	1848	19.09162816	31.61544236	0.640201	5E-11	4.98408

CYB561D1	cytochrome b561 family member D1	protein_coding	chr1:109494052-1	5690	1.251461261	3.088789603	1.214905	5E-11	5.02673
CTBP2	C-terminal binding protein 2	protein_coding	chr10:124984317-	11056	3.40541608	5.419989065	0.582516	5E-11	5.03872
UNG	uracil DNA glycosylase	protein_coding	chr12:109097574-	2567	11.427838	19.48867254	0.680987	5E-11	5.04758
OGA	O-GlcNAcase	protein_coding	chr10:101784443-	7247	10.47707365	24.20877157	1.128014	5E-11	5.07957
TMEM150C	transmembrane protein 150C	protein_coding	chr4:82483170-82	3497	0.341971566	1.621226117	2.146578	5E-11	5.07957
SP3	Sp3 transcription factor	protein_coding	chr2:173880850-1	17223	2.608717523	4.345294677	0.650811	6E-11	5.14450
NLK	nemo like kinase	protein_coding	chr17:28041737-2	4769	10.22353561	15.58498025	0.522193	6E-11	5.15496
NRTN	neurturin	protein_coding	chr19:5823802-58	1109	20.90034043	47.63793344	1.100947	6E-11	5.26177
CASP7	caspase 7	protein_coding	chr10:113679162-	3528	7.355410251	13.28369152	0.765286	6E-11	5.26329
SBF2-AS1	SBF2 antisense RNA 1	lncRNA	chr11:9758268-98	5177	1.570544684	3.346544621	1.004006	6E-11	5.29945
GLCCI1	glucocorticoid induced 1	protein_coding	chr7:7968796-809	6883	4.022777271	7.124468755	0.738318	6E-11	5.32313
GPR22	G protein-coupled receptor 22	protein_coding	chr7:107470018-1	3711	28.13280724	44.93223071	0.589133	6E-11	5.33449
TMEM250	transmembrane protein 250	protein_coding	chr9:136114581-1	3408	8.650891878	15.17721731	0.72848	6E-11	5.34450
RBFOX2	RNA binding fox-1 homolog 2	protein_coding	chr22:35738736-3	10413	18.50349926	28.01083326	0.51223	6E-11	5.37155
CAMKK2	calcium/calmodulin dependent protein k	protein_coding	chr12:121237675-	7173	3.018288376	5.128932588	0.679326	6E-11	5.39118
CLPB	ClpB homolog, mitochondrial AAA ATPase	protein_coding	chr11:72285495-7	12428	1.784782252	2.994947787	0.659043	6E-11	5.44603
SEMA7A	semaphorin 7A (John Milton Hagen blood	protein_coding	chr15:74409289-7	3800	1.011101898	2.632323336	1.285398	6E-11	5.47464
TGFB1I1	transforming growth factor beta 1 induce	protein_coding	chr16:31471585-3	5403	14.89227082	27.04153683	0.77634	6E-11	5.49098
ADD2	adducin 2	protein_coding	chr2:70607618-70	12673	5.941635907	10.36074087	0.716897	6E-11	5.56101
ITGB1BP2	integrin subunit beta 1 binding protein 2	protein_coding	chrX:71301750-71	2185	65.33156834	101.21696	0.544769	6E-11	5.58427
MYADM	myeloid associated differentiation marker	protein_coding	chr19:53864763-5	4298	52.82206433	80.96492122	0.529546	6E-11	5.58427
LAPTM4A	lysosomal protein transmembrane 4 alpha	protein_coding	chr2:20032650-20	1546	70.0955376	105.3181493	0.500941	6E-11	5.60256
RASSF3	Ras association domain family member 3	protein_coding	chr12:64507001-6	4241	10.97879052	16.53992218	0.505285	6E-11	5.61927
GPR63	G protein-coupled receptor 63	protein_coding	chr6:96794125-96	5967	1.771519977	3.926654987	1.063637	6E-11	5.67253
SHD	Src homology 2 domain containing transf	protein_coding	chr19:4278601-42	2811	125.7945011	191.0469411	0.516816	6E-11	5.72752
ARHGAP28	Rho GTPase activating protein 28	protein_coding	chr18:6729718-69	11910	0.468441368	1.158138996	1.214894	7E-11	5.87658
SCN2B	sodium voltage-gated channel beta subun	protein_coding	chr11:118157849-	10023	2.190893127	3.593706985	0.627974	7E-11	5.92358
USP3	ubiquitin specific peptidase 3	protein_coding	chr15:63504511-6	7764	2.609142812	4.365830871	0.656429	7E-11	5.95540
NR3C1	nuclear receptor subfamily 3 group C men	protein_coding	chr5:143277931-1	9161	3.111990275	4.986904423	0.592516	7E-11	6.08762
KLF2	Kruppel like factor 2	protein_coding	chr19:16324817-1	2018	1.777452819	4.638238458	1.300769	7E-11	6.21714
ELOVL5	ELOVL fatty acid elongase 5	protein_coding	chr6:53267398-53	3813	11.12882794	17.61140285	0.575279	7E-11	6.35692
UBE2Q2	ubiquitin conjugating enzyme E2 Q2	protein_coding	chr15:75843285-7	3852	17.2740743	26.60321772	0.537105	7E-11	6.39340
CDKN1A	cyclin dependent kinase inhibitor 1A	protein_coding	chr6:36676460-36 3050		275.6477276	425.9228466	0.541797	7E-11	6.51079
NIPSNAP1	nipsnap homolog 1	protein_coding	chr22:29554808-2	3067	17.17530103	26.93174927	0.564223	7E-11	6.51343
RPGR	retinitis pigmentosa GTPase regulator	protein_coding	chrX:38269170-38	8351	0.896321277	1.807522021	0.928084	7E-11	6.55354
RAB9B	RAB9B, member RAS oncogene family	protein_coding	chrX:103822327-1	3772	15.17740618	23.21739981	0.526	8E-11	6.66822
GJA1	gap junction protein alpha 1	protein_coding	chr6:121435595-1	3395	144.2629657	227.6579537	0.572735	8E-11	6.79179
IDS	iduronate 2-sulfatase	protein_coding	chrX:149476988-1	9294	0.804212052	1.561065574	0.867678	8E-11	6.85393
NAA50	N(alpha)-acetyltransferase 50, NatE cataly	protein_coding	chr3:113716458-1	6438	15.20937848	22.42071437	0.474041	8E-11	6.85393
RASA3	RAS p21 protein activator 3	protein_coding	chr13:113977783-	4775	3.113410428	6.049688663	0.872462	8E-11	6.95412
RTL8A	retrotransposon Gag like 8A	protein_coding	chrX:135050932-1	1265	46.67440137	73.60648296	0.570902	8E-11	6.98481
LATS2	large tumor suppressor kinase 2	protein_coding	chr13:20973036-2	5613	10.73003488	18.23225142	0.67992	8E-11	7.19370
OGFRL1	opioid growth factor receptor like 1	protein_coding	chr6:71288811-71	9176	1.871477527	3.131068072	0.656089	8E-11	7.23065
PRRT3	proline rich transmembrane protein 3	protein_coding	chr3:9945542-995	3953	1.569165348	3.514606934	1.070696	8E-11	7.24256
VMP1	vacuole membrane protein 1	protein_coding	chr17:59707192-5	6854	5.734846494	8.715701742	0.518163	8E-11	7.29069
ZFP36L1	ZFP36 ring finger protein like 1	protein_coding	chr14:68787660-6	5160	38.86860743	61.00791832	0.564186	8E-11	7.29069
TWSG1	twisted gastrulation BMP signaling modul	protein_coding	chr18:9334767-94	4435	9.921549668	15.29629525	0.537687	9E-11	7.47747
FBXL3	F-box and leucine rich repeat protein 3	protein_coding	chr13:76992598-7	7076	2.980141989	5.105439368	0.689475	9E-11	7.53731
TUT4	terminal uridylyl transferase 4	protein_coding	chr1:52408282-52	16199	4.492542503	6.68571699	0.487725	9E-11	7.53731
CORO1B	coronin 1B	protein_coding	chr11:67435510-6	5327	4.837100191	8.015765306	0.643088	9E-11	7.56304
RUFY3	RUN and FYVE domain containing 3	protein_coding	chr4:70704204-70	9405	2.585125012	4.374101353	0.672269	9E-11	7.60315
POGK	pogo transposable element derived with k	protein_coding	chr1:166839447-1	8448	7.82734741	12.36072359	0.572581	9E-11	7.77752
NEIL2	nei like DNA glycosylase 2	protein_coding	chr8:11769639-11	3366	3.043632644	7.516911552	1.211536	9E-11	7.80960
F2R	coagulation factor II thrombin receptor	protein_coding	chr5:76716126-76	3972	1.586377708	3.376054788	1.006344	9E-11	7.85340
LMBR1	limb development membrane protein 1	protein_coding	chr7:156668946-1	11819	10.38130915	15.37888339	0.480981	9E-11	7.87263
SUMF1	sulfatase modifying factor 1	protein_coding	chr3:3700814-446	6944	2.184881393	3.768861813	0.701906	9E-11	7.90882
SARDH	sarcosine dehydrogenase	protein_coding	chr9:133663560-1	5118	0.251288241	1.081482544	2.026427	9E-11	7.99138
SNHG16	small nucleolar RNA host gene 16	lncRNA	chr17:76557764-7	5364	12.96559161	21.10612363	0.617719	9E-11	7.99138
HIST1H2BG	histone cluster 1 H2B family member g	protein_coding	chr6:26215159-26	1534	1.197117147	4.493337549	1.831108	9E-11	8.03078
FMNL2	formin like 2	protein_coding	chr2:152335174-1	6574	2.138607555	3.732172915	0.719259	1E-10	8.06866
ATP11C	ATPase phospholipid transporting 11C	protein_coding	chrX:139726346-1	7407	1.803934248	3.473709588	0.856982	1E-10	8.15275
MVP	major vault protein	protein_coding	chr16:29820394-2	6141	2.486779272	4.758251895	0.852275	1E-10	8.25176
FBXO41	F-box protein 41	protein_coding	chr2:73254682-73	8011	1.284991652	2.777764345	1.026581	1E-10	8.26298
IRF6	interferon regulatory factor 6	protein_coding	chr1:209785617-2	4971	1.96629241	3.609763562	0.787496	1E-10	8.29999
LASP1	LIM and SH3 protein 1	protein_coding	chr17:38869859-3	7061	4.803344472	8.942320967	0.812828	1E-10	8.33928
EIF1AX	eukaryotic translation initiation factor 1A	protein_coding	chrX:20124525-20	4414	18.64213016	29.73974798	0.587721	1.00314	8.39127
CRK	CRK proto-oncogene, adaptor protein	protein_coding	chr17:1420689-14	4064	9.014111089	14.32510321	0.581107	1.01225	8.45025
FCHSD2	FCH and double SH3 domains 2	protein_coding	chr11:72836745-7	5245	10.8642704	16.62801536	0.527752	1.01815	8.48133
PGM2	phosphoglucomutase 2	protein_coding	chr4:37826660-37	3677	2.748044916	6.055276285	1.047663	1.03591	8.61127
ALDH5A1	aldehyde dehydrogenase 5 family membe	protein_coding	chr6:24494852-24	5525	8.535076713	13.65661885	0.592974	1.03880	8.62937
MACROD2	mono-ADP ribosylhydrolase 2	protein_coding	chr20:13995369-1	11428	0.562910929	1.224472074	1.035711	1.09224	9.04195
GCK	glucokinase	protein_coding	chr7:44144271-44	4138	1.052405039	2.266424929	1.019308	1.09604	9.06713
FAM222A	family with sequence similarity 222 meml	protein_coding	chr12:109714228-	3722	7.932708096	14.1671464	0.752817	1.09870	9.08290
CERCAM	cerebral endothelial cell adhesion molecu	protein_coding	chr9:128411751-1	6059	4.911529657	7.727207067	0.567907	1.11605	9.21360
RABEPK	Rab9 effector protein with kelch motifs	protein_coding	chr9:125200542-1	3401	2.153837707	4.933259408	1.105707	1.11882	9.22379
NCBP2	nuclear cap binding protein subunit 2	protein_coding	chr3:196935402-1	5111	10.16082165	15.22768456	0.49803	1.20623	9.80282
SLC22A17	solute carrier family 22 member 17	protein_coding	chr14:23346306-2	4278	5.666839099	9.264794896	0.623861	1.21173	9.84083
NUDCD3	NudC domain containing 3	protein_coding	chr7:44379119-44	9893	8.426279616	12.88771134	0.527109	1.21626	9.87091
NBPF15	NBPF member 15	protein_coding	chr1:144421386-1	5306	3.633126619	5.87038847	0.605441	1.23662	1E-09
TTLL3	tubulin tyrosine ligase like 3	protein_coding	chr3:9808086-985	10404	0.874529551	2.092644287	1.169082	1.24236	1E-09
KAT5	lysine acetyltransferase 5	protein_coding	chr11:65711996-6	3732	6.837302706	11.22637985	0.631868	1.24926	1E-09
TEF	TEF transcription factor, PAR bZIP family r	protein_coding	chr22:41367333-4	4702	5.801493118	9.134628773	0.569015	1.25086	1E-09
TMEM131L	transmembrane 131 like	protein_coding	chr4:153466346-1	6393	3.078823093	5.407823748	0.725315	1.25082	1E-09
FAM3C	family with sequence similarity 3 member	protein_coding	chr7:121348878-1	3395	10.23684398	16.67618081	0.615962	1.25198	1E-09
ATP6V0E2	ATPase H+ transporting V0 subunit e2	protein_coding	chr7:149872968-1	6751	8.381423918	14.55760332	0.712518	1.29806	1E-09
COQ7	coenzyme Q7, hydroxylase	protein_coding	chr16:19067614-1	4105	6.461544583	10.57737558	0.625511	1.33442	1E-09
SFRP2	secreted frizzled related protein 2	protein_coding	chr4:153780591-1	1996	0.518648936	2.402373431	2.10891	1.34454	1E-09
PGM3	phosphoglucomutase 3	protein_coding	chr6:83161150-83	7478	5.055266131	7.974094173	0.571499	1.34573	1E-09
ZRANB2	zinc finger RANBP2-type containing 2	protein_coding	chr1:71063291-71	4985	21.44159671	32.572416	0.517381	1.34782	1E-09
PTDSS1	phosphatidylserine synthase 1	protein_coding	chr8:96261715-96	6295	9.739393424	14.54120729	0.491386	1.37065	1E-09
ASB4	ankyrin repeat and SOCS box containing 4	protein_coding	chr7:95478444-95	4606	0.109973595	0.994497324	3.063669	1.37600	1E-09
SLC25A22	solute carrier family 25 member 22	protein_coding	chr11:790475-798	4595	2.258298482	4.200620994	0.805877	1.43098	1E-09
DZIP1	DAZ interacting zinc finger protein 1	protein_coding	chr13:95578202-9	8470	1.770369834	3.039574105	0.695311	1.44565	1E-09
MFN1	mitofusin 1	protein_coding	chr3:179347709-1	5945	9.61246698	15.06460741	0.563629	1.45099	1E-09
PLAGL1	PLAG1 like zinc finger 1	protein_coding	chr6:143940300-1	8733	3.106646	5.600757704	0.765195	1.47369	1E-09
ARNT2	aryl hydrocarbon receptor nuclear translo	protein_coding	chr15:80404350-8	7737	2.184635336	4.493104085	0.957873	1.48112	1E-09
ZNF91	zinc finger protein 91	protein_coding	chr19:23304991-2	10308	4.227304699	6.334475529	0.49764	1.51262	1E-09
FBXO17	F-box protein 17	protein_coding	chr19:38941401-3	4792	1.843159877	3.434556483	0.808014	1.52094	1E-09
GOLIM4	golgi integral membrane protein 4	protein_coding	chr3:168008677-1	4647	54.10523617	78.75655106	0.455426	1.52243	1E-09
TPD52	tumor protein D52	protein_coding	chr8:80034745-80	8197	3.936583802	7.31017885	0.809377	1.52146	1E-09
TOP1MT	DNA topoisomerase I mitochondrial	protein_coding	chr8:143304384-1	6441	0.366707179	1.059252697	1.43716	1.52518	1E-09
HINT3	histidine triad nucleotide binding protein	protein_coding	chr6:125956770-1	3325	14.87682665	25.25661161	0.677921	1.53733	1E-09
SLC39A11	solute carrier family 39 member 11	protein_coding	chr17:72645949-7	5583	0.458350463	1.300508476	1.41445	1.55573	1E-09
TUBB4A	tubulin beta 4A class IVa	protein_coding	chr19:6494319-65	3220	1.446723282	3.59972892	1.223461	1.55711	1E-09
AJM1	apical junction component 1 homolog	protein_coding	chr9:136844415-1	4387	2.84963926	6.19981591	1.036095	1.57287	1E-09
CCDC18-AS1	CCDC18 antisense RNA 1	lncRNA	chr1:93262186-93	6284	1.491698972	3.22012954	1.021913	1.58238	1E-09
GPR61	G protein-coupled receptor 61	protein_coding	chr1:109539872-1	5057	0.579192967	1.58352083	1.356821	1.58606	1E-09
RPL15	ribosomal protein L15	protein_coding	chr3:23916545-23	7222	54.96784391	88.54569667	0.602395	1.59032	1E-09
IVD	isovaleryl-CoA dehydrogenase	protein_coding	chr15:40405485-4	6857	21.02126629	31.51627778	0.498384	1.59500	1E-09
APH1B	aph-1 homolog B, gamma-secretase subur	protein_coding	chr15:63276018-6	5443	0.940279746	2.045760972	1.030715	1.60002	1E-09
MAGEE1	MAGE family member E1	protein_coding	chrX:76427724-76	3630	1.374268228	3.147561044	1.114481	1.60286	1E-09

HEIH	hepatocellular carcinoma up-regulated EZ	lncRNA	chr5:180826871-1	2205	6.056255069	10.58934588	0.717129	1.62502	1E-09
TWISTNB	TWIST neighbor	protein_coding	chr7:19695461-19	3948	6.104036573	10.27852227	0.664822	1.65409	1E-09
PNMA8A	PNMA family member 8A	protein_coding	chr19:46466491-4	3696	18.13744087	26.52106784	0.461366	1.65992	1E-09
GSKIP	GSK3B interacting protein	protein_coding	chr14:96363452-9	4567	4.727116711	7.787150707	0.632969	1.70418	1E-09
SEC61B	Sec61 translocon beta subunit	protein_coding	chr9:99222064-99	1209	14.93820774	27.74885708	0.803143	1.73494	1E-09
FAM189A2	family with sequence similarity 189 meml	protein_coding	chr9:69324567-69	9552	18.48857298	27.59228796	0.491517	1.74092	1E-09
CTNNBIP1	catenin beta interacting protein 1	protein_coding	chr1:9848276-991	3339	18.95191534	30.58232969	0.604299	1.75039	1E-09
NT5DC3	5'-nucleotidase domain containing 3	protein_coding	chr12:103770453-	8759	1.047763701	2.125230962	0.936369	1.76908	1E-09
ADRB1	adrenoceptor beta 1	protein_coding	chr10:114044056-	2853	2.283032753	5.300982384	1.130097	1.78888	1E-09
NME3	NME/NM23 nucleoside diphosphate kinas	protein_coding	chr16:1770286-17	1445	17.23068443	28.78645273	0.651488	1.79726	1E-09
RAVER2	ribonucleoprotein, PTB binding 2	protein_coding	chr1:64745095-64 6070		9.980850193	14.75928089	0.477746	1.80581	1E-09
TFAM	transcription factor A, mitochondrial	protein_coding	chr10:58385345-5	5282	6.364356366	10.65100115	0.657978	1.81673	1E-09
CAMSAP1	calmodulin regulated spectrin associated	protein_coding	chr9:135808487-1	8960	8.448669264	13.30479942	0.569976	1.90401	1E-09
MANBAL	mannosidase beta like	protein_coding	chr20:37289638-3	2045	11.38138582	18.08340585	0.5802	1.91643	1E-09
HSP90AA1	heat shock protein 90 alpha family class A	protein_coding	chr14:102080738-	5248	124.4554942	210.1288307	0.671195	1.93103	1E-09
GNPNAT1	glucosamine-phosphate N-acetyltransfera	protein_coding	chr14:52775193-5 4010		3.400153152	5.732393298	0.669566	1.95245	1E-09
SOWAHC	sosondowah ankyrin repeat domain famil	protein_coding	chr2:109614364-1	4627	2.162894915	4.150362685	0.851006	1.95148	1E-09
ATXN3	ataxin 3	protein_coding	chr14:92044496-9	9661	2.114069541	3.608504156	0.685076	1.98137	1E-09
NAB2	NGFI-A binding protein 2	protein_coding	chr12:57089043-5	2861	7.783373286	14.12280892	0.773856	1.98313	1E-09
AC010186.3	novel transcript	lncRNA	chr12:9658567-96	2508	0.702988464	2.367497271	1.673478	2.02634	2E-09
ARL3	ADP ribosylation factor like GTPase 3	protein_coding	chr10:102673731-	3834	7.819936094	12.77564879	0.62142	2.04451	2E-09
PPP3CA	protein phosphatase 3 catalytic subunit a	protein_coding	chr4:101023409-1	5213	4.233080246	6.961170628	0.629502	2.08439	2E-09
OGG1	8-oxoguanine DNA glycosylase	protein_coding	chr3:9749944-978	8733	4.148799421	6.104742825	0.470694	2.09638	2E-09
PPP1R3B	protein phosphatase 1 regulatory subunit	protein_coding	chr8:9136255-915	5738	26.28200143	39.01924377	0.484062	2.15653	2E-09
CYP4V2	cytochrome P450 family 4 subfamily V me	protein_coding	chr4:186191567-1	9767	1.324342729	2.584380149	0.885213	2.17273	2E-09
FXYD6	FXYD domain containing ion transport re	protein_coding	chr11:117836976-	4986	22.0490516	33.2921239	0.507675	2.18960	2E-09
ANO10	anoctamin 10	protein_coding	chr3:43354859-43	5287	1.080143157	2.169955422	0.919722	2.20286	2E-09
DCAF10	DDB1 and CUL4 associated factor 10	protein_coding	chr9:37800554-37	8291	4.810058068	7.838517421	0.616362	2.23755	2E-09
PEX11A	peroxisomal biogenesis factor 11 alpha	protein_coding	chr15:89677764-8	3171	0.889855279	2.369186017	1.320219	2.24035	2E-09
C11orf68	chromosome 11 open reading frame 68	protein_coding	chr11:65916810-6	1704	22.37694506	33.04416438	0.476086	2.24587	2E-09
OLFML2A	olfactomedin like 2A	protein_coding	chr9:124777133-1	6771	5.352399457	8.640090008	0.605015	2.32759	2E-09
ERGIC1	endoplasmic reticulum-golgi intermediat	protein_coding	chr5:172834251-1	14161	5.693644181	9.03916004	0.581843	2.34060	2E-09
FAM210B	family with sequence similarity 210 meml	protein_coding	chr20:56358974-5	3518	16.33031651	24.81239416	0.516977	2.35363	2E-09
PROB1	proline rich basic protein 1	protein_coding	chr5:139390592-1	5122	1.259946139	3.222958045	1.256359	2.36462	2E-09
NRIP1	nuclear receptor interacting protein 1	protein_coding	chr21:14961235-1	8497	4.035685018	7.081012139	0.725088	2.41645	2E-09
NEK7	NIMA related kinase 7	protein_coding	chr1:198156994-1	5454	31.34954798	47.47486474	0.513025	2.43767	2E-09
SLC25A29	solute carrier family 25 member 29	protein_coding	chr14:100291116-	7446	6.648212001	11.06310819	0.649599	2.44027	2E-09
HNRNPA1P48	heterogeneous nuclear ribonucleoprotei	protein_coding	chr16:51553436-5	1924	6.319833541	10.89767354	0.699751	2.46570	2E-09
FXR2	FMR1 autosomal homolog 2	protein_coding	chr17:7591230-76	4331	26.16876733	41.90297349	0.592855	2.47224	2E-09
PRKX	protein kinase X-linked	protein_coding	chrX:3604370-371	6452	2.527053779	4.212560036	0.648829	2.51433	2E-09
STEAP2	STEAP2 metalloreductase	protein_coding	chr7:90167590-90	8720	0.422180152	1.083786195	1.278155	2.53227	2E-09
STK35	serine/threonine kinase 35	protein_coding	chr20:2101611-21	7055	4.833590762	7.828650456	0.611124	2.53519	2E-09
EVC	EvC ciliary complex subunit 1	protein_coding	chr4:5711201-581	7144	1.281353082	3.428073509	1.346769	2.56743	2E-09
MXI1	MAX interactor 1, dimerization protein	protein_coding	chr10:110207605-	5907	8.529854939	13.13494806	0.537371	2.59199	2E-09
TENT4B	terminal nucleotidyltransferase 4B	protein_coding	chr16:50152918-5	8144	2.620176057	4.40429854	0.660426	2.60868	2E-09
SV2A	synaptic vesicle glycoprotein 2A	protein_coding	chr1:149903318-1	4786	1.133944366	2.593985363	1.112595	2.62195	2E-09
LIG3	DNA ligase 3	protein_coding	chr17:34980494-3	12919	2.445834295	3.918763543	0.595346	2.62845	2E-09
CEP170	centrosomal protein 170	protein_coding	chr1:243124428-2	10939	5.422942919	7.969035109	0.468841	2.68837	2E-09
GAS8	growth arrest specific 8	protein_coding	chr16:90019629-9	5615	1.242430702	2.400027619	0.867692	2.70677	2E-09
KLF16	Kruppel like factor 16	protein_coding	chr19:1852399-18	2966	4.457382567	7.879441039	0.736465	2.71153	2E-09
EFHC2	EF-hand domain containing 2	protein_coding	chrX:44147872-44	3329	0.987385666	2.217247709	1.074257	2.71565	2E-09
MTMR12	myotubularin related protein 12	protein_coding	chr5:32226994-32	5523	5.633242418	8.75764865	0.552591	2.72825	2E-09
GFPT1	glutamine--fructose-6-phosphate transam	protein_coding	chr2:69319769-69	8866	7.558342016	11.28050635	0.491233	2.74571	2E-09
ELF2	E74 like ETS transcription factor 2	protein_coding	chr4:139028112-1	8560	5.654621053	8.614583272	0.52111	2.75554	2E-09
FAM98C	family with sequence similarity 98 memb	protein_coding	chr19:38403135-3	4598	1.563501594	3.256341414	0.965323	2.75972	2E-09
ENAH	ENAH actin regulator	protein_coding	chr1:225486835-2	15044	11.48460402	16.64915877	0.449558	2.78287	2E-09
SPG21	SPG21 abhydrolase domain containing, m	protein_coding	chr15:64963022-6	3983	13.32425012	20.79099746	0.557045	2.79215	2E-09
CMBL	carboxymethylenebutenolidase homolog	protein_coding	chr5:10275875-10	4882	1.079837667	2.500097424	1.124856	2.81595	2E-09
SMIM15	small integral membrane protein 15	protein_coding	chr5:61157704-61	2888	6.411106995	10.4128934	0.613081	2.81954	2E-09
TIGAR	TP53 induced glycolysis regulatory phosp	protein_coding	chr12:4307763-43	9444	1.001460502	1.825136625	0.77582	2.82448	2E-09
ZNF532	zinc finger protein 532	protein_coding	chr18:58862600-5	11951	10.07158923	15.85869782	0.56912	2.84874	2E-09
SLC25A30	solute carrier family 25 member 30	protein_coding	chr13:45393316-4	6794	6.041938569	10.15804065	0.667334	2.85049	2E-09
UBE2R2	ubiquitin conjugating enzyme E2 R2	protein_coding	chr9:33817160-33	4477	18.85897968	27.76155244	0.47193	2.85931	2E-09
GCHFR	GTP cyclohydrolase I feedback regulator	protein_coding	chr15:40764068-4	1481	3.904445451	9.222590979	1.153598	2.86855	2E-09
CPPED1	calcineurin like phosphoesterase domain	protein_coding	chr16:12659799-1	6354	0.763246395	1.809340706	1.161682	2.96336	2E-09
AGK	acylglycerol kinase	protein_coding	chr7:141551278-1	10697	2.687437928	4.685366354	0.716194	2.96844	2E-09
NPTN	neuroplastin	protein_coding	chr15:73560014-7	4032	72.140309	101.869321	0.411522	3.03459	2E-09
XPC	XPC complex subunit, DNA damage recogi	protein_coding	chr3:14145147-14	4798	7.062064627	10.6849355	0.509882	3.03415	2E-09
BDH1	3-hydroxybutyrate dehydrogenase 1	protein_coding	chr3:197509783-1	11814	2.207151671	3.574537408	0.613035	3.06806	2E-09
SEC22B	SEC22 homolog B, vesicle trafficking prote	protein_coding	chr1:120150898-1	9034	6.797307278	10.13879957	0.490329	3.13794	2E-09
TP53TG1	TP53 target 1	lncRNA	chr7:87322943-87	5871	0.599251311	1.618475711	1.347682	3.14786	2E-09
SSH1	slingshot protein phosphatase 1	protein_coding	chr12:108778191-	14538	8.60509185	15.14601452	0.733004	3.18323	2E-09
SH3RF3	SH3 domain containing ring finger 3	protein_coding	chr2:109129348-1	5803	0.796145792	1.88074524	1.164488	3.20380	2E-09
PMM1	phosphomannomutase 1	protein_coding	chr22:41576900-4	5057	4.603764089	8.30281232	0.767466	3.21517	2E-09
PHAX	phosphorylated adaptor for RNA export	protein_coding	chr5:126600925-1	3662	7.105133276	11.14578397	0.562767	3.22084	2E-09
SLC25A6	solute carrier family 25 member 6	protein_coding	chrX:1386152-139	1553	359.7690221	532.4553764	0.479521	3.25540	2E-09
SLC41A3	solute carrier family 41 member 3	protein_coding	chr3:126006355-1	6592	3.869242491	6.113093751	0.573946	3.28560	2E-09
SVIP	small VCP interacting protein	protein_coding	chr11:22813799-2	5201	2.244163967	4.126290768	0.787187	3.28887	2E-09
GOLM1	golgi membrane protein 1	protein_coding	chr9:86026146-86	4233	6.262885216	10.38060214	0.640549	3.32324	2E-09
FSD1	fibronectin type III and SPRY domain cont	protein_coding	chr19:4304598-43	3113	1.131533117	2.888769286	1.265183	3.32824	2E-09
WWP1	WW domain containing E3 ubiquitin prot	protein_coding	chr8:86342738-86	6336	16.65131248	23.7880591	0.428033	3.33517	2E-09
NTN4	netrin 4	protein_coding	chr12:95657807-9	4166	0.420900882	1.330926205	1.577443	3.34826	2E-09
MVB12B	multivesicular body subunit 12B	protein_coding	chr9:126326829-1	7904	2.918176922	4.571099097	0.56041	3.35317	2E-09
AC107884.1	novel transcript, antisense to OLFML1	lncRNA	chr11:7418826-75	11336	0.49619316	1.008936763	0.936156	3.36973	2E-09
HSPA1A	heat shock protein family A (Hsp70) meml	protein_coding	chr6:31815464-31	2483	11.04720011	18.50296498	0.656207	3.38157	2E-09
CAPZA1	capping actin protein of muscle Z-line sub	protein_coding	chr1:112619805-1	3495	24.56374738	39.73345751	0.609248	3.40339	2E-09
PLBD2	phospholipase B domain containing 2	protein_coding	chr12:113358566-	5264	6.163171053	10.85977653	0.732059	3.42749	2E-09
UBQLN1	ubiquilin 1	protein_coding	chr9:83659968-83	6867	21.48624486	29.9932401	0.394938	3.44550	2E-09
ZNRF3	zinc and ring finger 3	protein_coding	chr22:28883592-2	6968	4.645336293	7.107460553	0.527777	3.47056	2E-09
ZNF33A	zinc finger protein 33A	protein_coding	chr10:38010650-3	7523	5.480252568	8.135693314	0.483754	3.55795	2E-09
LONRF2	LON peptidase N-terminal domain and rin	protein_coding	chr2:100273291-1	13933	1.352697367	2.630190488	0.875296	3.60105	2E-09
JARID2	jumonji and AT-rich interaction domain c	protein_coding	chr6:15246069-15	6244	4.700378322	7.421215897	0.572383	3.65571	3E-09
TMEM33	transmembrane protein 33	protein_coding	chr4:41935129-41	8462	3.649412282	5.639966509	0.543246	3.66154	3E-09
FZD6	frizzled class receptor 6	protein_coding	chr8:103298433-1	4065	7.776975378	12.91620933	0.644823	3.69207	3E-09
MAP1A	microtubule associated protein 1A	protein_coding	chr15:43510958-4	10646	8.759564944	14.38940479	0.631254	3.73244	3E-09
PLS3	plastin 3	protein_coding	chrX:115561174-1	4099	24.76434865	40.07530761	0.609842	3.76304	3E-09
ZNF846	zinc finger protein 846	protein_coding	chr19:9751993-97	3912	1.322717403	2.707967634	0.94468	3.81641	3E-09
SH3BGRL2	SH3 domain binding glutamate rich prote	protein_coding	chr6:79631329-79	4603	4.238986334	7.072915955	0.65048	3.84056	3E-09
TMED10	transmembrane p24 trafficking protein 1(	protein_coding	chr14:75131469-7	4956	36.18806147	51.96094393	0.435567	3.93600	3E-09
JADE3	jade family PHD finger 3	protein_coding	chrX:46912276-47	5149	2.318411261	4.011336401	0.70484	3.97500	3E-09
PBX2	PBX homeobox 2	protein_coding	chr6:32184733-32	4368	23.38870682	37.91546401	0.612345	4.06268	3E-09
NIPAL1	NIPA like domain containing 1	protein_coding	chr4:47914142-48	5983	0.941321964	2.484713314	1.323691	4.08121	3E-09
RMI1	RecQ mediated genome instability 1	protein_coding	chr9:83980711-84	3507	3.13896657	5.470101806	0.711677	4.07946	3E-09
EML1	EMAP like 1	protein_coding	chr14:99737693-9	8935	3.203675069	5.048523588	0.570508	4.12374	3E-09
PLEKHA5	pleckstrin homology domain containing A	protein_coding	chr12:19129752-1	12558	7.413327058	10.56071045	0.424162	4.13393	3E-09
IAH1	isoamyl acetate hydrolyzing esterase 1 (pu	protein_coding	chr2:9473658-949	5908	2.240291182	3.894040396	0.708402	4.14022	3E-09
TMX3	thioredoxin related transmembrane prote	protein_coding	chr18:68673688-6	7447	9.221978143	13.5460444	0.46777	4.15429	3E-09
FNBP4	formin binding protein 4	protein_coding	chr11:47716517-4	7596	10.17847732	14.79843055	0.453865	4.23817	3E-09
TCP11L1	t-complex 11 like 1	protein_coding	chr11:33039417-3	5370	1.124366084	2.176783747	0.868355	4.30257	3E-09
AHR	aryl hydrocarbon receptor	protein_coding	chr7:16916359-17	8051	0.634100965	1.234778514	0.877617	4.33005	3E-09
CUL4A	cullin 4A	protein_coding	chr13:113208193-	7813	26.32153166	38.05151489	0.445837	4.33547	3E-09

ASMTL	acetylserotonin O-methyltransferase like	protein_coding	chrX:1403139-145	3506	8.061172614	12.45376855	0.541322	4.36467	3E-09
KNDC1	kinase non-catalytic C-lobe domain conta	protein_coding	chr10:133160219-	8213	1.115740473	2.493883753	1.075942	4.41527	3E-09
RIF1	replication timing regulatory factor 1	protein_coding	chr2:151409883-1	18166	2.17862759	3.20918008	0.472076	4.44428	3E-09
SLC41A2	solute carrier family 41 member 2	protein_coding	chr12:104802553-	5151	0.359098717	1.132377523	1.574399	4.44823	3E-09
SHKBP1	SH3KBP1 binding protein 1	protein_coding	chr19:40576853-4	3934	4.814981697	7.929869585	0.632921	4.50586	3E-09
RIPOR2	RHO family interacting cell polarization re	protein_coding	chr6:24804282-25	9435	1.465326138	3.261112682	1.074307	4.51429	3E-09
ADSS	adenylosuccinate synthase	protein_coding	chr1:244408494-2	3441	4.685860248	8.432395208	0.75913	4.52283	3E-09
RALGPS1	Ral GEF with PH domain and SH3 binding	protein_coding	chr9:126914774-1	10507	3.714825628	6.08622283	0.628055	4.53336	3E-09
DECR2	2,4-dienoyl-CoA reductase 2	protein_coding	chr16:401858-412	4014	3.936474372	6.938303001	0.727728	4.53739	3E-09
NUP50	nucleoporin 50	protein_coding	chr22:45163925-4	8236	2.846838399	4.542092408	0.588429	4.57695	3E-09
TMEM143	transmembrane protein 143	protein_coding	chr19:48332356-4	3523	5.77422048	9.633947459	0.657315	4.59816	3E-09
DOCK1	dedicator of cytokinesis 1	protein_coding	chr10:126905409-	8142	3.728113181	5.991093938	0.600644	4.61499	3E-09
SLC9A3R2	SLC9A3 regulator 2	protein_coding	chr16:2025356-20	3516	9.067332602	14.35856868	0.575443	4.62426	3E-09
LINC01351	long intergenic non-protein coding RNA 1	lncRNA	chr1:190478551-1	607	2.590893007	7.440972852	1.42699	4.64354	3E-09
CDC42	cell division cycle 42	protein_coding	chr1:22025511-22	16278	10.83419696	15.8790593	0.465823	4.67264	3E-09
ANOS1	anosmin 1	protein_coding	chrX:8528874-873	7082	4.602252201	7.11668479	0.541385	4.68333	3E-09
ARHGDIA	Rho GDP dissociation inhibitor alpha	protein_coding	chr17:81867721-8	2875	44.98159676	67.85792713	0.506698	4.68556	3E-09
AC018464.1	novel transcript	lncRNA	chr7:112953282-1	3826	4.376658564	9.193650633	0.983648	4.69733	3E-09
MED22	mediator complex subunit 22	protein_coding	chr9:133338323-1	7224	5.645034089	8.45243011	0.495704	4.72492	3E-09
TCAP	titin-cap	protein_coding	chr17:39664187-3	2123	185.4865874	280.9062659	0.513106	4.84674	3E-09
MIR100HG	mir-100-let-7a-2-mir-125b-1 cluster host	lncRNA	chr11:122028325-	28649	1.582977705	2.543313865	0.600473	4.95228	3E-09
SLC8B1	solute carrier family 8 member B1	protein_coding	chr12:113298759-	6768	0.559110679	1.44007925	1.27733	4.94997	3E-09
XIAP	X-linked inhibitor of apoptosis	protein_coding	chrX:123859724-1	9025	5.593908905	8.725864487	0.555525	5.06610	3E-09
FANCF	FA complementation group F	protein_coding	chr11:22622890-2	2952	5.711657343	9.112534434	0.588309	5.10894	3E-09
SPINT1-AS1	SPINT1 antisense RNA 1	lncRNA	chr15:40835808-4	1239	0.459238469	2.120879891	2.102561	5.12869	3E-09
SENP6	SUMO specific peptidase 6	protein_coding	chr6:75601509-75	11417	6.401048178	9.317915398	0.455632	5.15152	3E-09
CFL2	cofilin 2	protein_coding	chr14:34706769-3	7179	42.06039378	64.96611709	0.542484	5.20068	3E-09
B4GALT2	beta-1,4-galactosyltransferase 2	protein_coding	chr1:43978943-43	3772	11.60417778	16.83394884	0.449755	5.24154	3E-09
TAGLN	transgelin	protein_coding	chr11:117199321-	3917	22.15493783	36.25350425	0.624273	5.24012	3E-09
RPS6KA3	ribosomal protein S6 kinase A3	protein_coding	chrX:20149911-20	9801	4.584511497	6.622181315	0.443449	5.26701	3E-09
JUP	junction plakoglobin	protein_coding	chr17:41754604-4	4942	83.25643266	125.6540224	0.507907	5.34712	3E-09
NEK10	NIMA related kinase 10	protein_coding	chr3:27110085-27	9582	1.437935621	2.688215403	0.819952	5.35410	3E-09
SERPINH1	serpin family H member 1	protein_coding	chr11:75562056-7	6281	39.1123497	55.33635218	0.414595	5.40608	4E-09
ASB10	ankyrin repeat and SOCS box containing 1	protein_coding	chr7:151175698-1	2375	0.743636561	2.593875353	1.716233	5.41314	4E-09
B3GNT2	UDP-GlcNAc:betaGal beta-1,3-N-acetylglu	protein_coding	chr2:62196115-62	2859	15.53469568	24.79764557	0.586047	5.45892	4E-09
MTF2	metal response element binding transcrip	protein_coding	chr1:93079235-93	5907	3.336914295	5.501074651	0.635021	5.48862	4E-09
C8orf82	chromosome 8 open reading frame 82	protein_coding	chr8:144525733-1	3001	12.02999695	19.81515285	0.634695	5.49254	4E-09
AC105118.1	novel transcript	lncRNA	chr8:143412749-1	544	0.321358584	4.211741178	3.562082	5.51125	4E-09
DEGS1	delta 4-desaturase, sphingolipid 1	protein_coding	chr1:224175756-2	2446	23.50154401	36.3886227	0.543844	5.51125	4E-09
NDN	necdin, MAGE family member	protein_coding	chr15:23685679-2	1627	44.38056173	64.28706301	0.448632	5.56657	4E-09
GATD1	glutamine amidotransferase like class 1 dc	protein_coding	chr11:767220-777	5510	11.89476944	17.86362363	0.501527	5.62238	4E-09
PPP2R1B	protein phosphatase 2 scaffold subunit Ab	protein_coding	chr11:111726908-	6720	0.673846614	1.599735043	1.169984	5.66185	4E-09
SMURF2	SMAD specific E3 ubiquitin protein ligase	protein_coding	chr17:64542282-6	7213	5.080325167	7.901205147	0.550754	5.66956	4E-09
FBXO21	F-box protein 21	protein_coding	chr12:117141988-	6949	7.69429524	11.14876588	0.448276	5.68376	4E-09
SPOP	speckle type BTB/POZ protein	protein_coding	chr17:49598884-4	6506	7.926240787	11.92965958	0.505067	5.68077	4E-09
RNF115	ring finger protein 115	protein_coding	chr1:145738868-1	11059	13.23470348	19.22743228	0.453018	5.72981	4E-09
RIMKLB	ribosomal modification protein rimK like	protein_coding	chr12:8681600-87	8597	8.172992304	12.28759435	0.503317	5.73589	4E-09
BCAS4	breast carcinoma amplified sequence 4	protein_coding	chr20:50794894-5	7583	1.423501546	2.459247311	0.700052	5.74921	4E-09
N4BP2L2	NEDD4 binding protein 2 like 2	protein_coding	chr13:32432417-3	16960	4.963813321	7.024106791	0.414472	5.80624	4E-09
TBC1D32	TBC1 domain family member 32	protein_coding	chr6:121079494-1	6912	0.595088965	1.288857201	1.027024	5.84721	4E-09
SOX12	SRY-box 12	protein_coding	chr20:325595-330	4630	30.20991945	44.08773924	0.459591	5.85615	4E-09
CIC	capicua transcriptional repressor	protein_coding	chr19:42268537-4	9494	12.61287301	21.17557364	0.66284	5.96248	4E-09
SMUG1	single-strand-selective monofunctional ur	protein_coding	chr12:54121277-5	6027	2.159459543	3.580647232	0.641733	5.99970	4E-09
PRKAG1	protein kinase AMP-activated non-catalyt	protein_coding	chr12:49002274-4	4184	10.16867802	15.61408686	0.531774	6.16945	4E-09
RTL8C	retrotransposon Gag like 8C	protein_coding	chrX:135032366-1	1181	278.6811298	398.1710597	0.42864	6.19729	4E-09
NEDD4	neural precursor cell expressed, developm	protein_coding	chr15:55826922-5	9122	0.676914395	1.378371587	0.933814	6.24542	4E-09
SKI	SKI proto-oncogene	protein_coding	chr1:2228319-231	6550	9.552406693	13.9213936	0.457569	6.42642	4E-09
MTX3	metaxin 3	protein_coding	chr5:79976731-79	7919	5.557771542	8.005888105	0.439679	6.58629	4E-09
MELTF-AS1	MELTF antisense RNA 1	lncRNA	chr3:196999460-1	1139	2.125050097	6.48823818	1.547779	6.59200	4E-09
ADCK2	aarF domain containing kinase 2	protein_coding	chr7:140672945-1	3110	4.336311512	7.229203734	0.651783	6.60916	4E-09
DHFR2	dihydrofolate reductase 2	protein_coding	chr3:94047836-94	4659	0.786486534	1.704866405	1.03342	6.80042	4E-09
RASA1	RAS p21 protein activator 1	protein_coding	chr5:87267888-87	5232	5.966750824	9.19783886	0.539627	6.87426	4E-09
PRPS2	phosphoribosyl pyrophosphate synthetas	protein_coding	chrX:12791355-12	2758	0.701344658	1.817038628	1.281923	6.93333	4E-09
HDAC1	histone deacetylase 1	protein_coding	chr1:32292083-32	3966	12.93430496	20.78215188	0.597073	6.98272	4E-09
SQLE	squalene epoxidase	protein_coding	chr8:124998497-1	4305	10.20606838	16.47387205	0.606197	7.02001	4E-09
CASP6	caspase 6	protein_coding	chr4:109688622-1	1943	3.688532845	7.598728996	0.954836	7.03416	4E-09
NUDT2	nudix hydrolase 2	protein_coding	chr9:34329506-34	1073	8.302852938	15.70862502	0.83904	7.06382	4E-09
AL391684.1	novel transcript	lncRNA	chr10:99430919-9	8535	1.175149365	2.191924165	0.814612	7.10531	4E-09
HIST3H2A	histone cluster 3 H2A	protein_coding	chr1:228456979-2	895	12.83072922	21.32784815	0.649986	7.14016	4E-09
ABR	ABR activator of RhoGEF and GTPase	protein_coding	chr17:1003518-12	12426	12.07191078	17.86295502	0.479794	7.23736	5E-09
MAPK8IP3	mitogen-activated protein kinase 8 intera	protein_coding	chr16:1706183-17	10285	17.42960838	28.11844542	0.60604	7.25603	5E-09
SPIN1	spindlin 1	protein_coding	chr9:88388430-88	4903	22.76806572	32.15230648	0.412069	7.27972	5E-09
VSTM2L	V-set and transmembrane domain contair	protein_coding	chr20:37903111-3	1952	36.42174456	58.24711373	0.590653	7.36530	5E-09
AC117464.1	novel transcript	lncRNA	chr3:83953639-84	3783	0.153155968	0.941078477	2.556957	7.56209	5E-09
CELF5	CUGBP Elav-like family member 5	protein_coding	chr19:3224661-32	6066	0.284610073	0.926762723	1.619906	7.56682	5E-09
ERCC1	ERCC excision repair 1, endonuclease non	protein_coding	chr19:45407333-4	5576	12.76751455	19.59210216	0.531992	7.57843	5E-09
CAPN5	calpain 5	protein_coding	chr11:77066961-7	6137	0.633989363	1.339350561	0.990203	7.69153	5E-09
PABPC5	poly(A) binding protein cytoplasmic 5	protein_coding	chrX:91434595-91	3450	1.725727574	3.649078846	0.99346	7.68710	5E-09
GAR1	GAR1 ribonucleoprotein	protein_coding	chr4:109815510-1	1964	4.146025046	7.408649595	0.751286	7.78768	5E-09
DLC1	DLC1 Rho GTPase activating protein	protein_coding	chr8:13083361-13	12794	14.82791013	21.16824589	0.427546	7.87170	5E-09
ARL4A	ADP ribosylation factor like GTPase 4A	protein_coding	chr7:12686856-12	3585	1.549543089	3.170841538	0.946433	8.11233	5E-09
CAD	carbamoyl-phosphate synthetase 2, aspar	protein_coding	chr2:27217369-27	8443	5.723742464	8.947624458	0.561399	8.15421	5E-09
BAIAP2L1	BAI1 associated protein 2 like 1	protein_coding	chr7:98291650-98	4821	0.94320513	1.998487838	1.005295	8.39966	5E-09
ACTB	actin beta	protein_coding	chr7:5527148-556	3413	99.7992832	164.1438457	0.633349	8.44204	5E-09
REEP2	receptor accessory protein 2	protein_coding	chr5:138439057-1	3244	15.15532175	24.59628879	0.61291	8.47184	5E-09
WBP4	WW domain binding protein 4	protein_coding	chr13:41061509-4	2388	9.280792492	15.40012947	0.645933	8.49276	5E-09
FXR1	FMR1 autosomal homolog 1	protein_coding	chr3:180868141-1	11874	19.82988528	27.59961931	0.390688	8.50249	5E-09
XRN2	5'-3' exoribonuclease 2	protein_coding	chr20:21303331-2	3408	18.38647657	26.00099169	0.413783	8.66236	5E-09
THNSL1	threonine synthase like 1	protein_coding	chr10:25016612-2	3834	1.999726265	3.780823132	0.829266	8.69248	5E-09
KIAA0930	KIAA0930	protein_coding	chr22:45190338-4	10019	2.180764661	3.246370778	0.486782	8.75935	5E-09
AC093866.1	novel transcript	lncRNA	chr4:89410960-89	6974	1.584153444	2.690263434	0.676132	8.86219	5E-09
DKK1	dickkopf WNT signaling pathway inhibitor	protein_coding	chr10:52314281-5	2221	0.285104032	1.642010713	2.486416	9.04814	5E-09
PCCB	propionyl-CoA carboxylase subunit beta	protein_coding	chr3:136250340-1	7498	13.30376702	18.64149311	0.400423	9.16162	6E-09
AC008771.1	novel transcript	lncRNA	chr5:80411231-80	8625	1.618157822	2.774370288	0.689814	9.22018	6E-09
MYO6	myosin VI	protein_coding	chr6:75749192-75	9322	2.046770472	3.120817105	0.521186	9.24040	6E-09
PLPPR2	phospholipid phosphatase related 2	protein_coding	chr19:11355386-1	3273	15.8100388	23.62075935	0.491996	9.24038	6E-09
FAM214B	family with sequence similarity 214 meml	protein_coding	chr9:35104112-35	6409	5.787449759	8.66012561	0.495417	9.26688	6E-09
ARMCX2	armadillo repeat containing X-linked 2	protein_coding	chrX:101655281-1	3548	23.4592363	33.51342234	0.427598	9.32353	6E-09
AC022007.1	novel transcript	lncRNA	chr3:10004048-10	4823	1.524488778	2.763105758	0.770443	9.35677	6E-09
CASC4	cancer susceptibility 4	protein_coding	chr15:44288719-4	7105	8.627523099	12.09077015	0.400832	9.38365	6E-09
C17orf58	chromosome 17 open reading frame 58	protein_coding	chr17:67991101-6	2396	0.993051029	2.549527452	1.277986	9.42737	6E-09
RAB40B	RAB40B, member RAS oncogene family	protein_coding	chr17:82654973-8	6164	4.045067461	6.146714697	0.516585	9.46115	6E-09
CDC23	cell division cycle 23	protein_coding	chr5:138187650-1	4616	8.491449033	12.32689827	0.450198	9.55094	6E-09
PHIP	pleckstrin homology domain interacting p	protein_coding	chr6:78934419-79	14718	5.580552839	7.786332742	0.394277	9.54874	6E-09
AC009061.2	novel transcript, antisense to AGRP	lncRNA	chr16:67481314-6	4828	0.894758517	1.888936914	0.982301	9.62834	6E-09
PEX10	peroxisomal biogenesis factor 10	protein_coding	chr1:2403964-241	3789	6.272316945	9.403080137	0.499026	9.90528	6E-09
GFY	golgi associated olfactory signaling regula	protein_coding	chr19:49423749-4	1876	0.876766639	2.928158153	1.640635	9.96797	6E-09
TOB2	transducer of ERBB2, 2	protein_coding	chr22:41433494-4	4341	12.46372159	17.75606416	0.423812	9.98886	6E-09
CACNA1G	calcium voltage-gated channel subunit alp	protein_coding	chr17:50560715-5	10062	15.67095159	28.30756593	0.771969	1E-09	6E-09
FAH	fumarylacetoacetate hydrolase	protein_coding	chr15:80152490-8	7203	2.153022126	3.601972481	0.66154	1E-09	6E-09
ACTN3	actinin alpha 3 (gene/pseudogene)	protein_coding	chr11:66546395-6	3460	1.17537321	2.478548478	0.993711	1E-09	6E-09

KCNJ8	potassium voltage-gated channel subfami	protein_coding	chr12:21764955-2	2511	7.016927362	11.34104804	0.608077	1E-09	6E-09
ARRDC1	arrestin domain containing 1	protein_coding	chr9:137605685-1	3790	10.55935097	17.46999009	0.639318	1E-09	6E-09
LAD1	ladinin 1	protein_coding	chr1:201380838-2	3401	0.724170179	1.904106252	1.300549	1E-09	6E-09
SLC9B2	solute carrier family 9 member B2	protein_coding	chr4:103019868-1	5374	3.738393425	6.193296112	0.638455	1E-09	6E-09
TMEM56	transmembrane protein 56	protein_coding	chr1:95117355-95	7385	19.0705831	27.75512791	0.455281	1E-09	6E-09
ZNF514	zinc finger protein 514	protein_coding	chr2:95145000-95	9384	1.742719139	2.888379605	0.642141	1E-09	6E-09
THUMPD3-AS1	THUMPD3 antisense RNA 1	lncRNA	chr3:9349689-939	9533	2.905797052	4.473072213	0.536417	1E-09	6E-09
PLCG2	phospholipase C gamma 2	protein_coding	chr16:81739097-8	13375	2.828046589	4.291614619	0.515683	1E-09	6E-09
PIR	pirin	protein_coding	chrX:15384799-15	2512	4.863362071	8.865788037	0.779237	1E-09	6E-09
PARD6G	par-6 family cell polarity regulator gamma	protein_coding	chr18:80157232-8	4192	2.424775983	4.122385199	0.678719	1E-09	6E-09
HOMER1	homer scaffold protein 1	protein_coding	chr5:79372636-79	6154	18.75700671	26.60270996	0.417249	1E-09	7E-09
HSPB3	heat shock protein family B (small) membe	protein_coding	chr5:54455699-54	679	190.080024	282.3162104	0.484486	1E-09	7E-09
MT1F	metallothionein 1F	protein_coding	chr16:56657731-5	1852	0.292208924	1.300086367	2.060861	1E-09	7E-09
PLCXD3	phosphatidylinositol specific phospholipa	protein_coding	chr5:41306952-41	7733	13.18798015	19.94877681	0.512161	1E-09	7E-09
ZNF622	zinc finger protein 622	protein_coding	chr5:16451519-16	1707	39.47153897	61.0673702	0.543349	1E-09	7E-09
AKR7A2	aldo-keto reductase family 7 member A2	protein_coding	chr1:19303965-19	1781	20.47387161	30.18691614	0.473453	1E-09	7E-09
HIST1H2BD	histone cluster 1 H2B family member d	protein_coding	chr6:26158146-26	871	4.898344367	12.74033785	1.281561	1E-09	7E-09
TFE3	transcription factor binding to IGHM enha	protein_coding	chrX:49028726-49	4207	12.05954522	17.5811897	0.45718	1E-09	7E-09
DIAPH2	diaphanous related formin 2	protein_coding	chrX:96684663-97	9550	0.918488367	1.715474571	0.814161	1E-09	7E-09
FGGY	FGGY carbohydrate kinase domain contai	protein_coding	chr1:59296638-59	12174	0.573090816	1.080428084	0.823504	1E-09	7E-09
APBA2	amyloid beta precursor protein binding fa	protein_coding	chr15:28884483-2	6869	0.899173244	1.825715312	0.930807	1E-09	7E-09
DPP3	dipeptidyl peptidase 3	protein_coding	chr11:66480013-6	3977	4.521821393	7.149065436	0.574603	1E-09	7E-09
RNPEP	arginyl aminopeptidase	protein_coding	chr1:201982372-2	3455	10.2201134	15.96959705	0.557364	1E-09	7E-09
NRSN2	neurensin 2	protein_coding	chr20:346782-359	2933	20.34152765	29.80147817	0.465255	1E-09	7E-09
HPS3	HPS3 biogenesis of lysosomal organelles c	protein_coding	chr3:149129584-1	6256	1.264096965	2.315308699	0.786223	1E-09	7E-09
SIRPA	signal regulatory protein alpha	protein_coding	chr20:1894167-19	5068	46.24387222	68.66767108	0.485076	1E-09	7E-09
MCRIP1	MAPK regulated corepressor interacting p	protein_coding	chr17:81822361-8	3726	18.41851372	27.75461635	0.506299	1E-09	7E-09
PCTP	phosphatidylcholine transfer protein	protein_coding	chr17:55750979-5	5880	2.660384236	4.36717655	0.628244	1E-09	7E-09
CCDC113	coiled-coil domain containing 113	protein_coding	chr16:58231157-5	5927	1.016810754	1.950212257	0.848978	1E-09	7E-09
RTL8B	retrotransposon Gag like 8B	protein_coding	chrX:135020504-1	2026	34.66077969	52.47906004	0.511025	1E-09	7E-09
GSTM2	glutathione S-transferase mu 2	protein_coding	chr1:109668022-1	9116	0.736004488	1.540175918	0.979173	1E-09	7E-09
ARPC1A	actin related protein 2/3 complex subunit	protein_coding	chr7:99325898-99	2065	12.67183409	21.80737167	0.695499	1E-09	7E-09
KCNC3	potassium voltage-gated channel subfami	protein_coding	chr19:50311937-5	6277	3.362811004	5.918997712	0.734711	1E-09	7E-09
SAMD11	sterile alpha motif domain containing 11	protein_coding	chr1:923928-9445	4173	1.28533961	3.134455439	1.197306	1E-09	7E-09
TBKBP1	TBK1 binding protein 1	protein_coding	chr17:47694081-4	4280	11.50213929	17.79547242	0.542559	1E-09	7E-09
ST13	ST13 Hsp70 interacting protein	protein_coding	chr22:40824535-4	3764	51.65836358	78.11404425	0.51182	1E-09	7E-09
CELF2	CUGBP Elav-like family member 2	protein_coding	chr10:10798397-1	10978	14.88631498	21.6770199	0.456175	1E-09	7E-09
RCBTB1	RCC1 and BTB domain containing protein	protein_coding	chr13:49531946-4	4509	10.19626465	15.78710847	0.545986	1E-09	7E-09
B4GALT5	beta-1,4-galactosyltransferase 5	protein_coding	chr20:49632945-4	4722	12.70849785	17.89464575	0.406874	1E-09	7E-09
U91328.1	novel transcript	lncRNA	chr6:25992641-26	4365	0.406956431	1.257331203	1.543633	1E-09	8E-09
MICU1	mitochondrial calcium uptake 1	protein_coding	chr10:72367327-7	3852	11.68745046	17.95219021	0.534894	1E-09	8E-09
DPP9	dipeptidyl peptidase 9	protein_coding	chr19:4675224-47	8324	6.97202978	9.99156465	0.432923	1E-09	8E-09
LINC01531	long intergenic non-protein coding RNA 1	lncRNA	chr19:35399511-3	4107	1.874387665	3.831905357	0.94281	1E-09	8E-09
MFSD13A	major facilitator superfamily domain con	protein_coding	chr10:102461395-	3526	0.475727903	1.234331758	1.288983	1E-09	8E-09
RTCB	RNA 2',3'-cyclic phosphate and 5'-OH ligas	protein_coding	chr22:32387582-3	2941	16.11759487	25.19615939	0.558365	1E-09	8E-09
DBNDD1	dysbindin domain containing 1	protein_coding	chr16:90004865-9	4791	1.207352799	2.480503738	0.954621	1E-09	8E-09
FBXL17	F-box and leucine rich repeat protein 17	protein_coding	chr5:107859035-1	8125	5.403192409	8.248075901	0.52461	1E-09	8E-09
TBX2	T-box 2	protein_coding	chr17:61399843-6	6082	5.042309098	7.583552951	0.50141	1E-09	8E-09
TMEM123	transmembrane protein 123	protein_coding	chr11:102396332-	4442	18.96912052	27.23423138	0.4363	1E-09	8E-09
RNF168	ring finger protein 168	protein_coding	chr3:196468783-1	5347	4.042311187	6.001453856	0.483316	1E-09	8E-09
SAC3D1	SAC3 domain containing 1	protein_coding	chr11:65040901-6	1772	5.848243683	10.64772905	0.776313	1E-09	8E-09
KANSL3	KAT8 regulatory NSL complex subunit 3	protein_coding	chr2:96593170-96	9022	8.120684637	11.35193737	0.396907	1E-09	8E-09
TMEM168	transmembrane protein 168	protein_coding	chr7:112762382-1	7995	4.129480343	6.323405056	0.527674	1E-09	8E-09
ZDHHC18	zinc finger DHHC-type containing 18	protein_coding	chr1:26826688-26	5460	3.692143844	6.011447256	0.614597	1E-09	8E-09
FUCA1	alpha-L-fucosidase 1	protein_coding	chr1:23845077-23	2047	6.503131372	10.78428652	0.643242	1E-09	8E-09
NFIC	nuclear factor I C	protein_coding	chr19:3314403-34	9801	15.83357425	22.6978776	0.433104	1E-09	8E-09
ELOVL1	ELOVL fatty acid elongase 1	protein_coding	chr1:43363398-43	2506	6.09831222	9.73523829	0.589211	1E-09	8E-09
ARSD	arylsulfatase D	protein_coding	chrX:2903972-292	7026	5.158390524	7.587649355	0.4714	1E-09	8E-09
TNK2	tyrosine kinase non receptor 2	protein_coding	chr3:195863365-1	5153	25.38273226	38.73989472	0.524902	1E-09	8E-09
CROT	carnitine O-octanoyltransferase	protein_coding	chr7:87345681-87	4979	2.779004255	4.93206747	0.740412	2E-09	9E-09
CRELD2	cysteine rich with EGF like domains 2	protein_coding	chr22:49918167-4	5165	4.526355149	7.193225729	0.582565	2E-09	9E-09
OGT	O-linked N-acetylglucosamine (GlcNAc) tr	protein_coding	chrX:71533104-71	9786	12.64030659	19.38331825	0.532469	2E-09	9E-09
CECR2	CECR2 histone acetyl-lysine reader	protein_coding	chr22:17359949-1	10759	2.048238126	3.75838691	0.795344	2E-09	9E-09
ALG13	ALG13 UDP-N-acetylglucosaminyltransfer	protein_coding	chrX:111665811-1	8188	6.780112704	9.623219108	0.418751	2E-09	9E-09
SPATA24	spermatogenesis associated 24	protein_coding	chr5:139396563-1	1204	3.085792623	7.229351952	1.132261	2E-09	9E-09
IRS2	insulin receptor substrate 2	protein_coding	chr13:109752695-	8156	2.369713753	4.25844141	0.767369	2E-09	9E-09
USP24	ubiquitin specific peptidase 24	protein_coding	chr1:55066359-55	12023	8.301775049	12.43291686	0.497974	2E-09	9E-09
POFUT2	protein O-fucosyltransferase 2	protein_coding	chr21:45263928-4	5862	4.929825904	7.660155794	0.548754	2E-09	9E-09
PTMA	prothymosin alpha	protein_coding	chr2:231706895-2	3378	42.6004551	71.96444603	0.673351	2E-09	9E-09
SNX7	sorting nexin 7	protein_coding	chr1:98661701-98	2067	8.335447432	14.11603805	0.672582	2E-09	9E-09
DDN	dendrin	protein_coding	chr12:48995149-4	3815	1.41680291	3.05517948	1.023876	2E-09	9E-09
SGK3	serum/glucocorticoid regulated kinase fa	protein_coding	chr8:66667552-66	8316	1.20943675	2.040942522	0.670123	2E-09	9E-09
DAZAP1	DAZ associated protein 1	protein_coding	chr19:1407569-14	9201	12.88273875	17.45081851	0.351597	2E-09	9E-09
GPR37	G protein-coupled receptor 37	protein_coding	chr7:124745997-1	3021	0.338693168	1.349242497	1.942949	2E-09	9E-09
INTS3	integrator complex subunit 3	protein_coding	chr1:153728050-1	10467	7.737032494	12.42592177	0.59908	2E-09	9E-09
HSPA13	heat shock protein family A (Hsp70) meml	protein_coding	chr21:14371115-1	4153	8.421078366	12.50135865	0.483912	2E-09	9E-09
DGKH	diacylglycerol kinase eta	protein_coding	chr13:42040036-4	19637	5.652164653	8.57472404	0.516577	2E-09	9E-09
CRTAP	cartilage associated protein	protein_coding	chr3:33113979-33	6695	13.82278918	19.56736578	0.41577	2E-09	9E-09
BFAR	bifunctional apoptosis regulator	protein_coding	chr16:14632931-1	4285	8.575713238	12.36117492	0.439925	2E-09	1E-08
DNAJB5	DnaJ heat shock protein family (Hsp40) m	protein_coding	chr9:34989641-34	5891	20.01593422	28.29622571	0.4132	2E-09	1E-08
KNOP1	lysine rich nucleolar protein 1	protein_coding	chr16:19701937-1	7758	1.578329627	2.642426738	0.658309	2E-09	1E-08
SPIRE1	spire type actin nucleation factor 1	protein_coding	chr18:12446512-1	6532	5.230099737	7.749926053	0.483505	2E-09	1E-08
STK32C	serine/threonine kinase 32C	protein_coding	chr10:132207492-	3553	1.505211924	3.07063476	0.936935	2E-09	1E-08
LINC02361	long intergenic non-protein coding RNA 2	lncRNA	chr12:132186735-	1484	0.372033959	1.562895601	1.988501	2E-09	1E-08
PAN3	poly(A) specific ribonuclease subunit PAN	protein_coding	chr13:28138506-2	6352	2.987869812	4.959693768	0.643015	2E-09	1E-08
MPND	MPN domain containing	protein_coding	chr19:4343527-43	2049	18.18666661	25.73826708	0.414793	2E-09	1E-08
EBLN3P	endogenous Bornavirus like nucleoprotei	lncRNA	chr9:37079645-37	10492	4.387084982	6.171137754	0.406475	2E-09	1E-08
ARMC9	armadillo repeat containing 9	protein_coding	chr2:231198546-2	9661	0.806512297	1.508731435	0.817808	2E-09	1E-08
MBLAC2	metallo-beta-lactamase domain containin	protein_coding	chr5:90458209-90	5673	1.507930786	2.634905334	0.722388	2E-09	1E-08
KIF5B	kinesin family member 5B	protein_coding	chr10:32009015-3	5866	35.03475312	47.58861457	0.355626	2E-09	1E-08
PRR16	proline rich 16	protein_coding	chr5:120464300-1	2252	3.423122584	6.958982401	0.940648	2E-09	1E-08
ABHD4	abhydrolase domain containing 4	protein_coding	chr14:22598237-2 4070		6.741940398	10.09874884	0.497423	2E-09	1E-08
SPRED1	sprouty related EVH1 domain containing	protein_coding	chr15:38252836-3	7750	1.105330904	1.852159208	0.658445	2E-09	1E-08
KCTD7	potassium channel tetramerization doma	protein_coding	chr7:66628767-66	7565	2.182803563	3.623972596	0.64954	2E-09	1E-08
MALT1	MALT1 paracaspase	protein_coding	chr18:58671465-5	11448	4.146116238	5.83132199	0.405951	2E-09	1E-08
NRAP	nebulin related anchoring protein	protein_coding	chr10:113588716-	5724	20.19551925	30.44845975	0.507257	2E-09	1E-08
WDYHV1	WDYHV motif containing 1	protein_coding	chr8:123416725-1	4930	1.654042475	2.958066279	0.748585	2E-09	1E-08
TXNDC15	thioredoxin domain containing 15	protein_coding	chr5:134873803-1	5265	6.361651017	9.119705108	0.434088	2E-09	1E-08
RAB10	RAB10, member RAS oncogene family	protein_coding	chr2:26034084-26	3654	33.41753902	46.62897997	0.394839	2E-09	1E-08
BTG2	BTG anti-proliferation factor 2	protein_coding	chr1:203305491-2	2757	42.24328701	57.82747018	0.36686	2E-09	1E-08
NIPSNAP3B	nipsnap homolog 3B	protein_coding	chr9:104764129-1	5630	2.11351312	3.460447671	0.627224	2E-09	1E-08
BZW2	basic leucine zipper and W2 domains 2	protein_coding	chr7:16646131-16	3662	82.65456638	115.7850146	0.400171	2E-09	1E-08
DOT1L	DOT1 like histone lysine methyltransfera	protein_coding	chr19:2163933-22	11488	17.09494126	25.80839367	0.509893	2E-09	1E-08
DHX34	DExH-box helicase 34	protein_coding	chr19:47349315-4	5662	3.268644604	5.217400599	0.588854	2E-09	1E-08
DDX10	DEAD-box helicase 10	protein_coding	chr11:108665069-	8606	1.052541695	1.924111424	0.782232	2E-09	1E-08
FAAP20	FA core complex associated protein 20	protein_coding	chr1:2184461-221	10916	4.268626948	6.509659046	0.521723	2E-09	1E-08
ABHD11	abhydrolase domain containing 11	protein_coding	chr7:73736094-73	2289	13.22494504	20.54632495	0.552239	2E-09	1E-08
NUDT3	nudix hydrolase 3	protein_coding	chr6:34279679-34	9900	6.259856618	8.678907568	0.384656	2E-09	1E-08
CACNB2	calcium voltage-gated channel auxiliary s	protein_coding	chr10:18140677-1	10933	7.896918542	11.20221753	0.418104	2E-09	1E-08
LPCAT4	lysophosphatidylcholine acyltransferase 4	protein_coding	chr15:34358633-3	4702	15.9060434	21.98499925	0.380657	2E-09	1E-08
VEZT	vezatin, adherens junctions transmembra	protein_coding	chr12:95217746-9	8766	10.10852342	14.13759268	0.397577	2E-09	1E-08

AQP1	aquaporin 1 (Colton blood group)	protein_coding	chr7:30911694-30	3436	29.26843692	46.10948135	0.570592	2E-09	1E-08
RSL24D1	ribosomal L24 domain containing 1	protein_coding	chr15:55180806-5	2965	10.79348292	19.39374997	0.763795	2E-09	1E-08
BTBD8	BTB domain containing 8	protein_coding	chr1:92080305-92	6887	0.44104679	1.078840065	1.209936	2E-09	1E-08
ACO1	aconitase 1	protein_coding	chr9:32384603-32	7555	8.240689052	11.38193405	0.380067	2E-09	1E-08
TP53I3	tumor protein p53 inducible protein 3	protein_coding	chr2:24077433-24	2312	40.42157765	70.64155624	0.719268	2E-09	1E-08
URI1	URI1 prefoldin like chaperone	protein_coding	chr19:29923644-3	5448	7.285446626	11.27364338	0.545984	2E-09	1E-08
TRIM28	tripartite motif containing 28	protein_coding	chr19:58544064-5	4306	61.61491605	85.31067937	0.383142	2E-09	1E-08
SHTN1	shootin 1	protein_coding	chr10:116881477-	8252	0.481149604	0.998533379	0.967131	2E-09	1E-08
ARHGAP32	Rho GTPase activating protein 32	protein_coding	chr11:128965060-	12841	1.744551376	2.755072888	0.575705	2E-09	1E-08
NFIL3	nuclear factor, interleukin 3 regulated	protein_coding	chr9:91409045-91	2055	7.641850375	12.3512542	0.60775	2E-09	1E-08
KDM4C	lysine demethylase 4C	protein_coding	chr9:6720863-717	10323	5.012325535	7.145269793	0.425177	2E-09	1E-08
FAM45A	family with sequence similarity 45 membe	protein_coding	chr10:119104086-	6291	3.855551451	5.913258062	0.53054	2E-09	1E-08
AC008443.1	novel transcript, antisense to TRIM52	lncRNA	chr5:181246507-1	3328	2.172876516	3.876265458	0.746196	2E-09	1E-08
ASB15	ankyrin repeat and SOCS box containing 1	protein_coding	chr7:123567010-1	4912	0.285465554	0.90988898	1.580003	2E-09	1E-08
DDX21	DExD-box helicase 21	protein_coding	chr10:68956170-6	4973	14.83648156	20.95641408	0.41163	3E-09	1E-08
MICAL1	microtubule associated monooxygenase,	protein_coding	chr6:109444062-1	6841	0.438403585	1.067011936	1.206085	3E-09	1E-08
JAM3	junctional adhesion molecule 3	protein_coding	chr11:134068925-	5451	4.508914112	6.954519326	0.538493	3E-09	1E-08
ECI2	enoyl-CoA delta isomerase 2	protein_coding	chr6:4115689-413	4579	16.83961201	23.88365758	0.418879	3E-09	1E-08
SUPT3H	SPT3 homolog, SAGA and STAGA complex	protein_coding	chr6:44809317-45	5200	1.464596259	2.631358478	0.759856	3E-09	1E-08
LZTS2	leucine zipper tumor suppressor 2	protein_coding	chr10:100996618-	6547	13.77710104	20.65743033	0.499253	3E-09	1E-08
HCG11	HLA complex group 11	lncRNA	chr6:26521709-26	5696	1.119588619	2.057840679	0.793539	3E-09	1E-08
IRX3	iroquois homeobox 3	protein_coding	chr16:54283304-5	2935	25.11544342	39.78327603	0.578131	3E-09	1E-08
LYSMD3	LysM domain containing 3	protein_coding	chr5:90515611-90	4459	4.748966658	7.378853446	0.547204	3E-09	1E-08
ANXA2	annexin A2	protein_coding	chr15:60347134-6	8824	16.32268785	25.71400981	0.570879	3E-09	1E-08
ZNF771	zinc finger protein 771	protein_coding	chr16:30407297-3	5637	2.678469502	4.531876529	0.668647	3E-09	1E-08
COQ2	coenzyme Q2, polyprenyltransferase	protein_coding	chr4:83261536-83	2774	6.369670416	10.50598769	0.635456	3E-09	1E-08
SEC61A1	Sec61 translocon alpha 1 subunit	protein_coding	chr3:128051641-1	4640	30.25426276	41.49178742	0.369355	3E-09	1E-08
HOMER2	homer scaffold protein 2	protein_coding	chr15:82836946-8	14685	1.690303619	2.608026938	0.537474	3E-09	1E-08
TRAM1L1	translocation associated membrane prote	protein_coding	chr4:117083554-1	2023	0.701676528	2.102137352	1.515626	3E-09	1E-08
ZNF579	zinc finger protein 579	protein_coding	chr19:55576774-5	3320	8.178506837	11.8507531	0.448561	3E-09	1E-08
GFPT2	glutamine-fructose-6-phosphate transami	protein_coding	chr5:180300698-1	4721	2.442554228	3.898875781	0.589763	3E-09	1E-08
DYNLL2	dynein light chain LC8-type 2	protein_coding	chr17:58083419-5	6807	21.40660264	32.61885282	0.52315	3E-09	1E-08
DOCK7	dedicator of cytokinesis 7	protein_coding	chr1:62454298-62	24872	1.979482508	2.924741111	0.477603	3E-09	1E-08
ERCC6L2	ERCC excision repair 6 like 2	protein_coding	chr9:95871264-96	15525	2.294875851	3.356724538	0.462672	3E-09	1E-08
CAPZA2	capping actin protein of muscle Z-line sub	protein_coding	chr7:116810070-1	8368	27.06242012	39.48372769	0.459932	3E-09	1E-08
NCKAP1	NCK associated protein 1	protein_coding	chr2:182909115-1	22520	15.19453085	20.4484109	0.342257	3E-09	1E-08
REPS2	RALBP1 associated Eps domain containing	protein_coding	chrX:16946658-17	11336	3.021881064	4.699454747	0.551442	3E-09	1E-08
SCFD2	sec1 family domain containing 2	protein_coding	chr4:52872982-53	3176	2.332018229	3.937630638	0.667728	3E-09	1E-08
SCAI	suppressor of cancer cell invasion	protein_coding	chr9:124942608-1	13014	1.705716472	2.664396658	0.555915	3E-09	1E-08
SPR	sepiapterin reductase	protein_coding	chr2:72887382-72	1458	10.31492332	18.13530685	0.728873	3E-09	1E-08
PES1	pescadillo ribosomal biogenesis factor 1	protein_coding	chr22:30576625-3	4527	9.561714814	13.67198471	0.429171	3E-09	1E-08
MEAK7	MTOR associated protein, eak-7 homolog	protein_coding	chr16:84476355-8	8157	1.650060067	2.585902772	0.56073	3E-09	1E-08
CHMP1A	charged multivesicular body protein 1A	protein_coding	chr16:89644435-8 3080		18.94357406	26.37481748	0.390502	3E-09	1E-08
EIF5B	eukaryotic translation initiation factor 5E	protein_coding	chr2:99337371-99	7865	18.83218182	30.88213943	0.630253	3E-09	2E-08
EZH1	enhancer of zeste 1 polycomb repressive c	protein_coding	chr17:42700275-4	6988	7.365581599	10.4009878	0.410639	3E-09	2E-08
TTC7B	tetratricopeptide repeat domain 7B	protein_coding	chr14:90524564-9	21268	2.844643996	4.040320322	0.419638	3E-09	2E-08
STAU2	staufen double-stranded RNA binding pro	protein_coding	chr8:73420369-73	8964	7.312295711	10.17684107	0.390541	3E-09	2E-08
SYCN	syncollin	protein_coding	chr19:39202831-3	573	0.169086267	1.79319734	3.206154	3E-09	2E-08
GK5	glycerol kinase 5	protein_coding	chr3:142157527-1	12143	1.338128307	2.025507091	0.512361	3E-09	2E-08
COG3	component of oligomeric golgi complex 3	protein_coding	chr13:45464898-4	6087	3.757607639	5.906819556	0.570865	3E-09	2E-08
MRPS2	mitochondrial ribosomal protein S2	protein_coding	chr9:135499984-1	2464	20.49305588	28.10357713	0.369537	3E-09	2E-08
FLVCR2	feline leukemia virus subgroup C cellular r	protein_coding	chr14:75578620-7	4910	0.546933303	1.324493377	1.196885	3E-09	2E-08
ERLIN1	ER lipid raft associated 1	protein_coding	chr10:100150094-	3491	2.953538045	4.708136068	0.586676	3E-09	2E-08
TENT2	terminal nucleotidyltransferase 2	protein_coding	chr5:79612120-79	5508	6.470187373	9.423519139	0.457124	3E-09	2E-08
MEA1	male-enhanced antigen 1	protein_coding	chr6:43011143-43	3017	10.02703306	14.74137719	0.469024	3E-09	2E-08
ZNF449	zinc finger protein 449	protein_coding	chrX:135344796-1	5330	2.348044764	3.633716814	0.545365	3E-09	2E-08
CITED2	Cbp/p300 interacting transactivator with	protein_coding	chr6:139371807-1	2563	66.09189071	92.61441477	0.400762	3E-09	2E-08
PCDH17	protocadherin 17	protein_coding	chr13:57631744-5	8392	2.356494701	3.51439874	0.491944	3E-09	2E-08
TMEM35A	transmembrane protein 35A	protein_coding	chrX:101078879-1	2415	0.592516415	1.676880337	1.407892	4E-09	2E-08
TRAPPC2	trafficking protein particle complex 2	protein_coding	chrX:13712244-13	6107	1.320240034	2.601475283	0.884601	4E-09	2E-08
POLR3G	RNA polymerase III subunit G	protein_coding	chr5:90471748-90	4023	0.427041185	1.195890252	1.408611	4E-09	2E-08
REEP3	receptor accessory protein 3	protein_coding	chr10:63521401-6	5215	22.30464595	30.30475327	0.355793	4E-09	2E-08
REXO1	RNA exonuclease 1 homolog	protein_coding	chr19:1815248-18	5579	9.247490773	12.96106529	0.401945	4E-09	2E-08
UBTF	upstream binding transcription factor	protein_coding	chr17:44205033-4	6993	13.19939652	18.37007828	0.390734	4E-09	2E-08
BTAF1	B-TFIID TATA-box binding protein associat	protein_coding	chr10:91923770-9	7581	12.22951127	18.25209522	0.493058	4E-09	2E-08
PNMA8B	PNMA family member 8B	protein_coding	chr19:46486906-4	5506	0.695114658	1.425392294	0.950716	4E-09	2E-08
MAN2B2	mannosidase alpha class 2B member 2	protein_coding	chr4:6575175-662	8898	2.209843986	3.774704807	0.692872	4E-09	2E-08
CXXC4	CXXC finger protein 4	protein_coding	chr4:104468308-1	5823	3.629813335	6.10928349	0.666277	4E-09	2E-08
EIF3B	eukaryotic translation initiation factor 3 s	protein_coding	chr7:2354086-238	9037	12.00992416	17.56497692	0.463246	4E-09	2E-08
ITPRIPL2	ITPRIP like 2	protein_coding	chr16:19113932-1	7440	1.941834334	3.713544076	0.852395	4E-09	2E-08
MAFA-AS1	MAFA antisense RNA 1	lncRNA	chr8:143417679-1	417	0.122958455	2.464537538	4.068126	4E-09	2E-08
GPR162	G protein-coupled receptor 162	protein_coding	chr12:6821624-68	3216	2.198418691	3.682079249	0.660191	4E-09	2E-08
TAF15	TATA-box binding protein associated factc	protein_coding	chr17:35713791-3	5883	23.66599858	33.00354516	0.393477	4E-09	2E-08
HSPA4	heat shock protein family A (Hsp70) meml	protein_coding	chr5:133052013-1	5102	37.78684718	51.54945228	0.362026	4E-09	2E-08
PARP11	poly(ADP-ribose) polymerase family meml	protein_coding	chr12:3791047-38	6200	0.862033084	1.666320233	0.873257	4E-09	2E-08
DHRS11	dehydrogenase/reductase 11	protein_coding	chr17:36591879-3	5379	1.529212607	2.789214593	0.775979	4E-09	2E-08
LSM2	LSM2 homolog, U6 small nuclear RNA anc	protein_coding	chr6:31797396-31	1059	12.10499723	19.45998717	0.602456	4E-09	2E-08
CAMK2D	calcium/calmodulin dependent protein k	protein_coding	chr4:113451032-1	7363	72.48974855	97.8417635	0.346568	4E-09	2E-08
C1orf115	chromosome 1 open reading frame 115	protein_coding	chr1:220690363-2	2891	3.158546564	6.212737052	0.891035	4E-09	2E-08
AKIRIN1	akirin 1	protein_coding	chr1:38991276-39	2688	87.54472272	117.5485131	0.338823	4E-09	2E-08
CCDC71L	coiled-coil domain containing 71 like	protein_coding	chr7:106654360-1	6799	1.275029125	2.160327161	0.670637	4E-09	2E-08
CLEC2A	C-type lectin domain family 2 member A	protein_coding	chr12:9898673-99	1220	1.62470498	4.681929005	1.437695	4E-09	2E-08
GABBR1	gamma-aminobutyric acid type B recepto	protein_coding	chr6:29555629-29	8468	11.01212064	18.10717171	0.633657	5E-09	2E-08
INPPL1	inositol polyphosphate phosphatase like :	protein_coding	chr11:72223701-7	6574	41.09113572	61.42782302	0.495202	5E-09	2E-08
PON2	paraoxonase 2	protein_coding	chr7:95404862-95	3844	14.19180575	20.03201096	0.411724	5E-09	2E-08
EDRF1	erythroid differentiation regulatory facto	protein_coding	chr10:125719515-	7741	3.583361614	5.070636088	0.41467	5E-09	2E-08
COL16A1	collagen type XVI alpha 1 chain	protein_coding	chr1:31652263-31	9253	10.53112361	15.86266384	0.504723	5E-09	2E-08
ABCB9	ATP binding cassette subfamily B member	protein_coding	chr12:122920951-	13291	1.030653817	1.543876214	0.496311	5E-09	2E-08
AC008966.1	novel transcript, antisense to MOCS2	lncRNA	chr5:53109816-53	8072	0.576385879	1.16197027	0.934931	5E-09	2E-08
VASP	vasodilator stimulated phosphoprotein	protein_coding	chr19:45506579-4	3538	19.24369653	27.27138764	0.416451	5E-09	2E-08
CLPX	caseinolytic mitochondrial matrix peptid	protein_coding	chr15:65148219-6	5461	14.59271661	19.71465834	0.347591	5E-09	2E-08
DCK	deoxycytidine kinase	protein_coding	chr4:70992538-71	3103	4.27723773	7.080673738	0.646629	5E-09	2E-08
ROR1	receptor tyrosine kinase like orphan recep	protein_coding	chr1:63774017-64	6720	22.90663754	31.76493652	0.385843	5E-09	2E-08
ZYX	zyxin	protein_coding	chr7:143381295-1	3267	66.93280741	93.33368234	0.393107	5E-09	2E-08
KCTD9	potassium channel tetramerization doma	protein_coding	chr8:25427847-25	4383	7.555004292	11.28623292	0.496017	5E-09	2E-08
HABP4	hyaluronan binding protein 4	protein_coding	chr9:96450169-96	4088	11.76201856	17.19902996	0.462317	5E-09	2E-08
SNHG4	small nucleolar RNA host gene 4	lncRNA	chr5:139274102-1	4469	1.196912863	2.706875205	1.089277	5E-09	2E-08
FRA10AC1	FRA10A associated CGG repeat 1	protein_coding	chr10:93667883-9	8162	3.559407231	5.35478145	0.504595	5E-09	2E-08
VAMP7	vesicle associated membrane protein 7	protein_coding	chrX:155881345-1	2615	8.438402364	14.38358218	0.689693	5E-09	2E-08
ZFP69	ZFP69 zinc finger protein	protein_coding	chr1:40477290-40	3173	0.404649227	1.246620862	1.549403	5E-09	2E-08
RABGGTB	Rab geranylgeranyltransferase subunit bet	protein_coding	chr1:75786197-75	5920	5.545084362	8.745675038	0.570774	5E-09	2E-08
POLR1E	RNA polymerase I subunit E	protein_coding	chr9:37485948-37	2499	3.286358841	5.441304671	0.639425	5E-09	2E-08
RFXAP	regulatory factor X associated protein	protein_coding	chr13:36819224-3	2306	2.527906738	5.300910839	0.985201	5E-09	2E-08
MIR1915HG	MIR1915 host gene	protein_coding	chr10:21492658-2	3823	12.28404866	19.31714909	0.565512	5E-09	3E-08
RHOC	ras homolog family member C	protein_coding	chr1:112701106-1	4028	18.6829287	30.47401137	0.622133	5E-09	3E-08
CALM3	calmodulin 3	protein_coding	chr19:46601074-4	5235	63.58243125	86.27108314	0.35375	5E-09	3E-08
WDFY1	WD repeat and FYVE domain containing 1	protein_coding	chr2:223855716-2	5485	3.501697041	5.500243359	0.568516	5E-09	3E-08
ZC2HC1A	zinc finger C2HC-type containing 1A	protein_coding	chr8:78666089-78	4139	3.453255194	5.283115555	0.528435	5E-09	3E-08
NSMCE1	NSE1 homolog, SMC5-SMC6 complex com	protein_coding	chr16:27224994-2	3745	1.585630823	3.082489876	0.869988	5E-09	3E-08
FOXK1	forkhead box K1	protein_coding	chr7:4682295-477	13106	5.389449857	7.875383362	0.46256	5E-09	3E-08
AC106820.4	novel transcript, intronic to TBC1D24	lncRNA	chr16:2476558-24	5616	0.465861295	1.01265023	1.028845	5E-09	3E-08

VRK2	VRK serine/threonine kinase 2	protein_coding	chr2:57907629-58	3531	4.099093433	6.583891672	0.596666	5E-09	3E-08
TMEM37	transmembrane protein 37	protein_coding	chr2:119429901-1	2291	0.462249204	1.761070168	1.822795	5E-09	3E-08
C1GALT1	core 1 synthase, glycoprotein-N-acetylgal	protein_coding	chr7:7156934-724	7805	4.567302002	6.600648825	0.444602	5E-09	3E-08
PGM1	phosphoglucomutase 1	protein_coding	chr1:63593411-63	4077	33.29314685	47.23854033	0.419566	5E-09	3E-08
FAM98B	family with sequence similarity 98 memb	protein_coding	chr15:38454127-3	6851	5.723405388	8.77242817	0.531682	6E-09	3E-08
ZNF354A	zinc finger protein 354A	protein_coding	chr5:178711512-1	2984	2.341889581	4.049053313	0.706144	6E-09	3E-08
CBX7	chromobox 7	protein_coding	chr22:39120167-3	7467	3.693309394	5.87381753	0.583675	6E-09	3E-08
SCART1	scavenger receptor family member expres	protein_coding	chr10:133453928-	8015	0.448930357	1.058129842	1.155144	6E-09	3E-08
ZCCHC2	zinc finger CCHC-type containing 2	protein_coding	chr18:62523007-6	10235	1.411623518	2.379713767	0.666476	6E-09	3E-08
MRPL45	mitochondrial ribosomal protein L45	protein_coding	chr17:38297023-3	1704	16.67283753	26.8384542	0.604826	6E-09	3E-08
PAK1	p21 (RAC1) activated kinase 1	protein_coding	chr11:77321707-7	6299	1.011818587	1.794865541	0.746293	6E-09	3E-08
RYBP	RING1 and YY1 binding protein	protein_coding	chr3:72371825-72	7215	8.421137881	12.02863594	0.428293	6E-09	3E-08
SERPINF1	serpin family F member 1	protein_coding	chr17:1762029-17	2990	0.649141734	1.831762481	1.420923	6E-09	3E-08
DOC2A	double C2 domain alpha	protein_coding	chr16:30005514-3	6368	0.454998808	1.082743536	1.165129	6E-09	3E-08
GPR27	G protein-coupled receptor 27	protein_coding	chr3:71754050-71	2447	2.436182951	5.153278177	0.990787	6E-09	3E-08
JOSD2	Josephin domain containing 2	protein_coding	chr19:50505998-5	1514	13.55655076	21.52804087	0.576119	6E-09	3E-08
PLPPR3	phospholipid phosphatase related 3	protein_coding	chr19:812488-821	2741	2.591223771	5.371253043	0.969578	6E-09	3E-08
BAIAP2	BAI1 associated protein 2	protein_coding	chr17:81035122-8	10288	0.861741994	1.617117034	0.813543	6E-09	3E-08
RBMX	RNA binding motif protein X-linked	protein_coding	chrX:136848004-1	5882	21.89897863	29.28020077	0.332707	6E-09	3E-08
BRD7	bromodomain containing 7	protein_coding	chr16:50313487-5	8492	6.890992814	9.711158775	0.409615	6E-09	3E-08
PRPF38A	pre-mRNA processing factor 38A	protein_coding	chr1:52404602-52	5617	4.66500988	7.058953637	0.514741	6E-09	3E-08
DCAF6	DDB1 and CUL4 associated factor 6	protein_coding	chr1:167935783-1	6306	29.2693094	39.3486638	0.340515	6E-09	3E-08
KCTD18	potassium channel tetramerization doma	protein_coding	chr2:200488958-2	4909	1.943858803	3.362884586	0.708548	6E-09	3E-08
MANSC1	MANSC domain containing 1	protein_coding	chr12:12326056-1	5716	1.565214257	2.575544948	0.635828	6E-09	3E-08
PSMD12	proteasome 26S subunit, non-ATPase 12	protein_coding	chr17:67337916-6	5342	9.012123213	12.52842928	0.389056	6E-09	3E-08
RARA-AS1	RARA antisense RNA 1	lncRNA	chr17:40340867-4	1790	0.881946438	2.228571547	1.242818	7E-09	3E-08
NCL	nucleolin	protein_coding	chr2:231453531-2	6819	60.83334785	83.15420576	0.364886	7E-09	3E-08
AP1S1	adaptor related protein complex 1 subuni	protein_coding	chr7:101154456-1	1699	8.6733243	15.97586651	0.795087	7E-09	3E-08
COPS7B	COP9 signalosome subunit 7B	protein_coding	chr2:231781671-2	6066	4.685448967	6.615826412	0.41225	7E-09	3E-08
SPIDR	scaffold protein involved in DNA repair	protein_coding	chr8:47260575-47	7477	2.415974218	3.650638258	0.509861	7E-09	3E-08
CENPBD1	CENPB DNA-binding domain containing 1	protein_coding	chr16:89969773-8 3060		3.937545499	6.278966496	0.585801	7E-09	3E-08
TSPYL4	TSPY like 4	protein_coding	chr6:116249961-1	4180	16.25897106	22.2943257	0.369631	7E-09	3E-08
ISYNA1	inositol-3-phosphate synthase 1	protein_coding	chr19:18434388-1	3284	72.49230822	97.38245814	0.339297	7E-09	3E-08
ARCN1	archain 1	protein_coding	chr11:118572390-	4177	33.24998569	44.82635643	0.344603	7E-09	3E-08
SPACA9	sperm acrosome associated 9	protein_coding	chr9:132878027-1	2823	1.593702345	3.090066733	0.865103	7E-09	3E-08
PTH1R	parathyroid hormone 1 receptor	protein_coding	chr3:46877721-46	2783	16.67116822	23.21777603	0.391023	7E-09	3E-08
NBN	nibrin	protein_coding	chr8:89933336-90	6156	4.539185871	6.401886282	0.409089	7E-09	3E-08
IFT46	intraflagellar transport 46	protein_coding	chr11:118544528-	3814	2.297478698	3.823459918	0.648465	7E-09	3E-08
ADA	adenosine deaminase	protein_coding	chr20:44619522-4	2809	2.714695609	4.719398446	0.706401	7E-09	3E-08
ATXN7L3	ataxin 7 like 3	protein_coding	chr17:44191805-4	5301	15.3944703	21.92021281	0.424977	7E-09	3E-08
ELK3	ETS transcription factor ELK3	protein_coding	chr12:96194375-9	4644	0.38045534	1.162659997	1.533406	7E-09	3E-08
NRBP1	nuclear receptor binding protein 1	protein_coding	chr2:27427790-27	5329	16.55923656	22.56428896	0.360164	7E-09	3E-08
MRPS27	mitochondrial ribosomal protein S27	protein_coding	chr5:72219409-72	6511	7.996320994	11.31005518	0.414105	7E-09	4E-08
DHRS7C	dehydrogenase/reductase 7C	protein_coding	chr17:9771434-97	1006	0.563884114	2.576384971	2.081423	8E-09	4E-08
GSPT2	G1 to S phase transition 2	protein_coding	chrX:51743431-51	2802	6.527188357	10.64690102	0.621781	8E-09	4E-08
PTER	phosphotriesterase related	protein_coding	chr10:16436943-1	4003	0.705456169	1.586008596	1.07857	8E-09	4E-08
MAP3K7	mitogen-activated protein kinase kinase k	protein_coding	chr6:90513573-90	7254	9.377523154	12.67721782	0.348623	8E-09	4E-08
AHI1	Abelson helper integration site 1	protein_coding	chr6:135283532-1	8694	1.243776535	1.971939507	0.581557	8E-09	4E-08
AL096870.1	TERF1 interacting nuclear factor 2	protein_coding	chr14:24239643-2	2712	6.923278024	10.2303229	0.476643	8E-09	4E-08
FAM241B	family with sequence similarity 241 meml	protein_coding	chr10:69630247-6	1350	3.466313947	6.160998705	0.74187	8E-09	4E-08
TBCD	tubulin folding cofactor D	protein_coding	chr17:82752065-8	13537	4.915813686	6.911426741	0.406691	8E-09	4E-08
RRP7A	ribosomal RNA processing 7 homolog A	protein_coding	chr22:42508344-4	5700	5.945454011	8.231185346	0.3818	8E-09	4E-08
PNISR	PNN interacting serine and arginine rich p	protein_coding	chr6:99398050-99	10871	17.0910435	23.49530341	0.372927	8E-09	4E-08
AP4B1	adaptor related protein complex 4 subuni	protein_coding	chr1:113894194-1	5847	2.221651321	3.598754866	0.612842	8E-09	4E-08
FAM169A	family with sequence similarity 169 meml	protein_coding	chr5:74777574-74	6875	1.84108133	2.910497083	0.572903	8E-09	4E-08
GTDC1	glycosyltransferase like domain containin	protein_coding	chr2:143938068-1	14162	0.944445633	1.448417138	0.529832	9E-09	4E-08
SLC30A2	solute carrier family 30 member 2	protein_coding	chr1:26037252-26	3498	0.387991042	1.006878896	1.281392	9E-09	4E-08
SPATA6	spermatogenesis associated 6	protein_coding	chr1:48295373-48	5938	0.85116399	1.52778335	0.755238	9E-09	4E-08
HIF3A	hypoxia inducible factor 3 subunit alpha	protein_coding	chr19:46297046-4	8371	13.05632816	18.72802765	0.435092	9E-09	4E-08
HSPA4L	heat shock protein family A (Hsp70) meml	protein_coding	chr4:127781821-1	11439	3.106711104	4.467482604	0.438079	9E-09	4E-08
MAP4K2	mitogen-activated protein kinase kinase k	protein_coding	chr11:64784918-6	7645	1.693547404	3.14885484	0.816764	9E-09	4E-08
FAM120AOS	family with sequence similarity 120A opp	protein_coding	chr9:93431441-93	10144	6.461843371	8.744962911	0.349773	9E-09	4E-08
PRRC2C	proline rich coiled-coil 2C	protein_coding	chr1:171485551-1	14173	9.009085412	14.07965555	0.559373	9E-09	4E-08
TADA2B	transcriptional adaptor 2B	protein_coding	chr4:7041899-705	5795	6.532244591	9.244580164	0.413431	9E-09	4E-08
FEZ1	fasciculation and elongation protein zeta	protein_coding	chr11:125442881-	9324	0.565358082	1.095934002	0.874683	9E-09	4E-08
NMRAL1	NmrA like redox sensor 1	protein_coding	chr16:4461680-44	3541	2.242791392	3.758923287	0.664546	9E-09	4E-08
SNHG19	small nucleolar RNA host gene 19	lncRNA	chr16:2154797-21	342	9.51795852	24.38374567	1.27086	1E-08	4E-08
MAGED2	MAGE family member D2	protein_coding	chrX:54807599-54	2943	128.1522682	175.147397	0.36465	1E-08	4E-08
SIAE	sialic acid acetylesterase	protein_coding	chr11:124633113-	8139	4.936393555	6.999598373	0.41738	1E-08	4E-08
COL14A1	collagen type XIV alpha 1 chain	protein_coding	chr8:120059780-1 10190		0.498766525	1.354597954	1.35733	1E-08	4E-08
CPOX	coproporphyrinogen oxidase	protein_coding	chr3:98579446-98	3157	8.0256917	13.16032207	0.629157	1E-08	4E-08
GSTP1	glutathione S-transferase pi 1	protein_coding	chr11:67583595-6	1911	78.62364245	123.8138581	0.572235	1E-08	4E-08
SUPT20H	SPT20 homolog, SAGA complex compone	protein_coding	chr13:37009312-3	10933	4.597493607	6.272239879	0.361403	1E-08	4E-08
LOH12CR2	loss of heterozygosity, 12, chromosomal r	lncRNA	chr12:12355406-1	1540	0.856126003	2.429575709	1.405023	1E-08	5E-08
ZNF33B	zinc finger protein 33B	protein_coding	chr10:42574185-4	8309	3.19336075	4.711613661	0.475308	1E-08	5E-08
C14orf28	chromosome 14 open reading frame 28	protein_coding	chr14:44897275-4	3884	1.47972685	2.728955239	0.792887	1E-08	5E-08
DNAJC3-DT	DNAJC3 divergent transcript	lncRNA	chr13:95648733-9	3591	1.340383662	2.460857398	0.785868	1E-08	5E-08
DBP	D-box binding PAR bZIP transcription fact	protein_coding	chr19:48630030-4	6544	4.039649447	6.13258938	0.518939	1E-08	5E-08
ZMAT1	zinc finger matrin-type 1	protein_coding	chrX:101882288-1	7562	2.309247778	3.524184017	0.523635	1E-08	5E-08
CAVIN1	caveolae associated protein 1	protein_coding	chr17:42402449-4	3570	210.580405	293.9722444	0.396405	1E-08	5E-08
ILRUN	inflammation and lipid regulator with UB	protein_coding	chr6:34587288-34	4551	27.57164256	38.01954679	0.378018	1E-08	5E-08
TAF2	TATA-box binding protein associated facto	protein_coding	chr8:119730774-1	6526	3.951484221	5.711392054	0.443712	1E-08	5E-08
PNPLA4	patatin like phospholipase domain contai	protein_coding	chrX:7898247-792	3411	9.089763108	13.61593387	0.497496	1E-08	5E-08
IL17RA	interleukin 17 receptor A	protein_coding	chr22:17084954-1	9036	0.583136678	1.02795566	0.730337	1E-08	5E-08
AREG	amphiregulin	protein_coding	chr4:74445136-74	1642	0.154376651	1.013949294	2.561013	1E-08	5E-08
PBX1	PBX homeobox 1	protein_coding	chr1:164555584-1	21249	8.586431569	12.16598762	0.416446	1E-08	5E-08
PRKAR1A	protein kinase cAMP-dependent type I reg	protein_coding	chr17:68511780-6	6821	78.32676514	108.3064767	0.382007	1E-08	5E-08
IQCB1	IQ motif containing B1	protein_coding	chr3:121769761-1	3051	5.726035546	8.210144966	0.434265	1E-08	5E-08
GUCY1B1	guanylate cyclase 1 soluble subunit beta 1	protein_coding	chr4:155758992-1	3907	3.393517155	5.32493377	0.560655	1E-08	5E-08
ZFP69B	ZFP69 zinc finger protein B	protein_coding	chr1:40450102-40	2566	0.379420016	1.264608417	1.645051	1E-08	5E-08
ABTB1	ankyrin repeat and BTB domain containin	protein_coding	chr3:127672935-1	6155	1.357669597	2.38364101	0.720216	1E-08	5E-08
CPNE1	copine 1	protein_coding	chr20:35626031-3	3608	15.44629998	22.74121692	0.474751	1E-08	5E-08
AL049834.1	spermatogenesis associated 7	protein_coding	chr14:88384924-8	6872	1.777210019	2.848537259	0.592662	1E-08	5E-08
NUDT12	nudix hydrolase 12	protein_coding	chr5:103548855-1	4301	1.845548928	3.134061751	0.675981	1E-08	5E-08
CHSY3	chondroitin sulfate synthase 3	protein_coding	chr5:129904465-1	3945	2.145636431	3.876499556	0.761479	1E-08	5E-08
CCDC159	coiled-coil domain containing 159	protein_coding	chr19:11344684-1	4064	1.299775659	2.609089174	0.922332	1E-08	5E-08
PABPC4L	poly(A) binding protein cytoplasmic 4 like	protein_coding	chr4:134196333-1	4985	0.812520266	1.637556628	0.920032	1E-08	5E-08
DENR	density regulated re-initiation and release	protein_coding	chr12:122752824-	3526	19.57744251	27.53213882	0.406521	1E-08	5E-08
RAB2B	RAB2B, member RAS oncogene family	protein_coding	chr14:21459020-2	3597	5.970420313	8.931808477	0.494008	1E-08	5E-08
FEZ2	fasciculation and elongation protein zeta	protein_coding	chr2:36531805-36	8938	3.226860066	4.636634142	0.436389	1E-08	5E-08
MTIF3	mitochondrial translational initiation fac	protein_coding	chr13:27435643-2	1603	17.83192581	26.01879661	0.459121	1E-08	5E-08
C17orf100	chromosome 17 open reading frame 100	protein_coding	chr17:6651718-66	2474	0.468882088	1.384780464	1.469812	1E-08	5E-08
IFNAR1	interferon alpha and beta receptor subuni	protein_coding	chr21:33324429-3	6879	6.059889605	8.242181176	0.357933	1E-08	5E-08
NPEPPS	aminopeptidase puromycin sensitive	protein_coding	chr17:47522942-4	11470	8.591184613	11.58449715	0.345503	1E-08	5E-08
HAND2-AS1	HAND2 antisense RNA 1	lncRNA	chr4:173527270-1	21212	20.43544696	27.79419021	0.357914	1E-08	5E-08
KIZ	kizuna centrosomal protein	protein_coding	chr20:21125983-2	5207	1.338740052	2.531326892	0.828707	1E-08	5E-08
GPATCH11	G-patch domain containing 11	protein_coding	chr2:37084451-37 4040		3.698753537	5.517902002	0.490262	1E-08	5E-08
IMPACT	impact RWD domain protein	protein_coding	chr18:24426634-2	4896	5.777015238	8.482712334	0.465137	1E-08	5E-08
TMEM260	transmembrane protein 260	protein_coding	chr14:56488354-5	8072	2.047038291	3.042749009	0.487481	1E-08	5E-08
LRP8	LDL receptor related protein 8	protein_coding	chr1:53242356-53	19676	0.674124094	1.049230434	0.552318	1E-08	5E-08
MT2A	metallothionein 2A	protein_coding	chr16:56608584-5	914	4.064290352	8.798599776	1.022748	1E-08	5E-08

STXBP3	syntaxin binding protein 3	protein_coding	chr1:108746674-1	5892	4.878245636	7.174840161	0.470166	1E-08	5E-08
HIBADH	3-hydroxyisobutyrate dehydrogenase	protein_coding	chr7:27525442-27	2211	29.39862186	40.10885363	0.362729	1E-08	5E-08
ANKRD36	ankyrin repeat domain 36	protein_coding	chr2:97113218-97	11132	0.709526337	1.274404135	0.767946	1E-08	5E-08
UBA2	ubiquitin like modifier activating enzyme	protein_coding	chr19:34428352-3	4815	10.68771342	15.15573059	0.419039	1E-08	5E-08
RPARP-AS1	RPARP antisense RNA 1	lncRNA	chr10:102449816-	6160	0.94333666	1.693772118	0.757059	1E-08	6E-08
NELFA	negative elongation factor complex meml	protein_coding	chr4:1982714-204	5935	4.171259277	5.904968538	0.415217	1E-08	6E-08
PPP4R3A	protein phosphatase 4 regulatory subunit	protein_coding	chr14:91457611-9	5912	12.30279828	16.56071046	0.342279	1E-08	6E-08
EPM2AIP1	EPM2A interacting protein 1	protein_coding	chr3:36985043-36	8126	12.96691597	17.42333605	0.339737	1E-08	6E-08
HYKK	hydroxylysine kinase	protein_coding	chr15:78507564-7	5707	0.478495319	1.070522901	1.078829	1E-08	6E-08
STT3A	STT3 oligosaccharyltransferase complex c	protein_coding	chr11:125591712-	7393	7.206349476	9.879158966	0.369048	1E-08	6E-08
TMEM173	transmembrane protein 173	protein_coding	chr5:139475533-1	4798	8.548428837	12.49609658	0.464702	1E-08	6E-08
OPHN1	oligophrenin 1	protein_coding	chrX:68042344-68	8089	5.179185511	7.259173591	0.399286	1E-08	6E-08
MCF2L	MCF.2 cell line derived transforming sequ	protein_coding	chr13:112894378-	23158	1.014840883	1.460073295	0.437391	1E-08	6E-08
VWA5A	von Willebrand factor A domain containir	protein_coding	chr11:124115362-	6403	2.344373723	3.502682628	0.493941	1E-08	6E-08
PAICS	phosphoribosylaminoimidazole carboxyla	protein_coding	chr4:56435741-56	7517	16.02773339	21.92666257	0.366375	1E-08	6E-08
TRPC1	transient receptor potential cation chann	protein_coding	chr3:142724034-1	4808	6.759759527	9.475792174	0.40006	1E-08	6E-08
TMED2	transmembrane p24 trafficking protein 2	protein_coding	chr12:123584533-	3833	27.4204238	36.69387834	0.33381	1E-08	6E-08
HACD3	3-hydroxyacyl-CoA dehydratase 3	protein_coding	chr15:65530418-6	7094	7.753924098	10.46204816	0.34547	1E-08	6E-08
TMEM106B	transmembrane protein 106B	protein_coding	chr7:12211270-12	13838	7.181460033	9.481298775	0.31428	1E-08	6E-08
UBLCP1	ubiquitin like domain containing CTD pho	protein_coding	chr5:159263290-1	2463	9.306483073	13.70386514	0.473853	1E-08	6E-08
H2AFJ	H2A histone family member J	protein_coding	chr12:14774383-1	3356	3.691663042	6.109830164	0.637991	1E-08	6E-08
C11orf95	chromosome 11 open reading frame 95	protein_coding	chr11:63759892-6	5948	8.352680628	12.72449061	0.519732	1E-08	6E-08
ERBB2	erb-b2 receptor tyrosine kinase 2	protein_coding	chr17:39687914-3	10321	60.48833711	85.14006449	0.408075	1E-08	6E-08
ZNF71	zinc finger protein 71	protein_coding	chr19:56595264-5	6170	1.434546942	2.337604614	0.615918	1E-08	6E-08
DHRS1	dehydrogenase/reductase 1	protein_coding	chr14:24290598-2	4949	1.402828541	2.389084493	0.676896	1E-08	6E-08
RBPJ	recombination signal binding protein for	protein_coding	chr4:26163455-26	9253	4.018871275	5.599557608	0.392153	1E-08	6E-08
TMEM177	transmembrane protein 177	protein_coding	chr2:119679167-1	2588	3.052119864	5.501627284	0.756483	1E-08	6E-08
WDFY2	WD repeat and FYVE domain containing 2	protein_coding	chr13:51584455-5	16199	0.810657689	1.248743192	0.534897	1E-08	6E-08
PDZRN3	PDZ domain containing ring finger 3	protein_coding	chr3:73382431-73	6139	21.85643666	29.49337664	0.346155	1E-08	6E-08
SEC23B	Sec23 homolog B, coat complex II compo	protein_coding	chr20:18507520-1	4347	3.721498569	5.908339803	0.575838	1E-08	6E-08
UBQLN2	ubiquilin 2	protein_coding	chrX:56563639-56	4230	20.45132006	27.95837348	0.364798	1E-08	6E-08
GALNT18	polypeptide N-acetylgalactosaminyltrans	protein_coding	chr11:11270877-1	3626	2.209049494	3.768397146	0.689716	1E-08	6E-08
IL17RD	interleukin 17 receptor D	protein_coding	chr3:57089982-57	9561	3.416323743	5.017706634	0.467511	1E-08	6E-08
KXD1	KxDL motif containing 1	protein_coding	chr19:18557762-1	4112	9.503357409	12.92225195	0.356416	1E-08	6E-08
RTN4R	reticulon 4 receptor	protein_coding	chr22:20241415-2	3126	2.755231815	4.591397845	0.651767	1E-08	6E-08
MYORG	myogenesis regulating glycosidase (putati	protein_coding	chr9:34366666-34	6447	1.025958687	1.879756511	0.781442	1E-08	6E-08
FAR1	fatty acyl-CoA reductase 1	protein_coding	chr11:13668668-1	7503	8.904738409	12.32620179	0.382992	1E-08	6E-08
C4B	complement C4B (Chido blood group)	protein_coding	chr6:32014795-32	7174	1.172190463	2.348397964	0.92251	1E-08	6E-08
RNF139	ring finger protein 139	protein_coding	chr8:124474738-1	2700	11.88464281	16.59358653	0.396446	1E-08	6E-08
AC091729.3	novel transcript	lncRNA	chr7:1160374-116	4569	0.858349815	1.767749993	0.955293	1E-08	6E-08
COG7	component of oligomeric golgi complex 7	protein_coding	chr16:23388493-2	4352	3.105943884	4.767807475	0.530906	1E-08	6E-08
PELI3	pellino E3 ubiquitin protein ligase family	protein_coding	chr11:66466327-6	3926	2.458638864	3.923433609	0.588687	2E-08	7E-08
AC092338.1	cerebellar degeneration related protein 2	protein_coding	chr16:22345936-2	3950	1.535640002	2.711971772	0.737411	2E-08	7E-08
FSD2	fibronectin type III and SPRY domain cont	protein_coding	chr15:82755362-8	6536	24.30165388	32.13341149	0.316716	2E-08	7E-08
SLC35B3	solute carrier family 35 member B3	protein_coding	chr6:8411463-843	2828	5.280138383	7.696964747	0.45583	2E-08	7E-08
RBMX2	RNA binding motif protein X-linked 2	protein_coding	chrX:130401987-1	2786	3.610185186	5.820492805	0.606151	2E-08	7E-08
PYGO1	pygopus family PHD finger 1	protein_coding	chr15:55538890-5	8615	2.128438269	3.271528373	0.531685	2E-08	7E-08
GDF11	growth differentiation factor 11	protein_coding	chr12:55743280-5	8657	8.32986706	12.28221101	0.474575	2E-08	7E-08
PDIA3	protein disulfide isomerase family A meml	protein_coding	chr15:43746410-4	4221	36.84427123	50.96527476	0.382222	2E-08	7E-08
CLIC4	chloride intracellular channel 4	protein_coding	chr1:24745382-24	4467	176.7603617	238.7763139	0.347551	2E-08	7E-08
SRD5A1	steroid 5 alpha-reductase 1	protein_coding	chr5:6633427-667	7631	1.407627418	2.214428032	0.566274	2E-08	7E-08
IFT88	intraflagellar transport 88	protein_coding	chr13:20567069-2	6111	2.690174243	3.965378374	0.472172	2E-08	7E-08
AC089985.1	novel transcript, antisense to SLC41A2	lncRNA	chr12:104858838-	1674	0.153575317	0.968488376	2.515161	2E-08	7E-08
EEF2	eukaryotic translation elongation factor 2	protein_coding	chr19:3976056-39	4021	623.8757459	855.2694988	0.368683	2E-08	7E-08
RPTOR	regulatory associated protein of MTOR co	protein_coding	chr17:80544819-8	10302	4.193705226	5.887391165	0.404908	2E-08	7E-08
KCTD5	potassium channel tetramerization doma	protein_coding	chr16:2682523-27	3372	8.26321469	12.46805202	0.509567	2E-08	7E-08
CTDSPL	CTD small phosphatase like	protein_coding	chr3:37861880-37	5876	3.420987385	5.060292625	0.478716	2E-08	7E-08
SSR2	signal sequence receptor subunit 2	protein_coding	chr1:156009048-1	6643	8.034095051	11.76783454	0.466324	2E-08	7E-08
PEF1	penta-EF-hand domain containing 1	protein_coding	chr1:31629866-31	2223	23.95271382	32.70588988	0.362439	2E-08	7E-08
CSNK1G3	casein kinase 1 gamma 3	protein_coding	chr5:123512099-1	5000	5.936526383	8.530197956	0.434565	2E-08	7E-08
RABL2B	RAB, member of RAS oncogene family like	protein_coding	chr22:50767501-5	5870	2.182565962	3.260751675	0.492689	2E-08	7E-08
WDSUB1	WD repeat, sterile alpha motif and U-box	protein_coding	chr2:159235793-1	2135	4.411095105	7.043113286	0.588359	2E-08	7E-08
HDGFL2	HDGF like 2	protein_coding	chr19:4472297-45	4286	20.54370775	28.21520839	0.371642	2E-08	7E-08
PCDHB2	protocadherin beta 2	protein_coding	chr5:141094578-1	4126	5.485542806	8.520118396	0.549276	2E-08	8E-08
RFC1	replication factor C subunit 1	protein_coding	chr4:39287456-39	6719	5.422017736	7.888373244	0.456158	2E-08	8E-08
MID2	midline 2	protein_coding	chrX:107825755-1	3097	0.781067604	1.816195009	1.127608	2E-08	8E-08
ANKRD63	ankyrin repeat domain 63	protein_coding	chr15:40281444-4	1143	2.860807033	6.629973242	1.120976	2E-08	8E-08
SHROOM1	shroom family member 1	protein_coding	chr5:132822141-1	4420	0.479368709	1.207462633	1.261354	2E-08	8E-08
ZNF213	zinc finger protein 213	protein_coding	chr16:3129777-31	6443	2.275992364	3.53047133	0.549989	2E-08	8E-08
GLUD1	glutamate dehydrogenase 1	protein_coding	chr10:87050202-8	3780	29.93206947	40.7061216	0.357636	2E-08	8E-08
BASP1	brain abundant membrane attached signa	protein_coding	chr5:17065598-17	2146	1.057285553	2.597546735	1.228228	2E-08	8E-08
NBEAL2	neurobeachin like 2	protein_coding	chr3:46979683-47	12041	5.481437224	9.261802447	0.675924	2E-08	8E-08
IARS2	isoleucyl-tRNA synthetase 2, mitochondri	protein_coding	chr1:220094132-2	4135	31.38013565	41.12025217	0.30377	2E-08	8E-08
ACSS3	acyl-CoA synthetase short chain family me	protein_coding	chr12:80936414-8	18465	0.889842793	1.277637399	0.435625	2E-08	8E-08
ACTN4	actinin alpha 4	protein_coding	chr19:38647649-3	6104	28.80361083	40.12354847	0.392881	2E-08	8E-08
PCDHB14	protocadherin beta 14	protein_coding	chr5:141222932-1	4828	0.798119004	1.480440561	0.802328	2E-08	8E-08
PTPN13	protein tyrosine phosphatase non-recept	protein_coding	chr4:86594315-86	9496	2.012727677	2.932654946	0.458126	2E-08	8E-08
STRBP	spermatid perinuclear RNA binding protei	protein_coding	chr9:123109500-1	8097	3.951212888	5.901704652	0.492944	2E-08	8E-08
ANGEL1	angel homolog 1	protein_coding	chr14:76786178-7	6414	4.348780164	6.387366479	0.467525	2E-08	8E-08
MEAF6	MYST/Esa1 associated factor 6	protein_coding	chr1:37492575-37	3487	9.106612787	12.65666209	0.387684	2E-08	8E-08
RIN2	Ras and Rab interactor 2	protein_coding	chr20:19757606-2	8273	5.419593233	7.440794486	0.370735	2E-08	8E-08
DRAM2	DNA damage regulated autophagy modula	protein_coding	chr1:111117333-1	3138	5.747666196	8.428683851	0.467411	2E-08	8E-08
GNPDA1	glucosamine-6-phosphate deaminase 1	protein_coding	chr5:141991749-1	3959	6.905344875	9.816299563	0.420972	2E-08	8E-08
USP2	ubiquitin specific peptidase 2	protein_coding	chr11:119355215-	4523	30.54141801	40.24379316	0.311651	2E-08	8E-08
DRG2	developmentally regulated GTP binding p	protein_coding	chr17:18087892-1	7404	3.970768187	5.593537627	0.408395	2E-08	8E-08
STXBP1	syntaxin binding protein 1	protein_coding	chr9:127579370-1	9373	3.347316535	4.898702146	0.463915	2E-08	8E-08
ZSCAN18	zinc finger and SCAN domain containing 1	protein_coding	chr19:58083838-5	8307	5.068452543	8.053407783	0.584207	2E-08	8E-08
MARCH2	membrane associated ring-CH-type finger	protein_coding	chr19:8413270-84	2216	25.13028617	33.95443316	0.347816	2E-08	8E-08
NFKB1	nuclear factor kappa B subunit 1	protein_coding	chr4:102501331-1	6359	3.472026796	4.91720846	0.417018	2E-08	8E-08
KIAA0895	KIAA0895	protein_coding	chr7:36324221-36	6728	2.936228905	4.26119261	0.448366	2E-08	8E-08
MAN1A1	mannosidase alpha class 1A member 1	protein_coding	chr6:119177205-1	5018	10.25949892	16.401006	0.592233	2E-08	8E-08
ANAPC15	anaphase promoting complex subunit 15	protein_coding	chr11:72106378-7	2883	5.852506226	9.364038485	0.593666	2E-08	8E-08
FEM1A	fem-1 homolog A	protein_coding	chr19:4791681-48	9593	11.67435504	15.30430015	0.304412	2E-08	8E-08
TMEM117	transmembrane protein 117	protein_coding	chr12:43835967-4	3440	7.010760878	9.803673628	0.396952	2E-08	8E-08
NOP10	NOP10 ribonucleoprotein	protein_coding	chr15:34341713-3	554	79.7195557	135.2006429	0.681864	2E-08	8E-08
CPS1	carbamoyl-phosphate synthase 1	protein_coding	chr2:210477682-2	10775	2.252948822	3.268588441	0.449362	2E-08	8E-08
ACTR3	actin related protein 3	protein_coding	chr2:113889960-1	12049	9.608249969	13.11636625	0.363522	2E-08	9E-08
EWSR1	EWS RNA binding protein 1	protein_coding	chr22:29268009-2	10326	19.29999685	26.42942703	0.368142	2E-08	9E-08
SRSF8	serine and arginine rich splicing factor 8	protein_coding	chr11:95067197-9	4028	5.927695046	8.551152047	0.442845	2E-08	9E-08
NES	nestin	protein_coding	chr1:156668763-1	5568	243.3500197	365.0758987	0.501043	2E-08	9E-08
PDCD7	programmed cell death 7	protein_coding	chr15:65117379-6	3235	8.991119065	12.38545766	0.375351	2E-08	9E-08
XKR8	XK related 8	protein_coding	chr1:27959462-27	2743	6.925123571	9.919895878	0.432705	2E-08	9E-08
CARMIL1	capping protein regulator and myosin 1 li	protein_coding	chr6:25279078-25	6853	4.350921512	6.59297414	0.515097	2E-08	9E-08
MOSPD3	motile sperm domain containing 3	protein_coding	chr7:100612102-1	1696	5.213617846	8.684315555	0.653236	2E-08	9E-08
SALL2	spalt like transcription factor 2	protein_coding	chr14:21521080-2	5383	5.14846267	7.164832808	0.390132	2E-08	9E-08
ADGRL1	adhesion G protein-coupled receptor L1	protein_coding	chr19:14147743-1	8487	10.72123266	16.20630729	0.51323	2E-08	9E-08
POT1	protection of telomeres 1	protein_coding	chr7:124822386-1	8717	1.210548539	1.834512922	0.511955	2E-08	9E-08
NLE1	notchless homolog 1	protein_coding	chr17:35128730-3	5475	1.873524601	2.877037585	0.529686	2E-08	9E-08
CAMLG	calcium modulating ligand	protein_coding	chr5:134738548-1	2171	8.579015564	13.45942191	0.55746	2E-08	9E-08
PHF19	PHD finger protein 19	protein_coding	chr9:120855651-1	6594	3.644256849	5.29068539	0.454753	2E-08	9E-08
PCMTD2	protein-L-isoaspartate (D-aspartate) O-me	protein_coding	chr20:64255695-6	5993	12.86219735	17.85379518	0.387483	2E-08	9E-08

POLK	DNA polymerase kappa	protein_coding	chr5:75511756-75	9105	3.005624944	4.454883244	0.481503	2E-08	9E-08
ZDHHC2	zinc finger DHHC-type containing 2	protein_coding	chr8:17156482-17	6282	5.32087193	7.641043194	0.434183	2E-08	9E-08
ZNF418	zinc finger protein 418	protein_coding	chr19:57921884-5	4501	1.714049376	2.847173235	0.640275	2E-08	9E-08
ZNF496	zinc finger protein 496	protein_coding	chr1:247297412-2	10531	3.930781218	5.551824817	0.411439	2E-08	9E-08
KCNJ3	potassium voltage-gated channel subfami	protein_coding	chr2:154697855-1	4907	0.674565993	1.464047844	1.02581	2E-08	9E-08
METTL15	methyltransferase like 15	protein_coding	chr11:28108248-2	7778	2.690273551	3.829287195	0.423108	2E-08	1E-07
ZNF589	zinc finger protein 589	protein_coding	chr3:48241100-48	5521	1.887750804	3.469759005	0.792414	2E-08	1E-07
NTN3	netrin 3	protein_coding	chr16:2471499-24	1986	0.656766542	1.71727121	1.298206	2E-08	1E-07
PSMG4	proteasome assembly chaperone 4	protein_coding	chr6:3231403-330	10418	0.72100434	1.369079956	0.829645	2E-08	1E-07
LRP1	LDL receptor related protein 1	protein_coding	chr12:57128493-5	20839	5.984641281	10.36679568	0.713911	2E-08	1E-07
IKZF2	IKAROS family zinc finger 2	protein_coding	chr2:212999691-2	12240	0.915107233	1.487773301	0.609802	2E-08	1E-07
MID1IP1	MID1 interacting protein 1	protein_coding	chrX:38801432-38	3790	6.848278066	9.790425244	0.428606	2E-08	1E-07
SH3GLB2	SH3 domain containing GRB2 like, endopl	protein_coding	chr9:129007036-1	6814	12.73381635	17.02122073	0.331787	2E-08	1E-07
GTF3C3	general transcription factor IIIC subunit 3	protein_coding	chr2:196763035-1	7330	5.595548322	7.569984286	0.349096	2E-08	1E-07
ARF6	ADP ribosylation factor 6	protein_coding	chr14:49893082-4	3875	12.6912686	16.85372448	0.322493	2E-08	1E-07
RSU1	Ras suppressor protein 1	protein_coding	chr10:16590611-1	4126	17.25625381	23.04984816	0.331244	2E-08	1E-07
CROCC	ciliary rootlet coiled-coil, rootletin	protein_coding	chr1:16740273-16	8875	2.073021664	3.642342187	0.730048	2E-08	1E-07
PHKA1	phosphorylase kinase regulatory subunit a	protein_coding	chrX:72578814-72	6350	3.834741561	5.841436014	0.524457	2E-08	1E-07
PDE8A	phosphodiesterase 8A	protein_coding	chr15:84980440-8	8004	1.848119175	2.946392251	0.58993	2E-08	1E-07
RBM25	RNA binding motif protein 25	protein_coding	chr14:73058532-7	11764	12.32492616	16.41631539	0.327739	2E-08	1E-07
AC074117.1	novel transcript, antisense to GTF3C2 and	lncRNA	chr2:27356246-27	3412	3.055148357	5.019941649	0.628961	2E-08	1E-07
NAT8L	N-acetyltransferase 8 like	protein_coding	chr4:2059512-206	5871	5.145445885	7.987646339	0.549531	2E-08	1E-07
SYNC	syncoilin, intermediate filament protein	protein_coding	chr1:32680360-32	3487	3.887727183	6.268524152	0.600719	3E-08	1E-07
RYK	receptor like tyrosine kinase	protein_coding	chr3:134065303-1	8047	6.820230718	9.36884945	0.371525	3E-08	1E-07
LMAN1	lectin, mannose binding 1	protein_coding	chr18:59327823-5 6070		16.77875389	22.05890858	0.308717	3E-08	1E-07
MFAP1	microfibril associated protein 1	protein_coding	chr15:43804492-4	2261	12.86541199	19.06714246	0.482124	3E-08	1E-07
VIM-AS1	VIM antisense RNA 1	lncRNA	chr10:17214239-1	2008	0.79435825	1.928134241	1.195247	3E-08	1E-07
MICU3	mitochondrial calcium uptake family mer	protein_coding	chr8:17027238-17	4604	1.700686975	2.813637407	0.637899	3E-08	1E-07
CPT2	carnitine palmitoyltransferase 2	protein_coding	chr1:53196792-53	10089	6.31474168	8.46297985	0.335405	3E-08	1E-07
GAMT	guanidinoacetate N-methyltransferase	protein_coding	chr19:1397026-14	2403	57.54867014	80.50881891	0.399118	3E-08	1E-07
AC012313.9	novel transcript, antisense to SLC27A5	lncRNA	chr19:58500505-5	633	1.448751048	4.363940503	1.495226	3E-08	1E-07
PPIB	peptidylprolyl isomerase B	protein_coding	chr15:64155812-6	1911	73.98968193	104.0324479	0.406539	3E-08	1E-07
MSTO1	misato mitochondrial distribution and m	protein_coding	chr1:155610205-1	4208	2.120937894	3.251784326	0.529479	3E-08	1E-07
EPB41L4A-AS1	EPB41L4A antisense RNA 1	lncRNA	chr5:112160526-1	3903	3.608346921	5.731128721	0.576184	3E-08	1E-07
FGD5-AS1	FGD5 antisense RNA 1	lncRNA	chr3:14920347-14	5162	21.43138577	28.16675056	0.308294	3E-08	1E-07
C17orf67	chromosome 17 open reading frame 67	protein_coding	chr17:56791913-5	2434	0.315456102	1.279569878	1.920682	3E-08	1E-07
ERLEC1	endoplasmic reticulum lectin 1	protein_coding	chr2:53787044-53	2592	14.45739545	21.40976989	0.47836	3E-08	1E-07
CRNDE	colorectal neoplasia differentially express	lncRNA	chr16:54845189-5	14708	4.176073616	6.19762929	0.483721	3E-08	1E-07
AATF	apoptosis antagonizing transcription facto	protein_coding	chr17:36948954-3	4635	4.544349804	6.575163234	0.447117	3E-08	1E-07
SSH3	slingshot protein phosphatase 3	protein_coding	chr11:67303478-6	3611	3.091328199	4.919091817	0.582115	3E-08	1E-07
WIPF2	WAS/WASL interacting protein family mem	protein_coding	chr17:40219304-4	8123	5.420737086	7.320671294	0.347359	3E-08	1E-07
SEM1	SEM1 26S proteasome complex subunit	protein_coding	chr7:96481626-96	11490	2.380434252	3.512688864	0.473283	3E-08	1E-07
PITX2	paired like homeodomain 2	protein_coding	chr4:110617423-1	5787	1.297729291	2.113579975	0.61526	3E-08	1E-07
BAG4	BCL2 associated athanogene 4	protein_coding	chr8:38176533-38	4809	3.041571837	4.499979115	0.476459	3E-08	1E-07
TRUB2	TruB pseudouridine synthase family meml	protein_coding	chr9:128305159-1	5832	7.510796796	10.18777837	0.354386	3E-08	1E-07
CA5B	carbonic anhydrase 5B	protein_coding	chrX:15688830-15	8522	0.450515191	0.959141871	1.013988	3E-08	1E-07
ANO6	anoctamin 6	protein_coding	chr12:45215987-4	8235	10.08351281	14.42705646	0.431012	3E-08	1E-07
CWC15	CWC15 spliceosome associated protein ho	protein_coding	chr11:94962620-9	2734	7.073562104	11.90313898	0.673465	3E-08	1E-07
IFT74	intraflagellar transport 74	protein_coding	chr9:26947039-27	4073	5.162728038	7.75548438	0.497268	3E-08	1E-07
ZNF277	zinc finger protein 277	protein_coding	chr7:112206588-1	4014	3.950554616	5.627944506	0.424752	3E-08	1E-07
CNP	2',3'-cyclic nucleotide 3' phosphodiestera	protein_coding	chr17:41966763-4	7413	7.253268072	10.76295799	0.481975	3E-08	1E-07
ACSF2	acyl-CoA synthetase family member 2	protein_coding	chr17:50426158-5	5797	10.36998842	14.49463083	0.398421	3E-08	1E-07
SNIP1	Smad nuclear interacting protein 1	protein_coding	chr1:37534449-37	5718	2.022554596	3.086693232	0.524064	3E-08	1E-07
YKT6	YKT6 v-SNARE homolog	protein_coding	chr7:44200968-44	3707	32.70426632	43.23136587	0.315823	3E-08	1E-07
GYG2	glycogenin 2	protein_coding	chrX:2828822-288	3621	6.87371963	9.760268994	0.418297	3E-08	1E-07
PPP2R5E	protein phosphatase 2 regulatory subunit	protein_coding	chr14:63371364-6	9184	4.9759813	6.701631748	0.342556	3E-08	1E-07
ARF5	ADP ribosylation factor 5	protein_coding	chr7:127588386-1	1825	26.52247944	37.57056907	0.417172	3E-08	1E-07
BBS10	Bardet-Biedl syndrome 10	protein_coding	chr12:76344474-7	3568	5.38416902	7.828263602	0.455686	3E-08	1E-07
TSPAN14	tetraspanin 14	protein_coding	chr10:80454166-8	17889	0.897738386	1.444117354	0.599451	3E-08	1E-07
CYS1	cystin 1	protein_coding	chr2:10056780-10	2847	0.394285101	1.152565025	1.458626	3E-08	1E-07
IRAK1BP1	interleukin 1 receptor associated kinase 1	protein_coding	chr6:78867551-78	7463	0.812583323	1.347938544	0.648331	3E-08	1E-07
NUMBL	NUMB like endocytic adaptor protein	protein_coding	chr19:40665905-4	5823	2.110132096	3.072059312	0.455147	3E-08	1E-07
BCAM	basal cell adhesion molecule (Lutheran blo	protein_coding	chr19:44809059-4	5009	163.4906096	214.0908943	0.30258	3E-08	1E-07
CYP46A1	cytochrome P450 family 46 subfamily A m	protein_coding	chr14:99684304-9	9352	4.067515445	5.701071239	0.400918	3E-08	1E-07
ARHGEF2	Rho/Rac guanine nucleotide exchange fac	protein_coding	chr1:155946851-1	9155	13.63115264	18.39965968	0.346894	3E-08	1E-07
RPL22	ribosomal protein L22	protein_coding	chr1:6185020-620	4665	16.84921317	24.06026829	0.429271	3E-08	1E-07
ZFPL1	zinc finger protein like 1	protein_coding	chr11:65084210-6	3120	5.263115504	7.707748671	0.46194	3E-08	1E-07
TUBB	tubulin beta class I	protein_coding	chr6:30720352-30	3208	189.2075815	267.6655494	0.41483	3E-08	1E-07
BCL9L	BCL9 like	protein_coding	chr11:118893875- 10470		13.73142693	19.9905829	0.456484	3E-08	1E-07
ZDHHC20	zinc finger DHHC-type containing 20	protein_coding	chr13:21372573-2	5574	6.474003516	8.702022576	0.339745	3E-08	1E-07
RAB3D	RAB3D, member RAS oncogene family	protein_coding	chr19:11322068-1	4384	1.121554674	2.127264635	0.825398	3E-08	1E-07
PGAM2	phosphoglycerate mutase 2	protein_coding	chr7:44062727-44	837	178.1760991	287.5967665	0.609653	3E-08	1E-07
TMEM106A	transmembrane protein 106A	protein_coding	chr17:43211835-4	3577	0.422137644	1.031962708	1.19655	3E-08	1E-07
LINC01182	long intergenic non-protein coding RNA 1	lncRNA	chr4:13654374-14	6649	0.738896082	1.93037651	1.324945	3E-08	1E-07
LYRM1	LYR motif containing 1	protein_coding	chr16:20899868-2	3593	9.410396776	13.43843989	0.428976	4E-08	1E-07
JPT1	Jupiter microtubule associated homolog	protein_coding	chr17:75135248-7	3406	12.88284583	17.38627567	0.346096	4E-08	1E-07
MTHFR	methylenetetrahydrofolate reductase	protein_coding	chr1:11785723-11	11978	11.74938441	16.7608196	0.427865	4E-08	1E-07
PYCR1	pyrroline-5-carboxylate reductase 1	protein_coding	chr17:81932384-8	2886	22.50306023	29.86136746	0.3213	4E-08	1E-07
CACNB3	calcium voltage-gated channel auxiliary su	protein_coding	chr12:48813794-4	7153	0.782498476	1.403848936	0.753781	4E-08	1E-07
HYI	hydroxypyruvate isomerase (putative)	protein_coding	chr1:43451003-43	2740	5.123476591	8.472351767	0.640227	4E-08	1E-07
XXYLT1	xyloside xylosyltransferase 1	protein_coding	chr3:195068284-1	5396	6.46889256	9.270656766	0.432131	4E-08	1E-07
JADE2	jade family PHD finger 2	protein_coding	chr5:134524312-1	9877	0.566202875	1.076597815	0.837924	4E-08	1E-07
VAMP1	vesicle associated membrane protein 1	protein_coding	chr12:6462237-64	3580	2.00746904	3.325622963	0.644943	4E-08	1E-07
FBXO31	F-box protein 31	protein_coding	chr16:87326987-8	6910	5.618131932	7.825337053	0.392956	4E-08	1E-07
UXS1	UDP-glucuronate decarboxylase 1	protein_coding	chr2:106093303-1	7023	4.189488078	6.204464844	0.482565	4E-08	1E-07
COTL1	coactosin like F-actin binding protein 1	protein_coding	chr16:84565596-8	6761	0.432228328	0.915443839	0.988953	4E-08	1E-07
TMEM179	transmembrane protein 179	protein_coding	chr14:104474678-	5356	0.930061036	1.777802775	0.847743	4E-08	1E-07
DBI	diazepam binding inhibitor, acyl-CoA binc	protein_coding	chr2:119366921-1	2509	17.34376305	27.67011803	0.592969	4E-08	1E-07
VAMP4	vesicle associated membrane protein 4	protein_coding	chr1:171700160-1	4986	1.004869781	2.029833331	0.92284	4E-08	1E-07
ALDH9A1	aldehyde dehydrogenase 9 family membe	protein_coding	chr1:165662216-1	3308	21.16741924	27.94329974	0.313719	4E-08	1E-07
LDLRAD3	low density lipoprotein receptor class A d	protein_coding	chr11:35943981-3	4575	1.790647823	3.225588477	0.762972	4E-08	1E-07
KPNA1	karyopherin subunit alpha 1	protein_coding	chr3:122421902-1	8498	10.23089364	13.4937565	0.313141	4E-08	1E-07
C3orf38	chromosome 3 open reading frame 38	protein_coding	chr3:88149959-88	3259	4.923684736	7.772152829	0.575222	4E-08	1E-07
REXO4	REX4 homolog, 3'-5' exonuclease	protein_coding	chr9:133406059-1	2654	7.863314686	11.20924491	0.424313	4E-08	1E-07
PARM1	prostate androgen-regulated mucin-like p	protein_coding	chr4:74933095-75	5034	38.96664552	53.44437085	0.37009	4E-08	1E-07
IER2	immediate early response 2	protein_coding	chr19:13150411-1	3079	8.066740306	12.01786387	0.489421	4E-08	1E-07
SMARCAL1	SWI/SNF related, matrix associated, actin	protein_coding	chr2:216412414-2	5092	3.966455039	5.624873665	0.418846	4E-08	2E-07
SSBP2	single stranded DNA binding protein 2	protein_coding	chr5:81413021-81	11284	5.339698636	7.543598953	0.412221	4E-08	2E-07
CCDC47	coiled-coil domain containing 47	protein_coding	chr17:63745255-6	4354	12.20644307	16.30656603	0.331607	4E-08	2E-07
GRAMD1B	GRAM domain containing 1B	protein_coding	chr11:123358428-	14944	3.334133768	5.230340053	0.56712	4E-08	2E-07
HIST1H2BK	histone cluster 1 H2B family member k	protein_coding	chr6:27146418-27	381	29.84803285	49.07896892	0.629189	4E-08	2E-07
SINHCAF	SIN3-HDAC complex associated factor	protein_coding	chr12:31280584-3	5575	7.176117287	10.00995508	0.393311	4E-08	2E-07
MTMR9	myotubularin related protein 9	protein_coding	chr8:11284816-11	8404	8.48006049	11.12669731	0.305559	4E-08	2E-07
PRX	periaxin	protein_coding	chr19:40393768-4	5767	1.062288616	1.859957322	0.720157	4E-08	2E-07
ZNF407	zinc finger protein 407	protein_coding	chr18:74597870-7	12190	1.775372949	2.604196951	0.467605	4E-08	2E-07
PRRX1	paired related homeobox 1	protein_coding	chr1:170662728-1	8248	21.38060482	27.90871379	0.297962	4E-08	2E-07
USP12	ubiquitin specific peptidase 12	protein_coding	chr13:27066156-2	5096	4.827339448	6.905436803	0.429106	4E-08	2E-07
BCCIP	BRCA2 and CDKN1A interacting protein	protein_coding	chr10:125823546-	4496	7.28667212	10.45295802	0.436648	4E-08	2E-07
NUDT4	nudix hydrolase 4	protein_coding	chr12:93377883-9	10078	26.66495196	35.50584498	0.327372	4E-08	2E-07
ADAM19	ADAM metallopeptidase domain 19	protein_coding	chr5:157395534-1	7704	41.43725608	63.27074991	0.528666	4E-08	2E-07
RTTN	rotatin	protein_coding	chr18:70003031-7	19323	0.75912397	1.153798334	0.519766	4E-08	2E-07

AC139768.1	POU class 6 homeobox 1	protein_coding	chr12:51186936-5	6048	0.732292771	1.314962278	0.756155	4E-08	2E-07
EEPD1	endonuclease/exonuclease/phosphatase f	protein_coding	chr7:36153254-36	4737	0.925537639	1.637248402	0.735812	4E-08	2E-07
TMEM161B-AS1	TMEM161B antisense RNA 1	lncRNA	chr5:88268891-88	35171	1.128790123	1.573075788	0.394123	4E-08	2E-07
TTC27	tetratricopeptide repeat domain 27	protein_coding	chr2:32628032-32	3443	2.558949941	3.939961479	0.535883	4E-08	2E-07
MOB3A	MOB kinase activator 3A	protein_coding	chr19:2071036-20	4145	4.921948344	7.26703239	0.479253	4E-08	2E-07
RCOR2	REST corepressor 2	protein_coding	chr11:63911230-6	3049	12.85289407	18.98443642	0.478425	4E-08	2E-07
ZBTB26	zinc finger and BTB domain containing 26	protein_coding	chr9:122915566-1	4657	2.559636398	3.990504937	0.551783	4E-08	2E-07
SARM1	sterile alpha and TIR motif containing 1	protein_coding	chr17:28364356-2	11105	1.086837298	1.804766887	0.648771	4E-08	2E-07
GTPBP10	GTP binding protein 10	protein_coding	chr7:90335223-90	10436	1.64293524	2.391894692	0.455221	4E-08	2E-07
EXD2	exonuclease 3'-5' domain containing 2	protein_coding	chr14:69191511-6	6263	5.64692652	7.714633783	0.364688	4E-08	2E-07
MAP3K5	mitogen-activated protein kinase kinase k	protein_coding	chr6:136557046-1	5263	2.422240691	3.616695409	0.491	4E-08	2E-07
EEF1AKMT1	EEF1A lysine methyltransferase 1	protein_coding	chr13:20728731-2	1339	2.884400011	5.361934404	0.805933	4E-08	2E-07
BRK1	BRICK1 subunit of SCAR/WAVE actin nucle	protein_coding	chr3:10115675-10	1150	101.3846884	138.3826572	0.362484	4E-08	2E-07
SLC30A3	solute carrier family 30 member 3	protein_coding	chr2:27253684-27	5978	19.38050224	25.13698188	0.288687	4E-08	2E-07
BEX2	brain expressed X-linked 2	protein_coding	chrX:103309346-1	1176	6.713954687	12.08064559	0.753334	4E-08	2E-07
MAPK6	mitogen-activated protein kinase 6	protein_coding	chr15:51952106-5	6373	9.999050274	13.2889078	0.32345	4E-08	2E-07
SGCB	sarcoglycan beta	protein_coding	chr4:52020706-52	4541	29.57783455	39.43342363	0.328477	4E-08	2E-07
STRN3	striatin 3	protein_coding	chr14:30893799-3	4965	11.2799315	15.06902836	0.332153	4E-08	2E-07
CELSR1	cadherin EGF LAG seven-pass G-type recep	protein_coding	chr22:46360834-4	12806	13.61757218	22.30757831	0.632325	4E-08	2E-07
NPNT	nephronectin	protein_coding	chr4:105894775-1	6117	34.5056228	45.23250526	0.303807	4E-08	2E-07
SMARCB1	SWI/SNF related, matrix associated, actin	protein_coding	chr22:23786931-2	16525	3.42072029	4.761725767	0.392494	4E-08	2E-07
SIMC1	SUMO interacting motifs containing 1	protein_coding	chr5:176238367-1	5536	1.826704129	2.888443192	0.569311	4E-08	2E-07
HNRNPU	heterogeneous nuclear ribonucleoprotein	protein_coding	chr1:244840638-2	22512	20.6668899	27.24554201	0.312646	5E-08	2E-07
HNRNPF	heterogeneous nuclear ribonucleoprotein	protein_coding	chr10:43385617-4	3253	40.65583965	52.67415991	0.286987	5E-08	2E-07
SBF1	SET binding factor 1	protein_coding	chr22:50445000-5	9452	17.56898686	24.81524235	0.414021	5E-08	2E-07
SBF2	SET binding factor 2	protein_coding	chr11:9778667-10	16767	3.548563491	4.80491217	0.350698	5E-08	2E-07
SCRN2	secernin 2	protein_coding	chr17:47837692-4	2999	10.82347804	15.60126238	0.439262	5E-08	2E-07
MAN2A1	mannosidase alpha class 2A member 1	protein_coding	chr5:109689366-1	11815	2.327586703	3.235976761	0.391741	5E-08	2E-07
SH3BGRL3	SH3 domain binding glutamate rich prote	protein_coding	chr1:26280086-26	876	17.59861285	28.37995832	0.6078	5E-08	2E-07
RP2	RP2 activator of ARL3 GTPase	protein_coding	chrX:46837043-46	3700	1.582655053	2.745337217	0.705482	5E-08	2E-07
HSP90AB1	heat shock protein 90 alpha family class B	protein_coding	chr6:44246166-44	2980	276.4176118	370.4653048	0.336957	5E-08	2E-07
C14orf132	chromosome 14 open reading frame 132	protein_coding	chr14:96039324-9	7775	1.479826083	2.512121124	0.676607	5E-08	2E-07
IFT52	intraflagellar transport 52	protein_coding	chr20:43590931-4	2542	3.11013995	4.797541637	0.535737	5E-08	2E-07
AC126468.1	novel transcript	lncRNA	chr12:20104590-2	483	10.39959118	19.02619091	0.782201	5E-08	2E-07
RPIA	ribose 5-phosphate isomerase A	protein_coding	chr2:88691673-88	1813	4.111029066	6.734463043	0.619761	5E-08	2E-07
USP28	ubiquitin specific peptidase 28	protein_coding	chr11:113797874-	5719	36.98020372	48.70829776	0.311453	5E-08	2E-07
IDE	insulin degrading enzyme	protein_coding	chr10:92451684-9	7607	10.62824718	13.88422642	0.299265	5E-08	2E-07
ASCC3	activating signal cointegrator 1 complex s	protein_coding	chr6:100508194-1	11731	8.420459924	11.17912738	0.323193	5E-08	2E-07
VPS41	VPS41 subunit of HOPS complex	protein_coding	chr7:38722974-38	7752	6.557405443	8.671470172	0.315724	5E-08	2E-07
ABHD15	abhydrolase domain containing 15	protein_coding	chr17:29560547-2	3492	3.098616557	5.108214853	0.633867	5E-08	2E-07
VLDLR	very low density lipoprotein receptor	protein_coding	chr9:2621787-266	9389	9.617835148	12.94336444	0.342778	5E-08	2E-07
MTHFD2L	methylenetetrahydrofolate dehydrogenas	protein_coding	chr4:74114174-74	9819	1.631896273	2.305306705	0.412156	5E-08	2E-07
KCNJ5	potassium voltage-gated channel subfami	protein_coding	chr11:128891356-	6237	15.19443906	21.79988932	0.436208	5E-08	2E-07
BPHL	biphenyl hydrolase like	protein_coding	chr6:3118374-315	5780	2.918967981	4.114988146	0.410653	5E-08	2E-07
EIF4G2	eukaryotic translation initiation factor 4 g	protein_coding	chr11:10797050-1	8485	114.7702036	149.3333641	0.293419	5E-08	2E-07
DOP1B	DOP1 leucine zipper like protein B	protein_coding	chr21:36156782-3 9030		0.502093634	0.960437478	0.860383	5E-08	2E-07
ZNF84	zinc finger protein 84	protein_coding	chr12:133037292-	9520	4.376599401	5.790603007	0.318339	5E-08	2E-07
JAK2	Janus kinase 2	protein_coding	chr9:4984390-512	6170	18.27227404	25.69078369	0.40575	5E-08	2E-07
TMEM206	transmembrane protein 206	protein_coding	chr1:212363931-2	2914	0.934715993	1.936012466	0.978231	5E-08	2E-07
SH3GL2	SH3 domain containing GRB2 like 2, endo	protein_coding	chr9:17579066-17	2693	0.721666144	1.601502762	1.056998	5E-08	2E-07
TMA16	translation machinery associated 16 hom	protein_coding	chr4:163494442-1	3406	2.819492799	4.534088757	0.602563	5E-08	2E-07
RPSA	ribosomal protein SA	protein_coding	chr3:39406716-39	3909	60.19139417	84.24555728	0.399344	5E-08	2E-07
GPSM1	G protein signaling modulator 1	protein_coding	chr9:136327476-1	5138	15.52869039	20.95494664	0.346383	5E-08	2E-07
SND1	staphylococcal nuclease and tudor domai	protein_coding	chr7:127652194-1	6486	27.3776441	35.37574574	0.283428	5E-08	2E-07
UBTD2	ubiquitin domain containing 2	protein_coding	chr5:172209646-1	2988	12.22192249	16.24980921	0.324816	5E-08	2E-07
ADAMTS6	ADAM metallopeptidase with thrombospo	protein_coding	chr5:65148738-65	8565	1.760179538	2.555227312	0.450652	5E-08	2E-07
PLPP6	phospholipid phosphatase 6	protein_coding	chr9:4662315-466	2944	2.176836491	3.792798454	0.709672	5E-08	2E-07
HEATR1	HEAT repeat containing 1	protein_coding	chr1:236549005-2	9061	3.378455692	4.710316275	0.393953	5E-08	2E-07
VPS37C	VPS37C subunit of ESCRT-I	protein_coding	chr11:61130257-6	3957	6.817649732	9.563467601	0.402291	5E-08	2E-07
AP000866.1	novel transcript, antisense to C11orf61	lncRNA	chr11:124800424-	10457	0.584908973	1.036552044	0.735937	5E-08	2E-07
LPIN2	lipin 2	protein_coding	chr18:2916994-30	6800	3.880854554	5.472610692	0.409344	5E-08	2E-07
KANK1	KN motif and ankyrin repeat domains 1	protein_coding	chr9:470291-7461	11908	4.392921457	5.803238795	0.315442	5E-08	2E-07
OTULIN	OTU deubiquitinase with linear linkage sp	protein_coding	chr5:14664664-14	8614	2.311029397	3.350798876	0.447411	5E-08	2E-07
PGGHG	protein-glucosylgalactosylhydroxylysine g	protein_coding	chr11:289135-296	5260	1.961501249	3.589239842	0.78602	5E-08	2E-07
GALK1	galactokinase 1	protein_coding	chr17:75751594-7	2891	3.970368849	6.14755016	0.5393	5E-08	2E-07
ZNF281	zinc finger protein 281	protein_coding	chr1:200404940-2	4895	6.232317827	8.569987309	0.373869	5E-08	2E-07
TDP1	tyrosyl-DNA phosphodiesterase 1	protein_coding	chr14:89954939-9	6227	1.273138234	1.933011007	0.515313	5E-08	2E-07
GRIP1	glutamate receptor interacting protein 1	protein_coding	chr12:66347431-6	6570	1.483976273	2.336416738	0.570561	5E-08	2E-07
PCDHB10	protocadherin beta 10	protein_coding	chr5:141192353-1	3290	1.347984646	2.374810437	0.731674	5E-08	2E-07
UNC5B	unc-5 netrin receptor B	protein_coding	chr10:71212570-7	6841	21.00655252	27.97632878	0.327812	6E-08	2E-07
DBR1	debranching RNA lariats 1	protein_coding	chr3:138161012-1	3160	2.335639204	3.674253489	0.564901	6E-08	2E-07
AGA	aspartylglucosaminidase	protein_coding	chr4:177430774-1	2605	0.613769176	1.500065708	1.192217	6E-08	2E-07
LYNX1	Ly6/neurotoxin 1	protein_coding	chr8:142771197-1	5022	2.849955632	4.415261038	0.544829	6E-08	2E-07
CARF	calcium responsive transcription factor	protein_coding	chr2:202912214-2	10112	1.939083955	2.788421537	0.435844	6E-08	2E-07
PSME4	proteasome activator subunit 4	protein_coding	chr2:53864069-53	10392	9.483328875	12.43241897	0.304414	6E-08	2E-07
REEP1	receptor accessory protein 1	protein_coding	chr2:86213993-86	5846	19.3585341	25.06273107	0.286043	6E-08	2E-07
TBCB	tubulin folding cofactor B	protein_coding	chr19:36114289-3	3373	15.24151202	20.96999049	0.374315	6E-08	2E-07
LPCAT1	lysophosphatidylcholine acyltransferase 1	protein_coding	chr5:1456480-152	6475	2.100006404	3.275161678	0.558564	6E-08	2E-07
CLTC	clathrin heavy chain	protein_coding	chr17:59619689-5	11836	45.82371442	59.6241836	0.293523	6E-08	2E-07
ZDHHC24	zinc finger DHHC-type containing 24	protein_coding	chr11:66520637-6	6535	2.780128825	4.208972745	0.507214	6E-08	2E-07
RAP2C	RAP2C, member of RAS oncogene family	protein_coding	chrX:132203024-1	4118	10.74026647	14.32414501	0.330122	6E-08	2E-07
AL441992.1	novel transcript	lncRNA	chr9:128724445-1	544	1.099183256	5.109599054	2.108231	6E-08	2E-07
BTBD10	BTB domain containing 10	protein_coding	chr11:13388001-1	3495	6.680094867	9.077954023	0.355698	6E-08	2E-07
SMG9	SMG9 nonsense mediated mRNA decay fac	protein_coding	chr19:43727983-4	7908	2.982971235	4.209320139	0.411408	6E-08	2E-07
SLC26A11	solute carrier family 26 member 11	protein_coding	chr17:80219699-8	4903	3.344998338	4.945714742	0.475555	6E-08	2E-07
EIF2AK2	eukaryotic translation initiation factor 2 a	protein_coding	chr2:37099210-37	10753	4.774193103	6.788438923	0.420247	6E-08	2E-07
PDIA5	protein disulfide isomerase family A mem	protein_coding	chr3:123067025-1	4067	0.966162433	1.734311124	0.759472	6E-08	2E-07
AC093673.1	novel transcript	lncRNA	chr7:143379692-1	648	5.561969318	9.979198006	0.758592	6E-08	2E-07
ZNF518A	zinc finger protein 518A	protein_coding	chr10:96129715-9	10891	3.375293037	4.729843931	0.399516	6E-08	2E-07
DENND2C	DENN domain containing 2C	protein_coding	chr1:114582848-1	7413	0.474651777	0.948130379	0.908464	6E-08	2E-07
SH3RF2	SH3 domain containing ring finger 2	protein_coding	chr5:145936578-1	7209	2.416385856	3.444470427	0.424034	6E-08	2E-07
IBA57	iron-sulfur cluster assembly factor IBA57	protein_coding	chr1:228165804-2 10230		2.00857318	3.277617262	0.624997	6E-08	2E-07
ERF	ETS2 repressor factor	protein_coding	chr19:42247569-4	3269	20.41047686	26.57133614	0.294602	6E-08	2E-07
NTAN1	N-terminal asparagine amidase	protein_coding	chr16:15037854-1	3539	4.163364834	6.536873513	0.568288	7E-08	2E-07
ATAT1	alpha tubulin acetyltransferase 1	protein_coding	chr6:30626842-30	4035	2.563904905	3.779829189	0.474612	7E-08	2E-07
API5	apoptosis inhibitor 5	protein_coding	chr11:43311963-4	4651	22.73497779	29.46951643	0.287884	7E-08	2E-07
PUS10	pseudouridine synthase 10	protein_coding	chr2:60940222-61	7651	0.587324555	0.991235214	0.665095	7E-08	2E-07
TMSB10	thymosin beta 10	protein_coding	chr2:84905656-84	461	322.420337	521.0212959	0.611728	7E-08	2E-07
SH3YL1	SH3 and SYLF domain containing 1	protein_coding	chr2:217730-2663	11165	2.296317136	3.193293389	0.391068	7E-08	2E-07
HYAL2	hyaluronidase 2	protein_coding	chr3:50317790-50	4421	6.017477442	8.586745304	0.423957	7E-08	2E-07
GSTA4	glutathione S-transferase alpha 4	protein_coding	chr6:52977948-52	2101	10.14883946	15.73142609	0.544652	7E-08	2E-07
CCDC90B	coiled-coil domain containing 90B	protein_coding	chr11:83259097-8	6561	4.809178632	6.836468368	0.4217	7E-08	2E-07
SPATA18	spermatogenesis associated 18	protein_coding	chr4:52051304-52	4975	4.821614685	6.577300099	0.36054	7E-08	2E-07
ARAP3	ArfGAP with RhoGAP domain, ankyrin rep	protein_coding	chr5:141653401-1	6195	3.150818144	4.991062752	0.580496	7E-08	2E-07
RNF215	ring finger protein 215	protein_coding	chr22:30368811-3	10624	1.192926472	1.918512922	0.596244	7E-08	3E-07
TRAPPC4	trafficking protein particle complex 4	protein_coding	chr11:119018432-	5140	4.253789281	6.071086094	0.425371	7E-08	3E-07
BTBD2	BTB domain containing 2	protein_coding	chr19:1985438-20	5944	18.56307258	24.64570086	0.322752	7E-08	3E-07
ZBTB7B	zinc finger and BTB domain containing 7B	protein_coding	chr1:155002630-1	4303	10.17002579	13.77053275	0.352594	7E-08	3E-07
WDR36	WD repeat domain 36	protein_coding	chr5:111091716-1	7656	3.566869521	4.822105118	0.34746	7E-08	3E-07
CSGALNACT2	chondroitin sulfate N-acetylgalactosamin	protein_coding	chr10:43138445-4	3765	3.783234045	5.491184241	0.447367	7E-08	3E-07
RGS12	regulator of G protein signaling 12	protein_coding	chr4:3293028-343	13755	1.775912008	2.659476791	0.499615	7E-08	3E-07

SMAD5	SMAD family member 5	protein_coding	chr5:136132845-1 9040		5.439643385	7.432179789	0.365086	7E-08	3E-07
HSPB11	heat shock protein family B (small) membe	protein_coding	chr1:53916574-53	3236	1.728151018	2.887312934	0.656239	7E-08	3E-07
YIPF2	Yip1 domain family member 2	protein_coding	chr19:10922185-1	2518	10.30024029	14.59482709	0.416064	7E-08	3E-07
DDX60	DExD/H-box helicase 60	protein_coding	chr4:168216294-1	6746	1.797285523	2.781364449	0.544229	7E-08	3E-07
TMBIM4	transmembrane BAX inhibitor motif conta	protein_coding	chr12:66135846-6	4137	1.416308497	2.340541331	0.634594	8E-08	3E-07
TMTC3	transmembrane and tetratricopeptide rep	protein_coding	chr12:88142296-8	7327	4.020319677	5.424945828	0.346101	8E-08	3E-07
IFT43	intraflagellar transport 43	protein_coding	chr14:75902136-7	4172	3.154714911	4.588749846	0.45034	8E-08	3E-07
BTBD9	BTB domain containing 9	protein_coding	chr6:38168451-38	9537	5.516280907	7.844037354	0.423806	8E-08	3E-07
POLE3	DNA polymerase epsilon 3, accessory subu	protein_coding	chr9:113407235-1	2607	12.53980252	16.88721486	0.344359	8E-08	3E-07
LIX1L	limb and CNS expressed 1 like	protein_coding	chr1:145933423-1	3991	10.33062841	14.15358624	0.366857	8E-08	3E-07
PLCXD1	phosphatidylinositol specific phospholipa	protein_coding	chrX:276322-3033	6065	2.89072239	4.239312704	0.467189	8E-08	3E-07
UBE2W	ubiquitin conjugating enzyme E2 W	protein_coding	chr8:73780097-73 9090		2.508834022	3.700330047	0.471876	8E-08	3E-07
DMAC2L	distal membrane arm assembly complex 2	protein_coding	chr14:50312326-5	11297	2.74579093	3.645697754	0.321917	8E-08	3E-07
PUM2	pumilio RNA binding family member 2	protein_coding	chr2:20248691-20	6978	14.46776559	18.92850733	0.301909	8E-08	3E-07
NFYA	nuclear transcription factor Y subunit alp	protein_coding	chr6:41072945-41	3811	4.152406471	5.993322703	0.441313	8E-08	3E-07
CARNMT1	carnosine N-methyltransferase 1	protein_coding	chr9:74980790-75	4840	8.474122182	11.40519401	0.343031	8E-08	3E-07
RECQL	RecQ like helicase	protein_coding	chr12:21468910-2	3779	2.133676018	3.40292861	0.587895	8E-08	3E-07
NQO1	NAD(P)H quinone dehydrogenase 1	protein_coding	chr16:69706996-6	2971	0.670356668	1.925335496	1.422483	8E-08	3E-07
PHF20L1	PHD finger protein 20 like 1	protein_coding	chr8:132775358-1	20571	7.701900968	9.990685777	0.289087	8E-08	3E-07
KIF21A	kinesin family member 21A	protein_coding	chr12:39293228-3	10117	13.53892704	17.3313745	0.269821	8E-08	3E-07
VPS72	vacuolar protein sorting 72 homolog	protein_coding	chr1:151169987-1	4545	5.462179631	8.243446403	0.508894	8E-08	3E-07
BCL7A	BAF chromatin remodeling complex subu	protein_coding	chr12:122019422-	6565	4.503436566	6.648205407	0.477769	8E-08	3E-07
ABCC4	ATP binding cassette subfamily C member	protein_coding	chr13:95019835-9	8629	1.038039076	1.68417848	0.610917	8E-08	3E-07
SRPK1	SRSF protein kinase 1	protein_coding	chr6:35832966-35	6692	5.977428834	7.969283349	0.329157	8E-08	3E-07
SWAP70	switching B cell complex subunit SWAP70	protein_coding	chr11:9664077-97	7192	3.901350869	5.229677981	0.336406	8E-08	3E-07
CDC42BPB	CDC42 binding protein kinase beta	protein_coding	chr14:102932380-	8095	20.47821115	28.21895157	0.377387	8E-08	3E-07
RTF1	RTF1 homolog, Paf1/RNA polymerase II cc	protein_coding	chr15:41408408-4	5770	12.75105355	16.68647588	0.301906	9E-08	3E-07
ACTL6A	actin like 6A	protein_coding	chr3:179562886-1	3668	5.961419841	8.338095616	0.39989	9E-08	3E-07
F11R	F11 receptor	protein_coding	chr1:160995211-1	5061	3.512215656	5.164052628	0.466057	9E-08	3E-07
PSMA4	proteasome subunit alpha 4	protein_coding	chr15:78540405-7	6994	8.9495791	12.49591471	0.397266	9E-08	3E-07
AP001372.2	uncharacterized LOC100287896	lncRNA	chr11:74493366-7	2493	1.338974191	2.517488787	0.818807	9E-08	3E-07
TSN	translin	protein_coding	chr2:121737103-1	4395	14.27202288	19.1665953	0.339874	9E-08	3E-07
SURF6	surfeit 6	protein_coding	chr9:133328776-1	4414	9.412509616	12.74422261	0.350367	9E-08	3E-07
AC010980.1	novel transcript	lncRNA	chr2:222317242-2	1412	9.960472038	14.91677285	0.498568	9E-08	3E-07
CLUAP1	clusterin associated protein 1	protein_coding	chr16:3500976-35	7746	3.023219258	4.103794983	0.354786	9E-08	3E-07
ERMAP	erythroblast membrane associated protei	protein_coding	chr1:42817122-42	5720	3.168812416	4.322134345	0.361362	9E-08	3E-07
PYURF	PIGY upstream reading frame	protein_coding	chr4:88520998-88	1311	27.88815294	39.93206199	0.433907	9E-08	3E-07
CIRBP	cold inducible RNA binding protein	protein_coding	chr19:1259384-12	6368	35.81589663	46.67053741	0.296001	9E-08	3E-07
KPNA4	karyopherin subunit alpha 4	protein_coding	chr3:160495007-1	9384	14.02386089	17.97119142	0.271406	9E-08	3E-07
IFIT5	interferon induced protein with tetratricc	protein_coding	chr10:89414568-8	4029	1.952467977	3.052432411	0.554652	9E-08	3E-07
TGFBR3	transforming growth factor beta receptor	protein_coding	chr1:91680343-91	8577	2.99220382	4.150569015	0.384995	9E-08	3E-07
TPD52L2	TPD52 like 2	protein_coding	chr20:63865228-6	4694	29.09714924	37.82972494	0.292122	9E-08	3E-07
NBR1	NBR1 autophagy cargo receptor	protein_coding	chr17:43170481-4	8405	12.68421117	16.38763376	0.283442	9E-08	3E-07
ZBTB12	zinc finger and BTB domain containing 12	protein_coding	chr6:31899607-31	1875	5.541699811	9.040272392	0.626477	9E-08	3E-07
SMIM8	small integral membrane protein 8	protein_coding	chr6:87322583-87	8750	2.334013296	3.255338733	0.39142	1E-07	3E-07
RRS1	ribosome biogenesis regulator 1 homolog	protein_coding	chr8:66429014-66	1720	10.45260535	16.93059929	0.612204	1E-07	3E-07
TMEM101	transmembrane protein 101	protein_coding	chr17:44011188-4	3029	6.704066315	9.665525634	0.443013	1E-07	3E-07
NSUN2	NOP2/Sun RNA methyltransferase 2	protein_coding	chr5:6599239-663	5506	9.178304458	12.05559635	0.306046	1E-07	3E-07
AC092164.1	novel transcript	lncRNA	chr2:28707511-28	6346	0.500272132	1.069654344	1.00932	1E-07	3E-07
DUSP10	dual specificity phosphatase 10	protein_coding	chr1:221701424-2	2827	1.344755122	2.393079836	0.737108	1E-07	3E-07
AXIN1	axin 1	protein_coding	chr16:287440-352	7537	3.138174363	4.287880216	0.362983	1E-07	3E-07
UBP1	upstream binding protein 1	protein_coding	chr3:33388336-33	9760	8.942577681	11.69445316	0.301132	1E-07	3E-07
TMEM200C	transmembrane protein 200C	protein_coding	chr18:5882072-58	10638	1.586131739	2.604180132	0.629794	1E-07	3E-07
PITPNM3	PITPNM family member 3	protein_coding	chr17:6451263-65	10671	0.750937831	1.23894137	0.636255	1E-07	3E-07
TTC8	tetratricopeptide repeat domain 8	protein_coding	chr14:88824153-8	6257	2.883537836	4.162336826	0.446579	1E-07	3E-07
NDUFAF8	NADH:ubiquinone oxidoreductase compl	protein_coding	chr17:81239305-8	1339	29.77887611	44.64613196	0.49634	1E-07	4E-07
NTHL1	nth like DNA glycosylase 1	protein_coding	chr16:2039815-20	2430	11.43865654	16.56917914	0.448237	1E-07	4E-07
KCNH2	potassium voltage-gated channel subfami	protein_coding	chr7:150944961-1	5877	54.81294304	73.09979511	0.330007	1E-07	4E-07
CHMP6	charged multivesicular body protein 6	protein_coding	chr17:80991598-8	2118	11.25635257	16.07705372	0.426882	1E-07	4E-07
LINC01963	long intergenic non-protein coding RNA 1	lncRNA	chr2:216217045-2	3148	3.060520993	4.77579408	0.553904	1E-07	4E-07
SYDE2	synapse defective Rho GTPase homolog 2	protein_coding	chr1:85156889-85	6405	7.182939712	10.59582515	0.476438	1E-07	4E-07
MYO9A	myosin IXA	protein_coding	chr15:71822291-7 20320		3.276733091	4.729863752	0.446191	1E-07	4E-07
PPIL4	peptidylprolyl isomerase like 4	protein_coding	chr6:149504733-1	2646	9.860264971	13.942626	0.411646	1E-07	4E-07
ASL	argininosuccinate lyase	protein_coding	chr7:66075798-66	3195	1.533442628	2.670801596	0.722606	1E-07	4E-07
WDR44	WD repeat domain 44	protein_coding	chrX:118346073-1	4202	6.064954646	8.274701479	0.360013	1E-07	4E-07
CENPX	centromere protein X	protein_coding	chr17:82018702-8	1531	9.490754504	13.96616502	0.472063	1E-07	4E-07
GULP1	GULP PTB domain containing engulfment	protein_coding	chr2:188291669-1	11979	1.510859804	2.125121483	0.405541	1E-07	4E-07
MAP4K3	mitogen-activated protein kinase kinase k	protein_coding	chr2:39249266-39	6056	11.39818492	14.75487223	0.286308	1E-07	4E-07
ALDH6A1	aldehyde dehydrogenase 6 family membe	protein_coding	chr14:74056850-7	6680	9.34915729	12.19333129	0.296965	1E-07	4E-07
EIF4B	eukaryotic translation initiation factor 4E	protein_coding	chr12:53006158-5	6146	62.6724227	82.59604969	0.31286	1E-07	4E-07
LINC00294	long intergenic non-protein coding RNA 2	lncRNA	chr11:33076149-3	3306	1.2233883	2.41530707	0.898726	1E-07	4E-07
MTMR4	myotubularin related protein 4	protein_coding	chr17:58489529-5	7475	6.292165578	8.910405193	0.417006	1E-07	4E-07
STIP1	stress induced phosphoprotein 1	protein_coding	chr11:64185272-6	4657	34.15968683	45.54559735	0.329106	1E-07	4E-07
ZFYVE21	zinc finger FYVE-type containing 21	protein_coding	chr14:103715730-	8390	7.534116835	10.15194822	0.342966	1E-07	4E-07
PTENP1-AS	PTENP1 antisense RNA	lncRNA	chr9:33676785-33	4823	0.603772093	1.180972679	0.876301	1E-07	4E-07
AL031985.3	novel transcript, sense overlapping with Z	lncRNA	chr1:40464319-40	2449	0.417816262	1.086223354	1.295188	1E-07	4E-07
PPP2R1A	protein phosphatase 2 scaffold subunit Aa	protein_coding	chr19:52190048-5	7413	54.11845355	70.21064024	0.289467	1E-07	4E-07
AC012146.1	novel transcript, antisense to ZNF232	lncRNA	chr17:5111468-51	2006	1.253044569	2.374108891	0.831769	1E-07	4E-07
SPATS2L	spermatogenesis associated serine rich 2 l	protein_coding	chr2:200305881-2	10453	3.856505833	5.572683577	0.446337	1E-07	4E-07
SLC20A2	solute carrier family 20 member 2	protein_coding	chr8:42416475-42	6424	27.62925528	36.70422583	0.324381	1E-07	4E-07
TMEM99	transmembrane protein 99	protein_coding	chr17:40819106-4	3057	1.915524705	3.159983955	0.632818	1E-07	4E-07
NBPF1	NBPF member 1	protein_coding	chr1:16562319-16	12212	2.796492974	3.92840761	0.404876	1E-07	4E-07
ANXA7	annexin A7	protein_coding	chr10:73375101-7	2862	24.42817039	32.97297989	0.347347	1E-07	4E-07
TRIM52	tripartite motif containing 52	protein_coding	chr5:181254417-1	6562	2.575490166	3.817450576	0.483004	1E-07	4E-07
ZNF664	zinc finger protein 664	protein_coding	chr12:123971845-	6340	34.17322615	44.02112599	0.279004	1E-07	4E-07
MRPL35	mitochondrial ribosomal protein L35	protein_coding	chr2:86199355-86	3825	15.16505421	20.88782314	0.377742	1E-07	4E-07
EPB41	erythrocyte membrane protein band 4.1	protein_coding	chr1:28887091-29	19993	2.343922336	3.132059716	0.331494	1E-07	4E-07
C12orf49	chromosome 12 open reading frame 49	protein_coding	chr12:116710171-	8678	3.581809031	4.98197685	0.389961	1E-07	4E-07
VPS9D1-AS1	VPS9D1 antisense RNA 1	lncRNA	chr16:89711856-8	1753	0.405655262	1.071381069	1.296006	1E-07	4E-07
ING4	inhibitor of growth family member 4	protein_coding	chr12:6650301-66	2709	8.696091066	12.42739955	0.427666	1E-07	4E-07
MEMO1	mediator of cell motility 1	protein_coding	chr2:31865060-32	5220	2.613767388	3.952893902	0.507503	1E-07	4E-07
DUBR	DPPA2 upstream binding RNA	lncRNA	chr3:107220744-1	15322	0.752863124	1.101059918	0.461468	1E-07	4E-07
AC139887.2	novel transcript, antisense to PCGF3	lncRNA	chr4:760202-7818	4671	1.544557535	2.623393947	0.674814	1E-07	4E-07
LRP12	LDL receptor related protein 12	protein_coding	chr8:104489231-1	9777	2.758172398	3.707556884	0.339073	1E-07	4E-07
FBXO33	F-box protein 33	protein_coding	chr14:39397669-3	3853	4.622166619	6.780417846	0.471593	1E-07	4E-07
CFAP410	cilia and flagella associated protein 410	protein_coding	chr21:44328944-4	6063	3.186485095	4.500140112	0.412091	1E-07	4E-07
GLIPR2	GLI pathogenesis related 2	protein_coding	chr9:36136536-36	3055	2.268252606	3.746735997	0.634408	1E-07	4E-07
GUCY1A1	guanylate cyclase 1 soluble subunit alpha	protein_coding	chr4:155666711-1	5766	20.02340135	29.29846906	0.466561	1E-07	4E-07
AL161772.1	novel transcript, antisense to IL17D	lncRNA	chr13:20699307-2	4412	1.608253935	2.499427749	0.549898	1E-07	4E-07
AL355574.1	novel transcript	lncRNA	chr9:135907812-1	839	2.967732577	6.053939276	0.944998	1E-07	4E-07
THOC1	THO complex 1	protein_coding	chr18:214520-268	8102	2.326959186	3.268075707	0.406977	1E-07	4E-07
DIS3	DIS3 homolog, exosome endoribonucleas	protein_coding	chr13:72752169-7	10835	3.778800428	5.119662535	0.351349	1E-07	4E-07
WDR5	WD repeat domain 5	protein_coding	chr9:134135365-1	3629	6.807830167	9.184814683	0.34796	1E-07	4E-07
ARL10	ADP ribosylation factor like GTPase 10	protein_coding	chr5:176365487-1	11440	2.763173066	3.883368666	0.405772	1E-07	4E-07
ATIC	5-aminoimidazole-4-carboxamide ribonu	protein_coding	chr2:215311956-2	3789	13.00768071	17.82560069	0.368213	1E-07	4E-07
ZDHHC13	zinc finger DHHC-type containing 13	protein_coding	chr11:19117099-1	7759	1.568963073	2.295257067	0.462418	1E-07	4E-07
TMOD1	tropomodulin 1	protein_coding	chr9:97501180-97	3676	120.5257004	154.0877973	0.268162	1E-07	5E-07
PKD2	polycystin 2, transient receptor potential	protein_coding	chr4:88007635-88	5531	12.00143009	16.10963423	0.339329	1E-07	5E-07
LINC01405	long intergenic non-protein coding RNA 1	lncRNA	chr12:110934590-	5646	22.20427101	29.13380605	0.30547	1E-07	5E-07
PAWR	pro-apoptotic WT1 regulator	protein_coding	chr12:79574979-7	11530	5.546997811	7.256757528	0.300166	1E-07	5E-07
TMTC4	transmembrane and tetratricopeptide rep	protein_coding	chr13:100603927-	6272	3.917281691	5.285100665	0.344935	1E-07	5E-07

LRRC57	leucine rich repeat containing 57	protein_coding	chr15:42537820-4	8114	2.260763279	3.099087305	0.368744	1E-07	5E-07
SRSF9	serine and arginine rich splicing factor 9	protein_coding	chr12:120461672-	3695	9.543732231	14.08673956	0.47448	1E-07	5E-07
C12orf4	chromosome 12 open reading frame 4	protein_coding	chr12:4487735-45	4932	4.317385748	6.075555888	0.404871	1E-07	5E-07
HEY1	hes related family bHLH transcription fact	protein_coding	chr8:79764010-79	3226	0.76801547	1.558937529	0.928263	1E-07	5E-07
RTN2	reticulon 2	protein_coding	chr19:45485294-4	3227	7.437820338	10.89448809	0.461651	1E-07	5E-07
CTNND1	catenin delta 1	protein_coding	chr11:57753243-5	8798	30.86060191	41.74876363	0.351256	1E-07	5E-07
APPL2	adaptor protein, phosphotyrosine interac	protein_coding	chr12:105173297-	5088	2.72419844	4.033541721	0.482593	2E-07	5E-07
HCFC1R1	host cell factor C1 regulator 1	protein_coding	chr16:3022620-30	1433	19.72007956	30.80149085	0.563179	2E-07	5E-07
LINC02593	long intergenic non-protein coding RNA 2	lncRNA	chr1:916865-9210	4152	1.592256098	3.003998759	0.833471	2E-07	5E-07
PRDX1	peroxiredoxin 1	protein_coding	chr1:45511036-45	1504	85.96124341	118.2392498	0.37368	2E-07	5E-07
SETD7	SET domain containing 7, histone lysine m	protein_coding	chr4:139495941-1	13916	9.270235845	12.73762656	0.373896	2E-07	5E-07
ARPC2	actin related protein 2/3 complex subunit	protein_coding	chr2:218217141-2	6515	23.82356399	32.2388392	0.351202	2E-07	5E-07
MRPS33	mitochondrial ribosomal protein S33	protein_coding	chr7:141002610-1	5245	5.57788417	8.19340282	0.465686	2E-07	5E-07
ADRB2	adrenoceptor beta 2	protein_coding	chr5:148825245-1	3443	2.134056028	3.266909277	0.523884	2E-07	5E-07
PALM	paralemmin	protein_coding	chr19:708935-748	4912	9.780209171	13.34930781	0.361497	2E-07	5E-07
TMF1	TATA element modulatory factor 1	protein_coding	chr3:69019827-69	7911	5.405667479	7.103809584	0.30709	2E-07	5E-07
SIRT1	sirtuin 1	protein_coding	chr10:67884656-6	4564	6.518978008	8.803004723	0.344859	2E-07	5E-07
RBL1	RB transcriptional corepressor like 1	protein_coding	chr20:36996349-3	5954	1.333653142	2.164966971	0.619762	2E-07	5E-07
GSDME	gasdermin E	protein_coding	chr7:24698355-24	8598	1.318141714	1.972924755	0.495378	2E-07	5E-07
FIP1L1	factor interacting with PAPOLA and CPSF1	protein_coding	chr4:53377641-53	5639	3.34862937	4.662615965	0.388931	2E-07	6E-07
CAPN1	calpain 1	protein_coding	chr11:65180566-6	7553	16.97422667	21.6360231	0.263715	2E-07	6E-07
SMIM17	small integral membrane protein 17	protein_coding	chr19:56643159-5	2543	1.03704985	2.093532198	0.921437	2E-07	6E-07
CLCC1	chloride channel CLIC like 1	protein_coding	chr1:108929508-1	4964	6.18890271	8.123393939	0.304956	2E-07	6E-07
IMMP2L	inner mitochondrial membrane peptidase	protein_coding	chr7:110663051-1	3411	1.31965702	2.227068165	0.672632	2E-07	6E-07
CASP9	caspase 9	protein_coding	chr1:15490832-15	5269	3.668253379	5.098589864	0.386112	2E-07	6E-07
ERV3-1	endogenous retrovirus group 3 member 1	protein_coding	chr7:64990356-65	5027	1.971519579	2.99106133	0.514258	2E-07	6E-07
ZNF621	zinc finger protein 621	protein_coding	chr3:40524878-40	8943	1.841571502	2.664129079	0.443693	2E-07	6E-07
MON1A	MON1 homolog A, secretory trafficking as	protein_coding	chr3:49907160-49	6446	1.941372893	2.753346854	0.415128	2E-07	6E-07
SNX10	sorting nexin 10	protein_coding	chr7:26291895-26	3518	0.432631681	0.992109796	1.109914	2E-07	6E-07
FBXO45	F-box protein 45	protein_coding	chr3:196568611-1	5976	2.56767284	3.711856017	0.447172	2E-07	6E-07
BBS5	Bardet-Biedl syndrome 5	protein_coding	chr2:169479480-1	4254	1.671207091	2.907754661	0.707119	2E-07	6E-07
UBXN7	UBX domain protein 7	protein_coding	chr3:196347662-1	11247	4.782367077	6.467919014	0.350433	2E-07	6E-07
COPS4	COP9 signalosome subunit 4	protein_coding	chr4:83034447-83	3566	10.81832643	14.44581491	0.332024	2E-07	6E-07
RNASEH1-AS1	RNASEH1 antisense RNA 1	lncRNA	chr2:3558474-356	4377	2.108295131	3.160137185	0.498736	2E-07	6E-07
SEC31A	SEC31 homolog A, COPII coat complex co	protein_coding	chr4:82818509-82	9297	20.5605315	26.81952361	0.297461	2E-07	6E-07
SLC25A33	solute carrier family 25 member 33	protein_coding	chr1:9539465-958	3865	2.885198297	4.589005806	0.577945	2E-07	6E-07
RPP40	ribonuclease P/MRP subunit p40	protein_coding	chr6:4994732-500	1962	2.05143836	3.916477693	0.839735	2E-07	6E-07
CEP290	centrosomal protein 290	protein_coding	chr12:88049014-8 10260		3.078225999	4.052514048	0.310224	2E-07	6E-07
C12orf66	chromosome 12 open reading frame 66	protein_coding	chr12:64186316-6	5999	0.586774851	1.127857323	0.862844	2E-07	6E-07
ARL2BP	ADP ribosylation factor like GTPase 2 binc	protein_coding	chr16:57245259-5	1994	37.00479239	49.01753303	0.319849	2E-07	6E-07
CASTOR2	cytosolic arginine sensor for mTORC1 sub	protein_coding	chr7:74964818-75	1363	2.999085006	5.165365195	0.701227	2E-07	6E-07
SERPINB9	serpin family B member 9	protein_coding	chr6:2887270-290	4143	1.264495255	2.4239187	0.853038	2E-07	6E-07
KMT5A	lysine methyltransferase 5A	protein_coding	chr12:123383773-	4574	7.162467709	9.39556168	0.305199	2E-07	6E-07
YWHAZ	tyrosine 3-monooxygenase/tryptophan 5-	protein_coding	chr8:100916523-1	8557	53.12785027	68.37822425	0.278123	2E-07	6E-07
GEM	GTP binding protein overexpressed in skel	protein_coding	chr8:94249253-94	2648	9.1319951	13.00882615	0.426535	2E-07	6E-07
CCM2	CCM2 scaffold protein	protein_coding	chr7:44999475-45	8196	3.20151605	4.252879536	0.3228	2E-07	6E-07
PHF13	PHD finger protein 13	protein_coding	chr1:6613731-662	3632	7.250649592	9.676804412	0.329332	2E-07	6E-07
C4A	complement C4A (Rodgers blood group)	protein_coding	chr6:31982057-32	7158	1.509826504	3.076060846	0.943284	2E-07	6E-07
SSR1	signal sequence receptor subunit 1	protein_coding	chr6:7268306-734	11349	5.459442429	7.020918491	0.276353	2E-07	6E-07
HIST1H2BC	histone cluster 1 H2B family member c	protein_coding	chr6:26114873-26	834	2.048724216	4.865556478	1.16104	2E-07	6E-07
GRAMD1A	GRAM domain containing 1A	protein_coding	chr19:34994784-3	5942	8.125263032	10.46878084	0.278802	2E-07	6E-07
STON1	stonin 1	protein_coding	chr2:48529383-48	7709	2.389348579	3.596376701	0.501009	2E-07	6E-07
CCDC9	coiled-coil domain containing 9	protein_coding	chr19:47255980-4	3781	6.217149972	8.64525442	0.391482	2E-07	6E-07
CYBC1	cytochrome b-245 chaperone 1	protein_coding	chr17:82442586-8	7237	2.955415626	4.038597072	0.365093	2E-07	6E-07
PRKAG2	protein kinase AMP-activated non-catalyt	protein_coding	chr7:151556124-1	16427	11.54312342	14.94676349	0.286972	2E-07	6E-07
TMEM131	transmembrane protein 131	protein_coding	chr2:97756333-97	13042	5.838704606	8.146747393	0.396384	2E-07	6E-07
CRNKL1	crooked neck pre-mRNA splicing factor 1	protein_coding	chr20:20034368-2	4567	6.614153017	8.745678295	0.315502	2E-07	6E-07
DCTN5	dynactin subunit 5	protein_coding	chr16:23641466-2	12683	5.649679349	7.471713925	0.317862	2E-07	6E-07
LINC00342	long intergenic non-protein coding RNA 3	lncRNA	chr2:95807052-95	8599	1.798893053	2.824286588	0.566484	2E-07	6E-07
SLBP	stem-loop binding protein	protein_coding	chr4:1692731-171	1884	10.89507338	15.22357576	0.393724	2E-07	6E-07
CRYAB	crystallin alpha B	protein_coding	chr11:111908619-	4333	110.6589408	161.4908797	0.461621	2E-07	6E-07
AC027796.4	novel transcript, antisense to CTNS	lncRNA	chr17:3655621-36	874	5.073325681	10.39946365	0.960393	2E-07	6E-07
TBC1D14	TBC1 domain family member 14	protein_coding	chr4:6909242-703	6260	10.21485275	13.31741634	0.296789	2E-07	6E-07
GNA12	G protein subunit alpha 12	protein_coding	chr7:2728105-284	6111	17.33939604	22.19268122	0.269674	2E-07	6E-07
IMP4	IMP U3 small nucleolar ribonucleoprotein	protein_coding	chr2:130342225-1	3861	10.02531076	13.36664764	0.329914	2E-07	6E-07
SLC1A5	solute carrier family 1 member 5	protein_coding	chr19:46774883-4	3332	4.858534588	6.920289096	0.42435	2E-07	6E-07
MOB1B	MOB kinase activator 1B	protein_coding	chr4:70902326-71	8801	7.413352731	9.563511248	0.281559	2E-07	6E-07
GVQW3	GVQW motif containing 3	protein_coding	chr11:76381303-7 20110		0.735578559	1.040067429	0.415006	2E-07	6E-07
AKT3	AKT serine/threonine kinase 3	protein_coding	chr1:243488233-2	9475	2.011279089	3.10417857	0.54267	2E-07	6E-07
MAPKBP1	mitogen-activated protein kinase binding	protein_coding	chr15:41774434-4	10357	10.02357168	14.23679842	0.421758	2E-07	6E-07
RBMXL1	RBMX like 1	protein_coding	chr1:88979456-88	5087	4.497740552	6.101083301	0.354368	2E-07	6E-07
RAMAC	RNA guanine-7 methyltransferase activati	protein_coding	chr15:82986210-8	1565	7.991566791	11.41109976	0.42967	2E-07	6E-07
DALRD3	DALR anticodon binding domain containi	protein_coding	chr3:49015488-49	3686	4.871134197	6.630757656	0.357207	2E-07	7E-07
REC114	REC114 meiotic recombination protein	protein_coding	chr15:73443164-7	920	1.066548333	2.934672895	1.349006	2E-07	7E-07
RPS19	ribosomal protein S19	protein_coding	chr19:41859918-4	3799	54.92891828	76.19731469	0.387463	2E-07	7E-07
MAST4-AS1	MAST4 antisense RNA 1	lncRNA	chr5:67001383-67	777	0.424499402	1.563240697	1.771058	2E-07	7E-07
PTGES3	prostaglandin E synthase 3	protein_coding	chr12:56663341-5	3399	38.48621236	49.16772559	0.267462	2E-07	7E-07
TMEM219	transmembrane protein 219	protein_coding	chr16:29940885-2	2377	9.875329581	14.23551077	0.437909	2E-07	7E-07
ANGEL2	angel homolog 2	protein_coding	chr1:212992182-2	7317	2.605825035	3.865663803	0.485587	2E-07	7E-07
COL6A2	collagen type VI alpha 2 chain	protein_coding	chr21:46098097-4	5350	11.02657208	15.31486073	0.387336	2E-07	7E-07
RAD51-AS1	RAD51 antisense RNA 1	lncRNA	chr15:40686183-4	2820	1.897637466	3.333813628	0.72548	2E-07	7E-07
MRPS18B	mitochondrial ribosomal protein S18B	protein_coding	chr6:30617840-30	1757	33.24892965	44.45725189	0.334203	2E-07	7E-07
HDLBP	high density lipoprotein binding protein	protein_coding	chr2:241227264-2	12160	44.80868749	58.51442484	0.299085	2E-07	7E-07
PLD3	phospholipase D family member 3	protein_coding	chr19:40348456-4	5186	79.20052251	101.1084941	0.266122	2E-07	7E-07
TIGD1	tigger transposable element derived 1	protein_coding	chr2:232547970-2	2623	1.521649633	2.730522019	0.760796	2E-07	7E-07
G6PD	glucose-6-phosphate dehydrogenase	protein_coding	chrX:154531391-1	3758	2.479657241	3.848509864	0.543358	2E-07	7E-07
PRR11	proline rich 11	protein_coding	chr17:59155499-5 4080		2.331687442	3.783003776	0.610461	2E-07	7E-07
MLIP	muscular LMNA interacting protein	protein_coding	chr6:53929982-54	7293	30.11569886	40.06222196	0.325751	2E-07	7E-07
GTPBP6	GTP binding protein 6 (putative)	protein_coding	chrX:304529-3188	2964	23.76372454	30.59919552	0.278459	2E-07	7E-07
FECH	ferrochelatase	protein_coding	chr18:57544389-5	8772	5.815475483	7.638279883	0.306235	2E-07	7E-07
UBE2G1	ubiquitin conjugating enzyme E2 G1	protein_coding	chr17:4269259-43	4539	21.14402375	27.90759336	0.314398	2E-07	7E-07
TMEM39A	transmembrane protein 39A	protein_coding	chr3:119428949-1	5867	3.48925045	4.794019693	0.371436	2E-07	7E-07
RNF11	ring finger protein 11	protein_coding	chr1:51236273-51	3112	42.00108395	54.67059897	0.293871	2E-07	7E-07
KIF2A	kinesin family member 2A	protein_coding	chr5:62306162-62	4774	6.386404903	8.459357371	0.319299	2E-07	7E-07
ZBTB22	zinc finger and BTB domain containing 22	protein_coding	chr6:33314406-33	2737	10.59577883	14.45508022	0.362645	2E-07	7E-07
RPL34	ribosomal protein L34	protein_coding	chr4:108620566-1	3393	20.18452459	29.50663518	0.459181	2E-07	7E-07
STEAP3	STEAP3 metalloreductase	protein_coding	chr2:119223831-1	4562	0.78101777	1.372515021	0.738241	2E-07	7E-07
RABL2A	RAB, member of RAS oncogene family like	protein_coding	chr2:113627229-1	6008	1.69014003	2.498343125	0.475891	2E-07	8E-07
GNE	glucosamine (UDP-N-acetyl)-2-epimerase/	protein_coding	chr9:36214441-36	5870	2.401893414	3.496844221	0.452141	2E-07	8E-07
STRADB	STE20 related adaptor beta	protein_coding	chr2:201387858-2	3177	14.88393221	19.69412616	0.316405	2E-07	8E-07
SOS2	SOS Ras/Rho guanine nucleotide exchang	protein_coding	chr14:50117130-5	6948	8.685457443	11.1100016	0.26862	2E-07	8E-07
TMEM50B	transmembrane protein 50B	protein_coding	chr21:33432485-3	4458	9.719887537	12.84008847	0.31629	2E-07	8E-07
ANKRD33B	ankyrin repeat domain 33B	protein_coding	chr5:10564070-10	10043	3.485076231	5.070105418	0.457612	2E-07	8E-07
EIF3L	eukaryotic translation initiation factor 3 s	protein_coding	chr22:37848868-3	4296	25.69896194	33.06668488	0.276941	2E-07	8E-07
TTC37	tetratricopeptide repeat domain 37	protein_coding	chr5:95461755-95	10454	10.02506227	12.85914304	0.273271	2E-07	8E-07
ARMH3	armadillo like helical domain containing 3	protein_coding	chr10:101845599-	4747	5.235436648	7.350091136	0.403034	3E-07	8E-07
CPD	carboxypeptidase D	protein_coding	chr17:30378927-3	12510	8.816105495	11.51124839	0.299291	3E-07	8E-07
STK19	serine/threonine kinase 19	protein_coding	chr6:31971091-31	3954	1.994515347	3.256858156	0.620461	3E-07	8E-07
ERN1	endoplasmic reticulum to nucleus signali	protein_coding	chr17:64039142-6	10864	0.929943904	1.532908152	0.643791	3E-07	8E-07
PPID	peptidylprolyl isomerase D	protein_coding	chr4:158709127-1	2516	20.93557243	28.66701941	0.368653	3E-07	8E-07
NISCH	nischarin	protein_coding	chr3:52455118-52	9062	21.061255	27.68269531	0.309236	3E-07	8E-07

ZNF841	zinc finger protein 841	protein_coding	chr19:52064466-5	4287	0.978571884	1.601295539	0.625531	3E-07	8E-07
AL603839.3	novel transcript	lncRNA	chr1:40493157-40	605	0.914834149	2.41898337	1.302435	3E-07	8E-07
GSDMB	gasdermin B	protein_coding	chr17:39904595-3	5905	0.899800402	1.514771511	0.661308	3E-07	8E-07
MANF	mesencephalic astrocyte derived neurotr	protein_coding	chr3:51385047-51	2383	13.3365274	20.21548273	0.517745	3E-07	8E-07
HHATL	hedgehog acyltransferase like	protein_coding	chr3:42692663-42	2891	13.7917303	19.84330548	0.444646	3E-07	8E-07
BBS9	Bardet-Biedl syndrome 9	protein_coding	chr7:33129244-33	6195	1.527702804	2.237483317	0.461819	3E-07	8E-07
FBXW2	F-box and WD repeat domain containing 2	protein_coding	chr9:120751978-1	11267	9.431397019	12.24439615	0.290517	3E-07	8E-07
NT5DC2	5'-nucleotidase domain containing 2	protein_coding	chr3:52524385-52	4799	13.88191661	18.09012507	0.295517	3E-07	8E-07
SYPL1	synaptophysin like 1	protein_coding	chr7:106090503-1	2804	23.6533715	30.93387893	0.300873	3E-07	8E-07
TTC32	tetratricopeptide repeat domain 32	protein_coding	chr2:19896631-19	1995	2.642251432	4.623421417	0.720391	3E-07	9E-07
APIP	APAF1 interacting protein	protein_coding	chr11:34853094-3	5217	3.899530135	5.686939372	0.463142	3E-07	9E-07
SCML2	Scm polycomb group protein like 2	protein_coding	chrX:18239313-18	4327	2.258018379	3.334981572	0.474029	3E-07	9E-07
OTUB1	OTU deubiquitinase, ubiquitin aldehyde b	protein_coding	chr11:63985853-6	5336	14.14546315	18.92355743	0.334872	3E-07	9E-07
PITHD1	PITH domain containing 1	protein_coding	chr1:23778418-23	2173	14.31384848	19.69069684	0.373188	3E-07	9E-07
AADAT	aminoadipate aminotransferase	protein_coding	chr4:170060222-1	2970	2.802447036	4.234851385	0.508807	3E-07	9E-07
RFFL	ring finger and FYVE like domain containir	protein_coding	chr17:35005990-3	8512	0.877058336	1.32463127	0.507746	3E-07	9E-07
PPIE	peptidylprolyl isomerase E	protein_coding	chr1:39692182-39	8071	2.51636414	3.45778379	0.370298	3E-07	9E-07
TTLL7	tubulin tyrosine ligase like 7	protein_coding	chr1:83865024-83	10547	0.8161211	1.231133051	0.506282	3E-07	9E-07
PCMT1	protein-L-isoaspartate (D-aspartate) O-me	protein_coding	chr6:149749443-1	2832	30.8913724	40.52817486	0.306604	3E-07	9E-07
BRF2	BRF2 RNA polymerase III transcription init	protein_coding	chr8:37843268-37	2760	4.118424314	6.162935966	0.490178	3E-07	9E-07
SPHK2	sphingosine kinase 2	protein_coding	chr19:48619291-4	6009	4.667647727	6.385688248	0.364852	3E-07	9E-07
UBE3C	ubiquitin protein ligase E3C	protein_coding	chr7:157138916-1	9215	13.62719997	17.78625003	0.298543	3E-07	9E-07
NUDT19	nudix hydrolase 19	protein_coding	chr19:32691961-3	2967	3.036773507	4.749832169	0.552168	3E-07	9E-07
AC012378.2	novel transcript, antisense to PYGO1	lncRNA	chr15:55588666-5	663	0.665168531	2.036418695	1.508139	3E-07	9E-07
RRP1B	ribosomal RNA processing 1B	protein_coding	chr21:43659560-4	5750	6.216212637	8.498029774	0.367116	3E-07	9E-07
CAPN3	calpain 3	protein_coding	chr15:42359500-4	4995	1.463903745	2.638982864	0.767217	3E-07	9E-07
SMO	smoothened, frizzled class receptor	protein_coding	chr7:129188633-1	4750	15.02512962	19.76012384	0.309439	3E-07	9E-07
CPT1C	carnitine palmitoyltransferase 1C	protein_coding	chr19:49690898-4	5864	1.608427776	2.301972756	0.433864	3E-07	9E-07
GTF2H3	general transcription factor IIH subunit 3	protein_coding	chr12:123633739-	3994	4.992492169	6.871685675	0.374751	3E-07	9E-07
MYPOP	Myb related transcription factor, partner	protein_coding	chr19:45890023-4	1896	8.51264209	11.81942555	0.38988	3E-07	9E-07
RNPC3	RNA binding region (RNP1, RRM) containi	protein_coding	chr1:103525691-1	6867	6.449057648	8.483742751	0.310799	3E-07	9E-07
MRPL19	mitochondrial ribosomal protein L19	protein_coding	chr2:75646783-75	8947	6.034664168	7.79401235	0.281952	3E-07	9E-07
SMYD5	SMYD family member 5	protein_coding	chr2:73214222-73	3364	8.55892962	12.16451744	0.423194	3E-07	1E-06
GPR161	G protein-coupled receptor 161	protein_coding	chr1:168079543-1	11224	0.818972911	1.193520973	0.461693	3E-07	1E-06
ANKRD13D	ankyrin repeat domain 13D	protein_coding	chr11:67288547-6	6643	2.948467569	4.083492255	0.382686	3E-07	1E-06
C6orf62	chromosome 6 open reading frame 62	protein_coding	chr6:24704861-24	2591	31.91303557	42.27412289	0.319463	3E-07	1E-06
NOA1	nitric oxide associated 1	protein_coding	chr4:56963350-56	2218	10.87546417	15.08830711	0.383568	3E-07	1E-06
KBTBD3	kelch repeat and BTB domain containing 3	protein_coding	chr11:106051098-	5210	0.708962045	1.263275864	0.740595	3E-07	1E-06
CANT1	calcium activated nucleotidase 1	protein_coding	chr17:78991716-7	4422	9.699619176	12.46915742	0.276334	3E-07	1E-06
TDRD7	tudor domain containing 7	protein_coding	chr9:97412096-97	4174	1.479895195	2.363165478	0.587108	3E-07	1E-06
SDF2L1	stromal cell derived factor 2 like 1	protein_coding	chr22:21642302-2	1059	11.18501992	16.74482602	0.491826	3E-07	1E-06
SLC6A16	solute carrier family 6 member 16	protein_coding	chr19:49289638-4	3599	0.803916735	1.642325315	0.927663	3E-07	1E-06
ALS2	alsin Rho guanine nucleotide exchange fac	protein_coding	chr2:201700267-2	10438	11.48964494	15.04182822	0.303372	3E-07	1E-06
PSIP1	PC4 and SFRS1 interacting protein 1	protein_coding	chr9:15464066-15	6744	7.240638853	9.677353369	0.333303	3E-07	1E-06
RPL31	ribosomal protein L31	protein_coding	chr2:101002229-1	5277	38.57823157	52.97136619	0.370689	3E-07	1E-06
HIST1H2AE	histone cluster 1 H2A family member e	protein_coding	chr6:26216975-26	509	1.02371044	3.607205166	1.749375	3E-07	1E-06
ZNF48	zinc finger protein 48	protein_coding	chr16:30378106-3	3744	4.203294611	6.2524716	0.484431	3E-07	1E-06
SLC12A2	solute carrier family 12 member 2	protein_coding	chr5:128083766-1	10653	2.418446377	3.237143584	0.334784	3E-07	1E-06
ZNF354C	zinc finger protein 354C	protein_coding	chr5:179060415-1	5411	0.573518905	1.016162796	0.740507	3E-07	1E-06
PPP1CA	protein phosphatase 1 catalytic subunit a	protein_coding	chr11:67398183-6	2651	30.22163378	39.59339004	0.303854	3E-07	1E-06
RPS8	ribosomal protein S8	protein_coding	chr1:44775251-44	2781	126.1591666	174.6641819	0.385243	3E-07	1E-06
SPAG16	sperm associated antigen 16	protein_coding	chr2:213284379-2	8257	2.438951259	3.490994917	0.429412	3E-07	1E-06
PLEKHH3	pleckstrin homology, MyTH4 and FERM do	protein_coding	chr17:42667914-4	4803	12.37266723	16.57246744	0.335271	3E-07	1E-06
PTPN1	protein tyrosine phosphatase non-recepto	protein_coding	chr20:50510321-5	4041	9.759983685	12.6020523	0.283051	3E-07	1E-06
SCO1	SCO cytochrome c oxidase assembly prote	protein_coding	chr17:10672474-1	10299	6.107167239	7.804579031	0.267492	3E-07	1E-06
JAZF1	JAZF zinc finger 1	protein_coding	chr7:27830573-28	4258	19.03045009	25.17315237	0.318368	3E-07	1E-06
NPDC1	neural proliferation, differentiation and c	protein_coding	chr9:137039463-1	2720	17.45617538	23.10831828	0.317933	3E-07	1E-06
ZFP14	ZFP14 zinc finger protein	protein_coding	chr19:36334453-3	7819	0.595292126	0.971084775	0.623392	4E-07	1E-06
C11orf80	chromosome 11 open reading frame 80	protein_coding	chr11:66744451-6	3765	0.741628688	1.316633792	0.740031	4E-07	1E-06
PSTPIP2	proline-serine-threonine phosphatase inte	protein_coding	chr18:45983536-4	4120	1.959005532	3.083184523	0.559998	4E-07	1E-06
MIR34AHG	MIR34A host gene	lncRNA	chr1:9148011-919	8702	2.529871477	3.456789843	0.362108	4E-07	1E-06
TMED3	transmembrane p24 trafficking protein 3	protein_coding	chr15:79311112-7	19413	1.606952317	2.311020372	0.438303	4E-07	1E-06
ZBED4	zinc finger BED-type containing 4	protein_coding	chr22:49853844-4	6893	3.252173238	4.593303149	0.412362	4E-07	1E-06
AC129492.2	novel transcript, antisense to ALOX12B	lncRNA	chr17:8079482-80	1804	0.28561492	0.961807645	1.679659	4E-07	1E-06
PTGR2	prostaglandin reductase 2	protein_coding	chr14:73851844-7	8072	1.91478143	2.781732004	0.453321	4E-07	1E-06
GEMIN8	gem nuclear organelle associated protein	protein_coding	chrX:14008279-14	2424	4.434023468	6.31236198	0.421181	4E-07	1E-06
LINC00957	long intergenic non-protein coding RNA 9	lncRNA	chr7:44039171-44	2703	2.15525741	3.755356351	0.72094	4E-07	1E-06
BET1L	Bet1 golgi vesicular membrane trafficking	protein_coding	chr11:167784-207	3868	10.78783902	13.78510674	0.267359	4E-07	1E-06
TXN2	thioredoxin 2	protein_coding	chr22:36467046-3	1650	43.42211286	59.27111653	0.364483	4E-07	1E-06
TRMT2A	tRNA methyltransferase 2 homolog A	protein_coding	chr22:20111875-2	3954	6.881067713	9.211970449	0.335436	4E-07	1E-06
SLC50A1	solute carrier family 50 member 1	protein_coding	chr1:155135344-1	2340	5.564482227	8.665836066	0.563666	4E-07	1E-06
DUS2	dihydrouridine synthase 2	protein_coding	chr16:67987746-6	4175	2.085954989	3.032608956	0.452229	4E-07	1E-06
UTP18	UTP18 small subunit processome compor	protein_coding	chr17:51260546-5	2703	2.943055022	4.61986236	0.56294	4E-07	1E-06
INAFM1	InaF motif containing 1	protein_coding	chr19:47274453-4	1255	7.687106183	11.45594283	0.495264	4E-07	1E-06
FHL2	four and a half LIM domains 2	protein_coding	chr2:105357712-1	6626	287.3529698	380.9163956	0.321311	4E-07	1E-06
TESC	tescalcin	protein_coding	chr12:117038923-	1926	4.016639408	6.365880099	0.578214	4E-07	1E-06
RCN1	reticulocalbin 1	protein_coding	chr11:32090904-3	4675	5.497072794	7.32341272	0.328294	4E-07	1E-06
GPR137C	G protein-coupled receptor 137C	protein_coding	chr14:52552836-5	6505	0.588061059	1.017536864	0.696942	4E-07	1E-06
L2HGDH	L-2-hydroxyglutarate dehydrogenase	protein_coding	chr14:50237563-5	6560	3.586212001	4.800520447	0.334345	4E-07	1E-06
SMARCC2	SWI/SNF related, matrix associated, actin	protein_coding	chr12:56162359-5	9179	11.3178577	15.33537948	0.353149	4E-07	1E-06
RPS4X	ribosomal protein S4 X-linked	protein_coding	chrX:72255679-72	2930	96.24247102	131.8173603	0.369335	4E-07	1E-06
RPLP0	ribosomal protein lateral stalk subunit P0	protein_coding	chr12:120196699-	3988	129.524362	174.4335617	0.343948	4E-07	1E-06
EEF1A2	eukaryotic translation elongation factor 1	protein_coding	chr20:63488014-6	4771	271.6357885	353.2080046	0.292979	4E-07	1E-06
IFNGR2	interferon gamma receptor 2	protein_coding	chr21:33402896-3	2960	9.487039266	12.32573518	0.291272	4E-07	1E-06
CYFIP2	cytoplasmic FMR1 interacting protein 2	protein_coding	chr5:157266079-1	10498	24.06934482	31.82153007	0.317403	4E-07	1E-06
BOLA3-AS1	BOLA3 divergent transcript	lncRNA	chr2:74148007-74	3727	1.391072562	2.293832213	0.644689	4E-07	1E-06
CLTCL1	clathrin heavy chain like 1	protein_coding	chr22:19179473-1	10052	0.947753692	1.459745074	0.54009	4E-07	1E-06
AC005332.4	novel transcript	lncRNA	chr17:68205489-6	2005	1.303455358	2.472261012	0.828082	4E-07	1E-06
FBXL2	F-box and leucine rich repeat protein 2	protein_coding	chr3:33277025-33	7664	1.48821711	2.244471366	0.500043	4E-07	1E-06
SMIM10	small integral membrane protein 10	protein_coding	chrX:134990991-1	1483	1.65846121	3.065828026	0.805652	4E-07	1E-06
TMLHE	trimethyllysine hydroxylase, epsilon	protein_coding	chrX:155489011-1	5042	2.835034978	4.226941051	0.497278	4E-07	1E-06
SLC12A9	solute carrier family 12 member 9	protein_coding	chr7:100826820-1	8901	2.529483293	3.612033815	0.426871	4E-07	1E-06
DKC1	dyskerin pseudouridine synthase 1	protein_coding	chrX:154762742-1	4203	6.921761646	9.116904663	0.30937	4E-07	1E-06
THAP12	THAP domain containing 12	protein_coding	chr11:76349956-7	7888	4.592031123	5.915597106	0.278909	4E-07	1E-06
UBE4A	ubiquitination factor E4A	protein_coding	chr11:118359585-	6576	10.84489189	13.87904565	0.270006	4E-07	1E-06
TM7SF2	transmembrane 7 superfamily member 2	protein_coding	chr11:65111845-6	3408	13.66755687	17.90324921	0.303766	5E-07	1E-06
EML3	EMAP like 3	protein_coding	chr11:62602218-6	5490	1.162674773	1.985687999	0.692732	5E-07	1E-06
TCFL5	transcription factor like 5	protein_coding	chr20:62841115-6	3436	2.177992047	3.483596009	0.584786	5E-07	1E-06
POLR3D	RNA polymerase III subunit D	protein_coding	chr8:22245133-22	5583	4.876495808	6.433455967	0.311385	5E-07	1E-06
MAPKAPK2	mitogen-activated protein kinase-activate	protein_coding	chr1:206684944-2	4195	66.97659084	86.72383811	0.285704	5E-07	1E-06
GATM	glycine amidinotransferase	protein_coding	chr15:45361124-4	6398	5.704452678	7.618324707	0.332152	5E-07	1E-06
LDB1	LIM domain binding 1	protein_coding	chr10:102107560-	2790	48.48707826	63.45438962	0.302416	5E-07	1E-06
COBLL1	cordon-bleu WH2 repeat protein like 1	protein_coding	chr2:164653624-1	17179	0.811589364	1.230663012	0.515671	5E-07	1E-06
MTERF3	mitochondrial transcription termination	protein_coding	chr8:96239398-96	1801	6.75985888	9.538303458	0.41023	5E-07	1E-06
DPH5	diphthamide biosynthesis 5	protein_coding	chr1:100989623-1	2970	9.969696067	13.60967819	0.364804	5E-07	1E-06
ZNF581	zinc finger protein 581	protein_coding	chr19:55635459-5	1635	5.58081072	8.218570538	0.472686	5E-07	1E-06
RAB26	RAB26, member RAS oncogene family	protein_coding	chr16:2140803-21	2488	0.621687765	1.375786875	1.069965	5E-07	1E-06
AGGF1	angiogenic factor with G-patch and FHA d	protein_coding	chr5:77029251-77	5032	2.821894362	3.991117359	0.41649	5E-07	1E-06
ZNF875	zinc finger protein 875	protein_coding	chr19:37312837-3	7337	4.33977692	5.767376133	0.32317	5E-07	1E-06
FAM228B	family with sequence similarity 228 meml	protein_coding	chr2:24076526-24	4927	0.618847421	1.151045575	0.802135	5E-07	1E-06
BCKDHB	branched chain keto acid dehydrogenase	protein_coding	chr6:80106647-80	4071	7.673519712	10.27382995	0.337328	5E-07	1E-06

FGF13	fibroblast growth factor 13	protein_coding	chrX:138614727-1	20737	2.841098707	3.655089095	0.276473	5E-07	1E-06
OSBPL3	oxysterol binding protein like 3	protein_coding	chr7:24796540-24	8089	1.774758926	2.409301347	0.354523	5E-07	1E-06
BTBD1	BTB domain containing 1	protein_coding	chr15:83016422-8	4169	62.32052083	80.7654473	0.288619	5E-07	1E-06
CCT3	chaperonin containing TCP1 subunit 3	protein_coding	chr1:156308968-1	3864	49.87835183	65.67079041	0.311128	5E-07	2E-06
BX293535.1	novel transcript	lncRNA	chr1:27525805-27	283	3.467764855	8.159668118	1.134712	5E-07	2E-06
DIPK2A	divergent protein kinase domain 2A	protein_coding	chr3:143971798-1	5487	9.232512426	12.09917212	0.302731	5E-07	2E-06
DAP	death associated protein	protein_coding	chr5:10679230-10	3361	37.07035634	47.3054182	0.265839	5E-07	2E-06
GLI4	GLI family zinc finger 4	protein_coding	chr8:143267433-1	3057	3.606581673	5.34124813	0.47927	5E-07	2E-06
ZNF333	zinc finger protein 333	protein_coding	chr19:14689801-1	11603	0.856256216	1.280102354	0.492911	5E-07	2E-06
UBE2L3	ubiquitin conjugating enzyme E2 L3	protein_coding	chr22:21549447-2	3838	19.50043336	26.05999409	0.333488	5E-07	2E-06
S100A4	S100 calcium binding protein A4	protein_coding	chr1:153543613-1	946	8.625022854	13.61758084	0.576386	5E-07	2E-06
ANKS6	ankyrin repeat and sterile alpha motif don	protein_coding	chr9:98731329-98	8862	1.82304235	2.484628702	0.360087	5E-07	2E-06
RTF2	replication termination factor 2	protein_coding	chr20:56468585-5	4063	11.17254642	14.49654029	0.288082	5E-07	2E-06
AC026471.1	novel transcript, antisense to ARMC5	lncRNA	chr16:31456711-3	3026	5.23298256	7.252191787	0.388133	5E-07	2E-06
XIST	X inactive specific transcript	lncRNA	chrX:73820649-73	25266	9.478010757	13.41098784	0.417754	5E-07	2E-06
P2RX4	purinergic receptor P2X 4	protein_coding	chr12:121209857-	8159	0.895500751	1.373136441	0.535826	5E-07	2E-06
C1orf53	chromosome 1 open reading frame 53	protein_coding	chr1:197902630-1	628	5.542794647	10.02614263	0.760437	6E-07	2E-06
DACT3	dishevelled binding antagonist of beta cat	protein_coding	chr19:46647551-4	5466	27.16096488	37.40008897	0.375854	6E-07	2E-06
MARK4	microtubule affinity regulating kinase 4	protein_coding	chr19:45079288-4	6656	10.87760952	13.9965319	0.277319	6E-07	2E-06
ABCF2	ATP binding cassette subfamily F member	protein_coding	chr7:151207837-1	2185	27.87827441	36.1753188	0.289979	6E-07	2E-06
TRO	trophinin	protein_coding	chrX:54920462-54	6716	5.083266588	6.997069222	0.375877	6E-07	2E-06
ALKBH7	alkB homolog 7	protein_coding	chr19:6372794-63	1093	63.83216549	86.04724461	0.345236	6E-07	2E-06
INVS	inversin	protein_coding	chr9:100099243-1	7131	1.583297568	2.25328055	0.422044	6E-07	2E-06
MAST3	microtubule associated serine/threonine	protein_coding	chr19:18097793-1	6772	2.769131987	3.807319755	0.37419	6E-07	2E-06
GPR173	G protein-coupled receptor 173	protein_coding	chrX:53049091-53	4608	1.312725157	2.110255044	0.59695	6E-07	2E-06
BBS2	Bardet-Biedl syndrome 2	protein_coding	chr16:56466836-5	8141	3.633167788	4.718232576	0.292287	6E-07	2E-06
DTNBP1	dystrobrevin binding protein 1	protein_coding	chr6:15522801-15	3052	4.167777556	5.995763339	0.441896	6E-07	2E-06
LRRC29	leucine rich repeat containing 29	protein_coding	chr16:67207139-6	2807	0.756327693	1.430443315	0.82666	6E-07	2E-06
C7orf50	chromosome 7 open reading frame 50	protein_coding	chr7:996986-1138	4961	18.4944053	23.73877634	0.273463	6E-07	2E-06
CAMK1	calcium/calmodulin dependent protein k	protein_coding	chr3:9757347-976	2715	4.063161617	5.845892151	0.436997	6E-07	2E-06
SKIL	SKI like proto-oncogene	protein_coding	chr3:170357678-1	7782	9.093257934	11.60583058	0.265523	6E-07	2E-06
GLE1	GLE1 RNA export mediator	protein_coding	chr9:128504700-1	3527	10.94207577	14.30155258	0.299465	6E-07	2E-06
CREBL2	cAMP responsive element binding protein	protein_coding	chr12:12611827-1	3922	6.983960334	9.357872975	0.333907	6E-07	2E-06
SLC37A3	solute carrier family 37 member 3	protein_coding	chr7:140293693-1	10588	2.246556512	3.105234931	0.380568	6E-07	2E-06
AC008124.1	novel transcript	lncRNA	chr12:45718046-4	9730	2.716757255	3.577762524	0.310412	6E-07	2E-06
CRIP1	cysteine rich protein 1	protein_coding	chr14:105486317-	2485	0.842145292	1.682421553	0.903678	6E-07	2E-06
NOC3L	NOC3 like DNA replication regulator	protein_coding	chr10:94333226-9	4464	2.394135012	3.574425375	0.494649	6E-07	2E-06
PTPRK	protein tyrosine phosphatase receptor typ	protein_coding	chr6:127968779-1	9000	13.86595128	17.97325158	0.288662	6E-07	2E-06
SAMD1	sterile alpha motif domain containing 1	protein_coding	chr19:14087840-1	2263	25.15710153	32.16349143	0.268106	6E-07	2E-06
ZBTB18	zinc finger and BTB domain containing 18	protein_coding	chr1:244048939-2	3906	7.960743986	10.65096817	0.332815	6E-07	2E-06
DPM2	dolichyl-phosphate mannosyltransferase	protein_coding	chr9:127935099-1	3133	4.761603117	7.10877981	0.501227	6E-07	2E-06
EVI5	ecotropic viral integration site 5	protein_coding	chr1:92508696-92	8276	2.849307906	3.741162322	0.305335	6E-07	2E-06
RNLS	renalase, FAD dependent amine oxidase	protein_coding	chr10:88273864-8	4790	0.56534612	1.007518281	0.748383	6E-07	2E-06
RIC1	RIC1 homolog, RAB6A GEF complex partn	protein_coding	chr9:5629025-577	8591	4.179411752	5.583402211	0.330126	7E-07	2E-06
GCFC2	GC-rich sequence DNA-binding factor 2	protein_coding	chr2:75652000-75	5465	3.82205217	5.340393219	0.397204	7E-07	2E-06
TRDN-AS1	TRDN antisense RNA 1	lncRNA	chr6:123389421-1	7268	9.552970836	12.561448	0.309608	7E-07	2E-06
SH3RF1	SH3 domain containing ring finger 1	protein_coding	chr4:169094259-1	5472	1.236587688	2.26865251	0.788551	7E-07	2E-06
RAB11A	RAB11A, member RAS oncogene family	protein_coding	chr15:65726054-6	5680	18.02912852	23.46534421	0.294791	7E-07	2E-06
SFMBT2	Scm like with four mbt domains 2	protein_coding	chr10:7158624-74	9953	1.800074252	2.63258622	0.464248	7E-07	2E-06
PWP1	PWP1 homolog, endonuclein	protein_coding	chr12:107685799-	2804	6.213933774	8.349423844	0.338435	7E-07	2E-06
SECISBP2	SECIS binding protein 2	protein_coding	chr9:89318506-89	9813	2.731816398	3.649010375	0.332135	7E-07	2E-06
PI4KB	phosphatidylinositol 4-kinase beta	protein_coding	chr1:151291797-1	5456	9.898291989	12.84377138	0.289494	7E-07	2E-06
RELCH	RAB11 binding and LisH domain, coiled-c	protein_coding	chr18:62187255-6 10690		2.902232483	3.742939443	0.27942	7E-07	2E-06
RAB30-DT	RAB30 divergent transcript	lncRNA	chr11:83072052-8	7192	1.517386258	2.327704121	0.526004	7E-07	2E-06
LXN	latexin	protein_coding	chr3:158645822-1	1459	2.607948105	4.576655168	0.719806	7E-07	2E-06
AL162595.1	novel transcript	lncRNA	chr1:229256892-2	8197	0.958356716	1.578076435	0.634278	7E-07	2E-06
NTN1	netrin 1	protein_coding	chr17:9021510-92	6415	1.557664776	2.482851272	0.590182	7E-07	2E-06
AKAP6	A-kinase anchoring protein 6	protein_coding	chr14:32329298-3	19726	14.96702813	19.97096562	0.33186	7E-07	2E-06
AFTPH	aftiphilin	protein_coding	chr2:64524305-64	5767	2.615524237	3.775165598	0.441995	7E-07	2E-06
CD81-AS1	CD81 antisense RNA 1	lncRNA	chr11:2328749-23	1542	0.397368833	1.017803005	1.263812	7E-07	2E-06
PIGB	phosphatidylinositol glycan anchor biosy	protein_coding	chr15:55318960-5	4542	0.583835335	1.22556679	0.994734	7E-07	2E-06
PECR	peroxisomal trans-2-enoyl-CoA reductase	protein_coding	chr2:215996329-2	3196	0.994422462	1.830737573	0.795564	7E-07	2E-06
GPRASP2	G protein-coupled receptor associated so	protein_coding	chrX:102712176-1	3975	5.863229266	7.74636849	0.315728	7E-07	2E-06
PYCR3	pyrroline-5-carboxylate reductase 3	protein_coding	chr8:143603210-1	3947	1.925449774	2.851005176	0.477152	7E-07	2E-06
PARN	poly(A)-specific ribonuclease	protein_coding	chr16:14435700-1	9284	3.603826399	4.865229496	0.344682	7E-07	2E-06
GBA	glucosylceramidase beta	protein_coding	chr1:155234452-1	3648	2.99491285	4.220576846	0.408265	7E-07	2E-06
FAM133B	family with sequence similarity 133 meml	protein_coding	chr7:92560793-92	4462	2.097143123	3.094156344	0.472196	7E-07	2E-06
ODF2	outer dense fiber of sperm tails 2	protein_coding	chr9:128455186-1	6393	3.664708082	5.151698103	0.407233	7E-07	2E-06
KCNIP2	potassium voltage-gated channel interact	protein_coding	chr10:101825974-	3397	0.4550823	1.10806067	1.227952	7E-07	2E-06
MBIP	MAP3K12 binding inhibitory protein 1	protein_coding	chr14:36298564-3	3423	3.568641142	4.921408262	0.378492	7E-07	2E-06
ICAM4	intercellular adhesion molecule 4 (Landst	protein_coding	chr19:10286955-1	1439	8.012042269	12.18982534	0.517707	7E-07	2E-06
ACP1	acid phosphatase 1	protein_coding	chr2:264140-2782	4160	15.06679831	19.60094362	0.292349	7E-07	2E-06
SLC25A16	solute carrier family 25 member 16	protein_coding	chr10:68477998-6	7865	2.215541593	3.221101681	0.452564	7E-07	2E-06
DHX57	DExH-box helicase 57	protein_coding	chr2:38797729-38	7985	3.076821633	4.073754502	0.316912	7E-07	2E-06
MIA2	MIA SH3 domain ER export factor 2	protein_coding	chr14:39230231-3	10539	1.629590233	2.212407322	0.354936	7E-07	2E-06
RBMS3	RNA binding motif single stranded interac	protein_coding	chr3:28574791-30	18339	1.050663682	1.419980539	0.34788	7E-07	2E-06
LIMD2	LIM domain containing 2	protein_coding	chr17:63695888-6	4751	0.89776286	1.608005693	0.743027	7E-07	2E-06
SFXN5	sideroflexin 5	protein_coding	chr2:72942036-73	11087	1.83146551	2.461222214	0.342934	7E-07	2E-06
TMEM220	transmembrane protein 220	protein_coding	chr17:10699015-1	3508	0.589366228	1.167228302	0.901224	8E-07	2E-06
FURIN	furin, paired basic amino acid cleaving en	protein_coding	chr15:90868588-9	4732	17.37364016	23.59904827	0.35822	8E-07	2E-06
SEMA4F	ssemaphorin 4F	protein_coding	chr2:74654228-74	6227	0.945189994	1.551520579	0.632827	8E-07	2E-06
EHBP1L1	EH domain binding protein 1 like 1	protein_coding	chr11:65576046-6	6733	0.835866508	1.609873815	0.871412	8E-07	2E-06
N6AMT1	N-6 adenine-specific DNA methyltransfera	protein_coding	chr21:28872191-2	4865	5.023007796	6.978955927	0.388971	8E-07	2E-06
CAPZB	capping actin protein of muscle Z-line sub	protein_coding	chr1:19338775-19	3239	58.5777942	76.27689142	0.29547	8E-07	2E-06
SDE2	SDE2 telomere maintenance homolog	protein_coding	chr1:225982702-2	3987	6.820837851	9.193038907	0.342134	8E-07	2E-06
S100A11	S100 calcium binding protein A11	protein_coding	chr1:152032506-1	649	9.82878752	18.99915906	0.893806	8E-07	2E-06
EIF3E	eukaryotic translation initiation factor 3 s	protein_coding	chr8:108201216-1	6005	27.3654695	36.36216827	0.325358	8E-07	2E-06
PC	pyruvate carboxylase	protein_coding	chr11:66848417-6	7355	2.048482286	2.830368805	0.383497	8E-07	2E-06
ARFIP1	ADP ribosylation factor interacting protei	protein_coding	chr4:152779937-1	4269	5.463641111	7.730610985	0.41901	8E-07	2E-06
PLEKHG2	pleckstrin homology and RhoGEF domain	protein_coding	chr19:39412669-3	9116	8.12361584	11.20786085	0.379684	8E-07	2E-06
DHRS7	dehydrogenase/reductase 7	protein_coding	chr14:60144119-6	3249	2.365874477	3.676264888	0.545656	8E-07	2E-06
NUP210	nucleoporin 210	protein_coding	chr3:13316235-13	8195	16.04068553	22.76869813	0.423018	8E-07	2E-06
SLC33A1	solute carrier family 33 member 1	protein_coding	chr3:155821024-1 10040		1.116091465	1.629736573	0.456497	8E-07	2E-06
DHX37	DEAH-box helicase 37	protein_coding	chr12:124946825-	6627	2.150290869	2.957519616	0.374313	8E-07	2E-06
CDKL2	cyclin dependent kinase like 2	protein_coding	chr4:75576496-75	5290	0.590640981	1.072163607	0.777003	8E-07	2E-06
FHL3	four and a half LIM domains 3	protein_coding	chr1:37996770-38	2004	2.764392716	4.344998215	0.561095	9E-07	2E-06
PRCP	prolylcarboxypeptidase	protein_coding	chr11:82822936-8	13197	2.766268604	3.741287972	0.351596	9E-07	2E-06
RRN3	RRN3 homolog, RNA polymerase I transcri	protein_coding	chr16:15060022-1	4156	7.311638152	9.51537587	0.2931	9E-07	2E-06
KDM3A	lysine demethylase 3A	protein_coding	chr2:86440647-86	13299	4.25769179	6.284484593	0.474957	9E-07	2E-06
PRPF4	pre-mRNA processing factor 4	protein_coding	chr9:113275343-1	3613	5.567549714	7.285860527	0.300681	9E-07	2E-06
RBM27	RNA binding motif protein 27	protein_coding	chr5:146203605-1	6664	2.168202062	3.016557225	0.391213	9E-07	2E-06
PGAP1	post-GPI attachment to proteins 1	protein_coding	chr2:196833004-1	12761	2.320284058	3.049289884	0.307795	9E-07	2E-06
HMGCLL1	3-hydroxymethyl-3-methylglutaryl-CoA ly	protein_coding	chr6:55434369-55	4800	1.407984649	2.193943736	0.549518	9E-07	2E-06
NAA80	N(alpha)-acetyltransferase 80, NatH cataly	protein_coding	chr3:50296402-50	2188	2.788489705	4.33995212	0.551105	9E-07	2E-06
VCAM1	vascular cell adhesion molecule 1	protein_coding	chr1:100719742-1	6049	14.2777065	19.34814333	0.352201	9E-07	3E-06
SUOX	sulfite oxidase	protein_coding	chr12:55997180-5	5008	6.998703988	9.13158569	0.298205	9E-07	3E-06
AP5S1	adaptor related protein complex 5 subuni	protein_coding	chr20:3820524-38	5491	2.641560123	3.603112194	0.36139	9E-07	3E-06
EPHX2	epoxide hydrolase 2	protein_coding	chr8:27490779-27	4473	9.576091112	12.69913514	0.324242	9E-07	3E-06
RUSC2	RUN and SH3 domain containing 2	protein_coding	chr9:35490111-35	5698	8.426738105	10.75547787	0.266523	9E-07	3E-06
SDHAF4	succinate dehydrogenase complex assemt	protein_coding	chr6:70566917-70	1440	6.659478698	9.250441258	0.387047	9E-07	3E-06
CNFN	cornifelin	protein_coding	chr19:42387019-4	613	1.250414483	3.481521404	1.402718	9E-07	3E-06

TBX2-AS1	TBX2 antisense RNA 1	lncRNA	chr17:61393456-6	1206	2.941844739	5.457544828	0.799799	9E-07	3E-06
PPM1M	protein phosphatase, Mg2+/Mn2+ depenc	protein_coding	chr3:52245759-52	3607	1.415438707	2.319083343	0.625685	9E-07	3E-06
AC026470.1	NEDD4 binding protein 1	protein_coding	chr16:48538726-4	7810	5.704330068	7.473796661	0.303758	1E-06	3E-06
SMIM1	small integral membrane protein 1 (Vel bl	protein_coding	chr1:3772761-377	981	6.323220534	9.32983049	0.472704	1E-06	3E-06
MARC1	mitochondrial amidoxime reducing comp	protein_coding	chr1:220786913-2	9106	0.931745847	1.384697653	0.483419	1E-06	3E-06
HPRT1	hypoxanthine phosphoribosyltransferase	protein_coding	chrX:134460165-1	1612	11.6911133	15.81926655	0.352532	1E-06	3E-06
RPL4	ribosomal protein L4	protein_coding	chr15:66498015-6	5609	107.1022515	144.3293489	0.346072	1E-06	3E-06
DYRK2	dual specificity tyrosine phosphorylation	protein_coding	chr12:67648338-6	9494	11.29559194	14.59279961	0.28453	1E-06	3E-06
C4orf3	chromosome 4 open reading frame 3	protein_coding	chr4:119296419-1	2960	33.72768807	45.81306277	0.357911	1E-06	3E-06
SUMO1	small ubiquitin like modifier 1	protein_coding	chr2:202206180-2	2164	30.92153022	40.04762357	0.286711	1E-06	3E-06
CNTLN	centlein	protein_coding	chr9:17134982-17	11064	0.759709278	1.120600304	0.46875	1E-06	3E-06
EVA1B	eva-1 homolog B	protein_coding	chr1:36322031-36	1211	2.697244638	4.638789817	0.694402	1E-06	3E-06
DCAF17	DDB1 and CUL4 associated factor 17	protein_coding	chr2:171434217-1	7657	2.758687193	3.649584654	0.315464	1E-06	3E-06
CDK20	cyclin dependent kinase 20	protein_coding	chr9:87966441-87	5727	1.340926715	2.016845539	0.501734	1E-06	3E-06
KCTD15	potassium channel tetramerization doma	protein_coding	chr19:33795933-3	6388	5.724696779	7.412833782	0.285785	1E-06	3E-06
RPAP2	RNA polymerase II associated protein 2	protein_coding	chr1:92299059-92	17048	2.899135604	3.839019757	0.320266	1E-06	3E-06
TET2	tet methylcytosine dioxygenase 2	protein_coding	chr4:105145875-1	16474	4.338850161	6.031997511	0.392424	1E-06	3E-06
CACNA1G-AS1	CACNA1G antisense RNA 1	lncRNA	chr17:50556207-5	1579	0.456525491	1.047926455	1.108982	1E-06	3E-06
GOLT1B	golgi transport 1B	protein_coding	chr12:21501781-2	3789	3.884371276	5.31353774	0.36576	1E-06	3E-06
FNBP1	formin binding protein 1	protein_coding	chr9:129887187-1	6600	8.811206733	11.70477938	0.324941	1E-06	3E-06
PARD6B	par-6 family cell polarity regulator beta	protein_coding	chr20:50731580-5	4690	3.694283184	5.270801364	0.425727	1E-06	3E-06
CDK9	cyclin dependent kinase 9	protein_coding	chr9:127785679-1	3000	14.39164268	18.33914018	0.264275	1E-06	3E-06
ZNF141	zinc finger protein 141	protein_coding	chr4:337814-3848	12944	2.204113394	2.971698964	0.347522	1E-06	3E-06
TPGS1	tubulin polyglutamylase complex subunit	protein_coding	chr19:507497-519	3642	7.039021454	10.22657024	0.449051	1E-06	3E-06
PXK	PX domain containing serine/threonine ki	protein_coding	chr3:58332880-58	5354	2.867417242	3.94963297	0.37527	1E-06	3E-06
LINC01165	long intergenic non-protein coding RNA 1	lncRNA	chr10:132520827-	1623	1.928367838	3.719598799	0.874845	1E-06	3E-06
MRPL37	mitochondrial ribosomal protein L37	protein_coding	chr1:54184041-54	2549	27.28338483	36.49925513	0.335957	1E-06	3E-06
IMPDH1	inosine monophosphate dehydrogenase 1	protein_coding	chr7:128392277-1	3662	5.705321352	7.45687507	0.299863	1E-06	3E-06
C6orf120	chromosome 6 open reading frame 120	protein_coding	chr6:169702190-1	2667	14.23987518	18.37834975	0.281272	1E-06	3E-06
AP001107.5	novel transcript	lncRNA	chr11:66409158-6	1375	4.338748837	6.549525317	0.511297	1E-06	3E-06
LINC02256	long intergenic non-protein coding RNA 2	lncRNA	chr15:32536047-3	909	1.436694506	3.091463621	1.017811	1E-06	3E-06
TIMM29	translocase of inner mitochondrial memb	protein_coding	chr19:10928811-1	1785	8.520990604	11.56336302	0.355567	1E-06	3E-06
SEC61G	Sec61 translocon gamma subunit	protein_coding	chr7:54752250-54	1842	15.04020717	21.00503735	0.393596	1E-06	3E-06
UFC1	ubiquitin-fold modifier conjugating enzyr	protein_coding	chr1:161152776-1	2100	22.48695796	30.25491723	0.344461	1E-06	3E-06
MRTFB	myocardin related transcription factor B	protein_coding	chr16:14071321-1	16578	3.049701313	4.268167845	0.401752	1E-06	3E-06
UBE2E1	ubiquitin conjugating enzyme E2 E1	protein_coding	chr3:23805903-23	3074	26.57988672	36.95864102	0.393057	1E-06	3E-06
PTPN14	protein tyrosine phosphatase non-recept	protein_coding	chr1:214348700-2	18752	1.404726113	1.996138215	0.42268	1E-06	3E-06
SIN3A	SIN3 transcription regulator family memb	protein_coding	chr15:75369379-7	12453	3.672328104	5.09052361	0.387416	1E-06	3E-06
FKTN	fukutin	protein_coding	chr9:105558130-1	8747	2.230919651	3.012858169	0.346323	1E-06	3E-06
CNPY4	canopy FGF signaling regulator 4	protein_coding	chr7:100119634-1	2451	2.816284427	4.118748571	0.4627	1E-06	3E-06
KHK	ketohexokinase	protein_coding	chr2:27086747-27 3050		0.607318794	1.355768662	1.086926	1E-06	3E-06
RABL3	RAB, member of RAS oncogene family like	protein_coding	chr3:120686681-1	4684	3.673490943	5.028924578	0.36685	1E-06	3E-06
FBXL8	F-box and leucine rich repeat protein 8	protein_coding	chr16:67159932-6	3129	0.60515073	1.189901929	0.889015	1E-06	3E-06
DDX49	DEAD-box helicase 49	protein_coding	chr19:18919705-1	3306	12.49426352	16.29387801	0.29618	1E-06	3E-06
RPL13	ribosomal protein L13	protein_coding	chr16:89560657-8	6172	69.79211556	95.10972954	0.36311	1E-06	3E-06
TTC30A	tetratricopeptide repeat domain 30A	protein_coding	chr2:177612999-1	5744	1.60315015	2.543278131	0.583429	1E-06	3E-06
GORAB	golgin, RAB6 interacting	protein_coding	chr1:170532129-1	3773	4.368280033	6.08464647	0.392455	1E-06	3E-06
LAMP1	lysosomal associated membrane protein 1	protein_coding	chr13:113297239-	5741	20.24191421	26.02793654	0.277181	1E-06	3E-06
CCDC167	coiled-coil domain containing 167	protein_coding	chr6:37482938-37	572	12.79567068	21.8164004	0.693593	1E-06	3E-06
UBR1	ubiquitin protein ligase E3 component n-	protein_coding	chr15:42942897-4	9928	8.583837716	11.0496988	0.278144	1E-06	3E-06
CCNT2-AS1	CCNT2 antisense RNA 1	lncRNA	chr2:134735464-1	5175	0.454775638	0.923017058	0.932649	1E-06	3E-06
ARID5B	AT-rich interaction domain 5B	protein_coding	chr10:61901684-6	8760	1.128551015	1.638437153	0.452016	1E-06	3E-06
SMG6	SMG6 nonsense mediated mRNA decay fac	protein_coding	chr17:2059839-23	10568	4.338573663	5.547484924	0.268275	1E-06	3E-06
TCEAL3	transcription elongation factor A like 3	protein_coding	chrX:103607451-1	2305	4.481765276	6.512621852	0.449972	1E-06	3E-06
SNRPA	small nuclear ribonucleoprotein polypep	protein_coding	chr19:40750637-4	2353	13.67187064	19.0357238	0.393412	1E-06	3E-06
KATNA1	katanin catalytic subunit A1	protein_coding	chr6:149594873-1	2255	5.399196765	7.635814609	0.410328	1E-06	3E-06
ASB8	ankyrin repeat and SOCS box containing 8	protein_coding	chr12:48147789-4	4086	9.940722934	12.9187645	0.2924	1E-06	3E-06
CREM	cAMP responsive element modulator	protein_coding	chr10:35126791-3	5699	9.990448289	13.12086483	0.307815	1E-06	3E-06
ZNF626	zinc finger protein 626	protein_coding	chr19:20619939-2	6835	0.860015074	1.380421703	0.59293	1E-06	3E-06
CALCOCO2	calcium binding and coiled-coil domain 2	protein_coding	chr17:48830988-4	8333	3.485554577	4.59423533	0.311454	1E-06	3E-06
NT5C	5', 3'-nucleotidase, cytosolic	protein_coding	chr17:75130225-7	1533	20.50169908	27.61302036	0.345786	1E-06	3E-06
ARHGEF25	Rho guanine nucleotide exchange factor 2	protein_coding	chr12:57610180-5	3391	0.553790454	1.063930365	0.874537	1E-06	3E-06
HMGCL	3-hydroxy-3-methylglutaryl-CoA lyase	protein_coding	chr1:23801885-23	5628	3.833168782	5.294398845	0.378421	1E-06	4E-06
PDCD11	programmed cell death 11	protein_coding	chr10:103396626-	7672	6.075532839	8.120598698	0.334136	1E-06	4E-06
ZFP82	ZFP82 zinc finger protein	protein_coding	chr19:36383120-3	7506	0.642937333	1.028388504	0.598051	1E-06	4E-06
STOML1	stomatin like 1	protein_coding	chr15:73978926-7	10458	0.724119968	1.05659469	0.45393	1E-06	4E-06
FAM126A	family with sequence similarity 126 meml	protein_coding	chr7:22889371-23	14499	2.572975941	3.309297332	0.276852	1E-06	4E-06
POLB	DNA polymerase beta	protein_coding	chr8:42338454-42	4106	3.094921549	4.307967041	0.388762	1E-06	4E-06
KIAA1147	KIAA1147	protein_coding	chr7:141656728-1	7369	2.183141448	3.012986559	0.376158	1E-06	4E-06
MRPL16	mitochondrial ribosomal protein L16	protein_coding	chr11:59806140-5	2172	21.29551696	28.98652079	0.361202	1E-06	4E-06
C1orf35	chromosome 1 open reading frame 35	protein_coding	chr1:228100726-2	4308	3.258614784	4.619599185	0.414234	1E-06	4E-06
MAGEH1	MAGE family member H1	protein_coding	chrX:55452105-55	1462	13.38801055	18.72092746	0.396645	1E-06	4E-06
AIG1	androgen induced 1	protein_coding	chr6:143060496-1	7677	5.446349232	7.336329621	0.341869	1E-06	4E-06
MESP1	mesoderm posterior bHLH transcription f	protein_coding	chr15:89748661-8	2369	0.91920046	1.630044955	0.741232	1E-06	4E-06
PARS2	prolyl-tRNA synthetase 2, mitochondrial	protein_coding	chr1:54756898-54	2356	3.084419007	4.642704488	0.500885	1E-06	4E-06
ZBTB45	zinc finger and BTB domain containing 45	protein_coding	chr19:58513530-5	3006	6.110409801	8.324330265	0.356255	1E-06	4E-06
GLS	glutaminase	protein_coding	chr2:190880827-1	9827	14.31315066	19.06910776	0.327993	1E-06	4E-06
ERI2	ERI1 exoribonuclease family member 2	protein_coding	chr16:20780193-2	6761	2.147022586	3.006863469	0.398879	1E-06	4E-06
SLC29A2	solute carrier family 29 member 2	protein_coding	chr11:66362521-6	2720	25.98663654	34.06572711	0.305386	1E-06	4E-06
NRAV	negative regulator of antiviral response	lncRNA	chr12:120488079-	4156	3.170022172	4.345372699	0.367684	1E-06	4E-06
MRPL28	mitochondrial ribosomal protein L28	protein_coding	chr16:366969-371	3042	26.65438199	34.47964804	0.286263	1E-06	4E-06
SLC25A23	solute carrier family 25 member 23	protein_coding	chr19:6436079-64	4881	36.33089014	46.90234678	0.2828	1E-06	4E-06
HSBP1L1	heat shock factor binding protein 1 like 1	protein_coding	chr18:79964582-7	2035	0.554599892	1.288341372	1.131166	1E-06	4E-06
ENOPH1	enolase-phosphatase 1	protein_coding	chr4:82430590-82	2083	14.83307417	19.20152804	0.287757	1E-06	4E-06
ARMCX5	armadillo repeat containing X-linked 5	protein_coding	chrX:102599168-1	4911	1.826671925	2.694090625	0.472867	1E-06	4E-06
OXSM	3-oxoacyl-ACP synthase, mitochondrial	protein_coding	chr3:25782917-25	2476	3.045745498	4.330496992	0.421188	1E-06	4E-06
ACTR6	actin related protein 6	protein_coding	chr12:100199122-	3813	3.377929983	4.732863094	0.398085	1E-06	4E-06
CTSH	cathepsin H	protein_coding	chr15:78921058-7	5892	0.968472284	1.576974353	0.616036	1E-06	4E-06
ZNF251	zinc finger protein 251	protein_coding	chr8:144720907-1	5045	3.782778355	5.161416666	0.364713	1E-06	4E-06
IER5	immediate early response 5	protein_coding	chr1:181088712-1	4188	13.59239758	18.82143258	0.381104	1E-06	4E-06
SNTB2	syntrophin beta 2	protein_coding	chr16:69187129-6	9875	1.950165292	2.711169513	0.390277	1E-06	4E-06
TEAD2	TEA domain transcription factor 2	protein_coding	chr19:49340595-4	2980	22.60381775	29.13411965	0.281241	1E-06	4E-06
MIXL1	Mix paired-like homeobox	protein_coding	chr1:226223618-2	2029	0.391585134	1.189745253	1.567789	1E-06	4E-06
HEBP2	heme binding protein 2	protein_coding	chr6:138403531-1	10352	3.652325098	4.673807407	0.269433	1E-06	4E-06
SLC45A4	solute carrier family 45 member 4	protein_coding	chr8:141207166-1	8608	1.427848666	2.172683999	0.528692	1E-06	4E-06
RAD17	RAD17 checkpoint clamp loader compon	protein_coding	chr5:69369293-69	4159	9.019792965	11.70973387	0.289554	1E-06	4E-06
TDP2	tyrosyl-DNA phosphodiesterase 2	protein_coding	chr6:24649979-24	2977	12.53352173	16.3368798	0.296723	2E-06	4E-06
NFKBIL1	NFKB inhibitor like 1	protein_coding	chr6:31546870-31	1752	8.914659441	12.0524097	0.346589	2E-06	4E-06
CCDC28B	coiled-coil domain containing 28B	protein_coding	chr1:32200386-32	2818	6.502034938	9.051666818	0.390834	2E-06	4E-06
NID2	nidogen 2	protein_coding	chr14:52004803-5	6392	31.13408138	42.63904869	0.370227	2E-06	4E-06
BNIP1	BCL2 interacting protein 1	protein_coding	chr5:173144442-1	2440	3.816853039	5.788961458	0.510262	2E-06	4E-06
AL050341.2	novel transcript, antisense to ZMPSTE24	lncRNA	chr1:40256427-40	1541	2.723947363	4.32247434	0.575942	2E-06	4E-06
RFXANK	regulatory factor X associated ankyrin cor	protein_coding	chr19:19192229-1	2503	11.74399671	15.5919154	0.32126	2E-06	4E-06
AC067852.2	novel transcript, antisense to HSD17B1	lncRNA	chr17:42552436-4	2313	7.475771301	10.09758641	0.347615	2E-06	4E-06
GRK3	G protein-coupled receptor kinase 3	protein_coding	chr22:25564675-2	9477	1.781601959	2.405996223	0.346319	2E-06	4E-06
KIAA0355	KIAA0355	protein_coding	chr19:34254537-3	7459	5.616526974	7.630143099	0.356507	2E-06	4E-06
ZNF529-AS1	ZNF529 antisense RNA 1	lncRNA	chr19:36573070-3	3657	0.685491831	1.262945702	0.787015	2E-06	4E-06
FNDC4	fibronectin type III domain containing 4	protein_coding	chr2:27491883-27	1937	2.37611528	3.707230281	0.555186	2E-06	4E-06
ZNF677	zinc finger protein 677	protein_coding	chr19:53235381-5	9348	1.216521051	1.720008786	0.410838	2E-06	4E-06
POLL	DNA polymerase lambda	protein_coding	chr10:101578882-	5420	4.99318188	6.420977621	0.275551	2E-06	4E-06
MRPS23	mitochondrial ribosomal protein S23	protein_coding	chr17:57834781-5	6379	8.237575189	11.03467703	0.335257	2E-06	4E-06

MRM1	mitochondrial rRNA methyltransferase 1	protein_coding	chr17:36601583-3	1839	4.64463225	6.858638805	0.484428	2E-06	4E-06
KLHL25	kelch like family member 25	protein_coding	chr15:85759326-8	7111	2.979206485	3.977761357	0.331663	2E-06	4E-06
LINC01139	long intergenic non-protein coding RNA 1	lncRNA	chr1:238476542-2	5965	0.991599169	1.46512481	0.474508	2E-06	4E-06
ERG28	ergosterol biosynthesis 28 homolog	protein_coding	chr14:75649791-7	2633	6.420262009	8.688934399	0.350666	2E-06	4E-06
H6PD	hexose-6-phosphate dehydrogenase/gluc	protein_coding	chr1:9234775-927	9321	10.59541737	14.48196731	0.368224	2E-06	4E-06
DNAJC28	DnaJ heat shock protein family (Hsp40) m	protein_coding	chr21:33485530-3	2484	1.578795982	2.521706765	0.586547	2E-06	4E-06
NAT9	N-acetyltransferase 9 (putative)	protein_coding	chr17:74770547-7	4049	4.935641885	6.61691187	0.335692	2E-06	5E-06
C12orf76	chromosome 12 open reading frame 76	protein_coding	chr12:110027028-	5061	1.053016872	1.57686059	0.492824	2E-06	5E-06
HRAS	HRas proto-oncogene, GTPase	protein_coding	chr11:532242-537	1880	17.78477027	22.8031806	0.272296	2E-06	5E-06
MOV10	Mov10 RISC complex RNA helicase	protein_coding	chr1:112673141-1	6509	6.586706753	8.874240202	0.345982	2E-06	5E-06
TNFRSF21	TNF receptor superfamily member 21	protein_coding	chr6:47231532-47	3595	11.99951258	15.90280967	0.320254	2E-06	5E-06
LLGL1	LLGL scribble cell polarity complex comp	protein_coding	chr17:18225635-1	6590	8.296187987	10.59425787	0.267374	2E-06	5E-06
GEMIN5	gem nuclear organelle associated protein	protein_coding	chr5:154887411-1	5644	5.894815561	7.705901311	0.300848	2E-06	5E-06
TRAPPC13	trafficking protein particle complex 13	protein_coding	chr5:65625004-65	6761	3.658918144	4.694857827	0.273216	2E-06	5E-06
PIAS4	protein inhibitor of activated STAT 4	protein_coding	chr19:4007736-40	3556	7.730962878	10.14978361	0.303915	2E-06	5E-06
MED21	mediator complex subunit 21	protein_coding	chr12:27022546-2	3632	6.333029529	8.423814212	0.324303	2E-06	5E-06
TMEM147-AS1	TMEM147 antisense RNA 1	lncRNA	chr19:35540738-3	4919	4.614721432	6.369082115	0.379594	2E-06	5E-06
SEMA5B	semaphorin 5B	protein_coding	chr3:122909082-1	8517	2.992265576	4.422900384	0.483461	2E-06	5E-06
INPP5F	inositol polyphosphate-5-phosphatase F	protein_coding	chr10:119726042-	7479	2.482814714	3.460511645	0.395375	2E-06	5E-06
RFNG	RFNG O-fucosylpeptide 3-beta-N-acetylglu	protein_coding	chr17:82047902-8	3865	6.581269736	8.531909879	0.288383	2E-06	5E-06
GTF2H5	general transcription factor IIH subunit 5	protein_coding	chr6:158168350-1	7565	2.892635725	3.994335151	0.378681	2E-06	5E-06
PPP1CB	protein phosphatase 1 catalytic subunit b	protein_coding	chr2:28751640-28	5760	55.03362529	71.20333923	0.287259	2E-06	5E-06
AL117335.1	novel transcript, antisense to SIRPA	lncRNA	chr20:1888128-18	1607	4.602092883	6.919855411	0.503252	2E-06	5E-06
SERTAD2	SERTA domain containing 2	protein_coding	chr2:64631621-64	6274	9.289671907	12.05141809	0.290555	2E-06	5E-06
HTR4	5-hydroxytryptamine receptor 4	protein_coding	chr5:148451032-1	7045	0.685394536	1.102968561	0.592088	2E-06	5E-06
SPATA2L	spermatogenesis associated 2 like	protein_coding	chr16:89696357-8	2468	1.792677852	2.958535877	0.630205	2E-06	5E-06
UNC93B1	unc-93 homolog B1, TLR signaling regulat	protein_coding	chr11:67991100-6	3536	0.760368903	1.281313474	0.66487	2E-06	5E-06
AC104170.1	novel transcript	lncRNA	chr1:51518309-51	476	0.837965521	2.7278038	1.601292	2E-06	5E-06
PHTF1	putative homeodomain transcription fact	protein_coding	chr1:113696831-1	7170	4.825544882	6.178715984	0.272092	2E-06	5E-06
CLEC11A	C-type lectin domain containing 11A	protein_coding	chr19:50723364-5	1434	4.100762634	6.686856029	0.62205	2E-06	5E-06
PIK3CD-AS2	PIK3CD antisense RNA 2	lncRNA	chr1:9672426-968	939	0.658393195	1.607250488	1.172519	2E-06	5E-06
SESTD1	SEC14 and spectrin domain containing 1	protein_coding	chr2:179101678-1	11405	1.965455549	2.604745974	0.319762	2E-06	5E-06
NRBP2	nuclear receptor binding protein 2	protein_coding	chr8:143833583-1	4934	13.96128438	20.08243378	0.44042	2E-06	5E-06
ANKRD34A	ankyrin repeat domain 34A	protein_coding	chr1:145959441-1	3671	2.768618054	4.121953123	0.492162	2E-06	5E-06
ANKRD16	ankyrin repeat domain 16	protein_coding	chr10:5861616-58 3060		2.150968702	3.176385243	0.476161	2E-06	5E-06
PAFAH1B3	platelet activating factor acetylhydrolase	protein_coding	chr19:42297033-4	1419	9.869084226	14.18341007	0.439628	2E-06	5E-06
DDB2	damage specific DNA binding protein 2	protein_coding	chr11:47214465-4	4897	5.834501489	7.558655004	0.287462	2E-06	5E-06
ZBED8	zinc finger BED-type containing 8	protein_coding	chr5:160393148-1	2882	1.984807153	3.046360513	0.535798	2E-06	5E-06
PCIF1	PDX1 C-terminal inhibiting factor 1	protein_coding	chr20:45934683-4	3164	15.36557343	19.7992212	0.282044	2E-06	5E-06
AC006504.5	novel transcript	lncRNA	chr19:27793431-2	14163	0.755268926	1.150404707	0.534503	2E-06	5E-06
ERO1A	endoplasmic reticulum oxidoreductase 1	protein_coding	chr14:52639915-5	7549	2.697390581	3.864484371	0.434089	2E-06	5E-06
SNX33	sorting nexin 33	protein_coding	chr15:75647906-7	8008	1.262383466	1.943685236	0.530146	2E-06	5E-06
HHLA3	HERV-H LTR-associating 3	protein_coding	chr1:70354805-70	1222	4.850080114	7.044499542	0.45402	2E-06	6E-06
PTPMT1	protein tyrosine phosphatase mitochond	protein_coding	chr11:47565430-4	2740	0.901779832	1.546255138	0.691448	2E-06	6E-06
CFDP1	craniofacial development protein 1	protein_coding	chr16:75293698-7	3335	5.658080086	7.429056962	0.30585	2E-06	6E-06
DOP1A	DOP1 leucine zipper like protein A	protein_coding	chr6:83067666-83	10045	6.103203157	7.805089707	0.269467	2E-06	6E-06
TFB2M	transcription factor B2, mitochondrial	protein_coding	chr1:246540561-2	1784	5.71333173	8.43452594	0.475479	2E-06	6E-06
KRT19	keratin 19	protein_coding	chr17:41523617-4	2336	1.068294997	2.073711107	0.866665	2E-06	6E-06
METTL6	methyltransferase like 6	protein_coding	chr3:15381275-15	5714	1.404736669	1.995814312	0.415229	2E-06	6E-06
HECA	hdc homolog, cell cycle regulator	protein_coding	chr6:139135080-1	5646	14.42062009	18.58657585	0.280007	2E-06	6E-06
SACS	sacsin molecular chaperone	protein_coding	chr13:23328826-2	15918	5.460903652	7.694723046	0.413762	2E-06	6E-06
AC008443.5	novel transcript	lncRNA	chr5:181191875-1	535	4.647458568	7.791374472	0.657086	2E-06	6E-06
ZNF83	zinc finger protein 83	protein_coding	chr19:52594060-5	8028	4.101907747	5.358694815	0.301602	2E-06	6E-06
DGKQ	diacylglycerol kinase theta	protein_coding	chr4:958887-9868	4983	2.841163326	3.96033092	0.394672	2E-06	6E-06
DEPP1	DEPP1 autophagy regulator	protein_coding	chr10:44970981-4	2979	5.457697271	7.803878323	0.436542	2E-06	6E-06
AKAP7	A-kinase anchoring protein 7	protein_coding	chr6:131135467-1	4593	1.780551217	2.581549863	0.44883	2E-06	6E-06
GOLGA5	golgin A5	protein_coding	chr14:92794305-9	3205	8.150807577	10.66287867	0.30057	2E-06	6E-06
LRRC27	leucine rich repeat containing 27	protein_coding	chr10:132332154-	11500	0.677649441	0.980512469	0.442261	2E-06	6E-06
SLC25A15	solute carrier family 25 member 15	protein_coding	chr13:40789412-4	4186	5.629980235	7.304136015	0.289507	2E-06	6E-06
PLAG1	PLAG1 zinc finger	protein_coding	chr8:56160909-56	7828	3.512935903	4.530883464	0.279367	2E-06	6E-06
EID2B	EP300 interacting inhibitor of differentiat	protein_coding	chr19:39530990-3	1865	2.799090406	4.477276511	0.582074	2E-06	6E-06
USP27X	ubiquitin specific peptidase 27 X-linked	protein_coding	chrX:49879948-49	2618	1.088121863	1.812377779	0.643297	2E-06	6E-06
PRDM15	PR/SET domain 15	protein_coding	chr21:41798225-4	13004	0.712906503	0.975387054	0.364901	2E-06	6E-06
PPP1R1A	protein phosphatase 1 regulatory inhibito	protein_coding	chr12:54575387-5	3984	38.60766774	49.41340084	0.270469	2E-06	6E-06
ARHGEF18	Rho/Rac guanine nucleotide exchange fac	protein_coding	chr19:7395113-74	6242	1.590828786	2.293000697	0.440595	2E-06	6E-06
RPS27A	ribosomal protein S27a	protein_coding	chr2:55231903-55	3130	37.28155373	50.77393778	0.358257	2E-06	6E-06
REX1BD	required for excision 1-B domain containi	protein_coding	chr19:18588685-1	3370	8.444156048	11.19799178	0.319194	2E-06	6E-06
FGFBP3	fibroblast growth factor binding protein 3	protein_coding	chr10:91906589-9	2545	0.794512385	1.563663789	0.870528	2E-06	6E-06
NKIRAS1	NFKB inhibitor interacting Ras like 1	protein_coding	chr3:23891660-23	3473	6.532472456	8.692435648	0.325253	2E-06	6E-06
SMAD1	SMAD family member 1	protein_coding	chr4:145481194-1	7094	3.437075742	4.444590045	0.286227	2E-06	6E-06
FOXN3	forkhead box N3	protein_coding	chr14:89124871-8	11033	8.634925392	11.23936769	0.2952	2E-06	6E-06
ZFP62	ZFP62 zinc finger protein	protein_coding	chr5:180847611-1	4197	3.658736424	4.895195672	0.331641	2E-06	6E-06
CEP170B	centrosomal protein 170B	protein_coding	chr14:104865268-	7211	11.58643887	15.1909738	0.306661	3E-06	7E-06
AC129492.1	period circadian regulator 1	protein_coding	chr17:8140472-81	7002	6.189477116	8.483783996	0.371219	3E-06	7E-06
SMYD3	SET and MYND domain containing 3	protein_coding	chr1:245749342-2	5866	1.474796809	2.179972692	0.477019	3E-06	7E-06
DYNC2H1	dynein cytoplasmic 2 heavy chain 1	protein_coding	chr11:103109410-	15012	1.000496683	1.498314896	0.499509	3E-06	7E-06
RSBN1	round spermatid basic protein 1	protein_coding	chr1:113761832-1	6819	2.966376911	3.950113278	0.326302	3E-06	7E-06
CCDC181	coiled-coil domain containing 181	protein_coding	chr1:169394870-1	3961	0.827350751	1.358725761	0.618714	3E-06	7E-06
ZNF879	zinc finger protein 879	protein_coding	chr5:179023752-1	3883	0.632062048	1.155192551	0.802217	3E-06	7E-06
RTCA-AS1	RTCA antisense RNA 1	lncRNA	chr1:100251528-1	10034	0.778905107	1.157856014	0.486176	3E-06	7E-06
ANKRD12	ankyrin repeat domain 12	protein_coding	chr18:9136228-92	12248	5.171980798	6.629135116	0.272737	3E-06	7E-06
GTF2F2	general transcription factor IIF subunit 2	protein_coding	chr13:45120510-4	3608	7.920568832	10.35361516	0.299386	3E-06	7E-06
TTC14	tetratricopeptide repeat domain 14	protein_coding	chr3:180602163-1	7900	4.378420215	5.67548715	0.288449	3E-06	7E-06
IQSEC2	IQ motif and Sec7 domain 2	protein_coding	chrX:53225828-53	13115	1.082231708	1.508133066	0.396833	3E-06	7E-06
AKAP17A	A-kinase anchoring protein 17A	protein_coding	chrX:1591604-160 4070		12.42766675	15.87499303	0.267969	3E-06	7E-06
PTK2B	protein tyrosine kinase 2 beta	protein_coding	chr8:27311482-27	7860	0.640188298	0.99342848	0.547082	3E-06	7E-06
TULP3	tubby like protein 3	protein_coding	chr12:2877223-29	3179	4.592491119	6.211391318	0.350057	3E-06	7E-06
HDHD5	haloacid dehalogenase like hydrolase dom	protein_coding	chr22:17137511-1	4936	7.731784888	9.947017399	0.279397	3E-06	7E-06
USP20	ubiquitin specific peptidase 20	protein_coding	chr9:129834698-1	5521	4.96996704	6.703317414	0.347686	3E-06	7E-06
HDDC3	HD domain containing 3	protein_coding	chr15:90929964-9	5233	1.891551858	2.725857052	0.445628	3E-06	7E-06
KAT6B	lysine acetyltransferase 6B	protein_coding	chr10:74824927-7	32495	1.687224722	2.169030907	0.276943	3E-06	7E-06
NUBPL	nucleotide binding protein like	protein_coding	chr14:31489956-3	7590	4.956499289	6.59719168	0.324039	3E-06	7E-06
AC074212.1	novel transcript, antisense to SIX5	lncRNA	chr19:45764785-4	2600	1.590540066	2.6335861	0.638512	3E-06	7E-06
LINC01144	long intergenic non-protein coding RNA 1	lncRNA	chr1:45303910-45	1710	0.268730239	0.826431932	1.541376	3E-06	7E-06
RAB34	RAB34, member RAS oncogene family	protein_coding	chr17:28714281-2	3303	18.44074296	23.83045016	0.283923	3E-06	7E-06
SRSF3	serine and arginine rich splicing factor 3	protein_coding	chr6:36594353-36	7118	17.36064942	22.22590948	0.270864	3E-06	7E-06
TOR3A	torsin family 3 member A	protein_coding	chr1:179082070-1	3224	4.084557413	5.370218367	0.309378	3E-06	7E-06
TRIM45	tripartite motif containing 45	protein_coding	chr1:117111060-1	4368	1.251320248	2.015960268	0.604063	3E-06	7E-06
AC012360.3	novel transcript	lncRNA	chr2:105334027-1	3449	0.61617286	1.119019653	0.786103	3E-06	7E-06
TRIM17	tripartite motif containing 17	protein_coding	chr1:228407935-2	3553	0.674145607	1.191963981	0.737289	3E-06	8E-06
TRMT112	tRNA methyltransferase subunit 11-2	protein_coding	chr11:64316460-6	1625	21.79740635	31.07401248	0.428917	3E-06	8E-06
LINC00663	long intergenic non-protein coding RNA 6	lncRNA	chr19:19757366-1	4334	0.478069065	1.018356855	1.006322	3E-06	8E-06
SPTY2D1	SPT2 chromatin protein domain containi	protein_coding	chr11:18606403-1	5899	4.608664928	6.005583838	0.294446	3E-06	8E-06
PEX2	peroxisomal biogenesis factor 2	protein_coding	chr8:76980258-77	4969	6.404295537	8.536333377	0.326933	3E-06	8E-06
AC004086.1	ribosomal protein L6	protein_coding	chr12:112405190-	4447	60.6860273	81.74974036	0.346296	3E-06	8E-06
ENPP1	ectonucleotide pyrophosphatase/phosph	protein_coding	chr6:131808016-1	9959	1.211680703	1.659953493	0.366322	3E-06	8E-06
CWC27	CWC27 spliceosome associated protein h	protein_coding	chr5:64768930-65	3727	7.739264061	10.52159678	0.358686	3E-06	8E-06
ZNF239	zinc finger protein 239	protein_coding	chr10:43556344-4	2509	1.859424296	2.825449494	0.522432	3E-06	8E-06
GALNT7	polypeptide N-acetylgalactosaminyltrans	protein_coding	chr4:173168811-1	6807	2.26530474	2.958610113	0.298202	3E-06	8E-06
KIDINS220	kinase D interacting substrate 220	protein_coding	chr2:8721081-883	15362	17.20189359	22.28966211	0.289276	3E-06	8E-06
AC007566.1	novel transcript, antisense to ERVW-1 and	lncRNA	chr7:92457564-92	5690	2.970345031	4.049727619	0.362044	3E-06	8E-06

PHLPP2	PH domain and leucine rich repeat protei	protein_coding	chr16:71637835-7	12493	1.210724354	1.744179003	0.440121	3E-06	8E-06
RHPN2	rhophilin Rho GTPase binding protein 2	protein_coding	chr19:32978592-3	4805	2.052885218	2.822504361	0.373627	3E-06	8E-06
PCGF6	polycomb group ring finger 6	protein_coding	chr10:103302796-	3008	5.287606602	7.001869538	0.319573	3E-06	8E-06
MALSU1	mitochondrial assembly of ribosomal larg	protein_coding	chr7:23298739-23	3851	5.31755729	7.484671835	0.409942	3E-06	8E-06
ZNF783	zinc finger family member 783	protein_coding	chr7:149262171-1	6723	1.459303343	2.187365943	0.493169	3E-06	8E-06
PM20D2	peptidase M20 domain containing 2	protein_coding	chr6:89146055-89	4703	3.788293982	5.096508798	0.340093	3E-06	8E-06
COX18	cytochrome c oxidase assembly factor CO:	protein_coding	chr4:73052362-73	6976	4.38189165	5.80146057	0.32007	3E-06	8E-06
HTR1B	5-hydroxytryptamine receptor 1B	protein_coding	chr6:77461753-77	2021	1.475711257	2.46513375	0.658677	3E-06	8E-06
ARFRP1	ADP ribosylation factor related protein 1	protein_coding	chr20:63698642-6	7627	5.69653234	7.365806594	0.283907	3E-06	8E-06
AC093627.5	novel transcript	lncRNA	chr7:135853-1495	5013	0.580536429	0.986306617	0.674288	3E-06	8E-06
FUNDC2	FUN14 domain containing 2	protein_coding	chrX:155025980-1	10094	6.085026593	8.082986616	0.325572	3E-06	8E-06
ZNF800	zinc finger protein 800	protein_coding	chr7:127346790-1	5793	2.214221733	3.10798994	0.39827	3E-06	8E-06
AC090204.1	novel transcript	lncRNA	chr8:32927913-33	1791	8.440387038	11.33792678	0.344257	3E-06	8E-06
DYRK4	dual specificity tyrosine phosphorylation	protein_coding	chr12:4562204-46	7054	2.170779079	3.002219197	0.384252	3E-06	8E-06
SNRPB2	small nuclear ribonucleoprotein polypep	protein_coding	chr20:16729961-1	2608	13.80252898	18.93442363	0.371241	3E-06	8E-06
TMEM68	transmembrane protein 68	protein_coding	chr8:55696424-55	8574	1.37496101	2.05477905	0.49342	3E-06	8E-06
DPYSL2	dihydropyrimidinase like 2	protein_coding	chr8:26514031-26	7296	2.083745893	2.723176023	0.301108	3E-06	8E-06
RBBP9	RB binding protein 9, serine hydrolase	protein_coding	chr20:18486540-1	3843	4.419317986	5.814239631	0.309745	3E-06	9E-06
LINC01011	long intergenic non-protein coding RNA 1	lncRNA	chr6:2987967-299	2811	0.389986028	0.934116652	1.152699	3E-06	9E-06
SCRIB	scribble planar cell polarity protein	protein_coding	chr8:143790920-1	6586	14.97362648	19.38408394	0.287003	3E-06	9E-06
SFI1	SFI1 centrin binding protein	protein_coding	chr22:31488688-3	12174	0.675525038	0.978692698	0.449746	3E-06	9E-06
ZKSCAN1	zinc finger with KRAB and SCAN domains 1	protein_coding	chr7:100015572-1	10319	8.775222958	11.27255812	0.276084	3E-06	9E-06
SDK1	sidekick cell adhesion molecule 1	protein_coding	chr7:3301252-426	12258	1.933027667	2.759535548	0.43748	3E-06	9E-06
MTAP	methylthioadenosine phosphorylase	protein_coding	chr9:21802636-21	14527	0.879013506	1.156301159	0.310736	3E-06	9E-06
MPLKIP	M-phase specific PLK1 interacting protein	protein_coding	chr7:40126023-40	7661	2.458566815	3.39610899	0.374482	4E-06	9E-06
OCIAD2	OCIA domain containing 2	protein_coding	chr4:48885019-48	2483	3.718268544	5.188857172	0.38991	4E-06	9E-06
RAD9B	RAD9 checkpoint clamp component B	protein_coding	chr12:110501655-	3178	0.573186468	1.036647517	0.773372	4E-06	9E-06
SLC9A6	solute carrier family 9 member A6	protein_coding	chrX:135973841-1	7465	2.832947153	3.634456507	0.271597	4E-06	9E-06
ZMAT2	zinc finger matrin-type 2	protein_coding	chr5:140698680-1	1812	21.10129839	27.00239548	0.271187	4E-06	9E-06
UBXN8	UBX domain protein 8	protein_coding	chr8:30732247-30	3218	0.650565817	1.112576236	0.682002	4E-06	9E-06
ZNF549	zinc finger protein 549	protein_coding	chr19:57527325-5	9279	0.695789249	1.036778728	0.493227	4E-06	9E-06
HR	HR lysine demethylase and nuclear recept	protein_coding	chr8:22114419-22	7964	1.261086237	1.85463455	0.485163	4E-06	9E-06
VEGFC	vascular endothelial growth factor C	protein_coding	chr4:176683538-1	2615	1.746377557	2.684831735	0.536349	4E-06	9E-06
AP003068.2	novel transcript, antisense to CAPN1	lncRNA	chr11:65177606-6	1791	2.679141554	4.311657469	0.610999	4E-06	9E-06
ADGRD1	adhesion G protein-coupled receptor D1	protein_coding	chr12:130953907-	14718	4.23752819	5.531786628	0.299716	4E-06	9E-06
AL512625.4	novel transcript	lncRNA	chr9:62801534-62	823	1.692738689	3.650565956	1.00365	4E-06	9E-06
AC079848.1	novel transcript	lncRNA	chr3:126083659-1	1656	0.772707203	1.53796937	0.898003	4E-06	9E-06
BABAM2	BRISC and BRCA1 A complex member 2	protein_coding	chr2:27889941-28	3392	7.764160001	10.22427439	0.308687	4E-06	9E-06
TBC1D23	TBC1 domain family member 23	protein_coding	chr3:100260992-1	5176	5.078942587	6.477237951	0.263053	4E-06	9E-06
MFSD8	major facilitator superfamily domain con	protein_coding	chr4:127917732-1	9103	2.261204289	2.958710871	0.300978	4E-06	9E-06
ZC3HAV1	zinc finger CCCH-type containing, antivira	protein_coding	chr7:139043515-1	8241	1.287813885	1.751121497	0.358061	4E-06	9E-06
IGF2R	insulin like growth factor 2 receptor	protein_coding	chr6:159969099-1	17843	15.82643747	20.47460818	0.286736	4E-06	9E-06
RNF6	ring finger protein 6	protein_coding	chr13:26132115-2	4442	7.600356644	9.717180539	0.268693	4E-06	9E-06
VPS29	VPS29 retromer complex component	protein_coding	chr12:110491097-	3211	13.56747113	17.76923924	0.304861	4E-06	1E-05
C19orf53	chromosome 19 open reading frame 53	protein_coding	chr19:13774456-1	1197	39.4964195	54.40720851	0.379014	4E-06	1E-05
HERPUD1	homocysteine inducible ER protein with u	protein_coding	chr16:56932142-5	5062	7.576350604	9.949494077	0.309725	4E-06	1E-05
SNX27	sorting nexin family member 27	protein_coding	chr1:151612006-1	16711	2.522176921	3.224603776	0.267379	4E-06	1E-05
EIF3J-DT	EIF3J divergent transcript	lncRNA	chr15:44527257-4	3453	3.249855896	4.574486368	0.401778	4E-06	1E-05
AQP11	aquaporin 11	protein_coding	chr11:77589391-7	2396	0.65852557	1.204113541	0.774069	4E-06	1E-05
NADK2	NAD kinase 2, mitochondrial	protein_coding	chr5:36192589-36	5015	9.026440747	11.58008893	0.273553	4E-06	1E-05
CBLN3	cerebellin 3 precursor	protein_coding	chr14:24426532-2	2562	0.562488789	1.126372392	0.906014	4E-06	1E-05
PRPF3	pre-mRNA processing factor 3	protein_coding	chr1:150321479-1	5408	4.505212551	5.796268598	0.276044	4E-06	1E-05
AL021707.2	novel transcript, antisense to CBY1	lncRNA	chr22:38667585-3	4445	0.722778426	1.182730558	0.616018	4E-06	1E-05
UGDH-AS1	UGDH antisense RNA 1	lncRNA	chr4:39528019-39	2787	1.134289792	1.895675839	0.661491	4E-06	1E-05
RPS2	ribosomal protein S2	protein_coding	chr16:1962058-19	2388	263.6338888	340.1372021	0.282425	4E-06	1E-05
AC027307.2	novel transcript, antisense to APC2	lncRNA	chr19:1457670-14	911	4.512158987	6.946104921	0.532363	4E-06	1E-05
CNTRL	centriolin	protein_coding	chr9:121074863-1	9417	1.313168445	1.872692966	0.425789	4E-06	1E-05
TRIM8	tripartite motif containing 8	protein_coding	chr10:102642310-	9542	10.29183076	13.51471539	0.309044	4E-06	1E-05
EEF1A1	eukaryotic translation elongation factor 1	protein_coding	chr6:73515750-73	5474	629.6642638	820.7811028	0.29749	4E-06	1E-05
TOR1A	torsin family 1 member A	protein_coding	chr9:129812942-1	3998	5.070463346	6.633463514	0.303646	4E-06	1E-05
MSH3	mutS homolog 3	protein_coding	chr5:80654652-80	5329	4.291449003	5.589658312	0.295	4E-06	1E-05
SRR	serine racemase	protein_coding	chr17:2303383-23	3252	7.109927703	9.153986485	0.276406	4E-06	1E-05
RPL27A	ribosomal protein L27a	protein_coding	chr11:8682788-87	6816	24.32586556	32.5977782	0.338414	4E-06	1E-05
ASB16	ankyrin repeat and SOCS box containing 1	protein_coding	chr17:44170447-4	2230	2.852878298	4.296227876	0.513654	4E-06	1E-05
AL512625.2	novel transcript	lncRNA	chr9:62837139-62	1164	0.488337187	1.300218343	1.327541	4E-06	1E-05
DNPH1	2'-deoxynucleoside 5'-phosphate N-hydro	protein_coding	chr6:43225629-43	1250	23.79798628	32.77501811	0.371118	4E-06	1E-05
DHRS4	dehydrogenase/reductase 4	protein_coding	chr14:23953734-2	2157	9.819679487	14.08672174	0.435052	5E-06	1E-05
ROM1	retinal outer segment membrane protein	protein_coding	chr11:62611722-6	2495	0.526412311	1.110280449	1.025486	5E-06	1E-05
ZNF263	zinc finger protein 263	protein_coding	chr16:3263800-33	6403	2.712823668	3.506828616	0.283382	5E-06	1E-05
PAXIP1-AS1	PAXIP1 antisense RNA 1 (head to head)	lncRNA	chr7:155003448-1	2256	2.944371664	4.371928616	0.492307	5E-06	1E-05
TBK1	TANK binding kinase 1	protein_coding	chr12:64452092-6	6382	3.004325463	3.855411603	0.274425	5E-06	1E-05
ILDR2	immunoglobulin like domain containing	protein_coding	chr1:166895711-1	13946	1.391979379	1.989365577	0.429475	5E-06	1E-05
MIEN1	migration and invasion enhancer 1	protein_coding	chr17:39728496-3	2037	21.32820623	29.97748758	0.409888	5E-06	1E-05
AC020909.3	novel transcript	lncRNA	chr19:50486810-5	626	821.4863494	1244.824465	0.512083	5E-06	1E-05
ZNF326	zinc finger protein 326	protein_coding	chr1:89995110-90	10988	4.581805144	5.908471089	0.283674	5E-06	1E-05
SUFU	SUFU negative regulator of hedgehog sign	protein_coding	chr10:102503987-	6865	3.619831467	4.888261782	0.349257	5E-06	1E-05
TVP23B	trans-golgi network vesicle protein 23 ho	protein_coding	chr17:18781111-1	5746	1.695631435	2.334892907	0.372505	5E-06	1E-05
KRR1	KRR1 small subunit processome compone	protein_coding	chr12:75490863-7	10747	4.277531364	5.519473431	0.281467	5E-06	1E-05
SHANK3	SH3 and multiple ankyrin repeat domains	protein_coding	chr22:50674415-5	8167	3.757349824	5.325140075	0.4233	5E-06	1E-05
SNRNP40	small nuclear ribonucleoprotein U5 subu	protein_coding	chr1:31259568-31	3887	4.863245431	6.502639988	0.332363	5E-06	1E-05
TLK1	tousled like kinase 1	protein_coding	chr2:170990823-1	9405	4.111505308	5.253977625	0.269383	5E-06	1E-05
CSPG4	chondroitin sulfate proteoglycan 4	protein_coding	chr15:75674322-7	8290	2.641589614	3.794760119	0.441161	5E-06	1E-05
C2CD5	C2 calcium dependent domain containing	protein_coding	chr12:22448583-2	6759	4.702664417	6.229369025	0.322447	5E-06	1E-05
WDR7	WD repeat domain 7	protein_coding	chr18:56651343-5	15694	1.46005458	1.936875927	0.326656	5E-06	1E-05
PPP1R3E	protein phosphatase 1 regulatory subunit	protein_coding	chr14:23295643-2	5548	3.315851787	4.456333993	0.341453	5E-06	1E-05
NUDT6	nudix hydrolase 6	protein_coding	chr4:122888697-1	6591	1.443339087	1.983294622	0.373402	5E-06	1E-05
PIP4K2C	phosphatidylinositol-5-phosphate 4-kina	protein_coding	chr12:57591174-5	3299	6.271957833	8.36858123	0.331373	5E-06	1E-05
RCCD1	RCC1 domain containing 1	protein_coding	chr15:90954870-9	4969	2.972468202	3.902866784	0.307051	5E-06	1E-05
DAG1	dystroglycan 1	protein_coding	chr3:49468703-49	6923	51.67135166	66.00097705	0.268156	5E-06	1E-05
MAP1S	microtubule associated protein 1S	protein_coding	chr19:17719242-1	4933	7.362924729	9.514364361	0.283312	5E-06	1E-05
LINC00526	long intergenic non-protein coding RNA 5	lncRNA	chr18:5236724-52	1875	0.83246519	1.570145619	0.814686	5E-06	1E-05
EDEM3	ER degradation enhancing alpha-mannosi	protein_coding	chr1:184690237-1	7792	6.876415194	8.930936639	0.289198	5E-06	1E-05
STK26	serine/threonine kinase 26	protein_coding	chrX:132023302-1	3317	8.678980615	11.50510164	0.321285	5E-06	1E-05
RIOK2	RIO kinase 2	protein_coding	chr5:97160867-97	5489	4.06406588	5.364083762	0.314785	5E-06	1E-05
AC093297.2	novel transcript	lncRNA	chr5:44826076-44	2517	3.160920287	4.516974521	0.426485	5E-06	1E-05
CAPS	calcyphosine	protein_coding	chr19:5911375-59	4429	1.212913755	2.086501526	0.702436	5E-06	1E-05
FAM218A	family with sequence similarity 218 meml	protein_coding	chr4:164956948-1	2175	0.701653183	1.351805363	0.864917	5E-06	1E-05
RILPL2	Rab interacting lysosomal protein like 2	protein_coding	chr12:123410683-	6141	0.592032515	0.987139696	0.64096	5E-06	1E-05
AL137003.1	novel transcript	lncRNA	chr6:16761138-16	698	11.3734332	16.54065208	0.457951	5E-06	1E-05
HSPA1L	heat shock protein family A (Hsp70) meml	protein_coding	chr6:31809619-31	2762	1.841097283	2.685610735	0.458495	5E-06	1E-05
TK2	thymidine kinase 2	protein_coding	chr16:66508003-6	7169	2.402027086	3.25349785	0.349167	5E-06	1E-05
RPUSD2	RNA pseudouridine synthase domain cont	protein_coding	chr15:40569299-4	2489	4.61471505	6.148527357	0.326307	5E-06	1E-05
NFXL1	nuclear transcription factor, X-box bindir	protein_coding	chr4:47847233-47	4494	1.929267479	2.727866191	0.419342	5E-06	1E-05
VMA21	vacuolar ATPase assembly factor VMA21	protein_coding	chrX:151396515-1	5109	5.158095126	6.696985178	0.292273	5E-06	1E-05
ZFP2	ZFP2 zinc finger protein	protein_coding	chr5:178895898-1	2637	0.4517766	0.909170042	0.92716	6E-06	1E-05
ZNF517	zinc finger protein 517	protein_coding	chr8:144798876-1	5308	1.389400153	1.99036704	0.438144	6E-06	1E-05
AC004069.1	novel transcript	lncRNA	chr4:105137280-1	549	0.577851264	1.661871596	1.444345	6E-06	1E-05
BRMS1	BRMS1 transcriptional repressor and anoi	protein_coding	chr11:66337333-6	2514	7.78774632	10.07461832	0.286772	6E-06	1E-05
RND2	Rho family GTPase 2	protein_coding	chr17:43025231-4	4368	1.066945864	1.673367544	0.568033	6E-06	1E-05
RPGRIP1L	RPGRIP1 like	protein_coding	chr16:53598153-5	12387	0.821906485	1.123860282	0.367428	6E-06	1E-05
ARL2	ADP ribosylation factor like GTPase 2	protein_coding	chr11:65014160-6	2037	10.62089377	13.65898862	0.2792	6E-06	1E-05

DRD4	dopamine receptor D4	protein_coding	chr11:637293-640	1477	0.721834017	1.67533772	1.121253	6E-06	1E-05
AL132780.2	novel transcript, antisense to C14orf93	lncRNA	chr14:22982698-2	1462	1.413974202	2.37809043	0.656921	6E-06	1E-05
PCDHAC2	protocadherin alpha subfamily C, 2	protein_coding	chr5:140966470-1	6086	0.868039164	1.452426155	0.657516	6E-06	1E-05
NCEH1	neutral cholesterol ester hydrolase 1	protein_coding	chr3:172630249-1	4808	2.070933993	2.94551212	0.427715	6E-06	1E-05
SFT2D1	SFT2 domain containing 1	protein_coding	chr6:166319728-1	3089	5.267573272	7.377550937	0.400083	6E-06	1E-05
CIR1	corepressor interacting with RBPJ, 1	protein_coding	chr2:174348022-1	3178	6.06381361	8.09872518	0.332571	6E-06	1E-05
RSAD1	radical S-adenosyl methionine domain co	protein_coding	chr17:50478860-5	4137	7.35008197	9.431788324	0.276064	6E-06	1E-05
MIR4453HG	MIR4453 host gene	lncRNA	chr4:152536264-1	3000	0.696638968	1.27658068	0.781898	6E-06	1E-05
RPL9	ribosomal protein L9	protein_coding	chr4:39452521-39	5022	34.56234433	45.02631944	0.296356	6E-06	1E-05
PPP1R9A	protein phosphatase 1 regulatory subunit	protein_coding	chr7:94907202-95	11194	4.745230579	6.328878484	0.331323	6E-06	1E-05
FAT1	FAT atypical cadherin 1	protein_coding	chr4:186587794-1	16167	18.42083671	26.64073381	0.452794	6E-06	2E-05
SLC30A4	solute carrier family 30 member 4	protein_coding	chr15:45479606-4	8026	1.019785242	1.390627284	0.357695	6E-06	2E-05
PLCB3	phospholipase C beta 3	protein_coding	chr11:64251523-6	4964	1.974546742	2.646335059	0.334599	6E-06	2E-05
KCTD13	potassium channel tetramerization doma	protein_coding	chr16:29905012-2	6458	1.141153365	1.650519167	0.447723	6E-06	2E-05
SEPSECS	Sep (O-phosphoserine) tRNA:Sec (selenocy	protein_coding	chr4:25120014-25	6239	2.057786359	2.726104683	0.320419	6E-06	2E-05
RNF220	ring finger protein 220	protein_coding	chr1:44405194-44	9687	3.787834317	4.887126109	0.281112	7E-06	2E-05
CEP89	centrosomal protein 89	protein_coding	chr19:32875925-3	9233	1.345092696	1.757020175	0.301631	7E-06	2E-05
OLA1	Obg like ATPase 1	protein_coding	chr2:174072447-1	6001	14.70742475	18.86079946	0.273826	7E-06	2E-05
AVEN	apoptosis and caspase activation inhibito	protein_coding	chr15:33866227-3	1836	11.75640002	15.99698865	0.35265	7E-06	2E-05
SMIM20	small integral membrane protein 20	protein_coding	chr4:25861830-25	1612	11.27254139	15.48365197	0.374155	7E-06	2E-05
NMNAT3	nicotinamide nucleotide adenylyltransfer	protein_coding	chr3:139560180-1	12983	0.838649304	1.179594422	0.40962	7E-06	2E-05
AC147651.1	novel transcript	lncRNA	chr7:520391-5252	4085	2.260636965	3.235417524	0.431034	7E-06	2E-05
NUDT16L1	nudix hydrolase 16 like 1	protein_coding	chr16:4693694-46	2068	34.73587416	44.93063792	0.283632	7E-06	2E-05
TUBG2	tubulin gamma 2	protein_coding	chr17:42659284-4	2793	10.15250896	13.79714577	0.363533	7E-06	2E-05
C14orf93	chromosome 14 open reading frame 93	protein_coding	chr14:22985894-2	4257	1.258296366	1.82575644	0.451206	7E-06	2E-05
ALAD	aminolevulinate dehydratase	protein_coding	chr9:113386312-1	4945	5.387913735	6.958076741	0.281333	7E-06	2E-05
AC087500.1	novel transcript, antisense to C17orf87	lncRNA	chr17:5192027-52	4419	0.681096627	1.102756558	0.609665	7E-06	2E-05
CAMSAP3	calmodulin regulated spectrin associated	protein_coding	chr19:7595902-76	4845	1.417300754	2.057248722	0.449048	7E-06	2E-05
POMK	protein-O-mannose kinase	protein_coding	chr8:43093506-43	1877	1.328592983	2.340016615	0.720408	7E-06	2E-05
RERG	RAS like estrogen regulated growth inhibi	protein_coding	chr12:15107783-1	4157	0.698511772	1.14358774	0.632796	7E-06	2E-05
AC012184.3	novel transcript, antisense to DDX19B anc	lncRNA	chr16:70315442-7	1765	0.803701995	1.667934305	0.945369	7E-06	2E-05
RBFA	ribosome binding factor A	protein_coding	chr18:80034389-8	7945	0.972338591	1.350279944	0.389718	7E-06	2E-05
CDKN2D	cyclin dependent kinase inhibitor 2D	protein_coding	chr19:10566462-1	1503	5.453652645	7.667015554	0.40772	7E-06	2E-05
CDC42EP5	CDC42 effector protein 5	protein_coding	chr19:54465026-5	935	0.275566313	1.437216922	2.348382	7E-06	2E-05
TASP1	taspase 1	protein_coding	chr20:13389392-1	2897	2.04940743	2.965630801	0.443314	7E-06	2E-05
AC007541.1	novel transcript, antisense to C12orf23	lncRNA	chr12:106954029-	1469	2.570570768	3.793468602	0.475956	7E-06	2E-05
IRF7	interferon regulatory factor 7	protein_coding	chr11:612553-615	2612	0.502056044	1.132177211	1.069862	7E-06	2E-05
MPPED2	metallophosphoesterase domain containi	protein_coding	chr11:30384493-3	8197	2.632883519	3.475983631	0.315284	8E-06	2E-05
XKR6	XK related 6	protein_coding	chr8:10896045-11	5209	1.732406051	2.516983497	0.453508	8E-06	2E-05
VARS2	valyl-tRNA synthetase 2, mitochondrial	protein_coding	chr6:30908242-30	8448	3.372897129	4.323800951	0.274405	8E-06	2E-05
TRMT5	tRNA methyltransferase 5	protein_coding	chr14:60971451-6	5692	1.983729651	2.765451486	0.390639	8E-06	2E-05
RPS18	ribosomal protein S18	protein_coding	chr6:33272075-33	1834	162.0198765	211.1756916	0.297934	8E-06	2E-05
FBXL12	F-box and leucine rich repeat protein 12	protein_coding	chr19:9810267-98	2913	7.642021838	10.10343164	0.32097	8E-06	2E-05
RAE1	ribonucleic acid export 1	protein_coding	chr20:57351223-5	5642	4.572135932	5.994646555	0.301963	8E-06	2E-05
ZNF614	zinc finger protein 614	protein_coding	chr19:52012765-5	5623	2.370819388	3.186492546	0.339094	8E-06	2E-05
RAB6B	RAB6B, member RAS oncogene family	protein_coding	chr3:133824235-1	6055	4.08989657	5.397876598	0.313658	8E-06	2E-05
AC104083.1	novel transcript, overlapping GUCY1A3	lncRNA	chr4:155734448-1	2615	12.86805442	16.45531605	0.266891	8E-06	2E-05
DNAJA4	DnaJ heat shock protein family (Hsp40) m	protein_coding	chr15:78264086-7	6591	1.178361197	1.612004613	0.363313	8E-06	2E-05
IDH1	isocitrate dehydrogenase (NADP(+)) 1, cyt	protein_coding	chr2:208236227-2	4721	14.4639316	19.51305089	0.348304	8E-06	2E-05
LINC00960	long intergenic non-protein coding RNA 9	lncRNA	chr3:75672300-75	6543	0.646535362	0.993220146	0.525417	8E-06	2E-05
CSRNP3	cysteine and serine rich nuclear protein 3	protein_coding	chr2:165469647-1	12565	1.234590012	1.697565903	0.378824	8E-06	2E-05
RAC1	Rac family small GTPase 1	protein_coding	chr7:6374527-640	3100	46.09338136	60.34733662	0.303267	8E-06	2E-05
MIR1-1HG	MIR1-1 host gene	lncRNA	chr20:62550453-6	1220	13.52622137	17.55626701	0.291549	8E-06	2E-05
PTGES3L	prostaglandin E synthase 3 like	protein_coding	chr17:42968088-4	2856	7.738238507	10.49715045	0.358168	8E-06	2E-05
HIC2	HIC ZBTB transcriptional repressor 2	protein_coding	chr22:21417371-2	7252	4.764654498	6.838025002	0.442176	8E-06	2E-05
SHF	Src homology 2 domain containing F	protein_coding	chr15:45167214-4	4894	0.872113864	1.368486532	0.562249	8E-06	2E-05
PGAP2	post-GPI attachment to proteins 2	protein_coding	chr11:3797724-38	3781	5.190195349	6.627028692	0.265999	8E-06	2E-05
ZNF334	zinc finger protein 334	protein_coding	chr20:46499630-4	6865	0.729704983	1.098927866	0.502314	8E-06	2E-05
HGSNAT	heparan-alpha-glucosaminide N-acetyltra	protein_coding	chr8:43140464-43	8934	6.027403046	8.040404205	0.334568	8E-06	2E-05
HOXB2	homeobox B2	protein_coding	chr17:48540894-4	2336	1.804337482	2.709155174	0.504245	8E-06	2E-05
SNHG3	small nucleolar RNA host gene 3	lncRNA	chr1:28505980-28	4088	1.742032908	2.615623367	0.502537	8E-06	2E-05
WDR83	WD repeat domain 83	protein_coding	chr19:12666802-1	2358	2.935863684	4.129256248	0.403507	8E-06	2E-05
MSANTD3	Myb/SANT DNA binding domain containir	protein_coding	chr9:100427156-1	3253	5.450726735	7.113877484	0.298538	8E-06	2E-05
EXD3	exonuclease 3'-5' domain containing 3	protein_coding	chr9:137306896-1	7575	2.955399172	3.936367938	0.326404	8E-06	2E-05
TMBIM1	transmembrane BAX inhibitor motif conta	protein_coding	chr2:218274197-2	5093	1.684360078	2.40948182	0.427626	8E-06	2E-05
FAM171B	family with sequence similarity 171 meml	protein_coding	chr2:186694060-1	5731	1.485561945	2.078405026	0.392301	9E-06	2E-05
AL355607.1	novel transcript	lncRNA	chr9:90957260-90	295	0.243957581	1.580279041	2.595392	9E-06	2E-05
ADI1	acireductone dioxygenase 1	protein_coding	chr2:3497366-351	5350	9.884694879	12.61621553	0.267391	9E-06	2E-05
CCDC115	coiled-coil domain containing 115	protein_coding	chr2:130337933-1	4440	2.697091074	3.751022063	0.39491	9E-06	2E-05
YRDC	yrdC N6-threonylcarbamoyltransferase dc	protein_coding	chr1:37802945-37	1848	6.034536264	8.538189431	0.410932	9E-06	2E-05
CAHM	colon adenocarcinoma hypermethylated	lncRNA	chr6:163413065-1	896	2.183147045	3.702333271	0.666644	9E-06	2E-05
OPN3	opsin 3	protein_coding	chr1:241590102-2	3724	2.055421655	2.995567885	0.448849	9E-06	2E-05
WDR27	WD repeat domain 27	protein_coding	chr6:169457212-1	12610	0.783118485	1.076032108	0.370391	9E-06	2E-05
TXLNG	taxilin gamma	protein_coding	chrX:16786432-16	4455	6.249404353	7.976114734	0.265059	9E-06	2E-05
MYDGF	myeloid derived growth factor	protein_coding	chr19:4641374-46	1678	17.29382056	22.14754671	0.268945	9E-06	2E-05
AC124242.1	novel transcript	lncRNA	chr8:18084386-18	3653	0.619368278	1.06041632	0.691777	9E-06	2E-05
CNDP2	carnosine dipeptidase 2	protein_coding	chr18:74495816-7	11822	3.63675391	4.652952998	0.268932	9E-06	2E-05
RP9	RP9 pre-mRNA splicing factor	protein_coding	chr7:33094797-33	1901	5.425734735	8.378107894	0.547463	9E-06	2E-05
PPIG	peptidylprolyl isomerase G	protein_coding	chr2:169584344-1	7364	8.519779231	10.91132645	0.272715	9E-06	2E-05
TIGD2	tigger transposable element derived 2	protein_coding	chr4:89111500-89	3217	2.392574936	3.272865843	0.367843	9E-06	2E-05
ATF1	activating transcription factor 1	protein_coding	chr12:50763710-5	2830	6.383052191	8.556623435	0.340257	9E-06	2E-05
TSNAX	translin associated factor X	protein_coding	chr1:231528541-2	6353	5.623382992	7.928419717	0.413856	9E-06	2E-05
RUFY2	RUN and FYVE domain containing 2	protein_coding	chr10:68341107-6	8137	1.533997092	2.117531929	0.376982	9E-06	2E-05
AEBP2	AE binding protein 2	protein_coding	chr12:19404045-1	6754	3.735455206	4.761263116	0.263403	9E-06	2E-05
SPRYD3	SPRY domain containing 3	protein_coding	chr12:53064316-5	3463	12.48201971	16.32414015	0.301548	9E-06	2E-05
DHODH	dihydroorotate dehydrogenase (quinone)	protein_coding	chr16:72008588-7	6921	1.016749637	1.532581884	0.505436	9E-06	2E-05
ZNF426-DT	ZNF426 divergent transcript	lncRNA	chr19:9538693-95	905	1.13980682	2.357613056	0.970702	9E-06	2E-05
PRKD1	protein kinase D1	protein_coding	chr14:29576479-3	5221	1.11438618	1.608674641	0.449806	9E-06	2E-05
AC084198.2	novel transcript	lncRNA	chr3:101686827-1	1704	2.854115059	4.329690545	0.504518	1E-05	2E-05
ZNF816	zinc finger protein 816	protein_coding	chr19:52949379-5	3310	0.793165845	1.338460821	0.664137	1E-05	2E-05
TPMT	thiopurine S-methyltransferase	protein_coding	chr6:18128311-18	3184	7.639266284	9.84339328	0.276552	1E-05	2E-05
B3GALT4	beta-1,3-galactosyltransferase 4	protein_coding	chr6:33277132-33	2356	2.510929968	3.684141843	0.469583	1E-05	2E-05
QRSL1	glutaminyl-tRNA amidotransferase subuni	protein_coding	chr6:106629578-1	5453	4.912799785	6.71394683	0.366191	1E-05	2E-05
PATJ	PATJ crumbs cell polarity complex compc	protein_coding	chr1:61742406-62	11990	1.967624294	2.517382486	0.268479	1E-05	2E-05
WDR46	WD repeat domain 46	protein_coding	chr6:33279108-33	2835	6.963781231	9.337133603	0.333632	1E-05	2E-05
PIH1D1	PIH1 domain containing 1	protein_coding	chr19:49446298-4	3331	4.132338629	5.616886217	0.357241	1E-05	2E-05
GNG11	G protein subunit gamma 11	protein_coding	chr7:93921699-93	3055	1.140834452	1.753031508	0.533643	1E-05	2E-05
ACACA	acetyl-CoA carboxylase alpha	protein_coding	chr17:37084992-3	13022	5.03058258	6.478696379	0.281738	1E-05	2E-05
LINC00847	long intergenic non-protein coding RNA 8	lncRNA	chr5:180830326-1	6088	0.965739846	1.352967818	0.404045	1E-05	2E-05
ARL16	ADP ribosylation factor like GTPase 16	protein_coding	chr17:81681174-8	2597	8.351316564	10.74892983	0.280042	1E-05	2E-05
SEC22A	SEC22 homolog A, vesicle trafficking prote	protein_coding	chr3:123201927-1	4704	1.322987992	1.879941116	0.416416	1E-05	2E-05
RPS3	ribosomal protein S3	protein_coding	chr11:75399515-7	5859	50.10062614	65.03505052	0.291216	1E-05	2E-05
PUS7L	pseudouridine synthase 7 like	protein_coding	chr12:43718993-4	14037	0.928480488	1.27027541	0.367433	1E-05	2E-05
TYW1	tRNA-yW synthesizing protein 1 homolog	protein_coding	chr7:66995173-67	3885	2.414229385	3.236285782	0.336653	1E-05	2E-05
SLC7A2	solute carrier family 7 member 2	protein_coding	chr8:17497088-17	7905	0.905660586	1.478895858	0.626413	1E-05	2E-05
LAMTOR4	late endosomal/lysosomal adaptor, MAPK	protein_coding	chr7:100148907-1	1936	14.71096682	19.75301355	0.335946	1E-05	2E-05
MYL9	myosin light chain 9	protein_coding	chr20:36541497-3	2808	836.1887822	1085.136197	0.291724	1E-05	2E-05
C11orf49	chromosome 11 open reading frame 49	protein_coding	chr11:46936689-4	6693	3.460858134	4.40713601	0.263719	1E-05	2E-05
LENG8-AS1	LENG8 antisense RNA 1	lncRNA	chr19:54444807-5	1662	1.969050425	3.085216526	0.56336	1E-05	3E-05
APTX	aprataxin	protein_coding	chr9:32972606-33	3789	7.355987715	9.557470168	0.290469	1E-05	3E-05
FIG4	FIG4 phosphoinositide 5-phosphatase	protein_coding	chr6:109691312-1	3592	3.393453969	4.384525755	0.281895	1E-05	3E-05

TRMT10C	tRNA methyltransferase 10C, mitochondr	protein_coding	chr3:101561868-1	1882	14.98256418	20.42343281	0.364343	1E-05	3E-05
GLMP	glycosylated lysosomal membrane protei	protein_coding	chr1:156290089-1	3471	4.43778603	5.787833931	0.30186	1E-05	3E-05
BLVRA	biliverdin reductase A	protein_coding	chr7:43758680-43	1821	12.48378519	16.47151152	0.30851	1E-05	3E-05
BOK	BCL2 family apoptosis regulator BOK	protein_coding	chr2:241551424-2	2680	13.05815072	16.88436033	0.282399	1E-05	3E-05
SNHG5	small nucleolar RNA host gene 5	lncRNA	chr6:85650491-85	8414	4.335887372	5.56562583	0.273611	1E-05	3E-05
RPL18	ribosomal protein L18	protein_coding	chr19:48615328-4	3857	62.28924644	82.07906665	0.314613	1E-05	3E-05
MCAT	malonyl-CoA-acyl carrier protein transacy	protein_coding	chr22:43132206-4	2388	8.732251579	11.17435265	0.270243	1E-05	3E-05
C20orf27	chromosome 20 open reading frame 27	protein_coding	chr20:3753508-37	2415	4.212893639	6.123233727	0.453865	1E-05	3E-05
CLIC1	chloride intracellular channel 1	protein_coding	chr6:31730581-31	1942	25.9620412	34.69736439	0.336464	1E-05	3E-05
BX890604.2	novel transcript	lncRNA	chrX:3817528-393	16206	1.472281494	1.895363474	0.276816	1E-05	3E-05
D2HGDH	D-2-hydroxyglutarate dehydrogenase	protein_coding	chr2:241734602-2	5256	4.477717568	6.117779008	0.364178	1E-05	3E-05
RPS6	ribosomal protein S6	protein_coding	chr9:19375715-19	2714	138.815452	185.1019421	0.331797	1E-05	3E-05
AGTPBP1	ATP/GTP binding protein 1	protein_coding	chr9:85546539-85	4939	7.435331862	9.493597878	0.269289	1E-05	3E-05
RPL37A	ribosomal protein L37a	protein_coding	chr2:216498189-2	6841	40.95774202	54.398975	0.326387	1E-05	3E-05
ZCRB1	zinc finger CCHC-type and RNA binding mo	protein_coding	chr12:42312086-4	2438	12.69541074	18.35318825	0.453418	1E-05	3E-05
FBXO46	F-box protein 46	protein_coding	chr19:45710629-4	3527	6.533078903	8.666572114	0.325187	1E-05	3E-05
TMEM163	transmembrane protein 163	protein_coding	chr2:134455759-1	6804	5.151201037	6.591831727	0.267992	1E-05	3E-05
PIM2	Pim-2 proto-oncogene, serine/threonine	protein_coding	chrX:48913182-48	2761	3.197423439	4.265002808	0.333487	1E-05	3E-05
NPTN-IT1	NPTN intronic transcript 1	lncRNA	chr15:73567012-7	2283	15.06536486	19.50410137	0.288132	1E-05	3E-05
TMEM186	transmembrane protein 186	protein_coding	chr16:8780384-87	1972	4.050321814	5.748416173	0.419093	1E-05	3E-05
CTTNBP2NL	CTTNBP2 N-terminal like	protein_coding	chr1:112396214-1	7371	3.260801076	4.244694696	0.295031	1E-05	3E-05
RPL12	ribosomal protein L12	protein_coding	chr9:127447674-1	2453	77.54284736	103.0397182	0.32717	1E-05	3E-05
PSMD10	proteasome 26S subunit, non-ATPase 10	protein_coding	chrX:108084207-1	2390	14.02763242	18.23537048	0.290375	1E-05	3E-05
HAUS6	HAUS augmin like complex subunit 6	protein_coding	chr9:19053141-19	7719	1.813681426	2.423974103	0.332756	1E-05	3E-05
AC138393.3	novel transcript	lncRNA	chr1:223994262-2	573	0.720143582	1.84274179	1.254445	1E-05	3E-05
RANGRF	RAN guanine nucleotide release factor	protein_coding	chr17:8288654-82	1420	11.58888607	15.55295771	0.342334	1E-05	3E-05
DMD	dystrophin	protein_coding	chrX:31097677-33	18910	17.6806529	23.57441017	0.332475	1E-05	3E-05
PNMT	phenylethanolamine N-methyltransferase	protein_coding	chr17:39667981-3	1457	5.129116112	7.170421963	0.400052	1E-05	3E-05
AC016747.1	novel transcript	lncRNA	chr2:61141592-61	3109	1.080142234	1.639382806	0.510517	1E-05	3E-05
CALM2	calmodulin 2	protein_coding	chr2:47160083-47	7114	27.7495959	35.5093058	0.271853	1E-05	3E-05
AC083906.3	novel transcript	lncRNA	chr3:129954105-1	466	0.167300074	0.861996466	2.149912	1E-05	3E-05
CAAP1	caspase activity and apoptosis inhibitor 1	protein_coding	chr9:26840685-26	3160	5.292647922	7.177642189	0.358835	1E-05	3E-05
NINJ1	ninjurin 1	protein_coding	chr9:93121496-93	2604	4.281071486	5.850019861	0.364619	1E-05	3E-05
AC064807.1	novel transcript	lncRNA	chr8:51899268-51	4813	1.510562408	2.066428039	0.366021	1E-05	3E-05
ZNF512	zinc finger protein 512	protein_coding	chr2:27582969-27	4400	3.771271561	5.04863081	0.337642	1E-05	3E-05
NAIF1	nuclear apoptosis inducing factor 1	protein_coding	chr9:128061233-1	3620	2.306763597	3.166255094	0.373848	1E-05	3E-05
ABCC6	ATP binding cassette subfamily C member	protein_coding	chr16:16148928-1	7569	5.501720177	7.201702067	0.302933	1E-05	3E-05
CHMP4C	charged multivesicular body protein 4C	protein_coding	chr8:81732448-81	1852	4.244977912	5.705503398	0.339977	1E-05	3E-05
THAP5	THAP domain containing 5	protein_coding	chr7:108554543-1	5056	5.982310359	7.677296711	0.272851	1E-05	3E-05
CMTR2	cap methyltransferase 2	protein_coding	chr16:71281389-7	7582	4.033086601	5.345453447	0.319948	1E-05	3E-05
MRPS34	mitochondrial ribosomal protein S34	protein_coding	chr16:1771890-17	1097	55.39034002	72.16612031	0.297125	1E-05	3E-05
POMT1	protein O-mannosyltransferase 1	protein_coding	chr9:131502902-1	6067	5.594831969	7.130416828	0.26506	1E-05	3E-05
GPATCH3	G-patch domain containing 3	protein_coding	chr1:26890488-26	2226	4.695969319	6.420572493	0.364596	1E-05	3E-05
CYP2R1	cytochrome P450 family 2 subfamily R me	protein_coding	chr11:14877440-1	4516	2.072965409	2.776151461	0.334259	1E-05	3E-05
MYCBP2	MYC binding protein 2	protein_coding	chr13:77044657-7	15987	3.474414002	4.744651101	0.370247	1E-05	3E-05
TTLL1	tubulin tyrosine ligase like 1	protein_coding	chr22:43039516-4	1868	8.038694065	10.40412126	0.287943	1E-05	3E-05
CENPH	centromere protein H	protein_coding	chr5:69189574-69	2218	2.731748552	3.947572079	0.448281	1E-05	3E-05
ZFPM1	zinc finger protein, FOG family member 1	protein_coding	chr16:88453280-8	6162	6.031097656	7.709442747	0.266817	1E-05	3E-05
B9D1	B9 domain containing 1	protein_coding	chr17:19334308-1	8483	1.92154316	2.507488762	0.29384	1E-05	3E-05
B3GALT6	beta-1,3-galactosyltransferase 6	protein_coding	chr1:1232237-123	2805	16.82594027	21.96829681	0.29701	1E-05	3E-05
TMCO3	transmembrane and coiled-coil domains	protein_coding	chr13:113491021-	10381	3.954093349	5.15904965	0.298428	1E-05	3E-05
MEGF9	multiple EGF like domains 9	protein_coding	chr9:120600811-1	6300	1.615355703	2.223359527	0.372405	1E-05	3E-05
TST	thiosulfate sulfurtransferase	protein_coding	chr22:37010859-3	1877	8.927059753	12.14058899	0.351992	1E-05	3E-05
TOR2A	torsin family 2 member A	protein_coding	chr9:127731524-1	3790	1.526171221	2.191047237	0.439716	1E-05	3E-05
USP49	ubiquitin specific peptidase 49	protein_coding	chr6:41789896-41	10921	0.930964757	1.327813345	0.428391	1E-05	3E-05
CLPP	caseinolytic mitochondrial matrix peptid	protein_coding	chr19:6361531-63	2195	19.42076869	25.00343583	0.280187	1E-05	3E-05
RNF111	ring finger protein 111	protein_coding	chr15:58865175-5	7181	4.801436644	6.166780741	0.274614	1E-05	3E-05
ERVK13-1	endogenous retrovirus group K13 membe	lncRNA	chr16:2660348-26	11465	5.365805903	6.873595288	0.273645	1E-05	3E-05
MAP3K2-DT	MAP3K2 divergent transcript	lncRNA	chr2:127388184-1	3254	0.689131455	1.107588007	0.599391	1E-05	3E-05
LSM7	LSM7 homolog, U6 small nuclear RNA and	protein_coding	chr19:2321520-23	1686	12.04855674	16.21107844	0.341164	1E-05	3E-05
ARMC2	armadillo repeat containing 2	protein_coding	chr6:108848416-1	4254	0.973951578	1.486819286	0.528631	1E-05	3E-05
MAD2L2	mitotic arrest deficient 2 like 2	protein_coding	chr1:11674480-11	3324	5.01914897	6.632303381	0.316988	1E-05	3E-05
WARS2	tryptophanyl tRNA synthetase 2, mitocho	protein_coding	chr1:119031216-1	3847	3.739169654	4.954825569	0.321865	2E-05	3E-05
SDK2	sidekick cell adhesion molecule 2	protein_coding	chr17:73334384-7	11963	6.809857522	9.552282429	0.408623	2E-05	3E-05
SORBS1	sorbin and SH3 domain containing 1	protein_coding	chr10:95311771-9	10951	5.03859415	6.529422304	0.29084	2E-05	3E-05
TAPT1	transmembrane anterior posterior transfo	protein_coding	chr4:16160505-16	7758	1.849760843	2.405979469	0.296206	2E-05	3E-05
AL442125.1	novel transcript, sense intronic to TMCO3	lncRNA	chr13:113527260-	3362	0.686502399	1.142191558	0.651857	2E-05	3E-05
SLC16A10	solute carrier family 16 member 10	protein_coding	chr6:111087503-1	11237	1.135574216	1.50786563	0.324716	2E-05	3E-05
ZNF839	zinc finger protein 839	protein_coding	chr14:102317377-	5590	2.61734523	3.561201383	0.359225	2E-05	3E-05
Z92544.2	novel transcript, antisense to FBXL16	lncRNA	chr16:689001-692	3554	0.765478484	1.232004993	0.592557	2E-05	3E-05
FKBP14	FKBP prolyl isomerase 14	protein_coding	chr7:30010587-30	5326	3.011462231	4.002400936	0.324501	2E-05	3E-05
FAM91A1	family with sequence similarity 91 memb	protein_coding	chr8:123768439-1	7263	2.830938679	3.61794983	0.267198	2E-05	3E-05
QSOX2	quiescin sulfhydryl oxidase 2	protein_coding	chr9:136206333-1	4530	3.846281746	5.105965933	0.324955	2E-05	4E-05
CMTM7	CKLF like MARVEL transmembrane domai	protein_coding	chr3:32391671-32	3751	0.974426641	1.524056202	0.559022	2E-05	4E-05
RPL7A	ribosomal protein L7a	protein_coding	chr9:133348218-1	2238	204.7408883	271.3784904	0.323578	2E-05	4E-05
RNF7	ring finger protein 7	protein_coding	chr3:141738249-1	3102	7.826120961	10.40483132	0.32117	2E-05	4E-05
LINC00174	long intergenic non-protein coding RNA 1	lncRNA	chr7:66376044-66	11632	1.103780753	1.546236698	0.409476	2E-05	4E-05
RAD54L2	RAD54 like 2	protein_coding	chr3:51541144-51	11924	3.475967209	4.732697761	0.364894	2E-05	4E-05
COIL	coilin	protein_coding	chr17:56938199-5	3014	6.449300495	8.236411813	0.266473	2E-05	4E-05
NIPSNAP3A	nipsnap homolog 3A	protein_coding	chr9:104747683-1	2588	9.643512768	12.99088522	0.341132	2E-05	4E-05
LRRC4	leucine rich repeat containing 4	protein_coding	chr7:128027071-1	4403	1.364476802	1.928081629	0.406741	2E-05	4E-05
A1BG-AS1	A1BG antisense RNA 1	lncRNA	chr19:58347718-5	3374	2.837973087	3.859974029	0.359979	2E-05	4E-05
DCHS1	dachsous cadherin-related 1	protein_coding	chr11:6621330-66	10713	9.117320398	11.76212821	0.284505	2E-05	4E-05
UTP23	UTP23 small subunit processome compor	protein_coding	chr8:116766505-1	5244	2.338039111	3.033674872	0.286332	2E-05	4E-05
BBS7	Bardet-Biedl syndrome 7	protein_coding	chr4:121824440-1	4829	2.339559716	3.139202178	0.340318	2E-05	4E-05
BLOC1S2	biogenesis of lysosomal organelles compl	protein_coding	chr10:100273280-	2911	5.704835792	7.561683331	0.321569	2E-05	4E-05
PIFO	primary cilia formation	protein_coding	chr1:111346600-1	3949	2.325455705	3.136726223	0.342654	2E-05	4E-05
CCDC93	coiled-coil domain containing 93	protein_coding	chr2:117915478-1	8065	6.081568797	7.875165917	0.291213	2E-05	4E-05
CDAN1	codanin 1	protein_coding	chr15:42723544-4	5448	4.344019164	5.797593437	0.332109	2E-05	4E-05
MZF1	myeloid zinc finger 1	protein_coding	chr19:58561931-5	6691	4.27697521	5.50194396	0.278164	2E-05	4E-05
WDR3	WD repeat domain 3	protein_coding	chr1:117929720-1	10276	2.160500792	2.750451721	0.264127	2E-05	4E-05
APOO	apolipoprotein O	protein_coding	chrX:23833353-23	2353	16.20194474	21.55928191	0.330684	2E-05	4E-05
ERMARD	ER membrane associated RNA degradatior	protein_coding	chr6:169751622-1	4267	1.955490291	2.672074463	0.364488	2E-05	4E-05
YPEL1	yippee like 1	protein_coding	chr22:21697536-2	4681	5.527486913	7.257577845	0.304963	2E-05	4E-05
WASF1	WASP family member 1	protein_coding	chr6:110099819-1	3260	3.751168318	4.881066931	0.2942	2E-05	4E-05
UAP1	UDP-N-acetylglucosamine pyrophosphory	protein_coding	chr1:162561506-1	3437	3.685427743	4.951375277	0.344262	2E-05	4E-05
TNIP2	TNFAIP3 interacting protein 2	protein_coding	chr4:2741648-275	2686	8.438437167	10.86132346	0.276085	2E-05	4E-05
DYNC2LI1	dynein cytoplasmic 2 light intermediate c	protein_coding	chr2:43774039-43	4618	2.662583514	3.80235435	0.421069	2E-05	4E-05
SLC25A21	solute carrier family 25 member 21	protein_coding	chr14:36678431-3	3703	0.803379739	1.302222762	0.594573	2E-05	4E-05
SGMS2	sphingomyelin synthase 2	protein_coding	chr4:107824563-1	7635	1.093294046	1.473551071	0.342745	2E-05	4E-05
DCUN1D3	defective in cullin neddylation 1 domain c	protein_coding	chr16:20854925-2	6222	2.987342802	3.822677826	0.26612	2E-05	4E-05
PINX1	PIN2 (TERF1) interacting telomerase inhib	protein_coding	chr8:10725399-10	1753	4.120969853	5.722304679	0.385142	2E-05	5E-05
MIB1	mindbomb E3 ubiquitin protein ligase 1	protein_coding	chr18:21704957-2	10033	9.0630631	11.61746324	0.273316	2E-05	5E-05
WHRN	whirlin	protein_coding	chr9:114402080-1	5565	0.906914558	1.293000909	0.425529	2E-05	5E-05
TCF20	transcription factor 20	protein_coding	chr22:42160013-4	7548	3.627541098	4.709245605	0.292975	2E-05	5E-05
AL590064.1	novel transcript	lncRNA	chr13:40874685-4	933	0.328163529	1.013205709	1.557004	2E-05	5E-05
KAT6A	lysine acetyltransferase 6A	protein_coding	chr8:41929479-42	24143	2.649544669	3.475905902	0.30732	2E-05	5E-05
ZNF711	zinc finger protein 711	protein_coding	chrX:85244032-85	5004	5.083651899	6.503112604	0.268249	2E-05	5E-05
FBXO6	F-box protein 6	protein_coding	chr1:11664200-11	1735	1.526933841	2.369088459	0.540571	2E-05	5E-05
RBM7	RNA binding motif protein 7	protein_coding	chr11:114400030-	7885	2.413220755	3.111050788	0.279164	2E-05	5E-05
SLC35B1	solute carrier family 35 member B1	protein_coding	chr17:49700934-4	5928	7.428900256	9.49343622	0.268171	2E-05	5E-05

TAF4	TATA-box binding protein associated facto	protein_coding	chr20:61953469-6	9808	1.912526167	2.446830788	0.272809	2E-05	5E-05
CEBPA	CCAAT enhancer binding protein alpha	protein_coding	chr19:33299934-3	2601	0.732884112	1.399000296	0.844479	2E-05	5E-05
AL121601.1	novel transcript	lncRNA	chrX:123959233-1	326	1.282396858	3.294357874	1.237937	2E-05	5E-05
SLC9A7	solute carrier family 9 member A7	protein_coding	chrX:46599251-46	9980	0.89294175	1.207715938	0.347776	2E-05	5E-05
SLC12A5	solute carrier family 12 member 5	protein_coding	chr20:46021690-4	8909	0.916429559	1.281421108	0.395281	2E-05	5E-05
TBL2	transducin beta like 2	protein_coding	chr7:73567533-73	6466	2.944070916	3.878384772	0.309669	2E-05	5E-05
AC100810.1	novel transcript	lncRNA	chr8:1761054-176	2264	6.361011298	8.652093355	0.362672	2E-05	5E-05
APOPT1	apoptogenic 1, mitochondrial	protein_coding	chr14:103562962-	3932	2.334773101	3.106963626	0.322681	2E-05	5E-05
EIF2A	eukaryotic translation initiation factor 2A	protein_coding	chr3:150546678-1	5905	10.73212309	13.83415309	0.28093	2E-05	5E-05
SUGCT	succinyl-CoA:glutarate-CoA transferase	protein_coding	chr7:40134977-40	2857	3.226998523	4.502101532	0.390643	2E-05	5E-05
CRYZ	crystallin zeta	protein_coding	chr1:74705482-74	4085	4.000408482	5.308635442	0.322341	2E-05	5E-05
TMEM158	transmembrane protein 158 (gene/pseud	protein_coding	chr3:45224466-45	1813	0.338842776	0.841869545	1.250423	2E-05	5E-05
EBPL	EBP like	protein_coding	chr13:49660674-4	1560	6.863191905	9.727276021	0.423454	2E-05	5E-05
PDP2	pyruvate dehyrogenase phosphatase catal	protein_coding	chr16:66878589-6	8364	2.236913955	2.893893657	0.291478	2E-05	5E-05
CFAP70	cilia and flagella associated protein 70	protein_coding	chr10:73253759-7	7432	0.59160475	0.960233928	0.620136	2E-05	5E-05
FITM1	fat storage inducing transmembrane prot	protein_coding	chr14:24131275-2	1355	94.0704916	126.4241332	0.345698	2E-05	5E-05
NFKBIE	NFKB inhibitor epsilon	protein_coding	chr6:44258166-44	2581	2.006702347	2.786943917	0.384963	2E-05	5E-05
SMIM29	small integral membrane protein 29	protein_coding	chr6:34246381-34	1866	3.126145643	4.898100951	0.574593	3E-05	5E-05
TIRAP	TIR domain containing adaptor protein	protein_coding	chr11:126283065-	3591	2.199342515	3.098310033	0.405327	3E-05	5E-05
FZD9	frizzled class receptor 9	protein_coding	chr7:73433783-73	2338	0.743205139	1.412426138	0.82178	3E-05	5E-05
TTC33	tetratricopeptide repeat domain 33	protein_coding	chr5:40512333-40	9708	2.334326564	3.05009666	0.295329	3E-05	6E-05
MTM1	myotubularin 1	protein_coding	chrX:150568619-1	4016	1.691058636	2.395147028	0.414123	3E-05	6E-05
CDH8	cadherin 8	protein_coding	chr16:61647242-6	13529	0.741582566	1.061597313	0.436399	3E-05	6E-05
CTU2	cytosolic thiouridylase subunit 2	protein_coding	chr16:88706483-8	3522	3.472764273	4.666937437	0.336086	3E-05	6E-05
SMG1	SMG1 nonsense mediated mRNA decay as	protein_coding	chr16:18804853-1	25988	5.530601761	7.398258928	0.339021	3E-05	6E-05
SNHG9	small nucleolar RNA host gene 9	lncRNA	chr16:1964959-19	551	6.316333871	9.923877385	0.562024	3E-05	6E-05
AC104596.1	novel transcript	lncRNA	chr4:142933195-1	1686	0.602955971	1.168063937	0.889529	3E-05	6E-05
MFSD4B	major facilitator superfamily domain con	protein_coding	chr6:111259348-1	5958	2.134269166	2.959065001	0.381282	3E-05	6E-05
LRRC24	leucine rich repeat containing 24	protein_coding	chr8:144522388-1	1762	4.148715997	6.086495007	0.463228	3E-05	6E-05
ZFYVE26	zinc finger FYVE-type containing 26	protein_coding	chr14:67727374-6	15704	2.994311895	3.882829321	0.293512	3E-05	6E-05
CARD19	caspase recruitment domain family memb	protein_coding	chr9:93096217-93	3082	6.240714579	7.971722565	0.269863	3E-05	6E-05
SNRPD2	small nuclear ribonucleoprotein D2 polyp	protein_coding	chr19:45687454-4	1608	35.72049206	46.95411871	0.311308	3E-05	6E-05
RNF180	ring finger protein 180	protein_coding	chr5:64165843-64	5435	1.097637215	1.529785655	0.388717	3E-05	6E-05
GPR135	G protein-coupled receptor 135	protein_coding	chr14:59429022-5	4977	2.521730299	3.620148448	0.438917	3E-05	6E-05
OTUD1	OTU deubiquitinase 1	protein_coding	chr10:23439458-2	2933	8.572288856	12.31549863	0.429331	3E-05	6E-05
CYP20A1	cytochrome P450 family 20 subfamily A n	protein_coding	chr2:203238940-2	3079	2.30105783	3.110011904	0.347254	3E-05	6E-05
TBC1D16	TBC1 domain family member 16	protein_coding	chr17:79932343-8	11579	3.786965408	5.211252149	0.381656	3E-05	6E-05
PAGR1	PAXIP1 associated glutamate rich protein	protein_coding	chr16:29816152-2	3874	2.14568985	3.24033923	0.520102	3E-05	7E-05
KLHDC9	kelch domain containing 9	protein_coding	chr1:161098361-1	1720	4.118759227	6.012705982	0.465295	3E-05	7E-05
ZFC3H1	zinc finger C3H1-type containing	protein_coding	chr12:71609472-7	9670	5.618669473	7.221163408	0.278666	3E-05	7E-05
TMEM18	transmembrane protein 18	protein_coding	chr2:663877-6774	8469	2.239285792	2.896693734	0.282838	3E-05	7E-05
AL662844.4	novel transcript, antisense to HCG27	lncRNA	chr6:31195200-31	2838	1.062948449	1.653212342	0.553274	3E-05	7E-05
AC005229.4	novel transcript, antisense to CUL1	lncRNA	chr7:148696467-1	2198	2.092776754	3.024078752	0.447681	3E-05	7E-05
AC073111.4	novel zinc finger protein	protein_coding	chr7:150400702-1	4333	0.69402595	1.122049438	0.619549	3E-05	7E-05
ADPRHL2	ADP-ribosylhydrolase like 2	protein_coding	chr1:36088892-36	1651	10.24276398	13.1045211	0.271344	3E-05	7E-05
OST4	oligosaccharyltransferase complex subun	protein_coding	chr2:27070472-27	600	126.3071208	172.2704688	0.367763	3E-05	7E-05
BICRAL	BRD4 interacting chromatin remodeling c	protein_coding	chr6:42746958-42	6843	3.089271783	4.120684115	0.330503	3E-05	7E-05
MANEAL	mannosidase endo-alpha like	protein_coding	chr1:37793802-37	3018	0.984432007	1.507806954	0.543538	3E-05	7E-05
Z93930.2	novel transcript	lncRNA	chr22:28800683-2	1470	0.452195439	0.95524244	1.004173	3E-05	7E-05
TGDS	TDP-glucose 4,6-dehydratase	protein_coding	chr13:94574054-9	2571	2.584211983	3.536123902	0.369566	3E-05	7E-05
C2orf76	chromosome 2 open reading frame 76	protein_coding	chr2:119302225-1	1554	2.310284364	3.488240679	0.512077	3E-05	7E-05
TADA1	transcriptional adaptor 1	protein_coding	chr1:166856510-1	2900	4.535330763	5.808805883	0.268804	3E-05	7E-05
NAPA-AS1	NAPA antisense RNA 1	lncRNA	chr19:47484282-4	1894	1.18808879	2.071565882	0.714092	3E-05	7E-05
TMEM70	transmembrane protein 70	protein_coding	chr8:73972437-73	2982	11.51285234	14.72407515	0.269144	3E-05	7E-05
SIRT7	sirtuin 7	protein_coding	chr17:81911939-8	6134	2.17577681	2.887473339	0.323715	3E-05	7E-05
TM2D3	TM2 domain containing 3	protein_coding	chr15:101621444-	11894	2.045949335	2.604797647	0.26384	3E-05	7E-05
USE1	unconventional SNARE in the ER 1	protein_coding	chr19:17215346-1	2431	6.361869146	8.444037473	0.326634	3E-05	7E-05
PHLDA3	pleckstrin homology like domain family A	protein_coding	chr1:201464383-2	3138	37.35956462	47.88320005	0.271018	4E-05	7E-05
C1QL1	complement C1q like 1	protein_coding	chr17:44959693-4	1527	1.816939145	2.984215247	0.62839	4E-05	7E-05
DCAF13	DDB1 and CUL4 associated factor 13	protein_coding	chr8:103414714-1	9199	3.468385216	4.42423861	0.263937	4E-05	7E-05
APOBEC3F	apolipoprotein B mRNA editing enzyme ca	protein_coding	chr22:39040604-3	5215	1.507972856	2.011951125	0.331851	4E-05	7E-05
SLC2A1-AS1	SLC2A1 antisense RNA 1	lncRNA	chr1:42959049-42	4275	0.640455229	0.993049188	0.541348	4E-05	8E-05
CFAP36	cilia and flagella associated protein 36	protein_coding	chr2:55519604-55	3505	3.166814653	4.204997713	0.320494	4E-05	8E-05
AC008267.5	novel transcript	lncRNA	chr7:66493607-66	1262	2.138483965	3.373849991	0.566574	4E-05	8E-05
TMOD3	tropomodulin 3	protein_coding	chr15:51829628-5	10632	0.753144806	0.990038796	0.307686	4E-05	8E-05
ZNF226	zinc finger protein 226	protein_coding	chr19:44165073-4	6922	1.925442843	2.521501664	0.303688	4E-05	8E-05
PRR14L	proline rich 14 like	protein_coding	chr22:31676256-3	11809	5.034290526	6.427686451	0.268552	4E-05	8E-05
THAP1	THAP domain containing 1	protein_coding	chr8:42836674-42	2462	3.616923454	4.946190088	0.364965	4E-05	8E-05
MYO1E	myosin IE	protein_coding	chr15:59132434-5	9106	4.469990539	5.93093204	0.326799	4E-05	8E-05
PLEKHA8	pleckstrin homology domain containing A	protein_coding	chr7:30027404-30	10886	1.826024322	2.394020802	0.303045	4E-05	8E-05
ZNF510	zinc finger protein 510	protein_coding	chr9:96755865-96	5921	2.288450514	2.945847934	0.277729	4E-05	8E-05
AC027644.3	novel transcript, antisense to RABGEF1	lncRNA	chr7:66739829-66	557	12.57414178	18.05652552	0.431295	4E-05	8E-05
LRFN3	leucine rich repeat and fibronectin type II	protein_coding	chr19:35935358-3	4044	3.057455626	3.941925823	0.284533	4E-05	8E-05
WIPF1	WAS/WASL interacting protein family me	protein_coding	chr2:174559572-1	9121	1.10654453	1.486294064	0.340548	4E-05	8E-05
MAB21L2	mab-21 like 2	protein_coding	chr4:150581922-1	2772	0.903518592	1.387445559	0.523787	4E-05	8E-05
ZNF300	zinc finger protein 300	protein_coding	chr5:150894392-1	4411	0.902105228	1.329250837	0.47393	4E-05	8E-05
MRM3	mitochondrial rRNA methyltransferase 3	protein_coding	chr17:782353-792	1985	7.284587033	9.762226669	0.332586	4E-05	8E-05
PCDHGB6	protocadherin gamma subfamily B, 6	protein_coding	chr5:141408021-1	6782	2.712061243	3.730274542	0.380929	4E-05	8E-05
VPS4A	vacuolar protein sorting 4 homolog A	protein_coding	chr16:69311350-6	5676	3.775088832	4.899325624	0.290082	4E-05	8E-05
LRRC37B	leucine rich repeat containing 37B	protein_coding	chr17:32007872-3	6281	0.727303906	1.056764869	0.454328	4E-05	8E-05
LZTFL1	leucine zipper transcription factor like 1	protein_coding	chr3:45823316-45	7180	0.86246465	1.191525693	0.379235	4E-05	9E-05
TFB1M	transcription factor B1, mitochondrial	protein_coding	chr6:155256134-1	4111	3.223453838	4.163866988	0.285075	4E-05	9E-05
DDAH2	dimethylarginine dimethylaminohydrola	protein_coding	chr6:31727038-31	2363	9.881626532	12.5904717	0.264639	4E-05	9E-05
ZSCAN20	zinc finger and SCAN domain containing 2	protein_coding	chr1:33472645-33	5308	1.138960251	1.572347015	0.372619	4E-05	9E-05
CD58	CD58 molecule	protein_coding	chr1:116514534-1	1784	4.656599571	6.408578173	0.371434	4E-05	9E-05
CCDC24	coiled-coil domain containing 24	protein_coding	chr1:43991359-43	3563	0.851125743	1.388289967	0.621509	4E-05	9E-05
ERRFI1	ERBB receptor feedback inhibitor 1	protein_coding	chr1:8004404-802	4179	2.204075617	2.882348429	0.3054	4E-05	9E-05
AP003390.1	novel transcript	lncRNA	chr11:119729583-	523	0.383490453	1.321108411	1.645184	4E-05	9E-05
AL451127.1	novel transcript	lncRNA	chr9:73573615-73	624	0.531757327	1.246859395	1.135632	4E-05	9E-05
SDCBP2-AS1	SDCBP2 antisense RNA 1	lncRNA	chr20:1325405-13	5308	1.01926114	1.475398454	0.443952	4E-05	9E-05
MINPP1	multiple inositol-polyphosphate phospha	protein_coding	chr10:87504875-8	3017	5.816918903	7.608196895	0.300047	4E-05	9E-05
POLI	DNA polymerase iota	protein_coding	chr18:54269404-5	9471	2.190758892	2.840554939	0.287648	4E-05	9E-05
FAM172A	family with sequence similarity 172 mem	protein_coding	chr5:93617725-94	5415	1.97586991	2.722115338	0.375334	5E-05	9E-05
ZNF436-AS1	ZNF436 antisense RNA 1	lncRNA	chr1:23368997-23	2674	1.305849072	1.914191663	0.460615	5E-05	9E-05
ZNF174	zinc finger protein 174	protein_coding	chr16:3401215-34	2890	3.501990871	4.777568016	0.364424	5E-05	9E-05
SETMAR	SET domain and mariner transposase fusic	protein_coding	chr3:4303304-431	3567	4.683875999	6.070114595	0.286141	5E-05	9E-05
TSPAN11	tetraspanin 11	protein_coding	chr12:30926428-3	7116	2.890426221	3.708564348	0.272947	5E-05	9E-05
AC068888.1	novel transcript, antisense to TENC1 & EIF	lncRNA	chr12:53012884-5	7742	0.6960482	1.035219829	0.485529	5E-05	9E-05
ZDHHC1	zinc finger DHHC-type containing 1	protein_coding	chr16:67394152-6	3540	1.770308273	2.442739244	0.373179	5E-05	1E-04
C6orf226	chromosome 6 open reading frame 226	protein_coding	chr6:42890265-42	557	4.457814844	6.997974407	0.555829	5E-05	1E-04
TBX5-AS1	TBX5 antisense RNA 1	lncRNA	chr12:114408173-	4201	4.261214858	5.739029293	0.3532	5E-05	1E-04
TMEM121	transmembrane protein 121	protein_coding	chr14:105526583-	1924	0.932728774	1.660316021	0.743525	5E-05	1E-04
TMEM62	transmembrane protein 62	protein_coding	chr15:43123279-4	4746	1.90116267	2.501008721	0.30703	5E-05	1E-04
KYAT3	kynurenine aminotransferase 3	protein_coding	chr1:88935773-88	2196	2.847211114	4.093543836	0.442246	5E-05	1E-04
DTD1	D-aminoacyl-tRNA deacylase 1	protein_coding	chr20:18587893-1	6465	0.661775877	1.010200344	0.536383	5E-05	1E-04
NENF	neudesin neurotrophic factor	protein_coding	chr1:212432920-2	1264	10.33009911	15.1450649	0.453364	5E-05	1E-04
AC008554.1	zinc finger protein 737	protein_coding	chr19:20535825-2	8978	0.787490197	1.106158323	0.412666	5E-05	1E-04
EXOC6	exocyst complex component 6	protein_coding	chr10:92834713-9	6193	2.708779812	3.484158714	0.272337	5E-05	0.0001
TSR3	TSR3 ribosome maturation factor	protein_coding	chr16:1349240-13	1210	44.40397292	59.11915111	0.321204	5E-05	0.0001
ZMYM1	zinc finger MYM-type containing 1	protein_coding	chr1:35032172-35	5205	1.972054014	2.620246158	0.328011	5E-05	0.0001
RGS2	regulator of G protein signaling 2	protein_coding	chr1:192809039-1	2088	1.156457601	1.804718047	0.553644	5E-05	0.0001

MTERF1	mitochondrial transcription termination	protein_coding	chr7:91692008-91	4890	1.490257826	1.953018603	0.301391	5E-05	0.0001
DHRS4L2	dehydrogenase/reductase 4 like 2	protein_coding	chr14:23969874-2	2516	2.780931537	3.918656392	0.415791	5E-05	0.0001
MAMSTR	MEF2 activating motif and SAP domain co	protein_coding	chr19:48712725-4	2286	0.832994123	1.333275989	0.589747	5E-05	0.0001
AL590814.1	novel transcript	lncRNA	chr6:92002610-92	2720	0.837909355	1.421859398	0.675093	5E-05	0.0001
AL080317.1	novel transcript	lncRNA	chr6:111273329-1 3030		1.914078063	2.828477332	0.47671	5E-05	0.0001
BLOC1S3	biogenesis of lysosomal organelles comple	protein_coding	chr19:45178745-4	3416	1.731990443	2.378858012	0.369449	5E-05	0.0001
DNASE2	deoxyribonuclease 2, lysosomal	protein_coding	chr19:12875209-1	2389	3.771392443	5.025528285	0.327233	6E-05	0.0001
ACD	ACD shelterin complex subunit and telom	protein_coding	chr16:67657512-6	3223	2.32696222	3.469404314	0.500512	6E-05	0.0001
AMN1	antagonist of mitotic exit network 1 hom	protein_coding	chr12:31671142-3	3137	1.586970025	2.197747229	0.388047	6E-05	0.0001
SFXN2	sideroflexin 2	protein_coding	chr10:102714538-	8034	1.8781657	2.44620223	0.301203	6E-05	0.0001
N4BP2	NEDD4 binding protein 2	protein_coding	chr4:40056850-40	9811	1.649709279	2.164858942	0.310292	6E-05	0.0001
ZNF32	zinc finger protein 32	protein_coding	chr10:43643860-4	1460	13.26062857	17.06125696	0.27808	6E-05	0.0001
CTU1	cytosolic thiouridylase subunit 1	protein_coding	chr19:51097606-5	2126	2.281071327	3.760326063	0.615494	6E-05	0.0001
MHENCR	melanoma highly expressed competing er	lncRNA	chr20:63627227-6	801	3.786594887	5.901902346	0.545104	6E-05	0.0001
RPL13A	ribosomal protein L13a	protein_coding	chr19:49487554-4	2369	202.0010697	270.7197943	0.342889	6E-05	0.0001
AC132872.1	novel transcript	lncRNA	chr17:82293716-8	1195	0.748437107	1.372495868	0.776812	6E-05	0.0001
DPM3	dolichyl-phosphate mannosyltransferase	protein_coding	chr1:155139891-1	622	19.43664037	27.42737972	0.419449	6E-05	0.0001
VPS37D	VPS37D subunit of ESCRT-I	protein_coding	chr7:73667831-73	1620	3.482058362	4.867335908	0.390011	6E-05	0.0001
AL353748.3	novel transcript, antisense to SPIN1	lncRNA	chr9:88384902-88	3329	1.090851263	1.7059428	0.564149	7E-05	0.0001
PKIB	cAMP-dependent protein kinase inhibitor	protein_coding	chr6:122471917-1	3432	1.091154522	1.600679991	0.458718	7E-05	0.0001
C1GALT1C1	C1GALT1 specific chaperone 1	protein_coding	chrX:120625674-1	1808	7.186767533	9.393868785	0.296111	7E-05	0.0001
ACVR1	activin A receptor type 1	protein_coding	chr2:157736444-1	3990	5.789208801	7.394055026	0.263392	7E-05	0.0001
CCDC149	coiled-coil domain containing 149	protein_coding	chr4:24806117-24	9582	1.22492932	1.652750481	0.346462	7E-05	0.0001
AC007405.3	glutamate rich 2	lncRNA	chr2:170766878-1	2905	1.40576436	2.238455965	0.5894	7E-05	0.0001
SPTLC1	serine palmitoyltransferase long chain bas	protein_coding	chr9:92000087-92	6513	4.280311142	5.460173603	0.263418	7E-05	0.0001
MYO15B	myosin XVB	protein_coding	chr17:75588058-7	13109	0.808267036	1.190131602	0.486727	7E-05	0.0001
STX8	syntaxin 8	protein_coding	chr17:9250471-95	3455	4.072897959	5.298482388	0.294859	7E-05	0.0001
RAB32	RAB32, member RAS oncogene family	protein_coding	chr6:146543833-1	1065	5.506382795	7.773068922	0.403643	7E-05	0.0001
GTF2H2	general transcription factor IIH subunit 2	protein_coding	chr5:71032670-71	8676	0.779132446	1.062938087	0.36225	7E-05	0.0001
RNFT1	ring finger protein, transmembrane 1	protein_coding	chr17:59952240-5	3399	1.832936644	2.525008176	0.378991	7E-05	0.0001
DGKA	diacylglycerol kinase alpha	protein_coding	chr12:55927319-5	10098	0.756037047	1.000891109	0.322089	7E-05	0.0001
ANKRD49	ankyrin repeat domain 49	protein_coding	chr11:94493979-9	3171	1.503388656	2.126740857	0.406809	7E-05	0.0001
MYL6B	myosin light chain 6B	protein_coding	chr12:56152256-5	3259	11.36864676	14.47873427	0.263939	7E-05	0.0001
THORLNC	testis associated oncogenic lncRNA	lncRNA	chr2:118132128-1	2765	1.559868992	2.27884826	0.454064	7E-05	0.0001
STS	steroid sulfatase	protein_coding	chrX:7147237-780	12052	1.320765958	1.698405083	0.277895	8E-05	0.0001
AC114811.2	novel transcript, antisense TSPAN5	lncRNA	chr4:98658904-98	5647	0.666481855	1.042158992	0.555615	8E-05	0.0002
BTD	biotinidase	protein_coding	chr3:15601341-15	5840	0.806547056	1.173317792	0.453206	8E-05	0.0002
AATK	apoptosis associated tyrosine kinase	protein_coding	chr17:81117295-8	6369	1.229474697	1.683989576	0.366771	8E-05	0.0002
RPS14	ribosomal protein S14	protein_coding	chr5:150442635-1	5082	68.74653744	87.88214366	0.269898	8E-05	0.0002
ZNF550	zinc finger protein 550	protein_coding	chr19:57535257-5	5087	2.816083855	3.66343922	0.292607	8E-05	0.0002
GPRC5B	G protein-coupled receptor class C group	protein_coding	chr16:19856691-1	6654	5.773096926	7.356492645	0.269848	8E-05	0.0002
PFDN4	prefoldin subunit 4	protein_coding	chr20:54208087-5	1743	7.165409509	10.19004306	0.429787	8E-05	0.0002
ACCS	1-aminocyclopropane-1-carboxylate synt	protein_coding	chr11:44065925-4	5826	0.855137579	1.272994014	0.489217	8E-05	0.0002
PXN-AS1	PXN antisense RNA 1	lncRNA	chr12:120201291-	4629	0.613669222	0.946636583	0.546902	8E-05	0.0002
DLG5	discs large MAGUK scaffold protein 5	protein_coding	chr10:77790791-7 10500		6.920787312	9.120947537	0.315679	8E-05	0.0002
AC018809.1	novel transcript, sense intronic to CRELD1	lncRNA	chr3:9935706-993	553	0.556759304	1.312413808	1.134802	8E-05	0.0002
TIGD6	tigger transposable element derived 6	protein_coding	chr5:149993118-1	4127	1.176828854	1.647609035	0.404258	8E-05	0.0002
UTP25	UTP25 small subunit processor componer	protein_coding	chr1:209827972-2	10411	0.801281994	1.053253173	0.312307	8E-05	0.0002
PHLPP1	PH domain and leucine rich repeat protei	protein_coding	chr18:62715541-6	9051	1.118505949	1.459816752	0.297609	9E-05	0.0002
PFKFB4	6-phosphofructo-2-kinase/fructose-2,6-bi	protein_coding	chr3:48517684-48	6107	1.011144231	1.759051416	0.70482	9E-05	0.0002
ZNF582	zinc finger protein 582	protein_coding	chr19:56375846-5	3350	0.985432375	1.498892753	0.523182	9E-05	0.0002
TCP11L2	t-complex 11 like 2	protein_coding	chr12:106301929-	6616	1.176056422	1.578548819	0.340664	9E-05	0.0002
SRM	spermidine synthase	protein_coding	chr1:11054584-11	1894	25.21607129	32.36804101	0.279577	9E-05	0.0002
ZNF14	zinc finger protein 14	protein_coding	chr19:19710472-1	2963	1.361883341	1.94977202	0.42998	9E-05	0.0002
TXN	thioredoxin	protein_coding	chr9:110243810-1 1010		36.99027561	49.15565613	0.325182	9E-05	0.0002
E2F5	E2F transcription factor 5	protein_coding	chr8:85177154-85	5999	1.5375106	2.031319866	0.317084	9E-05	0.0002
DRAP1	DR1 associated protein 1	protein_coding	chr11:65919274-6	1722	40.93573896	53.89242862	0.314567	9E-05	0.0002
DIPK1A	divergent protein kinase domain 1A	protein_coding	chr1:92832737-92	3091	2.470524449	3.364514034	0.358663	9E-05	0.0002
AC012645.1	novel transcript, antisense to YPEL3	lncRNA	chr16:30096430-3	1650	1.297562018	2.115136622	0.621033	9E-05	0.0002
ISCU	iron-sulfur cluster assembly enzyme	protein_coding	chr12:108562582-	4439	17.78279224	22.96133457	0.287429	9E-05	0.0002
SUMO2	small ubiquitin like modifier 2	protein_coding	chr17:75165586-7	3348	19.790493	25.17107304	0.264469	9E-05	0.0002
TMEM268	transmembrane protein 268	protein_coding	chr9:114611206-1	4792	0.78216836	1.097553696	0.400511	9E-05	0.0002
FGF13-AS1	FGF13 antisense RNA 1	lncRNA	chrX:138711452-1	1145	1.630409027	2.793287959	0.696949	9E-05	0.0002
PRIMPOL	primase and DNA directed polymerase	protein_coding	chr4:184649667-1	2789	2.719631684	3.61861379	0.325596	9E-05	0.0002
TESMIN	testis expressed metallothionein like prot	protein_coding	chr11:68707440-6	5789	0.944777097	1.314929564	0.391685	9E-05	0.0002
AP000873.2	novel transcript	lncRNA	chr11:83184491-8	3418	1.689904694	2.412603613	0.442041	9E-05	0.0002
ARHGAP31	Rho GTPase activating protein 31	protein_coding	chr3:119294373-1	9384	6.863423989	8.912589715	0.294065	9E-05	0.0002
TIPIN	TIMELESS interacting protein	protein_coding	chr15:66336191-6	2739	1.308112568	1.820150847	0.39234	1E-04	0.0002
NBPF11	NBPF member 11	protein_coding	chr1:148102046-1	5947	1.471428776	2.004178603	0.36787	1E-04	0.0002
C10orf88	chromosome 10 open reading frame 88	protein_coding	chr10:122930901-	3083	3.053334683	3.951113479	0.285468	1E-04	0.0002
ZNF461	zinc finger protein 461	protein_coding	chr19:36636618-3	4778	0.549058526	0.892468502	0.591998	1E-04	0.0002
AC005034.4	novel transcript	lncRNA	chr2:75719120-75	899	0.462942407	0.991810185	1.004115	1E-04	0.0002
AP006621.3	novel transcript	lncRNA	chr11:777578-784	1489	2.992081409	4.229996818	0.416191	1E-04	0.0002
COMMD7	COMM domain containing 7	protein_coding	chr20:32702691-3	2584	12.19470723	15.56802807	0.268033	0.0001	0.0002
CTXN1	cortexin 1	protein_coding	chr19:7924491-79	1237	11.57630452	15.76030436	0.35657	0.0001	0.0002
KLHL11	kelch like family member 11	protein_coding	chr17:41848518-4	7402	1.237892178	1.621333651	0.30069	0.0001	0.0002
TRIM21	tripartite motif containing 21	protein_coding	chr11:4384897-43	1935	2.395689648	3.387927273	0.412514	0.0001	0.0002
HIST1H2BJ	histone cluster 1 H2B family member j	protein_coding	chr6:27125897-27	950	1.185452004	2.175743267	0.779287	0.0001	0.0002
HSPB1	heat shock protein family B (small) memb	protein_coding	chr7:76302673-76	1505	1199.277142	1533.923837	0.271316	0.0001	0.0002
KIN	Kin17 DNA and RNA binding protein	protein_coding	chr10:7750962-77	6856	1.651856868	2.12617646	0.276462	0.0001	0.0002
PCDHB15	protocadherin beta 15	protein_coding	chr5:141245395-1	3971	0.747269423	1.257094553	0.677502	0.0001	0.0002
RAC3	Rac family small GTPase 3	protein_coding	chr17:82031678-8	1473	10.62581793	13.93415029	0.308147	0.0001	0.0002
RCAN3	RCAN family member 3	protein_coding	chr1:24502351-24	7138	0.886492831	1.219892829	0.370027	0.0001	0.0002
AGRN	agrin	protein_coding	chr1:1020120-105	10761	21.4677628	27.50178835	0.274461	0.0001	0.0002
RELA-DT	RELA divergent transcript	lncRNA	chr11:65663006-6	3728	0.861214583	1.248778924	0.450612	0.0001	0.0002
RNF227	ring finger protein 227	protein_coding	chr17:7913324-79	2953	0.716476518	1.227624665	0.708699	0.0001	0.0002
AC016650.1	novel transcript	lncRNA	chr5:16129178-16	708	4.601080839	6.811627698	0.479566	0.0001	0.0002
AC093752.2	novel transcript	lncRNA	chr4:119456350-1	573	0.549847615	1.351876598	1.186399	0.0001	0.0002
ICAM5	intercellular adhesion molecule 5	protein_coding	chr19:10289952-1	3935	5.914706146	7.588182736	0.276256	0.0001	0.0002
BX537318.1	novel transcript	lncRNA	chr22:46013606-4	1207	0.793602553	1.53557586	0.884632	0.0001	0.0002
CEND1	cell cycle exit and neuronal differentiatio	protein_coding	chr11:787115-790	1628	1.017541199	1.670006931	0.623955	0.0001	0.0002
FRG1	FSHD region gene 1	protein_coding	chr4:189940870-1	1623	6.932189627	9.93468047	0.449631	0.0001	0.0002
AC011445.1	novel transcript, sense overlapping LRFN1	lncRNA	chr19:39314651-3	877	0.489986253	1.130283793	1.101763	0.0001	0.0002
POP4	POP4 homolog, ribonuclease P/MRP subu	protein_coding	chr19:29606283-2	4308	4.519538102	5.99614661	0.323423	0.0001	0.0002
NME7	NME/NM23 family member 7	protein_coding	chr1:169132531-1	5718	0.958985968	1.315150266	0.369029	0.0001	0.0002
MED10	mediator complex subunit 10	protein_coding	chr5:6371874-637	1864	10.07523924	12.93068552	0.269337	0.0001	0.0002
ZNF354B	zinc finger protein 354B	protein_coding	chr5:178859953-1	3742	2.393201827	3.084310624	0.281589	0.0001	0.0002
FBXO16	F-box protein 16	protein_coding	chr8:28348287-28	3932	0.7620169	1.164048246	0.519268	0.0001	0.0002
MAML3	mastermind like transcriptional coactivat	protein_coding	chr4:139716753-1	7012	1.265604243	1.636392113	0.285658	0.0001	0.0002
HCG27	HLA complex group 27	lncRNA	chr6:31197760-31	2592	0.823258077	1.407958263	0.666822	0.0001	0.0002
QTRT2	queuine tRNA-ribosyltransferase accessor	protein_coding	chr3:114005833-1	8054	1.281361871	1.66798047	0.299662	0.0001	0.0002
A2M-AS1	A2M antisense RNA 1	lncRNA	chr12:9065163-90	2948	1.055840698	1.516970953	0.436779	0.0001	0.0002
MAP3K6	mitogen-activated protein kinase kinase k	protein_coding	chr1:27354067-27	5746	1.414167885	1.890870692	0.335371	0.0001	0.0002
KDM8	lysine demethylase 8	protein_coding	chr16:27203495-2	4039	0.984605722	1.387881648	0.412569	0.0001	0.0003
ABRACL	ABRA C-terminal like	protein_coding	chr6:139028745-1	1137	2.055203017	3.145271851	0.518133	0.0001	0.0003
ZNF30-AS1	ZNF30 antisense RNA 1	lncRNA	chr19:34923299-3	625	0.41025395	0.976560978	1.168538	0.0001	0.0003
TRADD	TNFRSF1A associated via death domain	protein_coding	chr16:67154185-6	2173	2.989438298	4.134116851	0.377267	0.0001	0.0003
SLC5A3	solute carrier family 5 member 3	protein_coding	chr21:34073578-3	11566	0.872662713	1.135486627	0.294527	0.0001	0.0003
AC027031.2	novel transcript	lncRNA	chr8:106270144-1	1422	0.737309797	1.496620736	0.983095	0.0001	0.0003
TBCCD1	TBCC domain containing 1	protein_coding	chr3:186546067-1	3398	2.61379454	3.388520771	0.288619	0.0001	0.0003
TSHZ2	teashirt zinc finger homeobox 2	protein_coding	chr20:52972358-5	12630	0.785309523	1.050577816	0.337494	0.0001	0.0003

LINC01003	long intergenic non-protein coding RNA 1	lncRNA	chr7:152463786-1	1764	3.991051342	5.57867275	0.397768	0.0001	0.0003
TSNARE1	t-SNARE domain containing 1	protein_coding	chr8:142212080-1	4339	2.597274175	3.321238484	0.268938	0.0001	0.0003
AC016727.1	novel transcript, antisense to XPO1	lncRNA	chr2:61471188-61	2409	1.22479028	1.866786791	0.52259	0.0001	0.0003
AC079922.2	novel transcript (FLJ42351)	lncRNA	chr2:112641832-1	3224	0.833402824	1.207976295	0.445475	0.0001	0.0003
CRLS1	cardiolipin synthase 1	protein_coding	chr20:6006090-60	5370	1.861908177	2.975836687	0.595787	0.0001	0.0003
AC018553.2	novel transcript, antisense to IRX3	lncRNA	chr16:54285604-5	1064	0.404164052	0.883493323	1.013258	0.0001	0.0003
CDCA4	cell division cycle associated 4	protein_coding	chr14:105009573-	2513	3.398071112	4.664784032	0.369861	0.0001	0.0003
AL355297.4	novel transcript, antisense to ARID1B	lncRNA	chr6:156776360-1	1249	1.358542906	2.43295757	0.742011	0.0001	0.0003
STXBP5	syntaxin binding protein 5	protein_coding	chr6:147204425-1	10504	1.081052032	1.415324038	0.302396	0.0001	0.0003
FKBP5	FKBP prolyl isomerase 5	protein_coding	chr6:35573585-35	10628	0.927017336	1.219052531	0.308097	0.0001	0.0003
TBC1D19	TBC1 domain family member 19	protein_coding	chr4:26576437-26	4138	1.805027336	2.540721809	0.411461	0.0001	0.0003
AC027307.1	novel transcript, antisense to ADAMTSL5	lncRNA	chr19:1508375-15	457	0.243152855	0.990042886	1.917597	0.0002	0.0003
IER5L	immediate early response 5 like	protein_coding	chr9:129175552-1	2711	2.963589135	3.964499463	0.345275	0.0002	0.0003
RWDD3	RWD domain containing 3	protein_coding	chr1:95234210-95	1927	2.781460671	3.791228011	0.360114	0.0002	0.0003
C12orf73	chromosome 12 open reading frame 73	protein_coding	chr12:103940763-	4435	2.463697601	3.194340975	0.290106	0.0002	0.0003
LRRC56	leucine rich repeat containing 56	protein_coding	chr11:537527-554	2765	0.596583991	0.927736724	0.544907	0.0002	0.0003
UBOX5	U-box domain containing 5	protein_coding	chr20:3107573-31	4738	2.667197479	3.414713111	0.276619	0.0002	0.0003
IFNAR2	interferon alpha and beta receptor subuni	protein_coding	chr21:33229901-3	4614	0.944814641	1.32510628	0.398265	0.0002	0.0003
AC097634.1	novel transcript	lncRNA	chr3:71584943-71	2467	0.795170444	1.212247407	0.512658	0.0002	0.0003
PTPRS	protein tyrosine phosphatase receptor typ	protein_coding	chr19:5158495-53	8565	12.28638068	15.98689599	0.300126	0.0002	0.0003
MOCS3	molybdenum cofactor synthesis 3	protein_coding	chr20:50958826-5	5106	2.110198484	2.745467298	0.288738	0.0002	0.0003
PTRH1	peptidyl-tRNA hydrolase 1 homolog	protein_coding	chr9:127690348-1	4072	1.607400791	2.190978322	0.362801	0.0002	0.0003
ZNF862	zinc finger protein 862	protein_coding	chr7:149838375-1	8422	2.649232248	3.371579304	0.265323	0.0002	0.0003
CEP41	centrosomal protein 41	protein_coding	chr7:130393771-1	12539	0.696727865	0.93606494	0.347154	0.0002	0.0003
FAM131A	family with sequence similarity 131 meml	protein_coding	chr3:184335926-1	8925	0.963360022	1.239592149	0.274462	0.0002	0.0003
HSD17B14	hydroxysteroid 17-beta dehydrogenase 14	protein_coding	chr19:48813018-4	1112	1.800530825	2.959101649	0.629942	0.0002	0.0003
ZNF718	zinc finger protein 718	protein_coding	chr4:124501-2023	5073	1.115443893	1.491992943	0.335398	0.0002	0.0003
BCDIN3D	BCDIN3 domain containing RNA methyltr	protein_coding	chr12:49836043-4	3694	1.313610102	1.8951668	0.443585	0.0002	0.0003
DICER1-AS1	DICER1 antisense RNA 1	lncRNA	chr14:95157645-9	5264	0.656513516	0.958925291	0.46241	0.0002	0.0003
ARL13B	ADP ribosylation factor like GTPase 13B	protein_coding	chr3:93980139-94	4154	1.312835346	1.749266106	0.324357	0.0002	0.0003
TEX22	testis expressed 22	protein_coding	chr14:105398538-	2878	0.58944616	1.009429618	0.687031	0.0002	0.0003
ZNF287	zinc finger protein 287	protein_coding	chr17:16546954-1	7997	1.042795792	1.330030468	0.263945	0.0002	0.0003
ZSCAN23	zinc finger and SCAN domain containing 2	protein_coding	chr6:28431930-28	3283	1.273613995	1.936016744	0.533747	0.0002	0.0003
AC004982.2	novel transcript	lncRNA	chr7:7552462-756	1730	1.4518079	2.306795143	0.585003	0.0002	0.0003
E2F8	E2F transcription factor 8	protein_coding	chr11:19224063-1	4134	6.226280812	7.969509136	0.273875	0.0002	0.0003
B3GNT10	UDP-GlcNAc:betaGal beta-1,3-N-acetylglu	protein_coding	chr9:120792403-1	2516	0.587802463	1.056422559	0.771041	0.0002	0.0003
FARS2	phenylalanyl-tRNA synthetase 2, mitocho	protein_coding	chr6:5261044-582	4835	1.929242871	2.52919778	0.298773	0.0002	0.0003
DMTN	dematin actin binding protein	protein_coding	chr8:22048995-22	4473	0.87307502	1.184849897	0.350891	0.0002	0.0003
FAM89B	family with sequence similarity 89 membe	protein_coding	chr11:65572349-6	1458	1.062136468	1.639529727	0.539118	0.0002	0.0004
UFSP1	UFM1 specific peptidase 1 (inactive)	protein_coding	chr7:100888721-1	995	2.44596035	3.516585165	0.434681	0.0002	0.0004
SLC22A15	solute carrier family 22 member 15	protein_coding	chr1:115976513-1	5277	2.507417646	3.257226392	0.291974	0.0002	0.0004
DGKE	diacylglycerol kinase epsilon	protein_coding	chr17:56834107-5	10925	0.920986967	1.210140833	0.310051	0.0002	0.0004
LCN9	lipocalin 9	protein_coding	chr9:135663322-1	1105	0.688437506	1.396835239	0.948262	0.0002	0.0004
ZNF561-AS1	ZNF561 antisense RNA 1 (head to head)	lncRNA	chr19:9621249-96	8073	1.05009563	1.354230819	0.279054	0.0002	0.0004
ETHE1	ETHE1 persulfide dioxygenase	protein_coding	chr19:43506719-4	1590	4.573803053	5.995277469	0.301256	0.0002	0.0004
ARHGAP39	Rho GTPase activating protein 39	protein_coding	chr8:144529179-1	4888	3.023917941	3.960571431	0.302179	0.0002	0.0004
LHPP	phospholysine phosphohistidine inorgani	protein_coding	chr10:124461823-	4075	2.029502422	2.672247051	0.311323	0.0002	0.0004
ERICH2	glutamate rich 2	protein_coding	chr2:170783786-1	601	1.689799916	2.911153239	0.696703	0.0002	0.0004
ABCC10	ATP binding cassette subfamily C member	protein_coding	chr6:43427366-43	8087	3.17560103	4.072479578	0.275617	0.0002	0.0004
RHOG	ras homolog family member G	protein_coding	chr11:3826978-38	1758	7.095608348	9.265049741	0.298771	0.0002	0.0004
AC017100.1	novel transcript, antisense to B4GALT6	lncRNA	chr18:31685655-3	1169	1.087369811	1.718310342	0.571643	0.0002	0.0004
TET1	tet methylcytosine dioxygenase 1	protein_coding	chr10:68560337-6	9612	3.113451614	4.071591925	0.303296	0.0002	0.0004
PCSK4	proprotein convertase subtilisin/kexin typ	protein_coding	chr19:1481428-14	3792	0.683782379	0.977205391	0.423203	0.0002	0.0004
ENDOD1	endonuclease domain containing 1	protein_coding	chr11:95089846-9	4651	1.1412006	1.65645888	0.440081	0.0002	0.0004
MECR	mitochondrial trans-2-enoyl-CoA reducta	protein_coding	chr1:29192873-29	3922	5.702583921	7.281049933	0.265708	0.0002	0.0004
GLB1L	galactosidase beta 1 like	protein_coding	chr2:219236598-2	3419	1.008980565	1.494702749	0.486512	0.0002	0.0004
ATPSCKMT	ATP synthase c subunit lysine N-methyltra	protein_coding	chr5:10225507-10	3285	3.565661262	4.576343704	0.275095	0.0002	0.0004
HENMT1	HEN methyltransferase 1	protein_coding	chr1:108648290-1	2426	0.56975721	0.965752077	0.672922	0.0002	0.0004
BCL10	BCL10 immune signaling adaptor	protein_coding	chr1:85265776-85	4346	1.665224228	2.272898463	0.362633	0.0002	0.0004
C1D	C1D nuclear receptor corepressor	protein_coding	chr2:68041130-68	3581	2.722417751	3.641207477	0.336456	0.0002	0.0004
MAP3K15	mitogen-activated protein kinase kinase k	protein_coding	chrX:19360056-19	5573	0.782051854	1.075154921	0.36771	0.0002	0.0004
HACL1	2-hydroxyacyl-CoA lyase 1	protein_coding	chr3:15560704-15	3055	3.843402182	4.91734944	0.272096	0.0002	0.0004
AL133243.2	novel transcript, sense intronic to BIRC6	lncRNA	chr2:32521927-32	1621	1.021457217	1.617222638	0.573824	0.0002	0.0004
NCCRP1	non-specific cytotoxic cell receptor prote	protein_coding	chr19:39196964-3	1975	0.667954905	1.103321043	0.655881	0.0002	0.0004
C9orf40	chromosome 9 open reading frame 40	protein_coding	chr9:74946583-74	2351	3.000494193	3.935834902	0.307919	0.0002	0.0005
DBNDD2	dysbindin domain containing 2	protein_coding	chr20:45406057-4	2764	1.278623134	1.881739709	0.478815	0.0003	0.0005
MTRF1	mitochondrial translation release factor 1	protein_coding	chr13:41216369-4	3841	1.870396808	2.514258397	0.345241	0.0003	0.0005
AC015871.3	novel transcript, sense intronic to ST20	lncRNA	chr15:79920195-7	2261	2.614100391	3.383228192	0.284728	0.0003	0.0005
PFKL	phosphofructokinase, liver type	protein_coding	chr21:44300051-4	8926	10.39547668	13.21946406	0.264997	0.0003	0.0005
KDELR3	KDEL endoplasmic reticulum protein rete	protein_coding	chr22:38468078-3	1913	3.323411887	4.358585022	0.307651	0.0003	0.0005
MROH7	maestro heat like repeat family member 7	protein_coding	chr1:54641754-54	9088	2.294868897	2.949337689	0.274933	0.0003	0.0005
AC023157.3	novel transcript, antisense to AMN1	lncRNA	chr12:31729117-3	2088	0.687170831	1.119254313	0.613111	0.0003	0.0005
B3GNT8	UDP-GlcNAc:betaGal beta-1,3-N-acetylglu	protein_coding	chr19:41425359-4	1874	5.775957372	7.542143609	0.304927	0.0003	0.0005
AL356488.3	novel transcript, antisense to TMEM167B	lncRNA	chr1:109087971-1	2888	1.666340782	2.284465771	0.375953	0.0003	0.0005
MT1X	metallothionein 1X	protein_coding	chr16:56682470-5	1727	0.808029491	1.285752054	0.593271	0.0003	0.0005
DNAH10OS	dynein axonemal heavy chain 10 opposite	lncRNA	chr12:123925461-	6003	1.601560301	2.083568009	0.297998	0.0003	0.0005
NUDT13	nudix hydrolase 13	protein_coding	chr10:73110375-7	2521	1.288529608	1.800646125	0.394827	0.0003	0.0005
AC016394.1	novel transcript, antisense to TTC18	lncRNA	chr10:73252791-7	940	1.398276842	2.26887586	0.60235	0.0003	0.0005
AC020915.3	novel transcript, antisense to ZNF544	lncRNA	chr19:58257270-5	2708	1.741237216	2.321292315	0.328479	0.0003	0.0005
SDSL	serine dehydratase like	protein_coding	chr12:113422380-	2073	4.635367886	6.215532069	0.325887	0.0003	0.0005
ZNF337	zinc finger protein 337	protein_coding	chr20:25673195-2	4855	3.435891827	4.443724664	0.288312	0.0003	0.0005
GPR153	G protein-coupled receptor 153	protein_coding	chr1:6247353-626	4198	2.153374521	2.875236224	0.332026	0.0003	0.0005
SFMBT1	Scm like with four mbt domains 1	protein_coding	chr3:52903572-53	5681	1.477137102	1.96049598	0.323933	0.0003	0.0005
TRMT61B	tRNA methyltransferase 61B	protein_coding	chr2:28849821-28	2064	4.137621677	5.523706653	0.322265	0.0003	0.0005
FAM122A	family with sequence similarity 122A	protein_coding	chr9:68780034-68	4575	0.893195414	1.244674825	0.395537	0.0003	0.0005
JMJD1C-AS1	JMJD1C antisense RNA 1	lncRNA	chr10:63465229-6	1335	1.559756983	2.319475037	0.481196	0.0003	0.0006
ADRA2B	adrenoceptor alpha 2B	protein_coding	chr2:96112876-96	3696	0.804860957	1.19227157	0.468543	0.0003	0.0006
BFSP1	beaded filament structural protein 1	protein_coding	chr20:17493905-1	3371	0.879721145	1.241796549	0.406706	0.0003	0.0006
ZFHX4	zinc finger homeobox 4	protein_coding	chr8:76681239-76	15855	1.446975684	1.886008977	0.301558	0.0003	0.0006
TRRAP	transformation/transcription domain ass	protein_coding	chr7:98877933-99	19972	5.831172944	7.557236598	0.294975	0.0003	0.0006
Z83843.1	novel transcript, sense intronic FTX	lncRNA	chrX:74209976-74	3685	2.519038998	3.331694574	0.326081	0.0003	0.0006
ANKRD10-IT1	ANKRD10 intronic transcript 1	lncRNA	chr13:110894639-	800	9.121153222	12.33227397	0.34542	0.0004	0.0006
FRAT1	FRAT regulator of WNT signaling pathway	protein_coding	chr10:97319267-9	2649	1.436802999	2.015834337	0.406199	0.0004	0.0006
EFNA4	ephrin A4	protein_coding	chr1:155063737-1	1257	2.769823069	3.823025075	0.377752	0.0004	0.0006
AC137767.1	novel transcript	lncRNA	chr12:123363868-	2246	0.815108506	1.307228314	0.584516	0.0004	0.0006
ZNF416	zinc finger protein 416	protein_coding	chr19:57571566-5	2545	1.413180974	2.008898904	0.419749	0.0004	0.0006
ZNF789	zinc finger protein 789	protein_coding	chr7:99472890-99	6645	1.30961111	1.669678566	0.264218	0.0004	0.0006
AP001528.1	novel transcript	lncRNA	chr11:86955616-8	1988	0.72467243	1.181053446	0.599206	0.0004	0.0006
NR2F6	nuclear receptor subfamily 2 group F men	protein_coding	chr19:17231883-1	2676	26.5937102	34.79519889	0.297682	0.0004	0.0006
DUSP18	dual specificity phosphatase 18	protein_coding	chr22:30652051-3	4450	0.730313492	1.019580375	0.394455	0.0004	0.0007
IZUMO4	IZUMO family member 4	protein_coding	chr19:2096429-20	2770	0.893752815	1.362551856	0.527939	0.0004	0.0007
CHCHD5	coiled-coil-helix-coiled-coil-helix domain	protein_coding	chr2:112584240-1	3759	4.011720775	5.224598195	0.305339	0.0004	0.0007
LENG8	leukocyte receptor cluster member 8	protein_coding	chr19:54448887-5	6469	43.55215662	57.2879224	0.311309	0.0004	0.0007
TIGD7	tigger transposable element derived 7	protein_coding	chr16:3298808-33	4140	1.690631277	2.224225121	0.301437	0.0004	0.0007
CIART	circadian associated repressor of transcrip	protein_coding	chr1:150282543-1	2849	2.190975544	3.010005551	0.380021	0.0004	0.0007
POGLUT3	protein O-glucosyltransferase 3	protein_coding	chr11:108472112-	4938	1.814348749	2.342844648	0.282035	0.0004	0.0007
AL445222.1	novel transcript	lncRNA	chr9:127366770-1	628	0.784790932	1.66428462	1.026185	0.0004	0.0007
SNHG18	small nucleolar RNA host gene 18	lncRNA	chr5:9546200-955	1951	7.119218791	9.074949003	0.263773	0.0004	0.0007
CTBS	chitobiase	protein_coding	chr1:84549611-84	6934	1.24205908	1.620140485	0.296547	0.0004	0.0007
TMEM102	transmembrane protein 102	protein_coding	chr17:7435443-74	2129	1.092967977	1.660440293	0.523535	0.0004	0.0007
AC021087.1	novel transcript	lncRNA	chr5:269858-2715	522	0.21620608	0.699062668	1.554853	0.0004	0.0007

CRYL1	crystallin lambda 1	protein_coding	chr13:20403666-2	4327	1.715195137	2.277339489	0.336646	0.0004	0.0007
LINC01629	long intergenic non-protein coding RNA 1	lncRNA	chr14:76959638-7	581	10.38708932	14.30222195	0.372013	0.0004	0.0007
CEP19	centrosomal protein 19	protein_coding	chr3:196706277-1	2158	1.042196954	1.587158337	0.523653	0.0004	0.0007
AP002387.2	novel transcript	lncRNA	chr11:71448674-7	680	2.901796688	4.350535621	0.509805	0.0004	0.0007
CEP112	centrosomal protein 112	protein_coding	chr17:65635537-6	7899	0.804905625	1.053305105	0.300934	0.0004	0.0007
WWOX	WW domain containing oxidoreductase	protein_coding	chr16:78099430-7	4869	1.358863797	1.814411451	0.340167	0.0004	0.0007
AC090114.2	novel transcript	lncRNA	chr7:128524016-1	7054	1.785636446	2.301217366	0.27546	0.0004	0.0007
TSSK6	testis specific serine kinase 6	protein_coding	chr19:19512418-1	1666	0.971479386	1.504442148	0.529226	0.0004	0.0007
PANCR	PITX2 adjacent non-coding RNA	lncRNA	chr4:110595513-1	832	2.210042472	3.359503602	0.517092	0.0004	0.0007
STX1A	syntaxin 1A	protein_coding	chr7:73699206-73	3693	0.770704001	1.093884673	0.41895	0.0004	0.0008
COPZ2	coatomer protein complex subunit zeta 2	protein_coding	chr17:48026167-4	1866	3.55089191	4.592578929	0.281887	0.0004	0.0008
AL596442.3	novel transcript	lncRNA	chr6:170158728-1	659	0.3568738	1.033292838	1.49528	0.0004	0.0008
B3GNT7	UDP-GlcNAc:betaGal beta-1,3-N-acetylglu	protein_coding	chr2:231395710-2	3639	0.951409298	1.408808921	0.479635	0.0004	0.0008
VASH1-AS1	VASH1 antisense RNA 1	lncRNA	chr14:76781733-7	1843	1.775125269	2.455740745	0.378977	0.0005	0.0008
SPSB2	splA/ryanodine receptor domain and SOC	protein_coding	chr12:6870935-68	2452	1.200534295	1.695994296	0.412319	0.0005	0.0008
SNHG12	small nucleolar RNA host gene 12	lncRNA	chr1:28578538-28	4266	0.73768543	1.012322609	0.369889	0.0005	0.0008
DTX2	deltex E3 ubiquitin ligase 2	protein_coding	chr7:76461676-76	5042	1.323606071	1.724816706	0.297334	0.0005	0.0008
TMEM182	transmembrane protein 182	protein_coding	chr2:102736905-1	6084	0.882396262	1.276477373	0.427477	0.0005	0.0008
SPEN	spen family transcriptional repressor	protein_coding	chr1:15847707-15	13447	4.517547458	5.765016008	0.272431	0.0005	0.0008
AL031848.2	novel transcript, antisense to ACOT7	lncRNA	chr1:6393555-639	837	0.393179734	0.900341205	1.061115	0.0005	0.0009
RIOX1	ribosomal oxygenase 1	protein_coding	chr14:73490964-7	2429	6.483069786	8.300984506	0.266775	0.0005	0.0009
TIAF1	TGFB1-induced anti-apoptotic factor 1	protein_coding	chr17:29073521-2	5337	2.724704524	3.471484082	0.26577	0.0005	0.0009
GCC2-AS1	GCC2 antisense RNA 1	lncRNA	chr2:108507515-1	799	0.47883791	1.047459806	1.013363	0.0005	0.0009
NUP50-DT	NUP50 divergent transcript	lncRNA	chr22:45133020-4	3141	3.180961619	4.124888174	0.297353	0.0005	0.0009
RAB29	RAB29, member RAS oncogene family	protein_coding	chr1:205767986-2	3904	2.065174572	2.746761147	0.339993	0.0005	0.0009
AC010654.1	novel transcript	lncRNA	chr16:11797468-1	808	0.835194396	1.559943123	0.858956	0.0005	0.0009
PRELID3A	PRELI domain containing 3A	protein_coding	chr18:12407896-1	2643	1.396855636	1.927111494	0.366273	0.0005	0.0009
CHRNB1	cholinergic receptor nicotinic beta 1 subu	protein_coding	chr17:7445061-74	4332	1.711385286	2.239742303	0.300524	0.0005	0.0009
AP006621.1	novel transcript	lncRNA	chr11:823634-832	792	1.329767299	2.259477286	0.679289	0.0006	0.0009
HIST1H2AH	histone cluster 1 H2A family member h	protein_coding	chr6:27147129-27	387	0.502047209	1.142294491	1.076513	0.0006	0.0009
EGFL7	EGF like domain multiple 7	protein_coding	chr9:136658856-1	2564	0.851209356	1.218973775	0.439773	0.0006	0.001
PLK3	polo like kinase 3	protein_coding	chr1:44800377-44	3327	0.960101366	1.33089818	0.393631	0.0006	0.001
EMID1	EMI domain containing 1	protein_coding	chr22:29205896-2	4854	1.259712217	1.660690378	0.316384	0.0006	0.001
AC007066.2	novel transcript, overlapping RABGAP1	lncRNA	chr9:122937623-1	2674	0.792631573	1.192975135	0.495031	0.0006	0.001
AL354920.1	novel transcript, antisense to UBQLN1	lncRNA	chr9:83707594-83	1134	1.720741613	2.780800823	0.631152	0.0006	0.001
OLFML3	olfactomedin like 3	protein_coding	chr1:113979391-1	2821	0.870287945	1.267996356	0.454267	0.0006	0.001
PLEC	plectin	protein_coding	chr8:143915147-1	16991	22.35766089	28.6582103	0.278753	0.0006	0.001
HDAC4-AS1	HDAC4 antisense RNA 1	lncRNA	chr2:239401436-2	613	1.042555461	1.966721814	0.816261	0.0006	0.001
FBXO4	F-box protein 4	protein_coding	chr5:41925254-41	3014	0.628034374	1.003143759	0.565246	0.0006	0.001
AL133346.1	novel transcript	lncRNA	chr6:131950946-1	495	0.835065667	1.535586327	0.796511	0.0006	0.0011
ALMS1	ALMS1 centrosome and basal body associ	protein_coding	chr2:73385758-73	14783	1.628219336	2.159211771	0.32565	0.0006	0.0011
ST20	suppressor of tumorigenicity 20	protein_coding	chr15:79898840-7	1614	1.636555458	2.45558737	0.511215	0.0006	0.0011
GRTP1	growth hormone regulated TBC protein 1	protein_coding	chr13:113324164-	2916	0.877479593	1.322898618	0.518277	0.0006	0.0011
RABIF	RAB interacting factor	protein_coding	chr1:202878957-2	2430	3.612670405	4.761718112	0.308949	0.0006	0.0011
FAM133A	family with sequence similarity 133 meml	protein_coding	chrX:93674013-93	3381	0.778209266	1.147155336	0.480017	0.0007	0.0011
AC073073.2	novel transcript, antisense to EFCAB10	lncRNA	chr7:105571083-1	2578	0.727860436	1.058645825	0.464006	0.0007	0.0011
AC007114.1	novel transcript	lncRNA	chr17:57071814-5	1540	0.931766321	1.458299015	0.564652	0.0007	0.0011
AC124016.1	novel transcript, antisense to HNRNPD	lncRNA	chr4:82374301-82	906	2.282079151	3.319624241	0.449916	0.0007	0.0011
TOR4A	torsin family 4 member A	protein_coding	chr9:137277726-1	4171	2.367536961	3.040023857	0.28467	0.0007	0.0012
MAIP1	matrix AAA peptidase interacting protein	protein_coding	chr2:199955317-2	1624	7.3864517	9.441876979	0.2689	0.0007	0.0012
AC107027.3	novel transcript, overlapping to MRPL3	lncRNA	chr3:131455126-1	3473	0.785614636	1.105064933	0.402211	0.0007	0.0012
RAB13	RAB13, member RAS oncogene family	protein_coding	chr1:153981617-1	2751	3.194137881	4.119535835	0.280131	0.0008	0.0013
MERTK	MER proto-oncogene, tyrosine kinase	protein_coding	chr2:111898479-1	4700	1.054760874	1.404155192	0.320867	0.0008	0.0013
AL117379.1	novel transcript	lncRNA	chr20:62928621-6	677	0.893268365	1.736211122	0.883697	0.0008	0.0013
ZNF593	zinc finger protein 593	protein_coding	chr1:26169908-26	732	13.55641927	18.92999287	0.397761	0.0008	0.0013
DNMT3B	DNA methyltransferase 3 beta	protein_coding	chr20:32762385-3	4630	1.429096806	1.833097056	0.271792	0.0008	0.0013
TFAP4	transcription factor AP-4	protein_coding	chr16:4257186-42	7867	0.895166271	1.165801277	0.289626	0.0008	0.0013
EPCAM	epithelial cell adhesion molecule	protein_coding	chr2:47345158-47	2519	0.826310364	1.242070416	0.504086	0.0008	0.0013
SOS1-IT1	SOS1 intronic transcript 1	lncRNA	chr2:38992279-38	1254	2.672716773	3.667696859	0.372557	0.0008	0.0014
ADPRM	ADP-ribose/CDP-alcohol diphosphatase, r	protein_coding	chr17:10697594-1	1620	1.454658352	2.043976714	0.404113	0.0008	0.0014
LEF1	lymphoid enhancer binding factor 1	protein_coding	chr4:108047545-1	5361	1.03755312	1.415035107	0.365031	0.0008	0.0014
ZSWIM9	zinc finger SWIM-type containing 9	protein_coding	chr19:48170680-4	3763	4.023170617	5.309768867	0.321568	0.0008	0.0014
AL158835.2	novel transcript	lncRNA	chr10:125718771-	300	0.375448511	1.106680036	1.33012	0.0008	0.0014
MRPS30-DT	MRPS30 divergent transcript	lncRNA	chr5:44742420-44	4749	1.275657581	1.701711636	0.335968	0.0008	0.0014
ISY1	ISY1 splicing factor homolog	protein_coding	chr3:129127415-1	4102	1.553405345	2.036288412	0.297673	0.0009	0.0014
UNC5CL	unc-5 family C-terminal like	protein_coding	chr6:41026895-41	3156	1.260323504	1.70804069	0.348047	0.0009	0.0015
TSPAN33	tetraspanin 33	protein_coding	chr7:129144707-1	3530	0.616390636	0.957116558	0.571362	0.0009	0.0015
OSGEPL1-AS1	OSGEPL1 antisense RNA 1	lncRNA	chr2:189762704-1	860	0.437320879	0.842363202	0.827712	0.0009	0.0015
LINC01355	long intergenic non-protein coding RNA 1	lncRNA	chr1:23281307-23	6182	1.355371969	1.735753282	0.278453	0.0009	0.0015
NEK3	NIMA related kinase 3	protein_coding	chr13:52132639-5	4272	1.464696327	1.910463688	0.29962	0.0009	0.0015
HM13-AS1	HM13 antisense RNA 1	lncRNA	chr20:31567707-3	489	0.547319264	1.123814561	1.009577	0.001	0.0016
LINC00648	long intergenic non-protein coding RNA 6	lncRNA	chr14:47764954-4	3253	0.900587897	1.333758219	0.477805	0.001	0.0016
XK	X-linked Kx blood group	protein_coding	chrX:37685791-37	5174	1.685614599	2.181803345	0.284272	0.001	0.0017
CCNQ	cyclin Q	protein_coding	chrX:153587925-1	1677	6.874415565	8.884114935	0.283613	0.001	0.0017
FAM161B	FAM161 centrosomal protein B	protein_coding	chr14:73931501-7	5056	1.198505258	1.566798046	0.309665	0.001	0.0017
ZNF573	zinc finger protein 573	protein_coding	chr19:37735833-3	4689	0.564974354	0.848889921	0.498603	0.001	0.0017
HSCB	HscB mitochondrial iron-sulfur cluster co	protein_coding	chr22:28742039-2	1454	4.972450969	6.499772461	0.293767	0.0011	0.0017
LCA5	lebercilin LCA5	protein_coding	chr6:79484991-79	5964	0.727415972	0.980208977	0.3458	0.0011	0.0018
OSCP1	organic solute carrier partner 1	protein_coding	chr1:36415827-36	2788	1.778406813	2.299225613	0.292217	0.0011	0.0018
AC139768.2	novel transcript	lncRNA	chr12:51201684-5	898	1.544103183	2.494497721	0.612809	0.0011	0.0018
SPTY2D1OS	SPTY2D1 opposite strand	protein_coding	chr11:18588781-1	2283	0.893634208	1.292953372	0.441702	0.0012	0.0019
ZNF782	zinc finger protein 782	protein_coding	chr9:96816472-96	4622	0.756862731	1.004035489	0.32242	0.0012	0.0019
C8orf48	chromosome 8 open reading frame 48	protein_coding	chr8:13566869-13	1420	0.467716812	0.938110447	0.873192	0.0012	0.0019
AC090587.2	novel transcript, sense overlapping STIM1	lncRNA	chr11:3854612-38	527	0.991633268	1.996153048	0.957315	0.0012	0.0019
ZKSCAN2-DT	ZKSCAN2 divergent transcript	lncRNA	chr16:25257952-2	3115	0.744901803	1.071312996	0.428082	0.0012	0.002
NOXA1	NADPH oxidase activator 1	protein_coding	chr9:137423350-1	1678	3.07559106	4.037559954	0.309483	0.0012	0.002
AC092687.3	novel transcript	lncRNA	chr2:10767875-10	393	0.239345382	0.796917452	1.696477	0.0013	0.0021
BICC1	BicC family RNA binding protein 1	protein_coding	chr10:58513140-5	6569	1.157333284	1.586852576	0.379296	0.0013	0.0021
AL139288.1	novel transcript	lncRNA	chr1:228407365-2	485	2.900308679	4.274848244	0.459933	0.0013	0.0022
AL731577.2	novel transcript, sense overlapping ZRANE	lncRNA	chr10:124917143-	555	2.790872549	5.443452508	0.900824	0.0013	0.0022
AL355338.1	novel transcript	lncRNA	chr13:100053074-	2077	3.291813635	4.218125372	0.276382	0.0014	0.0022
SLC6A13	solute carrier family 6 member 13	protein_coding	chr12:220621-262	4243	0.777054901	1.043576372	0.33995	0.0014	0.0022
VMAC	vimentin type intermediate filament asso	protein_coding	chr19:5904858-59	2254	1.737362902	2.444016671	0.408423	0.0014	0.0023
AL590062.1	novel transcript	lncRNA	chr6:27018935-27	1346	0.614766645	1.102897439	0.776841	0.0015	0.0023
AC024257.4	novel transcript	lncRNA	chr12:48360920-4	458	0.340993803	0.871243301	1.232055	0.0015	0.0024
PTPRF	protein tyrosine phosphatase receptor typ	protein_coding	chr1:43525187-43	10712	1.160346813	1.479185083	0.270642	0.0015	0.0024
AC053513.2	novel transcript	lncRNA	chr12:22460519-2	738	1.24912513	2.068883864	0.674573	0.0015	0.0024
AC245407.2	novel transcript	lncRNA	chr1:146387202-1	668	1.346085743	2.185653138	0.599351	0.0015	0.0024
DFFB	DNA fragmentation factor subunit beta	protein_coding	chr1:3857267-388	3870	1.000844059	1.438642754	0.438656	0.0015	0.0025
LTC4S	leukotriene C4 synthase	protein_coding	chr5:179793980-1	1192	0.817051018	1.432239454	0.721521	0.0015	0.0025
MAFG-DT	MAFG divergent transcript	lncRNA	chr17:81927829-8	1895	2.28434214	3.053324737	0.345748	0.0016	0.0025
NLGN3	neuroligin 3	protein_coding	chrX:71144831-71	4006	0.773758808	1.10594923	0.438275	0.0016	0.0025
TRMT10B	tRNA methyltransferase 10B	protein_coding	chr9:37753803-37	2866	1.501412754	1.970924025	0.303015	0.0016	0.0025
GUSBP11	GUSB pseudogene 11	lncRNA	chr22:23638487-2	7764	1.01625148	1.313624049	0.285904	0.0016	0.0026
AL121749.2	novel transcript, antisense to FZD8	lncRNA	chr10:35641097-3	1350	1.150524837	1.622722058	0.406389	0.0016	0.0026
AL022328.4	novel transcript, antisense TRABD	lncRNA	chr22:50199090-5	1001	1.452225375	2.191199516	0.491291	0.0016	0.0026
ZFP30	ZFP30 zinc finger protein	protein_coding	chr19:37613749-3	7705	1.793062359	2.303165792	0.27202	0.0016	0.0026
GLUD2	glutamate dehydrogenase 2	protein_coding	chrX:121047588-1	2493	1.530776328	2.339953967	0.553913	0.0016	0.0026
THAP2	THAP domain containing 2	protein_coding	chr12:71664301-7	5240	1.615732762	2.059438039	0.279236	0.0017	0.0027
ZNF485	zinc finger protein 485	protein_coding	chr10:43606419-4	2386	0.756923902	1.080661529	0.425851	0.0017	0.0027
AL354811.1	novel transcript, antisense to GPC6	lncRNA	chr13:93226612-9	706	1.491490474	2.317269195	0.551607	0.0018	0.0028

SLC35G1	solute carrier family 35 member G1	protein_coding	chr10:93893973-9	6829	1.005641668	1.318354194	0.303327	0.0018	0.0029
TRAF3IP2-AS1	TRAF3IP2 antisense RNA 1	lncRNA	chr6:111483511-1	4819	0.923103932	1.252022253	0.363164	0.0018	0.0029
TMEM44	transmembrane protein 44	protein_coding	chr3:194587673-1	4561	1.078810664	1.420201784	0.304943	0.0019	0.0029
AC011450.1	novel transcript	lncRNA	chr19:49331525-4	707	1.032114348	1.659349664	0.589611	0.0019	0.003
FAM227B	family with sequence similarity 227 meml	protein_coding	chr15:49326962-4 6020		1.119579259	1.487067281	0.318511	0.0019	0.003
AL355297.3	novel transcript	lncRNA	chr6:156774217-1	446	0.632604166	1.321750923	0.941278	0.0019	0.0031
HSD11B1L	hydroxysteroid 11-beta dehydrogenase 1	protein_coding	chr19:5680604-56	2991	2.014031776	2.651176848	0.304519	0.002	0.0031
AC019080.5	novel transcript	lncRNA	chr2:177317715-1	757	2.106361705	3.228178449	0.502311	0.0021	0.0032
AC026471.4	novel transcript	lncRNA	chr16:31449535-3	449	4.028194659	5.786311481	0.4486	0.0021	0.0033
AC018690.1	novel transcript	lncRNA	chr2:99405218-99	626	0.593614867	1.074159018	0.785706	0.0021	0.0033
STAC3	SH3 and cysteine rich domain 3	protein_coding	chr12:57243453-5	2091	0.682905948	0.991240797	0.444318	0.0021	0.0033
AC016394.2	novel transcript to NUDT13	lncRNA	chr10:73124573-7	960	0.561809895	0.921673394	0.608618	0.0022	0.0034
RGS14	regulator of G protein signaling 14	protein_coding	chr5:177357924-1	3705	1.010606348	1.351248295	0.330937	0.0022	0.0035
RAMACL	RNA guanine-7 methyltransferase activati	protein_coding	chr6:166585604-1	1991	0.720375914	1.065983591	0.468875	0.0022	0.0035
AC022916.1	novel transcript, antisense SLFN5	lncRNA	chr17:35231450-3	2814	0.978472145	1.324011824	0.343152	0.0023	0.0036
Z82243.1	novel transcript	lncRNA	chr22:45155539-4	473	0.917600091	1.699337164	0.761553	0.0023	0.0036
AC009812.1	novel transcript, antisense to ZBTB10	lncRNA	chr8:80484561-80	1672	1.295804421	1.893334938	0.46649	0.0024	0.0037
HOXB-AS1	HOXB cluster antisense RNA 1	lncRNA	chr17:48543551-4	1559	2.248517226	2.996362838	0.333439	0.0024	0.0038
AP001267.3	novel transcript, antisense to MLL and TTC	lncRNA	chr11:118510273-	2692	1.871663128	2.501461047	0.323968	0.0025	0.0038
NUDT17	nudix hydrolase 17	protein_coding	chr1:145845630-1	1657	2.984563221	3.823283205	0.265757	0.0025	0.0038
AL589986.2	novel transcript	lncRNA	chr1:152205858-1	397	0.35525959	0.825697422	1.096195	0.0025	0.0039
PHOSPHO2	phosphatase, orphan 2	protein_coding	chr2:169694454-1	1488	0.876628506	1.37195082	0.554596	0.0025	0.0039
AC016586.2	novel transcript, sense intronic to ZBTB7A	lncRNA	chr19:4061615-40	684	0.778525052	1.475658165	0.812765	0.0025	0.0039
GINS3	GINS complex subunit 3	protein_coding	chr16:58295080-5	5105	0.884792891	1.153653418	0.303471	0.0026	0.004
HMGN3-AS1	HMGN3 antisense RNA 1	lncRNA	chr6:79233718-79	2946	1.370013755	1.802397278	0.305186	0.0026	0.004
RAI2	retinoic acid induced 2	protein_coding	chrX:17800049-17	2622	0.984927926	1.376612052	0.391387	0.0027	0.0041
AL353803.5	novel transcript	lncRNA	chr9:129328261-1	141	0.349182608	1.1010031	1.872419	0.0027	0.0041
FUT10	fucosyltransferase 10	protein_coding	chr8:33370824-33	5606	0.953018743	1.260957327	0.314153	0.0027	0.0042
LCN12	lipocalin 12	protein_coding	chr9:136949551-1	2862	2.323237843	3.151588927	0.357229	0.0028	0.0044
AC009403.1	novel transcript, antisense to RBM33	lncRNA	chr7:155611231-1	2031	0.718122603	1.104833249	0.509456	0.0029	0.0044
AL035587.2	novel transcript, antisense to CNPY3	lncRNA	chr6:42928287-42	653	1.253247397	2.042325408	0.615015	0.0029	0.0045
FAM229A	family with sequence similarity 229 meml	protein_coding	chr1:32361270-32	2845	2.211983186	2.900900636	0.295431	0.003	0.0045
DM1-AS	DM1 locus antisense RNA	lncRNA	chr19:45767796-4	1027	0.737304886	1.227953048	0.673329	0.003	0.0046
SWSAP1	SWIM-type zinc finger 7 associated protei	protein_coding	chr19:11374685-1	1684	1.244282055	1.771442282	0.42306	0.003	0.0046
FALEC	focally amplified long non-coding RNA in	lncRNA	chr1:150515757-1	566	0.726541941	1.539348594	0.981407	0.003	0.0046
ZNF830	zinc finger protein 830	protein_coding	chr17:34961540-3	2238	5.051168002	6.488816714	0.26846	0.003	0.0047
C8orf58	chromosome 8 open reading frame 58	protein_coding	chr8:22599599-22	3319	1.253360243	1.697577606	0.350321	0.0031	0.0048
AC007773.1	novel transcript, antisense to DPY19L3	lncRNA	chr19:32390050-3	972	1.19475816	1.716310215	0.433474	0.0031	0.0048
AC004706.3	novel protein	protein_coding	chr17:6628619-66	4843	0.601211898	0.98484661	0.671592	0.0032	0.0049
AL109615.4	novel transcript	lncRNA	chr6:44090787-44	4799	0.765998597	1.027099673	0.331408	0.0033	0.005
AC006504.7	novel transcript, sense intronic to novel t	lncRNA	chr19:27802838-2	635	2.795637391	4.012795429	0.440092	0.0033	0.005
AC008462.1	novel transcript	lncRNA	chr5:108382156-1	681	0.819611098	1.387217573	0.661054	0.0033	0.0051
PEX11G	peroxisomal biogenesis factor 11 gamma	protein_coding	chr19:7476875-74	1482	1.904184956	2.617577943	0.359746	0.0034	0.0051
CCDC120	coiled-coil domain containing 120	protein_coding	chrX:49053572-49	5318	0.980593076	1.244218606	0.266568	0.0034	0.0052
FAIM	Fas apoptotic inhibitory molecule	protein_coding	chr3:138608606-1	4072	0.906503353	1.175180918	0.292339	0.0035	0.0053
SS18L2	SS18 like 2	protein_coding	chr3:42581840-42	1298	3.409014343	4.564863186	0.332343	0.0035	0.0053
PRAF2	PRA1 domain family member 2	protein_coding	chrX:49071161-49	1662	2.039606827	2.871732751	0.396796	0.0036	0.0054
ZNF438	zinc finger protein 438	protein_coding	chr10:30820207-3	4267	1.47346569	1.909857399	0.284306	0.0036	0.0054
CMTM1	CKLF like MARVEL transmembrane domai	protein_coding	chr16:66566393-6	1465	0.696913026	1.086300725	0.559474	0.0036	0.0054
HSD17B11	hydroxysteroid 17-beta dehydrogenase 1	protein_coding	chr4:87336515-87	2332	1.299652789	1.713357644	0.308267	0.0037	0.0055
FKBP7	FKBP prolyl isomerase 7	protein_coding	chr2:178463664-1 3080		0.967866513	1.300954896	0.340827	0.0037	0.0056
AL392172.1	novel transcript	lncRNA	chr1:222815022-2	1227	0.814995298	1.222609001	0.498981	0.0038	0.0058
AC093227.1	novel transcript	lncRNA	chr19:37728586-3	1360	1.432381401	2.000640454	0.390932	0.0038	0.0058
PDF	peptide deformylase, mitochondrial	protein_coding	chr16:69326913-6	2859	0.973481962	1.43747153	0.461721	0.0039	0.0058
AC027796.1	novel transcript, sense intronic to readth	lncRNA	chr17:3601761-36	512	0.685260602	1.156948969	0.641308	0.0039	0.0059
AGER	advanced glycosylation end-product spec	protein_coding	chr6:32180968-32	2075	0.867719849	1.256715561	0.450145	0.004	0.006
TTLL11	tubulin tyrosine ligase like 11	protein_coding	chr9:121821928-1	3535	1.193151304	1.588086815	0.318062	0.0042	0.0063
AC127024.2	novel transcript	lncRNA	chr17:30729469-3	894	0.604679288	0.989082941	0.58755	0.0043	0.0065
FBXL19-AS1	FBXL19 antisense RNA 1	lncRNA	chr16:30919319-3	3951	0.868090358	1.231261787	0.41206	0.0044	0.0065
AC073389.1	novel transcript, antisense to USP54	lncRNA	chr10:73625996-7	795	0.766147207	1.322931396	0.721993	0.0044	0.0066
RNF122	ring finger protein 122	protein_coding	chr8:33547754-33	1872	2.726245531	3.602623724	0.312571	0.0044	0.0066
COX16	cytochrome c oxidase assembly factor CO	protein_coding	chr14:70325081-7	1917	0.876541038	1.188154788	0.357804	0.0045	0.0067
ARL4D	ADP ribosylation factor like GTPase 4D	protein_coding	chr17:43398993-4	1578	1.76130803	2.313355185	0.308443	0.0046	0.0068
AC012629.2	novel transcript, antisense to DAP	lncRNA	chr5:10761065-10	377	0.560908885	1.030218437	0.763088	0.0046	0.0069
AP002748.3	novel transcript	lncRNA	chr11:66473490-6	2057	0.796996456	1.109517191	0.382556	0.0048	0.0071
AL096870.10	novel transcript, antisense to RABGGTAan	lncRNA	chr14:24271213-2	331	8.892754622	12.05535363	0.355012	0.0048	0.0071
AC036176.1	novel transcript, antisense to KDSR	lncRNA	chr18:63367328-6	785	1.315347755	2.038326807	0.548196	0.0048	0.0072
SEPT5	septin 5	protein_coding	chr22:19714503-1	4564	1.265256064	1.645650003	0.295345	0.005	0.0074
AC004918.4	novel transcript	lncRNA	chr7:141662922-1	925	0.732252748	1.233594599	0.655872	0.005	0.0075
DANT2	DXZ4 associated non-coding transcript 2,	lncRNA	chrX:115694880-1	4381	0.655852483	0.89560317	0.374397	0.0053	0.0078
SSC4D	scavenger receptor cysteine rich family m	protein_coding	chr7:76389334-76	2821	0.807922178	1.089974237	0.352513	0.0055	0.0081
RXRG	retinoid X receptor gamma	protein_coding	chr1:165400922-1	2691	1.669019541	2.221614651	0.332845	0.0056	0.0082
AC007038.2	novel transcript, antisense to KANSL1L	lncRNA	chr2:210028417-2	740	1.306203173	1.870393424	0.422318	0.0057	0.0084
EIF5AL1	eukaryotic translation initiation factor 5A	protein_coding	chr10:79512601-7	3840	0.789420838	1.041972575	0.316239	0.0058	0.0085
AC008870.2	novel transcript	lncRNA	chr16:23568673-2	1024	0.573745812	0.929340489	0.62025	0.0061	0.0089
ZNF486	zinc finger protein 486	protein_coding	chr19:20167214-2	4273	0.874631821	1.128473413	0.291953	0.0061	0.0089
ZNF346-IT1	ZNF346 intronic transcript 1	lncRNA	chr5:177051714-1	745	0.508819843	0.926975173	0.719865	0.0062	0.009
AL451064.1	novel transcript	lncRNA	chr6:79561132-79	471	0.466383391	0.898451008	0.869643	0.0064	0.0093
YTHDF3-AS1	YTHDF3 antisense RNA 1 (head to head)	lncRNA	chr8:63167725-63	718	0.564030883	0.965318549	0.656457	0.0064	0.0093
AL049795.1	novel transcript	lncRNA	chr1:32204769-32	1357	1.128540742	1.721001626	0.541731	0.0065	0.0095
C6orf163	chromosome 6 open reading frame 163	protein_coding	chr6:87344813-87	2920	1.239410263	1.660877395	0.336648	0.0067	0.0097
AC131009.3	novel transcript, sense intronic to PUS1	lncRNA	chr12:131934642-	287	1.3988073	2.26536921	0.636693	0.007	0.0102
AL161729.1	novel transcript, antisense to PTCH1	lncRNA	chr9:95506235-95	1402	1.634163121	2.258102099	0.390687	0.0071	0.0103
AL158151.4	novel transcript	lncRNA	chr9:129170434-1	507	0.604011174	1.195409891	1.002168	0.0072	0.0104
AC116312.1	novel transcript, antisense to SH3TC2	lncRNA	chr5:148970340-1	314	0.25212563	0.78326467	1.445121	0.0073	0.0105
AL662791.1	HLA complex group 16	lncRNA	chr6:28986800-28	317	0.746125398	1.602424875	1.104013	0.0075	0.0108
AL359380.1	novel transcript, sense intronic to MLIP	lncRNA	chr6:54190206-54	360	2.096643657	3.15794737	0.505111	0.0075	0.0108
AL021807.1	novel transcript	lncRNA	chr6:27122657-27	565	0.417513691	0.820600067	0.821923	0.0077	0.011
AC009283.1	novel transcript	lncRNA	chr17:39566915-3	645	2.444949849	3.637462331	0.471821	0.0082	0.0117
AC005034.5	novel transcript, sense intronic to MRPL1	lncRNA	chr2:75669989-75	466	3.373524327	4.59089095	0.371816	0.0083	0.0119
AC078909.2	novel transcript	lncRNA	chr15:37109587-3	398	0.483393906	0.983817672	0.928189	0.0084	0.012
AL122035.1	novel transcript	lncRNA	chr14:64422935-6	550	2.789440897	3.735663359	0.33832	0.0086	0.0123
AC005076.1	novel transcript, antisense to DMTF1	lncRNA	chr7:87151422-87	908	0.67070137	0.983792043	0.461509	0.0086	0.0123
C1orf54	chromosome 1 open reading frame 54	protein_coding	chr1:150268200-1	1270	0.808982893	1.159944054	0.42941	0.009	0.0128
AL022322.1	novel transcript, antisense to BAIAP2L2	lncRNA	chr22:38090127-3	1433	2.121380588	2.720566917	0.275685	0.0091	0.0129
AC069503.1	novel transcript	lncRNA	chr12:122007434-	1143	1.429933165	1.996339945	0.391912	0.0091	0.013
AC005332.5	novel transcript	lncRNA	chr17:68133201-6	2735	1.221170641	1.607987512	0.312849	0.0094	0.0134
SNHG30	small nucleolar RNA host gene 30	lncRNA	chr17:35568076-3	1594	1.801601259	2.334231507	0.275438	0.0096	0.0136
AL512422.1	novel transcript	lncRNA	chr6:56843928-56	1100	1.242530258	1.752893143	0.451647	0.0098	0.0139
AC024575.1	novel transcript, antisense to SWSAP1	lncRNA	chr19:11368123-1	682	0.860270486	1.308811492	0.536293	0.0098	0.0139
AC245140.2	novel transcript, antisense to RPL10	lncRNA	chrX:154396878-1	1939	1.033001762	1.384451172	0.338081	0.0099	0.0141
ZSCAN16	zinc finger and SCAN domain containing 1	protein_coding	chr6:28124609-28	1279	2.153138291	2.939237934	0.372172	0.01	0.0141
HILPDA	hypoxia inducible lipid droplet associatec	protein_coding	chr7:128455849-1 2070		1.662048303	2.175088842	0.300193	0.0105	0.0148
AC068790.3	novel transcript, sense intronic to CCDC9	lncRNA	chr12:123966077-	553	0.79719111	1.415740404	0.748102	0.0107	0.0151
AC004918.1	novel transcript, antisense to AGK	lncRNA	chr7:141652381-1	770	0.702370261	1.073862383	0.528405	0.0108	0.0152
AL138955.1	novel transcript, sense intronic to DNAJC	lncRNA	chr13:95744726-9 1040		0.481328627	0.858692122	0.761531	0.0109	0.0153
AC244090.1	novel transcript	lncRNA	chrX:154424380-1	598	6.93091731	9.001458407	0.30507	0.0115	0.0162
AC010894.2	novel transcript	lncRNA	chr2:174487380-1	800	1.678718585	2.31433657	0.36903	0.0118	0.0164
AC024560.4	novel transcript	lncRNA	chr3:197649303-1	1454	1.032039996	1.439874603	0.400831	0.0118	0.0165
AL691432.4	novel transcript	lncRNA	chr1:1600314-160	1830	1.352669374	1.734556846	0.269767	0.0119	0.0167
AC006001.2	novel transcript	lncRNA	chr7:66654538-66	2678	1.435383656	1.882616441	0.309236	0.0122	0.017

FSCN2	fascin actin-bundling protein 2, retinal	protein_coding	chr17:81528396-8	2019	1.138826639	1.505755108	0.341965	0.0123	0.0171
AC034236.2	novel transcript, antisense to COMMD10	lncRNA	chr5:116083807-1	1610	0.893449245	1.238901974	0.378504	0.0126	0.0175
AC003102.1	novel transcript, antisense to UBTF	lncRNA	chr17:44221401-4	2012	1.607596791	2.093405786	0.305644	0.013	0.0181
AC139100.2	novel transcript	lncRNA	chr18:80161752-8	662	0.640286366	1.086122346	0.62495	0.0131	0.0182
SLC25A5-AS1	SLC25A5 antisense RNA 1	lncRNA	chrX:119465986-1	3143	0.796909503	1.062022761	0.372068	0.0133	0.0185
AC026801.2	novel transcript, antisense to TTC23L	lncRNA	chr5:34837549-34	1565	0.64274359	0.948500211	0.470969	0.0133	0.0185
LINC02019	long intergenic non-protein coding RNA 2	lncRNA	chr3:50669989-50 2060		0.714288059	0.992331431	0.395192	0.0137	0.019
SLC9A3R1	SLC9A3 regulator 1	protein_coding	chr17:74748628-7	2339	0.81126113	1.069208616	0.308593	0.014	0.0194
AC015922.3	novel transcript	lncRNA	chr17:15789016-1	690	2.418108448	3.361571976	0.38876	0.0143	0.0198
CCR10	C-C motif chemokine receptor 10	protein_coding	chr17:42678889-4	2619	0.822937697	1.069389014	0.299075	0.0144	0.0198
TRAPPC2B	trafficking protein particle complex 2B	protein_coding	chr19:57363511-5	1843	0.876043114	1.176246069	0.332941	0.0145	0.0201
NALT1	NOTCH1 associated lncRNA in T cell acute	lncRNA	chr9:136546212-1	1088	2.644492027	3.515321049	0.313624	0.0146	0.0201
PAN3-AS1	PAN3 antisense RNA 1	lncRNA	chr13:28136843-2	1351	1.373733151	1.798303469	0.313805	0.0146	0.0201
AP001816.1	novel transcript	lncRNA	chr4:101347780-1	790	1.170836594	1.737353773	0.470576	0.0151	0.0208
AC138207.2	novel transcript, antisense to NF1	lncRNA	chr17:31090787-3	492	1.658863761	2.522395891	0.499795	0.0155	0.0214
AC093627.2	novel transcript	lncRNA	chr7:174920-1760	568	0.525455991	0.88506159	0.678324	0.0157	0.0216
AP000926.2	novel transcript, antisense to HSPA8and C	lncRNA	chr11:123062655-	724	0.597968601	0.940402865	0.629134	0.0158	0.0217
AC139149.1	novel transcript, antisense to ACTG1	lncRNA	chr17:81514047-8	957	1.160928898	1.71696563	0.466	0.0158	0.0217
DUSP13	dual specificity phosphatase 13	protein_coding	chr10:75094432-7	2351	0.989530502	1.276417273	0.279634	0.0159	0.0218
AP001453.1	novel transcript	lncRNA	chr11:64229214-6	1051	0.750518698	1.1647023	0.580972	0.0159	0.0218
AC083799.1	novel transcript, sense intronic to TMCC1	lncRNA	chr3:129847048-1	910	1.863158545	2.432212348	0.298457	0.0162	0.0222
AC080023.1	novel transcript	lncRNA	chr11:10302657-1	803	1.039487852	1.563845891	0.537304	0.0163	0.0223
AL356489.2	novel transcript	lncRNA	chr9:33697459-33	3528	1.421308867	1.844540711	0.291426	0.0167	0.0229
AC005759.2	novel transcript, antisense to PDE4C	lncRNA	chr19:18204730-1	1212	0.686474962	1.072386441	0.530305	0.018	0.0244
AL356512.1	novel transcript, antisense to EFCAB2	lncRNA	chr1:244969350-2	1739	0.966374881	1.306134358	0.336298	0.0182	0.0247
AC138150.1	novel transcript, antisense to HEXIM1 and	lncRNA	chr17:45150400-4	696	1.950578175	2.673938829	0.357436	0.0184	0.025
C1orf229	chromosome 1 open reading frame 229	lncRNA	chr1:247110160-2	2258	0.923243315	1.201030921	0.28748	0.019	0.0257
AC010809.1	novel transcript, antisense to RYR3	lncRNA	chr15:33858602-3	562	0.830714555	1.233554997	0.487441	0.0201	0.0271
AC068279.2	novel transcript	lncRNA	chr2:87311460-87	1417	1.03641179	1.385738317	0.325533	0.0202	0.0273
SLC35D2	solute carrier family 35 member D2	protein_coding	chr9:96313444-96	3827	1.325715219	1.729417937	0.279887	0.0206	0.0277
AL117336.2	novel transcript, antisense to CCNY	lncRNA	chr10:35314552-3	1426	1.986420775	2.540109193	0.270396	0.0208	0.0281
AC026304.1	novel transcript	lncRNA	chr3:143000907-1	561	1.081867363	1.633643476	0.533676	0.0211	0.0284
RAB20	RAB20, member RAS oncogene family	protein_coding	chr13:110523066-	1507	1.881598154	2.434781987	0.28267	0.0213	0.0286
FO393418.1	novel transcript	lncRNA	chr22:36388626-3	510	1.355707953	2.107237222	0.588198	0.0219	0.0294
AL133520.1	novel transcript, sense intronic to SLC13A	lncRNA	chr20:46681676-4	700	0.629938415	0.93873053	0.500851	0.022	0.0295
AC012442.2	novel transcript	lncRNA	chr2:112589040-1	425	0.599138077	0.991225939	0.581792	0.022	0.0295
HIST1H4K	histone cluster 1 H4 family member k	protein_coding	chr6:27831216-27	312	0.670963645	1.136292914	0.802412	0.0238	0.0318
AC145207.1	novel transcript	lncRNA	chr17:81941869-8	691	1.298639499	1.878518149	0.44675	0.0242	0.0323
AP000894.4	novel transcript	lncRNA	chr18:813274-813	483	0.759165091	1.249337682	0.593253	0.0246	0.0328
AC011481.1	novel transcript	lncRNA	chr19:44950044-4	361	0.980607779	1.72079137	0.785699	0.0249	0.0332
AL442125.2	novel transcript, sense intronic	lncRNA	chr13:113511747-	418	0.709348474	1.191004849	0.679362	0.0252	0.0336
AL355987.4	novel transcript	lncRNA	chr9:136799223-1	4335	0.859779187	1.124085001	0.31098	0.0257	0.0342
AP000255.1	novel transcript	lncRNA	chr21:31735732-3	676	0.368346092	0.688895767	0.750558	0.0262	0.0348
AC008393.1	novel transcript	lncRNA	chr5:179859013-1	1454	1.206208495	1.583935379	0.314537	0.0264	0.035
HPDL	4-hydroxyphenylpyruvate dioxygenase lik	protein_coding	chr1:45326905-45	1629	0.96720733	1.3226979	0.398116	0.0264	0.035
AC010531.6	novel transcript, antisense to FBXO31 and	lncRNA	chr16:87317509-8	401	0.808343937	1.309761313	0.614887	0.0267	0.0353
PCF11-AS1	PCF11 antisense RNA 1	lncRNA	chr11:83185521-8	1516	1.345011084	1.771935994	0.319555	0.0281	0.0371
AC138932.5	novel transcript, antisense to PDXDC1	lncRNA	chr16:15015828-1	563	0.781102935	1.185095219	0.486448	0.0282	0.0372
LINC01311	long intergenic non-protein coding RNA 1	lncRNA	chr22:19171395-1	1445	0.813162476	1.083414874	0.316754	0.0289	0.0381
AL627309.2	novel transcript	lncRNA	chr1:139790-1403	323	0.496185805	0.925311393	1.000288	0.0291	0.0383
LINC02315	long intergenic non-protein coding RNA 2	lncRNA	chr14:40954693-4	1136	1.508702981	1.972294433	0.284097	0.0291	0.0383
ATP2A1-AS1	ATP2A1 antisense RNA 1	lncRNA	chr16:28878957-2	583	1.969962604	2.76499046	0.403377	0.0291	0.0384
PRRT3-AS1	PRRT3 antisense RNA 1	lncRNA	chr3:9947404-995	614	0.586534722	0.981749471	0.824548	0.0298	0.0392
AC004908.2	novel transcript, sense intronic to ZNF59(	lncRNA	chr8:233119-2336	574	1.602787923	2.170427639	0.375963	0.0305	0.0401
AC010809.2	novel transcript, antisense to RYR3	lncRNA	chr15:33851785-3	964	0.591195894	0.867092718	0.465914	0.0306	0.0402
AC005391.1	novel transcript, sense intronic to ARID3A	lncRNA	chr19:956485-958	341	0.632367354	1.028359199	0.673045	0.0307	0.0403
SLC25A41	solute carrier family 25 member 41	protein_coding	chr19:6426037-64	1649	0.835774613	1.083327567	0.282189	0.0329	0.043
AL355999.1	novel transcript, antisense to DHX9	lncRNA	chr1:182837185-1	1000	0.807911219	1.112803284	0.363169	0.0335	0.0437
AC106820.5	novel transcript	lncRNA	chr16:2456252-24	879	0.517264801	0.86095786	0.559118	0.0349	0.0454
HK2	hexokinase 2	protein_coding	chr2:74833981-74	6064	9.887346244	12.67056069	0.265582	0.0349	0.0455
AC008610.1	novel transcript	lncRNA	chr5:180293245-1	393	1.503191936	2.178338486	0.450546	0.0351	0.0457
AP005205.2	novel transcript, antisense to ARHGAP28.	lncRNA	chr18:6728821-67	491	0.452378422	0.802679655	0.97301	0.0356	0.0464
LINC01004	long intergenic non-protein coding RNA 1	lncRNA	chr7:104950315-1	1483	1.278979541	1.680630235	0.319007	0.0357	0.0465
AC092574.1	novel transcript	lncRNA	chr4:416118-4165	420	0.684099625	1.077840304	0.583156	0.0376	0.0487
LINC01909	long intergenic non-protein coding RNA 1	lncRNA	chr18:70335439-7 1040		0.717788294	1.004091124	0.407324	0.0376	0.0488
BST2	bone marrow stromal cell antigen 2	protein_coding	chr19:17402939-1	1101	0.944388732	1.274188712	0.348676	0.0377	0.0488
LINC00653	long intergenic non-protein coding RNA 6	lncRNA	chr20:18794529-1	1539	0.884557791	1.146958319	0.29859	0.0381	0.0493
AF131215.7	novel transcript	lncRNA	chr8:11202965-11	707	0.653686577	1.006689071	0.576936	0.0381	0.0493

Supplemental Table 2: Underexpressed genes in hsa-miR-548v transfected cells compared to miR negative transfected cells

Gene_Name	Description	Gene_biotype	Gene_position	CumulativeLengthExon	FPKM.miR negative control	FPKM.Hsa-mir-548v	log2FC	pval	qval
NPPB	natriuretic peptide B	protein_coding	chr1:11857464-	708	824.817662	53.69000697	-4.025	4.04E-21	4.85E-17
GNS	glucosamine (N-acetyl)-6-sulfatase	protein_coding	chr12:64713445	6268	31.90711848	4.865041018	-2.799	2.020176	1.21E-16
ANKRD1	ankyrin repeat domain 1	protein_coding	chr10:90912096	1979	1092.822125	171.1878754	-2.76	3.955689	1.58E-16
ABAT	4-aminobutyrate aminotransferase	protein_coding	chr16:8674596-	9713	19.8976529	3.358567053	-2.652	3.68E-19	1.10E-15
CCND2	cyclin D2	protein_coding	chr12:4273762-	7170	27.37262401	7.556994709	-1.943	4.64E-19	1.11E-15
ACADM	acyl-CoA dehydrogenase medium chain	protein_coding	chr1:75724347-	4783	42.14770003	9.263375056	-2.27	1.55E-18	2.06E-15
LBH	limb bud and heart development	protein_coding	chr2:30231534-	4532	66.51243693	15.90366076	-2.149	1.10E-18	2.06E-15
SERINC3	serine incorporator 3	protein_coding	chr20:44496221	4649	24.89203048	7.521386574	-1.813	1.39E-18	2.06E-15
WSB2	WD repeat and SOCS box containing 2	protein_coding	chr12:11803269	4616	16.51512229	2.330223617	-2.908	1.29E-18	2.06E-15
CXCL12	C-X-C motif chemokine ligand 12	protein_coding	chr10:44370165	6077	15.422372	1.733606225	-3.239	1.83E-18	2.20E-15
PITPNA	phosphatidylinositol transfer protein alpl	protein_coding	chr17:1517718-	4784	37.5361439	11.88762919	-1.745	2.22E-18	2.22E-15
YWHAG	tyrosine 3-monooxygenase/tryptophan 5-	protein_coding	chr7:76326799-	3705	98.97793606	30.39387471	-1.789	2.61E-18	2.41E-15
NNT	nicotinamide nucleotide transhydrogena	protein_coding	chr5:43602692-	12018	43.21462821	14.64319377	-1.647	3.86E-18	3.08E-15
AP002956.1	melanoma cell adhesion molecule	protein_coding	chr11:11930852	7422	27.09858953	6.767641273	-2.086	4.40E-18	3.29E-15
EFCAB14	EF-hand calcium binding domain 14	protein_coding	chr1:46675159-	6335	15.37787601	2.675579655	-2.606	4.78E-18	3.37E-15
LRRC10	leucine rich repeat containing 10	protein_coding	chr12:69608564	2599	172.2062682	55.29795828	-1.725	7.72E-18	4.03E-15
PSAP	prosaposin	protein_coding	chr10:71816298	3189	307.5904264	110.7400764	-1.56	7.43E-18	4.03E-15
RCSD1	RCSD domain containing 1	protein_coding	chr1:167630093	8165	29.14247646	10.49710863	-1.559	7.72E-18	4.03E-15
TIMP3	TIMP metallopeptidase inhibitor 3	protein_coding	chr22:32801701	4603	83.94855838	24.86182514	-1.841	7.11E-18	4.03E-15
TP53INP2	tumor protein p53 inducible nuclear prot	protein_coding	chr20:34704339	4179	104.4135171	28.80803023	-1.944	7.28E-18	4.03E-15
WLS	Wnt ligand secretion mediator	protein_coding	chr1:68098473-	4459	36.68002444	12.34984141	-1.657	7.59E-18	4.03E-15
SLC35A4	solute carrier family 35 member A4	protein_coding	chr5:140564828	3648	28.89881867	9.973738039	-1.621	1.17E-17	5.84E-15
PRPS1	phosphoribosyl pyrophosphate synthetas	protein_coding	chrX:107628424	2623	13.33139868	2.271971815	-2.637	1.30E-17	6.15E-15
TSC22D3	TSC22 domain family member 3	protein_coding	chrX:107713221	4766	9.986569188	2.569765025	-2.046	1.33E-17	6.15E-15
SFXN1	sideroflexin 1	protein_coding	chr5:175477062	7407	18.56364193	5.927643713	-1.732	1.40E-17	6.22E-15
DCBLD2	discoidin, CUB and LCCL domain containi	protein_coding	chr3:98795941-	11280	34.05630144	13.4383268	-1.428	1.53E-17	6.56E-15
KPNA2	karyopherin subunit alpha 2	protein_coding	chr17:68035708	2963	50.26092008	8.384922718	-2.669	1.71E-17	7.05E-15
APLP2	amyloid beta precursor like protein 2	protein_coding	chr11:13006814	7463	97.33144208	38.62099915	-1.42	1.84E-17	7.35E-15
ECPAS	Ecm29 proteasome adaptor and scaffold	protein_coding	chr9:111360692	8449	19.75657818	6.514185913	-1.686	2.54E-17	9.40E-15
EIF2S3	eukaryotic translation initiation factor 2 s	protein_coding	chrX:24054946-	4081	60.41456922	22.28418101	-1.525	2.59E-17	9.40E-15
SYNJ2BP	synaptojanin 2 binding protein	protein_coding	chr14:70366499	7057	12.22137363	4.297157971	-1.593	2.56E-17	9.40E-15
RAI14	retinoic acid induced 14	protein_coding	chr5:34656328-	7581	18.94873655	6.572862341	-1.614	3.03E-17	1.07E-14
HEG1	heart development protein with EGF like (	protein_coding	chr3:124965710	9964	25.59280187	4.605499237	-2.557	3.60E-17	1.23E-14
BICD2	BICD cargo adaptor 2	protein_coding	chr9:92711363-	6448	13.89097721	4.361060044	-1.759	4.13E-17	1.38E-14
TMEM71	transmembrane protein 71	protein_coding	chr8:132685007	3332	22.46946149	5.958168842	-2.003	4.42E-17	1.43E-14
DGKI	diacylglycerol kinase iota	protein_coding	chr7:137381037	15549	14.01641137	3.56069472	-2.062	5.07E-17	1.56E-14
SSPN	sarcospan	protein_coding	chr12:26121991	6821	9.60331058	2.893621123	-1.817	5.82E-17	1.70E-14
ANP32B	acidic nuclear phosphoprotein 32 family r	protein_coding	chr9:97983341-	1870	40.5387719	4.901182454	-3.13	7.38E-17	1.86E-14
CCT5	chaperonin containing TCP1 subunit 5	protein_coding	chr5:10249929-	5638	30.16760393	10.42220542	-1.618	7.37E-17	1.86E-14
CLINT1	clathrin interactor 1	protein_coding	chr5:157785743	6368	13.45998738	5.040630561	-1.504	7.30E-17	1.86E-14
HDGF	heparin binding growth factor	protein_coding	chr1:156742109	3674	63.3988578	19.14235345	-1.812	7.46E-17	1.86E-14
NOLC1	nucleolar and coiled-body phosphoprotei	protein_coding	chr10:10215217	3970	23.1473474	6.393211436	-1.942	7.20E-17	1.86E-14
WASF2	WASP family member 2	protein_coding	chr1:27404230-	5681	23.54625761	7.41802977	-1.751	6.74E-17	1.86E-14
SPARC	secreted protein acidic and cysteine rich	protein_coding	chr5:151661096	4641	26.23947249	9.523579698	-1.55	7.73E-17	1.89E-14
RAB18	RAB18, member RAS oncogene family	protein_coding	chr10:27504174	6223	16.93167148	5.801499552	-1.63	8.01E-17	1.92E-14
HYOU1	hypoxia up-regulated 1	protein_coding	chr11:11904418	7723	14.78762007	5.499016054	-1.513	8.39E-17	1.97E-14
C1orf198	chromosome 1 open reading frame 198	protein_coding	chr1:230837119	5161	27.94122572	9.791972068	-1.598	9.34E-17	2.11E-14
STAT3	signal transducer and activator of transcri	protein_coding	chr17:42313324	8812	32.74202846	11.82585902	-1.556	9.30E-17	2.11E-14
SDC2	syndecan 2	protein_coding	chr8:96493813-	4084	11.03247247	3.612988499	-1.697	1.12E-16	2.50E-14
MASP1	mannan binding lectin serine peptidase 1	protein_coding	chr3:187217285	13161	28.91785173	10.80813233	-1.507	1.23E-16	2.58E-14
RMND5A	required for meiotic nuclear division 5 ho	protein_coding	chr2:86720291-	6686	28.83750439	8.326652905	-1.877	1.23E-16	2.58E-14
PAIP2	poly(A) binding protein interacting protei	protein_coding	chr5:139341587	3158	37.78569143	10.88241443	-1.881	1.27E-16	2.62E-14
PKIA	cAMP-dependent protein kinase inhibitor	protein_coding	chr8:78516340-	4015	23.53276572	8.969134617	-1.477	1.35E-16	2.73E-14
RTN3	reticulon 3	protein_coding	chr11:63681446	5792	24.38222744	10.34541767	-1.322	1.37E-16	2.74E-14
EPN3	epsin 3	protein_coding	chr17:50532682	4503	10.57850251	1.319900468	-3.084	1.40E-16	2.75E-14
SAR1A	secretion associated Ras related GTPase 1/	protein_coding	chr10:70147289	6914	16.06755409	5.800998952	-1.558	1.56E-16	2.95E-14
DYSF	dysferlin	protein_coding	chr2:71453722-	8389	16.89881691	6.367392311	-1.493	1.69E-16	3.09E-14
TNIP1	TNFAIP3 interacting protein 1	protein_coding	chr5:151029945	5793	18.08507068	4.644569571	-2.048	1.70E-16	3.09E-14
ZNF385A	zinc finger protein 385A	protein_coding	chr12:54369133	3179	9.116630413	1.736605625	-2.474	2.00E-16	3.57E-14
OSTM1	osteoclastogenesis associated transmemb	protein_coding	chr6:108041409	5325	12.29901299	5.022176088	-1.378	2.11E-16	3.73E-14
SLC26A2	solute carrier family 26 member 2	protein_coding	chr5:149960737	8606	4.350195712	1.018362603	-2.178	2.17E-16	3.77E-14
ISCA1	iron-sulfur cluster assembly 1	protein_coding	chr9:86264546-	3251	19.25941908	6.304695824	-1.696	2.26E-16	3.87E-14
TMEM64	transmembrane protein 64	protein_coding	chr8:90621995-	5870	11.45603132	3.280524427	-1.892	2.51E-16	4.18E-14
RB1	RB transcriptional corepressor 1	protein_coding	chr13:48303726	6452	12.67228344	5.606263443	-1.263	2.61E-16	4.29E-14
CYB5R1	cytochrome b5 reductase 1	protein_coding	chr1:202961873	4495	28.45073265	13.56823186	-1.155	2.75E-16	4.40E-14
NRP1	neuropilin 1	protein_coding	chr10:33177492	11522	12.38252284	4.290625889	-1.615	2.96E-16	4.67E-14
STRIP2	striatin interacting protein 2	protein_coding	chr7:129434432	5877	41.82980574	18.90450288	-1.232	3.04E-16	4.72E-14
SCD	stearoyl-CoA desaturase	protein_coding	chr10:10034723	5245	25.184809	5.694930425	-2.228	3.25E-16	4.93E-14
NDUFB3	NADH:ubiquinone oxidoreductase subuni	protein_coding	chr2:201071433	841	82.45765761	17.29098619	-2.335	3.35E-16	5.01E-14
PRUNE1	prune exopolyphosphatase 1	protein_coding	chr1:151008420	3156	14.37952557	4.449449017	-1.778	3.57E-16	5.29E-14
ANAPC16	anaphase promoting complex subunit 16	protein_coding	chr10:72216000	4324	33.66656159	15.2244621	-1.232	3.64E-16	5.32E-14
CDKN1B	cyclin dependent kinase inhibitor 1B	protein_coding	chr12:12715058	2565	31.48598392	12.62003332	-1.405	4.02E-16	5.74E-14
ZNF629	zinc finger protein 629	protein_coding	chr16:30778456	6083	18.28268771	7.654995138	-1.341	4.45E-16	6.28E-14
HNRNPA3	heterogeneous nuclear ribonucleoprotein	protein_coding	chr2:177212563	6033	43.21642957	17.60222824	-1.381	4.61E-16	6.43E-14
EXTL1	exostosin like glycosyltransferase 1	protein_coding	chr1:26019884-	4537	22.18177224	5.202174043	-2.176	4.78E-16	6.59E-14
SGPL1	sphingosine-1-phosphate lyase 1	protein_coding	chr10:70815961	7486	8.721111408	3.37073112	-1.457	5.04E-16	6.79E-14
CAP1	cyclase associated actin cytoskeleton regu	protein_coding	chr1:40040233-	3814	19.74396786	7.996507704	-1.392	5.25E-16	7.00E-14
HNRNPA0	heterogeneous nuclear ribonucleoprotein	protein_coding	chr5:137745651	8726	16.73651365	5.639367975	-1.654	5.34E-16	7.03E-14
FBXO32	F-box protein 32	protein_coding	chr8:123497889	7395	22.80392473	6.499364894	-1.894	5.43E-16	7.07E-14
CHRM2	cholinergic receptor muscarinic 2	protein_coding	chr7:136868669	8322	24.83747726	9.824281309	-1.424	5.91E-16	7.46E-14
DACT1	dishevelled binding antagonist of beta cat	protein_coding	chr14:58633967	4490	7.710732028	1.723772507	-2.244	5.93E-16	7.46E-14
NAPG	NSF attachment protein gamma	protein_coding	chr18:10525905	5589	7.099620008	2.252581372	-1.742	5.98E-16	7.46E-14
THBS1	thrombospondin 1	protein_coding	chr15:39581079	9158	6.117852562	2.068258362	-1.653	6.10E-16	7.46E-14
ADIPOR1	adiponectin receptor 1	protein_coding	chr1:202940826	3113	42.76882191	17.98009069	-1.337	6.63E-16	7.94E-14
LGI2	leucine rich repeat LGI family member 2	protein_coding	chr4:24998847-	6495	3.819457725	0.717899701	-2.491	6.61E-16	7.94E-14
KRT18	keratin 18	protein_coding	chr12:52948871	3069	110.2271236	48.80915516	-1.261	6.73E-16	7.97E-14
SOD2	superoxide dismutase 2	protein_coding	chr6:159669069	16431	27.03949754	13.12002071	-1.13	6.78E-16	7.97E-14
AL159166.1	novel transcript	lncRNA	chr6:4341756-4	3725	12.22939033	3.296540919	-1.973	6.87E-16	7.99E-14
ALDOC	aldolase, fructose-bisphosphate C	protein_coding	chr17:28573115	2612	71.8300254	11.84631581	-2.69	7.24E-16	8.34E-14
GBE1	1,4-alpha-glucan branching enzyme 1	protein_coding	chr3:81489703-	4651	32.26422541	15.92434226	-1.105	8.34E-16	9.25E-14
VAPB	VAMP associated protein B and C	protein_coding	chr20:58389122	10585	7.0932319	2.750447422	-1.451	8.76E-16	9.63E-14
SCUBE3	signal peptide, CUB domain and EGF like c	protein_coding	chr6:35213956-	7819	14.2869551	3.268513027	-2.21	8.92E-16	9.72E-14
IGSF3	immunoglobulin superfamily member 3	protein_coding	chr1:116574399	7400	17.72504775	5.846640824	-1.684	9.18E-16	9.82E-14
TMEM59	transmembrane protein 59	protein_coding	chr1:54026681-	9149	11.01488182	4.84713551	-1.27	9.38E-16	9.96E-14
NDUFB5	NADH:ubiquinone oxidoreductase subuni	protein_coding	chr3:179604690	5871	32.85319623	9.474695271	-1.876	1.06E-15	1.11E-13
PLCL2	phospholipase C like 2	protein_coding	chr3:16802651-	5160	11.97237598	5.138492882	-1.307	1.07E-15	1.11E-13
EIF4EBP2	eukaryotic translation initiation factor 4E	protein_coding	chr10:70404145	7491	38.18625574	16.70643626	-1.278	1.14E-15	1.17E-13
SMC1A	structural maintenance of chromosomes	protein_coding	chrX:53374149-	10484	11.69244433	4.658478239	-1.412	1.21E-15	1.23E-13
TMCO1	transmembrane and coiled-coil domains :	protein_coding	chr1:165724293	5584	7.821763058	2.66562994	-1.638	1.21E-15	1.23E-13
ARL6IP1	ADP ribosylation factor like GTPase 6 inte	protein_coding	chr16:18791669	3558	10.33567272	2.761387939	-1.991	1.25E-15	1.25E-13
SREBF2	sterol regulatory element binding transcri	protein_coding	chr22:41833079	7556	76.96532474	36.73942172	-1.153	1.26E-15	1.25E-13
PDPN	podoplanin	protein_coding	chr1:13583465-	3707	23.83361152	9.632272276	-1.393	1.30E-15	1.27E-13
C6orf89	chromosome 6 open reading frame 89	protein_coding	chr6:36871870-	7340	16.74440919	6.309267913	-1.493	1.37E-15	1.33E-13
GATA4	GATA binding protein 4	protein_coding	chr8:11676959-	4443	87.96287377	43.10405513	-1.115	1.62E-15	1.53E-13

MRPS7	mitochondrial ribosomal protein S7	protein_coding	chr17:75261674	3327	26.38718233	8.467916697	-1.727	1.65E-15	1.55E-13
ARHGEF6	Rac/Cdc42 guanine nucleotide exchange f	protein_coding	chrX:136665547	5278	8.501261383	2.03071126	-2.156	1.68E-15	1.55E-13
CBX5	chromobox 5	protein_coding	chr12:54230942	15038	14.92532747	6.030022222	-1.393	1.68E-15	1.55E-13
FTO	FTO alpha-ketoglutarate dependent dioxy	protein_coding	chr16:53701692	21379	2.687541207	1.274658378	-1.162	1.78E-15	1.62E-13
RFX5	regulatory factor X5	protein_coding	chr1:15134064C	4586	11.55108849	5.974390983	-1.186	1.79E-15	1.62E-13
RBMS1	RNA binding motif single stranded interac	protein_coding	chr2:160272151	11778	19.12684747	8.777732014	-1.209	1.85E-15	1.66E-13
AC126773.2	novel transcript, antisense to CDH3	lncRNA	chr16:68644248	1682	8.732745947	1.406918693	-2.716	1.87E-15	1.66E-13
TP53BP2	tumor protein p53 binding protein 2	protein_coding	chr1:223779893	5979	11.55108849	4.935512889	-1.313	1.97E-15	1.72E-13
UBE2E3	ubiquitin conjugating enzyme E2 E3	protein_coding	chr2:180967248	2974	21.16037847	8.08551524	-1.472	1.96E-15	1.72E-13
CCDC117	coiled-coil domain containing 117	protein_coding	chr22:28772674	4326	13.00398666	5.408997426	-1.351	1.99E-15	1.73E-13
PFKFB2	6-phosphofructo-2-kinase/fructose-2,6-bi	protein_coding	chr1:207034366	10176	11.99759919	4.617698969	-1.462	2.03E-15	1.74E-13
ULK3	unc-51 like kinase 3	protein_coding	chr15:74836118	3514	16.32118267	6.467772795	-1.42	2.02E-15	1.74E-13
RPRD1B	regulation of nuclear pre-mRNA domain c	protein_coding	chr20:38033546	5511	10.1446496	4.671238884	-1.205	2.28E-15	1.92E-13
GOLGA1	golgin A1	protein_coding	chr9:124878357	6019	7.223223525	2.922146111	-1.391	2.33E-15	1.95E-13
CIAPIN1	cytokine induced apoptosis inhibitor 1	protein_coding	chr16:57428187	3383	24.27646146	8.067471129	-1.676	2.35E-15	1.95E-13
MTMR2	myotubularin related protein 2	protein_coding	chr11:95832882	6119	6.482673901	2.303384166	-1.578	2.52E-15	2.08E-13
CDH3	cadherin 3	protein_coding	chr16:68636189	6076	4.796328126	1.474798946	-1.787	2.59E-15	2.12E-13
ZBTB5	zinc finger and BTB domain containing 5	protein_coding	chr9:37438102-	4690	8.366643866	3.029087964	-1.554	2.71E-15	2.19E-13
SPCS2	signal peptidase complex subunit 2	protein_coding	chr11:74949247	2984	12.94416471	3.941754868	-1.799	2.76E-15	2.22E-13
ABHD2	abhydrolase domain containing 2	protein_coding	chr15:89087459	10068	6.843738439	1.877876226	-1.948	2.98E-15	2.37E-13
ADAMTSL4	ADAMTS like 4	protein_coding	chr1:150549369	6432	10.74940421	3.204687077	-1.828	2.98E-15	2.37E-13
CUL5	cullin 5	protein_coding	chr11:10800889	6562	8.984910545	4.179335591	-1.192	3.03E-15	2.39E-13
C18orf25	chromosome 18 open reading frame 25	protein_coding	chr18:46173553	9577	7.145118621	3.594069611	-1.077	3.13E-15	2.43E-13
ETF1	eukaryotic translation termination factor	protein_coding	chr5:138506095	4537	30.36747573	16.23241519	-0.99	3.15E-15	2.43E-13
UHMK1	U2AF homology motif kinase 1	protein_coding	chr1:162497251	9248	10.13499678	4.195812638	-1.357	3.11E-15	2.43E-13
CSTF2T	cleavage stimulation factor subunit 2 tau	protein_coding	chr10:51695487	4105	11.39618302	4.452662845	-1.441	3.23E-15	2.48E-13
KLHDC3	kelch domain containing 3	protein_coding	chr6:43014103-	2019	114.3078374	63.70099243	-0.93	3.40E-15	2.54E-13
XYLT2	xylosyltransferase 2	protein_coding	chr17:50346092	5472	11.38938445	5.192567802	-1.22	3.74E-15	2.77E-13
PDLIM3	PDZ and LIM domain 3	protein_coding	chr4:185500660	9117	4.771789477	2.010433964	-1.332	3.77E-15	2.77E-13
RBM18	RNA binding motif protein 18	protein_coding	chr9:122237622	5196	9.699060084	4.02035577	-1.357	3.84E-15	2.81E-13
FAM53B	family with sequence similarity 53 membe	protein_coding	chr10:12461929	6533	10.7040077	4.428420487	-1.358	3.96E-15	2.88E-13
MESD	mesoderm development LRP chaperone	protein_coding	chr15:80946289	7455	5.221552724	2.147332736	-1.368	4.03E-15	2.91E-13
KLHL3	kelch like family member 3	protein_coding	chr5:137617500	11853	3.458071886	1.228435839	-1.579	4.07E-15	2.91E-13
ARF4	ADP ribosylation factor 4	protein_coding	chr3:57571363-	2595	41.4734567	18.85876551	-1.222	4.48E-15	3.18E-13
ST5	suppression of tumorigenicity 5	protein_coding	chr11:8693351-	14262	3.230344718	1.170816268	-1.551	4.72E-15	3.33E-13
CEMIP2	cell migration inducing hyaluronidase 2	protein_coding	chr9:71683366-	9254	6.022284061	2.377233537	-1.426	4.80E-15	3.34E-13
COG2	component of oligomeric golgi complex 2	protein_coding	chr1:230642481	10946	4.27876854	1.684516289	-1.432	4.86E-15	3.37E-13
AREL1	apoptosis resistant E3 ubiquitin protein li	protein_coding	chr14:74653437	7297	9.534166026	4.184543	-1.273	4.92E-15	3.39E-13
C22orf39	chromosome 22 open reading frame 39	protein_coding	chr22:19351368	6943	7.473644893	3.765499709	-1.075	5.06E-15	3.45E-13
MAST4	microtubule associated serine/threonine	protein_coding	chr5:66596361-	14675	10.19734064	4.60186743	-1.233	5.36E-15	3.61E-13
TOMM7	translocase of outer mitochondrial memb	protein_coding	chr7:22812628-	1751	27.06897689	6.993617129	-2.044	5.49E-15	3.68E-13
TRAM1	translocation associated membrane prote	protein_coding	chr8:70573218-	4131	17.55924467	7.427647725	-1.328	5.60E-15	3.73E-13
GTF2A2	general transcription factor IIA subunit 2	protein_coding	chr15:59638062	3204	13.57653219	4.2697446	-1.757	5.78E-15	3.81E-13
XIRP2	xin actin binding repeat containing 2	protein_coding	chr2:166888487	13031	11.79540215	4.34565217	-1.525	5.78E-15	3.81E-13
CTPS2	CTP synthase 2	protein_coding	chrX:16587999-	5531	7.925144921	3.226439002	-1.384	5.86E-15	3.84E-13
SMARCA4	SWI/SNF related, matrix associated, actin	protein_coding	chr19:10960825	15778	11.25016915	6.061372686	-0.978	5.90E-15	3.85E-13
SLC5A1	solute carrier family 5 member 1	protein_coding	chr22:32043032	5310	22.87162254	8.367935864	-1.541	6.23E-15	4.01E-13
ARSB	arylsulfatase B	protein_coding	chr5:78777209-	8899	6.872225877	3.48382357	-1.066	6.61E-15	4.24E-13
PLA2G12A	phospholipase A2 group XIIA	protein_coding	chr4:109709989	5221	8.137772359	3.58430229	-1.268	6.76E-15	4.31E-13
TUB	tubby bipartite transcription factor	protein_coding	chr11:8019244-	6722	14.74394222	4.508313499	-1.792	7.03E-15	4.39E-13
ZNF106	zinc finger protein 106	protein_coding	chr15:42412823	12226	36.32302167	18.7373579	-1.041	7.08E-15	4.40E-13
SHLD2	shieldin complex subunit 2	protein_coding	chr10:87094161	4242	6.852089964	2.572966081	-1.496	7.30E-15	4.51E-13
CDK8	cyclin dependent kinase 8	protein_coding	chr13:26254104	3538	15.60297662	7.938420458	-1.061	7.41E-15	4.55E-13
BNIP3L	BCL2 interacting protein 3 like	protein_coding	chr8:26383054-	4149	17.85401408	4.389990404	-2.112	7.45E-15	4.56E-13
IST1	IST1 factor associated with ESCRT-III	protein_coding	chr16:71845996	7806	13.91177774	6.507753365	-1.181	7.57E-15	4.58E-13
NPC2	NPC intracellular cholesterol transporter	protein_coding	chr14:74476192	2749	20.43925146	3.24177089	-2.735	7.88E-15	4.73E-13
DSG2	desmoglein 2	protein_coding	chr18:31498177	6056	14.75243909	6.589663047	-1.248	8.27E-15	4.86E-13
TFG	TRK-fused gene	protein_coding	chr3:100709331	4992	31.00449758	15.37461527	-1.098	8.23E-15	4.86E-13
CDK14	cyclin dependent kinase 14	protein_coding	chr7:90466424-	7017	12.60946814	6.006718464	-1.155	8.39E-15	4.91E-13
EFNB2	ephrin B2	protein_coding	chr13:10648974	5005	3.855613936	1.304764533	-1.65	8.57E-15	4.99E-13
MIDN	midnolin	protein_coding	chr19:1248553-	4492	29.01083713	16.23394623	-0.923	8.78E-15	5.08E-13
SLC25A3	solute carrier family 25 member 3	protein_coding	chr12:98593591	8896	78.14778271	35.9386934	-1.205	9.13E-15	5.26E-13
HOPX	HOP homeobox	protein_coding	chr4:56647988-	7753	1.752459195	0.40620913	-2.188	9.32E-15	5.35E-13
CRKL	CRK like proto-oncogene, adaptor protein	protein_coding	chr22:20917407	5342	14.31933947	6.935828952	-1.131	1.02E-14	5.78E-13
ARL5A	ADP ribosylation factor like GTPase 5A	protein_coding	chr2:151788984	6721	12.67673853	6.668088454	-1.014	1.05E-14	5.89E-13
GNB1	G protein subunit beta 1	protein_coding	chr1:1785285-1	6417	38.22004659	21.44757929	-0.92	1.06E-14	5.89E-13
IMPAD1	inositol monophosphatase domain conta	protein_coding	chr8:56957931-	7619	18.65103081	10.33261584	-0.938	1.06E-14	5.89E-13
RNGTT	RNA guanylyltransferase and 5'-phosphata	protein_coding	chr6:88609897-	5188	3.950281463	1.169862238	-1.844	1.07E-14	5.89E-13
ST8SIA2	ST8 alpha-N-acetyl-neuraminide alpha-2,8	protein_coding	chr15:92393881	5850	4.447560483	1.504055284	-1.645	1.05E-14	5.89E-13
CCN1	cellular communication network factor 1	protein_coding	chr1:85580761-	2443	14.08697889	5.284499479	-1.502	1.10E-14	6.00E-13
GNG12	G protein subunit gamma 12	protein_coding	chr1:67701475-	4715	17.69819989	8.728121576	-1.107	1.10E-14	6.00E-13
DPF3	double PHD fingers 3	protein_coding	chr14:72619296	6889	14.49385431	7.353853131	-1.064	1.11E-14	6.04E-13
RCAN1	regulator of calcineurin 1	protein_coding	chr21:34513142	6964	7.156760939	2.752107754	-1.462	1.20E-14	6.45E-13
VKORC1L1	vitamin K epoxide reductase complex sub	protein_coding	chr7:65873074-	6092	8.79563805	4.374970495	-1.093	1.20E-14	6.45E-13
ANKRD6	ankyrin repeat domain 6	protein_coding	chr6:89433152-	8347	8.546273449	4.697556715	-0.949	1.23E-14	6.46E-13
PBX3	PBX homeobox 3	protein_coding	chr9:125747345	4529	6.099049701	2.139837268	-1.595	1.23E-14	6.46E-13
PXDN	peroxidasin	protein_coding	chr2:1631887-1	11266	8.728196018	3.441384156	-1.427	1.23E-14	6.46E-13
SLC25A1	solute carrier family 25 member 1	protein_coding	chr22:19175581	1999	21.665165	8.746054619	-1.397	1.23E-14	6.46E-13
TERF2IP	TERF2 interacting protein	protein_coding	chr16:75647773	4422	23.98615045	13.26259096	-0.941	1.29E-14	6.72E-13
IRAK1	interleukin 1 receptor associated kinase 1	protein_coding	chrX:154010506	3917	7.355932663	2.799472771	-1.481	1.32E-14	6.80E-13
DUSP5	dual specificity phosphatase 5	protein_coding	chr10:11049790	2535	42.26688708	22.43511254	-1.001	1.35E-14	6.94E-13
PHACTR4	phosphatase and actin regulator 4	protein_coding	chr1:28369582-	7317	8.996943886	4.543538919	-1.072	1.41E-14	7.22E-13
CDK6	cyclin dependent kinase 6	protein_coding	chr7:92604921-	12260	11.08304882	4.277261747	-1.457	1.43E-14	7.28E-13
PPT1	palmitoyl-protein thioesterase 1	protein_coding	chr1:40071461-	5088	5.2811322	1.764623779	-1.668	1.45E-14	7.35E-13
IGFBP3	insulin like growth factor binding protein	protein_coding	chr7:45912245-	4571	7.056689208	2.776702506	-1.43	1.47E-14	7.41E-13
CAND1	cullin associated and neddylation dissoci	protein_coding	chr12:67269358	13580	10.75896465	5.407451949	-1.078	1.49E-14	7.47E-13
PTPRJ	protein tyrosine phosphatase receptor typ	protein_coding	chr11:47980558	9620	3.02507251	1.12195663	-1.515	1.51E-14	7.51E-13
MICAL2	microtubule associated monooxygenase,	protein_coding	chr11:12094008	20191	15.86234747	7.937018954	-1.084	1.54E-14	7.64E-13
MATN2	matrilin 2	protein_coding	chr8:97868840-	6941	16.12599268	7.212300158	-1.247	1.57E-14	7.74E-13
HIPK1	homeodomain interacting protein kinase	protein_coding	chr1:113929324	9148	7.399293027	2.373226323	-1.722	1.61E-14	7.88E-13
ATP1B1	ATPase Na+/K+ transporting subunit beta	protein_coding	chr1:169105697	2962	132.8623996	69.71809025	-1.016	1.65E-14	7.93E-13
TNRC6C-AS1	TNRC6C antisense RNA 1	lncRNA	chr17:78107398	4402	6.083628989	1.698977846	-1.921	1.65E-14	7.93E-13
NDFIP1	Nedd4 family interacting protein 1	protein_coding	chr5:142108779	5747	22.07383391	11.13681194	-1.073	1.67E-14	7.99E-13
EFNA1	ephrin A1	protein_coding	chr1:155127876	2165	15.13511018	5.946518884	-1.43	1.80E-14	8.50E-13
LPGAT1	lysophosphatidylglycerol acyltransferase	protein_coding	chr1:211743457	8790	8.964645163	4.510596384	-1.077	1.81E-14	8.50E-13
ELMOD2	ELMO domain containing 2	protein_coding	chr4:140524168	6617	5.881627497	2.637820206	-1.244	1.94E-14	9.03E-13
COLQ	collagen like tail subunit of asymmetric ac	protein_coding	chr3:15450133-	4175	15.01045975	6.115580399	-1.38	1.97E-14	9.12E-13
GREB1	growth regulating estrogen receptor bind	protein_coding	chr2:11482341-	12432	3.52075774	1.616814975	-1.206	1.99E-14	9.12E-13
SHC2	SHC adaptor protein 2	protein_coding	chr19:416583-4	4131	12.59186166	5.666887798	-1.236	1.98E-14	9.12E-13
RGS3	regulator of G protein signaling 3	protein_coding	chr9:113444731	13793	13.06425131	7.777984501	-0.834	2.08E-14	9.52E-13
DENND1B	DENN domain containing 1B	protein_coding	chr1:197504748	13203	3.542730972	1.474517226	-1.351	2.14E-14	9.76E-13
LRRC2	leucine rich repeat containing 2	protein_coding	chr3:46515385-	5634	3.631860405	0.951496583	-2.016	2.29E-14	1.04E-12
FRK	fyn related Src family tyrosine kinase	protein_coding	chr6:115931149	13363	1.318727326	0.236637395	-2.565	2.38E-14	1.07E-12
CGNL1	cingulin like 1	protein_coding	chr15:57375967	8663	16.91842433	7.566353649	-1.245	2.43E-14	1.08E-12
STAT5B	signal transducer and activator of transcri	protein_coding	chr17:42199168	6447	8.821228749	4.258012756	-1.135	2.50E-14	1.11E-12
RBM38	RNA binding motif protein 38	protein_coding	chr20:57391396	2697	111.9109348	63.2222775	-0.91	2.68E-14	1.18E-12
AASS	aminoadipate-semialdehyde synthase	protein_coding	chr7:122073549	6664	26.98814671	15.64549901	-0.873	2.72E-14	1.20E-12
SLC39A9	solute carrier family 39 member 9	protein_coding	chr14:69398015	7138	4.841379162	1.820385902	-1.497	2.80E-14	1.23E-12
TRIB2	tribbles pseudokinase 2	protein_coding	chr2:12716889-	4588	13.54811792	6.587755168	-1.125	2.83E-14	1.23E-12

FNIP2	folliculin interacting protein 2	protein_coding	chr4:158769026	10981	4.945360447	1.780247863	-1.556	2.85E-14	1.24E-12
SF3B4	splicing factor 3b subunit 4	protein_coding	chr1:149923317	1704	25.3636597	11.66187919	-1.206	2.91E-14	1.25E-12
CPNE5	copine 5	protein_coding	chr6:36740775-	5772	8.803986769	2.882626359	-1.693	3.01E-14	1.27E-12
DAZAP2	DAZ associated protein 2	protein_coding	chr12:51238724	5299	17.41363951	9.455417612	-0.967	3.02E-14	1.27E-12
MPP2	membrane palmitoylated protein 2	protein_coding	chr17:43875357	6114	10.38802437	5.316769274	-1.052	2.99E-14	1.27E-12
SPSB4	splA/ryanodine receptor domain and SOC	protein_coding	chr3:141051347	3795	15.86641872	8.37720092	-1.007	2.99E-14	1.27E-12
TAPBP	TAP binding protein	protein_coding	chr6:33299694-	5056	23.02520177	10.74166812	-1.187	3.01E-14	1.27E-12
XPR1	xenotropic and polytropic retrovirus rece	protein_coding	chr1:180632004	8903	26.32723386	11.7013341	-1.254	3.07E-14	1.28E-12
PHC2	polyhomeotic homolog 2	protein_coding	chr1:33323623-	4975	20.05668706	9.432292173	-1.174	3.29E-14	1.36E-12
ZMYM2	zinc finger MYM-type containing 2	protein_coding	chr13:19958670	13618	8.372180319	4.653345489	-0.934	3.36E-14	1.39E-12
OCRL	OCRL inositol polyphosphate-5-phosphat	protein_coding	chrX:129539849 6080		5.562898386	2.603643581	-1.18	3.40E-14	1.39E-12
SELENOI	selenoprotein I	protein_coding	chr2:26308547-	8552	6.319625594	3.080796988	-1.122	3.42E-14	1.39E-12
ZFP36L2	ZFP36 ring finger protein like 2	protein_coding	chr2:43222402-	3693	39.53068449	21.09942792	-0.991	3.41E-14	1.39E-12
IGFBP7	insulin like growth factor binding protein	protein_coding	chr4:57030773-	1930	162.3953542	88.36598558	-0.963	3.44E-14	1.40E-12
PHGDH	phosphoglycerate dehydrogenase	protein_coding	chr1:119648411	9217	4.702726269	2.466909249	-1.016	3.74E-14	1.50E-12
SLC2A4	solute carrier family 2 member 4	protein_coding	chr17:7281667-	4046	42.22477097	17.69649073	-1.338	3.87E-14	1.55E-12
DNAL1	dynein axonemal light chain 1	protein_coding	chr14:73644875	8884	2.669819622	0.928249492	-1.61	4.06E-14	1.61E-12
MARK1	microtubule affinity regulating kinase 1	protein_coding	chr1:220528183	5417	10.47053487	5.268694802	-1.078	4.08E-14	1.61E-12
RPP25	ribonuclease P and MRP subunit p25	protein_coding	chr15:74954416	3049	6.76173572	2.404479065	-1.575	4.16E-14	1.63E-12
CERK	ceramide kinase	protein_coding	chr22:46684410	4930	31.14547433	17.42326247	-0.924	4.47E-14	1.74E-12
SLC38A1	solute carrier family 38 member 1	protein_coding	chr12:46183063	10015	61.79360748	32.5268435	-1.012	4.54E-14	1.77E-12
ZNF704	zinc finger protein 704	protein_coding	chr8:80628451-	15008	5.455981022	1.895232814	-1.609	4.59E-14	1.78E-12
LDLR	low density lipoprotein receptor	protein_coding	chr19:11089362	7718	18.39522128	9.85097561	-0.986	4.77E-14	1.84E-12
MYH7	myosin heavy chain 7	protein_coding	chr14:23412740	6027	3368.861177	1511.24423	-1.243	4.81E-14	1.84E-12
AXL	AXL receptor tyrosine kinase	protein_coding	chr19:41219223	5134	5.158943312	1.247933929	-2.127	4.91E-14	1.87E-12
DCAF7	DDB1 and CUL4 associated factor 7	protein_coding	chr17:63550477	6572	25.53346961	14.73135376	-0.879	5.02E-14	1.91E-12
ZFYVE16	zinc finger FYVE-type containing 16	protein_coding	chr5:80408013-	15490	6.570018056	3.747661334	-0.895	5.17E-14	1.93E-12
GUCY1A2	guanylate cyclase 1 soluble subunit alpha	protein_coding	chr11:10667401	16361	5.728365547	2.494096993	-1.283	5.19E-14	1.94E-12
CLDND1	claudin domain containing 1	protein_coding	chr3:98497912-	5210	9.219558112	4.145539162	-1.239	5.21E-14	1.94E-12
DPYSL4	dihydropyrimidinase like 4	protein_coding	chr10:13218694	3111	35.75396026	21.20492463	-0.84	5.45E-14	2.02E-12
ICK	intestinal cell kinase	protein_coding	chr6:53001279-	6259	5.225565939	2.112826472	-1.391	5.47E-14	2.02E-12
SDCBP	syndecan binding protein	protein_coding	chr8:58552924-	4777	26.63934391	14.38763909	-0.975	5.49E-14	2.02E-12
KRBA1	KRAB-A domain containing 1	protein_coding	chr7:149714781	5337	21.65984206	9.945899146	-1.207	5.68E-14	2.08E-12
UBE2D1	ubiquitin conjugating enzyme E2 D1	protein_coding	chr10:58334979	3480	9.12294896	3.132061031	-1.625	6.05E-14	2.19E-12
ZCCHC24	zinc finger CCHC-type containing 24	protein_coding	chr10:79382325	5051	57.76344524	33.10654853	-0.889	6.13E-14	2.21E-12
ZNF267	zinc finger protein 267	protein_coding	chr16:31873807	6284	1.859774378	0.381332388	-2.37	6.12E-14	2.21E-12
RBX1	ring-box 1	protein_coding	chr22:40951347	3092	21.41445858	10.04028932	-1.178	6.47E-14	2.32E-12
ACTA1	actin alpha 1, skeletal muscle	protein_coding	chr1:229431245	1495	405.7545351	178.1007006	-1.275	6.55E-14	2.34E-12
NAA60	N(alpha)-acetyltransferase 60, NatF cataly	protein_coding	chr16:3443649-	7349	7.285019721	3.708605638	-1.061	6.72E-14	2.38E-12
AMMECR1	Alport syndrome, mental retardation, mic	protein_coding	chrX:110194186	6362	1.294354132	0.177772955	-2.947	6.81E-14	2.41E-12
IGFBP2	insulin like growth factor binding protein	protein_coding	chr2:216632828	4742	8.979846803	3.386453004	-1.495	6.84E-14	2.41E-12
PPP1R12C	protein phosphatase 1 regulatory subunit	protein_coding	chr19:55090914	7803	18.45547401	11.1977732	-0.807	6.88E-14	2.42E-12
DAAM1	dishevelled associated activator of morph	protein_coding	chr14:59188646	10712	17.88959385	10.76549956	-0.819	7.11E-14	2.49E-12
NPPA	natriuretic peptide A	protein_coding	chr1:11845709-	1120	4214.761415	1776.214056	-1.33	7.12E-14	2.49E-12
UGP2	UDP-glucose pyrophosphorylase 2	protein_coding	chr2:63840940-	6501	78.35787387	43.41303824	-0.938	7.20E-14	2.51E-12
LSS	lanosterol synthase	protein_coding	chr21:46188141	7238	16.68991973	9.124411141	-0.957	7.24E-14	2.52E-12
ATP1B2	ATPase Na+/K+ transporting subunit beta	protein_coding	chr17:7646627-	3464	21.70331385	10.41107692	-1.145	7.33E-14	2.54E-12
SLC4A4	solute carrier family 4 member 4	protein_coding	chr4:71062667-	9120	4.507928085	2.126689996	-1.168	7.49E-14	2.59E-12
UBE2J1	ubiquitin conjugating enzyme E2 J1	protein_coding	chr6:89326625-	4164	14.82237542	6.559000895	-1.263	7.79E-14	2.67E-12
OSGIN2	oxidative stress induced growth inhibitor	protein_coding	chr8:89901849-	4655	4.778577434	1.886226291	-1.427	7.95E-14	2.72E-12
PCMTD1	protein-L-isoaspartate (D-aspartate) O-me	protein_coding	chr8:51817575-	7323	12.77462994	6.668243685	-1.023	8.30E-14	2.83E-12
RC3H2	ring finger and CCCH-type domains 2	protein_coding	chr9:122844556	11563	10.3401209	4.755194778	-1.207	8.40E-14	2.86E-12
PLEKHA2	pleckstrin homology domain containing A	protein_coding	chr8:38901235-	6328	14.47200211	6.267153032	-1.293	8.48E-14	2.87E-12
SLC40A1	solute carrier family 40 member 1	protein_coding	chr2:189560579	4330	17.49260023	10.10789589	-0.878	8.83E-14	2.98E-12
TSPAN4	tetraspanin 4	protein_coding	chr11:842812-8	5837	6.237517022	3.191175052	-1.053	8.94E-14	3.01E-12
SLC2A12	solute carrier family 2 member 12	protein_coding	chr6:133988697	4468	10.72946726	5.542176155	-1.037	9.34E-14	3.13E-12
APOE	apolipoprotein E	protein_coding	chr19:44905791	2154	118.5559874	48.99241424	-1.36	9.38E-14	3.13E-12
DES	desmin	protein_coding	chr2:219418377	2464	152.9437014	54.6186058	-1.566	9.52E-14	3.16E-12
TRAK2	trafficking kinesin protein 2	protein_coding	chr2:201377207	7516	20.72662837	12.35975914	-0.832	9.53E-14	3.16E-12
CDCA7L	cell division cycle associated 7 like	protein_coding	chr7:21900899-	3894	25.20032643	12.9769009	-1.042	9.60E-14	3.17E-12
COMMD8	COMM domain containing 8	protein_coding	chr4:47450787-	1758	16.54582822	7.59870062	-1.208	9.63E-14	3.17E-12
GNB4	G protein subunit beta 4	protein_coding	chr3:179396088	6922	10.91029285	5.773652161	-1.003	9.63E-14	3.17E-12
TMEM127	transmembrane protein 127	protein_coding	chr2:96248516-	6367	25.03372295	15.21675428	-0.804	9.73E-14	3.19E-12
PLXNB2	plexin B2	protein_coding	chr22:50274979	7276	53.37834014	27.36927399	-1.049	1.00E-13	3.28E-12
LCLAT1	lysocardiolipin acyltransferase 1	protein_coding	chr2:30447226-	7517	3.648941511	1.714217329	-1.176	1.05E-13	3.36E-12
NCKAP5L	NCK associated protein 5 like	protein_coding	chr12:49791146 8090		21.29032827	12.80197766	-0.82	1.04E-13	3.36E-12
NXPH4	neurexophilin 4	protein_coding	chr12:57216794	2151	8.036024938	2.225369879	-1.933	1.04E-13	3.36E-12
TMOD2	tropomodulin 2	protein_coding	chr15:51751597	11595	2.715085229	0.719382036	-1.996	1.04E-13	3.36E-12
TNFRSF12A	TNF receptor superfamily member 12A	protein_coding	chr16:3018445-	1848	92.63341368	45.38691684	-1.116	1.05E-13	3.36E-12
TRAPPC9	trafficking protein particle complex 9	protein_coding	chr8:139727725	9655	3.966948176	2.150758849	-0.97	1.08E-13	3.43E-12
SNX4	sorting nexin 4	protein_coding	chr3:125446650	2708	16.4241156	7.234623452	-1.27	1.09E-13	3.46E-12
H2AFV	H2A histone family member V	protein_coding	chr7:44826791-	4749	28.23886889	14.50645922	-1.048	1.14E-13	3.59E-12
PAPPA2	pappalysin 2	protein_coding	chr1:176463171	10926	1.531396338	0.217281572	-2.895	1.14E-13	3.59E-12
UBE2J2	ubiquitin conjugating enzyme E2 J2	protein_coding	chr1:1253909-1	4712	10.05750273	5.512036964	-0.954	1.14E-13	3.59E-12
AQP10	aquaporin 10	protein_coding	chr1:154321090	2292	4.874663497	0.754946194	-2.776	1.17E-13	3.68E-12
PROS1	protein S	protein_coding	chr3:93873033-	5444	43.90504893	24.42518699	-0.932	1.19E-13	3.73E-12
BAMBI	BMP and activin membrane bound inhibit	protein_coding	chr10:28677510	2139	73.34478474	36.85249738	-1.08	1.24E-13	3.85E-12
PFDN6	prefoldin subunit 6	protein_coding	chr6:33289302-	2953	6.080124926	1.634264688	-1.976	1.25E-13	3.85E-12
NUDT21	nudix hydrolase 21	protein_coding	chr16:56429133	5930	15.60227551	9.392121909	-0.818	1.26E-13	3.87E-12
SQSTM1	sequestosome 1	protein_coding	chr5:179806398	7535	39.11859572	24.56149879	-0.757	1.28E-13	3.93E-12
TMED8	transmembrane p24 trafficking protein fa	protein_coding	chr14:77335029	7761	3.194059034	1.185439757	-1.511	1.28E-13	3.93E-12
OIP5-AS1	OIP5 antisense RNA 1	lncRNA	chr15:41283990	12673	15.16885242	9.301176101	-0.792	1.28E-13	3.93E-12
CBFB	core-binding factor subunit beta	protein_coding	chr16:67028984	4652	9.254993498	4.078521485	-1.268	1.30E-13	3.96E-12
RELN	reelin	protein_coding	chr7:103471784	12784	1.703608377	0.585258216	-1.628	1.30E-13	3.96E-12
GTPBP1	GTP binding protein 1	protein_coding	chr22:38705742	8021	12.49207506	6.85060662	-0.953	1.31E-13	3.97E-12
LEPROT	leptin receptor overlapping transcript	protein_coding	chr1:65420587-	5237	20.60497664	12.93529523	-0.758	1.31E-13	3.98E-12
PPP1R15B	protein phosphatase 1 regulatory subunit	protein_coding	chr1:204403381	5259	11.17500809	6.8082974	-0.801	1.35E-13	4.06E-12
TBL1XR1	transducin beta like 1 X-linked receptor 1	protein_coding	chr3:177019340	11603	9.863194358	6.003758761	-0.802	1.35E-13	4.07E-12
ASPH	aspartate beta-hydroxylase	protein_coding	chr8:61500556-	20047	15.91484877	10.09245654	-0.743	1.40E-13	4.22E-12
PALM2	paralemmin 2	protein_coding	chr9:109640788	9921	4.253624425	2.127397859	-1.083	1.44E-13	4.29E-12
RGL1	ral guanine nucleotide dissociation stimu	protein_coding	chr1:183636085	5305	16.83296037	10.26927053	-0.799	1.44E-13	4.29E-12
CKAP2	cytoskeleton associated protein 2	protein_coding	chr13:52455429	4547	30.07059409	16.11679968	-0.986	1.46E-13	4.33E-12
ST3GAL1	ST3 beta-galactoside alpha-2,3-sialyltrans	protein_coding	chr8:133454848	8852	23.91338912	12.6105407	-1.009	1.51E-13	4.46E-12
EMP2	epithelial membrane protein 2	protein_coding	chr16:10528422	8652	53.5950165	32.92118892	-0.789	1.54E-13	4.53E-12
KCNC4	potassium voltage-gated channel subfami	protein_coding	chr1:110211343	25042	1.935506826	1.05486594	-0.962	1.58E-13	4.60E-12
KCTD17	potassium channel tetramerization doma	protein_coding	chr22:37051736	2786	20.28746316	11.20894735	-0.944	1.61E-13	4.67E-12
ACER3	alkaline ceramidase 3	protein_coding	chr11:76860867	8442	6.610830142	3.144099273	-1.159	1.64E-13	4.74E-12
TAF9B	TATA-box binding protein associated facto	protein_coding	chrX:78129748-	2735	7.959704267	3.767803108	-1.162	1.66E-13	4.78E-12
CRY2	cryptochrome circadian regulator 2	protein_coding	chr11:45847118	6200	26.47535034	15.61537459	-0.848	1.67E-13	4.80E-12
VTI1B	vesicle transport through interaction with	protein_coding	chr14:67647085	5768	10.91808212	6.612627591	-0.81	1.69E-13	4.83E-12
TNS3	tensin 3	protein_coding	chr7:47275154-	11764	6.960981992	3.833591557	-0.946	1.73E-13	4.91E-12
PLEKHA6	pleckstrin homology domain containing A	protein_coding	chr1:204218853	8262	8.94856182	4.299215764	-1.143	1.81E-13	5.12E-12
SSRP1	structure specific recognition protein 1	protein_coding	chr11:57325986	6337	14.05732201	8.421778384	-0.825	1.83E-13	5.15E-12
PHACTR2	phosphatase and actin regulator 2	protein_coding	chr6:143536845	11224	8.827055525	4.135189751	-1.178	1.87E-13	5.25E-12
EPHB3	EPH receptor B3	protein_coding	chr3:184561785	4530	27.39880095	14.61041676	-0.993	1.89E-13	5.28E-12
TACC1	transforming acidic coiled-coil containing	protein_coding	chr8:38728186-	12577	3.781027559	1.823659361	-1.136	1.90E-13	5.31E-12
TTC39A	tetratricopeptide repeat domain 39A	protein_coding	chr1:51287258-	9362	5.357327975	2.430695325	-1.226	1.90E-13	5.31E-12
NFATC3	nuclear factor of activated T cells 3	protein_coding	chr16:68084751	8806	3.731704554	1.804628863	-1.134	1.92E-13	5.34E-12
PDPK1	3-phosphoinositide dependent protein ki	protein_coding	chr16:2537979-	10911	7.256751805	4.080947889	-0.916	1.95E-13	5.40E-12

PPIL1	peptidylprolyl isomerase like 1	protein_coding	chr6:36854827-	1589	26.63976973	13.08756919	-1.112	1.95E-13	5.41E-12
ALCAM	activated leukocyte cell adhesion molecul	protein_coding	chr3:10536690	7179	10.79612401	6.344817671	-0.853	1.99E-13	5.47E-12
MRS2	magnesium transporter MRS2	protein_coding	chr6:24402908-	5133	17.85862177	10.39270705	-0.867	1.99E-13	5.47E-12
GADD45G	growth arrest and DNA damage inducible	protein_coding	chr9:89605012-	1323	20.99375051	7.687686703	-1.538	2.01E-13	5.50E-12
COMTD1	catechol-O-methyltransferase domain cor	protein_coding	chr10:75233969	1580	26.79583685	10.82974342	-1.391	2.06E-13	5.64E-12
OTUD3	OTU deubiquitinase 3	protein_coding	chr1:19882395-	6885	4.014960759	1.782351702	-1.258	2.17E-13	5.80E-12
PGBD5	piggyBac transposable element derived 5	protein_coding	chr1:23031449(	11257	2.42171262	1.079774477	-1.252	2.17E-13	5.80E-12
CSNK1A1	casein kinase 1 alpha 1	protein_coding	chr5:14949298	8505	16.02282981	10.23583489	-0.733	2.27E-13	5.99E-12
AC107294.2	novel transcript	lncRNA	chr3:18471224	4617	3.373369501	0.888628877	-2.015	2.28E-13	6.01E-12
ITGA6	integrin subunit alpha 6	protein_coding	chr2:17242735	6710	5.429986044	2.72579478	-1.078	2.29E-13	6.01E-12
HPS5	HPS5 biogenesis of lysosomal organelles c	protein_coding	chr11:18278668	6433	5.171503039	2.605304097	-1.078	2.32E-13	6.10E-12
TCF12	transcription factor 12	protein_coding	chr15:5691862	9446	8.00225799	4.214720764	-1.011	2.34E-13	6.11E-12
SLC39A8	solute carrier family 39 member 8	protein_coding	chr4:102251041	6480	3.413050015	1.560396591	-1.212	2.36E-13	6.18E-12
ADAMTSL5	ADAMTS like 5	protein_coding	chr19:1505022-	3621	11.88418327	5.879793251	-1.099	2.40E-13	6.24E-12
XPO4	exportin 4	protein_coding	chr13:2077732	10500	20.82912356	10.40897706	-1.085	2.41E-13	6.26E-12
APBB1	amyloid beta precursor protein binding fa	protein_coding	chr11:6395124-	5244	32.57734088	20.83435454	-0.731	2.42E-13	6.27E-12
PLEKHA3	pleckstrin homology domain containing A	protein_coding	chr2:17848045	14205	1.72529997	0.786946923	-1.218	2.43E-13	6.28E-12
KIAA0319L	KIAA0319 like	protein_coding	chr1:35433492-	9619	14.57027479	9.240090077	-0.743	2.44E-13	6.29E-12
PHF10	PHD finger protein 10	protein_coding	chr6:16970390	5982	21.75696169	14.0676141	-0.715	2.48E-13	6.37E-12
SESN2	sestrin 2	protein_coding	chr1:28259518-	3462	15.21982499	8.678240768	-0.899	2.49E-13	6.38E-12
SH3D19	SH3 domain containing 19	protein_coding	chr4:15110275	7789	25.77157765	16.03336562	-0.771	2.58E-13	6.58E-12
NFIB	nuclear factor I B	protein_coding	chr9:14081843-	12765	5.347109039	2.944794545	-0.946	2.65E-13	6.74E-12
PLPP7	phospholipid phosphatase 7 (inactive)	protein_coding	chr9:13128945	4349	14.47841995	7.418020389	-1.051	2.67E-13	6.76E-12
OXSR1	oxidative stress responsive kinase 1	protein_coding	chr3:38165089-	6071	9.290063491	4.857895954	-1.02	2.72E-13	6.88E-12
CLASP2	cytoplasmic linker associated protein 2	protein_coding	chr3:33496245-	15214	6.104450272	3.795090048	-0.772	2.77E-13	6.96E-12
ARPP19	cAMP regulated phosphoprotein 19	protein_coding	chr15:52547045	6917	25.43140954	15.3429609	-0.815	2.80E-13	7.03E-12
NCKIPSD	NCK interacting protein with SH3 domain	protein_coding	chr3:48673844-	3431	13.51887889	5.560109709	-1.364	2.82E-13	7.05E-12
TMEM129	transmembrane protein 129	protein_coding	chr4:1715952-1	3442	39.85760582	22.45684505	-0.912	2.95E-13	7.38E-12
OGDHL	oxoglutarate dehydrogenase like	protein_coding	chr10:49734641	5726	10.48599951	4.879781425	-1.187	2.97E-13	7.39E-12
CPEB4	cytoplasmic polyadenylation element bin	protein_coding	chr5:17388834	10444	22.24768991	13.32280065	-0.825	3.05E-13	7.54E-12
FADS3	fatty acid desaturase 3	protein_coding	chr11:61873519	6294	8.132533909	4.600549784	-0.907	3.05E-13	7.54E-12
ANKFY1	ankyrin repeat and FYVE domain containi	protein_coding	chr17:4163821-	14224	6.920511266	3.921745821	-0.904	3.06E-13	7.55E-12
AIF1L	allograft inflammatory factor 1 like	protein_coding	chr9:13109647	5299	2.794506957	0.96358465	-1.62	3.07E-13	7.57E-12
PANK3	pantothenate kinase 3	protein_coding	chr5:16854849	10348	21.0310989	13.00612491	-0.779	3.12E-13	7.67E-12
PLEKHM2	pleckstrin homology and RUN domain cor	protein_coding	chr1:15684320-	4134	36.81000797	22.92816438	-0.769	3.24E-13	7.94E-12
PLEKHG3	pleckstrin homology and RhoGEF domain	protein_coding	chr14:64704102	11924	5.553170694	2.951722968	-0.997	3.27E-13	7.99E-12
PGAM1	phosphoglycerate mutase 1	protein_coding	chr10:97426191	2540	115.6723554	48.7707159	-1.336	3.31E-13	8.07E-12
AP3M1	adaptor related protein complex 3 subuni	protein_coding	chr10:74120255	5912	15.05290224	8.50477897	-0.91	3.34E-13	8.12E-12
FOXRED1	FAD dependent oxidoreductase domain c	protein_coding	chr11:12626905	5657	6.969115642	3.882878862	-0.931	3.49E-13	8.43E-12
KCTD2	potassium channel tetramerization doma	protein_coding	chr17:75032575	5279	10.17838147	5.616284502	-0.943	3.49E-13	8.43E-12
CALM1	calmodulin 1	protein_coding	chr14:90396502	7833	16.94772668	10.61772858	-0.76	3.52E-13	8.49E-12
ACVR2B	activin A receptor type 2B	protein_coding	chr3:38453890-	15705	3.850100653	1.479610282	-1.462	3.55E-13	8.53E-12
CD47	CD47 molecule	protein_coding	chr3:10804309	8228	8.235276755	4.687781672	-0.899	3.55E-13	8.53E-12
LNX2	ligand of numb-protein X 2	protein_coding	chr13:2754591	4815	7.514035524	3.667860474	-1.121	3.58E-13	8.57E-12
WASF3	WASP family member 3	protein_coding	chr13:26557703	5162	8.710203357	5.203823086	-0.829	3.59E-13	8.59E-12
CDK18	cyclin dependent kinase 18	protein_coding	chr1:20550459	6831	4.375629182	1.447534695	-1.675	3.68E-13	8.78E-12
LYRM7	LYR motif containing 7	protein_coding	chr5:13117094	6219	8.902619412	4.84316605	-0.963	3.69E-13	8.79E-12
CD24	CD24 molecule	protein_coding	chr6:10696983	3096	4.608069574	1.174659005	-2.069	3.75E-13	8.91E-12
MOB1A	MOB kinase activator 1A	protein_coding	chr2:74152528-	6440	10.68837984	6.538482961	-0.795	3.77E-13	8.92E-12
PRKRA	protein activator of interferon induced pr	protein_coding	chr2:17843141	3885	11.32599568	5.241898793	-1.196	3.77E-13	8.92E-12
LBR	lamin B receptor	protein_coding	chr1:22540150	5710	4.036890849	1.600218745	-1.417	3.78E-13	8.93E-12
FOXO3	forkhead box O3	protein_coding	chr6:10855983	7670	6.567974558	2.592264253	-1.425	3.79E-13	8.93E-12
TMEM40	transmembrane protein 40	protein_coding	chr3:12733528-	3297	5.603891	2.240163909	-1.405	3.85E-13	9.04E-12
CAPN7	calpain 7	protein_coding	chr3:15206152-	5596	10.02623632	6.117662209	-0.798	3.86E-13	9.05E-12
ARRB1	arrestin beta 1	protein_coding	chr11:75260122	9888	3.347565115	1.925402978	-0.883	4.08E-13	9.48E-12
KIAA0040	KIAA0040	protein_coding	chr1:17515698	4767	3.02273055	0.634440389	-2.336	4.08E-13	9.48E-12
SEC14L5	SEC14 like lipid binding 5	protein_coding	chr16:4958330-	6463	9.17803361	3.890314246	-1.323	4.07E-13	9.48E-12
EPS8	epidermal growth factor receptor pathwa	protein_coding	chr12:15620134	8540	3.381122623	1.630341822	-1.14	4.14E-13	9.61E-12
ARHGAP26	Rho GTPase activating protein 26	protein_coding	chr5:14277037	17158	4.318042894	1.918648813	-1.254	4.23E-13	9.80E-12
KCNJ4	potassium voltage-gated channel subfami	protein_coding	chr22:38426327	2065	22.03828024	13.42507391	-0.801	4.31E-13	9.93E-12
EPHA2	EPH receptor A2	protein_coding	chr1:16124337-	4141	21.54296808	10.65579798	-1.1	4.40E-13	1.01E-11
RAP2A	RAP2A, member of RAS oncogene family	protein_coding	chr13:97434169	5623	8.168954431	4.717283965	-0.879	4.41E-13	1.01E-11
GOLGA2	golgin A2	protein_coding	chr9:12825582	6961	17.38491176	10.90832596	-0.758	4.43E-13	1.01E-11
SPAG9	sperm associated antigen 9	protein_coding	chr17:50962174	11966	8.425859399	5.216112635	-0.778	4.43E-13	1.01E-11
TMEM178A	transmembrane protein 178A	protein_coding	chr2:39664982-	2472	16.7036424	8.301647585	-1.093	4.61E-13	1.05E-11
CYCS	cytochrome c, somatic	protein_coding	chr7:25120091-	4457	138.707933	70.53639413	-1.059	4.62E-13	1.05E-11
PACSIN3	protein kinase C and casein kinase substra	protein_coding	chr11:47177522	2755	14.0919094	8.48943969	-0.818	4.77E-13	1.08E-11
ZBED1	zinc finger BED-type containing 1	protein_coding	chrX:2486414-2	4764	9.251332233	4.585205472	-1.096	4.79E-13	1.08E-11
FAM222B	family with sequence similarity 222 mem	protein_coding	chr17:28755978	5514	8.100507244	3.87721737	-1.147	4.81E-13	1.08E-11
GMNN	geminin DNA replication inhibitor	protein_coding	chr6:24774931-	2476	6.503147086	2.246655227	-1.619	4.86E-13	1.09E-11
TNNI3	troponin I3, cardiac type	protein_coding	chr19:55151767	2034	92.6471629	35.47104029	-1.465	4.88E-13	1.09E-11
TMED7	transmembrane p24 trafficking protein 7	protein_coding	chr5:11561321(	4055	13.45618342	7.722580899	-0.888	4.91E-13	1.10E-11
CLIC5	chloride intracellular channel 5	protein_coding	chr6:45880827-	9390	11.94367681	5.750790339	-1.139	5.06E-13	1.12E-11
PHC1	polyhomeotic homolog 1	protein_coding	chr12:8913896-	6134	5.202940612	2.661872446	-1.051	5.02E-13	1.12E-11
UCK2	uridine-cytidine kinase 2	protein_coding	chr1:16582761	6984	9.486958687	5.090527563	-0.983	5.05E-13	1.12E-11
PRDM2	PR/SET domain 2	protein_coding	chr1:13700198-	10923	5.751279228	2.807802665	-1.119	5.37E-13	1.18E-11
TSPAN13	tetraspanin 13	protein_coding	chr7:16753755-	2004	22.14523414	9.661955954	-1.281	5.40E-13	1.18E-11
TDGF1	teratocarcinoma-derived growth factor 1	protein_coding	chr3:46574534-	2489	10.77186139	3.80506007	-1.587	5.44E-13	1.19E-11
APCDD1	APC down-regulated 1	protein_coding	chr18:10454635	5937	3.033859372	0.566156628	-2.517	5.45E-13	1.19E-11
DIAPH1	diaphanous related formin 1	protein_coding	chr5:14151501	7375	6.914598261	4.041949492	-0.86	5.49E-13	1.19E-11
DIRAS1	DIRAS family GTPase 1	protein_coding	chr19:2714567-	3439	6.049490477	2.723882532	-1.239	5.49E-13	1.19E-11
RHOT2	ras homolog family member T2	protein_coding	chr16:668105-6	5155	44.2169077	27.91584628	-0.75	5.49E-13	1.19E-11
AZI2	5-azacytidine induced 2	protein_coding	chr3:28315003-	6272	6.036653723	3.415319539	-0.908	5.52E-13	1.19E-11
TMTC1	transmembrane and tetratricopeptide rep	protein_coding	chr12:29500840	14681	12.09641876	7.32256823	-0.81	5.63E-13	1.21E-11
ANXA3	annexin A3	protein_coding	chr4:78551747-	4895	17.10779757	8.535645237	-1.088	5.65E-13	1.22E-11
SPRY2	sprouty RTK signaling antagonist 2	protein_coding	chr13:80335976	3213	8.523737909	4.203954439	-1.108	5.66E-13	1.22E-11
FAM219B	family with sequence similarity 219 mem	protein_coding	chr15:74899992	5015	19.30198303	12.17824926	-0.75	5.76E-13	1.23E-11
NMNAT2	nicotinamide nucleotide adenylyltransfer	protein_coding	chr1:18324823	6943	1.754132858	0.502622114	-1.885	5.98E-13	1.28E-11
CACHD1	cache domain containing 1	protein_coding	chr1:64470129-	7022	17.02696577	11.14820517	-0.697	6.10E-13	1.29E-11
GALNT10	polypeptide N-acetylgalactosaminyltrans	protein_coding	chr5:15419073(	10496	3.327558883	1.530377433	-1.204	6.11E-13	1.29E-11
MLEC	malectin	protein_coding	chr12:12068714	6438	32.16081956	18.62120893	-0.873	6.10E-13	1.29E-11
SOD3	superoxide dismutase 3	protein_coding	chr4:24789912-	2018	11.13710287	4.044551106	-1.546	6.06E-13	1.29E-11
SLC35F5	solute carrier family 35 member F5	protein_coding	chr2:113705011	13398	6.454066925	3.948877158	-0.794	6.13E-13	1.29E-11
GPX3	glutathione peroxidase 3	protein_coding	chr5:15102043	3162	17.94098601	8.764326119	-1.12	6.17E-13	1.29E-11
EHD3	EH domain containing 3	protein_coding	chr2:31234152-	4825	88.40934519	57.38771441	-0.71	6.20E-13	1.30E-11
JPH3	junctophilin 3	protein_coding	chr16:87601835	6320	3.80179307	1.623088549	-1.317	6.22E-13	1.30E-11
MOAP1	modulator of apoptosis 1	protein_coding	chr14:93182199	2529	47.94813791	29.84859415	-0.771	6.28E-13	1.31E-11
CBR4	carbonyl reductase 4	protein_coding	chr4:16886377(	5110	6.373757264	3.361596096	-1.009	6.43E-13	1.34E-11
COL2A1	collagen type II alpha 1 chain	protein_coding	chr12:47972967	6662	5.471832615	2.484077452	-1.223	6.43E-13	1.34E-11
TENT5A	terminal nucleotidyltransferase 5A	protein_coding	chr6:81491439-	6053	13.11093086	7.867319528	-0.825	6.45E-13	1.34E-11
EIF3J	eukaryotic translation initiation factor 3 s	protein_coding	chr15:44537125	3127	32.65964327	17.5084934	-0.984	6.52E-13	1.35E-11
RNF157	ring finger protein 157	protein_coding	chr17:76142465	7913	2.081439095	0.822466162	-1.421	6.55E-13	1.35E-11
DDX3X	DEAD-box helicase 3 X-linked	protein_coding	chrX:41333284-	15892	17.49883703	11.06409797	-0.747	6.67E-13	1.37E-11
EIF2AK3	eukaryotic translation initiation factor 2 a	protein_coding	chr2:88556740-	5470	3.605174491	1.692749136	-1.177	6.70E-13	1.38E-11
RILP	Rab interacting lysosomal protein	protein_coding	chr17:1646145-	1892	23.63304967	13.82564243	-0.859	6.74E-13	1.38E-11
ABHD12	abhydrolase domain containing 12	protein_coding	chr20:25294743	3413	26.183503	16.36683803	-0.763	6.82E-13	1.39E-11
PARVA	parvin alpha	protein_coding	chr11:12377563	7916	10.65126953	6.670956235	-0.762	6.89E-13	1.41E-11
ATP7A	ATPase copper transporting alpha	protein_coding	chrX:77910656-	10920	2.450121616	1.226798688	-1.084	6.94E-13	1.41E-11
RAB15	RAB15, member RAS oncogene family	protein_coding	chr14:64945814	5015	8.953845493	3.852298632	-1.305	6.94E-13	1.41E-11

SORD	sorbitol dehydrogenase	protein_coding	chr15:45023181	6082	7.630740082	4.522710135	-0.839	7.16E-13	1.45E-11
VPS53	VPS53 subunit of GARP complex	protein_coding	chr17:508668-7	20899	2.008093591	1.145043602	-0.896	7.18E-13	1.45E-11
FAM102A	family with sequence similarity 102 meml	protein_coding	chr9:127940582	6573	15.48982687	8.532692414	-0.945	7.24E-13	1.46E-11
EDNRA	endothelin receptor type A	protein_coding	chr4:147480917	4238	42.10743993	28.01963345	-0.674	7.28E-13	1.47E-11
SOCS2	suppressor of cytokine signaling 2	protein_coding	chr12:93569814	6477	3.658946573	1.369068691	-1.507	7.42E-13	1.49E-11
MCMBP	minichromosome maintenance complex I	protein_coding	chr10:11982940	6000	7.7112154	4.259964205	-0.942	7.49E-13	1.50E-11
TK1	thymidine kinase 1	protein_coding	chr17:78174091	1845	9.993103775	4.368566953	-1.277	7.53E-13	1.51E-11
FAM234A	family with sequence similarity 234 meml	protein_coding	chr16:234521-2	6132	23.67228429	15.48513307	-0.699	7.71E-13	1.54E-11
AEBP1	AE binding protein 1	protein_coding	chr7:44104345-	4971	27.88736526	13.67484551	-1.114	7.74E-13	1.54E-11
CNOT6	CCR4-NOT transcription complex subunit	protein_coding	chr5:180494379	6619	7.024752345	3.185338787	-1.225	7.77E-13	1.55E-11
DMKN	dermokine	protein_coding	chr19:35497220	7444	2.623325022	0.925054426	-1.583	8.01E-13	1.59E-11
MSRB3	methionine sulfoxide reductase B3	protein_coding	chr12:65278643	7405	10.85836504	6.512652817	-0.825	8.14E-13	1.61E-11
CD81	CD81 molecule	protein_coding	chr11:2376177-	7394	53.95923277	34.33929578	-0.738	8.27E-13	1.63E-11
EXT1	exostosin glycosyltransferase 1	protein_coding	chr8:117794490	8325	6.6452129	3.668275894	-0.943	8.26E-13	1.63E-11
ZNF652	zinc finger protein 652	protein_coding	chr17:49289206	8810	3.043782666	1.653091431	-0.968	8.24E-13	1.63E-11
AGO4	argonaute RISC component 4	protein_coding	chr1:35808016-	7423	7.906381823	4.322819134	-0.956	8.48E-13	1.66E-11
MXD4	MAX dimerization protein 4	protein_coding	chr4:2247432-2	6873	12.01212568	6.228823338	-1.034	8.56E-13	1.67E-11
SLC39A7	solute carrier family 39 member 7	protein_coding	chr6:33200445-	2845	57.63013156	37.43955936	-0.708	8.89E-13	1.74E-11
DNAJC10	DnaJ heat shock protein family (Hsp40) m	protein_coding	chr2:182716041	21873	4.519290749	2.983757011	-0.684	8.93E-13	1.74E-11
MYLIP	myosin regulatory light chain interacting	protein_coding	chr6:16129086-	3072	11.90800221	7.060746869	-0.84	8.92E-13	1.74E-11
OSR1	odd-skipped related transcription factor 1	protein_coding	chr2:19351485-	5279	2.449754088	0.809151046	-1.685	9.06E-13	1.76E-11
TOMM40	translocase of outer mitochondrial memb	protein_coding	chr19:44890569	3610	33.61797279	20.62716653	-0.79	9.16E-13	1.77E-11
UNC13B	unc-13 homolog B	protein_coding	chr9:35161992-	17916	4.77550609	2.647558015	-0.935	9.16E-13	1.77E-11
ATP1A2	ATPase Na+/K+ transporting subunit alpha	protein_coding	chr1:160115759	6298	19.27926534	7.567415159	-1.436	9.20E-13	1.78E-11
CDKN1C	cyclin dependent kinase inhibitor 1C	protein_coding	chr11:2883213-	2478	7.069800505	3.087769613	-1.283	9.35E-13	1.80E-11
MMP24	matrix metallopeptidase 24	protein_coding	chr20:35226690	4376	1.605785929	0.149792628	-3.515	9.46E-13	1.82E-11
BMP1	bone morphogenetic protein 1	protein_coding	chr8:22165140-	7067	10.82755394	6.419560094	-0.84	9.58E-13	1.84E-11
PLIN3	perilipin 3	protein_coding	chr19:4838341-	2739	17.01376856	10.84026398	-0.737	1.02E-12	1.94E-11
SMC2	structural maintenance of chromosomes 2	protein_coding	chr9:104094260	6470	10.45522371	5.8824359	-0.914	1.03E-12	1.95E-11
SSBP3	single stranded DNA binding protein 3	protein_coding	chr1:54225432-	5227	12.11373032	6.7273348	-0.933	1.03E-12	1.95E-11
CHMP3	charged multivesicular body protein 3	protein_coding	chr2:86503430-	7019	7.199382477	4.30486843	-0.828	1.04E-12	1.95E-11
POMGNT2	protein O-linked mannose N-acetylglucosa	protein_coding	chr3:43079232-	2671	6.714931372	3.384050934	-1.075	1.05E-12	1.98E-11
PPP2R2A	protein phosphatase 2 regulatory subunit	protein_coding	chr8:26291508-	8777	5.98845003	3.482386947	-0.867	1.07E-12	2.00E-11
RAD23B	RAD23 homolog B, nucleotide excision re	protein_coding	chr9:107283137	4613	38.20157862	25.36577563	-0.677	1.08E-12	2.02E-11
HMGCR	3-hydroxy-3-methylglutaryl-CoA reductas	protein_coding	chr5:75336329-	6160	9.963184865	6.157989469	-0.779	1.10E-12	2.06E-11
SLC8A1	solute carrier family 8 member A1	protein_coding	chr2:40097270-	21922	59.2872096	35.6987264	-0.817	1.14E-12	2.11E-11
ADO	2-aminoethanethiol dioxygenase	protein_coding	chr10:62804857	3627	16.75595671	9.708734397	-0.873	1.16E-12	2.14E-11
SLK	STE20 like kinase	protein_coding	chr10:10396714	8035	12.81313124	8.31656236	-0.709	1.16E-12	2.15E-11
BRSK1	BR serine/threonine kinase 1	protein_coding	chr19:55282072	3841	6.242878495	2.749052239	-1.268	1.18E-12	2.16E-11
CLSTN2	calsyntenin 2	protein_coding	chr3:139935185	14596	3.348852111	1.1148701	-1.672	1.18E-12	2.16E-11
C20orf194	chromosome 20 open reading frame 194	protein_coding	chr20:3249305-	8541	11.99082913	6.734753774	-0.916	1.18E-12	2.17E-11
FGF1	fibroblast growth factor 1	protein_coding	chr5:142592178	4980	35.15094929	24.13785391	-0.629	1.19E-12	2.18E-11
IRGQ	immunity related GTPase Q	protein_coding	chr19:43584369	9869	6.519247055	3.481753133	-0.99	1.24E-12	2.26E-11
EIF5A2	eukaryotic translation initiation factor 5A	protein_coding	chr3:170888418	5541	2.51308607	1.020943372	-1.39	1.25E-12	2.27E-11
MDK	midkine	protein_coding	chr11:46380756	2196	40.57410885	20.78981817	-1.05	1.25E-12	2.27E-11
MARS	methionyl-tRNA synthetase	protein_coding	chr12:57475445	7028	21.4605477	14.43101128	-0.659	1.26E-12	2.28E-11
NDRG4	NDRG family member 4	protein_coding	chr16:58462846	9647	27.068932	17.40753112	-0.723	1.27E-12	2.29E-11
ARAP1	ArfGAP with RhoGAP domain, ankyrin rep	protein_coding	chr11:72685069	9056	5.854967141	3.332579778	-0.898	1.28E-12	2.30E-11
IGDCC4	immunoglobulin superfamily DCC subclas	protein_coding	chr15:65381484	7619	1.244438538	0.338465827	-1.965	1.30E-12	2.34E-11
CHPF	chondroitin polymerizing factor	protein_coding	chr2:219538948	3375	81.56980031	54.94416217	-0.656	1.31E-12	2.34E-11
STRA6	stimulated by retinoic acid 6	protein_coding	chr15:74179466	7669	5.078573523	2.424472358	-1.153	1.31E-12	2.35E-11
IL6R	interleukin 6 receptor	protein_coding	chr1:154405193	6453	1.246109685	0.28742716	-2.197	1.34E-12	2.39E-11
FITM2	fat storage inducing transmembrane prot	protein_coding	chr20:44302840	4628	34.72395319	20.90691779	-0.818	1.35E-12	2.40E-11
SLC4A2	solute carrier family 4 member 2	protein_coding	chr7:151057210	7570	28.92997621	18.38460959	-0.74	1.35E-12	2.41E-11
HTRA1	HtrA serine peptidase 1	protein_coding	chr10:12245855	2250	42.02027129	26.42911764	-0.754	1.36E-12	2.42E-11
STUM	stum, mechanosensory transduction med	protein_coding	chr1:226548764	7843	5.034168217	1.824638359	-1.547	1.36E-12	2.42E-11
TTLL12	tubulin tyrosine ligase like 12	protein_coding	chr22:43166622	4623	7.902078864	4.44146866	-0.919	1.37E-12	2.42E-11
TXNDC16	thioredoxin domain containing 16	protein_coding	chr14:52430596	4733	1.832108727	0.521662956	-1.898	1.37E-12	2.42E-11
KIF1A	kinesin family member 1A	protein_coding	chr2:240713761	16695	1.15643892	0.558864891	-1.131	1.39E-12	2.46E-11
GMPR	guanosine monophosphate reductase	protein_coding	chr6:16238580-	2232	10.45347332	4.882706366	-1.186	1.40E-12	2.46E-11
NEU3	neuraminidase 3	protein_coding	chr11:74988279	7212	4.450415429	2.563036679	-0.884	1.43E-12	2.51E-11
AIDA	axin interactor, dorsalization associated	protein_coding	chr1:222668013	4232	4.714501562	2.314369644	-1.112	1.44E-12	2.53E-11
NECAB3	N-terminal EF-hand calcium binding prot	protein_coding	chr20:33657087	4342	27.30431011	18.75889454	-0.628	1.47E-12	2.57E-11
PMP22	peripheral myelin protein 22	protein_coding	chr17:15229777	3191	25.55636226	14.32818991	-0.919	1.48E-12	2.58E-11
SUSD5	sushi domain containing 5	protein_coding	chr3:33150043-	4602	1.757142292	0.522162091	-1.829	1.50E-12	2.61E-11
MAPRE1	microtubule associated protein RP/EB fan	protein_coding	chr20:32819954	2562	135.5946689	91.86150287	-0.647	1.54E-12	2.67E-11
PHLDA1	pleckstrin homology like domain family A	protein_coding	chr12:76025447	8069	4.957542014	2.227499916	-1.238	1.56E-12	2.70E-11
MLLT11	MLLT11 transcription factor 7 cofactor	protein_coding	chr1:151057758	4075	69.96393149	40.73347355	-0.866	1.65E-12	2.84E-11
SBK1	SH3 domain binding kinase 1	protein_coding	chr16:28259228	5261	3.236184639	1.330039826	-1.368	1.65E-12	2.85E-11
FLRT2	fibronectin leucine rich transmembrane p	protein_coding	chr14:85530144	34901	1.051591835	0.489426642	-1.189	1.70E-12	2.92E-11
PDE4DIP	phosphodiesterase 4D interacting protein	protein_coding	chr1:148808181	27623	32.14064194	17.80690034	-0.936	1.73E-12	2.97E-11
EXOC6B	exocyst complex component 6B	protein_coding	chr2:72175984-	10197	19.91052308	12.53027359	-0.754	1.74E-12	2.98E-11
MLLT3	MLLT3 super elongation complex subunit	protein_coding	chr9:20341669-	8100	5.759684168	3.264260403	-0.906	1.76E-12	3.00E-11
PBXIP1	PBX homeobox interacting protein 1	protein_coding	chr1:154944076	3851	34.23094836	20.17122796	-0.85	1.78E-12	3.03E-11
SLC25A4	solute carrier family 25 member 4	protein_coding	chr4:185143266	4819	182.8712488	115.4678573	-0.749	1.78E-12	3.03E-11
PCYOX1	prenylcysteine oxidase 1	protein_coding	chr2:70257386-	5864	19.6840023	13.15840297	-0.667	1.79E-12	3.04E-11
GIPR	gastric inhibitory polypeptide receptor	protein_coding	chr19:45668221	5069	5.429577944	2.298042522	-1.321	1.87E-12	3.15E-11
DANCR	differentiation antagonizing non-protein	lncRNA	chr4:52712404-	2328	13.32260624	5.30034181	-1.413	1.89E-12	3.18E-11
VPS54	VPS54 subunit of GARP complex	protein_coding	chr2:63892146-	5447	6.973361804	3.912507348	-0.919	1.92E-12	3.22E-11
IER3	immediate early response 3	protein_coding	chr6:30743199-	1350	28.15263394	13.21795401	-1.177	1.98E-12	3.30E-11
TSC1	TSC complex subunit 1	protein_coding	chr9:132891348	17600	4.576692905	2.78507294	-0.802	1.99E-12	3.31E-11
TAF13	TATA-box binding protein associated facto	protein_coding	chr1:109062486	1400	18.19324763	8.341850267	-1.21	2.00E-12	3.32E-11
AC009041.2	novel transcript	lncRNA	chr16:975761-9	3905	3.979607966	1.456469644	-1.528	2.08E-12	3.44E-11
METTL2A	methyltransferase like 2A	protein_coding	chr17:62423867	3843	6.229188824	3.083550847	-1.1	2.09E-12	3.46E-11
YES1	YES proto-oncogene 1, Src family tyrosine	protein_coding	chr18:721588-8	5100	8.105385914	4.577550109	-0.913	2.14E-12	3.53E-11
TSPAN32	tetraspanin 32	protein_coding	chr11:2301997-	3793	8.775485531	2.671281272	-1.789	2.15E-12	3.55E-11
PPARGC1A	PPARG coactivator 1 alpha	protein_coding	chr4:23755041- 10870		15.38878419	9.930122196	-0.718	2.16E-12	3.55E-11
FBXL18	F-box and leucine rich repeat protein 18	protein_coding	chr7:5431335-5	9753	3.850314724	1.77945933	-1.198	2.19E-12	3.58E-11
RBM24	RNA binding motif protein 24	protein_coding	chr6:17281361-	3425	147.7952772	100.3724133	-0.645	2.18E-12	3.58E-11
UHRF1BP1	UHRF1 binding protein 1	protein_coding	chr6:34792015-	9908	7.346871186	4.276148779	-0.865	2.20E-12	3.59E-11
TPM3	tropomyosin 3	protein_coding	chr1:154155304	15156	4.254979317	2.71048232	-0.736	2.20E-12	3.59E-11
ATAD2B	ATPase family AAA domain containing 2B	protein_coding	chr2:23748664-	10501	3.566155064	1.917471188	-0.981	2.21E-12	3.59E-11
PRKACB	protein kinase cAMP-activated catalytic su	protein_coding	chr1:84078062-	6730	9.820078428	6.376154006	-0.709	2.28E-12	3.70E-11
NAP1L4	nucleosome assembly protein 1 like 4	protein_coding	chr11:2944431-	5200	37.77332626	26.18484986	-0.615	2.31E-12	3.72E-11
PGGT1B	protein geranylgeranyltransferase type I su	protein_coding	chr5:115204012	10069	3.194646537	1.893351793	-0.84	2.31E-12	3.72E-11
RRAGD	Ras related GTP binding D	protein_coding	chr6:89364636-	5072	51.15044621	32.63776021	-0.734	2.32E-12	3.73E-11
TNNT1	troponin T1, slow skeletal type	protein_coding	chr19:55132698	2119	52.41294698	32.87794055	-0.76	2.35E-12	3.76E-11
HMGB3	high mobility group box 3	protein_coding	chrX:150980509	3914	15.32458315	7.816972054	-1.057	2.36E-12	3.77E-11
LPL	lipoprotein lipase	protein_coding	chr8:19901717-	4131	13.81611585	5.165015767	-1.497	2.37E-12	3.79E-11
ZNF181	zinc finger protein 181	protein_coding	chr19:34734155	6266	3.680491467	2.005045968	-0.961	2.38E-12	3.79E-11
IGFBP4	insulin like growth factor binding protein	protein_coding	chr17:40443450	2205	5.641981588	0.91051789	-2.714	2.39E-12	3.80E-11
SSR3	signal sequence receptor subunit 3	protein_coding	chr3:156540140	4627	49.2376665	31.43422427	-0.733	2.41E-12	3.83E-11
B4GAT1	beta-1,4-glucuronyltransferase 1	protein_coding	chr11:66345374	2007	25.9151231	17.05684176	-0.689	2.44E-12	3.86E-11
NIPAL4	NIPA like domain containing 4	protein_coding	chr5:157460019	4208	4.782658417	1.563155398	-1.697	2.43E-12	3.86E-11
MBD2	methyl-CpG binding domain protein 2	protein_coding	chr18:54151606	5686	5.561406799	2.918032543	-1.02	2.45E-12	3.87E-11
C1orf226	chromosome 1 open reading frame 226	protein_coding	chr1:162378841	4313	4.606986773	2.335420955	-1.064	2.46E-12	3.88E-11
PPM1K	protein phosphatase, Mg2+/Mn2+ depenc	protein_coding	chr4:88257620-	11371	12.00247118	7.708712554	-0.725	2.47E-12	3.90E-11
MAPK8	mitogen-activated protein kinase 8	protein_coding	chr10:48306639	9674	3.600542329	2.094003589	-0.87	2.48E-12	3.90E-11
PI4K2A	phosphatidylinositol 4-kinase type 2 alph	protein_coding	chr10:97640686	4189	6.340453254	3.783444028	-0.832	2.49E-12	3.91E-11

NHLRC3	NHL repeat containing 3	protein_coding	chr13:39038306	6409	4.140738549	2.20564415	-0.994	2.51E-12	3.94E-11
DRD1	dopamine receptor D1	protein_coding	chr5:17544003€	4031	12.84013438	7.427570849	-0.874	2.53E-12	3.96E-11
FAM214A	family with sequence similarity 214 memb	protein_coding	chr15:52581317	9832	6.897157635	4.217503717	-0.796	2.55E-12	4.00E-11
UBE2V2	ubiquitin conjugating enzyme E2 V2	protein_coding	chr8:48008415-	6591	4.518398396	2.253993665	-1.089	2.57E-12	4.01E-11
NEBL	nebulette	protein_coding	chr10:20779973	13654	187.314635	118.9619545	-0.741	2.64E-12	4.10E-11
DDX6	DEAD-box helicase 6	protein_coding	chr11:11874776	11462	13.47253593	8.969535895	-0.672	2.67E-12	4.14E-11
PIEZO1	piezo type mechanosensitive ion channel	protein_coding	chr16:88715338	11292	6.333093238	3.23735045	-1.051	2.71E-12	4.19E-11
GNL3L	G protein nucleolar 3 like	protein_coding	chrX:54530211-	8807	6.328080014	3.965875221	-0.76	2.72E-12	4.19E-11
STAM	signal transducing adaptor molecule	protein_coding	chr10:17644151	4130	9.310475643	5.951817388	-0.732	2.71E-12	4.19E-11
TNPO1	transportin 1	protein_coding	chr5:72816312-	11977	16.38522319	10.61537727	-0.712	2.73E-12	4.20E-11
DAB2IP	DAB2 interacting protein	protein_coding	chr9:121567057	9127	14.18156563	8.52154387	-0.82	2.75E-12	4.22E-11
COL21A1	collagen type XXI alpha 1 chain	protein_coding	chr6:56056590-	6912	16.86915206	10.73408932	-0.74	2.79E-12	4.28E-11
CHERP	calcium homeostasis endoplasmic reticul	protein_coding	chr19:16517894	6015	10.04606627	6.260722954	-0.767	2.82E-12	4.31E-11
ADORA1	adenosine A1 receptor	protein_coding	chr1:203090654	4191	14.46969778	9.687439367	-0.666	2.88E-12	4.40E-11
CDK19	cyclin dependent kinase 19	protein_coding	chr6:110609978	7645	2.59641821	1.236037547	-1.153	2.99E-12	4.54E-11
TUBE1	tubulin epsilon 1	protein_coding	chr6:112070663	7087	4.866641305	2.60951627	-0.983	3.02E-12	4.58E-11
TTC28	tetratricopeptide repeat domain 28	protein_coding	chr22:27978014	12645	8.061213103	4.048226076	-1.076	3.10E-12	4.69E-11
GUF1	GUF1 homolog, GTPase	protein_coding	chr4:44678420-	4463	10.50807476	6.444516567	-0.791	3.13E-12	4.72E-11
MIOS	meiosis regulator for oocyte development	protein_coding	chr7:7566875-7	6714	4.408668422	2.619457371	-0.838	3.13E-12	4.72E-11
MPP3	membrane palmitoylated protein 3	protein_coding	chr17:43800799	4491	7.098555492	4.097979168	-0.881	3.17E-12	4.78E-11
HADHA	hydroxyacyl-CoA dehydrogenase trifuncti	protein_coding	chr2:26190635-	4331	62.57274323	43.05476854	-0.625	3.21E-12	4.82E-11
MTR	5-methyltetrahydrofolate-homocysteine r	protein_coding	chr1:236795292	12357	9.830422021	5.643167268	-0.885	3.21E-12	4.82E-11
TMPO	thymopoietin	protein_coding	chr12:98515512	8593	9.694154783	6.063320094	-0.763	3.22E-12	4.82E-11
GFOD1	glucose-fructose oxidoreductase domain c	protein_coding	chr6:13357830-	10686	6.12705169	2.858438809	-1.187	3.24E-12	4.85E-11
AC034102.1	RAB5B, member RAS oncogene family	protein_coding	chr12:55973913	6525	31.72757327	21.99009602	-0.615	3.26E-12	4.85E-11
EPM2A	EPM2A glucan phosphatase, laforin	protein_coding	chr6:145382535	18617	2.085003202	1.214660969	-0.862	3.25E-12	4.85E-11
KCTD20	potassium channel tetramerization doma	protein_coding	chr6:36442767-	6239	15.40222109	8.593331884	-0.926	3.25E-12	4.85E-11
CEBPG	CCAAT enhancer binding protein gamma	protein_coding	chr19:33373685	4056	11.5299116	7.051439165	-0.794	3.27E-12	4.86E-11
TMEM88	transmembrane protein 88	protein_coding	chr17:7855065-	875	7.720153487	1.716515465	-2.245	3.33E-12	4.94E-11
TPD52L1	TPD52 like 1	protein_coding	chr6:125119049	7595	4.15026202	1.78830624	-1.306	3.44E-12	5.09E-11
CACNA2D2	calcium voltage-gated channel auxiliary su	protein_coding	chr3:50362799-	6322	8.997954033	4.104884395	-1.214	3.65E-12	5.34E-11
RANBP6	RAN binding protein 6	protein_coding	chr9:6011043-6	4583	20.17330229	12.89488921	-0.732	3.67E-12	5.37E-11
C4orf46	chromosome 4 open reading frame 46	protein_coding	chr4:158666675	3630	5.282263771	2.639460054	-1.085	3.70E-12	5.40E-11
APOA1	apolipoprotein A1	protein_coding	chr11:11683575	1424	10.44043316	3.287252089	-1.76	3.75E-12	5.46E-11
CLDN6	claudin 6	protein_coding	chr16:3014712-	1739	32.65578082	20.37806953	-0.765	3.76E-12	5.46E-11
MEF2C	myocyte enhancer factor 2C	protein_coding	chr5:88717117-	11755	3.124039963	1.80216042	-0.88	3.76E-12	5.46E-11
RAP1GAP2	RAP1 GTPase activating protein 2	protein_coding	chr17:2755705-	7532	16.79089616	9.019994355	-0.98	3.76E-12	5.46E-11
ZNF330	zinc finger protein 330	protein_coding	chr4:141220887	2536	39.53181291	23.07166632	-0.863	3.81E-12	5.53E-11
PRKN	parkin RBR E3 ubiquitin protein ligase	protein_coding	chr6:161347417	5689	2.568444594	1.062185243	-1.356	3.85E-12	5.58E-11
ARHGAP42	Rho GTPase activating protein 42	protein_coding	chr11:10068728	9313	7.673250006	4.713355043	-0.788	3.88E-12	5.61E-11
FERMT2	fermitin family member 2	protein_coding	chr14:52857268	8463	14.7920495	10.45700189	-0.586	3.89E-12	5.61E-11
CTTN	cortactin	protein_coding	chr11:70398404	6949	22.09776858	14.70431354	-0.673	3.92E-12	5.64E-11
SHB	SH2 domain containing adaptor protein B	protein_coding	chr9:37915898-	6035	4.242972444	2.253428567	-1.003	3.94E-12	5.66E-11
UBE2G2	ubiquitin conjugating enzyme E2 G2	protein_coding	chr21:44768580	7195	20.43270014	14.41355845	-0.59	4.02E-12	5.77E-11
TMEM19	transmembrane protein 19	protein_coding	chr12:71686087	6488	5.842504039	3.18560926	-0.96	4.03E-12	5.78E-11
YPEL3	yippee like 3	protein_coding	chr16:30092314	3181	6.970508115	3.251104064	-1.187	4.12E-12	5.89E-11
PTP4A2	protein tyrosine phosphatase 4A2	protein_coding	chr1:31906421-	6708	24.66075193	17.387888	-0.59	4.19E-12	5.98E-11
PRODH	proline dehydrogenase 1	protein_coding	chr22:18912777	8776	1.962028905	0.922066743	-1.172	4.23E-12	6.02E-11
SMARCA2	SWI/SNF related, matrix associated, actin	protein_coding	chr9:1980290-2	18595	4.481634647	2.562186859	-0.891	4.33E-12	6.17E-11
SYNPO2	synaptopodin 2	protein_coding	chr4:118850688	19530	5.859422693	3.75662932	-0.727	4.37E-12	6.21E-11
ARHGAP27	Rho GTPase activating protein 27	protein_coding	chr17:45393902	10426	2.746624794	1.561713691	-0.902	4.38E-12	6.21E-11
KIAA1522	KIAA1522	protein_coding	chr1:32741830- 6020		4.065305549	1.718111617	-1.327	4.39E-12	6.22E-11
LYPLA1	lysophospholipase 1	protein_coding	chr8:54046367-	4694	34.26290071	19.89215169	-0.868	4.46E-12	6.29E-11
NDUFA1	NADH:ubiquinone oxidoreductase subuni	protein_coding	chrX:119871832	421	236.4089934	141.2636702	-0.829	4.46E-12	6.29E-11
RNF19A	ring finger protein 19A, RBR E3 ubiquitin p	protein_coding	chr8:100257060	5852	13.19010824	8.727878739	-0.682	4.60E-12	6.45E-11
SH3BP4	SH3 domain binding protein 4	protein_coding	chr2:234952017	7085	13.54538392	8.496084517	-0.758	4.74E-12	6.62E-11
CRABP2	cellular retinoic acid binding protein 2	protein_coding	chr1:156699606	1082	18.12210549	8.314368186	-1.213	4.78E-12	6.66E-11
ANKRD13A	ankyrin repeat domain 13A	protein_coding	chr12:10999918	7350	7.824903937	4.939611465	-0.749	4.80E-12	6.67E-11
SLC22A23	solute carrier family 22 member 23	protein_coding	chr6:3268962-3	8898	5.70179875	2.780592799	-1.119	4.79E-12	6.67E-11
RNF44	ring finger protein 44	protein_coding	chr5:176526712	5627	24.3387832	15.38158409	-0.748	4.82E-12	6.69E-11
B4GALNT3	beta-1,4-N-acetyl-galactosaminyltransfera	protein_coding	chr12:460364-5	6867	47.66678645	27.69795879	-0.869	4.83E-12	6.70E-11
ASNSD1	asparagine synthetase domain containing	protein_coding	chr2:189661385	2485	4.432826822	1.581296011	-1.577	4.85E-12	6.71E-11
MPZL1	myelin protein zero like 1	protein_coding	chr1:167721192	7069	4.425387862	2.628951662	-0.836	4.85E-12	6.71E-11
RBPMS2	RNA binding protein, mRNA processing fa	protein_coding	chr15:64739891	2104	113.3742069	73.80710486	-0.706	4.86E-12	6.72E-11
M6PR	mannose-6-phosphate receptor, cation de	protein_coding	chr12:8940361-	3585	20.49279548	12.61281869	-0.786	5.02E-12	6.91E-11
CHD7	chromodomain helicase DNA binding pro	protein_coding	chr8:60678740- 17020		4.920200986	3.137580556	-0.735	5.05E-12	6.94E-11
TACC2	transforming acidic coiled-coil containing	protein_coding	chr10:12198916	18855	19.65852148	13.66203586	-0.611	5.16E-12	7.06E-11
XIRP1	xin actin binding repeat containing 1	protein_coding	chr3:39183210-	6467	178.3568189	88.97023357	-1.086	5.23E-12	7.15E-11
KCNMB4	potassium calcium-activated channel sub	protein_coding	chr12:70366290	4860	8.166593734	4.51499121	-0.939	5.36E-12	7.31E-11
MASTL	microtubule associated serine/threonine	protein_coding	chr10:27154824	3631	4.181290127	2.090325941	-1.083	5.54E-12	7.52E-11
FSCN1	fascin actin-bundling protein 1	protein_coding	chr7:5592816-5	3699	56.85456476	39.09244107	-0.626	5.55E-12	7.53E-11
INKA2-AS1	INKA2 antisense RNA 1	lncRNA	chr1:111739579	2414	3.560459241	0.902196237	-2.073	5.58E-12	7.56E-11
MFF	mitochondrial fission factor	protein_coding	chr2:227325151	6150	11.85991952	6.508407711	-0.95	5.59E-12	7.56E-11
HIPK3	homeodomain interacting protein kinase	protein_coding	chr11:33256672	8164	26.14800345	15.28306709	-0.859	5.95E-12	8.00E-11
NR1D1	nuclear receptor subfamily 1 group D mer	protein_coding	chr17:40092793	2630	4.879613533	2.060383826	-1.323	6.00E-12	8.04E-11
TOM1L2	target of myb1 like 2 membrane traffickin	protein_coding	chr17:17843511	7863	58.71530844	41.02472145	-0.603	6.00E-12	8.04E-11
CTSV	cathepsin V	protein_coding	chr9:97029677-	4534	2.246773124	0.838773139	-1.509	6.03E-12	8.06E-11
DOLK	dolichol kinase	protein_coding	chr9:128945530 2090		12.27311856	6.516312024	-1.004	6.05E-12	8.07E-11
STK40	serine/threonine kinase 40	protein_coding	chr1:36339624-	5590	17.74900611	11.97448106	-0.654	6.07E-12	8.09E-11
FAM3A	family with sequence similarity 3 member	protein_coding	chrX:154506159	5098	19.53801088	13.72086232	-0.596	6.09E-12	8.12E-11
DESI2	desumoylating isopeptidase 2	protein_coding	chr1:244653103	4936	19.00544323	13.75053029	-0.553	6.11E-12	8.13E-11
CDV3	CDV3 homolog	protein_coding	chr3:133573730	4367	40.7487085	28.02877135	-0.626	6.14E-12	8.16E-11
ERC1	ELKS/RAB6-interacting/CAST family memb	protein_coding	chr12:990509-1	13484	8.919418068	5.067107162	-0.899	6.15E-12	8.16E-11
DCUN1D2	defective in cullin neddylation 1 domain c	protein_coding	chr13:11345581	4345	12.85721829	8.472088324	-0.689	6.34E-12	8.40E-11
AL136171.1	disrupted in schizophrenia 2	lncRNA	chr1:231767464	16539	36.22610088	16.37493126	-1.225	6.39E-12	8.44E-11
SELENOT	selenoprotein T	protein_coding	chr3:150602875	4135	33.78412148	23.78283062	-0.592	6.38E-12	8.44E-11
KLHL4	kelch like family member 4	protein_coding	chrX:87517409-	6158	4.716780419	2.75542446	-0.862	6.42E-12	8.47E-11
CCDC51	coiled-coil domain containing 51	protein_coding	chr3:48432164-	2410	5.199848316	2.304939374	-1.262	6.55E-12	8.61E-11
IGSF9	immunoglobulin superfamily member 9	protein_coding	chr1:159927039	6637	8.428046058	4.231949634	-1.077	6.55E-12	8.61E-11
ABCA7	ATP binding cassette subfamily A member	protein_coding	chr19:1040101-	8367	5.015889764	2.616806062	-1.022	6.59E-12	8.64E-11
JADE1	jade family PHD finger 1	protein_coding	chr4:128809700	9052	4.863244138	2.941197874	-0.813	6.59E-12	8.64E-11
MRPL33	mitochondrial ribosomal protein L33	protein_coding	chr2:27771717-	1532	20.14120016	11.03294409	-0.952	6.59E-12	8.64E-11
SELENOF	selenoprotein F	protein_coding	chr1:86862445-	2201	22.80489041	11.17509228	-1.116	6.65E-12	8.71E-11
PRKAA2	protein kinase AMP-activated catalytic sul	protein_coding	chr1:56645314-	9362	17.16613733	11.58402176	-0.653	6.75E-12	8.82E-11
LPP	LIM domain containing preferred transloc	protein_coding	chr3:188153284	25535	7.877432071	3.78926199	-1.137	6.77E-12	8.84E-11
DTNA	dystrobrevin alpha	protein_coding	chr18:34493290	12634	6.953371887	4.656349272	-0.664	6.79E-12	8.85E-11
PPP1R12B	protein phosphatase 1 regulatory subunit	protein_coding	chr1:202348699	21069	77.54261238	47.89031611	-0.781	6.82E-12	8.85E-11
TSEN54	tRNA splicing endonuclease subunit 54	protein_coding	chr17:75516060	3435	8.423113737	4.621633698	-0.953	6.82E-12	8.85E-11
VGLL4	vestigial like family member 4	protein_coding	chr3:11556069-	7228	12.96117794	8.233489394	-0.739	6.81E-12	8.85E-11
GTF2E1	general transcription factor IIE subunit 1	protein_coding	chr3:120742637	3370	3.509172256	1.67850968	-1.147	6.85E-12	8.88E-11
RAB3GAP1	RAB3 GTPase activating protein catalytic s	protein_coding	chr2:135052265	6629	13.72864746	9.579161775	-0.606	6.89E-12	8.92E-11
PLEKHH1	pleckstrin homology, MyTH4 and FERM do	protein_coding	chr14:67533301	10817	2.76388291	1.51167951	-0.953	6.96E-12	9.00E-11
SUGT1	SGT1 homolog, MIS12 kinetochore compl	protein_coding	chr13:52652709	15102	4.112461835	2.685548932	-0.702	7.00E-12	9.03E-11
DNAJC5	DnaJ heat shock protein family (Hsp40) m	protein_coding	chr20:63895126	5343	16.91690833	10.77983688	-0.736	7.05E-12	9.08E-11
RBSN	rabenosyn, RAB effector	protein_coding	chr3:15070073-	7921	5.056642769	2.743423205	-0.968	7.05E-12	9.08E-11
SLC27A3	solute carrier family 27 member 3	protein_coding	chr1:153774354	5470	1.51753459	0.467753643	-1.785	7.08E-12	9.11E-11
G0S2	G0/G1 switch 2	protein_coding	chr1:209675412	876	8.255777171	2.215423991	-1.979	7.13E-12	9.16E-11
TRIM2	tripartite motif containing 2	protein_coding	chr4:153152342	9425	7.972045147	5.143915877	-0.718	7.21E-12	9.24E-11
YARS	tyrosyl-tRNA synthetase	protein_coding	chr1:32775237-	8212	21.28787932	12.55930502	-0.847	7.31E-12	9.35E-11

ABCA1	ATP binding cassette subfamily A member	protein_coding	chr9:10478100€	11264	3.321822678	1.863641847	-0.918	7.36E-12	9.41E-11
TSC22D1	TSC22 domain family member 1	protein_coding	chr13:4443214З	8586	23.77907769	17.33524934	-0.542	7.42E-12	9.46E-11
ATXN1L	ataxin 1 like	protein_coding	chr16:7184597€	8552	4.840051694	2.243865601	-1.195	7.56E-12	9.59E-11
TMEM116	transmembrane protein 116	protein_coding	chr12:1119312ε	7989	3.78398495	1.992065814	-1.01	7.75E-12	9.81E-11
DCUN1D4	defective in cullin neddylation 1 domain (	protein_coding	chr4:51843000-	7625	8.324910924	5.478000799	-0.689	7.79E-12	9.83E-11
FIZ1	FLT3 interacting zinc finger 1	protein_coding	chr19:5559137€	6785	3.434855283	1.969412237	-0.89	7.82E-12	9.86E-11
ZNF185	zinc finger protein 185 with LIM domain	protein_coding	chrX:15291444Ż	4812	9.612471504	5.049608269	-1.014	7.89E-12	9.91E-11
DEPDC1	DEP domain containing 1	protein_coding	chr1:68474152-	5904	5.583068455	3.115586695	-0.927	7.93E-12	9.95E-11
LGALS3	galectin 3	protein_coding	chr14:5512411(	2199	22.83613389	14.81922029	-0.711	7.97E-12	9.98E-11
SMPDL3B	sphingomyelin phosphodiesterase acid lik	protein_coding	chr1:27935000-	2663	3.671201436	1.311838085	-1.579	7.98E-12	9.99E-11
ADD3	adducin 3	protein_coding	chr10:1099963€	6541	42.74140601	29.24081343	-0.633	8.02E-12	1.002592
RASD1	ras related dexamethasone induced 1	protein_coding	chr17:1749443	1748	20.23385452	11.20863507	-0.936	8.11E-12	1.012431
PRKCZ	protein kinase C zeta	protein_coding	chr1:2050411-2	7695	4.293282548	2.607255017	-0.808	8.37E-12	1.043967
ST3GAL4	ST3 beta-galactoside alpha-2,3-sialyltrans	protein_coding	chr11:1263556	4465	16.52953864	8.840163349	-0.986	8.47E-12	1.055053
RACGAP1	Rac GTPase activating protein 1	protein_coding	chr12:4997692	4487	5.402950263	3.111314105	-0.881	8.49E-12	1.056278
MGAT5	alpha-1,6-mannosylglycoprotein 6-beta-N	protein_coding	chr2:13411998	8765	55.44548245	34.38185862	-0.775	8.75E-12	1.086368
GLP1R	glucagon like peptide 1 receptor	protein_coding	chr6:39048781-	6682	2.12897705	0.32401521	-2.799	8.78E-12	1.089164
RAD1	RAD1 checkpoint DNA exonuclease	protein_coding	chr5:34905260-	6770	7.372675994	4.800292505	-0.707	8.87E-12	1.099026
MIGA2	mitoguardin 2	protein_coding	chr9:12903662	6302	16.8627887	9.267743183	-0.95	8.91E-12	1.103379
AK1	adenylate kinase 1	protein_coding	chr9:12786648	2593	11.40497502	6.990779383	-0.794	8.94E-12	1.106317
BMP2K	BMP2 inducible kinase	protein_coding	chr4:78776342-	9938	9.504158139	6.202553606	-0.701	8.98E-12	1.108821
TMC6	transmembrane channel like 6	protein_coding	chr17:7811045	8669	20.6477494	11.4416054	-0.936	8.98E-12	1.108821
ATP1A1	ATPase Na+/K+ transporting subunit alpha	protein_coding	chr1:11637266	7277	119.6931486	87.06570867	-0.545	9.18E-12	1.130522
TCF7L2	transcription factor 7 like 2	protein_coding	chr10:1129502	6739	3.748326588	2.019494243	-0.979	9.22E-12	1.134847
FUCA2	alpha-L-fucosidase 2	protein_coding	chr6:14349481	2793	17.48625278	10.12093249	-0.877	9.48E-12	1.164330
ADCY6	adenylate cyclase 6	protein_coding	chr12:4876619	8410	36.46131299	18.74316336	-1.042	9.55E-12	1.171475
GORASP1	golgi reassembly stacking protein 1	protein_coding	chr3:39096659-	6256	11.48043065	7.735429463	-0.655	9.60E-12	1.176300
ELK4	ETS transcription factor ELK4	protein_coding	chr1:20559755	12142	3.342435698	1.896697939	-0.904	9.72E-12	1.190509
CCNI	cyclin I	protein_coding	chr4:77047155-	3728	68.97035561	48.61205207	-0.591	9.78E-12	1.195956
GM2A	GM2 ganglioside activator	protein_coding	chr5:15121215	4479	28.01848594	17.28152733	-0.782	9.89E-12	1.208866
STK39	serine/threonine kinase 39	protein_coding	chr2:16795402	3701	87.09277794	63.16995614	-0.549	9.93E-12	1.211562
CHN1	chimerin 1	protein_coding	chr2:17479931	8898	3.917367771	2.303803278	-0.851	1.00E-11	1.221447
FLII	FLII actin remodeling protein	protein_coding	chr17:1824481	6308	37.8471511	27.04378432	-0.571	1.03E-11	1.248743
GALNT17	polypeptide N-acetylgalactosaminyltrans	protein_coding	chr7:71132144-	4895	8.222905117	4.398291835	-0.988	1.03E-11	1.256593
ACOT9	acyl-CoA thioesterase 9	protein_coding	chrX:23701055-	6244	9.650619894	6.521720036	-0.653	1.04E-11	1.265342
MRPS14	mitochondrial ribosomal protein S14	protein_coding	chr1:17501078	2594	8.902338858	5.090303885	-0.894	1.04E-11	1.265342
RANBP10	RAN binding protein 10	protein_coding	chr16:6772307	6456	7.947786618	5.041643602	-0.741	1.05E-11	1.269791
SLC25A46	solute carrier family 25 member 46	protein_coding	chr5:11073813	8270	12.30064497	8.512158542	-0.618	1.06E-11	1.285964
VTN	vitronectin	protein_coding	chr17:2836728	1904	2.880190018	0.237602197	-3.656	1.07E-11	1.289152
HMG20B	high mobility group 20B	protein_coding	chr19:3572777-	3857	20.79234638	14.57075076	-0.599	1.07E-11	1.292316
LAMB1	laminin subunit beta 1	protein_coding	chr7:10792379	8072	65.82739798	43.24161438	-0.692	1.07E-11	1.294781
RAB23	RAB23, member RAS oncogene family	protein_coding	chr6:57186992-	4919	3.075232261	1.519448202	-1.107	1.08E-11	1.301735
LANCL1	LanC like 1	protein_coding	chr2:21043124	5254	29.78824908	21.20326282	-0.577	1.13E-11	1.352518
FGD6	FYVE, RhoGEF and PH domain containing	protein_coding	chr12:9507674	10044	8.428290751	5.232649493	-0.772	1.14E-11	1.355145
PIGN	phosphatidylinositol glycan anchor biosy	protein_coding	chr18:6190525	19003	1.568904889	0.930066	-0.839	1.13E-11	1.355145
TMEM245	transmembrane protein 245	protein_coding	chr9:10901513	9503	43.70243549	29.44326371	-0.655	1.13E-11	1.355145
ANKRD29	ankyrin repeat domain 29	protein_coding	chr18:2359892	6417	1.519029141	0.579847819	-1.469	1.15E-11	1.371710
RASGRP2	RAS guanyl releasing protein 2	protein_coding	chr11:6472691	6084	5.604400636	3.121255665	-0.929	1.16E-11	1.380313
EPHA7	EPH receptor A7	protein_coding	chr6:93240020-	7468	12.77583395	9.25132644	-0.552	1.16E-11	1.385780
FADS2	fatty acid desaturase 2	protein_coding	chr11:6179298	10553	9.807189544	6.528766996	-0.673	1.17E-11	1.385780
SNX24	sorting nexin 24	protein_coding	chr5:12284343	3474	10.94743057	5.667265771	-1.033	1.17E-11	1.385780
AC097359.2	novel transcript, antisense to GOLGA4	lncRNA	chr3:37241789-	2389	3.878655125	1.532455341	-1.419	1.18E-11	1.403543
RNF145	ring finger protein 145	protein_coding	chr5:15915740	4614	14.34465379	9.935605677	-0.616	1.19E-11	1.412428
C1orf105	chromosome 1 open reading frame 105	protein_coding	chr1:17242068	1728	13.17331457	4.631362269	-1.591	1.22E-11	1.442815
SLC16A6	solute carrier family 16 member 6	protein_coding	chr17:6826702	4061	1.316751794	0.366296858	-1.924	1.23E-11	1.450858
ZNF814	zinc finger protein 814	protein_coding	chr19:5784873	13421	1.214933457	0.536656026	-1.259	1.24E-11	1.465080
NAPA	NSF attachment protein alpha	protein_coding	chr19:4748763	5690	12.28628152	8.42515525	-0.631	1.24E-11	1.468021
TRPC4AP	transient receptor potential cation chann	protein_coding	chr20:3500240	3162	27.49452179	18.28360702	-0.674	1.25E-11	1.470368
CAVIN3	caveolae associated protein 3	protein_coding	chr11:6318946-	1557	20.14857341	10.87823305	-0.974	1.26E-11	1.485562
DAPK1	death associated protein kinase 1	protein_coding	chr9:87497228-	8908	5.466975041	3.429974954	-0.758	1.27E-11	1.486378
PPARGC1B	PPARG coactivator 1 beta	protein_coding	chr5:14973029	12889	6.486277438	4.127329083	-0.737	1.29E-11	1.511072
ZNF367	zinc finger protein 367	protein_coding	chr9:96385941-	3687	4.93283501	2.306678626	-1.176	1.29E-11	1.511072
P2RX1	purinergic receptor P2X 1	protein_coding	chr17:3896592-	3946	9.260213169	4.11119268	-1.257	1.32E-11	1.535010
CRYBG3	crystallin beta-gamma domain containing	protein_coding	chr3:97822011-	10979	11.58146232	6.139317081	-0.999	1.36E-11	1.571199
SYNPO2L	synaptopodin 2 like	protein_coding	chr10:7364488	5104	279.2158971	191.560539	-0.63	1.37E-11	1.590739
EFCAB2	EF-hand calcium binding domain 2	protein_coding	chr1:24496970	8607	2.541880443	1.238964723	-1.126	1.39E-11	1.608081
SLC43A2	solute carrier family 43 member 2	protein_coding	chr17:1569267-	10606	1.575831451	0.593194498	-1.495	1.40E-11	1.618144
NCK2	NCK adaptor protein 2	protein_coding	chr2:10574491	2975	17.9483385	12.42867342	-0.616	1.47E-11	1.686676
GRID1	glutamate ionotropic receptor delta type	protein_coding	chr10:8559955	6232	4.282509058	1.543356058	-1.552	1.48E-11	1.696687
CDK1	cyclin dependent kinase 1	protein_coding	chr10:6077833	3931	6.524526396	3.665675571	-0.917	1.48E-11	1.704609
KTN1	kinectin 1	protein_coding	chr14:5555907	17578	26.046439	18.7032332	-0.564	1.49E-11	1.706940
AGPS	alkylglycerone phosphate synthase	protein_coding	chr2:17739276	8839	9.605367741	6.981595744	-0.546	1.50E-11	1.712643
MSS51	MSS51 mitochondrial translational activa	protein_coding	chr10:7342357	2423	7.358676922	2.137799957	-1.869	1.51E-11	1.721488
RGL2	ral guanine nucleotide dissociation stimu	protein_coding	chr6:33291654-	4159	13.84743442	8.658575448	-0.763	1.53E-11	1.740814
VASN	vasorin	protein_coding	chr16:4371848-	2816	6.713971574	3.797124924	-0.908	1.55E-11	1.763145
IPO9	importin 9	protein_coding	chr1:20182914	11904	10.8266605	7.56523369	-0.603	1.56E-11	1.777296
DNAJC14	DnaJ heat shock protein family (Hsp40) m	protein_coding	chr12:5582096	4192	4.603917291	2.651041925	-0.88	1.57E-11	1.782351
FAM8A1	family with sequence similarity 8 member	protein_coding	chr6:17600302-	4726	11.23750791	7.180030348	-0.733	1.57E-11	1.787100
FEM1C	fem-1 homolog C	protein_coding	chr5:11552090	5697	7.081897184	4.420185781	-0.766	1.58E-11	1.787100
EAF1	ELL associated factor 1	protein_coding	chr3:15427598-	4909	3.067089388	1.533231606	-1.084	1.58E-11	1.787111
AMER1	APC membrane recruitment protein 1	protein_coding	chrX:64185117-	8407	4.924315334	2.842216769	-0.876	1.59E-11	1.799972
GPR1	G protein-coupled receptor 1	protein_coding	chr2:20617531	2834	3.93990699	1.426846748	-1.552	1.61E-11	1.808239
RALGAPA1	Ral GTPase activating protein catalytic alp	protein_coding	chr14:3553835	14258	2.839411712	1.4776572	-1.026	1.61E-11	1.817136
APOL2	apolipoprotein L2	protein_coding	chr22:3622620	3417	11.89913973	5.750805825	-1.129	1.65E-11	1.853868
MEGF6	multiple EGF like domains 6	protein_coding	chr1:3487951-3	9021	3.53103836	1.064978795	-1.816	1.66E-11	1.862932
WWC2	WW and C2 domain containing 2	protein_coding	chr4:18309925	10002	9.397032075	5.857010085	-0.767	1.67E-11	1.871759
NEURL1B	neuralized E3 ubiquitin protein ligase 1B	protein_coding	chr5:17264126	6424	9.172550225	4.46140579	-1.123	1.68E-11	1.877217
RBBP7	RB binding protein 7, chromatin remodel	protein_coding	chrX:16839283-	7392	35.68815091	26.21492229	-0.531	1.68E-11	1.880780
LMOD2	leiomodin 2	protein_coding	chr7:12365586	2672	110.5598602	72.29524814	-0.699	1.73E-11	1.931939
CASP3	caspase 3	protein_coding	chr4:18462769	2899	18.00099791	12.11678967	-0.658	1.74E-11	1.939513
FAM20A	FAM20A golgi associated secretory pathw	protein_coding	chr17:6853511	6181	2.717395081	1.221762544	-1.25	1.75E-11	1.948084
SARAF	store-operated calcium entry associated r	protein_coding	chr8:30063012-	2990	55.66245285	38.76870173	-0.607	1.76E-11	1.950039
ZNF503	zinc finger protein 503	protein_coding	chr10:7539783	3205	4.6341457	2.452657084	-1.002	1.76E-11	1.956954
PLEKHA7	pleckstrin homology domain containing A	protein_coding	chr11:1677729	10978	42.16642646	26.61644815	-0.748	1.77E-11	1.961904
GPR107	G protein-coupled receptor 107	protein_coding	chr9:13005342	9139	9.52979454	5.958076605	-0.762	1.80E-11	1.990486
MYO1C	myosin IC	protein_coding	chr17:1464186-	7806	33.23265423	23.44850091	-0.589	1.80E-11	1.993766
NADK	NAD kinase	protein_coding	chr1:1751232-1	5570	7.56464145	4.651181408	-0.789	1.81E-11	1.995892
ATP6V1D	ATPase H+ transporting V1 subunit D	protein_coding	chr14:6729437	4247	23.35351368	14.59804145	-0.765	1.81E-11	1.996818
CCSER2	coiled-coil serine rich protein 2	protein_coding	chr10:8643285	8424	25.2378162	18.60487656	-0.526	1.84E-11	2.023333
SYNJ2	synaptojanin 2	protein_coding	chr6:15798186	12517	3.297258333	1.776801725	-0.978	1.88E-11	2.060081
FAM155B	family with sequence similarity 155 mem	protein_coding	chrX:69505241-	4013	1.81566643	0.67462151	-1.517	1.93E-11	2.105465
CMTM3	CKLF like MARVEL transmembrane domai	protein_coding	chr16:6660387	3123	8.802114261	5.449091882	-0.776	1.94E-11	2.109860
KARS	lysyl-tRNA synthetase	protein_coding	chr16:7562747	4065	26.85307616	17.02969779	-0.742	1.94E-11	2.109860
FASTKD5	FAST kinase domains 5	protein_coding	chr20:3146519-	2842	8.654410045	4.919009207	-0.9	2.07E-11	2.239673
CLTA	clathrin light chain A	protein_coding	chr9:36190856-	2515	25.02755663	15.51145098	-0.775	2.15E-11	2.319901
LGALSL	galectin like	protein_coding	chr2:64453969-	3933	4.991637998	1.613313042	-1.709	2.18E-11	2.348544
LLGL2	LLGL scribble cell polarity complex comp	protein_coding	chr17:7552508	7909	5.57926164	3.623323418	-0.708	2.19E-11	2.355829
EFNB3	ephrin B3	protein_coding	chr17:7705202-	3216	22.61574958	11.21722505	-1.094	2.21E-11	2.376241
KDELR2	KDEL endoplasmic reticulum protein rete	protein_coding	chr7:6445953-6	3930	25.27046578	17.79271112	-0.592	2.22E-11	2.390825

YIPF5	Yip1 domain family member 5	protein_coding	chr5:14415816	3698	12.27673819	8.120482652	-0.683	2.23E-11	2.391491
PLEKHG5	pleckstrin homology and RhoGEF domain	protein_coding	chr1:6466092-6	7053	4.905062883	2.807511574	-0.887	2.24E-11	2.400185
TMEM87A	transmembrane protein 87A	protein_coding	chr15:4221045	5668	4.989612935	2.964603924	-0.837	2.24E-11	2.400185
UBE2H	ubiquitin conjugating enzyme E2 H	protein_coding	chr7:12983073	6503	65.28246508	39.92291823	-0.793	2.25E-11	2.401590
MAGI1	membrane associated guanylate kinase, W	protein_coding	chr3:65353525-	11649	10.80022186	6.75563073	-0.761	2.26E-11	2.416132
PJA1	praja ring finger ubiquitin ligase 1	protein_coding	chrX:69160851-	3117	9.482830978	5.701341838	-0.821	2.27E-11	2.424227
UBXN2A	UBX domain protein 2A	protein_coding	chr2:23927285-	6838	5.824348039	3.920288245	-0.657	2.28E-11	2.424227
LETM1	leucine zipper and EF-hand containing tra	protein_coding	chr4:1811479-1	8093	11.40843729	7.489010199	-0.692	2.33E-11	2.472816
DBT	dihydrolipoamide branched chain transac	protein_coding	chr1:10018691	11303	5.399874356	3.58280999	-0.677	2.40E-11	2.539417
CD63	CD63 molecule	protein_coding	chr12:5572532	2633	96.95661619	63.4315933	-0.698	2.40E-11	2.539904
PTK2	protein tyrosine kinase 2	protein_coding	chr8:14065790	13742	6.335789402	4.579055602	-0.554	2.44E-11	2.569611
KIRREL1	kirre like nephrin family adhesion molecu	protein_coding	chr1:15799327	7872	7.12432317	3.940896306	-0.938	2.50E-11	2.628536
LYSMD1	LysM domain containing 1	protein_coding	chr1:15115974	2508	7.148827147	4.150715516	-0.869	2.53E-11	2.658921
GCOM1	GRINL1A complex locus 1	protein_coding	chr15:5759190	10302	4.123797125	2.572547802	-0.766	2.55E-11	2.671489
C7	complement C7	protein_coding	chr5:40909252-	5223	8.153076737	4.803392273	-0.848	2.61E-11	2.734088
ZNF275	zinc finger protein 275	protein_coding	chrX:15333414	8147	3.188659724	1.754822497	-0.947	2.62E-11	2.738193
ATP6V1E1	ATPase H+ transporting V1 subunit E1	protein_coding	chr22:1759213	3143	50.30107761	31.051272	-0.779	2.68E-11	2.786489
SLAIN2	SLAIN motif family member 2	protein_coding	chr4:48341529-	6306	12.42688879	8.916100672	-0.565	2.68E-11	2.786489
SCD5	stearoyl-CoA desaturase 5	protein_coding	chr4:82629539-	4231	1.761457482	0.725676963	-1.359	2.71E-11	2.816498
BCL2L12	BCL2 like 12	protein_coding	chr19:4966514	2216	8.299785796	4.738796192	-0.893	2.72E-11	2.818869
N4BP3	NEDD4 binding protein 3	protein_coding	chr5:17811353	5985	4.201096906	1.582985274	-1.484	2.73E-11	2.835834
ARHGEF16	Rho guanine nucleotide exchange factor 1	protein_coding	chr1:3454665-3	5324	1.545771563	0.348403424	-2.246	2.76E-11	2.864192
SLC41A1	solute carrier family 41 member 1	protein_coding	chr1:20578909	6378	23.91485958	15.56159915	-0.704	2.85E-11	2.940545
CRTC3	CREB regulated transcription coactivator	protein_coding	chr15:9052992	10816	5.142360291	3.061688121	-0.831	2.87E-11	2.955380
CST3	cystatin C	protein_coding	chr20:2362670	3615	40.63007436	24.84558381	-0.794	2.93E-11	3.010742
TENT5B	terminal nucleotidyltransferase 5B	protein_coding	chr1:27005020-	2382	9.806860466	3.242125704	-1.684	2.93E-11	3.010742
INTS2	integrator complex subunit 2	protein_coding	chr17:6186536	8627	2.437166371	1.487337201	-0.797	2.95E-11	3.022754
SLC35B4	solute carrier family 35 member B4	protein_coding	chr7:13428933	6988	2.608443573	1.538081041	-0.848	2.95E-11	3.022754
MSI2	musashi RNA binding protein 2	protein_coding	chr17:5725585	21494	4.824723067	3.210973107	-0.672	2.98E-11	3.045760
MYL2	myosin light chain 2	protein_coding	chr12:1109108	1417	1448.091054	757.0821838	-1.019	3.01E-11	3.080181
NXPH2	neurexophilin 2	protein_coding	chr2:13866915	2709	3.547979543	1.377788702	-1.451	3.02E-11	3.086103
CYP2J2	cytochrome P450 family 2 subfamily J me	protein_coding	chr1:59893308-	2973	67.46478411	49.99842274	-0.519	3.07E-11	3.117573
PEX19	peroxisomal biogenesis factor 19	protein_coding	chr1:16027681	4461	12.14513025	8.216787613	-0.649	3.06E-11	3.117573
ECE1	endothelin converting enzyme 1	protein_coding	chr1:21217247-	7308	8.2785342	5.540276563	-0.664	3.08E-11	3.124126
NAV1	neuron navigator 1	protein_coding	chr1:20162288	22453	52.18742147	26.06672879	-1.082	3.14E-11	3.172184
ARNT	aryl hydrocarbon receptor nuclear translo	protein_coding	chr1:15080971	6448	6.652079963	4.277200323	-0.723	3.18E-11	3.209853
DHCR24	24-dehydrocholesterol reductase	protein_coding	chr1:54849627-	6214	15.44726152	10.12121269	-0.695	3.19E-11	3.222227
LRRC49	leucine rich repeat containing 49	protein_coding	chr15:7085323	9991	14.54833027	9.136915754	-0.758	3.23E-11	3.256833
CLCN5	chloride voltage-gated channel 5	protein_coding	chrX:49922596-	11457	1.475289735	0.796808513	-0.973	3.25E-11	3.277190
RSPH3	radial spoke head 3	protein_coding	chr6:15897287	6561	5.132299831	3.236837077	-0.754	3.28E-11	3.306437
PLCG1	phospholipase C gamma 1	protein_coding	chr20:4113696	9648	8.580247618	5.417300352	-0.747	3.35E-11	3.361920
RB1CC1	RB1 inducible coiled-coil 1	protein_coding	chr8:52622458-	7613	15.26303123	11.41372184	-0.505	3.43E-11	3.433189
MAP1LC3A	microtubule associated protein 1 light ch	protein_coding	chr20:3454685	1152	24.80081214	14.85311162	-0.825	3.48E-11	3.477062
TRIP6	thyroid hormone receptor interactor 6	protein_coding	chr7:10086738	2553	12.57230916	7.807533011	-0.771	3.52E-11	3.509229
CARS	cysteinyl-tRNA synthetase	protein_coding	chr11:3000922-	10037	3.511393979	2.084762151	-0.836	3.53E-11	3.522199
DTX3	deltex E3 ubiquitin ligase 3	protein_coding	chr12:5760462	4087	7.13341734	4.552070065	-0.733	3.56E-11	3.542808
FADS1	fatty acid desaturase 1	protein_coding	chr11:6179962! 10230		12.94057763	8.463242032	-0.699	3.60E-11	3.572159
SPECC1	sperm antigen with calponin homology ar	protein_coding	chr17:2000934	13676	1.80919222	1.083358559	-0.822	3.60E-11	3.574771
PLEKHM3	pleckstrin homology domain containing M	protein_coding	chr2:20782128	11638	2.14472598	1.094348689	-1.054	3.65E-11	3.611443
HECTD2	HECT domain E3 ubiquitin protein ligase 2	protein_coding	chr10:9140928	11550	3.88666995	2.268394161	-0.86	3.73E-11	3.684939
RBCK1	RANBP2-type and C3HC4-type zinc finger	protein_coding	chr20:407498-4	4024	21.71255216	15.42521602	-0.58	3.73E-11	3.684939
KCTD21	potassium channel tetramerization doma	protein_coding	chr11:7817124	3968	4.002408841	2.269885596	-0.908	3.78E-11	3.718757
BTBD3	BTB domain containing 3	protein_coding	chr20:1189072	6228	4.770072577	2.929866631	-0.789	3.81E-11	3.751781
CHSY1	chondroitin sulfate synthase 1	protein_coding	chr15:1011757	5667	3.714539788	2.171459978	-0.859	3.82E-11	3.752812
TNKS	tankyrase	protein_coding	chr8:9555912-9	14188	4.533661118	3.062261379	-0.651	3.83E-11	3.763242
MINDY2	MINDY lysine 48 deubiquitinase 2	protein_coding	chr15:5877119	9454	6.478076894	4.332723036	-0.667	3.88E-11	3.803226
STC2	stanniocalcin 2	protein_coding	chr5:17331471	6128	2.966252705	1.272423483	-1.311	3.94E-11	3.857872
DCAF12	DDB1 and CUL4 associated factor 12	protein_coding	chr9:34086387-	4315	9.755364394	6.784037105	-0.611	4.08E-11	3.984281
STARD10	StAR related lipid transfer domain contai	protein_coding	chr11:7275472	3710	5.574571093	2.661537107	-1.158	4.08E-11	3.984281
KLF13	Kruppel like factor 13	protein_coding	chr15:3132683	9429	19.28898541	11.26814197	-0.858	4.21E-11	4.089094
MFN2	mitofusin 2	protein_coding	chr1:11980181-	4973	126.0370458	90.61235889	-0.562	4.22E-11	4.096803
SHMT2	serine hydroxymethyltransferase 2	protein_coding	chr12:5722957	4592	14.26646394	9.050663453	-0.742	4.31E-11	4.167804
AC018647.1	novel transcript	lncRNA	chr7:35715032-	6724	2.832814175	1.334489137	-1.183	4.31E-11	4.169828
CAVIN2	caveolae associated protein 2	protein_coding	chr2:19183431	3054	1.527934475	0.177594662	-3.203	4.39E-11	4.237015
HAGHL	hydroxyacylglutathione hydrolase like	protein_coding	chr16:726936-7	5287	6.83071871	3.763374787	-0.942	4.42E-11	4.267461
ISG20L2	interferon stimulated exonuclease gene 2	protein_coding	chr1:15672189	5262	4.787846352	3.091295743	-0.717	4.43E-11	4.269083
LSR	lipolysis stimulated lipoprotein receptor	protein_coding	chr19:3524833	2916	6.867234105	4.207862127	-0.794	4.57E-11	4.394362
ATG4A	autophagy related 4A cysteine peptidase	protein_coding	chrX:10809166	2927	8.949158178	5.615898161	-0.759	4.59E-11	4.409600
VANGL1	VANGL planar cell polarity protein 1	protein_coding	chr1:11564197	9045	3.043983603	1.869337344	-0.789	4.66E-11	4.462117
SLC30A1	solute carrier family 30 member 1	protein_coding	chr1:21157156	5893	10.71691442	7.503496428	-0.602	4.67E-11	4.468260
CHMP4B	charged multivesicular body protein 4B	protein_coding	chr20:3381134	1602	83.70229604	58.33030899	-0.608	4.68E-11	4.476573
RNF144A	ring finger protein 144A	protein_coding	chr2:6917412-7	6450	4.288816419	2.316623229	-0.972	4.73E-11	4.510997
HIF1A	hypoxia inducible factor 1 subunit alpha	protein_coding	chr14:6169551	5151	56.04622348	41.20221842	-0.53	4.74E-11	4.513962
DCLK2	doublecortin like kinase 2	protein_coding	chr4:15007844	4416	20.71573478	15.06747466	-0.545	4.75E-11	4.520852
JUN	Jun proto-oncogene, AP-1 transcription fa	protein_coding	chr1:58780788-	3540	46.19423669	33.00244911	-0.571	4.77E-11	4.531162
POMT2	protein O-mannosyltransferase 2	protein_coding	chr14:7727495	10008	4.145665703	2.798578537	-0.653	4.84E-11	4.586667
SASH1	SAM and SH3 domain containing 1	protein_coding	chr6:14827230	10896	2.677720529	1.467349487	-0.951	4.86E-11	4.604328
ADAMTS15	ADAM metallopeptidase with thrombospo	protein_coding	chr11:1304489	5673	12.55355382	6.572525256	-1.017	4.91E-11	4.629719
DGCR6	DiGeorge syndrome critical region gene 6	protein_coding	chr22:1890602	5165	4.912142481	3.029973305	-0.784	5.00E-11	4.719571
GLRB	glycine receptor beta	protein_coding	chr4:15707612	3391	4.405437943	2.516799805	-0.898	5.02E-11	4.733403
PVR	PVR cell adhesion molecule	protein_coding	chr19:4464379	6098	9.113775464	6.372473993	-0.602	5.04E-11	4.741244
NSUN4	NOP2/Sun RNA methyltransferase 4	protein_coding	chr1:46340177-	8468	2.940193192	1.886919456	-0.727	5.08E-11	4.778195
KLHL28	kelch like family member 28	protein_coding	chr14:4492431	8076	3.036196249	1.840492487	-0.806	5.09E-11	4.787323
KLHDC10	kelch domain containing 10	protein_coding	chr7:13007053	6583	7.931090115	5.57951781	-0.593	5.10E-11	4.789258
FGFRL1	fibroblast growth factor receptor like 1	protein_coding	chr4:1009936-1	4342	9.934087187	6.024701706	-0.806	5.13E-11	4.804675
STK17B	serine/threonine kinase 17b	protein_coding	chr2:19613358	6566	2.748041943	1.580939541	-0.884	5.19E-11	4.857910
ATPAF1	ATP synthase mitochondrial F1 complex a	protein_coding	chr1:46632737-	5677	33.75442893	24.66544721	-0.539	5.30E-11	4.948635
BRCA1	BRCA1 DNA repair associated	protein_coding	chr17:4304429	9306	1.369758701	0.731470282	-0.991	5.32E-11	4.967641
IP6K1	inositol hexakisphosphate kinase 1	protein_coding	chr3:49724294-	5200	8.105054957	5.676172804	-0.599	5.34E-11	4.977633
TMEM72	transmembrane protein 72	protein_coding	chr10:4491131	3049	1.178032859	0.238097298	-2.379	5.34E-11	4.977633
TINAGL1	tubulointerstitial nephritis antigen like 1	protein_coding	chr1:31576485-	5281	7.669990185	4.456626084	-0.866	5.48E-11	5.079574
ZMYND8	zinc finger MYND-type containing 8	protein_coding	chr20:4720921	7625	8.713220632	6.275810065	-0.559	5.50E-11	5.091653
GNAL	G protein subunit alpha L	protein_coding	chr18:1168926	8137	1.316707978	0.499564985	-1.487	5.54E-11	5.120208
SLC3A2	solute carrier family 3 member 2	protein_coding	chr11:6285610	5178	11.29426392	7.746331116	-0.629	5.69E-11	5.248824
FRYL	FRY like transcription coactivator	protein_coding	chr4:48497361-	18161	3.72205817	2.042515987	-0.948	5.70E-11	5.259805
PCNX1	pecanex 1	protein_coding	chr14:7090740	16715	6.55120399	4.060197688	-0.774	5.74E-11	5.278289
DTD2	D-aminoacyl-tRNA deacylase 2	protein_coding	chr14:3144603	2967	6.930736043	4.422570351	-0.734	5.79E-11	5.314090
ITGB1	integrin subunit beta 1	protein_coding	chr10:3290031	6012	101.3993937	76.41513135	-0.494	5.79E-11	5.314090
GPC4	glypican 4	protein_coding	chrX:13330010	4959	3.244236623	1.823842923	-0.918	5.84E-11	5.344502
MYZAP	myocardial zonula adherens protein	protein_coding	chr15:5759190	2598	22.93319689	15.96437539	-0.609	5.85E-11	5.349634
BRD8	bromodomain containing 8	protein_coding	chr5:13813976	7046	7.037219071	4.604031808	-0.696	5.87E-11	5.364658
DCP2	decapping mRNA 2	protein_coding	chr5:11297670	11176	2.329962532	1.368392892	-0.86	5.95E-11	5.420384
SLC6A6	solute carrier family 6 member 6	protein_coding	chr3:14402576-	8254	1.086630135	0.44986771	-1.352	5.95E-11	5.420384
RAB1A	RAB1A, member RAS oncogene family	protein_coding	chr2:65070696-	3313	48.26159383	34.5538842	-0.568	5.98E-11	5.442873
INTS6L	integrator complex subunit 6 like	protein_coding	chrX:13552064	7193	10.92653985	7.869814315	-0.559	6.06E-11	5.502000
ADGRL3	adhesion G protein-coupled receptor L3	protein_coding	chr4:61201258-	13991	5.832004791	3.79643013	-0.705	6.17E-11	5.584276
IQCJ-SCHIP1	IQCJ-SCHIP1 readthrough	protein_coding	chr3:15896223	4409	3.302799604	1.594086752	-1.142	6.20E-11	5.602563
YWHAQ	tyrosine 3-monooxygenase/tryptophan 5-	protein_coding	chr2:9583967-9	2701	137.7692372	98.07039988	-0.576	6.21E-11	5.605744
LINC01844	long intergenic non-protein coding RNA 1	lncRNA	chr5:14271622	3601	12.04746489	7.962203147	-0.685	6.25E-11	5.628505

NAAA	N-acylethanolamine acid amidase	protein_coding	chr4:75913660-	4416	6.544926585	3.847878842	-0.847	6.25E-11	5.628505
KCNQ1	potassium voltage-gated channel subfami	protein_coding	chr11:2444684-	4447	18.72848526	13.07213764	-0.605	6.27E-11	5.645702
SAFB2	scaffold attachment factor B2	protein_coding	chr19:5586999-	6018	12.11027223	8.599478235	-0.579	6.28E-11	5.647472
TMEM198	transmembrane protein 198	protein_coding	chr2:21954366	2578	3.244671568	1.303921447	-1.397	6.36E-11	5.712200
RNF123	ring finger protein 123	protein_coding	chr3:49689538-	7419	15.41553743	10.22993813	-0.676	6.45E-11	5.785232
VPS52	VPS52 subunit of GARP complex	protein_coding	chr6:33250272-	4493	11.78786706	7.907853606	-0.661	6.46E-11	5.791093
ABTB2	ankyrin repeat and BTB domain containin	protein_coding	chr11:34150987	4976	15.45463975	9.443237986	-0.795	6.48E-11	5.802298
WDR48	WD repeat domain 48	protein_coding	chr3:39052013-	7473	5.326876425	3.44659801	-0.717	6.53E-11	5.841635
DHX15	DEAH-box helicase 15	protein_coding	chr4:24517441-	8166	14.07218132	10.43130926	-0.518	6.55E-11	5.858783
CBWD2	COBW domain containing 2	protein_coding	chr2:113437691	8396	3.846442036	2.368317141	-0.787	6.58E-11	5.877261
MAP2K3	mitogen-activated protein kinase kinase 3	protein_coding	chr17:21284672	3783	12.67182817	9.015772868	-0.576	6.62E-11	5.902020
PPP6C	protein phosphatase 6 catalytic subunit	protein_coding	chr9:12514657	4443	12.27325251	8.662739494	-0.59	6.66E-11	5.931861
RAP1B	RAP1B, member of RAS oncogene family	protein_coding	chr12:68610855	16926	1.390353702	0.773755512	-0.933	6.72E-11	5.973508
AURKA	aurora kinase A	protein_coding	chr20:56369389	2928	12.97647928	8.828374035	-0.641	6.74E-11	5.989240
PPIA	peptidylprolyl isomerase A	protein_coding	chr7:44796680-	4432	77.40403491	49.35790598	-0.733	6.77E-11	6.012093
NUP62	nucleoporin 62	protein_coding	chr19:49906825 6020		8.135836099	4.934266119	-0.806	6.80E-11	6.035860
TMEM38A	transmembrane protein 38A	protein_coding	chr19:16661139	2987	46.70059952	34.91690893	-0.506	7.06E-11	6.252666
SNAPIN	SNAP associated protein	protein_coding	chr1:15365865	1544	24.35369073	15.62248591	-0.724	7.09E-11	6.273368
PDLIM5	PDZ and LIM domain 5	protein_coding	chr4:94451857- 11090		80.1620261	59.5791704	-0.514	7.13E-11	6.304548
SPTLC3	serine palmitoyltransferase long chain bas	protein_coding	chr20:13008972	8527	1.69705255	1.007186859	-0.84	7.22E-11	6.376274
UBE2N	ubiquitin conjugating enzyme E2 N	protein_coding	chr12:93405673	5954	13.24097301	8.736516252	-0.685	7.25E-11	6.393401
CAPN14	calpain 14	protein_coding	chr2:31173056-	3984	1.590682599	0.491000406	-1.784	7.29E-11	6.421323
MTRR	5-methyltetrahydrofolate-homocysteine	protein_coding	chr5:7851186-7	6498	4.346263206	2.842088446	-0.699	7.31E-11	6.433022
PAPPA	pappalysin 1	protein_coding	chr9:116153791	11585	2.031172387	0.438402357	-2.287	7.45E-11	6.545019
DENND3	DENN domain containing 3	protein_coding	chr8:14111727	14670	1.615693521	0.934340634	-0.87	7.54E-11	6.610230
ADAT1	adenosine deaminase tRNA specific 1	protein_coding	chr16:75596981	4486	4.116305771	2.406138939	-0.856	7.64E-11	6.689977
SERPINB1	serpin family B member 1	protein_coding	chr6:2832332-2	3047	3.22828124	1.59024996	-1.113	7.68E-11	6.716279
AC105940.2	novel transcript	lncRNA	chr1:20311546	3051	1.941067363	0.629743816	-1.696	7.76E-11	6.780959
ZNF720	zinc finger protein 720	protein_coding	chr16:31713229	9816	1.268018067	0.70141415	-0.939	7.81E-11	6.821297
SLC1A4	solute carrier family 1 member 4	protein_coding	chr2:64988477-	6941	5.835097614	3.394022427	-0.867	7.83E-11	6.830795
PRELID3B	PRELI domain containing 3B	protein_coding	chr20:59033145	2877	17.07506876	10.92187735	-0.729	7.91E-11	6.885593
SENP1	SUMO specific peptidase 1	protein_coding	chr12:48042897	6114	3.017619741	1.74854552	-0.876	7.97E-11	6.929150
COQ10B	coenzyme Q10B	protein_coding	chr2:19745342	2722	14.30430241	8.627688386	-0.813	7.97E-11	6.931327
NKX3-1	NK3 homeobox 1	protein_coding	chr8:23678697-	3278	2.580441226	1.150725713	-1.251	8.23E-11	7.141380
SLC4A3	solute carrier family 4 member 3	protein_coding	chr2:21962739	4952	80.88454931	51.16989771	-0.747	8.31E-11	7.200807
CMTR1	cap methyltransferase 1	protein_coding	chr6:37433219-	5276	12.72733698	8.900275647	-0.603	8.44E-11	7.290697
APOLD1	apolipoprotein L domain containing 1	protein_coding	chr12:12725917	7243	0.991120374	0.310695876	-1.751	8.47E-11	7.299130
PDGFD	platelet derived growth factor D	protein_coding	chr11:10390718	4263	10.71667265	6.794407868	-0.74	8.47E-11	7.299130
ZNF3	zinc finger protein 3	protein_coding	chr7:10006403	5424	5.717766792	3.988762739	-0.605	8.52E-11	7.333914
SCARF1	scavenger receptor class F member 1	protein_coding	chr17:1633858-	4958	3.089319624	1.322192482	-1.307	8.55E-11	7.359065
H2AFY	H2A histone family member Y	protein_coding	chr5:13533390	17730	28.58908153	21.36265414	-0.506	8.93E-11	7.641810
PAG1	phosphoprotein membrane anchor with	protein_coding	chr8:80967810-	11023	8.79448164	6.294634067	-0.568	8.92E-11	7.641810
ACTR1A	actin related protein 1A	protein_coding	chr10:10246188	4655	64.38221816	50.26687515	-0.443	9.03E-11	7.721559
GUCD1	guanylyl cyclase domain containing 1	protein_coding	chr22:24540423	4529	26.2445294	19.47272196	-0.516	9.03E-11	7.721559
TOMM34	translocase of outer mitochondrial memb	protein_coding	chr20:44942130	1973	17.11922032	10.58115619	-0.778	9.26E-11	7.887894
AC010327.4	novel transcript, antisense to C19orf51 ar	lncRNA	chr19:55158939	1643	4.610349823	1.609348752	-1.601	9.33E-11	7.939011
FMR1	fragile X mental retardation 1	protein_coding	chrX:147911951	8919	5.421741058	3.819034471	-0.591	9.41E-11	7.991387
WDR37	WD repeat domain 37	protein_coding	chr10:1049538-	6000	8.550355664	6.060773629	-0.581	9.44E-11	8.010313
LRSAM1	leucine rich repeat and sterile alpha motif	protein_coding	chr9:12745148	4365	10.77088331	7.209625641	-0.666	9.52E-11	8.066231
AFF3	AF4/FMR2 family member 3	protein_coding	chr2:99545419-	12737	3.153168316	2.017092372	-0.729	9.56E-11	8.090381
OXNAD1	oxidoreductase NAD binding domain cont	protein_coding	chr3:16265160-	6095	4.891825901	3.302784566	-0.654	9.60E-11	8.117113
EIF2S3B	eukaryotic translation initiation factor 2 s	protein_coding	chr12:10505602	2423	2.604534969	0.907173672	-1.597	9.62E-11	8.123502
GLRX5	glutaredoxin 5	protein_coding	chr14:95533503	2388	26.29738835	15.1038238	-0.889	9.69E-11	8.177715
PAFAH1B2	platelet activating factor acetylhydrolase	protein_coding	chr11:11714428	7084	16.3722523	12.56833279	-0.468	9.87E-11	8.300476
ZWINT	ZW10 interacting kinetochore protein	protein_coding	chr10:56357227	2377	9.330174233	5.939241759	-0.738	9.92E-11	8.334694
WASL	WASP like actin nucleation promoting fac	protein_coding	chr7:12368194	4363	19.96713005	15.34542402	-0.466	9.94E-11	8.344303
MAPK12	mitogen-activated protein kinase 12	protein_coding	chr22:50245450	9315	2.855816654	1.796254341	-0.756	9.97E-11	8.362224
AC144831.1	novel transcript	lncRNA	chr17:83104255	2656	1.675885121	0.339645624	-2.394	1.002697	8.391276
KNSTRN	kinetochore localized astrin (SPAG5) bind	protein_coding	chr15:40382721	3654	6.619372524	4.273815429	-0.715	1.002506	8.391276
TP53INP1	tumor protein p53 inducible nuclear prot	protein_coding	chr8:94925972-	5643	16.07748645	11.08793485	-0.621	1.002011	8.391276
LRPAP1	LDL receptor related protein associated p	protein_coding	chr4:3503612-3	11816	15.7627043	11.95077958	-0.486	1.011834	8.450258
TMEM63B	transmembrane protein 63B	protein_coding	chr6:44126914-	4425	19.88997092	14.68078232	-0.524	1.012310	8.450258
CHST3	carbohydrate sulfotransferase 3	protein_coding	chr10:71964395	6934	3.578389795	1.673342828	-1.174	1.016441	8.472934
LINC00702	long intergenic non-protein coding RNA 7	lncRNA	chr10:4201141-	12382	1.965713408	0.832563536	-1.317	1.015950	8.472934
SLC39A6	solute carrier family 39 member 6	protein_coding	chr18:36108531	3683	8.653034797	5.635845006	-0.707	1.026789	8.547307
CAST	calpastatin	protein_coding	chr5:96525267-	12448	14.60644367	11.11907542	-0.48	1.034615	8.606481
MTHFD2	methylenetetrahydrofolate dehydrogenas	protein_coding	chr2:74198610-	4822	9.223856977	6.469068312	-0.596	1.051115	8.725566
BUB1	BUB1 mitotic checkpoint serine/threonin	protein_coding	chr2:11063752	6304	7.047764115	4.554673549	-0.716	1.067942	8.859120
CYTH1	cytohesin 1	protein_coding	chr17:78674048	7754	5.007247549	3.102041765	-0.776	1.080045	8.953321
MED15	mediator complex subunit 15	protein_coding	chr22:20495913	10374	10.00507438	7.094044914	-0.582	1.083020	8.971778
ANKIB1	ankyrin repeat and IBR domain containing	protein_coding	chr7:92245974-	7389	5.629044282	3.624185549	-0.717	1.101806	9.102240
AVPI1	arginine vasopressin induced 1	protein_coding	chr10:97677424	1375	12.3303604	6.166675829	-1.079	1.117940	9.222815
REXO5	RNA exonuclease 5	protein_coding	chr16:20806429	4114	4.659535781	2.773624938	-0.831	1.120422	9.230586
CTIF	cap binding complex dependent translati	protein_coding	chr18:48539031	8115	12.08550297	8.270725457	-0.633	1.124314	9.256293
PAQR8	progestin and adipoQ receptor family mer	protein_coding	chr6:52361421-	5034	5.609853664	2.973123981	-1.001	1.126844	9.270749
NSL1	NSL1 component of MIS12 kinetochore co	protein_coding	chr1:21272615	13867	2.349298525	1.222518819	-1.022	1.128747	9.273676
RFTN1	raftlin, lipid raft linker 1	protein_coding	chr3:16313574-	4769	7.852317083	5.25864981	-0.664	1.128146	9.273676
MIR22HG	MIR22 host gene	lncRNA	chr17:1711493-	2986	12.7624095	8.755593573	-0.632	1.131519	9.290084
ATP1B3	ATPase Na+/K+ transporting subunit beta	protein_coding	chr3:14187612	2617	14.61067889	9.971126737	-0.636	1.139734	9.351128
HMG20A	high mobility group 20A	protein_coding	chr15:77420412	6403	8.682087592	6.420648519	-0.522	1.146087	9.396515
PA2G4	proliferation-associated 2G4	protein_coding	chr12:56104537	3580	24.80747058	16.89410178	-0.639	1.146834	9.396515
FAXDC2	fatty acid hydroxylase domain containing	protein_coding	chr5:15481849	6928	4.701667054	3.154913669	-0.661	1.149005	9.407873
TRAK1	trafficking kinesin protein 1	protein_coding	chr3:42013802-	10706	41.92201842	32.03746359	-0.474	1.150276	9.411855
KIF14	kinesin family member 14	protein_coding	chr1:20055149	7294	1.622142335	0.71602918	-1.266	1.152628	9.424661
MRPS35	mitochondrial ribosomal protein S35	protein_coding	chr12:27710822	1930	33.8174256	23.50468107	-0.61	1.163138	9.504114
YME1L1	YME1 like 1 ATPase	protein_coding	chr10:27110112	7335	29.47939078	22.51274401	-0.476	1.165161	9.514166
CTF1	cardiotrophin 1	protein_coding	chr16:30896607	1664	10.3881103	6.021908669	-0.873	1.167171	9.524085
AKAP13	A-kinase anchoring protein 13	protein_coding	chr15:8538057120280		26.10704175	15.63370215	-0.822	1.182521	9.642781
ESRP1	epithelial splicing regulatory protein 1	protein_coding	chr8:94641074-	4875	1.29566062	0.291133694	-2.248	1.183379	9.643215
CISH	cytokine inducible SH2 containing protei	protein_coding	chr3:50606490-	5112	6.82947725	4.753800461	-0.607	1.188501	9.678378
GPD1L	glycerol-3-phosphate dehydrogenase 1 lik	protein_coding	chr3:32105689-	4802	13.07976773	9.615567392	-0.529	1.191216	9.693899
KIF1C	kinesin family member 1C	protein_coding	chr17:4997950-	8578	52.68162121	40.48044463	-0.466	1.197587	9.739128
TCF7L1	transcription factor 7 like 1	protein_coding	chr2:85133392-	4431	7.364335829	4.514390492	-0.791	1.223872	9.925957
ZNF24	zinc finger protein 24	protein_coding	chr18:35332227	8242	12.05713842	8.794034293	-0.541	1.231698	9.982669
SLC20A1	solute carrier family 20 member 1	protein_coding	chr2:11264593	5493	7.025209901	4.281198399	-0.796	1.271150	1.03E-09
VASH1	vasohibin 1	protein_coding	chr14:76761468	7223	14.46347604	7.142594945	-1.097	1.275894	1.03E-09
IPO7	importin 7	protein_coding	chr11:9384652-	7216	40.30993825	31.10545683	-0.46	1.282532	1.03E-09
ITGB5	integrin subunit beta 5	protein_coding	chr3:12476194	6515	19.22013619	14.14325134	-0.528	1.283853	1.03E-09
NASP	nuclear autoantigenic sperm protein	protein_coding	chr1:45583846-	8304	15.98865832	12.31715019	-0.462	1.309022	1.05E-09
DCP1A	decapping mRNA 1A	protein_coding	chr3:53283429-	6102	7.540182544	4.897327049	-0.709	1.313364	1.06E-09
FAM78A	family with sequence similarity 78 memb	protein_coding	chr9:13125807	5462	51.62543096	36.65735876	-0.58	1.315335	1.06E-09
KRT8	keratin 8	protein_coding	chr12:52897187	6881	108.209179	78.57884863	-0.547	1.333998	1.07E-09
SUDS3	SDS3 homolog, SIN3A corepressor comple	protein_coding	chr12:11837655	5558	10.14938331	7.245762826	-0.572	1.339787	1.07E-09
PRSS8	serine protease 8	protein_coding	chr16:31131433	2975	1.994258317	0.41619178	-2.339	1.361903	1.09E-09
G3BP2	G3BP stress granule assembly factor 2	protein_coding	chr4:75642782-	7183	35.12293452	27.45658061	-0.441	1.380586	1.10E-09
LARP1	La ribonucleoprotein domain family mem	protein_coding	chr5:15471284	8549	36.90444151	26.88651641	-0.543	1.390258	1.11E-09
MED25	mediator complex subunit 25	protein_coding	chr19:49818279	7687	9.641132088	7.396773196	-0.469	1.393766	1.11E-09
CBWD1	COBW domain containing 1	protein_coding	chr9:121038-17	14170	1.692947899	1.009175021	-0.831	1.395593	1.11E-09
B3GALT2	beta-1,3-galactosyltransferase 2	protein_coding	chr1:19317904	3274	3.478880044	1.826830047	-1.019	1.423630	1.13E-09

WWTR1	WW domain containing transcription reg	protein_coding	chr3:14951723	8413	11.07479036	8.15623154	-0.527	1.444905	1.15E-09
PPM1L	protein phosphatase, Mg2+/Mn2+ depenc	protein_coding	chr3:16075560	12442	1.449969815	0.739808438	-1.054	1.450368	1.15E-09
LTBP4	latent transforming growth factor beta bi	protein_coding	chr19:4059288	8108	6.730351929	4.817092429	-0.568	1.453331	1.15E-09
LURAP1	leucine rich adaptor protein 1	protein_coding	chr1:46203334-	1849	9.592214906	5.050379164	-1.008	1.465926	1.16E-09
LIN7A	lin-7 homolog A, crumbs cell polarity com	protein_coding	chr12:8079252	6795	5.850698621	3.915990192	-0.665	1.471773	1.16E-09
NHSL1	NHS like 1	protein_coding	chr6:13842204	8741	10.92077833	7.420964287	-0.643	1.474733	1.17E-09
RARS2	arginyl-tRNA synthetase 2, mitochondrial	protein_coding	chr6:87513938-	2969	12.89354826	7.751788831	-0.82	1.514365	1.19E-09
PQLC1	PQ loop repeat containing 1	protein_coding	chr18:7990242	5218	6.157272843	3.425366166	-0.938	1.526837	1.20E-09
RAB30	RAB30, member RAS oncogene family	protein_coding	chr11:8297313	11832	2.078970263	1.090326179	-1.021	1.529326	1.20E-09
GASK1B	golgi associated kinase 1B	protein_coding	chr4:15812447	7880	9.066844903	6.669061966	-0.53	1.558626	1.22E-09
ARPIN	actin related protein 2/3 complex inhibit	protein_coding	chr15:8989500	8590	2.690469179	1.639337151	-0.8	1.563136	1.22E-09
TXNDC12	thioredoxin domain containing 12	protein_coding	chr1:52020131-	3844	1.123310241	0.262803775	-2.167	1.574013	1.23E-09
WFS1	wolframin ER transmembrane glycoprotei	protein_coding	chr4:6269849-6	4513	19.06000199	13.95233258	-0.536	1.585112	1.24E-09
SLC7A5	solute carrier family 7 member 5	protein_coding	chr16:8783002	4764	11.80966667	8.521016054	-0.557	1.587842	1.24E-09
CAPN2	calpain 2	protein_coding	chr1:22370159	8585	19.11553281	15.15405311	-0.421	1.599798	1.24E-09
TANGO2	transport and golgi organization 2 homol	protein_coding	chr22:2001701	6157	7.607520945	3.968794461	-1.016	1.633804	1.27E-09
KIF4A	kinesin family member 4A	protein_coding	chrX:70290104-	4573	2.522249167	1.353006091	-0.982	1.638987	1.27E-09
SLIT3	slit guidance ligand 3	protein_coding	chr5:16866173	14260	18.6949172	12.13007684	-0.71	1.643329	1.27E-09
CA13	carbonic anhydrase 13	protein_coding	chr8:85220587-	5597	1.130114179	0.382861132	-1.642	1.645407	1.27E-09
AGFG2	ArfGAP with FG repeats 2	protein_coding	chr7:10053920	5487	3.321807049	2.204697318	-0.676	1.663489	1.29E-09
GALC	galactosylceramidase	protein_coding	chr14:8783782	6736	8.971640198	6.773188372	-0.492	1.669063	1.29E-09
SPRED2	sprouty related EVH1 domain containing	protein_coding	chr2:65310851-	5505	1.611466216	0.688747849	-1.309	1.686223	1.30E-09
CYGB	cytoglobin	protein_coding	chr17:7652735	2753	1.22887666	0.355042307	-1.869	1.695044	1.31E-09
SYNDIG1	synapse differentiation inducing 1	protein_coding	chr20:2446962	2417	12.57139914	8.018065168	-0.733	1.712372	1.32E-09
NACA	nascent polypeptide associated complex	protein_coding	chr12:5671242	10008	41.11533054	29.90567181	-0.545	1.716904	1.32E-09
SERAC1	serine active site containing 1	protein_coding	chr6:15810951	11003	1.193766118	0.692033254	-0.87	1.724309	1.33E-09
NUP93	nucleoporin 93	protein_coding	chr16:5673011	11123	7.208990075	5.578719175	-0.456	1.728370	1.33E-09
LARP6	La ribonucleoprotein domain family mem	protein_coding	chr15:7082913	5554	6.794809106	4.752252333	-0.602	1.777491	1.36E-09
TBC1D8	TBC1 domain family member 8	protein_coding	chr2:101007617 6030		18.23564987	13.60059383	-0.508	1.785110	1.37E-09
DOK4	docking protein 4	protein_coding	chr16:5747192	4786	18.12630031	10.70115477	-0.844	1.813103	1.39E-09
FYCO1	FYVE and coiled-coil domain containing 1	protein_coding	chr3:45917899-	8895	25.44105178	15.61245753	-0.788	1.815977	1.39E-09
MARK3	microtubule affinity regulating kinase 3	protein_coding	chr14:1033853	8891	10.06371763	7.273648807	-0.555	1.827633	1.39E-09
SEPT11	septin 11	protein_coding	chr4:76949703-	9787	4.115750412	2.996390298	-0.543	1.835307	1.40E-09
SOX4	SRY-box 4	protein_coding	chr6:21592768-	5852	22.68374492	17.71367554	-0.443	1.838957	1.40E-09
APEH	acylaminoacyl-peptide hydrolase	protein_coding	chr3:49674014-	4425	29.98468079	21.69148245	-0.552	1.840444	1.40E-09
BCL7B	BAF chromatin remodeling complex subu	protein_coding	chr7:73536356-	3230	12.40401803	8.366765322	-0.655	1.842959	1.40E-09
FAM193B	family with sequence similarity 193 mem	protein_coding	chr5:17751978	5825	8.2908788	5.939104055	-0.566	1.872461	1.42E-09
RNPS1	RNA binding protein with serine rich dom	protein_coding	chr16:2253116-	8567	16.27912317	12.42333996	-0.476	1.878026	1.43E-09
GPN3	GPN-loop GTPase 3	protein_coding	chr12:1104524	2593	9.3287266	5.548867711	-0.836	1.900750	1.44E-09
PPM1H	protein phosphatase, Mg2+/Mn2+ depenc	protein_coding	chr12:6264399	7164	1.470978918	0.585452578	-1.416	1.959929	1.48E-09
PHLDB1	pleckstrin homology like domain family B	protein_coding	chr11:1186064	21715	9.078236546	5.914366628	-0.702	1.986657	1.50E-09
NMRK2	nicotinamide riboside kinase 2	protein_coding	chr19:3933103-	1141	37.65432388	11.02363943	-1.838	2.009407	1.52E-09
HSPA8	heat shock protein family A (Hsp70) mem	protein_coding	chr11:1230574	4849	181.3105233	129.3690281	-0.572	2.013682	1.52E-09
MCM5	minichromosome maintenance complex	protein_coding	chr22:3540013	7268	5.557191899	3.619263841	-0.701	2.022049	1.52E-09
SFT2D2	SFT2 domain containing 2	protein_coding	chr1:16822593	11217	1.585089494	0.914715534	-0.879	2.035289	1.53E-09
FANCD2	FA complementation group D2	protein_coding	chr3:10026414-	8378	2.155751376	1.303160761	-0.81	2.064114	1.55E-09
LRR1	leucine rich repeat protein 1	protein_coding	chr14:4959876	2295	4.760820939	2.572547188	-0.974	2.097113	1.57E-09
AIFM1	apoptosis inducing factor mitochondria a	protein_coding	chrX:13012936	3306	45.18546986	35.41301855	-0.438	2.101088	1.58E-09
BZW1	basic leucine zipper and W2 domains 1	protein_coding	chr2:20081059	8085	9.708209367	7.219423054	-0.514	2.109287	1.58E-09
LGALS3BP	galectin 3 binding protein	protein_coding	chr17:7897123	3422	74.60086324	55.82524232	-0.505	2.112108	1.58E-09
FBH1	F-box DNA helicase 1	protein_coding	chr10:5890203-	6676	23.70697922	18.29958446	-0.461	2.121371	1.59E-09
MYH7B	myosin heavy chain 7B	protein_coding	chr20:3497540	7931	98.11007292	59.76446206	-0.801	2.133054	1.60E-09
PLAA	phospholipase A2 activating protein	protein_coding	chr9:26903372-	5315	10.60602692	7.714978165	-0.544	2.133035	1.60E-09
DVL2	dishevelled segment polarity protein 2	protein_coding	chr17:7225342-	5321	11.20774059	7.439721764	-0.676	2.146640	1.60E-09
IGFBP6	insulin like growth factor binding protein	protein_coding	chr12:5309743	1346	4.855568368	1.689449852	-1.611	2.152909	1.61E-09
ARHGAP17	Rho GTPase activating protein 17	protein_coding	chr16:2491938	9903	3.825199883	2.794821862	-0.538	2.161841	1.61E-09
UTP4	UTP4 small subunit processome compone	protein_coding	chr16:6913129	5600	6.373009578	4.144358592	-0.706	2.187133	1.63E-09
ANTXR2	ANTXR cell adhesion molecule 2	protein_coding	chr4:79901146- 10010		9.74143905	7.333535781	-0.496	2.196389	1.63E-09
DAND5	DAN domain BMP antagonist family mem	protein_coding	chr19:1296515	1997	2.836517455	0.943386858	-1.674	2.217584	1.65E-09
CACNA1C	calcium voltage-gated channel subunit al	protein_coding	chr12:1970786-	19103	29.87437718	18.97182865	-0.738	2.270447	1.68E-09
ATP6V1G1	ATPase H+ transporting V1 subunit G1	protein_coding	chr9:11458776	1788	54.96242994	35.63496346	-0.709	2.275269	1.69E-09
CLSPN	claspin	protein_coding	chr1:35720218-	9591	1.306363941	0.708077611	-0.967	2.338943	1.73E-09
G2E3	G2/M-phase specific E3 ubiquitin protein	protein_coding	chr14:3055915	9319	2.097886632	1.299917435	-0.773	2.346258	1.73E-09
PLS1	plastin 1	protein_coding	chr3:14259639	5288	2.843299177	1.841309912	-0.713	2.346276	1.73E-09
EDIL3	EGF like repeats and discoidin domains 3	protein_coding	chr5:83940554-	5825	1.740778853	0.889724	-1.058	2.351888	1.74E-09
DDX41	DEAD-box helicase 41	protein_coding	chr5:17751157	4177	10.79539375	7.765147753	-0.56	2.359028	1.74E-09
PACSIN2	protein kinase C and casein kinase substra	protein_coding	chr22:4283541	4558	25.98195744	20.53009883	-0.426	2.379563	1.75E-09
PPP1R15A	protein phosphatase 1 regulatory subunit	protein_coding	chr19:4887242	3065	34.71585679	26.83685733	-0.458	2.382169	1.75E-09
LNPEP	leucyl and cystinyl aminopeptidase	protein_coding	chr5:96935394-	14966	9.034121667	6.266713055	-0.613	2.388299	1.76E-09
TPCN1	two pore segment channel 1	protein_coding	chr12:1132210	8728	16.36414334	11.71741723	-0.568	2.430431	1.79E-09
WDR73	WD repeat domain 73	protein_coding	chr15:8463928	9626	3.738203235	2.281308825	-0.804	2.438495	1.79E-09
WDR61	WD repeat domain 61	protein_coding	chr15:7827783	7188	4.631184486	3.304350834	-0.574	2.472114	1.81E-09
CUX2	cut like homeobox 2	protein_coding	chr12:1110341	7507	16.39441385	10.87061681	-0.677	2.479121	1.81E-09
ARHGEF12	Rho guanine nucleotide exchange factor 1	protein_coding	chr11:1203364	24906	11.77187151	7.651061159	-0.705	2.485996	1.82E-09
GAB1	GRB2 associated binding protein 1	protein_coding	chr4:14333676	10119	21.99102474	17.21232073	-0.44	2.524330	1.84E-09
GON7	GON7 subunit of KEOPS complex	protein_coding	chr14:9320289	1125	9.253771492	4.605749845	-1.093	2.543433	1.85E-09
MORN4	MORN repeat containing 4	protein_coding	chr10:9761455	2512	6.196590046	3.44604766	-0.929	2.545882	1.85E-09
ZNF317	zinc finger protein 317	protein_coding	chr19:9140397-	5048	5.63181304	3.896234985	-0.616	2.555752	1.86E-09
FAM120B	family with sequence similarity 120B	protein_coding	chr6:17029070	6029	8.825883822	6.632855097	-0.498	2.571473	1.87E-09
SEPT8	septin 8	protein_coding	chr5:13275081	7709	12.73021263	9.857565098	-0.455	2.590589	1.88E-09
P3H2	prolyl 3-hydroxylase 2	protein_coding	chr3:18995672	5573	12.69278545	8.974748047	-0.586	2.596548	1.88E-09
MGST1	microsomal glutathione S-transferase 1	protein_coding	chr12:1634714	7441	2.424993777	1.295898216	-0.993	2.606840	1.89E-09
RBM5	RNA binding motif protein 5	protein_coding	chr3:50088908-	10368	14.49000713	11.34002301	-0.44	2.610951	1.89E-09
PIK3R3	phosphoinositide-3-kinase regulatory sub	protein_coding	chr1:46040140-	7397	15.63904987	11.11650983	-0.578	2.633597	1.90E-09
PLCD3	phospholipase C delta 3	protein_coding	chr17:4510895	6748	11.98121324	8.735687388	-0.542	2.651216	1.92E-09
SEC62	SEC62 homolog, preprotein translocatior	protein_coding	chr3:16996663	7928	7.13160985	5.186703887	-0.546	2.654391	1.92E-09
FHOD1	formin homology 2 domain containing 1	protein_coding	chr16:6722938	5230	4.454874177	2.760603649	-0.772	2.660894	1.92E-09
PLCE1	phospholipase C epsilon 1	protein_coding	chr10:9403081	12930	2.233156992	0.903688859	-1.382	2.684495	1.94E-09
RSF1	remodeling and spacing factor 1	protein_coding	chr11:7765999	15740	5.892130898	4.475504567	-0.483	2.710204	1.95E-09
SEPT2	septin 2	protein_coding	chr2:24131510	7378	67.64626678	53.23047176	-0.432	2.715030	1.95E-09
LHFPL6	LHFPL tetraspan subfamily member 6	protein_coding	chr13:3920911	4564	19.82097899	15.39618412	-0.45	2.726874	1.96E-09
UBXN11	UBX domain protein 11	protein_coding	chr1:26281328-	4704	2.445757191	1.454712509	-0.836	2.740150	1.97E-09
MYCN	MYCN proto-oncogene, bHLH transcriptic	protein_coding	chr2:15940550-	2616	18.04037896	12.53009631	-0.613	2.757435	1.97E-09
CENPT	centromere protein T	protein_coding	chr16:6782815	5660	8.297180759	6.310578746	-0.481	2.838202	2.02E-09
NACC2	NACC family member 2	protein_coding	chr9:13600653	7216	16.63327865	10.79132214	-0.708	2.861965	2.04E-09
DGLUCY	D-glutamate cyclase	protein_coding	chr14:9106033	7212	18.56798077	14.34020086	-0.459	2.891961	2.05E-09
SMG5	SMG5 nonsense mediated mRNA decay fac	protein_coding	chr1:15624922	5304	52.51084496	38.0455297	-0.55	2.927617	2.08E-09
CORO1C	coronin 1C	protein_coding	chr12:1086451	6532	12.39152862	9.586290398	-0.457	2.956976	2.10E-09
CEACAM19	carcinoembryonic antigen related cell adl	protein_coding	chr19:4466227	5203	3.12125224	1.754903578	-0.916	2.961405	2.10E-09
PRKAR2A	protein kinase cAMP-dependent type II re	protein_coding	chr3:48744597-	6665	22.44730533	16.79105058	-0.504	2.967796	2.10E-09
FYTTD1	forty-two-three domain containing 1	protein_coding	chr3:19773717	7897	8.472979844	6.46107593	-0.477	3.010151	2.13E-09
ECT2	epithelial cell transforming 2	protein_coding	chr3:17275068	5499	5.409521623	3.431315032	-0.74	3.027884	2.14E-09
SDC3	syndecan 3	protein_coding	chr1:30869466-	7065	32.38501353	22.1601879	-0.632	3.094661	2.18E-09
CNBP	CCHC-type zinc finger nucleic acid binding	protein_coding	chr3:12916948	1927	129.1140338	98.73722832	-0.473	3.097796	2.18E-09
STRN	striatin	protein_coding	chr2:36837698-	14351	13.76098499	10.61341416	-0.461	3.152393	2.22E-09
INKA2	inka box actin regulator 2	protein_coding	chr1:11168063	6393	11.71769239	8.679058845	-0.52	3.159037	2.22E-09
SNX19	sorting nexin 19	protein_coding	chr11:1308754	8152	11.17551914	8.175717882	-0.536	3.159903	2.22E-09
SIKE1	suppressor of IKBKE 1	protein_coding	chr1:11476947	5894	13.39503426	10.32575096	-0.462	3.181343	2.24E-09
PSAT1	phosphoserine aminotransferase 1	protein_coding	chr9:78297125-	2206	6.391359844	3.631077026	-0.903	3.185121	2.24E-09

SLC6A8	solute carrier family 6 member 8	protein_coding	chrX:15368792€	5666	30.94268027	22.73447938	-0.53	3.241640	2.27E-09
PNO1	partner of NOB1 homolog	protein_coding	chr2:68157888-	2630	10.01000927	6.167646726	-0.781	3.245121	2.27E-09
FNDC10	fibronectin type III domain containing 10	protein_coding	chr1:1598012-1	2124	22.5270483	10.14669304	-1.229	3.274851	2.29E-09
DUSP3	dual specificity phosphatase 3	protein_coding	chr17:43766125	4997	78.31531713	61.87850221	-0.426	3.358479	2.34E-09
DNAJC30	DnaJ heat shock protein family (Hsp40) m	protein_coding	chr7:73680969-	2485	14.66923691	8.606999697	-0.861	3.383585	2.35E-09
MN1	MN1 proto-oncogene, transcriptional reg	protein_coding	chr22:27748277	7951	1.158607267	0.286201762	-2.105	3.470311	2.40E-09
NOG	noggin	protein_coding	chr17:56593699	1892	2.163681074	0.770472762	-1.572	3.485510	2.41E-09
MAP3K9	mitogen-activated protein kinase kinase k	protein_coding	chr14:70722526	12386	1.414729525	0.531889188	-1.483	3.501617	2.42E-09
METTL2B	methyltransferase like 2B	protein_coding	chr7:12847672$	6749	4.397594409	2.876839166	-0.699	3.542065	2.45E-09
POLD1	DNA polymerase delta 1, catalytic subunit	protein_coding	chr19:50384204	4934	6.018253927	4.248396666	-0.587	3.552508	2.45E-09
AP1M1	adaptor related protein complex 1 subuni	protein_coding	chr19:16197578	14371	4.560215128	3.410642211	-0.505	3.585217	2.47E-09
ARID3B	AT-rich interaction domain 3B	protein_coding	chr15:74541177	6212	3.04608611	1.50021702	-1.103	3.612255	2.49E-09
H1F0	H1 histone family member 0	protein_coding	chr22:37805093	2344	11.19437207	6.489361681	-0.87	3.617767	2.49E-09
PPME1	protein phosphatase methylesterase 1	protein_coding	chr11:74171267	7749	7.142932546	4.653693629	-0.703	3.650421	2.51E-09
AC022966.1	ubiquitin specific peptidase 36	protein_coding	chr17:78787381	9235	4.778174277	3.135057541	-0.691	3.724487	2.56E-09
GADD45A	growth arrest and DNA damage inducible	protein_coding	chr1:67685201-	1925	60.35611998	43.89119808	-0.544	3.743210	2.57E-09
GGA2	golgi associated, gamma adaptin ear cont	protein_coding	chr16:23463542	8231	20.90703936	15.46084667	-0.521	3.778376	2.59E-09
SUSD4	sushi domain containing 4	protein_coding	chr1:22322081$	4686	1.595775218	0.689036134	-1.296	3.860566	2.64E-09
PPP2CB	protein phosphatase 2 catalytic subunit b	protein_coding	chr8:30774457-	4510	26.28114474	19.68971581	-0.503	3.865817	2.64E-09
HMGXB3	HMG-box containing 3	protein_coding	chr5:15000004€	6263	9.483805894	7.359217028	-0.452	3.882655	2.65E-09
PATL1	PAT1 homolog 1, processing body mRNA (	protein_coding	chr11:59636716	4335	10.70688378	6.834952481	-0.732	3.966872	2.71E-09
PDGFA	platelet derived growth factor subunit A	protein_coding	chr7:497258-52	4151	4.581307901	2.943292722	-0.724	4.002618	2.73E-09
RALA	RAS like proto-oncogene A	protein_coding	chr7:39623565-	3579	13.35407132	9.722173679	-0.544	4.019079	2.74E-09
GATA5	GATA binding protein 5	protein_coding	chr20:62463497	2620	2.968418899	1.253952833	-1.32	4.043869	2.75E-09
PRDM5	PR/SET domain 5	protein_coding	chr4:12068491$	9517	1.793624999	1.02295477	-0.896	4.082818	2.77E-09
TRIM24	tripartite motif containing 24	protein_coding	chr7:13846025$	9301	21.95755704	16.6504734	-0.486	4.102863	2.79E-09
MYRF	myelin regulatory factor	protein_coding	chr11:61752642 10680		1.108702926	0.292434815	-1.99	4.224676	2.86E-09
STYX	serine/threonine/tyrosine interacting pro	protein_coding	chr14:52730166	4983	8.682697197	6.099830721	-0.597	4.237189	2.87E-09
PLXNA2	plexin A2	protein_coding	chr1:208022242	13845	9.265030872	5.136232707	-0.931	4.250053	2.87E-09
ADAMTSL1	ADAMTS like 1	protein_coding	chr9:17906563-	13446	0.93355673	0.413344528	-1.255	4.298063	2.90E-09
S100A16	S100 calcium binding protein A16	protein_coding	chr1:15360688€	1701	23.44867085	14.56381375	-0.771	4.297591	2.90E-09
FREM1	FRAS1 related extracellular matrix 1	protein_coding	chr9:14734666-	10762	4.143632237	2.262135955	-0.955	4.376310	2.95E-09
LOXL4	lysyl oxidase like 4	protein_coding	chr10:98247690	3597	2.357255078	1.229070625	-1.028	4.390042	2.95E-09
CUL3	cullin 3	protein_coding	chr2:224470150	11505	8.171314158	6.214716284	-0.481	4.411241	2.97E-09
TMEM176B	transmembrane protein 176B	protein_coding	chr7:15079128$	1979	1.795305057	0.625444427	-1.6	4.444824	2.98E-09
RREB1	ras responsive element binding protein 1	protein_coding	chr6:7107597-7	17254	5.380877781	3.756452642	-0.603	4.450480	2.99E-09
NFIX	nuclear factor I X	protein_coding	chr19:12995608	6302	2.302219867	1.286227291	-0.922	4.496973	3.01E-09
AMIGO1	adhesion molecule with Ig like domain 1	protein_coding	chr1:109504178	5141	5.410700434	3.894541864	-0.561	4.568153	3.05E-09
COL11A1	collagen type XI alpha 1 chain	protein_coding	chr1:102876467	9091	1.166630478	0.493144005	-1.328	4.587131	3.06E-09
ATF7	activating transcription factor 7	protein_coding	chr12:53507856	7985	2.988635539	2.037152536	-0.638	4.591801	3.06E-09
ATG4D	autophagy related 4D cysteine peptidase	protein_coding	chr19:10543895	2278	32.11638801	25.2534219	-0.433	4.640535	3.09E-09
ATAD1	ATPase family AAA domain containing 1	protein_coding	chr10:87751512	5067	30.38006935	23.24310182	-0.472	4.671600	3.10E-09
NCOA1	nuclear receptor coactivator 1	protein_coding	chr2:24491914-	8633	8.203220614	5.567836592	-0.643	4.682360	3.11E-09
PNPLA2	patatin like phospholipase domain contai	protein_coding	chr11:818914-8	3831	47.7356123	38.54858681	-0.395	4.775254	3.16E-09
PPP1R1B	protein phosphatase 1 regulatory inhibitc	protein_coding	chr17:39626740	4543	2.430488427	1.3317779	-0.953	4.926262	3.26E-09
PCYT2	phosphate cytidylyltransferase 2, ethanol	protein_coding	chr17:81900965	6897	14.16736119	10.28049344	-0.548	4.965087	3.28E-09
DUSP15	dual specificity phosphatase 15	protein_coding	chr20:31847637	3791	4.006775545	2.387688496	-0.831	4.987756	3.29E-09
TMEM209	transmembrane protein 209	protein_coding	chr7:13016471З	4669	2.885185751	1.800999848	-0.766	5.001650	3.30E-09
TGFB2	transforming growth factor beta 2	protein_coding	chr1:21834533€ 6050		2.567292117	1.589421471	-0.776	5.071360	3.34E-09
OSBPL10	oxysterol binding protein like 10	protein_coding	chr3:31657890-	11976	1.309230698	0.760320436	-0.869	5.130000	3.38E-09
PPM1G	protein phosphatase, Mg2+/Mn2+ depenc	protein_coding	chr2:27381195-	3995	22.93228719	17.6343564	-0.465	5.137924	3.38E-09
TBC1D22B	TBC1 domain family member 22B	protein_coding	chr6:37257772-	3462	6.746052818	4.407105411	-0.7	5.148637	3.38E-09
ABHD18	abhydrolase domain containing 18	protein_coding	chr4:12796530€	6707	12.13109261	9.294310541	-0.47	5.154674	3.38E-09
RAD51	RAD51 recombinase	protein_coding	chr15:40694774	3084	1.701713411	0.385762663	-2.222	5.165111	3.39E-09
ANKRD9	ankyrin repeat domain 9	protein_coding	chr14:10250176	7208	22.79604757	14.91367136	-0.697	5.207918	3.41E-09
TACC3	transforming acidic coiled-coil containing	protein_coding	chr4:1712891-1	8202	5.238277175	3.722287481	-0.579	5.243142	3.43E-09
GYPC	glycophorin C (Gerbich blood group)	protein_coding	chr2:126656133	3609	5.233163258	2.697598378	-1.036	5.279303	3.45E-09
NAPRT	nicotinate phosphoribosyltransferase	protein_coding	chr8:14357478$	2837	18.46116195	13.20456701	-0.568	5.285416	3.45E-09
NDUFC1	NADH:ubiquinone oxidoreductase subuni	protein_coding	chr4:13926688C	1941	33.88575344	17.78738632	-1.009	5.284957	3.45E-09
STX7	syntaxin 7	protein_coding	chr6:132445867	16387	4.690381528	3.641485421	-0.45	5.311338	3.47E-09
ZADH2	zinc binding alcohol dehydrogenase doma	protein_coding	chr18:75195113	7943	10.39408051	7.836337874	-0.494	5.329363	3.48E-09
PODN	podocan	protein_coding	chr1:53062052-	4900	2.346729358	1.173439254	-1.081	5.379464	3.50E-09
DAGLB	diacylglycerol lipase beta	protein_coding	chr7:6409126-6	5715	1.869155414	1.108485132	-0.842	5.420931	3.52E-09
PCGF3	polycomb group ring finger 3	protein_coding	chr4:705748-77	7425	8.989632977	6.691560097	-0.513	5.419604	3.52E-09
HES4	hes family bHLH transcription factor 4	protein_coding	chr1:998962-10	1118	54.76438771	31.88532912	-0.868	5.425457	3.52E-09
SLC25A39	solute carrier family 25 member 39	protein_coding	chr17:44319625	3526	34.9429533	26.53937052	-0.482	5.471930	3.55E-09
MTRF1L	mitochondrial translational release factor	protein_coding	chr6:152987362	4297	3.816265855	2.501310549	-0.696	5.481474	3.55E-09
DPY19L3	dpy-19 like C-mannosyltransferase 3	protein_coding	chr19:32405543	10781	2.686723358	1.655643752	-0.783	5.540492	3.58E-09
POLG	DNA polymerase gamma, catalytic subuni	protein_coding	chr15:89305198	5980	9.703099711	6.997264885	-0.557	5.573743	3.60E-09
CAPN10	calpain 10	protein_coding	chr2:240586734	7456	8.045907003	5.772608903	-0.564	5.587571	3.61E-09
STX6	syntaxin 6	protein_coding	chr1:180972712	6477	3.865871858	2.656885255	-0.63	5.597783	3.61E-09
C8orf33	chromosome 8 open reading frame 33	protein_coding	chr8:14505246$	4353	11.76358909	8.331735001	-0.582	5.618183	3.62E-09
CRACR2B	calcium release activated channel regulat	protein_coding	chr11:826144-8	4224	8.193926475	4.738125522	-0.87	5.722969	3.68E-09
HAGH	hydroxyacylglutathione hydrolase	protein_coding	chr16:1795620-	5839	18.65500364	13.06933801	-0.601	5.750920	3.69E-09
ERI1	exoribonuclease 1	protein_coding	chr8:9002147-9	6385	1.661714634	0.943416985	-0.909	5.756285	3.69E-09
FSTL1	follistatin like 1	protein_coding	chr3:12039229З	8467	36.46492918	29.18047596	-0.407	5.811414	3.72E-09
KRT80	keratin 80	protein_coding	chr12:52168996	4364	1.862079782	0.475899758	-2.04	5.840332	3.74E-09
SDR39U1	short chain dehydrogenase/reductase fam	protein_coding	chr14:24439766	3099	18.78540542	13.8471968	-0.527	5.852360	3.74E-09
KIF18B	kinesin family member 18B	protein_coding	chr17:44924709	4655	2.766471507	1.556331974	-0.915	5.880314	3.76E-09
PPP1R3D	protein phosphatase 1 regulatory subunit	protein_coding	chr20:59936663	3643	8.875149903	6.191685931	-0.606	5.892847	3.76E-09
KLF11	Kruppel like factor 11	protein_coding	chr2:10042849-	4594	3.840938616	2.201454211	-0.888	5.967108	3.80E-09
FAM114A2	family with sequence similarity 114 meml	protein_coding	chr5:153990148	6523	3.597754905	2.578592757	-0.566	6.009991	3.83E-09
C8orf37	chromosome 8 open reading frame 37	protein_coding	chr8:95244913-	3340	2.239800033	1.097195587	-1.119	6.021381	3.83E-09
KLF15	Kruppel like factor 15	protein_coding	chr3:126342635	2814	4.55041038	2.319837898	-1.05	6.035259	3.84E-09
SHROOM3	shroom family member 3	protein_coding	chr4:76435229-	13829	8.64239909	5.946295694	-0.624	6.083731	3.87E-09
NFE2L1	nuclear factor, erythroid 2 like 1	protein_coding	chr17:48048329	5883	122.2408216	93.70152265	-0.47	6.090373	3.87E-09
CDR2L	cerebellar degeneration related protein 2	protein_coding	chr17:74987632	3536	3.359292782	1.807819484	-0.979	6.112487	3.88E-09
KLHL24	kelch like family member 24	protein_coding	chr3:183635610	8650	18.7724469	14.56953098	-0.451	6.135328	3.90E-09
ZNF853	zinc finger protein 853	protein_coding	chr7:6615610-6	3864	7.600660278	5.311104911	-0.603	6.161067	3.91E-09
QARS	glutaminyl-tRNA synthetase	protein_coding	chr3:49095932-	4703	48.44532598	34.88224362	-0.559	6.249365	3.96E-09
RRM2	ribonucleotide reductase regulatory subu	protein_coding	chr2:10120698-	9155	4.99690686	3.256124036	-0.702	6.351249	4.02E-09
SLC16A9	solute carrier family 16 member 9	protein_coding	chr10:59650761	4429	1.827983002	0.936061302	-1.043	6.497761	4.11E-09
SLCO3A1	solute carrier organic anion transporter fa	protein_coding	chr15:91853708	9422	10.92011047	8.541467773	-0.44	6.503267	4.11E-09
SPRY1	sprouty RTK signaling antagonist 1	protein_coding	chr4:123396795	3963	2.488338761	1.245873956	-1.086	6.532454	4.13E-09
SPPL2B	signal peptide peptidase like 2B	protein_coding	chr19:2328615-	7419	11.0628229	7.845784236	-0.58	6.573589	4.15E-09
TAF3	TATA-box binding protein associated facto	protein_coding	chr10:7818505-	4875	3.210643132	1.975289962	-0.783	6.615600	4.17E-09
SRPRA	SRP receptor subunit alpha	protein_coding	chr11:12626293	4148	14.37509063	11.02668237	-0.467	6.706910	4.22E-09
SCHIP1	schwannomin interacting protein 1	protein_coding	chr3:159839861	7645	1.604280192	0.932182491	-0.869	6.740853	4.24E-09
RARA	retinoic acid receptor alpha	protein_coding	chr17:40309180	4743	13.81275319	8.753778156	-0.745	6.822858	4.29E-09
TIPRL	TOR signaling pathway regulator	protein_coding	chr1:168178962	3186	17.13383492	11.85689698	-0.616	6.837056	4.30E-09
WDR45	WD repeat domain 45	protein_coding	chrX:49074433-	4691	3.458353265	2.240747278	-0.709	6.891151	4.32E-09
C8orf49	chromosome 8 open reading frame 49	lncRNA	chr8:11761256-	1968	3.478392074	1.766049027	-1.063	6.898391	4.33E-09
GDF2	growth differentiation factor 2	protein_coding	chr10:47322490	1955	1.61253073	0.441950713	-1.954	6.909654	4.33E-09
CCNDBP1	cyclin D1 binding protein 1	protein_coding	chr15:43185118	6655	9.519934383	7.411668258	-0.448	6.946224	4.35E-09
PKN3	protein kinase N3	protein_coding	chr9:128702503	3393	2.915709646	1.593591235	-0.955	7.060773	4.41E-09
HAUS8	HAUS augmin like complex subunit 8	protein_coding	chr19:17049729	5533	1.225027551	0.493749355	-1.387	7.151122	4.46E-09
DDX46	DEAD-box helicase 46	protein_coding	chr5:134758771	9205	6.278011118	4.534148499	-0.557	7.235745	4.51E-09
CCP110	centriolar coiled-coil protein 110	protein_coding	chr16:19523811	10142	2.022303033	1.357550057	-0.664	7.410671	4.61E-09
TSPAN9	tetraspanin 9	protein_coding	chr12:3077355-	6905	60.55532149	46.73791178	-0.46	7.435847	4.62E-09

LACC1	laccase domain containing 1	protein_coding	chr13:43879284	4479	3.831899876	2.154092453	-0.917	7.460395	4.63E-09
EMC10	ER membrane protein complex subunit 1(	protein_coding	chr19:50476400	10748	19.28714818	15.56684599	-0.396	7.465696	4.63E-09
ICMT	isoprenylcysteine carboxyl methyltransfe	protein_coding	chr1:6221193-6	4910	24.74041068	19.49483511	-0.43	7.654213	4.74E-09
CXorf38	chromosome X open reading frame 38	protein_coding	chrX:40626921- 6050		1.909992586	1.098537529	-0.886	7.689064	4.76E-09
ACSS2	acyl-CoA synthetase short chain family me	protein_coding	chr20:34872146	5248	22.76494237	16.64978062	-0.536	7.754363	4.79E-09
CORIN	corin, serine peptidase	protein_coding	chr4:47593999-	10086	28.50717982	23.16754173	-0.386	7.807146	4.82E-09
LINC00997	long intergenic non-protein coding RNA 9	lncRNA	chr7:32760279-	2646	1.912547427	0.923510604	-1.132	7.841155	4.84E-09
CNN3	calponin 3	protein_coding	chr1:94896949-	2783	61.99313781	48.53963609	-0.439	7.947005	4.90E-09
FOSL2	FOS like 2, AP-1 transcription factor subui	protein_coding	chr2:28392448-	7162	2.698885668	1.25429328	-1.184	7.961666	4.91E-09
TAOK2	TAO kinase 2	protein_coding	chr16:29973868	8831	15.33747829	11.59050631	-0.489	8.031258	4.95E-09
ITGA7	integrin subunit alpha 7	protein_coding	chr12:55684568	6373	71.8919739	48.40416457	-0.655	8.120257	5.00E-09
USP31	ubiquitin specific peptidase 31	protein_coding	chr16:23061406	12017	1.859326752	1.039795729	-0.921	8.140795	5.01E-09
UAP1L1	UDP-N-acetylglucosamine pyrophosphory	protein_coding	chr9:137077517	5267	7.028954186	2.705284045	-1.447	8.214647	5.05E-09
TMEM139	transmembrane protein 139	protein_coding	chr7:143279957	2564	1.42630195	0.537064841	-1.485	8.290957	5.09E-09
CENPL	centromere protein L	protein_coding	chr1:173799550	4914	1.669359567	0.800216631	-1.14	8.361301	5.13E-09
AL356740.1	novel transcript	lncRNA	chr13:11296483	1297	2.023743441	0.427652942	-2.304	8.385697	5.14E-09
ATP6V0B	ATPase H+ transporting V0 subunit b	protein_coding	chr1:43974487-	3239	33.12490831	24.28224944	-0.535	8.384196	5.14E-09
AL590004.3	novel transcript	lncRNA	chr6:3904920-3 7060		5.652039631	1.891436941	-1.648	8.464583	5.18E-09
ZNF689	zinc finger protein 689	protein_coding	chr16:30602558	4159	4.231959948	2.662523447	-0.754	8.587101	5.25E-09
TMX2	thioredoxin related transmembrane prot	protein_coding	chr11:57712593	2253	35.01831141	26.31741708	-0.497	8.667848	5.29E-09
SLC38A3	solute carrier family 38 member 3	protein_coding	chr3:50205246-	3968	2.562308327	1.458427478	-0.896	8.687299	5.30E-09
FAM162A	family with sequence similarity 162 mem	protein_coding	chr3:122384176	3793	23.24808527	16.44426667	-0.586	8.764737	5.34E-09
CSNK1G1	casein kinase 1 gamma 1	protein_coding	chr15:64165525	9618	1.568239432	0.932206263	-0.833	8.835089	5.38E-09
AUP1	AUP1 lipid droplet regulating VLDL assem	protein_coding	chr2:74526645-	2455	20.67328765	14.72260634	-0.576	8.924858	5.43E-09
DUSP22	dual specificity phosphatase 22	protein_coding	chr6:291630-35	5686	7.391648331	5.242903117	-0.578	9.048978	5.50E-09
THUMPD1	THUMP domain containing 1	protein_coding	chr16:20702816	6501	3.724435906	2.41989849	-0.709	9.094034	5.52E-09
NCAPD3	non-SMC condensin II complex subunit D	protein_coding	chr11:13415011	9114	3.081845871	1.95816603	-0.737	9.100946	5.52E-09
PIGT	phosphatidylinositol glycan anchor biosy	protein_coding	chr20:45416084	19653	5.094872514	3.799032979	-0.509	9.304268	5.63E-09
SIK2	salt inducible kinase 2	protein_coding	chr11:11160244	9740	5.624242294	3.459574923	-0.784	9.311668	5.63E-09
CDC45	cell division cycle 45	protein_coding	chr22:19474945	2846	2.455785279	1.296924166	-1.007	9.399486	5.67E-09
MXRA7	matrix remodeling associated 7	protein_coding	chr17:76672551	9257	42.94363202	34.94179273	-0.384	9.439690	5.69E-09
P4HTM	prolyl 4-hydroxylase, transmembrane	protein_coding	chr3:48989889-	7637	4.022265725	2.782143708	-0.62	9.452426	5.70E-09
NPR3	natriuretic peptide receptor 3	protein_coding	chr5:32689070-	9979	4.988358324	2.493869902	-1.074	9.483861	5.71E-09
AC125807.2	novel transcript	lncRNA	chr12:3041437-	3514	2.698437718	1.634336366	-0.809	9.510133	5.72E-09
WWC3	WWC family member 3	protein_coding	chrX:10015562-	7114	6.934990484	4.538686038	-0.695	9.527408	5.73E-09
MCM3	minichromosome maintenance complex	protein_coding	chr6:52264009-	3650	24.80679194	18.29703402	-0.524	9.539157	5.73E-09
FANCG	FA complementation group G	protein_coding	chr9:35073835-	3614	2.64735734	1.507429971	-0.892	9.733381	5.84E-09
CCND1	cyclin D1	protein_coding	chr11:69641087	4830	58.15949301	45.28187716	-0.446	9.823127	5.89E-09
ETS2	ETS proto-oncogene 2, transcription facto	protein_coding	chr21:38805183	4977	11.99219894	8.75912992	-0.538	9.874798	5.92E-09
ATF2	activating transcription factor 2	protein_coding	chr2:175072250	5751	6.49336445	4.74900617	-0.537	9.931213	5.95E-09
PRKCA	protein kinase C alpha	protein_coding	chr17:66302613	11175	3.287585332	2.166333248	-0.685	1.02E-09	6.09E-09
CKB	creatine kinase B	protein_coding	chr14:10351966	2383	112.9767101	83.57278229	-0.521	1.02E-09	6.10E-09
EIPR1	EARP complex and GARP complex interact	protein_coding	chr2:3188925-3	5775	1.141902713	0.508689691	-1.245	1.04E-09	6.18E-09
PICALM	phosphatidylinositol binding clathrin ass	protein_coding	chr11:85957175	6342	29.67972634	23.54611599	-0.42	1.06E-09	6.30E-09
CDC40	cell division cycle 40	protein_coding	chr6:110180141	5955	3.124313195	1.921608432	-0.789	1.06E-09	6.32E-09
WNT5B	Wnt family member 5B	protein_coding	chr12:1529891-	4240	8.415709892	6.215155355	-0.525	1.07E-09	6.37E-09
KLHL31	kelch like family member 31	protein_coding	chr6:53647916-	5776	34.77360768	28.55985873	-0.37	1.08E-09	6.40E-09
PRADC1	protease associated domain containing 1	protein_coding	chr2:73228010-	1404	38.56220653	25.17927007	-0.698	1.08E-09	6.40E-09
TRAF3	TNF receptor associated factor 3	protein_coding	chr14:10277747	8263	3.537604379	2.198503956	-0.776	1.09E-09	6.47E-09
GTF2B	general transcription factor IIB	protein_coding	chr1:88852633-	2524	6.65478906	4.141119704	-0.768	1.10E-09	6.53E-09
PIN1	peptidylprolyl cis/trans isomerase, NIMA-	protein_coding	chr19:9835257-	4570	22.43059805	17.27210174	-0.464	1.11E-09	6.53E-09
MRPL57	mitochondrial ribosomal protein L57	protein_coding	chr13:21176658	2233	17.92464065	11.66488527	-0.708	1.12E-09	6.59E-09
MAP3K10	mitogen-activated protein kinase kinase k	protein_coding	chr19:40191426	4884	4.992317092	2.817723622	-0.909	1.12E-09	6.60E-09
TOX	thymocyte selection associated high mob	protein_coding	chr8:58805412-	4076	3.58016616	2.086671431	-0.864	1.14E-09	6.70E-09
EFR3B	EFR3 homolog B	protein_coding	chr2:25042076-	10643	1.211784767	0.731187002	-0.814	1.14E-09	6.70E-09
NKX2-6	NK2 homeobox 6	protein_coding	chr8:23702451-	906	16.35923182	10.87007633	-0.675	1.16E-09	6.80E-09
FAM171A1	family with sequence similarity 171 mem	protein_coding	chr10:15211643	4182	14.7963734	11.31017513	-0.474	1.16E-09	6.81E-09
SMC4	structural maintenance of chromosomes	protein_coding	chr3:160399274	8164	11.11449799	8.058061166	-0.55	1.19E-09	6.97E-09
TGFB1	transforming growth factor beta 1	protein_coding	chr19:41301587	3238	14.22065577	9.431067325	-0.675	1.20E-09	7.01E-09
WDR86	WD repeat domain 86	protein_coding	chr7:151375909	4044	1.317734044	0.596079492	-1.221	1.21E-09	7.05E-09
STAMBP	STAM binding protein	protein_coding	chr2:73828916-	7694	3.737220127	2.756225255	-0.526	1.22E-09	7.12E-09
AMOTL2	angiomotin like 2	protein_coding	chr3:134355874	5984	18.22746542	13.4113732	-0.528	1.25E-09	7.29E-09
AC068700.1	novel transcript, overlapping to PKIA	lncRNA	chr8:78605952-	3754	1.074015546	0.361370196	-1.642	1.26E-09	7.30E-09
HMGB2	high mobility group box 2	protein_coding	chr4:173331695	2775	43.94594046	31.70693807	-0.557	1.26E-09	7.32E-09
MIS12	MIS12 kinetochore complex component	protein_coding	chr17:5486285-	3157	5.790131736	3.939879803	-0.638	1.27E-09	7.35E-09
RAF1	Raf-1 proto-oncogene, serine/threonine k	protein_coding	chr3:12583601-	7847	24.98571681	20.47778131	-0.373	1.27E-09	7.35E-09
TTPAL	alpha tocopherol transfer protein like	protein_coding	chr20:44475874	7056	2.814198456	1.761577488	-0.763	1.27E-09	7.36E-09
PPFIBP1	PPFIA binding protein 1	protein_coding	chr12:27523431	11690	4.648340308	3.40855646	-0.532	1.28E-09	7.42E-09
MAP9	microtubule associated protein 9	protein_coding	chr4:155342658	8857	7.220802785	5.524936249	-0.473	1.28E-09	7.43E-09
ARHGAP29	Rho GTPase activating protein 29	protein_coding	chr1:94148988-	18370	1.614754112	1.191415088	-0.524	1.29E-09	7.44E-09
ADAM9	ADAM metallopeptidase domain 9	protein_coding	chr8:38996869- 7030		16.11278352	12.84509167	-0.414	1.29E-09	7.44E-09
LYN	LYN proto-oncogene, Src family tyrosine k	protein_coding	chr8:55879835-	6164	1.378211604	0.721630857	-1.028	1.29E-09	7.44E-09
PPP1R2	protein phosphatase 1 regulatory inhibitc	protein_coding	chr3:195514428	5123	14.4729675	11.15351672	-0.462	1.29E-09	7.44E-09
PRKCH	protein kinase C eta	protein_coding	chr14:61187559	8830	1.101810456	0.608820419	-0.944	1.29E-09	7.46E-09
HDHD2	haloacid dehalogenase like hydrolase dom	protein_coding	chr18:47107408	4679	2.929233065	1.494610364	-1.051	1.31E-09	7.52E-09
MAPK1	mitogen-activated protein kinase 1	protein_coding	chr22:21754500	11336	19.18856184	15.28426319	-0.414	1.33E-09	7.66E-09
NEU1	neuraminidase 1	protein_coding	chr6:31857659-	4822	8.624737729	6.073754721	-0.588	1.36E-09	7.79E-09
CDKN2C	cyclin dependent kinase inhibitor 2C	protein_coding	chr1:50960745-	3018	3.126081007	1.876874484	-0.825	1.36E-09	7.81E-09
MPV17L2	MPV17 mitochondrial inner membrane p	protein_coding	chr19:18193182	2135	18.28396156	11.38446704	-0.764	1.37E-09	7.82E-09
YIPF6	Yip1 domain family member 6	protein_coding	chrX:68498562-	8608	7.176831218	5.760238139	-0.404	1.38E-09	7.88E-09
TSPAN5	tetraspanin 5	protein_coding	chr4:98470367-	7412	5.855169446	3.970516663	-0.647	1.39E-09	7.93E-09
AP2A1	adaptor related protein complex 2 subuni	protein_coding	chr19:49766968	8746	16.46661536	13.16362545	-0.409	1.39E-09	7.95E-09
SNAP25	synaptosome associated protein 25	protein_coding	chr20:10218830	3357	1.17747347	0.441546855	-1.501	1.40E-09	7.97E-09
DLG2	discs large MAGUK scaffold protein 2	protein_coding	chr11:83455012	16709	1.047406122	0.58454348	-0.923	1.40E-09	7.99E-09
CHCHD4	coiled-coil-helix-coiled-coil-helix domain	protein_coding	chr3:14112077-	1772	10.38309572	7.051604035	-0.644	1.41E-09	8.06E-09
MIA3	MIA SH3 domain ER export factor 3	protein_coding	chr1:222618097	10155	7.575451005	5.919512763	-0.441	1.42E-09	8.06E-09
IFNGR1	interferon gamma receptor 1	protein_coding	chr6:137197484	3277	6.781471615	4.85600523	-0.569	1.42E-09	8.06E-09
CACNA2D1	calcium voltage-gated channel auxiliary s	protein_coding	chr7:81946444-	8989	17.24925679	13.76045082	-0.412	1.43E-09	8.15E-09
ERICD	E2F1-regulated inhibitor of cell death	lncRNA	chr8:140636281	2003	1.542736388	0.51532429	-1.662	1.46E-09	8.30E-09
TGFBRAP1	transforming growth factor beta receptor	protein_coding	chr2:105264391	5706	4.74766845	3.402021558	-0.565	1.48E-09	8.39E-09
KLHDC2	kelch domain containing 2	protein_coding	chr14:49768130	6319	15.38772582	12.14639333	-0.428	1.49E-09	8.45E-09
AGBL5	ATP/GTP binding protein like 5	protein_coding	chr2:27042364-	5283	5.104587997	3.658085561	-0.569	1.50E-09	8.51E-09
SDC1	syndecan 1	protein_coding	chr2:20200797-	3613	1.011792862	0.281067151	-1.92	1.50E-09	8.51E-09
SULT1A2	sulfotransferase family 1A member 2	protein_coding	chr16:28591943	2216	2.959260519	1.024090045	-1.628	1.54E-09	8.67E-09
POPDC2	popeye domain containing 2	protein_coding	chr3:119636457	2445	217.1696874	175.6902638	-0.392	1.55E-09	8.75E-09
UNKL	unk like zinc finger	protein_coding	chr16:1363205-	7494	2.618297379	1.695598586	-0.716	1.58E-09	8.90E-09
GFM2	G elongation factor mitochondrial 2	protein_coding	chr5:74721206-	3971	21.81746517	17.7539719	-0.384	1.59E-09	8.94E-09
SC5D	sterol-C5-desaturase	protein_coding	chr11:12129268	10054	6.329947701	4.308755725	-0.638	1.59E-09	8.94E-09
SLC25A40	solute carrier family 25 member 40	protein_coding	chr7:87833568-	5084	4.922606775	3.647023738	-0.518	1.59E-09	8.95E-09
TARS2	threonyl-tRNA synthetase 2, mitochondri	protein_coding	chr1:150487364	4077	8.121800123	6.179816488	-0.481	1.60E-09	8.96E-09
ROR2	receptor tyrosine kinase like orphan recep	protein_coding	chr9:91563091-	5880	12.32624766	9.222304463	-0.503	1.60E-09	8.97E-09
RAD18	RAD18 E3 ubiquitin protein ligase	protein_coding	chr3:8775402-8	6984	1.250821798	0.645080797	-1.038	1.60E-09	8.97E-09
ACOT11	acyl-CoA thioesterase 11	protein_coding	chr1:54542257-	9768	17.75701161	13.42879934	-0.488	1.61E-09	9.00E-09
TRIM5	tripartite motif containing 5	protein_coding	chr11:5663557-	4804	6.60184082	5.024542288	-0.48	1.61E-09	9.00E-09
PPP1R13B	protein phosphatase 1 regulatory subunit	protein_coding	chr14:10373319	9588	13.65806115	10.88645476	-0.414	1.62E-09	9.08E-09
GYS1	glycogen synthase 1	protein_coding	chr19:48968130	4241	82.1033991	59.2561153	-0.557	1.63E-09	9.11E-09
SOX11	SRY-box 11	protein_coding	chr2:5692384-5	9002	8.676631057	6.787898139	-0.44	1.65E-09	9.22E-09
MID1	midline 1	protein_coding	chrX:10445310-	9295	6.317632051	4.816394874	-0.477	1.66E-09	9.24E-09
ANK3	ankyrin 3	protein_coding	chr10:60026298	25759	6.411399319	4.749863071	-0.518	1.67E-09	9.30E-09

TOLLIP	toll interacting protein	protein_coding	chr11:1274371-	5983	12.00834338	9.538757427	-0.418	1.68E-09	9.33E-09
ZDHHC5	zinc finger DHHC-type containing 5	protein_coding	chr11:57667747	5424	13.51222332	10.23897329	-0.485	1.68E-09	9.33E-09
ZEB2	zinc finger E-box binding homeobox 2	protein_coding	chr2:144364364	24861	1.268760504	0.850492505	-0.665	1.68E-09	9.33E-09
ABCA8	ATP binding cassette subfamily A member	protein_coding	chr17:68867289	11246	3.087888754	2.026262837	-0.697	1.69E-09	9.41E-09
PRRC2B	proline rich coiled-coil 2B	protein_coding	chr9:131373636	12390	32.02734848	20.53504166	-0.723	1.72E-09	9.53E-09
PRDM16-DT	PRDM16 divergent transcript	lncRNA	chr1:3059615-3	5600	6.554134815	3.74059051	-0.89	1.72E-09	9.56E-09
MCC	MCC regulator of WNT signaling pathway	protein_coding	chr5:113022099	10739	8.808800688	6.353402674	-0.556	1.75E-09	9.72E-09
SMPX	small muscle protein X-linked	protein_coding	chrX:21705978-	1324	322.1887857	217.4438666	-0.651	1.76E-09	9.73E-09
SACM1L	SAC1 like phosphatidylinositide phosphat	protein_coding	chr3:45689056-	7337	6.857901947	5.28911482	-0.46	1.76E-09	9.74E-09
MICOS10	mitochondrial contact site and cristae org	protein_coding	chr1:19484403-	5480	1.478203398	0.612459757	-1.341	1.78E-09	9.83E-09
PDGFB	platelet derived growth factor subunit B	protein_coding	chr22:39223359	3761	1.026106605	0.402438658	-1.437	1.79E-09	9.86E-09
PRRG1	proline rich and Gla domain 1	protein_coding	chrX:37349275-	6180	2.695641061	1.901660931	-0.591	1.79E-09	9.89E-09
PTPN3	protein tyrosine phosphatase non-recepto	protein_coding	chr9:109375466	9995	0.980853743	0.569095087	-0.868	1.80E-09	9.91E-09
ERP44	endoplasmic reticulum protein 44	protein_coding	chr9:99979185-	4808	7.783985574	5.983707915	-0.465	1.81E-09	9.96E-09
PTS	6-pyruvoyltetrahydropterin synthase	protein_coding	chr11:11222636	3128	3.975275915	2.259655351	-0.904	1.82E-09	1.00E-08
GK	glycerol kinase	protein_coding	chrX:30653359-	5359	1.528927433	0.894979414	-0.859	1.84E-09	1.01E-08
LMTK2	lemur tyrosine kinase 2	protein_coding	chr7:98106862-	9167	8.638910338	6.137099887	-0.578	1.86E-09	1.02E-08
DDIT4	DNA damage inducible transcript 4	protein_coding	chr10:72273924	2054	9.593849352	2.69295379	-1.939	1.86E-09	1.02E-08
PHF24	PHD finger protein 24	protein_coding	chr9:34957608-	5813	2.14968257	1.194545498	-0.931	1.87E-09	1.03E-08
AC005747.1	growth arrest specific 7	protein_coding	chr17:9910609- 10100		3.135223532	2.087181329	-0.671	1.87E-09	1.03E-08
TIMP2	TIMP metallopeptidase inhibitor 2	protein_coding	chr17:78852977	5633	20.92490086	15.88366528	-0.483	1.90E-09	1.04E-08
FAM83D	family with sequence similarity 83 membe	protein_coding	chr20:38926312	2475	5.5013716	2.803062638	-1.058	1.91E-09	1.04E-08
CHP1	calcineurin like EF-hand protein 1	protein_coding	chr15:41230839	3947	32.15842497	25.33781751	-0.43	1.92E-09	1.05E-08
ARMC1	armadillo repeat containing 1	protein_coding	chr8:65602458-	3419	13.98960405	10.56922204	-0.49	1.94E-09	1.06E-08
ANO5	anoctamin 5	protein_coding	chr11:21799934	7547	13.19857291	10.5042331	-0.416	1.95E-09	1.06E-08
LPP-AS2	LPP antisense RNA 2	lncRNA	chr3:188151206	2852	4.780268043	2.586333019	-0.97	1.95E-09	1.07E-08
SOX9	SRY-box 9	protein_coding	chr17:72121020	3931	0.876463636	0.258258227	-1.837	1.96E-09	1.07E-08
KDM2A	lysine demethylase 2A	protein_coding	chr11:67119263	10381	9.553276215	7.12581083	-0.507	2.00E-09	1.09E-08
SYNGR1	synaptogyrin 1	protein_coding	chr22:39349925	6940	7.163158001	5.582604133	-0.446	2.03E-09	1.10E-08
MAP1LC3B	microtubule associated protein 1 light ch	protein_coding	chr16:87383953	5037	34.82248341	26.68425015	-0.469	2.04E-09	1.11E-08
ZNF655	zinc finger protein 655	protein_coding	chr7:99558406-	8401	8.203176292	6.610393016	-0.398	2.05E-09	1.11E-08
KIF26B	kinesin family member 26B	protein_coding	chr1:245154985	14404	30.22412526	21.09881249	-0.604	2.05E-09	1.12E-08
SGCA	sarcoglycan alpha	protein_coding	chr17:50164214	2449	12.60862004	9.157891632	-0.545	2.06E-09	1.12E-08
C17orf75	chromosome 17 open reading frame 75	protein_coding	chr17:32324565	5742	4.720414713	3.334390468	-0.587	2.08E-09	1.13E-08
SFRP5	secreted frizzled related protein 5	protein_coding	chr10:97766751	1883	1.929255531	0.846937028	-1.274	2.09E-09	1.13E-08
CYP7B1	cytochrome P450 family 7 subfamily B me	protein_coding	chr8:64587763-	7542	2.086821514	1.316765661	-0.746	2.10E-09	1.14E-08
ANK1	ankyrin 1	protein_coding	chr8:41653220-	10797	1.857739046	0.937921927	-1.068	2.12E-09	1.15E-08
ARF1	ADP ribosylation factor 1	protein_coding	chr1:228082660	3281	118.6047913	95.68560033	-0.396	2.17E-09	1.17E-08
DYRK1B	dual specificity tyrosine phosphorylation	protein_coding	chr19:39825350	3192	30.50564515	24.731279	-0.389	2.21E-09	1.19E-08
CDC6	cell division cycle 6	protein_coding	chr17:40287879	5311	3.838866376	2.595968033	-0.648	2.21E-09	1.19E-08
MKRN2	makorin ring finger protein 2	protein_coding	chr3:12557087-	2775	31.61420028	25.83127973	-0.377	2.22E-09	1.20E-08
ASB13	ankyrin repeat and SOCS box containing 1	protein_coding	chr10:5638867-	3003	4.265737115	2.757907541	-0.718	2.24E-09	1.21E-08
ADAMTS1	ADAM metallopeptidase with thrombosp	protein_coding	chr21:26835755	7055	14.72789012	10.46318624	-0.578	2.27E-09	1.22E-08
NAP1L3	nucleosome assembly protein 1 like 3	protein_coding	chrX:93670930-	2639	31.54289717	24.56977305	-0.447	2.28E-09	1.22E-08
HHAT	hedgehog acyltransferase	protein_coding	chr1:210328252	6645	2.318005151	1.534368546	-0.684	2.28E-09	1.22E-08
VSIR	V-set immunoregulatory receptor	protein_coding	chr10:71747556	5153	2.60005748	1.051910609	-1.388	2.29E-09	1.23E-08
RENBP	renin binding protein	protein_coding	chrX:153935269	2047	8.881660194	3.2621836	-1.514	2.31E-09	1.24E-08
RILPL1	Rab interacting lysosomal protein like 1	protein_coding	chr12:12347005	8671	13.51315512	10.96043767	-0.388	2.32E-09	1.24E-08
MECP2	methyl-CpG binding protein 2	protein_coding	chrX:154021573	18366	5.310086489	4.058464515	-0.473	2.34E-09	1.25E-08
PKP2	plakophilin 2	protein_coding	chr12:32790745	7043	60.69807703	49.08828012	-0.392	2.35E-09	1.26E-08
AAGAB	alpha and gamma adaptin binding protein	protein_coding	chr15:67201033	3833	8.723818475	6.223408552	-0.576	2.36E-09	1.26E-08
NOMO1	NODAL modulator 1	protein_coding	chr16:14833721	5776	18.02653875	14.36560189	-0.413	2.36E-09	1.26E-08
GYG1	glycogenin 1	protein_coding	chr3:148991341	3511	81.08764092	66.00362314	-0.383	2.38E-09	1.27E-08
PLPBP	pyridoxal phosphate binding protein	protein_coding	chr8:37762595-	3427	18.76801597	14.56947429	-0.451	2.40E-09	1.28E-08
SNX30	sorting nexin family member 30	protein_coding	chr9:112750760	11656	1.951969015	1.386712323	-0.577	2.42E-09	1.29E-08
DPP7	dipeptidyl peptidase 7	protein_coding	chr9:137110546	2823	10.98127368	7.919838779	-0.555	2.45E-09	1.30E-08
RIPOR1	RHO family interacting cell polarization re	protein_coding	chr16:67518418	6729	16.47147255	13.15374466	-0.41	2.46E-09	1.31E-08
BRINP1	BMP/retinoic acid inducible neural specif	protein_coding	chr9:119153458	3874	1.063312809	0.410644711	-1.455	2.49E-09	1.32E-08
SMIM5	small integral membrane protein 5	protein_coding	chr17:75633434	5117	0.93032792	0.322538007	-1.603	2.51E-09	1.33E-08
STX3	syntaxin 3	protein_coding	chr11:59713456	7308	6.883696339	4.994180168	-0.546	2.52E-09	1.33E-08
C2CD2L	C2CD2 like	protein_coding	chr11:11910219	9216	2.225162376	1.37682582	-0.777	2.52E-09	1.34E-08
COL13A1	collagen type XIII alpha 1 chain	protein_coding	chr10:69801867	5888	1.93044343	0.922293601	-1.145	2.53E-09	1.34E-08
TUBGCP4	tubulin gamma complex associated prote	protein_coding	chr15:43369221	11171	2.689919434	1.911209321	-0.578	2.58E-09	1.37E-08
CMIP	c-Maf inducing protein	protein_coding	chr16:81445170	12715	2.34402883	1.707231291	-0.542	2.59E-09	1.37E-08
SLF1	SMC5-SMC6 complex localization factor 1	protein_coding	chr5:94618669-	6863	4.416929255	3.191577587	-0.558	2.61E-09	1.38E-08
GNAI2	G protein subunit alpha i2	protein_coding	chr3:50226292-	7405	16.02174664	12.80881426	-0.409	2.62E-09	1.38E-08
NUDT5	nudix hydrolase 5	protein_coding	chr10:12165330	4964	6.631529117	5.001580545	-0.492	2.63E-09	1.38E-08
C11orf24	chromosome 11 open reading frame 24	protein_coding	chr11:68261338	3403	11.69485849	8.404794017	-0.562	2.64E-09	1.39E-08
SERINC2	serine incorporator 2	protein_coding	chr1:31409565-	3452	2.9050645	1.435601946	-1.094	2.64E-09	1.39E-08
CCNG2	cyclin G2	protein_coding	chr4:77157207-	7416	2.34549484	1.559754099	-0.675	2.65E-09	1.39E-08
COPZ1	coatomer protein complex subunit zeta 1	protein_coding	chr12:54301202	3898	20.25844054	15.26970845	-0.493	2.66E-09	1.40E-08
SNX16	sorting nexin 16	protein_coding	chr8:81799581-	4608	3.794623564	2.146571744	-0.91	2.66E-09	1.40E-08
NARS	asparaginyl-tRNA synthetase	protein_coding	chr18:57600656	4089	59.55400632	49.03344512	-0.366	2.67E-09	1.40E-08
TAP1	transporter 1, ATP binding cassette subfar	protein_coding	chr6:32845209-	4805	2.735977594	1.546652909	-0.907	2.67E-09	1.40E-08
B2M	beta-2-microglobulin	protein_coding	chr15:44711487	6079	35.08107156	25.78400934	-0.528	2.71E-09	1.42E-08
RECQL4	RecQ like helicase 4	protein_coding	chr8:144511288	4236	3.376827106	2.095372255	-0.775	2.72E-09	1.42E-08
CHCHD3	coiled-coil-helix-coiled-coil-helix domain	protein_coding	chr7:132784870	3428	36.10415257	26.80707475	-0.514	2.72E-09	1.42E-08
RYR2	ryanodine receptor 2	protein_coding	chr1:237042184	17855	39.9180122	24.65910442	-0.776	2.75E-09	1.44E-08
VEGFA	vascular endothelial growth factor A	protein_coding	chr6:43770184-	14431	21.45251029	13.48119152	-0.76	2.76E-09	1.44E-08
DTX3L	deltex E3 ubiquitin ligase 3L	protein_coding	chr3:122564338	5768	1.649808492	0.974090813	-0.842	2.80E-09	1.46E-08
NANOS1	nanos C2HC-type zinc finger 1	protein_coding	chr10:11902971	4017	1.079894256	0.336418392	-1.773	2.82E-09	1.47E-08
DCUN1D1	defective in cullin neddylation 1 domain c	protein_coding	chr3:182938074	8412	5.937222127	4.546427449	-0.472	2.86E-09	1.48E-08
APC	APC regulator of WNT signaling pathway	protein_coding	chr5:112707498	12440	4.117383345	3.313687494	-0.399	2.86E-09	1.48E-08
GINS1	GINS complex subunit 1	protein_coding	chr20:25407673	4130	2.465796974	1.549119249	-0.755	2.87E-09	1.48E-08
SH3GL3	SH3 domain containing GRB2 like 3, endo	protein_coding	chr15:83447228	3363	16.6996358	12.57806838	-0.497	2.89E-09	1.49E-08
PTPA	protein phosphatase 2 phosphatase activa	protein_coding	chr9:129110950	5624	38.99191152	31.23566863	-0.407	2.89E-09	1.50E-08
AP002761.3	novel transcript	lncRNA	chr11:73307235	1695	3.431915829	1.272348761	-1.513	2.93E-09	1.51E-08
EBF4	EBF family member 4	protein_coding	chr20:2692878-	3717	3.425587102	1.865468754	-0.969	2.94E-09	1.52E-08
RNF4	ring finger protein 4	protein_coding	chr4:2462220-2	7141	9.987773492	7.338864301	-0.528	2.96E-09	1.53E-08
CEBPD	CCAAT enhancer binding protein delta	protein_coding	chr8:47736909-	2178	10.36287836	7.411239535	-0.568	2.99E-09	1.54E-08
MPC1	mitochondrial pyruvate carrier 1	protein_coding	chr6:166364919	1546	91.24214527	63.53619222	-0.606	2.99E-09	1.54E-08
TBX3	T-box 3	protein_coding	chr12:11467025	5960	6.787599656	5.149442383	-0.484	3.00E-09	1.55E-08
SLC19A2	solute carrier family 19 member 2	protein_coding	chr1:169463909	4364	5.007673642	3.033962398	-0.801	3.02E-09	1.56E-08
CPVL	carboxypeptidase vitellogenic like	protein_coding	chr7:28995235-	3336	51.80646849	37.59089286	-0.548	3.04E-09	1.57E-08
IFI27L1	interferon alpha inducible protein 27 like	protein_coding	chr14:94081301	2356	5.249304785	3.292411381	-0.756	3.05E-09	1.57E-08
PANX2	pannexin 2	protein_coding	chr22:50170731	3102	3.408297562	2.075412384	-0.795	3.15E-09	1.62E-08
ZNF75D	zinc finger protein 75D	protein_coding	chrX:135248920	6838	3.562931809	2.702143836	-0.484	3.18E-09	1.64E-08
UBA7	ubiquitin like modifier activating enzyme	protein_coding	chr3:49805209-	4550	3.605120029	2.117036017	-0.849	3.20E-09	1.64E-08
XBP1	X-box binding protein 1	protein_coding	chr22:28794555	3028	26.99746681	20.905971	-0.455	3.20E-09	1.64E-08
DHX36	DEAH-box helicase 36	protein_coding	chr3:154272546	10552	8.358727889	6.732063581	-0.399	3.22E-09	1.65E-08
ZC3H12A	zinc finger CCCH-type containing 12A	protein_coding	chr1:37474580-	3091	1.650984706	0.873010332	-1.003	3.25E-09	1.66E-08
RALGAPA2	Ral GTPase activating protein catalytic alp	protein_coding	chr20:20389530	11649	3.17799936	2.166316878	-0.635	3.28E-09	1.68E-08
NUP188	nucleoporin 188	protein_coding	chr9:128947699	8041	9.391341174	6.860811457	-0.537	3.31E-09	1.69E-08
SUSD6	sushi domain containing 6	protein_coding	chr14:69611596	5832	12.25199396	8.968601931	-0.535	3.34E-09	1.71E-08
SLC35F6	solute carrier family 35 member F6	protein_coding	chr2:26764284-	3902	5.871654702	4.159427859	-0.584	3.35E-09	1.71E-08
AL132855.1	novel transcript, antisense to MYH7	lncRNA	chr14:23415339	207	37.63465496	18.35761617	-1.113	3.35E-09	1.71E-08
STARD7	StAR related lipid transfer domain contair	protein_coding	chr2:96184859-	4414	69.22529472	57.52322726	-0.353	3.37E-09	1.72E-08
ARHGEF40	Rho guanine nucleotide exchange factor 4	protein_coding	chr14:21070273	8194	5.068728705	3.571541829	-0.591	3.41E-09	1.74E-08
TEX2	testis expressed 2	protein_coding	chr17:64147227	9094	11.8953555	9.66093711	-0.386	3.49E-09	1.78E-08

SLC38A7	solute carrier family 38 member 7	protein_coding	chr16:5866510	6657	8.020634453	6.275830453	-0.439	3.53E-09	1.80E-08
DICER1	dicer 1, ribonuclease III	protein_coding	chr14:9508622	12294	9.50350114	7.267052059	-0.472	3.55E-09	1.81E-08
FAM95C	family with sequence similarity 95 memb	protein_coding	chr9:38540569-	3936	1.29110264	0.571394172	-1.265	3.57E-09	1.81E-08
INCENP	inner centromere protein	protein_coding	chr11:6212399	4454	4.725025036	2.939336071	-0.767	3.64E-09	1.85E-08
CNTROB	centrobin, centriole duplication and spin	protein_coding	chr17:7932101-	6234	3.555041705	2.649066485	-0.509	3.65E-09	1.85E-08
KLHL2	kelch like family member 2	protein_coding	chr4:16520756	3929	8.911352633	6.928765752	-0.45	3.69E-09	1.87E-08
ZFHX3	zinc finger homeobox 3	protein_coding	chr16:7278288	22347	2.240806428	1.276082348	-0.891	3.73E-09	1.89E-08
TMEM165	transmembrane protein 165	protein_coding	chr4:55395957-	7048	6.654633955	5.123849359	-0.464	3.74E-09	1.90E-08
WDR26	WD repeat domain 26	protein_coding	chr1:22438514	10976	14.27278818	11.67709613	-0.376	3.77E-09	1.91E-08
YIPF3	Yip1 domain family member 3	protein_coding	chr6:43511832-	3043	20.98169964	16.90667522	-0.399	3.81E-09	1.93E-08
CDCA3	cell division cycle associated 3	protein_coding	chr12:6844793-	5653	1.874891087	1.117223332	-0.835	3.87E-09	1.96E-08
MXD1	MAX dimerization protein 1	protein_coding	chr2:69897688-	6351	1.628452126	0.933413079	-0.886	3.88E-09	1.96E-08
NAV2	neuron navigator 2	protein_coding	chr11:1935072	15174	8.195281623	6.115114212	-0.508	3.90E-09	1.97E-08
PSMB5	proteasome subunit beta 5	protein_coding	chr14:2301654	1765	48.91603629	36.07015764	-0.526	3.90E-09	1.97E-08
DYRK1A	dual specificity tyrosine phosphorylation	protein_coding	chr21:3736557	26763	4.142549017	3.095737403	-0.506	3.96E-09	2.00E-08
SLC30A6	solute carrier family 30 member 6	protein_coding	chr2:32165841-	5410	6.635185198	5.213388197	-0.434	4.00E-09	2.02E-08
ATP6V1H	ATPase H+ transporting V1 subunit H	protein_coding	chr8:53715543-	5104	13.5766746	10.30291284	-0.484	4.04E-09	2.04E-08
AC105942.1	novel transcript	lncRNA	chr1:94927361-	2517	7.141321166	4.584915775	-0.726	4.06E-09	2.05E-08
FOXO1	forkhead box O1	protein_coding	chr13:4046995	6378	12.64470096	10.03703813	-0.419	4.09E-09	2.06E-08
TMCO4	transmembrane and coiled-coil domains	protein_coding	chr1:19682213-	4054	0.927334355	0.340998955	-1.524	4.12E-09	2.07E-08
CENPM	centromere protein M	protein_coding	chr22:4193873	1750	4.251627248	2.166543655	-1.062	4.21E-09	2.11E-08
UQCRH	ubiquinol-cytochrome c reductase hinge	protein_coding	chr1:46303698-	1341	172.0227786	121.8374458	-0.582	4.21E-09	2.11E-08
AC079834.2	novel transcript	lncRNA	chr2:22157250	1949	1.796853182	0.753087155	-1.336	4.22E-09	2.11E-08
IRF2	interferon regulatory factor 2	protein_coding	chr4:18438772 3070		5.724839257	3.896008921	-0.64	4.26E-09	2.13E-08
BX640514.1	novel transcript	lncRNA	chr20:6257559	1918	4.299307123	2.242257808	-1.023	4.29E-09	2.14E-08
C11orf21	chromosome 11 open reading frame 21	protein_coding	chr11:2295645-	4199	3.561261443	0.506302198	-2.872	4.33E-09	2.16E-08
EIF3K	eukaryotic translation initiation factor 3	protein_coding	chr19:3861908	2798	74.52213925	50.06140065	-0.656	4.34E-09	2.16E-08
ADAMTS12	ADAM metallopeptidase with thrombosp	protein_coding	chr5:33523535-	9605	2.712121296	1.917477042	-0.588	4.44E-09	2.21E-08
USP46	ubiquitin specific peptidase 46	protein_coding	chr4:52590960-	8895	10.68213303	8.458458164	-0.422	4.46E-09	2.22E-08
VARS	valyl-tRNA synthetase	protein_coding	chr6:31777518-	5880	15.25235998	12.65978955	-0.355	4.45E-09	2.22E-08
PDE1C	phosphodiesterase 1C	protein_coding	chr7:31751179-	13821	6.047888446	4.427294202	-0.535	4.47E-09	2.22E-08
YPEL5	yippee like 5	protein_coding	chr2:30146941-	3421	24.64997454	19.40002261	-0.431	4.46E-09	2.22E-08
NCR3LG1	natural killer cell cytotoxicity receptor 3	protein_coding	chr11:1735172	6438	3.375961937	2.021927673	-0.819	4.48E-09	2.23E-08
MYBL1	MYB proto-oncogene like 1	protein_coding	chr8:66562175-	7239	1.879920744	1.176802461	-0.755	4.48E-09	2.23E-08
STAT4	signal transducer and activator of transcri	protein_coding	chr2:19102957	4606	6.780786515	4.796296825	-0.586	4.48E-09	2.23E-08
AC104825.1	novel transcript	lncRNA	chr4:8355090-8	3249	3.3147762	2.006593152	-0.811	4.49E-09	2.23E-08
SPDL1	spindle apparatus coiled-coil protein 1	protein_coding	chr5:16958363	4775	3.29759377	2.16321565	-0.694	4.50E-09	2.24E-08
CSF1	colony stimulating factor 1	protein_coding	chr1:10991024	5238	8.072381268	6.033533416	-0.504	4.54E-09	2.25E-08
ZC3H15	zinc finger CCCH-type containing 15	protein_coding	chr2:18648625	3026	23.95868702	17.1781545	-0.563	4.56E-09	2.26E-08
PDE6B	phosphodiesterase 6B	protein_coding	chr4:625584-67	5086	1.30179363	0.559877115	-1.299	4.61E-09	2.28E-08
MED28	mediator complex subunit 28	protein_coding	chr4:17614641-	10972	4.019637235	3.080301497	-0.47	4.62E-09	2.29E-08
SGMS1	sphingomyelin synthase 1	protein_coding	chr10:5030558	6423	7.125837588	5.761814833	-0.394	4.64E-09	2.30E-08
KIF20A	kinesin family member 20A	protein_coding	chr5:13817871	3852	9.446546657	6.851298819	-0.55	4.65E-09	2.30E-08
TLE2	TLE family member 2, transcriptional core	protein_coding	chr19:2997639-	4150	10.99493273	8.330048768	-0.487	4.68E-09	2.31E-08
DDR1	discoidin domain receptor tyrosine kinas	protein_coding	chr6:30876421-	9122	17.23333277	12.9904039	-0.493	4.68E-09	2.31E-08
GSTO1	glutathione S-transferase omega 1	protein_coding	chr10:1042353	1691	30.46685278	21.293388	-0.603	4.70E-09	2.32E-08
HACD2	3-hydroxyacyl-CoA dehydratase 2	protein_coding	chr3:12349082	5219	9.646249138	7.711349726	-0.409	4.71E-09	2.32E-08
SLC1A3	solute carrier family 1 member 3	protein_coding	chr5:36606355-	6265	15.17924822	10.77365989	-0.58	4.76E-09	2.35E-08
CELF1	CUGBP Elav-like family member 1	protein_coding	chr11:4746593	10452	17.26816647	13.60299368	-0.43	4.79E-09	2.36E-08
B4GALT4	beta-1,4-galactosyltransferase 4	protein_coding	chr3:11921173	6988	2.21269922	1.515431565	-0.633	4.81E-09	2.37E-08
KMT5B	lysine methyltransferase 5B	protein_coding	chr11:6815486	8632	6.95873025	5.420352392	-0.447	4.84E-09	2.38E-08
CRY1	cryptochrome circadian regulator 1	protein_coding	chr12:1069913	4782	4.070098846	2.943775122	-0.555	4.84E-09	2.38E-08
PEX1	peroxisomal biogenesis factor 1	protein_coding	chr7:92487020-	7838	4.318498925	3.160628984	-0.539	4.88E-09	2.40E-08
PNRC2	proline rich nuclear receptor coactivator	protein_coding	chr1:23956839-	2763	19.21725059	13.56407893	-0.585	4.90E-09	2.40E-08
SORT1	sortilin 1	protein_coding	chr1:10930956	9127	12.29523176	9.208986144	-0.502	4.90E-09	2.40E-08
DCAF8	DDB1 and CUL4 associated factor 8	protein_coding	chr1:16021571	7587	9.496070985	7.723616157	-0.384	4.95E-09	2.43E-08
VPS45	vacuolar protein sorting 45 homolog	protein_coding	chr1:15006727	6637	9.599542865	7.70309983	-0.405	4.99E-09	2.44E-08
CHDH	choline dehydrogenase	protein_coding	chr3:53812335-	7787	1.244958327	0.748350086	-0.814	5.01E-09	2.45E-08
ATG16L2	autophagy related 16 like 2	protein_coding	chr11:7281440	8519	2.095092111	1.106093443	-1.002	5.03E-09	2.46E-08
PSMD11	proteasome 26S subunit, non-ATPase 11	protein_coding	chr17:3244437	7083	11.05618089	9.106399768	-0.366	5.06E-09	2.47E-08
ACOX1	acyl-CoA oxidase 1	protein_coding	chr17:7594150	8286	10.19298704	8.231942077	-0.394	5.07E-09	2.47E-08
KCNIP4	potassium voltage-gated channel interact	protein_coding	chr4:20728606-	4239	1.245263451	0.566404972	-1.212	5.07E-09	2.47E-08
SLC4A1AP	solute carrier family 4 member 1 adaptor	protein_coding	chr2:27663471-	5207	7.102394725	5.441462777	-0.471	5.08E-09	2.48E-08
CREB3L1	cAMP responsive element binding protei	protein_coding	chr11:4627766	4037	0.945958331	0.375535488	-1.422	5.09E-09	2.48E-08
ODF2L	outer dense fiber of sperm tails 2 like	protein_coding	chr1:86346824-	10898	3.096928072	2.381933847	-0.466	5.10E-09	2.48E-08
PPP1R13L	protein phosphatase 1 regulatory subunit	protein_coding	chr19:4537963	5028	12.21671567	7.621511229	-0.766	5.11E-09	2.49E-08
TNS2	tensin 2	protein_coding	chr12:5304696	8093	2.543803142	1.371625855	-0.974	5.15E-09	2.50E-08
WARS	tryptophanyl-tRNA synthetase	protein_coding	chr14:1003337	6356	7.191025432	5.397570595	-0.499	5.17E-09	2.51E-08
TTI2	TELO2 interacting protein 2	protein_coding	chr8:33473386-	4222	4.019420874	2.952021952	-0.532	5.17E-09	2.51E-08
ABCD1	ATP binding cassette subfamily D member	protein_coding	chrX:1537248	4238	10.51018043	7.957129154	-0.488	5.21E-09	2.52E-08
TBC1D7	TBC1 domain family member 7	protein_coding	chr6:13266542-	8300	3.696643577	2.228477977	-0.813	5.32E-09	2.57E-08
ACSL3	acyl-CoA synthetase long chain family me	protein_coding	chr2:22286094	7232	15.40968635	12.82571844	-0.351	5.39E-09	2.61E-08
TMX4	thioredoxin related transmembrane prot	protein_coding	chr20:7977346-	6557	22.11161943	18.43563758	-0.348	5.41E-09	2.62E-08
NFKBIZ	NFKB inhibitor zeta	protein_coding	chr3:10182799	5902	1.637751766	1.088797257	-0.678	5.44E-09	2.63E-08
NUDCD2	NudC domain containing 2	protein_coding	chr5:16344652	8809	2.555989911	1.830388851	-0.572	5.44E-09	2.63E-08
AKNA	AT-hook transcription factor	protein_coding	chr9:11433415	10601	1.280955973	0.7405393	-0.869	5.46E-09	2.63E-08
VCP	valosin containing protein	protein_coding	chr9:35056064-	4816	80.08650429	67.04586858	-0.342	5.46E-09	2.64E-08
AP1G1	adaptor related protein complex 1 subun	protein_coding	chr16:7172900 10980		7.026758007	5.312213252	-0.491	5.50E-09	2.65E-08
PBK	PDZ binding kinase	protein_coding	chr8:27809624-	2148	9.924635867	6.290888671	-0.742	5.59E-09	2.69E-08
PDXK	pyridoxal kinase	protein_coding	chr21:4371909	11912	8.384892474	6.857159005	-0.376	5.62E-09	2.70E-08
S100A10	S100 calcium binding protein A10	protein_coding	chr1:15198291	1012	12.10267347	5.779408181	-1.149	5.63E-09	2.71E-08
ID2	inhibitor of DNA binding 2	protein_coding	chr2:8678845-8	2213	31.48662924	20.22830782	-0.718	5.72E-09	2.74E-08
PDZD2	PDZ domain containing 2	protein_coding	chr5:31639131-	15922	4.298337087	3.335128642	-0.451	5.73E-09	2.75E-08
TYMS	thymidylate synthetase	protein_coding	chr18:657653-6	2421	31.25797851	22.90066724	-0.533	5.76E-09	2.76E-08
BTF3L4	basic transcription factor 3 like 4	protein_coding	chr1:52056199-	5948	11.08464064	8.464805241	-0.475	5.78E-09	2.77E-08
STAP2	signal transducing adaptor family membe	protein_coding	chr19:4324043-	2766	3.948248038	1.918253253	-1.124	5.78E-09	2.77E-08
MTREX	Mtr4 exosome RNA helicase	protein_coding	chr5:55307989-	6347	13.83347509	11.28130881	-0.381	5.79E-09	2.77E-08
FAM124A	family with sequence similarity 124 mem	protein_coding	chr13:5122233	6702	2.588513788	1.7420814	-0.657	5.86E-09	2.80E-08
ST3GAL6	ST3 beta-galactoside alpha-2,3-sialyltrans	protein_coding	chr3:98732236-	6288	8.903774457	7.129961346	-0.407	5.89E-09	2.82E-08
TMEM51	transmembrane protein 51	protein_coding	chr1:15152532-	2238	5.128667754	3.304240132	-0.722	5.94E-09	2.84E-08
PSMG3-AS1	PSMG3 antisense RNA 1 (head to head)	lncRNA	chr7:1570073-1	6436	3.330077309	2.166456907	-0.705	6.13E-09	2.92E-08
IRX6	iroquois homeobox 6	protein_coding	chr16:5532376	3399	1.047160469	0.484141868	-1.197	6.17E-09	2.94E-08
CTNNA3	catenin alpha 3	protein_coding	chr10:6591252	11157	12.79483813	9.330290977	-0.54	6.23E-09	2.96E-08
ASAP1	ArfGAP with SH3 domain, ankyrin repeat	protein_coding	chr8:13005210	8294	15.88965555	11.88223749	-0.505	6.25E-09	2.97E-08
FOXO4	forkhead box O4	protein_coding	chrX:71096197-	3447	6.671611614	4.588000711	-0.624	6.30E-09	2.99E-08
SAE1	SUMO1 activating enzyme subunit 1	protein_coding	chr19:4711327	3742	32.43673663	26.93918194	-0.354	6.34E-09	3.01E-08
PDGFC	platelet derived growth factor C	protein_coding	chr4:15676045	5998	3.314431106	2.14399131	-0.725	6.36E-09	3.02E-08
ZFYVE27	zinc finger FYVE-type containing 27	protein_coding	chr10:9773712	3899	11.67492396	8.476296954	-0.549	6.38E-09	3.02E-08
SLC4A7	solute carrier family 4 member 7	protein_coding	chr3:27372721-	9616	14.50757651	11.90302075	-0.371	6.49E-09	3.07E-08
COL7A1	collagen type VII alpha 1 chain	protein_coding	chr3:48564073-	11071	16.53853019	7.389038932	-1.243	6.56E-09	3.10E-08
ZNF703	zinc finger protein 703	protein_coding	chr8:37695782-	3316	7.45409242	5.208213228	-0.601	6.62E-09	3.13E-08
GOT1	glutamic-oxaloacetic transaminase 1	protein_coding	chr10:9939687	2424	199.9967208	155.5915188	-0.447	6.63E-09	3.14E-08
GPAA1	glycosylphosphatidylinositol anchor atta	protein_coding	chr8:14408259	3268	28.31963988	23.28956581	-0.367	6.66E-09	3.14E-08
NOS1AP	nitric oxide synthase 1 adaptor protein	protein_coding	chr1:16206969	7854	1.57509485	0.827857102	-1.008	6.81E-09	3.21E-08
IRF1	interferon regulatory factor 1	protein_coding	chr5:13248160	7778	1.315889527	0.848932442	-0.714	6.85E-09	3.23E-08
RFX7	regulatory factor X7	protein_coding	chr15:5608728	10588	2.400113012	1.641351876	-0.631	6.88E-09	3.24E-08
SAP130	Sin3A associated protein 130	protein_coding	chr2:12794121	4373	7.109671227	5.388633627	-0.485	6.95E-09	3.27E-08
MPC2	mitochondrial pyruvate carrier 2	protein_coding	chr1:16791667	2638	63.85382235	43.57393768	-0.635	7.02E-09	3.30E-08
SPX	spexin hormone	protein_coding	chr12:2152629	7242	3.171162086	2.309791786	-0.545	7.02E-09	3.30E-08

HES6	hes family bHLH transcription factor 6	protein_coding	chr2:238238267	1954	3.218418717	1.139945278	-1.574	7.09E-09	3.33E-08
SLC25A45	solute carrier family 25 member 45	protein_coding	chr11:65375192	7855	1.204705631	0.767980665	-0.736	7.22E-09	3.39E-08
TRMT9B	tRNA methyltransferase 9B (putative)	protein_coding	chr8:12945642-	14669	5.637768313	4.467204998	-0.421	7.22E-09	3.39E-08
RETREG1	reticulophagy regulator 1	protein_coding	chr5:16473038-	4568	17.84841703	13.74315491	-0.462	7.24E-09	3.40E-08
TNS1	tensin 1	protein_coding	chr2:21779958	16281	33.16059817	23.58643653	-0.575	7.25E-09	3.40E-08
KLHL8	kelch like family member 8	protein_coding	chr4:87160103-	7354	2.575174888	1.771184025	-0.628	7.60E-09	3.55E-08
COL1A1	collagen type I alpha 1 chain	protein_coding	chr17:50183289	9819	4.701897304	3.494754693	-0.512	7.63E-09	3.56E-08
RASL10B	RAS like family 10 member B	protein_coding	chr17:35731649	3502	29.31799058	24.26890215	-0.359	7.65E-09	3.57E-08
HMMR	hyaluronan mediated motility receptor	protein_coding	chr5:16346020	3932	4.328600224	3.009321898	-0.609	7.76E-09	3.62E-08
UQCR11	ubiquinol-cytochrome c reductase, comp	protein_coding	chr19:1597169-	2137	12.61247174	9.084586946	-0.558	7.80E-09	3.64E-08
C16orf70	chromosome 16 open reading frame 70	protein_coding	chr16:67109958	5965	3.087468846	2.156624533	-0.605	7.82E-09	3.65E-08
DDX19A	DEAD-box helicase 19A	protein_coding	chr16:70346861	7847	2.002563035	1.411949601	-0.59	7.84E-09	3.65E-08
CTNND2	catenin delta 2	protein_coding	chr5:10971836-	7270	11.222685	8.493465224	-0.487	7.90E-09	3.67E-08
COQ8A	coenzyme Q8A	protein_coding	chr1:22689753€	7797	9.522801101	7.96635277	-0.344	7.93E-09	3.69E-08
AC010136.1	novel transcript	lncRNA	chr2:217978707 2060		1.904412776	0.776195444	-1.369	7.95E-09	3.69E-08
ZMPSTE24	zinc metallopeptidase STE24	protein_coding	chr1:40258236-	3439	6.866587912	5.071125223	-0.524	8.10E-09	3.76E-08
CCNJL	cyclin J like	protein_coding	chr5:16024910€	9209	3.577246098	2.847986304	-0.415	8.22E-09	3.81E-08
MAPK4	mitogen-activated protein kinase 4	protein_coding	chr18:50560087	7594	8.260564054	5.54264476	-0.658	8.37E-09	3.87E-08
PTTG1	PTTG1 regulator of sister chromatid separ	protein_coding	chr5:16042185	1975	13.67936928	8.602005819	-0.75	8.42E-09	3.90E-08
MFSD3	major facilitator superfamily domain con	protein_coding	chr8:14450907C	1827	23.48193489	17.33285424	-0.524	8.71E-09	4.02E-08
CNKSR3	CNKSR family member 3	protein_coding	chr6:15438751	21579	3.107592434	2.50085268	-0.4	8.74E-09	4.03E-08
POM121C	POM121 transmembrane nucleoporin C	protein_coding	chr7:75416786-	8796	7.436520474	5.843601401	-0.432	8.76E-09	4.04E-08
CCDC121	coiled-coil domain containing 121	protein_coding	chr2:27625639-	2844	1.987287319	0.99689634	-1.082	8.77E-09	4.05E-08
ANKRD46	ankyrin repeat domain 46	protein_coding	chr8:100509752	5820	6.670261131	4.885328759	-0.538	8.94E-09	4.12E-08
IRS1	insulin receptor substrate 1	protein_coding	chr2:22673131	9953	7.472637435	4.800235063	-0.72	9.02E-09	4.16E-08
PRPSAP1	phosphoribosyl pyrophosphate synthetas	protein_coding	chr17:76309486	6939	5.232666301	4.037785619	-0.458	9.06E-09	4.17E-08
PYGL	glycogen phosphorylase L	protein_coding	chr14:50857891	4546	1.491659845	0.834251135	-0.918	9.07E-09	4.18E-08
MEIS1	Meis homeobox 1	protein_coding	chr2:66433452-	10942	3.321465428	2.487414905	-0.502	9.12E-09	4.20E-08
MGST2	microsomal glutathione S-transferase 2	protein_coding	chr4:139665768	3172	13.12081616	9.268534869	-0.585	9.16E-09	4.21E-08
FAHD1	fumarylacetoacetate hydrolase domain cc	protein_coding	chr16:1826941-	3039	23.68754593	17.61482573	-0.513	9.21E-09	4.24E-08
FCHO2	FCH domain only 2	protein_coding	chr5:72956041-	6067	3.125712618	2.305966975	-0.526	9.25E-09	4.25E-08
RFLNB	refilin B	protein_coding	chr17:439978-4	3621	1.60141373	0.760164303	-1.16	9.31E-09	4.28E-08
PLXNA3	plexin A3	protein_coding	chrX:154458281	11710	4.547143782	2.997810365	-0.682	9.33E-09	4.28E-08
TGFBR2	transforming growth factor beta receptor	protein_coding	chr3:30606502-	4704	13.52599044	10.50652366	-0.45	9.34E-09	4.28E-08
VPS37B	VPS37B subunit of ESCRT-I	protein_coding	chr12:12286533	5634	10.92999028	8.831512264	-0.394	9.42E-09	4.32E-08
ACVR2A	activin A receptor type 2A	protein_coding	chr2:147844517	6448	2.894058889	2.119862527	-0.534	9.55E-09	4.37E-08
CHAC1	ChaC glutathione specific gamma-glutamy	protein_coding	chr15:40952962	2051	3.282167821	1.538262777	-1.165	9.61E-09	4.40E-08
CTNS	cystinosin, lysosomal cystine transporter	protein_coding	chr17:3636468-	3398	6.692064774	4.338830929	-0.703	9.65E-09	4.41E-08
ANKRD28	ankyrin repeat domain 28	protein_coding	chr3:15667236-	11276	2.313379338	1.744031797	-0.496	9.68E-09	4.42E-08
LIMK1	LIM domain kinase 1	protein_coding	chr7:74082933-	7038	3.838602521	2.689080008	-0.596	9.69E-09	4.42E-08
WNT11	Wnt family member 11	protein_coding	chr11:76186325	2371	5.070364178	2.979191233	-0.849	9.78E-09	4.46E-08
TOB1	transducer of ERBB2, 1	protein_coding	chr17:50862223	2521	22.57754099	16.73692563	-0.516	9.79E-09	4.47E-08
PPP4R3B	protein phosphatase 4 regulatory subunit	protein_coding	chr2:55547292-	8217	5.630816523	4.515745698	-0.404	9.81E-09	4.47E-08
UBR2	ubiquitin protein ligase E3 component n-	protein_coding	chr6:42564062-	8840	8.858737238	6.974750992	-0.43	9.85E-09	4.49E-08
PAK4	p21 (RAC1) activated kinase 4	protein_coding	chr19:39125770	7115	13.92329659	11.17108577	-0.403	9.92E-09	4.51E-08
AMPH	amphiphysin	protein_coding	chr7:38383704-	5489	1.502068556	0.814992565	-0.957	9.94E-09	4.52E-08
MAP7D1	MAP7 domain containing 1	protein_coding	chr1:36155579-	5271	60.51474111	50.94899982	-0.335	1.02E-08	4.62E-08
ANKRD2	ankyrin repeat domain 2	protein_coding	chr10:97572499	1451	1.116434382	0.141536551	-3.057	1.02E-08	4.63E-08
ARHGEF37	Rho guanine nucleotide exchange factor 3	protein_coding	chr5:149551947	5299	2.980547038	1.591871782	-0.984	1.03E-08	4.65E-08
USP21	ubiquitin specific peptidase 21	protein_coding	chr1:16115945C	3764	5.565316995	3.95192034	-0.578	1.03E-08	4.67E-08
AL451069.1	novel transcript	lncRNA	chr10:13250839	2466	9.914040182	7.788880167	-0.434	1.04E-08	4.68E-08
USH1C	USH1 protein network component harmo	protein_coding	chr11:17493895	4904	1.640619974	0.743693912	-1.215	1.04E-08	4.68E-08
KIAA0232	KIAA0232	protein_coding	chr4:6781375-6	8499	26.15620704	21.19247769	-0.389	1.04E-08	4.69E-08
MZF1-AS1	MZF1 antisense RNA 1	lncRNA	chr19:58559129	3838	1.699323004	1.021220221	-0.824	1.06E-08	4.76E-08
RCL1	RNA terminal phosphate cyclase like 1	protein_coding	chr9:4792944-4	4140	2.177118099	1.459340663	-0.664	1.06E-08	4.79E-08
EIF1B	eukaryotic translation initiation factor 1E	protein_coding	chr3:40309707-	1889	102.8953556	81.02893752	-0.43	1.08E-08	4.85E-08
PDCD10	programmed cell death 10	protein_coding	chr3:16768329E	3275	11.55440187	7.881005673	-0.637	1.08E-08	4.85E-08
GNAI3	G protein subunit alpha i3	protein_coding	chr1:10954861	27173	1.155403505	0.902434352	-0.444	1.08E-08	4.87E-08
ASXL2	ASXL transcriptional regulator 2	protein_coding	chr2:25733753-	13023	5.250015769	3.978507103	-0.484	1.09E-08	4.91E-08
SMG7	SMG7 nonsense mediated mRNA decay fa	protein_coding	chr1:18347221€	8317	13.35765469	10.57103231	-0.423	1.10E-08	4.92E-08
CYLD	CYLD lysine 63 deubiquitinase	protein_coding	chr16:5074205C	12954	2.345614145	1.86612163	-0.417	1.11E-08	4.96E-08
FBXO8	F-box protein 8	protein_coding	chr4:174236658	2810	7.571358327	5.701660598	-0.497	1.11E-08	4.96E-08
SGCG	sarcoglycan gamma	protein_coding	chr13:23180952	1624	3.063790889	1.243441798	-1.405	1.11E-08	4.96E-08
NEK6	NIMA related kinase 6	protein_coding	chr9:12425760€	4419	2.749561586	1.736872159	-0.752	1.11E-08	4.97E-08
RNF219	ring finger protein 219	protein_coding	chr13:78614289	3507	8.359694843	6.571572456	-0.435	1.12E-08	5.03E-08
ATP6V0E2-AS1	ATP6V0E2 antisense RNA 1	lncRNA	chr7:149867697	3291	3.26819273	2.14561651	-0.698	1.13E-08	5.05E-08
ZNRF1	zinc and ring finger 1	protein_coding	chr16:7499903C	8824	8.664090466	6.924139721	-0.41	1.14E-08	5.10E-08
SLC25A10	solute carrier family 25 member 10	protein_coding	chr17:81712236	2621	1.981115974	0.958839191	-1.145	1.15E-08	5.13E-08
PUM1	pumilio RNA binding family member 1	protein_coding	chr1:30931506-	6621	18.89457023	15.7751204	-0.346	1.17E-08	5.23E-08
GLA	galactosidase alpha	protein_coding	chrX:101397803	2275	2.305261648	1.015071549	-1.257	1.18E-08	5.25E-08
BMI1	BMI1 proto-oncogene, polycomb ring fing	protein_coding	chr10:22321099	4395	5.144359312	3.757156251	-0.541	1.19E-08	5.29E-08
AL118511.1	novel transcript	lncRNA	chr1:23086825S	1944	2.833618365	1.648674272	-0.867	1.19E-08	5.31E-08
TNNI1	troponin I1, slow skeletal type	protein_coding	chr1:201403768	7524	413.7635034	330.5854046	-0.409	1.20E-08	5.32E-08
ASB2	ankyrin repeat and SOCS box containing 2	protein_coding	chr14:93934153 5040		82.79147746	69.56761007	-0.337	1.20E-08	5.32E-08
DARS	aspartyl-tRNA synthetase	protein_coding	chr2:135905881	4752	12.65708071	9.554718031	-0.492	1.22E-08	5.41E-08
RGS5	regulator of G protein signaling 5	protein_coding	chr1:163111121	6624	69.80600047	57.3611062	-0.369	1.24E-08	5.49E-08
MB	myoglobin	protein_coding	chr22:35606764	2136	68.79533935	53.4519453	-0.45	1.24E-08	5.51E-08
PBLD	phenazine biosynthesis like protein doma	protein_coding	chr10:6828266C	5172	2.368118479	1.437664432	-0.801	1.28E-08	5.65E-08
DHRS7B	dehydrogenase/reductase 7B	protein_coding	chr17:21123364	5544	1.850940678	1.158076033	-0.759	1.30E-08	5.72E-08
MAPKAPK3	mitogen-activated protein kinase-activate	protein_coding	chr3:50611520-	3915	36.45313537	29.7304388	-0.38	1.30E-08	5.74E-08
NFATC2	nuclear factor of activated T cells 2	protein_coding	chr20:51386957	8286	2.408666099	1.678866368	-0.603	1.30E-08	5.74E-08
HARS2	histidyl-tRNA synthetase 2, mitochondria	protein_coding	chr5:14069143C	3719	8.628105817	6.743872933	-0.443	1.36E-08	5.97E-08
RIMS4	regulating synaptic membrane exocytosis	protein_coding	chr20:44751808	5206	13.17282838	9.314777848	-0.586	1.36E-08	5.99E-08
MIER3	MIER family member 3	protein_coding	chr5:56919602-	5763	5.331794238	3.988537134	-0.507	1.37E-08	6.02E-08
ATP13A2	ATPase cation transporting 13A2	protein_coding	chr1:16985958-	5253	7.127487607	5.352033534	-0.498	1.41E-08	6.18E-08
CERS2	ceramide synthase 2	protein_coding	chr1:15096058E	4644	21.41444193	17.83525998	-0.35	1.41E-08	6.18E-08
AC111170.1	novel transcript, antisense to SEPT9	lncRNA	chr17:77469162	1132	1.072913272	0.214605233	-2.38	1.42E-08	6.21E-08
TMEM120A	transmembrane protein 120A	protein_coding	chr7:75986831-	2000	14.81920353	11.20661959	-0.488	1.42E-08	6.22E-08
LINC01186	long intergenic non-protein coding RNA 1	lncRNA	chrX:46256759-	2526	1.009116319	0.254764489	-2.116	1.42E-08	6.23E-08
SPATA20	spermatogenesis associated 20	protein_coding	chr17:50543058	5337	15.63097868	12.46441823	-0.413	1.42E-08	6.23E-08
ZNF697	zinc finger protein 697	protein_coding	chr1:119619377	5579	2.907306368	1.847059902	-0.736	1.43E-08	6.24E-08
SLC25A25	solute carrier family 25 member 25	protein_coding	chr9:128068201	4936	2.630951184	1.852636886	-0.595	1.43E-08	6.26E-08
DNAJC27	DnaJ heat shock protein family (Hsp40) m	protein_coding	chr2:24943636-	5624	4.007020753	2.996645026	-0.501	1.45E-08	6.32E-08
OS9	OS9 endoplasmic reticulum lectin	protein_coding	chr12:57693955	5169	19.35917219	16.2257139	-0.341	1.45E-08	6.33E-08
PHYHD1	phytanoyl-CoA dioxygenase domain conta	protein_coding	chr9:12892096€	2844	3.252818516	2.125796337	-0.705	1.45E-08	6.33E-08
OPA3	OPA3 outer mitochondrial membrane lipi	protein_coding	chr19:45527427	9999	8.626587439	6.837842895	-0.421	1.46E-08	6.36E-08
RNMT	RNA guanine-7 methyltransferase	protein_coding	chr18:1372666C	6982	10.22117815	7.978518497	-0.441	1.46E-08	6.36E-08
DVL3	dishevelled segment polarity protein 3	protein_coding	chr3:184155377	6302	36.78070185	29.28221994	-0.414	1.47E-08	6.37E-08
MCF2L-AS1	MCF2L antisense RNA 1	lncRNA	chr13:11296748	712	8.89009841	4.178851121	-1.176	1.48E-08	6.43E-08
GRK4	G protein-coupled receptor kinase 4	protein_coding	chr4:2963571-3	4736	3.794309381	2.764745013	-0.546	1.49E-08	6.47E-08
AMHR2	anti-Mullerian hormone receptor type 2	protein_coding	chr12:53423855	3656	1.5740339	0.413607925	-1.992	1.51E-08	6.52E-08
PARP12	poly(ADP-ribose) polymerase family meml	protein_coding	chr7:140023744	6285	2.278130913	1.305056009	-0.89	1.52E-08	6.59E-08
BTBD6	BTB domain containing 6	protein_coding	chr14:10524849	2392	11.2352945	8.777413551	-0.443	1.54E-08	6.66E-08
GADD45B	growth arrest and DNA damage inducible	protein_coding	chr19:2476122-	2138	19.2304536	14.15255909	-0.53	1.55E-08	6.70E-08
SASS6	SAS-6 centriolar assembly protein	protein_coding	chr1:10008356	3972	1.529290651	0.901686259	-0.845	1.58E-08	6.80E-08
NATD1	N-acetyltransferase domain containing 1	protein_coding	chr17:2123887C	4931	9.886037209	7.730060051	-0.442	1.61E-08	6.91E-08
PSKH1	protein serine kinase H1	protein_coding	chr16:67893254	3993	24.02320126	20.40172966	-0.322	1.61E-08	6.91E-08
PGP	phosphoglycolate phosphatase	protein_coding	chr16:2211997-	2727	23.62247006	18.83517779	-0.412	1.61E-08	6.92E-08
SH3BP5L	SH3 binding domain protein 5 like	protein_coding	chr1:24881044€	5724	10.07538309	8.507554463	-0.33	1.62E-08	6.95E-08

TCEA1	transcription elongation factor A1	protein_coding	chr8:53966552-	4342	13.73792225	10.90055946	-0.419	1.64E-08	7.03E-08
FASTKD1	FAST kinase domains 1	protein_coding	chr2:169528508	5692	21.43931481	17.54963546	-0.375	1.66E-08	7.11E-08
FABP3	fatty acid binding protein 3	protein_coding	chr1:31365625-	1026	1207.13515	858.3696411	-0.575	1.67E-08	7.16E-08
TOPBP1	DNA topoisomerase II binding protein 1	protein_coding	chr3:133598175	7304	3.876028873	3.043737654	-0.435	1.67E-08	7.17E-08
AC130371.2	novel transcript	lncRNA	chr17:83098377	611	3.316164842	0.836730847	-2.071	1.68E-08	7.18E-08
ANKRD52	ankyrin repeat domain 52	protein_coding	chr12:56237807	8813	19.4522112	14.10962079	-0.546	1.68E-08	7.20E-08
ZNF738	zinc finger protein 738	protein_coding	chr19:2135893C	3905	3.380233767	2.390779224	-0.583	1.69E-08	7.22E-08
VPS18	VPS18 core subunit of CORVET and HOPS (	protein_coding	chr15:4089445C	4029	11.51575243	9.057185297	-0.43	1.70E-08	7.25E-08
CORO2B	coronin 2B	protein_coding	chr15:68578969	3998	4.418555084	3.177735196	-0.558	1.72E-08	7.34E-08
HEY2	hes related family bHLH transcription fact	protein_coding	chr6:125747664	2689	24.47923484	20.29121788	-0.357	1.74E-08	7.43E-08
PLK2	polo like kinase 2	protein_coding	chr5:58453982-	4647	7.449931697	5.01843172	-0.652	1.74E-08	7.43E-08
CEP350	centrosomal protein 350	protein_coding	chr1:179954773	14261	5.062010848	3.585091744	-0.58	1.75E-08	7.47E-08
PARP4	poly(ADP-ribose) polymerase family meml	protein_coding	chr13:24420931	6043	7.320012354	5.408862807	-0.522	1.78E-08	7.58E-08
POLR2B	RNA polymerase II subunit B	protein_coding	chr4:56977722-	5891	27.74591052	23.88199401	-0.302	1.78E-08	7.58E-08
WDR34	WD repeat domain 34	protein_coding	chr9:128633661	2604	17.12165581	14.14923282	-0.361	1.79E-08	7.63E-08
H1FX	H1 histone family member X	protein_coding	chr3:129314771	1516	31.5423654	25.45980375	-0.396	1.81E-08	7.70E-08
VWC2	von Willebrand factor C domain containii	protein_coding	chr7:49773638-	11322	8.426880389	6.76814454	-0.402	1.81E-08	7.71E-08
RNF20	ring finger protein 20	protein_coding	chr9:101533853	4692	10.64929521	8.557278435	-0.401	1.84E-08	7.81E-08
SEPT7	septin 7	protein_coding	chr7:35800932-	10906	21.38000982	18.42238448	-0.301	1.85E-08	7.83E-08
SERINC1	serine incorporator 1	protein_coding	chr6:122443351	3125	62.24824597	53.42485654	-0.307	1.88E-08	7.97E-08
POLH	DNA polymerase eta	protein_coding	chr6:43576150-	3540	10.02255087	8.177590549	-0.38	1.88E-08	7.97E-08
KCND3	potassium voltage-gated channel subfami	protein_coding	chr1:111770662	7863	1.664815832	1.039879759	-0.759	1.89E-08	7.98E-08
DTL	denticleless E3 ubiquitin protein ligase hc	protein_coding	chr1:212035553	4923	3.380757243	1.907643699	-0.916	1.91E-08	8.05E-08
FANCI	FA complementation group I	protein_coding	chr15:89243949	7766	3.227914182	2.145137874	-0.675	1.91E-08	8.06E-08
TMEM41A	transmembrane protein 41A	protein_coding	chr3:185476496	8668	3.446590509	2.669769784	-0.453	1.93E-08	8.12E-08
GGT7	gamma-glutamyltransferase 7	protein_coding	chr20:3484472C	2962	35.57265269	28.38000347	-0.411	1.94E-08	8.19E-08
ITPKA	inositol-trisphosphate 3-kinase A	protein_coding	chr15:41493393	2093	1.403783642	0.593404405	-1.322	1.95E-08	8.22E-08
RDX	radixin	protein_coding	chr11:10986429	8516	22.48990811	19.39330123	-0.3	1.96E-08	8.23E-08
MICALL1	MICAL like 1	protein_coding	chr22:37905657	5968	11.83606606	9.153284114	-0.454	1.96E-08	8.25E-08
TMEM97	transmembrane protein 97	protein_coding	chr17:2831920C	2929	7.098895399	5.358557164	-0.491	1.97E-08	8.27E-08
YY1AP1	YY1 associated protein 1	protein_coding	chr1:155659443	5001	8.287655775	6.541741201	-0.427	2.02E-08	8.48E-08
TSPAN18	tetraspanin 18	protein_coding	chr11:44726465	5871	9.558651384	6.371352795	-0.669	2.03E-08	8.51E-08
SCAPER	S-phase cyclin A associated protein in the	protein_coding	chr15:76347904	11610	2.440840061	1.907050498	-0.442	2.04E-08	8.53E-08
CCDC126	coiled-coil domain containing 126	protein_coding	chr7:23597382-	3547	2.457084587	1.548046851	-0.749	2.06E-08	8.62E-08
AC073896.2	novel transcript, antisense to CNPY2 and (	lncRNA	chr12:56300076	3826	1.565133274	0.898606414	-0.887	2.08E-08	8.69E-08
SLC35F1	solute carrier family 35 member F1	protein_coding	chr6:117907264	5117	6.491884994	4.607272824	-0.576	2.09E-08	8.73E-08
TNFRSF25	TNF receptor superfamily member 25	protein_coding	chr1:6460786-6	4283	2.167720021	1.18513777	-0.952	2.11E-08	8.78E-08
SAR1B	secretion associated Ras related GTPase 1I	protein_coding	chr5:134601149	10798	7.128482336	5.938849211	-0.35	2.13E-08	8.88E-08
OSBPL11	oxysterol binding protein like 11	protein_coding	chr3:125528858	4598	7.491909059	6.015097617	-0.401	2.14E-08	8.92E-08
ATP2B4	ATPase plasma membrane Ca2+ transporti	protein_coding	chr1:203626561	9721	87.86068974	67.61563559	-0.463	2.15E-08	8.92E-08
SPTBN1	spectrin beta, non-erythrocytic 1	protein_coding	chr2:54456317-	18473	86.00143944	63.96967415	-0.511	2.15E-08	8.95E-08
PKP4	plakophilin 4	protein_coding	chr2:158456964	9525	5.976594795	4.735826118	-0.421	2.15E-08	8.95E-08
RNF5	ring finger protein 5	protein_coding	chr6:32178405-	1127	73.37773014	53.82393117	-0.53	2.17E-08	9.00E-08
DHRS3	dehydrogenase/reductase 3	protein_coding	chr1:12567910-	3515	1.490117774	0.784650951	-1.012	2.17E-08	9.00E-08
GBA2	glucosylceramidase beta 2	protein_coding	chr9:35736866-	4803	70.37245632	60.25528525	-0.31	2.17E-08	9.01E-08
DNMT1	DNA methyltransferase 1	protein_coding	chr19:10133345	9796	10.48374904	8.709388809	-0.354	2.19E-08	9.08E-08
FBXO9	F-box protein 9	protein_coding	chr6:53051991-	8164	16.76283758	14.19785472	-0.326	2.21E-08	9.16E-08
ACADVL	acyl-CoA dehydrogenase very long chain	protein_coding	chr17:7217125-	4820	78.54024476	66.6951063	-0.322	2.24E-08	9.28E-08
SERPINE1	serpin family E member 1	protein_coding	chr7:101127089	3190	2.774207099	1.820647564	-0.69	2.25E-08	9.31E-08
NDFIP2	Nedd4 family interacting protein 2	protein_coding	chr13:79481124	4994	13.5485522	10.79232131	-0.414	2.26E-08	9.34E-08
MAF1	MAF1 homolog, negative regulator of RNA	protein_coding	chr8:144104461	2415	52.13903045	42.55611665	-0.378	2.27E-08	9.36E-08
APAF1	apoptotic peptidase activating factor 1	protein_coding	chr12:98645141	7908	1.358522957	0.767952737	-0.919	2.27E-08	9.39E-08
FGF12	fibroblast growth factor 12	protein_coding	chr3:192139395	7355	48.04236961	40.36974683	-0.337	2.32E-08	9.55E-08
ATXN1	ataxin 1	protein_coding	chr6:16299112-	17812	7.351600157	5.418112049	-0.523	2.35E-08	9.67E-08
AKT2	AKT serine/threonine kinase 2	protein_coding	chr19:40230317	15035	9.210165296	7.532924265	-0.375	2.37E-08	9.73E-08
CAPRIN2	caprin family member 2	protein_coding	chr12:30709552	8392	5.584914796	4.325930977	-0.453	2.37E-08	9.75E-08
PRDM4	PR/SET domain 4	protein_coding	chr12:10773287	4772	4.740444818	3.472402336	-0.535	2.39E-08	9.83E-08
OPLAH	5-oxoprolinase, ATP-hydrolysing	protein_coding	chr8:144051266	4441	12.04431248	9.307740391	-0.458	2.42E-08	9.94E-08
MAN1A2	mannosidase alpha class 1A member 2	protein_coding	chr1:117367449	9667	7.555730362	5.801214356	-0.466	2.47E-08	1.01E-07
RMDN1	regulator of microtubule dynamics 1	protein_coding	chr8:86468257-	5552	16.09722112	13.69126147	-0.32	2.47E-08	1.01E-07
PSD	pleckstrin and Sec7 domain containing	protein_coding	chr10:10240261	5979	1.391298791	0.861821313	-0.774	2.48E-08	1.01E-07
LGI4	leucine rich repeat LGI family member 4	protein_coding	chr19:35124513	4532	1.057268507	0.45267115	-1.306	2.48E-08	1.02E-07
BRIX1	biogenesis of ribosomes BRX1	protein_coding	chr5:34915376-	5011	4.085318363	2.827946791	-0.618	2.49E-08	1.02E-07
LOXL2	lysyl oxidase like 2	protein_coding	chr8:23297189-	5707	5.59271012	4.174155743	-0.504	2.49E-08	1.02E-07
TMEM223	transmembrane protein 223	protein_coding	chr11:62771629	2280	16.44461114	12.85296757	-0.44	2.54E-08	1.04E-07
PACS1	phosphofurin acidic cluster sorting protei	protein_coding	chr11:66070272	9125	27.55293712	22.21810867	-0.396	2.58E-08	1.05E-07
PHF2	PHD finger protein 2	protein_coding	chr9:93576584-	5392	10.61071314	7.643684232	-0.556	2.59E-08	1.05E-07
AC004151.1	Rho GTPase activating protein 45	protein_coding	chr19:1065923-	6238	2.231900236	0.158383259	-3.917	2.59E-08	1.06E-07
RPL22L1	ribosomal protein L22 like 1	protein_coding	chr3:170864875	2051	5.573420765	3.411875471	-0.793	2.67E-08	1.09E-07
CEP68	centrosomal protein 68	protein_coding	chr2:65056366-	8623	6.800924367	5.712240801	-0.338	2.75E-08	1.11E-07
UBE2B	ubiquitin conjugating enzyme E2 B	protein_coding	chr5:134371179	3291	21.58851863	17.3253532	-0.404	2.75E-08	1.11E-07
MARCH3	membrane associated ring-CH-type finger	protein_coding	chr5:126867714	4929	9.322619799	7.11860183	-0.476	2.75E-08	1.11E-07
LINC00205	long intergenic non-protein coding RNA 2	lncRNA	chr21:4528805C	7619	4.387460868	3.142075585	-0.564	2.75E-08	1.11E-07
CLCNKB	chloride voltage-gated channel Kb	protein_coding	chr1:16043736-	2939	2.722209461	1.657685849	-0.802	2.76E-08	1.12E-07
PHF20	PHD finger protein 20	protein_coding	chr20:35771974	8372	6.181118864	5.085735534	-0.367	2.76E-08	1.12E-07
DNPEP	aspartyl aminopeptidase	protein_coding	chr2:219373527	4753	16.81309236	14.18956161	-0.33	2.76E-08	1.12E-07
DIP2B	disco interacting protein 2 homolog B	protein_coding	chr12:50504985	10351	12.31459935	9.527571566	-0.454	2.79E-08	1.13E-07
PALB2	partner and localizer of BRCA2	protein_coding	chr16:2360316C	4747	2.324127973	1.562774037	-0.654	2.80E-08	1.13E-07
NOP9	NOP9 nucleolar protein	protein_coding	chr14:2429985C	6045	6.969619922	5.243882408	-0.493	2.81E-08	1.13E-07
LINC00472	long intergenic non-protein coding RNA 4	lncRNA	chr6:71343427-	19875	1.187325074	0.932237253	-0.437	2.81E-08	1.14E-07
NFATC2IP	nuclear factor of activated T cells 2 intera(	protein_coding	chr16:28950807	5738	12.25189455	9.908402544	-0.391	2.85E-08	1.15E-07
ATP5MC1	ATP synthase membrane subunit c locus 1	protein_coding	chr17:48892765	2615	67.68775079	48.17992037	-0.572	2.88E-08	1.16E-07
GRAMD4	GRAM domain containing 4	protein_coding	chr22:46576012	4901	17.69715243	13.90255402	-0.433	2.88E-08	1.16E-07
SFRP1	secreted frizzled related protein 1	protein_coding	chr8:41261962-	4733	132.1838424	111.7297646	-0.329	2.89E-08	1.16E-07
DDX39B	DExD-box helicase 39B	protein_coding	chr6:31530219-	7007	18.31069771	14.94895856	-0.378	2.90E-08	1.17E-07
S100PBP	S100P binding protein	protein_coding	chr1:32816767-	9190	3.746788517	2.791298139	-0.51	2.94E-08	1.18E-07
CHFR	checkpoint with forkhead and ring finger (	protein_coding	chr12:13282218	17127	1.594141346	1.273905028	-0.408	2.99E-08	1.20E-07
SHROOM2	shroom family member 2	protein_coding	chrX:9786429-9	8218	4.578707569	3.383936271	-0.52	2.99E-08	1.20E-07
C18orf32	chromosome 18 open reading frame 32	protein_coding	chr18:4947725C	5740	1.154305864	0.676690803	-0.857	3.02E-08	1.21E-07
ENOX1	ecto-NOX disulfide-thiol exchanger 1	protein_coding	chr13:43213518	3101	3.270566628	2.114609405	-0.718	3.02E-08	1.21E-07
NCOA4	nuclear receptor coactivator 4	protein_coding	chr10:46005088	4079	75.3063066	62.64402195	-0.351	3.03E-08	1.21E-07
SLC25A44	solute carrier family 25 member 44	protein_coding	chr1:156193932	7293	4.375846338	3.459103846	-0.423	3.03E-08	1.21E-07
DLGAP5	DLG associated protein 5	protein_coding	chr14:55148112	3597	5.710725822	3.909854963	-0.634	3.07E-08	1.23E-07
CD2AP	CD2 associated protein	protein_coding	chr6:47477789-	6751	5.218314415	3.976601834	-0.48	3.08E-08	1.23E-07
PTBP3	polypyrimidine tract binding protein 3	protein_coding	chr9:112217716	8239	2.373920563	1.767402677	-0.51	3.10E-08	1.24E-07
SNX29	sorting nexin 29	protein_coding	chr16:11976734	11299	1.374438596	0.744154868	-0.96	3.11E-08	1.24E-07
MTOR	mechanistic target of rapamycin kinase	protein_coding	chr1:11106535-	12119	11.37761779	8.583759837	-0.49	3.12E-08	1.25E-07
AP1B1	adaptor related protein complex 1 subuni	protein_coding	chr22:2932768C	6721	18.44571106	15.95591425	-0.295	3.14E-08	1.25E-07
C1QTNF1-AS1	C1QTNF1 antisense RNA 1	lncRNA	chr17:79018131	2062	1.416235833	0.518080834	-1.51	3.16E-08	1.26E-07
RGMB	repulsive guidance molecule BMP co-rece	protein_coding	chr5:98768650-	6671	5.557003941	4.636350701	-0.348	3.18E-08	1.27E-07
IL17RC	interleukin 17 receptor C	protein_coding	chr3:9917074-9	4540	9.54602192	7.817204245	-0.373	3.20E-08	1.27E-07
STX11	syntaxin 11	protein_coding	chr6:144150526	1926	5.350138553	3.671746093	-0.627	3.21E-08	1.28E-07
CDC42SE2	CDC42 small effector 2	protein_coding	chr5:131245493	6024	3.424073475	2.524529765	-0.528	3.22E-08	1.28E-07
PABPC1	poly(A) binding protein cytoplasmic 1	protein_coding	chr8:100685816	4147	96.7681136	81.71606648	-0.33	3.22E-08	1.28E-07
RRM2B	ribonucleotide reductase regulatory TP53	protein_coding	chr8:102204502	5548	9.76185984	8.02920957	-0.37	3.23E-08	1.28E-07
USP32	ubiquitin specific peptidase 32	protein_coding	chr17:60177327	9522	8.956669666	7.39018884	-0.363	3.27E-08	1.30E-07
CDK16	cyclin dependent kinase 16	protein_coding	chrX:47217860-	6531	56.58950243	49.04082577	-0.293	3.28E-08	1.30E-07
HEATR3	HEAT repeat containing 3	protein_coding	chr16:5006597C	5039	6.135249923	4.844866623	-0.425	3.32E-08	1.32E-07
RAP1GAP	RAP1 GTPase activating protein	protein_coding	chr1:21596215-	5755	1.051348102	0.614174858	-0.858	3.33E-08	1.32E-07

ERBB4	erb-b2 receptor tyrosine kinase 4	protein_coding	chr2:21137571‍7	13015	8.844527776	7.085851192	-0.405	3.33E-08	1.32E-07
AL031123.1	novel transcript	lncRNA	chr6:6692741-6	3056	0.901660559	0.133724694	-2.791	3.35E-08	1.33E-07
MMP23B	matrix metallopeptidase 23B	protein_coding	chr1:1632095-1	2273	4.169601825	2.904829885	-0.608	3.35E-08	1.33E-07
SMAD7	SMAD family member 7	protein_coding	chr18:48919853	5033	7.541163819	5.623389478	-0.507	3.36E-08	1.33E-07
AC112220.2	novel transcript	lncRNA	chr3:33793644-	4524	1.735558755	1.016346	-0.854	3.36E-08	1.33E-07
BMPR1A	bone morphogenetic protein receptor typ	protein_coding	chr10:86756601	11412	8.556286635	7.40486565	-0.295	3.36E-08	1.33E-07
SLC44A1	solute carrier family 44 member 1	protein_coding	chr9:105244622	12172	1.170426016	0.841123302	-0.568	3.37E-08	1.33E-07
MAPRE3	microtubule associated protein RP/EB fan	protein_coding	chr2:26970612-	3630	22.07739924	18.4855934	-0.342	3.39E-08	1.34E-07
ATP6AP1	ATPase H+ transporting accessory protein	protein_coding	chrX:154428645	4871	27.84294715	23.86061033	-0.309	3.44E-08	1.36E-07
AC005670.3	novel transcript	lncRNA	chr17:46983287	16607	2.553986665	1.730106644	-0.643	3.46E-08	1.37E-07
RAB11FIP5	RAB11 family interacting protein 5	protein_coding	chr2:73073382-	7058	8.793157271	6.409152329	-0.539	3.48E-08	1.37E-07
UPP1	uridine phosphorylase 1	protein_coding	chr7:48088628-	4580	18.55898036	15.13231838	-0.379	3.49E-08	1.38E-07
TRIQK	triple QxxK/R motif containing	protein_coding	chr8:92883530-	6724	1.461474015	0.960291316	-0.697	3.53E-08	1.39E-07
BAHD1	bromo adjacent homology domain contai	protein_coding	chr15:40439721	5449	9.3582327	7.237252724	-0.456	3.54E-08	1.39E-07
ENTPD7	ectonucleoside triphosphate diphosphoh	protein_coding	chr10:99659509	8655	1.027174416	0.692839845	-0.653	3.54E-08	1.39E-07
HJURP	Holliday junction recognition protein	protein_coding	chr2:23383341‍6	3821	3.246748465	2.021303432	-0.773	3.55E-08	1.39E-07
MEIS3	Meis homeobox 3	protein_coding	chr19:47403124	5122	2.799017312	2.032265702	-0.553	3.59E-08	1.41E-07
AKAP8L	A-kinase anchoring protein 8 like	protein_coding	chr19:15380050	5079	20.58205593	17.61588685	-0.311	3.61E-08	1.41E-07
PACSIN1	protein kinase C and casein kinase substra	protein_coding	chr6:34466061-	4805	1.819048116	1.068244027	-0.842	3.61E-08	1.41E-07
PKMYT1	protein kinase, membrane associated tyro	protein_coding	chr16:2968024-	4974	2.614259948	1.689739773	-0.711	3.61E-08	1.41E-07
CDON	cell adhesion associated, oncogene regula	protein_coding	chr11:12595579 10600		2.32105367	1.609279162	-0.612	3.62E-08	1.42E-07
ZDHHC8	zinc finger DHHC-type containing 8	protein_coding	chr22:20129456	5542	18.30077769	14.35193664	-0.436	3.63E-08	1.42E-07
GPT	glutamic--pyruvic transaminase	protein_coding	chr8:144502973	2756	3.165936785	1.822399693	-0.88	3.65E-08	1.43E-07
LDLRAD4	low density lipoprotein receptor class A d	protein_coding	chr18:13217498	16759	3.931437563	2.847855203	-0.549	3.66E-08	1.43E-07
ZZEF1	zinc finger ZZ-type and EF-hand domain cc	protein_coding	chr17:4004445-	15888	4.669693069	3.439194847	-0.523	3.69E-08	1.44E-07
PSRC1	proline and serine rich coiled-coil 1	protein_coding	chr1:109279556	2855	1.854968305	0.907233648	-1.119	3.71E-08	1.45E-07
POLQ	DNA polymerase theta	protein_coding	chr3:121431427	9382	1.348687191	0.893839868	-0.679	3.71E-08	1.45E-07
AC006449.6	novel transcript	lncRNA	chr17:38702452	2296	2.737743202	1.525290972	-0.922	3.76E-08	1.47E-07
SH3TC2	SH3 domain and tetratricopeptide repeat	protein_coding	chr5:148923639	15165	1.740898242	1.100885275	-0.741	3.80E-08	1.48E-07
TSPEAR	thrombospondin type laminin G domain	protein_coding	chr21:44497893	4462	3.638033877	2.304882568	-0.741	3.80E-08	1.48E-07
FBXW8	F-box and WD repeat domain containing	protein_coding	chr12:11691095	5209	4.967311643	3.778768385	-0.479	3.81E-08	1.48E-07
AGT	angiotensinogen	protein_coding	chr1:230702523	2116	34.39600459	27.8982398	-0.386	3.82E-08	1.49E-07
MB21D2	Mab-21 domain containing 2	protein_coding	chr3:192796815	3063	7.142547247	5.121329445	-0.564	3.82E-08	1.49E-07
RIC3	RIC3 acetylcholine receptor chaperone	protein_coding	chr11:8106056-	7694	3.815713733	2.879400517	-0.492	3.84E-08	1.49E-07
POLR2G	RNA polymerase II subunit G	protein_coding	chr11:62761565	1902	15.03196319	10.20990911	-0.648	3.90E-08	1.51E-07
CNTN1	contactin 1	protein_coding	chr12:40692439	7492	8.715333567	7.103865222	-0.383	3.96E-08	1.53E-07
TMEM67	transmembrane protein 67	protein_coding	chr8:93754844-	8281	3.73165298	2.841169122	-0.479	3.96E-08	1.53E-07
SYTL5	synaptotagmin like 5	protein_coding	chrX:38006553-	4820	10.17791166	8.028820827	-0.429	3.96E-08	1.53E-07
ODR4	odr-4 GPCR localization factor homolog	protein_coding	chr1:186375838	4073	4.393350827	3.39472884	-0.457	4.02E-08	1.56E-07
PERP	p53 apoptosis effector related to PMP22	protein_coding	chr6:138088505	4198	40.72187956	34.84831244	-0.312	4.02E-08	1.56E-07
PPP3R1	protein phosphatase 3 regulatory subunit	protein_coding	chr2:68178857-	3364	14.11116445	11.41187463	-0.392	4.03E-08	1.56E-07
ANAPC13	anaphase promoting complex subunit 13	protein_coding	chr3:134477706	2359	18.88836849	13.30895015	-0.587	4.06E-08	1.57E-07
LRATD2	LRAT domain containing 2	protein_coding	chr8:126552443	5734	22.46797304	17.2040845	-0.469	4.07E-08	1.57E-07
PPAT	phosphoribosyl pyrophosphate amidotra	protein_coding	chr4:56393362-	5439	3.932753887	2.966843496	-0.493	4.10E-08	1.58E-07
TONSL	tonsoku like, DNA repair protein	protein_coding	chr8:144428775	7107	2.991828894	2.275440768	-0.478	4.11E-08	1.58E-07
PINK1	PTEN induced kinase 1	protein_coding	chr1:20633458-	4833	30.08582405	26.18301262	-0.287	4.14E-08	1.59E-07
NXN	nucleoredoxin	protein_coding	chr17:799310-9	5889	13.27091009	11.41156686	-0.304	4.17E-08	1.60E-07
FANCA	FA complementation group A	protein_coding	chr16:89737549	9736	1.274288841	0.863559455	-0.644	4.19E-08	1.61E-07
PAQR4	progestin and adipoQ receptor family mer	protein_coding	chr16:2969245-	2972	4.324296805	2.888201982	-0.664	4.21E-08	1.61E-07
CENPU	centromere protein U	protein_coding	chr4:184694085	3483	4.521149104	3.241977925	-0.566	4.26E-08	1.63E-07
RAB27A	RAB27A, member RAS oncogene family	protein_coding	chr15:55202966	5603	1.55784849	0.916833812	-0.842	4.27E-08	1.63E-07
EEFSEC	eukaryotic elongation factor, selenocyste	protein_coding	chr3:128153481	2394	7.857614292	5.853797427	-0.511	4.27E-08	1.64E-07
EMSY	EMSY transcriptional repressor, BRCA2 int	protein_coding	chr11:76444923	11783	2.79237316	1.944898574	-0.605	4.28E-08	1.64E-07
TSPYL2	TSPY like 2	protein_coding	chrX:53082367-	4948	4.35851491	3.351371999	-0.465	4.28E-08	1.64E-07
CWF19L1	CWF19 like cell cycle control factor 1	protein_coding	chr10:10023229	3985	5.648712728	4.312887614	-0.476	4.29E-08	1.64E-07
RELL1	RELT like 1	protein_coding	chr4:37590800-	4214	2.218459171	1.450998534	-0.699	4.36E-08	1.67E-07
RAD54L	RAD54 like	protein_coding	chr1:46246461-	4078	1.633679033	0.96562489	-0.836	4.38E-08	1.67E-07
MAPK9	mitogen-activated protein kinase 9	protein_coding	chr5:180233143	8955	8.066458677	6.568459088	-0.381	4.39E-08	1.68E-07
SLC25A13	solute carrier family 25 member 13	protein_coding	chr7:96120220-	3710	10.27405662	7.946365162	-0.454	4.41E-08	1.68E-07
TANC1	tetratricopeptide repeat, ankyrin repeat a	protein_coding	chr2:159868640	14915	25.94469312	21.30900311	-0.369	4.42E-08	1.68E-07
GPRC5C	G protein-coupled receptor class C group	protein_coding	chr17:74424851	8005	1.553609563	0.977646979	-0.744	4.46E-08	1.70E-07
PRDM16	PR/SET domain 16	protein_coding	chr1:3069168-3	9767	4.329381512	3.147766176	-0.543	4.53E-08	1.72E-07
CDC42EP3	CDC42 effector protein 3	protein_coding	chr2:37641882-	10406	23.59788757	20.01059427	-0.324	4.54E-08	1.73E-07
UBE2S	ubiquitin conjugating enzyme E2 S	protein_coding	chr19:55399745	2799	29.23929963	23.44333083	-0.407	4.54E-08	1.73E-07
NUP62CL	nucleoporin 62 C-terminal like	protein_coding	chrX:107123427	1867	1.961042773	1.110292547	-0.906	4.55E-08	1.73E-07
AL953883.1	novel transcript	lncRNA	chr9:38949734-	1015	2.49622583	0.969307167	-1.443	4.56E-08	1.73E-07
LHFPL2	LHFPL tetraspan subfamily member 2	protein_coding	chr5:78485215-	7977	4.013134326	2.961616125	-0.522	4.62E-08	1.75E-07
BTRC	beta-transducin repeat containing E3 ubi	protein_coding	chr10:10135403	6613	11.3778515	9.359715861	-0.367	4.62E-08	1.75E-07
EGF	epidermal growth factor	protein_coding	chr4:109912884	7616	3.150205443	2.214200619	-0.6	4.63E-08	1.75E-07
PNRC1	proline rich nuclear receptor coactivator	protein_coding	chr6:89080751-	2992	40.86932657	34.69515451	-0.322	4.69E-08	1.78E-07
CDC20	cell division cycle 20	protein_coding	chr1:43358981-	2012	8.866157156	6.127025617	-0.613	4.71E-08	1.78E-07
EVL	Enah/Vasp-like	protein_coding	chr14:99971449	10482	13.83620637	12.09129952	-0.281	4.72E-08	1.78E-07
SCLT1	sodium channel and clathrin linker 1	protein_coding	chr4:128864921	8980	1.495448813	1.016971328	-0.648	4.75E-08	1.80E-07
KIF9-AS1	KIF9 antisense RNA 1	lncRNA	chr3:47164186-	6987	1.251137987	0.842247932	-0.654	4.77E-08	1.80E-07
CCDC50	coiled-coil domain containing 50	protein_coding	chr3:191329085	9183	11.9999207	10.23807747	-0.316	4.78E-08	1.80E-07
Z99572.1	novel transcript	lncRNA	chr1:169486076	14107	0.989833929	0.709952166	-0.564	4.81E-08	1.81E-07
MICA	MHC class I polypeptide-related sequence	protein_coding	chr6:31399784-	2370	6.549127281	4.747973882	-0.55	4.84E-08	1.82E-07
ITPRIP	inositol 1,4,5-trisphosphate receptor inte	protein_coding	chr10:10430969	7296	5.620101957	4.05913653	-0.552	4.90E-08	1.85E-07
BX255925.3	novel RING finger protein	protein_coding	chr9:137217452	1910	6.935993305	4.581277301	-0.68	4.95E-08	1.86E-07
MPHOSPH9	M-phase phosphoprotein 9	protein_coding	chr12:12315232	10304	5.252353853	4.441655823	-0.328	4.95E-08	1.86E-07
GAK	cyclin G associated kinase	protein_coding	chr4:849276-93	15155	6.03758693	5.015605133	-0.353	4.97E-08	1.87E-07
RBFOX1	RNA binding fox-1 homolog 1	protein_coding	chr16:5239802-	10121	1.215773458	0.826422819	-0.644	5.00E-08	1.88E-07
DHDDS	dehydrodolichyl diphosphate synthase su	protein_coding	chr1:26432282-	5608	5.825082757	4.840645354	-0.353	5.02E-08	1.88E-07
SYNJ1	synaptojanin 1	protein_coding	chr21:32628759 8070		3.623098441	2.768627045	-0.472	5.08E-08	1.90E-07
AKT1	AKT serine/threonine kinase 1	protein_coding	chr14:10476934	11162	18.50116254	16.28117897	-0.271	5.10E-08	1.91E-07
MAN2A2	mannosidase alpha class 2A member 2	protein_coding	chr15:90902218	9763	11.2017398	8.610110362	-0.464	5.11E-08	1.91E-07
SH3PXD2B	SH3 and PX domains 2B	protein_coding	chr5:172325000	8244	1.148460776	0.738880176	-0.716	5.14E-08	1.92E-07
B3GALNT2	beta-1,3-N-acetylgalactosaminyltransfera	protein_coding	chr1:235447190	8406	5.908806844	4.803164537	-0.386	5.19E-08	1.94E-07
RPN2	ribophorin II	protein_coding	chr20:37178410	2700	53.71805031	45.68127565	-0.32	5.19E-08	1.94E-07
TKT	transketolase	protein_coding	chr3:53224712-	9502	6.411405835	5.212471522	-0.385	5.21E-08	1.94E-07
SCNN1D	sodium channel epithelial 1 delta subunit	protein_coding	chr1:1280436-1	4025	2.170794265	1.421723502	-0.695	5.28E-08	1.97E-07
ESPL1	extra spindle pole bodies like 1, separase	protein_coding	chr12:53268299	8890	1.193743857	0.619304044	-1.018	5.29E-08	1.97E-07
ASCC2	activating signal cointegrator 1 complex s	protein_coding	chr22:29788609	5415	10.61312861	8.547159368	-0.399	5.30E-08	1.97E-07
MARC2	mitochondrial amidoxime reducing comp	protein_coding	chr1:220748225	2597	24.39391537	20.58942473	-0.331	5.33E-08	1.98E-07
FDPS	farnesyl diphosphate synthase	protein_coding	chr1:155308748	3718	18.6380252	14.61066293	-0.435	5.36E-08	1.99E-07
KIFC1	kinesin family member C1	protein_coding	chr6:33391823- 3030		6.057607156	4.258277845	-0.592	5.40E-08	2.01E-07
ETV5	ETS variant 5	protein_coding	chr3:186046314	5773	1.748397071	0.950223798	-0.972	5.41E-08	2.01E-07
FBXL7	F-box and leucine rich repeat protein 7	protein_coding	chr5:15500180-	4870	22.57280961	17.94194297	-0.416	5.46E-08	2.03E-07
SHBG	sex hormone binding globulin	protein_coding	chr17:7613946-	1971	1.069280795	0.37105078	-1.62	5.49E-08	2.03E-07
DBN1	drebrin 1	protein_coding	chr5:177456608	5119	52.06295493	44.41588852	-0.316	5.50E-08	2.04E-07
MCOLN1	mucolipin 1	protein_coding	chr19:7522624-	3958	3.218470987	2.39104607	-0.515	5.55E-08	2.05E-07
GALNT16	polypeptide N-acetylgalactosaminyltrans	protein_coding	chr14:69259277	11462	2.490558922	1.726288675	-0.614	5.56E-08	2.06E-07
IDH3A	isocitrate dehydrogenase 3 (NAD(+)) alpha	protein_coding	chr15:78131498	6823	66.63347749	54.47777131	-0.376	5.59E-08	2.07E-07
MRFAP1	Morf4 family associated protein 1	protein_coding	chr4:6640091-6	2655	142.8287195	118.0320596	-0.361	5.60E-08	2.07E-07
GJD2	gap junction protein delta 2	protein_coding	chr15:34751032	2922	7.10774278	4.729047823	-0.674	5.61E-08	2.07E-07
CLK3	CDC like kinase 3	protein_coding	chr15:74598500	20443	3.315823863	2.761792959	-0.351	5.63E-08	2.08E-07
GPR146	G protein-coupled receptor 146	protein_coding	chr7:1044546-1	3190	1.292449227	0.651454829	-1.074	5.64E-08	2.08E-07
NAA40	N(alpha)-acetyltransferase 40, NatD catal	protein_coding	chr11:63938959	5312	16.17875216	13.78737673	-0.316	5.66E-08	2.09E-07
AC026748.3	novel transcript	lncRNA	chr5:1345197-1	3302	3.790474988	2.566275205	-0.646	5.70E-08	2.10E-07

DNAJB6	DnaJ heat shock protein family (Hsp40) m	protein_coding	chr7:157335381	14981	8.638852874	7.375644007	-0.314	5.73E-08	2.11E-07
AKT1S1	AKT1 substrate 1	protein_coding	chr19:49869033	5737	15.24640592	12.78269135	-0.341	5.78E-08	2.13E-07
PLCB4	phospholipase C beta 4	protein_coding	chr20:9068763-	6900	7.845385778	6.359236385	-0.388	5.80E-08	2.13E-07
TMEM230	transmembrane protein 230	protein_coding	chr20:5068232-	3051	29.48579125	22.41336451	-0.479	5.82E-08	2.14E-07
LRRC52-AS1	LRRC52 antisense RNA 1	lncRNA	chr1:16547683	4348	1.102771804	0.603176163	-0.947	5.88E-08	2.16E-07
AC027237.1	kinesin family member 23	protein_coding	chr15:69414246	6576	5.413764063	3.734741696	-0.624	5.89E-08	2.16E-07
C1orf159	chromosome 1 open reading frame 159	protein_coding	chr1:1081818-1	6322	2.057963137	1.497460221	-0.542	5.90E-08	2.17E-07
ENPP5	ectonucleotide pyrophosphatase/phosph	protein_coding	chr6:46159185-	4275	1.689561311	1.082653947	-0.723	5.94E-08	2.18E-07
HEXD	hexosaminidase D	protein_coding	chr17:82418318	4541	6.159265538	4.716455072	-0.473	5.97E-08	2.19E-07
HK1	hexokinase 1	protein_coding	chr10:69269984	6296	82.70541158	70.79721895	-0.311	5.97E-08	2.19E-07
SIPA1L2	signal induced proliferation associated 1 l	protein_coding	chr1:23239796	7641	93.88432457	75.04323781	-0.407	5.99E-08	2.20E-07
HERPUD2	HERPUD family member 2	protein_coding	chr7:35632659-	3246	7.531835153	6.045305343	-0.403	6.02E-08	2.21E-07
CYB561	cytochrome b561	protein_coding	chr17:63432304	5296	3.467649973	2.530075585	-0.541	6.14E-08	2.25E-07
SMIM3	small integral membrane protein 3	protein_coding	chr5:15077794	2449	13.61577374	10.96918132	-0.396	6.15E-08	2.25E-07
CEP85	centrosomal protein 85	protein_coding	chr1:26234200-	5145	8.680794043	7.106442514	-0.375	6.18E-08	2.26E-07
AQR	aquarius intron-binding spliceosomal fact	protein_coding	chr15:34851782	13431	3.137166106	2.635382965	-0.338	6.22E-08	2.27E-07
BHLHE40	basic helix-loop-helix family member e40	protein_coding	chr3:4979116-4	3837	8.63220477	2.56388032	-1.871	6.23E-08	2.28E-07
TMCC1	transmembrane and coiled-coil domain fa	protein_coding	chr3:129647792	8138	4.735326361	3.503163919	-0.52	6.29E-08	2.30E-07
SUN2	Sad1 and UNC84 domain containing 2	protein_coding	chr22:38734725	6489	6.224578184	4.782837305	-0.465	6.29E-08	2.30E-07
FUZ	fuzzy planar cell polarity protein	protein_coding	chr19:49806869	2950	6.258688142	4.637335762	-0.517	6.29E-08	2.30E-07
NUF2	NUF2 component of NDC80 kinetochore c	protein_coding	chr1:16326657	3362	2.170545444	1.271920476	-0.856	6.30E-08	2.30E-07
ZNF121	zinc finger protein 121	protein_coding	chr19:9560353-	7251	4.84890444	4.035891753	-0.35	6.39E-08	2.33E-07
FBXO22	F-box protein 22	protein_coding	chr15:75903876	11821	5.257853749	4.440616518	-0.331	6.48E-08	2.36E-07
THG1L	tRNA-histidine guanylyltransferase 1 like	protein_coding	chr5:15773142	2998	4.469008418	3.180275389	-0.576	6.49E-08	2.36E-07
OPRL1	opioid related nociceptin receptor 1	protein_coding	chr20:64080082	3640	1.371773107	0.619361316	-1.227	6.52E-08	2.37E-07
FZD3	frizzled class receptor 3	protein_coding	chr8:28494205-	13786	2.221059221	1.777831828	-0.408	6.59E-08	2.40E-07
CAMK1D	calcium/calmodulin dependent protein k	protein_coding	chr10:12349482	8762	1.522227174	1.112940048	-0.539	6.68E-08	2.43E-07
SEC23A	Sec23 homolog A, coat complex II compo	protein_coding	chr14:39031919	8660	24.23717978	21.20746684	-0.279	6.69E-08	2.43E-07
VPS9D1	VPS9 domain containing 1	protein_coding	chr16:89707134	3340	18.63506812	15.65694466	-0.336	6.78E-08	2.46E-07
PNKD	PNKD metallo-beta-lactamase domain cor	protein_coding	chr2:218270392	5184	19.17411239	16.42584328	-0.309	6.83E-08	2.47E-07
TGFBR1	transforming growth factor beta receptor	protein_coding	chr9:99104038-	6822	3.395154252	2.65164274	-0.442	6.88E-08	2.49E-07
FRMD4B	FERM domain containing 4B	protein_coding	chr3:69168782-	11201	1.993211927	1.448371698	-0.548	6.89E-08	2.49E-07
PIGK	phosphatidylinositol glycan anchor biosy	protein_coding	chr1:77088989-	5514	4.102144969	3.177380323	-0.452	7.05E-08	2.55E-07
SYT7	synaptotagmin 7	protein_coding	chr11:61515313	5953	23.79700803	20.13210932	-0.327	7.13E-08	2.57E-07
THOC7	THO complex 7	protein_coding	chr3:63833870-	1749	22.02363061	16.31774746	-0.515	7.19E-08	2.59E-07
ETFA	electron transfer flavoprotein subunit alp	protein_coding	chr15:76215353	5033	42.06519519	34.82365856	-0.359	7.21E-08	2.60E-07
USP18	ubiquitin specific peptidase 18	protein_coding	chr22:18149899	2129	1.21243856	0.485689229	-1.42	7.22E-08	2.60E-07
PPP1R26	protein phosphatase 1 regulatory subunit	protein_coding	chr9:135479079	5307	8.463556329	7.120694234	-0.335	7.32E-08	2.64E-07
CHST6	carbohydrate sulfotransferase 6	protein_coding	chr16:75472052	8620	1.054449647	0.681422511	-0.719	7.43E-08	2.67E-07
ARHGAP10	Rho GTPase activating protein 10	protein_coding	chr4:14773206	10759	4.783306835	3.934557708	-0.368	7.44E-08	2.68E-07
AC022239.2	novel transcript	lncRNA	chr8:11643404-	725	3.242196865	1.226693437	-1.463	7.48E-08	2.69E-07
SLC36A4	solute carrier family 36 member 4	protein_coding	chr11:93144174	9709	1.903071925	1.401218755	-0.526	7.54E-08	2.71E-07
EIF4G3	eukaryotic translation initiation factor 4 g	protein_coding	chr1:20806292-	9596	15.75980472	12.88594816	-0.376	7.61E-08	2.74E-07
DNAJC15	DnaJ heat shock protein family (Hsp40) m	protein_coding	chr13:43023203	8031	3.831719518	3.015520734	-0.431	7.64E-08	2.74E-07
SGTA	small glutamine rich tetratricopeptide rep	protein_coding	chr19:2754715-	3711	28.59575076	24.91246955	-0.285	7.66E-08	2.75E-07
C15orf39	chromosome 15 open reading frame 39	protein_coding	chr15:75195643	5807	10.15787546	8.219689475	-0.391	7.70E-08	2.76E-07
GRN	granulin precursor	protein_coding	chr17:44345246	3524	73.19371472	63.97010597	-0.281	7.71E-08	2.77E-07
SPTB	spectrin beta, erythrocytic	protein_coding	chr14:64746283	14796	18.50326234	13.91260945	-0.494	7.73E-08	2.77E-07
CMTM6	CKLF like MARVEL transmembrane domain	protein_coding	chr3:32481312-	3857	8.241974255	6.438827477	-0.442	7.99E-08	2.86E-07
MAGEF1	MAGE family member F1	protein_coding	chr3:184710367	1636	66.23023704	57.76909319	-0.283	8.08E-08	2.89E-07
COL27A1	collagen type XXVII alpha 1 chain	protein_coding	chr9:114155537	14011	2.731513056	1.741726022	-0.727	8.09E-08	2.89E-07
XRCC1	X-ray repair cross complementing 1	protein_coding	chr19:43543040	3459	5.465611097	4.22648137	-0.456	8.17E-08	2.92E-07
NUP107	nucleoporin 107	protein_coding	chr12:68686951	8103	3.387678209	2.680117106	-0.423	8.27E-08	2.95E-07
NUP35	nucleoporin 35	protein_coding	chr2:18311751	4025	2.103208304	1.210054825	-0.879	8.29E-08	2.96E-07
SULF2	sulfatase 2	protein_coding	chr20:47656348	6657	1.784590773	1.202816244	-0.656	8.31E-08	2.96E-07
SNN	stannin	protein_coding	chr16:11668455	3264	9.288545934	7.36813447	-0.421	8.37E-08	2.98E-07
TMEM237	transmembrane protein 237	protein_coding	chr2:201620184	7647	3.402715329	2.653681707	-0.449	8.44E-08	3.00E-07
PIP5K1B	phosphatidylinositol-4-phosphate 5-kinas	protein_coding	chr9:68705240-	6082	4.682025068	3.806508369	-0.383	8.47E-08	3.01E-07
SGO1	shugoshin 1	protein_coding	chr3:20160593-	3742	1.137930893	0.665189989	-0.86	8.48E-08	3.02E-07
PPP1R3C	protein phosphatase 1 regulatory subunit	protein_coding	chr10:91628442	2541	32.87885529	26.52561435	-0.393	8.49E-08	3.02E-07
POLR2E	RNA polymerase II subunit E	protein_coding	chr19:1086574-	4870	37.0277375	31.65713253	-0.312	8.52E-08	3.03E-07
ADAL	adenosine deaminase like	protein_coding	chr15:43330657	5735	8.438007732	6.865635737	-0.386	8.63E-08	3.07E-07
INHA	inhibin subunit alpha	protein_coding	chr2:219569162	1636	6.429579348	4.580691452	-0.574	8.67E-08	3.07E-07
UBE2K	ubiquitin conjugating enzyme E2 K	protein_coding	chr4:39698109-	5180	21.67618041	19.00205705	-0.276	8.70E-08	3.09E-07
TSPOAP1	TSPO associated protein 1	protein_coding	chr17:58301228	11389	2.641012885	1.680376551	-0.736	8.70E-08	3.09E-07
NPTXR	neuronal pentraxin receptor	protein_coding	chr22:38818452	5830	20.95796352	17.23657685	-0.367	8.71E-08	3.09E-07
ADAMTSL2	ADAMTS like 2	protein_coding	chr9:133532164	4677	1.324784949	0.763589751	-0.871	8.73E-08	3.09E-07
PPP1R14B	protein phosphatase 1 regulatory inhibito	protein_coding	chr11:64244479	1658	35.16604705	21.97680801	-0.758	8.74E-08	3.10E-07
NDUFA2	NADH:ubiquinone oxidoreductase subuni	protein_coding	chr5:14063874	1488	51.36210777	38.898283	-0.485	8.95E-08	3.17E-07
MAP11	microtubule associated protein 11	protein_coding	chr7:10015442	3263	5.77138865	4.525514754	-0.437	8.96E-08	3.17E-07
MEX3A	mex-3 RNA binding family member A	protein_coding	chr1:15607201	6591	5.260725763	4.23742381	-0.399	9.05E-08	3.20E-07
ALG9	ALG9 alpha-1,2-mannosyltransferase	protein_coding	chr11:11117821 8070		0.986442439	0.615711149	-0.761	9.09E-08	3.21E-07
ZFAND6	zinc finger AN1-type containing 6	protein_coding	chr15:80059568	5153	16.42107865	13.70096693	-0.347	9.13E-08	3.22E-07
USP4	ubiquitin specific peptidase 4	protein_coding	chr3:49277831-	6659	12.49917083	10.85256525	-0.29	9.19E-08	3.24E-07
PIAS2	protein inhibitor of activated STAT 2	protein_coding	chr18:46803218	17535	3.499179345	2.999030378	-0.308	9.44E-08	3.33E-07
PDLIM1	PDZ and LIM domain 1	protein_coding	chr10:95237572	2227	213.3937224	180.802561	-0.324	9.45E-08	3.33E-07
THUMPD2	THUMP domain containing 2	protein_coding	chr2:39736060-	3823	2.754529633	1.970599033	-0.564	9.49E-08	3.34E-07
MBNL2	muscleblind like splicing regulator 2	protein_coding	chr13:97221434	4908	7.828980033	6.16884608	-0.432	9.52E-08	3.35E-07
HUNK	hormonally up-regulated Neu-associated l	protein_coding	chr21:31873020	8589	1.994051738	1.304932902	-0.694	9.57E-08	3.36E-07
CHAMP1	chromosome alignment maintaining phos	protein_coding	chr13:11431448	5615	6.322916835	5.204117165	-0.368	9.59E-08	3.37E-07
AC007920.2	novel transcript, antisense to MASP1	lncRNA	chr3:187291272	1973	1.212364382	0.522322416	-1.303	9.64E-08	3.38E-07
XRN1	5'-3' exoribonuclease 1	protein_coding	chr3:142306607	11982	3.755428204	2.967238361	-0.425	9.65E-08	3.39E-07
BRICD5	BRICHOS domain containing 5	protein_coding	chr16:2209253-	2083	3.075455128	1.751422731	-0.896	9.82E-08	3.44E-07
NIPAL2	NIPA like domain containing 2	protein_coding	chr8:98189826-	5606	4.567980488	3.673701508	-0.399	9.86E-08	3.45E-07
RHOF	ras homolog family member F, filopodia a	protein_coding	chr12:12177775	6181	5.432500835	4.031442377	-0.513	9.87E-08	3.46E-07
DNAJC1	DnaJ heat shock protein family (Hsp40) m	protein_coding	chr10:21756548	2490	5.636597361	4.211250666	-0.507	9.88E-08	3.46E-07
NARF	nuclear prelamin A recognition factor	protein_coding	chr17:82458180	7919	11.95009036	9.782432441	-0.374	9.99E-08	3.49E-07
ASIC3	acid sensing ion channel subunit 3	protein_coding	chr7:151048292	2748	4.517225053	2.762814287	-0.794	1.00E-07	3.51E-07
ULBP3	UL16 binding protein 3	protein_coding	chr6:15006105	3111	1.582722174	0.969871309	-0.788	1.01E-07	3.52E-07
ATP2B1	ATPase plasma membrane Ca2+ transporti	protein_coding	chr12:89588049	9493	2.529429583	2.05115518	-0.387	1.02E-07	3.55E-07
KRTAP5-AS1	KRTAP5-1/KRTAP5-2 antisense RNA 1	lncRNA	chr11:1571353-	2931	1.208091949	0.513210879	-1.304	1.03E-07	3.58E-07
FAM111B	family with sequence similarity 111 meml	protein_coding	chr11:59107185	3865	3.999247438	2.679241521	-0.661	1.04E-07	3.62E-07
SLC39A10	solute carrier family 39 member 10	protein_coding	chr2:195575977	6917	5.1962132	4.119091174	-0.421	1.04E-07	3.63E-07
REEP6	receptor accessory protein 6	protein_coding	chr19:1491166- 2030		11.32018316	7.337836976	-0.702	1.05E-07	3.66E-07
RNF167	ring finger protein 167	protein_coding	chr17:4940008-	2744	28.31037244	24.25284152	-0.309	1.05E-07	3.66E-07
DPY19L4	dpy-19 like 4	protein_coding	chr8:94719703-	7136	11.71290695	9.909851335	-0.328	1.05E-07	3.66E-07
NDC1	NDC1 transmembrane nucleoporin	protein_coding	chr1:53765478-	4720	5.181177861	4.028257674	-0.447	1.05E-07	3.67E-07
NUFIP2	nuclear FMR1 interacting protein 2	protein_coding	chr17:29255839	10877	12.57512409	11.05705266	-0.272	1.05E-07	3.67E-07
TP53I11	tumor protein p53 inducible protein 11	protein_coding	chr11:44885903	7345	10.05106377	8.245952541	-0.371	1.05E-07	3.67E-07
USP47	ubiquitin specific peptidase 47	protein_coding	chr11:11841423	11158	11.20234807	9.572187647	-0.313	1.06E-07	3.68E-07
BCOR	BCL6 corepressor	protein_coding	chrX:40049815-	8544	11.72703804	9.137644825	-0.445	1.06E-07	3.69E-07
SLC27A4	solute carrier family 27 member 4	protein_coding	chr9:128340527	3229	12.23426879	10.21285829	-0.346	1.06E-07	3.69E-07
GUSB	glucuronidase beta	protein_coding	chr7:65960684-	3893	4.077196934	3.013762277	-0.52	1.07E-07	3.71E-07
FLCN	folliculin	protein_coding	chr17:17212212	9786	4.339112696	3.216492528	-0.514	1.07E-07	3.71E-07
RPS6KA1	ribosomal protein S6 kinase A1	protein_coding	chr1:26529761-	5884	1.094664615	0.680611022	-0.77	1.08E-07	3.76E-07
EPC1	enhancer of polycomb homolog 1	protein_coding	chr10:32267751	11286	3.123700566	2.629154461	-0.334	1.09E-07	3.77E-07
AC004803.1	novel transcript	lncRNA	chr12:974133-9	3669	2.822826791	2.142547307	-0.484	1.09E-07	3.79E-07
NOMO3	NODAL modulator 3	protein_coding	chr16:16232528	8238	2.519560787	1.93860087	-0.462	1.10E-07	3.80E-07
TRAPPC6B	trafficking protein particle complex 6B	protein_coding	chr14:39147811	4533	8.28960616	6.935251574	-0.343	1.10E-07	3.82E-07

ZNF646	zinc finger protein 646	protein_coding	chr16:31074422	8635	2.684666097	1.954690907	-0.54	1.11E-07	3.83E-07
TMEM201	transmembrane protein 201	protein_coding	chr1:9588911-9	6981	6.254649785	5.053816956	-0.396	1.11E-07	3.84E-07
VPS16	VPS16 core subunit of CORVET and HOPS c	protein_coding	chr20:2840703-	3364	5.856648516	4.483436542	-0.471	1.11E-07	3.85E-07
ZNF837	zinc finger protein 837	protein_coding	chr19:58367618	2046	1.806981832	0.871284029	-1.159	1.12E-07	3.87E-07
MROH1	maestro heat like repeat family member 1	protein_coding	chr8:14414801€	10903	9.226897451	6.855246499	-0.512	1.12E-07	3.88E-07
ARRDC3	arrestin domain containing 3	protein_coding	chr5:91368631-	4985	8.292540249	6.826887452	-0.365	1.12E-07	3.88E-07
ZNF843	zinc finger protein 843	protein_coding	chr16:31432593	4568	1.113478136	0.670996544	-0.809	1.13E-07	3.89E-07
CENPC	centromere protein C	protein_coding	chr4:67468762-	8496	1.845770076	1.36236834	-0.525	1.13E-07	3.90E-07
E2F3	E2F transcription factor 3	protein_coding	chr6:20401879-	5190	5.791199272	4.830269954	-0.348	1.13E-07	3.90E-07
AK9	adenylate kinase 9	protein_coding	chr6:10949285€	10098	1.708476438	1.322976136	-0.452	1.13E-07	3.91E-07
HMGA1	high mobility group AT-hook 1	protein_coding	chr6:34236873-	2327	39.01783542	32.80033722	-0.337	1.14E-07	3.92E-07
DUSP27	dual specificity phosphatase 27, atypical	protein_coding	chr1:16709404€	4304	63.48641215	47.16012504	-0.511	1.14E-07	3.92E-07
ZNF609	zinc finger protein 609	protein_coding	chr15:64460742	12112	6.832840223	5.527319477	-0.391	1.15E-07	3.94E-07
SLC25A11	solute carrier family 25 member 11	protein_coding	chr17:4937130-	2471	91.09106522	73.0284016	-0.403	1.16E-07	3.97E-07
HDAC5	histone deacetylase 5	protein_coding	chr17:44076746	7618	39.23419198	33.51541033	-0.313	1.16E-07	3.98E-07
PSMD2	proteasome 26S subunit, non-ATPase 2	protein_coding	chr3:18429919€	4960	47.08667413	41.46437947	-0.27	1.16E-07	3.99E-07
RANGAP1	Ran GTPase activating protein 1	protein_coding	chr22:41245611	5348	18.88677953	16.35563138	-0.294	1.18E-07	4.05E-07
FAM117B	family with sequence similarity 117 meml	protein_coding	chr2:20263496¦	6205	1.447938428	0.96552035	-0.672	1.19E-07	4.08E-07
CENPN	centromere protein N	protein_coding	chr16:81006498	3568	4.758728389	3.464532756	-0.547	1.20E-07	4.09E-07
PHC3	polyhomeotic homolog 3	protein_coding	chr3:17008673⁄	16919	3.727490151	3.087519943	-0.357	1.20E-07	4.11E-07
STX10	syntaxin 10	protein_coding	chr19:13144058	1796	12.38635044	9.987927967	-0.396	1.21E-07	4.14E-07
BTN2A2	butyrophilin subfamily 2 member A2	protein_coding	chr6:26383096-	6398	1.157998323	0.636816379	-0.947	1.23E-07	4.19E-07
UCP2	uncoupling protein 2	protein_coding	chr11:73974672	3171	11.29062158	8.867316986	-0.431	1.23E-07	4.19E-07
PRMT5	protein arginine methyltransferase 5	protein_coding	chr14:22920525	3276	27.14031554	23.4991515	-0.294	1.23E-07	4.21E-07
RABEP1	rabaptin, RAB GTPase binding effector prc	protein_coding	chr17:5282265-	7059	17.14410684	15.15217263	-0.264	1.25E-07	4.25E-07
SATB1	SATB homeobox 1	protein_coding	chr3:18345377-	13419	6.019163433	4.904901316	-0.38	1.25E-07	4.25E-07
BANCR	BRAF-activated non-protein coding RNA	lncRNA	chr9:69296682-	848	293.7380857	217.1195192	-0.517	1.25E-07	4.25E-07
SYT9	synaptotagmin 9	protein_coding	chr11:7238778-	4774	9.727104326	7.319349853	-0.497	1.25E-07	4.26E-07
RLF	rearranged L-myc fusion	protein_coding	chr1:40161387-	6232	7.263049729	5.575887129	-0.466	1.25E-07	4.27E-07
NTF4	neurotrophin 4	protein_coding	chr19:49061066	1469	3.839440585	2.230300158	-0.869	1.25E-07	4.27E-07
SERF2	small EDRK-rich factor 2	protein_coding	chr15:43777087	7347	40.80015983	30.00849781	-0.524	1.26E-07	4.30E-07
CHAF1A	chromatin assembly factor 1 subunit A	protein_coding	chr19:4402640-	4326	4.939851313	3.847864449	-0.447	1.28E-07	4.34E-07
AC012313.2	novel transcript, antisense to ZNF497	lncRNA	chr19:58357999	1154	4.141592385	2.149052293	-1.041	1.29E-07	4.37E-07
PPP5C	protein phosphatase 5 catalytic subunit	protein_coding	chr19:46346994	7059	19.62608554	17.25180998	-0.272	1.29E-07	4.38E-07
GTPBP3	GTP binding protein 3, mitochondrial	protein_coding	chr19:17334920	4528	4.708211538	3.734283454	-0.42	1.32E-07	4.48E-07
TTC7A	tetratricopeptide repeat domain 7A	protein_coding	chr2:46916157-	7180	1.514109985	1.027222977	-0.643	1.32E-07	4.48E-07
KIF22	kinesin family member 22	protein_coding	chr16:29790719	4312	7.560390462	6.002089628	-0.418	1.32E-07	4.48E-07
GOLGA7	golgin A7	protein_coding	chr8:41490396-	2474	38.75394678	31.91816329	-0.365	1.35E-07	4.57E-07
ATF4	activating transcription factor 4	protein_coding	chr22:39519695	2514	76.59408985	61.97135007	-0.39	1.36E-07	4.59E-07
PHF6	PHD finger protein 6	protein_coding	chrX:134373253	8016	5.030118039	4.237711781	-0.333	1.36E-07	4.61E-07
NUFIP1	nuclear FMR1 interacting protein 1	protein_coding	chr13:44939249	3480	2.57936556	1.759160105	-0.64	1.37E-07	4.63E-07
HERC2	HECT and RLD domain containing E3 ubiq	protein_coding	chr15:28111040	20183	10.04598532	7.7183901	-0.463	1.38E-07	4.65E-07
PCDHGC3	protocadherin gamma subfamily C, 3	protein_coding	chr5:141475947	5430	0.987690718	0.586621599	-0.838	1.38E-07	4.68E-07
STOM	stomatin	protein_coding	chr9:121338988	3198	12.54954818	10.47063869	-0.347	1.39E-07	4.70E-07
GRHPR	glyoxylate and hydroxypyruvate reductas	protein_coding	chr9:37422666-	7226	18.00237849	14.47068844	-0.4	1.40E-07	4.72E-07
TRIOBP	TRIO and F-actin binding protein	protein_coding	chr22:37697048	12189	17.60515497	14.82675051	-0.333	1.42E-07	4.80E-07
RHBDD2	rhomboid domain containing 2	protein_coding	chr7:75842602-	2390	23.8368649	19.62880402	-0.366	1.45E-07	4.88E-07
DAPK3	death associated protein kinase 3	protein_coding	chr19:3958453-	3549	43.54005689	36.88204274	-0.326	1.45E-07	4.88E-07
PPFIA1	PTPRF interacting protein alpha 1	protein_coding	chr11:70270700	11704	4.639074675	3.914567715	-0.33	1.46E-07	4.90E-07
EZH2	enhancer of zeste 2 polycomb repressive c	protein_coding	chr7:148807383	4522	4.302429012	3.449674461	-0.402	1.51E-07	5.07E-07
RAD23A	RAD23 homolog A, nucleotide excision re	protein_coding	chr19:12945855	2547	66.62582084	54.14486966	-0.384	1.52E-07	5.10E-07
CCDC122	coiled-coil domain containing 122	protein_coding	chr13:43823909	5416	1.647943059	1.199559538	-0.543	1.52E-07	5.12E-07
SV2C	synaptic vesicle glycoprotein 2C	protein_coding	chr5:76083383-	11817	3.782521606	2.79197569	-0.52	1.55E-07	5.19E-07
CHUK	component of inhibitor of nuclear factor I	protein_coding	chr10:10018830	6903	3.090067323	2.448449725	-0.422	1.55E-07	5.19E-07
ENSA	endosulfine alpha	protein_coding	chr1:150600851	5493	17.11617038	14.99247917	-0.277	1.55E-07	5.19E-07
GSTM4	glutathione S-transferase mu 4	protein_coding	chr1:109656099	5727	8.846440993	6.982290676	-0.428	1.55E-07	5.20E-07
EXOC8	exocyst complex component 8	protein_coding	chr1:231332753	5100	4.234106545	3.455301037	-0.38	1.56E-07	5.22E-07
TELO2	telomere maintenance 2	protein_coding	chr16:1493344-	4239	13.25990391	10.17679844	-0.467	1.58E-07	5.29E-07
RNF169	ring finger protein 169	protein_coding	chr11:74748849	7842	2.567649676	2.092685713	-0.382	1.58E-07	5.29E-07
ALAS1	5'-aminolevulinate synthase 1	protein_coding	chr3:52198086-	2536	50.19914673	42.7132555	-0.318	1.60E-07	5.34E-07
SETDB2	SET domain bifurcated histone lysine metl	protein_coding	chr13:49444374	6678	4.338257356	3.412879941	-0.428	1.60E-07	5.35E-07
HELQ	helicase, POLQ like	protein_coding	chr4:83407343-	3712	3.819154912	2.904214236	-0.477	1.60E-07	5.35E-07
SMIM13	small integral membrane protein 13	protein_coding	chr6:11093834-	4887	9.888169639	8.565413594	-0.292	1.61E-07	5.38E-07
HCN4	hyperpolarization activated cyclic nucleo	protein_coding	chr15:73319859	6922	47.64962197	34.16551193	-0.56	1.62E-07	5.41E-07
EZR	ezrin	protein_coding	chr6:158765741	3298	173.9214014	151.9737667	-0.281	1.63E-07	5.42E-07
TMPO-AS1	TMPO antisense RNA 1	lncRNA	chr12:98512973	3357	2.014569385	1.23932414	-0.793	1.63E-07	5.44E-07
EPB41L4A	erythrocyte membrane protein band 4.1 l	protein_coding	chr5:112142441	8579	3.826729574	3.011441503	-0.43	1.65E-07	5.48E-07
ZNF764	zinc finger protein 764	protein_coding	chr16:30553764	3113	8.646171329	7.040746067	-0.383	1.66E-07	5.53E-07
SLC18B1	solute carrier family 18 member B1	protein_coding	chr6:132769370	2844	6.282668663	4.698217898	-0.506	1.67E-07	5.55E-07
ADAM15	ADAM metallopeptidase domain 15	protein_coding	chr1:155050566	6584	3.641593237	2.991958828	-0.369	1.68E-07	5.57E-07
CMTM8	CKLF like MARVEL transmembrane domaiı	protein_coding	chr3:32238679-	1170	1.122259946	0.393424218	-1.602	1.69E-07	5.62E-07
SOCS3	suppressor of cytokine signaling 3	protein_coding	chr17:78356778	2734	3.05279175	1.901710036	-0.764	1.71E-07	5.68E-07
ERMP1	endoplasmic reticulum metallopeptidase	protein_coding	chr9:5765076-5	6528	7.892399452	6.577817548	-0.348	1.72E-07	5.70E-07
AC116351.1	novel transcript	lncRNA	chr5:987180-99	3482	5.115994308	4.105789878	-0.406	1.72E-07	5.72E-07
TBL1X	transducin beta like 1 X-linked	protein_coding	chrX:9463295-9	9039	2.989927265	2.344697197	-0.435	1.73E-07	5.74E-07
CIP2A	cell proliferation regulating inhibitor of p	protein_coding	chr3:108549864	4584	2.203278268	1.507862349	-0.639	1.73E-07	5.74E-07
GATA6	GATA binding protein 6	protein_coding	chr18:22169392	3996	49.14971817	40.52977647	-0.363	1.73E-07	5.74E-07
RHBDD1	rhomboid domain containing 1	protein_coding	chr2:226835581	6926	2.236697434	1.677286034	-0.502	1.74E-07	5.77E-07
APLP1	amyloid beta precursor like protein 1	protein_coding	chr19:35867899	3656	12.78782588	10.7272701	-0.339	1.75E-07	5.80E-07
ZNF8	zinc finger protein 8	protein_coding	chr19:58278955	9110	2.26784028	1.680179199	-0.519	1.76E-07	5.82E-07
RIDA	reactive intermediate imine deaminase A I	protein_coding	chr8:98102344-	1497	13.69330367	9.636336144	-0.593	1.80E-07	5.93E-07
DYRK3	dual specificity tyrosine phosphorylation	protein_coding	chr1:206635536	10314	2.808583588	2.324541817	-0.359	1.81E-07	5.97E-07
UPF1	UPF1 RNA helicase and ATPase	protein_coding	chr19:18831938	8600	13.36710285	11.13842076	-0.348	1.82E-07	5.98E-07
MARVELD1	MARVEL domain containing 1	protein_coding	chr10:97713173	3371	13.22716457	10.66737934	-0.396	1.84E-07	6.05E-07
UBE2QL1	ubiquitin conjugating enzyme E2 Q family	protein_coding	chr5:6448859-6	5895	2.415332738	1.862354667	-0.459	1.85E-07	6.07E-07
FERMT1	fermitin family member 1	protein_coding	chr20:6074845-	5359	2.156851157	1.565904413	-0.548	1.85E-07	6.07E-07
SHQ1	SHQ1, H/ACA ribonucleoprotein assembly	protein_coding	chr3:72749277-	5833	2.608469415	2.073916834	-0.415	1.85E-07	6.08E-07
AL731567.1	novel transcript	lncRNA	chr10:45444570	462	0.855223576	0.056890871	-3.821	1.89E-07	6.19E-07
ENO3	enolase 3	protein_coding	chr17:4948092-	4292	200.8975666	167.8039592	-0.345	1.89E-07	6.19E-07
HOOK2	hook microtubule tethering protein 2	protein_coding	chr19:12763003	5950	10.55459417	8.290553836	-0.434	1.91E-07	6.25E-07
NDNF	neuron derived neurotrophic factor	protein_coding	chr4:121035613	3573	2.022827834	1.263390596	-0.765	1.93E-07	6.30E-07
ADAMTS9	ADAM metallopeptidase with thrombosp	protein_coding	chr3:64515654-	10680	10.0748941	8.377696446	-0.351	1.94E-07	6.35E-07
ABCD4	ATP binding cassette subfamily D member	protein_coding	chr14:74285269	8922	4.736867999	3.849220319	-0.386	1.95E-07	6.36E-07
EYA4	EYA transcriptional coactivator and phosp	protein_coding	chr6:133240598	8976	1.566296128	0.97541736	-0.773	1.96E-07	6.40E-07
PRDM11	PR/SET domain 11	protein_coding	chr11:45095806	13940	5.118853599	3.704017506	-0.547	1.96E-07	6.40E-07
ZBTB8OS	zinc finger and BTB domain containing 8 c	protein_coding	chr1:32600172-	3531	4.377560557	3.410997688	-0.447	1.97E-07	6.42E-07
VPS36	vacuolar protein sorting 36 homolog	protein_coding	chr13:52412602	7299	17.34665521	14.89505488	-0.305	1.99E-07	6.47E-07
SDF4	stromal cell derived factor 4	protein_coding	chr1:1216908-1	4745	35.12028375	31.0361556	-0.265	2.00E-07	6.50E-07
BRD1	bromodomain containing 1	protein_coding	chr22:49773283	5850	8.130888479	6.606268215	-0.384	2.00E-07	6.51E-07
ANKRD18A	ankyrin repeat domain 18A	protein_coding	chr9:38571358-	5239	2.444221974	1.694481288	-0.612	2.01E-07	6.54E-07
ARL6IP5	ADP ribosylation factor like GTPase 6 inte	protein_coding	chr3:69084937-	2555	90.95278683	73.00399292	-0.401	2.04E-07	6.63E-07
NDC80	NDC80 kinetochore complex component	protein_coding	chr18:2571557-	3554	3.517392846	2.391308778	-0.641	2.08E-07	6.75E-07
ZNF780A	zinc finger protein 780A	protein_coding	chr19:40069152	7972	1.496876429	1.082700705	-0.55	2.08E-07	6.76E-07
ALDH3A2	aldehyde dehydrogenase 3 family membe	protein_coding	chr17:19648136	6457	16.89971426	14.54055217	-0.302	2.09E-07	6.78E-07
AC107294.3	novel transcript	lncRNA	chr3:184741937	526	1.976237605	0.408459271	-2.329	2.10E-07	6.80E-07
MIEF2	mitochondrial elongation factor 2	protein_coding	chr17:18260597	3906	11.19914052	9.399703781	-0.34	2.11E-07	6.84E-07
PATZ1	POZ/BTB and AT hook containing zinc fing	protein_coding	chr22:31325804	5807	6.529586815	5.62970672	-0.3	2.11E-07	6.85E-07
ACOT8	acyl-CoA thioesterase 8	protein_coding	chr20:45841721	4354	5.135512901	4.211690805	-0.372	2.13E-07	6.90E-07
STMN1	stathmin 1	protein_coding	chr1:25884181-	6247	17.17329757	12.11640388	-0.583	2.14E-07	6.94E-07

KIFC3	kinesin family member C3	protein_coding	chr16:57758217	9600	4.915291975	3.836288302	-0.44	2.17E-07	7.02E-07
ZNF25	zinc finger protein 25	protein_coding	chr10:37949573	4257	2.908347079	2.280309637	-0.435	2.21E-07	7.14E-07
MYL7	myosin light chain 7	protein_coding	chr7:44138864-	1458	6435.436456	5075.926201	-0.426	2.21E-07	7.15E-07
AXIN2	axin 2	protein_coding	chr17:65528563	5227	2.606683659	2.008242778	-0.463	2.24E-07	7.22E-07
CENPK	centromere protein K	protein_coding	chr5:65517766-	4309	1.694871763	1.178894194	-0.61	2.27E-07	7.31E-07
SCX	scleraxis bHLH transcription factor	protein_coding	chr8:144266453	1106	5.623666414	3.210582636	-0.906	2.27E-07	7.31E-07
C9orf72	C9orf72-SMCR8 complex subunit	protein_coding	chr9:27546545-	6332	2.604144262	2.018091881	-0.456	2.29E-07	7.36E-07
DGAT1	diacylglycerol O-acyltransferase 1	protein_coding	chr8:144314584	4131	9.559256826	7.429244028	-0.454	2.29E-07	7.37E-07
AL035252.5	novel transcript	lncRNA	chr20:25184658	1670	1.69730478	0.65454708	-1.454	2.29E-07	7.38E-07
REST	RE1 silencing transcription factor	protein_coding	chr4:56907876-	7983	9.492461096	8.260127694	-0.287	2.30E-07	7.41E-07
NAA25	N(alpha)-acetyltransferase 25, NatB auxilia	protein_coding	chr12:11202668	9902	6.733274458	5.746861063	-0.315	2.31E-07	7.43E-07
ATP10D	ATPase phospholipid transporting 10D (pu	protein_coding	chr4:47485288-	7965	2.212425607	1.734077212	-0.436	2.33E-07	7.47E-07
ATP6V0D1	ATPase H+ transporting V0 subunit d1	protein_coding	chr16:67438014	8156	19.7565149	16.1837946	-0.372	2.33E-07	7.48E-07
ZNF587B	zinc finger protein 587B	protein_coding	chr19:57819721	5312	2.306305885	1.721706014	-0.51	2.33E-07	7.48E-07
DSN1	DSN1 component of MIS12 kinetochore c	protein_coding	chr20:36751791	2739	3.921603485	2.888683424	-0.525	2.33E-07	7.49E-07
NCAM1	neural cell adhesion molecule 1	protein_coding	chr11:11296124	12737	61.8831012	50.39390651	-0.381	2.33E-07	7.49E-07
SPATA5	spermatogenesis associated 5	protein_coding	chr4:122923078	8226	1.03231961	0.723605117	-0.595	2.34E-07	7.52E-07
ADGRA3	adhesion G protein-coupled receptor A3	protein_coding	chr4:22345071-	9261	6.995404841	5.963159174	-0.317	2.35E-07	7.53E-07
GMCL1	germ cell-less 1, spermatogenesis associat	protein_coding	chr2:69829660-	4892	4.487705573	3.711161137	-0.36	2.36E-07	7.56E-07
PIGW	phosphatidylinositol glycan anchor biosy	protein_coding	chr17:36534987	2832	5.429383099	3.977978467	-0.531	2.37E-07	7.58E-07
PI4KA	phosphatidylinositol 4-kinase alpha	protein_coding	chr22:20707691	11407	12.2970842	10.07412375	-0.372	2.38E-07	7.63E-07
CLCN6	chloride voltage-gated channel 6	protein_coding	chr1:11806096-	8173	8.224457754	6.974643085	-0.322	2.40E-07	7.68E-07
POLR1A	RNA polymerase I subunit A	protein_coding	chr2:86020216-	13519	7.900165097	6.78669179	-0.305	2.43E-07	7.77E-07
IQSEC1	IQ motif and Sec7 domain 1	protein_coding	chr3:12897043-	10181	19.46835032	15.69726628	-0.396	2.46E-07	7.85E-07
RIMS3	regulating synaptic membrane exocytosis	protein_coding	chr1:40620680-	7259	2.15575622	1.383124549	-0.717	2.48E-07	7.92E-07
MAML1	mastermind like transcriptional coactivat	protein_coding	chr5:179732822	5967	9.776985641	8.005795343	-0.373	2.49E-07	7.94E-07
UNK	unk zinc finger	protein_coding	chr17:75784806	5540	8.276966373	7.153660991	-0.296	2.49E-07	7.95E-07
BHLHE41	basic helix-loop-helix family member e41	protein_coding	chr12:26120030	3854	1.320031177	0.757762402	-0.879	2.55E-07	8.15E-07
ATP1A3	ATPase Na+/K+ transporting subunit alpha	protein_coding	chr19:41966582	5983	2.186792049	0.979042513	-1.214	2.56E-07	8.16E-07
CDC42EP4	CDC42 effector protein 4	protein_coding	chr17:73283624	4837	2.722780046	1.619049714	-0.833	2.57E-07	8.20E-07
AC124798.1	antisense to KCNJ11 and overlapping to a	lncRNA	chr11:17380649	2883	1.679707912	0.927791969	-0.937	2.59E-07	8.25E-07
COBL	cordon-bleu WH2 repeat protein	protein_coding	chr7:51016212-	14349	3.144117885	2.54887139	-0.388	2.60E-07	8.27E-07
SWT1	SWT1 RNA endoribonuclease homolog	protein_coding	chr1:185157080	4068	2.378724897	1.716658204	-0.553	2.60E-07	8.27E-07
XPO5	exportin 5	protein_coding	chr6:43522334-	8481	5.787264415	4.860955437	-0.338	2.61E-07	8.28E-07
MMP11	matrix metallopeptidase 11	protein_coding	chr22:23768226	5149	2.201210862	1.603542012	-0.539	2.61E-07	8.29E-07
FHOD3	formin homology 2 domain containing 3	protein_coding	chr18:36297714	7965	139.1393622	119.5331887	-0.305	2.62E-07	8.31E-07
CCHCR1	coiled-coil alpha-helical rod protein 1	protein_coding	chr6:31142439-	5182	3.047339556	2.452948418	-0.398	2.64E-07	8.38E-07
AC018647.2	novel transcript	lncRNA	chr7:35695214-	4200	3.241356653	2.232078173	-0.619	2.67E-07	8.47E-07
EPG5	ectopic P-granules autophagy protein 5 ho	protein_coding	chr18:45847609	13730	7.806166204	6.521605888	-0.344	2.67E-07	8.47E-07
MRE11	MRE11 homolog, double strand break rep	protein_coding	chr11:94415578	7689	2.529791313	2.02656748	-0.404	2.71E-07	8.56E-07
PXDC1	PX domain containing 1	protein_coding	chr6:3722614-3	2474	18.87533822	15.62657616	-0.357	2.75E-07	8.69E-07
TMEM126B	transmembrane protein 126B	protein_coding	chr11:85628573	2416	19.91068942	15.60936323	-0.436	2.75E-07	8.70E-07
PPM1F	protein phosphatase, Mg2+/Mn2+ depend	protein_coding	chr22:21919425	10089	3.469528291	2.683747284	-0.454	2.76E-07	8.71E-07
CSDC2	cold shock domain containing C2	protein_coding	chr22:41561010	3003	5.376493047	3.722508981	-0.614	2.80E-07	8.85E-07
PCCA	propionyl-CoA carboxylase subunit alpha	protein_coding	chr13:10008901	7288	4.248288816	3.636848227	-0.311	2.80E-07	8.85E-07
SPINT2	serine peptidase inhibitor, Kunitz type 2	protein_coding	chr19:38244035	5085	4.341927635	3.268169055	-0.497	2.80E-07	8.85E-07
MITF	melanocyte inducing transcription factor	protein_coding	chr3:69739435-	7181	13.65608576	12.05941218	-0.266	2.87E-07	9.04E-07
EFL1	elongation factor like GTPase 1	protein_coding	chr15:82130233	4708	5.287268613	4.328315993	-0.372	2.88E-07	9.07E-07
HIVEP1	human immunodeficiency virus type I enh	protein_coding	chr6:12008762-	11005	3.037894371	2.241640235	-0.522	2.90E-07	9.12E-07
FKBP4	FKBP prolyl isomerase 4	protein_coding	chr12:2794970-	5163	35.65224454	31.33643016	-0.273	2.93E-07	9.22E-07
NCAPG2	non-SMC condensin II complex subunit G2	protein_coding	chr7:158631311	6988	2.246323948	1.780866545	-0.42	2.93E-07	9.22E-07
ATF3	activating transcription factor 3	protein_coding	chr1:212565334 4040		3.724263775	2.492008425	-0.67	2.95E-07	9.27E-07
CYBA	cytochrome b-245 alpha chain	protein_coding	chr16:88643289	3253	5.133060589	3.825841504	-0.507	3.00E-07	9.41E-07
VRK3	VRK serine/threonine kinase 3	protein_coding	chr19:49976468	9795	2.668037732	1.997062188	-0.503	3.02E-07	9.48E-07
HNRNPUL2	heterogeneous nuclear ribonucleoprotein	protein_coding	chr11:62712630	5416	6.142488475	5.023321227	-0.378	3.02E-07	9.48E-07
PIGX	phosphatidylinositol glycan anchor biosy	protein_coding	chr3:196639775	4446	5.875563759	4.717764153	-0.403	3.04E-07	9.54E-07
AP000919.4	novel transcript, antisense to LPIN2	lncRNA	chr18:2920966-	720	1.225414144	0.353156785	-1.841	3.07E-07	9.60E-07
VPS37A	VPS37A subunit of ESCRT-I	protein_coding	chr8:17246931-	6424	9.767998768	8.55610909	-0.277	3.07E-07	9.61E-07
USP22	ubiquitin specific peptidase 22	protein_coding	chr17:20999596	6777	66.5471366	57.33923563	-0.3	3.08E-07	9.64E-07
CDH24	cadherin 24	protein_coding	chr14:23047062	3748	1.32507776	0.725531553	-0.951	3.09E-07	9.67E-07
FABP2	fatty acid binding protein 2	protein_coding	chr4:119317250	2478	2.14323045	1.248559948	-0.868	3.10E-07	9.69E-07
COA7	cytochrome c oxidase assembly factor 7 (p	protein_coding	chr1:52684449-	4148	7.129227011	6.018407014	-0.329	3.11E-07	9.72E-07
RUNDC1	RUN domain containing 1	protein_coding	chr17:42980565	4757	8.661254594	7.499368199	-0.294	3.11E-07	9.73E-07
HOMER3	homer scaffold protein 3	protein_coding	chr19:18929201	3422	2.815197327	2.149659245	-0.473	3.15E-07	9.83E-07
TOGARAM1	TOG array regulator of axonemal microtul	protein_coding	chr14:44962190	10292	5.098584637	4.434086821	-0.287	3.17E-07	9.89E-07
ZNF684	zinc finger protein 684	protein_coding	chr1:40531573-	3101	3.293133148	2.0993161	-0.73	3.17E-07	9.90E-07
LGALS8	galectin 8	protein_coding	chr1:236518000	9710	4.496941211	3.74934425	-0.349	3.19E-07	9.93E-07
CCNA2	cyclin A2	protein_coding	chr4:121816444	2798	10.32056688	7.612303765	-0.527	3.19E-07	9.93E-07
TYW3	tRNA-yW synthesizing protein 3 homolog	protein_coding	chr1:74733152-	4616	4.795383276	3.757727079	-0.444	3.19E-07	9.94E-07
LINC01521	long intergenic non-protein coding RNA 1	lncRNA	chr22:31346777	1943	5.52583835	3.791256729	-0.625	3.23E-07	1.01E-06
CLCN7	chloride voltage-gated channel 7	protein_coding	chr16:1444934-	7623	11.56661184	9.700317119	-0.339	3.25E-07	1.01E-06
GIGYF2	GRB10 interacting GYF protein 2	protein_coding	chr2:232697299	13416	6.283333194	5.353224508	-0.317	3.29E-07	1.02E-06
ZNF425	zinc finger protein 425	protein_coding	chr7:149102784	3665	1.501938107	0.809514115	-0.97	3.31E-07	1.03E-06
SNU13	small nuclear ribonucleoprotein 13	protein_coding	chr22:41673933	2927	44.31270481	37.97411865	-0.309	3.32E-07	1.03E-06
ACAP3	ArfGAP with coiled-coil, ankyrin repeat an	protein_coding	chr1:1292390-1	8707	11.37702975	8.636946574	-0.482	3.35E-07	1.04E-06
LOXL1	lysyl oxidase like 1	protein_coding	chr15:73925989	3637	3.807032222	2.664396897	-0.597	3.35E-07	1.04E-06
MFSD12	major facilitator superfamily domain cont	protein_coding	chr19:3538261-	5290	5.612611482	4.519024728	-0.397	3.36E-07	1.04E-06
MROH8	maestro heat like repeat family member 8	protein_coding	chr20:37101226	3878	2.139459871	1.561825618	-0.541	3.36E-07	1.04E-06
PDE4B	phosphodiesterase 4B	protein_coding	chr1:65792514-	8930	1.141599026	0.801732291	-0.597	3.41E-07	1.05E-06
BSG	basigin (Ok blood group)	protein_coding	chr19:571277-5	3797	189.5652919	161.123087	-0.32	3.41E-07	1.06E-06
AKAP10	A-kinase anchoring protein 10	protein_coding	chr17:19904302	4839	7.278079981	6.308332968	-0.293	3.45E-07	1.07E-06
MDFI	MyoD family inhibitor	protein_coding	chr6:41636882-	2537	3.52141843	2.53843151	-0.559	3.48E-07	1.07E-06
RAB8A	RAB8A, member RAS oncogene family	protein_coding	chr19:16111889	4931	8.551523665	7.155428457	-0.343	3.48E-07	1.08E-06
ZNF706	zinc finger protein 706	protein_coding	chr8:101177878	7393	3.435696189	2.748853537	-0.409	3.49E-07	1.08E-06
CHST11	carbohydrate sulfotransferase 11	protein_coding	chr12:10445529	6492	3.647464578	2.931548965	-0.404	3.53E-07	1.09E-06
RRP8	ribosomal RNA processing 8	protein_coding	chr11:6595072-	6921	3.686461309	3.041761137	-0.362	3.55E-07	1.10E-06
ZNF76	zinc finger protein 76	protein_coding	chr6:35258909-	6782	8.397773725	6.883165266	-0.372	3.58E-07	1.10E-06
PHETA1	PH domain containing endocytic trafficki	protein_coding	chr12:11136065	3361	8.760735398	7.134387391	-0.385	3.62E-07	1.11E-06
BORCS6	BLOC-1 related complex subunit 6	protein_coding	chr17:8188333-	2575	6.717797607	4.783533512	-0.573	3.70E-07	1.14E-06
EGR1	early growth response 1	protein_coding	chr5:138465479	3137	5.41962188	3.722663411	-0.623	3.70E-07	1.14E-06
SHCBP1	SHC binding and spindle associated 1	protein_coding	chr16:46578591	6175	2.640809132	1.876192041	-0.581	3.71E-07	1.14E-06
MYH9	myosin heavy chain 9	protein_coding	chr22:36281277	9012	89.08686193	74.72293545	-0.339	3.71E-07	1.14E-06
IFT20	intraflagellar transport 20	protein_coding	chr17:28328325	3227	4.336832218	3.288845956	-0.482	3.72E-07	1.15E-06
PIK3IP1	phosphoinositide-3-kinase interacting pro	protein_coding	chr22:31281593	3805	3.252471754	2.407189062	-0.518	3.76E-07	1.16E-06
CENPE	centromere protein E	protein_coding	chr4:103105806	9444	1.96102221	1.384880393	-0.587	3.77E-07	1.16E-06
CEP57	centrosomal protein 57	protein_coding	chr11:95789965	7101	7.150872462	5.982398223	-0.345	3.78E-07	1.16E-06
MAML2	mastermind like transcriptional coactivat	protein_coding	chr11:95976598	7124	2.177246178	1.490037549	-0.631	3.84E-07	1.18E-06
COX20	cytochrome c oxidase assembly factor CO:	protein_coding	chr1:244835616	4570	13.44489227	11.24707396	-0.343	3.90E-07	1.20E-06
DNM1L	dynamin 1 like	protein_coding	chr12:32679200	7282	17.59427533	15.53657388	-0.265	3.92E-07	1.20E-06
HLA-A	major histocompatibility complex, class I	protein_coding	chr6:29941260-	2710	21.31035975	17.30905465	-0.383	3.92E-07	1.20E-06
CCDC160	coiled-coil domain containing 160	protein_coding	chrX:134237047	1790	2.551643169	1.385742529	-0.979	3.94E-07	1.21E-06
MYOZ2	myozenin 2	protein_coding	chr4:119135784	2597	260.6290741	206.4769067	-0.42	3.98E-07	1.22E-06
LAMA5	laminin subunit alpha 5	protein_coding	chr20:62307955	14291	28.94010113	19.44871484	-0.653	4.02E-07	1.23E-06
SGO2	shugoshin 2	protein_coding	chr2:200510008	6336	2.013468923	1.3815365	-0.629	4.04E-07	1.23E-06
NSD1	nuclear receptor binding SET domain prot	protein_coding	chr5:177133025	15493	10.51524597	8.238329763	-0.435	4.06E-07	1.24E-06
TXLNA	taxilin alpha	protein_coding	chr1:32179675-	5114	13.19145066	10.86715738	-0.365	4.07E-07	1.24E-06
KIAA1143	KIAA1143	protein_coding	chr3:44737661-	5216	9.953648905	8.599670768	-0.298	4.08E-07	1.25E-06
CALCOCO1	calcium binding and coiled-coil domain 1	protein_coding	chr12:53708517	8898	15.03457645	12.94901203	-0.301	4.11E-07	1.26E-06
PARP14	poly(ADP-ribose) polymerase family meml	protein_coding	chr3:122680839	10274	1.131057562	0.850114526	-0.499	4.13E-07	1.26E-06

KAT2B	lysine acetyltransferase 2B	protein_coding	chr3:20040446-	5211	8.333114034	7.291632287	-0.279	4.20E-07	1.28E-06
AC051618.1	novel transcript	lncRNA	chr10:54864674	207	4.89846615	1.094278799	-2.2	4.22E-07	1.28E-06
PARD3	par-3 family cell polarity regulator	protein_coding	chr10:34109560	6513	9.003996032	7.655199234	-0.32	4.22E-07	1.28E-06
HDDC2	HD domain containing 2	protein_coding	chr6:125219962	5888	9.637124805	7.147220612	-0.512	4.24E-07	1.29E-06
EXO1	exonuclease 1	protein_coding	chr1:241847967	4137	1.392077658	0.816863972	-0.849	4.25E-07	1.29E-06
ZNF618	zinc finger protein 618	protein_coding	chr9:113876282	10384	2.089633661	1.547224234	-0.515	4.28E-07	1.30E-06
HECW2	HECT, C2 and WW domain containing E3 u	protein_coding	chr2:196189099	23675	3.925499356	3.216061953	-0.373	4.32E-07	1.31E-06
JMJD8	jumonji domain containing 8	protein_coding	chr16:681670-6	2628	25.64761573	22.58790738	-0.27	4.32E-07	1.31E-06
TERF1	telomeric repeat binding factor 1	protein_coding	chr8:73008864-	8832	4.759059324	4.142148317	-0.288	4.33E-07	1.31E-06
LMAN2L	lectin, mannose binding 2 like	protein_coding	chr2:96705929-	2709	10.52592954	8.547028486	-0.388	4.34E-07	1.32E-06
LRRC28	leucine rich repeat containing 28	protein_coding	chr15:99251362	9279	1.303229051	1.020133853	-0.441	4.38E-07	1.33E-06
SREBF1	sterol regulatory element binding transcri	protein_coding	chr17:17810399	8243	5.502706057	3.93999975	-0.562	4.40E-07	1.33E-06
ATG2A	autophagy related 2A	protein_coding	chr11:64894546	7833	8.205981537	6.841180041	-0.347	4.42E-07	1.34E-06
SMURF1	SMAD specific E3 ubiquitin protein ligase	protein_coding	chr7:99027438-	6276	8.724523567	7.35624034	-0.331	4.42E-07	1.34E-06
WDR77	WD repeat domain 77	protein_coding	chr1:111439890	2685	15.25780525	13.06476024	-0.311	4.47E-07	1.35E-06
AL137002.2	uncharacterized LOC102724474	lncRNA	chr13:11314943	2238	1.541720615	0.851313463	-0.939	4.49E-07	1.36E-06
CYBRD1	cytochrome b reductase 1	protein_coding	chr2:171522247	5172	1.158634054	0.700309933	-0.802	4.50E-07	1.36E-06
AC092718.4	novel transcript	lncRNA	chr16:81030770	716	6.336329402	3.652396983	-0.884	4.54E-07	1.37E-06
PRXL2A	peroxiredoxin like 2A	protein_coding	chr10:80407829	6520	11.8854784	10.4106581	-0.276	4.55E-07	1.37E-06
SLC35A5	solute carrier family 35 member A5	protein_coding	chr3:112561709	4828	4.380168746	3.610917541	-0.367	4.56E-07	1.37E-06
RAB3A	RAB3A, member RAS oncogene family	protein_coding	chr19:18196784	1818	13.81645466	10.32596877	-0.499	4.71E-07	1.41E-06
IP6K3	inositol hexakisphosphate kinase 3	protein_coding	chr6:33721662-	2793	1.334795106	0.642607091	-1.14	4.77E-07	1.43E-06
AC006538.1	thimet oligopeptidase 1	protein_coding	chr19:2785503-	7421	8.208800252	7.006859038	-0.316	4.77E-07	1.43E-06
GRB10	growth factor receptor bound protein 10	protein_coding	chr7:50590063-	9795	3.546975719	2.863098388	-0.395	4.79E-07	1.44E-06
MSX1	msh homeobox 1	protein_coding	chr4:4859665-4	2121	7.091902231	5.456888902	-0.469	4.81E-07	1.44E-06
PTPRD	protein tyrosine phosphatase receptor typ	protein_coding	chr9:8314246-1	11198	7.583699603	6.050052783	-0.409	4.82E-07	1.45E-06
SNRNP70	small nuclear ribonucleoprotein U1 subu	protein_coding	chr19:49085419	4684	75.43979962	65.13093893	-0.298	4.83E-07	1.45E-06
ATP6V1A	ATPase H+ transporting V1 subunit A	protein_coding	chr3:113747033	5116	8.726906623	7.415559517	-0.319	4.85E-07	1.45E-06
PAIP2B	poly(A) binding protein interacting protei	protein_coding	chr2:71182738-	6300	10.35093452	8.837460343	-0.314	4.86E-07	1.46E-06
PANX1	pannexin 1	protein_coding	chr11:94128841	2852	7.666729235	6.394990179	-0.349	4.89E-07	1.46E-06
CPLX1	complexin 1	protein_coding	chr4:784957-82	2349	1.356654512	0.551877501	-1.351	4.90E-07	1.47E-06
TTC23	tetratricopeptide repeat domain 23	protein_coding	chr15:99136323	6148	2.662139772	2.180343285	-0.374	4.96E-07	1.48E-06
NEK2	NIMA related kinase 2	protein_coding	chr1:211658657	3095	2.56369707	1.716347048	-0.653	4.97E-07	1.49E-06
HSPA14	heat shock protein family A (Hsp70) meml	protein_coding	chr10:14838160	4924	7.760579751	6.744799726	-0.29	5.03E-07	1.50E-06
SYNRG	synergin gamma	protein_coding	chr17:37514807	14243	3.137686698	2.610507349	-0.35	5.05E-07	1.51E-06
KIF15	kinesin family member 15	protein_coding	chr3:44761721-	5281	1.080032379	0.701952227	-0.701	5.05E-07	1.51E-06
MTERF2	mitochondrial transcription termination	protein_coding	chr12:10697727	3634	12.74189939	10.89395055	-0.313	5.07E-07	1.51E-06
POM121	POM121 transmembrane nucleoporin	protein_coding	chr7:72879365-	9822	6.668979295	5.237833823	-0.431	5.08E-07	1.51E-06
USP48	ubiquitin specific peptidase 48	protein_coding	chr1:21678298-	9497	7.424964369	6.394600426	-0.303	5.09E-07	1.52E-06
CD151	CD151 molecule (Raph blood group)	protein_coding	chr11:832887-8	3894	68.11658357	59.76243937	-0.275	5.10E-07	1.52E-06
CTSD	cathepsin D	protein_coding	chr11:1752755-	3079	20.21310983	16.77851331	-0.353	5.13E-07	1.53E-06
ATP5F1D	ATP synthase F1 subunit delta	protein_coding	chr19:1241746-	2249	120.931977	103.7236595	-0.307	5.19E-07	1.54E-06
PLEKHJ1	pleckstrin homology domain containing J	protein_coding	chr19:2230084-	4655	21.0197685	17.53253786	-0.349	5.21E-07	1.55E-06
MCM7	minichromosome maintenance complex	protein_coding	chr7:100092728	4386	16.71698908	14.61354881	-0.28	5.28E-07	1.57E-06
IPO13	importin 13	protein_coding	chr1:43946950-	5072	9.224010416	7.814539121	-0.326	5.31E-07	1.58E-06
CEP76	centrosomal protein 76	protein_coding	chr18:12661833	6158	2.060040189	1.589948024	-0.46	5.33E-07	1.58E-06
AC003112.1	transmembrane protein 59 like	protein_coding	chr19:18607430	2626	1.084279997	0.45905474	-1.303	5.37E-07	1.59E-06
LRP10	LDL receptor related protein 10	protein_coding	chr14:22871740	7118	13.43193433	11.6840373	-0.287	5.42E-07	1.61E-06
C5orf46	chromosome 5 open reading frame 46	protein_coding	chr5:147880726	1218	1.650941168	0.673810787	-1.388	5.42E-07	1.61E-06
YAF2	YY1 associated factor 2	protein_coding	chr12:42157104	10839	5.647121634	4.507470132	-0.408	5.50E-07	1.63E-06
AC009506.1	novel transcript, antisense to BAZ2B	lncRNA	chr2:159615296	1494	5.996905409	4.531196333	-0.489	5.56E-07	1.65E-06
WRB	tryptophan rich basic protein	protein_coding	chr21:39377698	8639	2.173755582	1.678883465	-0.46	5.58E-07	1.65E-06
USP51	ubiquitin specific peptidase 51	protein_coding	chrX:55484616-	4407	2.299468137	1.644428565	-0.561	5.60E-07	1.66E-06
RND3	Rho family GTPase 3	protein_coding	chr2:150468195	3594	7.576142834	6.225469003	-0.373	5.61E-07	1.66E-06
RAB31	RAB31, member RAS oncogene family	protein_coding	chr18:9708275-	5476	5.365946123	4.214689266	-0.434	5.64E-07	1.67E-06
AC093827.4	Novel transcript, antisense to AFF1	lncRNA	chr4:86924630-	2801	1.759872187	1.184378802	-0.657	5.69E-07	1.68E-06
SH3BP5	SH3 domain binding protein 5	protein_coding	chr3:15254353-	4616	7.117983675	5.679995971	-0.415	5.76E-07	1.70E-06
CSPG5	chondroitin sulfate proteoglycan 5	protein_coding	chr3:47562238-	4367	3.147056976	2.366354915	-0.499	5.76E-07	1.70E-06
CDCA2	cell division cycle associated 2	protein_coding	chr8:25459199-	3697	2.258887038	1.466315565	-0.716	5.80E-07	1.71E-06
THBS4	thrombospondin 4	protein_coding	chr5:79991311-	4605	1.81663254	1.3079129	-0.56	5.92E-07	1.74E-06
KNTC1	kinetochore associated 1	protein_coding	chr12:12252724	11325	1.33746786	1.051013587	-0.428	5.95E-07	1.75E-06
SURF1	SURF1 cytochrome c oxidase assembly fac	protein_coding	chr9:133351758	1758	15.58894428	13.10026666	-0.339	6.01E-07	1.77E-06
HEBP1	heme binding protein 1	protein_coding	chr12:12974870	3428	11.31901277	9.378322507	-0.356	6.04E-07	1.77E-06
LSG1	large 60S subunit nuclear export GTPase 1	protein_coding	chr3:194640791	4801	5.913262595	4.665747582	-0.423	6.14E-07	1.80E-06
Z99496.2	novel transcript, antisense to CEP85L	lncRNA	chr6:118565660	906	34.21909169	26.81834826	-0.432	6.27E-07	1.84E-06
SNF8	SNF8 subunit of ESCRT-II	protein_coding	chr17:48929316	5270	9.903480117	8.461860995	-0.314	6.30E-07	1.85E-06
MVK	mevalonate kinase	protein_coding	chr12:10957325	6488	4.130458384	3.407821375	-0.366	6.31E-07	1.85E-06
PGAM4	phosphoglycerate mutase family member	protein_coding	chrX:77968874-	765	2.349629122	0.98324628	-1.326	6.31E-07	1.85E-06
PIK3CB	phosphatidylinositol-4,5-bisphosphate 3-	protein_coding	chr3:138652699	7850	5.85217442	5.066591631	-0.294	6.36E-07	1.86E-06
MCM4	minichromosome maintenance complex	protein_coding	chr8:47960185-	6973	13.97050175	11.8469657	-0.323	6.47E-07	1.89E-06
ERCC4	ERCC excision repair 4, endonuclease cata	protein_coding	chr16:13920157	8460	3.158772162	2.688596139	-0.32	6.53E-07	1.91E-06
SPSB3	splA/ryanodine receptor domain and SOC	protein_coding	chr16:1776712-	3099	12.60885534	10.81706843	-0.308	6.56E-07	1.92E-06
RHOD	ras homolog family member D	protein_coding	chr11:67056847	1240	45.60363676	36.70167005	-0.4	6.58E-07	1.92E-06
COQ3	coenzyme Q3, methyltransferase	protein_coding	chr6:99369401-	1448	6.553098657	4.95673217	-0.49	6.61E-07	1.93E-06
TEAD4	TEA domain transcription factor 4	protein_coding	chr12:2959330-	2275	14.38035851	11.87316987	-0.36	6.61E-07	1.93E-06
HCCS	holocytochrome c synthase	protein_coding	chrX:11111301-	2343	29.60932088	25.63501528	-0.293	6.67E-07	1.94E-06
RNF150	ring finger protein 150	protein_coding	chr4:140859807	11012	18.55406714	15.79928718	-0.317	6.71E-07	1.95E-06
RBM17	RNA binding motif protein 17	protein_coding	chr10:6089034-	6372	10.48540814	9.164463753	-0.28	6.72E-07	1.96E-06
PLPP3	phospholipid phosphatase 3	protein_coding	chr1:56494761-	5272	1.879586378	1.428053446	-0.483	6.78E-07	1.97E-06
PSME2	proteasome activator subunit 2	protein_coding	chr14:24143362	3582	10.44715842	8.781959604	-0.335	6.78E-07	1.97E-06
NUP88	nucleoporin 88	protein_coding	chr17:5360963-	6003	6.024820524	5.219775849	-0.292	6.90E-07	2.01E-06
CNTFR	ciliary neurotrophic factor receptor	protein_coding	chr9:34551432-	2098	1.589594652	0.879285327	-0.95	7.01E-07	2.03E-06
ICE1	interactor of little elongation complex EL	protein_coding	chr5:5420664-5	8681	7.016953683	5.934892387	-0.326	7.07E-07	2.05E-06
COX15	cytochrome c oxidase assembly homolog	protein_coding	chr10:99711844	4177	4.592307162	3.409723169	-0.514	7.16E-07	2.07E-06
PRXL2B	peroxiredoxin like 2B	protein_coding	chr1:2586491-2	3371	11.88850123	9.610014025	-0.394	7.19E-07	2.08E-06
RFC2	replication factor C subunit 2	protein_coding	chr7:74231499-	2211	6.822611441	5.291229178	-0.449	7.34E-07	2.12E-06
TGIF1	TGFB induced factor homeobox 1	protein_coding	chr18:3411608-	8222	1.403688413	1.052391105	-0.507	7.53E-07	2.17E-06
ZNF22	zinc finger protein 22	protein_coding	chr10:45000923	2103	13.13894868	11.01397934	-0.34	7.53E-07	2.17E-06
ACTC1	actin alpha cardiac muscle 1	protein_coding	chr15:34788095	4602	2542.83793	2066.456516	-0.384	7.56E-07	2.18E-06
MTMR1	myotubularin related protein 1	protein_coding	chrX:150692971	6328	8.46353618	6.970987341	-0.363	7.56E-07	2.18E-06
ATF5	activating transcription factor 5	protein_coding	chr19:49928702	2892	5.212803905	3.757545716	-0.561	7.57E-07	2.18E-06
H19	H19 imprinted maternally expressed trans	lncRNA	chr11:1995176-	2897	418.0504846	315.0031447	-0.498	7.59E-07	2.19E-06
RASSF5	Ras association domain family member 5	protein_coding	chr1:206507530	6976	5.139280652	4.114260178	-0.405	7.61E-07	2.19E-06
TPRA1	transmembrane protein adipocyte associ	protein_coding	chr3:127571266	5622	5.280178754	4.537487179	-0.305	7.72E-07	2.22E-06
PIK3C2B	phosphatidylinositol-4-phosphate 3-kina	protein_coding	chr1:204422628	9414	5.023885893	4.352830346	-0.292	7.82E-07	2.25E-06
RPS6KA4	ribosomal protein S6 kinase A4	protein_coding	chr11:64359148	3807	8.984164777	7.529138177	-0.339	7.86E-07	2.26E-06
ANGPTL2	angiopoietin like 2	protein_coding	chr9:127087348	3625	3.058541499	2.189416102	-0.56	7.95E-07	2.28E-06
MSMO1	methylsterol monooxygenase 1	protein_coding	chr4:165327667	2852	24.27665585	18.90068178	-0.442	7.95E-07	2.28E-06
DOK7	docking protein 7	protein_coding	chr4:3463306-3	4036	10.04831426	8.42878113	-0.339	7.99E-07	2.29E-06
AL162171.4	novel transcript, sense intronic to ZC3H14	lncRNA	chr14:88589231	3178	2.10339913	1.4431769	-0.626	8.10E-07	2.32E-06
HSD17B10	hydroxysteroid 17-beta dehydrogenase 10	protein_coding	chrX:53431258-	1115	51.95307176	43.03300797	-0.357	8.23E-07	2.36E-06
SCFD1	sec1 family domain containing 1	protein_coding	chr14:30622112	6571	7.490140631	6.296866635	-0.334	8.27E-07	2.37E-06
NDUFA5	NADH:ubiquinone oxidoreductase subuni	protein_coding	chr7:123536997	9500	17.85264381	15.05738869	-0.33	8.33E-07	2.39E-06
ZBTB2	zinc finger and BTB domain containing 2	protein_coding	chr6:151364115	3103	4.44933118	3.235870616	-0.537	8.42E-07	2.41E-06
KLHL17	kelch like family member 17	protein_coding	chr1:960584-96	3402	3.338904308	2.607388584	-0.441	8.48E-07	2.42E-06
DENND5B	DENN domain containing 5B	protein_coding	chr12:31382226	11346	19.52814004	16.74225593	-0.307	8.49E-07	2.43E-06
DAGLA	diacylglycerol lipase alpha	protein_coding	chr11:61680391	5799	2.119384081	1.497307943	-0.586	8.50E-07	2.43E-06
SIRT2	sirtuin 2	protein_coding	chr19:38878555	6224	11.6087355	10.2558955	-0.265	8.52E-07	2.43E-06
POP1	POP1 homolog, ribonuclease P/MRP subu	protein_coding	chr8:98117293-	4857	3.084162718	2.282027594	-0.52	8.53E-07	2.44E-06

CDK2	cyclin dependent kinase 2	protein_coding	chr12:55966781	3887	6.392708195	5.20964885	-0.381	8.67E-07	2.47E-06
AC244033.2	novel transcript	lncRNA	chr1:15004566C	3586	1.897911748	1.320582439	-0.611	8.76E-07	2.50E-06
ABLIM1	actin binding LIM protein 1	protein_coding	chr10:11443111	10231	83.31032611	72.49583623	-0.287	8.79E-07	2.50E-06
LRRN4	leucine rich repeat neuronal 4	protein_coding	chr20:6040778-	2692	3.556441833	2.181844203	-0.786	8.89E-07	2.53E-06
SKIDA1	SKI/DACH domain containing 1	protein_coding	chr10:21513478	6700	1.889612448	1.528461965	-0.391	8.93E-07	2.54E-06
PPP1R3F	protein phosphatase 1 regulatory subunit	protein_coding	chrX:49269843-	4333	7.464347579	6.023326818	-0.396	8.93E-07	2.54E-06
SFXN4	sideroflexin 4	protein_coding	chr10:11914076	1868	29.96482381	25.75895261	-0.303	8.97E-07	2.55E-06
SLFN13	schlafen family member 13	protein_coding	chr17:35435096	9329	1.550529463	1.250991243	-0.394	9.00E-07	2.56E-06
BANP	BTG3 associated nuclear protein	protein_coding	chr16:87949244	8154	2.065864349	1.629964236	-0.432	9.08E-07	2.58E-06
TMED9	transmembrane p24 trafficking protein 9	protein_coding	chr5:17759215{8}	2440	29.02172155	25.659502	-0.264	9.10E-07	2.58E-06
SECISBP2L	SECIS binding protein 2 like	protein_coding	chr15:48988476	8583	7.026317812	5.815013832	-0.358	9.11E-07	2.59E-06
PLEKHG4B	pleckstrin homology and RhoGEF domain	protein_coding	chr5:92151-189	13304	1.617124258	1.300591103	-0.398	9.16E-07	2.60E-06
MAPK1IP1L	mitogen-activated protein kinase 1 intera	protein_coding	chr14:55051647	6974	18.19150571	15.53108571	-0.314	9.28E-07	2.63E-06
STN1	STN1 subunit of CST complex	protein_coding	chr10:10387756	6569	5.366392575	4.65116177	-0.293	9.36E-07	2.65E-06
GPAT3	glycerol-3-phosphate acyltransferase 3	protein_coding	chr4:83535914-	3623	22.44629557	18.17648592	-0.387	9.41E-07	2.66E-06
CASQ1	calsequestrin 1	protein_coding	chr1:16019057{5}	2157	9.629303058	7.829198677	-0.385	9.43E-07	2.67E-06
FAM234B	family with sequence similarity 234 memb	protein_coding	chr12:13044381	6710	1.867965838	1.482699521	-0.42	9.50E-07	2.69E-06
ST6GALNAC4	ST6 N-acetylgalactosaminide alpha-2,6-sia	protein_coding	chr9:12790788{6}	2633	24.50903807	21.34562634	-0.285	9.54E-07	2.70E-06
FAM171A2	family with sequence similarity 171 memb	protein_coding	chr17:44353215	3592	6.498332454	5.101271108	-0.434	9.86E-07	2.78E-06
RHPN1	rhophilin Rho GTPase binding protein 1	protein_coding	chr8:14336887{6}	4184	1.923474718	1.319666251	-0.627	1.00E-06	2.82E-06
COG1	component of oligomeric golgi complex 1	protein_coding	chr17:73192632	5317	7.184672243	5.938102938	-0.357	1.00E-06	2.82E-06
FBN2	fibrillin 2	protein_coding	chr5:12825790{9}	13218	142.6169782	112.4911747	-0.424	1.01E-06	2.83E-06
PCNA	proliferating cell nuclear antigen	protein_coding	chr20:5114953-	1403	41.70206446	33.54494302	-0.395	1.01E-06	2.83E-06
VWA8	von Willebrand factor A domain containin	protein_coding	chr13:41566835	7635	15.64849509	13.68725337	-0.278	1.01E-06	2.84E-06
RNF149	ring finger protein 149	protein_coding	chr2:10127121{9}	4836	9.192253509	7.789895563	-0.326	1.01E-06	2.84E-06
MAP3K3	mitogen-activated protein kinase kinase k	protein_coding	chr17:63622415	6107	9.773026691	8.327670826	-0.316	1.02E-06	2.85E-06
SAPCD2	suppressor APC domain containing 2	protein_coding	chr9:137062127	3814	3.189953505	2.20133046	-0.619	1.02E-06	2.85E-06
YAE1	YAE1 maturation factor of ABCE1	protein_coding	chr7:39566385-	2560	4.210410672	3.294825222	-0.44	1.03E-06	2.88E-06
AL109741.1	novel transcript	lncRNA	chr1:10123568{3}	403	12.12423868	8.352900942	-0.627	1.04E-06	2.91E-06
AAK1	AP2 associated kinase 1	protein_coding	chr2:69457997-	24843	5.07376095	4.3966502	-0.292	1.04E-06	2.92E-06
ECEL1	endothelin converting enzyme like 1	protein_coding	chr2:23247982{7}	2978	1.299648098	0.647122759	-1.117	1.05E-06	2.94E-06
CMTM4	CKLF like MARVEL transmembrane domai	protein_coding	chr16:66614750	8117	10.62108141	8.654789223	-0.378	1.06E-06	2.97E-06
C10orf71	chromosome 10 open reading frame 71	protein_coding	chr10:49299170	5439	27.63251411	21.41666966	-0.45	1.07E-06	2.98E-06
ATAD3A	ATPase family AAA domain containing 3A	protein_coding	chr1:1512151-1	3069	12.18276066	9.6248556	-0.432	1.08E-06	3.03E-06
RECK	reversion inducing cysteine rich protein w	protein_coding	chr9:36036913-	5569	2.136908923	1.436155284	-0.649	1.09E-06	3.04E-06
GOLGA3	golgin A3	protein_coding	chr12:13276891	10615	8.717311357	7.079008831	-0.384	1.09E-06	3.05E-06
CCNB2	cyclin B2	protein_coding	chr15:59105126	2753	4.916511003	3.75151371	-0.475	1.09E-06	3.06E-06
SCARF2	scavenger receptor class F member 2	protein_coding	chr22:20424815	3341	10.99928081	8.647976855	-0.435	1.10E-06	3.06E-06
PYROXD1	pyridine nucleotide-disulphide oxidoredu	protein_coding	chr12:21437615	5723	3.328470537	2.691422517	-0.394	1.11E-06	3.09E-06
KATNB1	katanin regulatory subunit B1	protein_coding	chr16:57735739	4576	3.661960813	2.938372394	-0.404	1.11E-06	3.10E-06
CBARP	CACN subunit beta associated regulatory p	protein_coding	chr19:1228287-	4591	2.431389204	1.626533478	-0.666	1.11E-06	3.10E-06
TIMM44	translocase of inner mitochondrial memb	protein_coding	chr19:7926718-	4635	16.24510748	14.29471132	-0.27	1.12E-06	3.12E-06
ZBTB41	zinc finger and BTB domain containing 41	protein_coding	chr1:19715368C	8562	2.41502347	1.918057477	-0.416	1.12E-06	3.12E-06
ABCA3	ATP binding cassette subfamily A member	protein_coding	chr16:2275881-	7804	4.150290558	3.362665859	-0.388	1.12E-06	3.13E-06
GLT8D1	glycosyltransferase 8 domain containing 1	protein_coding	chr3:52694488-	3783	7.784983895	6.660819681	-0.312	1.13E-06	3.15E-06
TRIM23	tripartite motif containing 23	protein_coding	chr5:65589690-	4654	7.098733865	5.867096832	-0.362	1.14E-06	3.17E-06
UBE3B	ubiquitin protein ligase E3B	protein_coding	chr12:10947740	12453	6.227745542	5.213372243	-0.341	1.15E-06	3.19E-06
CSRP3	cysteine and glycine rich protein 3	protein_coding	chr11:19182037	1638	761.8273103	659.2032362	-0.294	1.15E-06	3.21E-06
MTX2	metaxin 2	protein_coding	chr2:17626939{5}	1717	49.74080471	43.44688703	-0.281	1.15E-06	3.21E-06
ARRDC4	arrestin domain containing 4	protein_coding	chr15:97960703	4062	1.513594926	1.08844846	-0.56	1.16E-06	3.22E-06
GINS4	GINS complex subunit 4	protein_coding	chr8:41529218-	5447	1.973143531	1.468963206	-0.508	1.16E-06	3.22E-06
ZFYVE9	zinc finger FYVE-type containing 9	protein_coding	chr1:52142094-	5809	5.850646999	4.994198863	-0.314	1.16E-06	3.22E-06
SHANK1	SH3 and multiple ankyrin repeat domains	protein_coding	chr19:50661827	7567	1.015788445	0.648633135	-0.724	1.16E-06	3.23E-06
UBA5	ubiquitin like modifier activating enzyme	protein_coding	chr3:13265444{6}	6827	7.814190582	6.843799874	-0.277	1.18E-06	3.25E-06
RHEB	Ras homolog, mTORC1 binding	protein_coding	chr7:151466012	3533	26.61131917	22.45159929	-0.33	1.18E-06	3.27E-06
RDH10	retinol dehydrogenase 10	protein_coding	chr8:73294602-	5331	2.837622096	2.007985454	-0.598	1.19E-06	3.30E-06
GDAP1	ganglioside induced differentiation associ	protein_coding	chr8:74321130-	4052	1.770611806	1.225218876	-0.609	1.20E-06	3.32E-06
CHTF18	chromosome transmission fidelity factor	protein_coding	chr16:788046-8	5916	2.6494367	1.85050528	-0.603	1.21E-06	3.33E-06
WRAP53	WD repeat containing antisense to TP53	protein_coding	chr17:7686071-	5087	1.598864299	1.204881952	-0.492	1.22E-06	3.36E-06
IQGAP3	IQ motif containing GTPase activating pro	protein_coding	chr1:15652540{5}	6742	2.57309572	1.727344254	-0.652	1.22E-06	3.36E-06
PTMS	parathymosin	protein_coding	chr12:6765516-	1515	112.7403239	93.18396208	-0.358	1.22E-06	3.37E-06
PCK2	phosphoenolpyruvate carboxykinase 2, m	protein_coding	chr14:24094053	4189	1.212883712	0.778177897	-0.722	1.22E-06	3.37E-06
CLDN12	claudin 12	protein_coding	chr7:90383721-	6708	3.759668443	3.166625094	-0.334	1.23E-06	3.38E-06
KLHL23	kelch like family member 23	protein_coding	chr2:16969448{8}	5513	7.641576748	6.617200831	-0.294	1.23E-06	3.38E-06
MLX	MAX dimerization protein MLX	protein_coding	chr17:42567068	4259	8.64775313	7.415114931	-0.307	1.24E-06	3.42E-06
NDUFS7	NADH:ubiquinone oxidoreductase core su	protein_coding	chr19:1383527-	7456	24.07636285	20.42923869	-0.321	1.26E-06	3.46E-06
TPX2	TPX2 microtubule nucleation factor	protein_coding	chr20:31739271	3605	17.97730829	13.5690954	-0.495	1.26E-06	3.46E-06
AL355488.1	novel transcript, antisense to SLC16A4	lncRNA	chr1:11037015{4}	2850	2.019555283	1.219180151	-0.794	1.27E-06	3.48E-06
OTUD7B	OTU deubiquitinase 7B	protein_coding	chr1:14993781{2}	12227	3.318984851	2.734275382	-0.363	1.27E-06	3.50E-06
DDX39A	DExD-box helicase 39A	protein_coding	chr19:14408819	3840	8.744967685	7.15490188	-0.374	1.30E-06	3.57E-06
MARCKS	myristoylated alanine rich protein kinase	protein_coding	chr6:11385734{5}	4296	1.47525369	0.985693748	-0.664	1.31E-06	3.58E-06
OTUD4	OTU deubiquitinase 4	protein_coding	chr4:14511083{8}	9085	11.14876877	9.602021882	-0.301	1.31E-06	3.59E-06
PROX1	prospero homeobox 1	protein_coding	chr1:213983181	8928	13.84956301	12.12419287	-0.278	1.31E-06	3.59E-06
SS18L1	SS18L1 subunit of BAF chromatin remode	protein_coding	chr20:62143769	5150	8.812391855	7.610915779	-0.294	1.31E-06	3.59E-06
CREG1	cellular repressor of E1A stimulated genes	protein_coding	chr1:16752911{7}	3286	16.36472137	13.97477313	-0.317	1.31E-06	3.60E-06
HNRNPH3	heterogeneous nuclear ribonucleoprotein	protein_coding	chr10:68331174	5373	19.85173436	17.28803834	-0.285	1.32E-06	3.62E-06
KCNK3	potassium two pore domain channel subf	protein_coding	chr2:26692690-	6223	6.185886606	4.667372485	-0.485	1.35E-06	3.70E-06
MYBL2	MYB proto-oncogene like 2	protein_coding	chr20:43667019	2776	8.48983132	6.937987374	-0.377	1.37E-06	3.73E-06
HINFP	histone H4 transcription factor	protein_coding	chr11:11912158	9836	1.843221289	1.530792149	-0.356	1.38E-06	3.76E-06
C21orf91	chromosome 21 open reading frame 91	protein_coding	chr21:17788974	6228	3.092949119	2.468216861	-0.416	1.38E-06	3.77E-06
BBC3	BCL2 binding component 3	protein_coding	chr19:47220822	3180	7.509847696	6.290643727	-0.343	1.39E-06	3.77E-06
MBTD1	mbt domain containing 1	protein_coding	chr17:51177425	7184	5.743553225	5.046679404	-0.274	1.39E-06	3.79E-06
CENPF	centromere protein F	protein_coding	chr1:21460319{5}	11673	6.684770175	5.339835263	-0.412	1.41E-06	3.84E-06
GEMIN2	gem nuclear organelle associated protein	protein_coding	chr14:39114223	2757	3.555754745	2.379645888	-0.656	1.41E-06	3.84E-06
MGST3	microsomal glutathione S-transferase 3	protein_coding	chr1:16563121{3}	8052	31.58360818	26.83640163	-0.32	1.42E-06	3.86E-06
UNC5B-AS1	UNC5B antisense RNA 1	lncRNA	chr10:71217224	707	52.70358806	39.77541834	-0.485	1.45E-06	3.94E-06
RAMP1	receptor activity modifying protein 1	protein_coding	chr2:23785889{3}	1147	13.79809348	10.83764834	-0.435	1.48E-06	4.02E-06
PSMG3	proteasome assembly chaperone 3	protein_coding	chr7:1567330-1	1960	7.12676345	5.309344475	-0.511	1.49E-06	4.02E-06
AC106786.1	novel transcript	lncRNA	chr5:12308724{8}	1020	2.599345197	1.374533923	-0.998	1.51E-06	4.07E-06
MRPL27	mitochondrial ribosomal protein L27	protein_coding	chr17:50367857	3020	14.49185921	12.65277252	-0.283	1.51E-06	4.07E-06
CACNA1B	calcium voltage-gated channel subunit alp	protein_coding	chr9:137877782	9904	2.627641512	1.893529797	-0.553	1.51E-06	4.09E-06
ZNF747	zinc finger protein 747	protein_coding	chr16:30530367	3936	2.125662525	1.542240283	-0.543	1.52E-06	4.10E-06
RAB3IL1	RAB3A interacting protein like 1	protein_coding	chr11:61897301	3191	1.64262969	0.826243273	-1.066	1.53E-06	4.12E-06
DHRSX	dehydrogenase/reductase X-linked	protein_coding	chrX:2219516-2	3620	5.396655315	3.764090718	-0.591	1.53E-06	4.13E-06
SSR4	signal sequence receptor subunit 4	protein_coding	chrX:15379351{6}	4590	12.4021468	10.78200212	-0.287	1.56E-06	4.20E-06
AFMID	arylformamidase	protein_coding	chr17:78187317	4048	6.802348897	5.627601041	-0.357	1.56E-06	4.21E-06
MAMDC4	MAM domain containing 4	protein_coding	chr9:13685094{3}	5490	5.690109802	3.626648593	-0.726	1.56E-06	4.21E-06
IRX5	iroquois homeobox 5	protein_coding	chr16:54930862	2845	12.64664536	10.66219651	-0.335	1.57E-06	4.22E-06
LINGO1	leucine rich repeat and Ig domain contain	protein_coding	chr15:77613027	4902	0.978496529	0.624287874	-0.73	1.59E-06	4.28E-06
CTSA	cathepsin A	protein_coding	chr20:45890144	5856	23.0590907	19.52539844	-0.325	1.59E-06	4.29E-06
YOD1	YOD1 deubiquitinase	protein_coding	chr1:20704384{9}	6521	2.623451604	2.168526973	-0.362	1.60E-06	4.29E-06
MAP4K3-DT	MAP4K3 divergent transcript	lncRNA	chr2:39436530-	12609	1.101993144	0.917815072	-0.351	1.60E-06	4.31E-06
CDADC1	cytidine and dCMP deaminase domain co	protein_coding	chr13:49247925	3863	2.100713044	1.598120699	-0.48	1.61E-06	4.33E-06
SGPP2	sphingosine-1-phosphate phosphatase 2	protein_coding	chr2:22242454{3}	4983	3.586827675	3.002079011	-0.341	1.61E-06	4.33E-06
ZHX3	zinc fingers and homeoboxes 3	protein_coding	chr20:41178448	11621	2.547339921	2.118441594	-0.35	1.62E-06	4.34E-06
MYADML2	myeloid associated differentiation marker	protein_coding	chr17:81939645	2452	5.92405978	4.564672928	-0.458	1.62E-06	4.36E-06
TTK	TTK protein kinase	protein_coding	chr6:80003887-	4579	2.313172082	1.605261182	-0.61	1.62E-06	4.36E-06
WDR12	WD repeat domain 12	protein_coding	chr2:20287478{2}	9098	6.10083661	5.163049339	-0.326	1.63E-06	4.38E-06
LUZP1	leucine zipper protein 1	protein_coding	chr1:23084023-	9658	11.51381978	9.984649714	-0.29	1.68E-06	4.48E-06

PMVK	phosphomevalonate kinase	protein_coding	chr1:15492474(	1002	37.21030913	29.82071901	-0.406	1.68E-06	4.50E-06
TRNP1	TMF1 regulated nuclear protein 1	protein_coding	chr1:26993692-	1979	21.42739188	18.48916385	-0.301	1.69E-06	4.53E-06
NUP214	nucleoporin 214	protein_coding	chr9:13112558(	13213	4.067970186	3.076465107	-0.483	1.70E-06	4.54E-06
ANKRD17	ankyrin repeat domain 17	protein_coding	chr4:73073376-	12042	11.15717016	9.24493567	-0.355	1.70E-06	4.55E-06
ORC4	origin recognition complex subunit 4	protein_coding	chr2:147930397	8915	3.986012097	3.452028539	-0.295	1.72E-06	4.61E-06
AL109955.1	novel transcript	lncRNA	chr20:5737714(	1754	4.272328497	3.253008633	-0.482	1.73E-06	4.62E-06
ITGB3	integrin subunit beta 3	protein_coding	chr17:4725384(	4394	4.529081615	3.076346982	-0.642	1.74E-06	4.64E-06
NAGLU	N-acetyl-alpha-glucosaminidase	protein_coding	chr17:42536241	2935	4.419100057	3.311357025	-0.497	1.75E-06	4.66E-06
MMADHC	metabolism of cobalamin associated D	protein_coding	chr2:14956963?	2150	39.60049883	34.72296955	-0.276	1.75E-06	4.68E-06
EMC7	ER membrane protein complex subunit 7	protein_coding	chr15:34084017	1452	27.03096318	22.0290771	-0.383	1.76E-06	4.70E-06
NUAK1	NUAK family kinase 1	protein_coding	chr12:10606334	7008	13.20866848	11.09279548	-0.336	1.76E-06	4.70E-06
WBP1L	WW domain binding protein 1 like	protein_coding	chr10:10274394	7833	5.250149148	4.565644783	-0.288	1.79E-06	4.75E-06
THAP11	THAP domain containing 11	protein_coding	chr16:67842082	2114	19.63373732	16.66262406	-0.325	1.80E-06	4.78E-06
POC1A	POC1 centriolar protein A	protein_coding	chr3:52075253-	2247	3.359690545	2.432281837	-0.548	1.82E-06	4.83E-06
AL359715.3	novel transcript	lncRNA	chr6:80355424-	1436	2.005640383	1.175848959	-0.857	1.83E-06	4.87E-06
FIGNL2	fidgetin like 2	protein_coding	chr12:5181784(	4973	1.282389782	0.852683958	-0.671	1.84E-06	4.88E-06
RAB33A	RAB33A, member RAS oncogene family	protein_coding	chrX:13017196?	945	2.932504325	1.424806926	-1.133	1.87E-06	4.96E-06
NCAPG	non-SMC condensin I complex subunit G	protein_coding	chr4:17810979-	4847	4.325901522	3.312717032	-0.466	1.88E-06	4.97E-06
LYPLA2	lysophospholipase 2	protein_coding	chr1:23791145-	2021	29.0556742	25.12257656	-0.296	1.92E-06	5.09E-06
FOXM1	forkhead box M1	protein_coding	chr12:2857681-	4899	5.290489092	4.133793741	-0.443	1.93E-06	5.10E-06
KLHL38	kelch like family member 38	protein_coding	chr8:123645527	1982	6.526229084	4.807607246	-0.523	1.97E-06	5.22E-06
AFAP1	actin filament associated protein 1	protein_coding	chr4:7758714-7	10036	6.894404732	5.298922434	-0.461	1.98E-06	5.23E-06
HS1BP3	HCLS1 binding protein 3	protein_coding	chr2:20560448-	14625	1.310126383	1.090821122	-0.347	2.00E-06	5.27E-06
PAK2	p21 (RAC1) activated kinase 2	protein_coding	chr3:196739857	6270	13.78074722	12.14249057	-0.268	2.01E-06	5.29E-06
TUBA4A	tubulin alpha 4a	protein_coding	chr2:21924971(	3966	7.554210244	5.799939687	-0.463	2.02E-06	5.33E-06
TIMELESS	timeless circadian regulator	protein_coding	chr12:56416363	5851	3.952109695	3.207498012	-0.384	2.06E-06	5.43E-06
FAM168A	family with sequence similarity 168 meml	protein_coding	chr11:73400487	7887	17.57118107	15.48722169	-0.268	2.08E-06	5.48E-06
ETV3	ETS variant 3	protein_coding	chr1:157121191	6707	3.702181929	3.212979013	-0.289	2.08E-06	5.48E-06
CHD6	chromodomain helicase DNA binding pro	protein_coding	chr20:41402083	14034	5.393358903	4.5242224	-0.337	2.08E-06	5.49E-06
GPATCH4	G-patch domain containing 4	protein_coding	chr1:156594487	4437	5.281529096	4.396754339	-0.351	2.09E-06	5.51E-06
ZNF30	zinc finger protein 30	protein_coding	chr19:34926903	2745	2.558361601	1.888189838	-0.524	2.10E-06	5.52E-06
MGAT2	mannosyl (alpha-1,6-)-glycoprotein beta-:	protein_coding	chr14:49620795	2687	19.39938024	16.0309159	-0.363	2.11E-06	5.55E-06
USP30	ubiquitin specific peptidase 30	protein_coding	chr12:10902308	5142	3.832900788	3.187198594	-0.351	2.11E-06	5.55E-06
KLRG1	killer cell lectin like receptor G1	protein_coding	chr12:8950044-	2593	1.843641475	1.221964687	-0.678	2.13E-06	5.60E-06
TMEM179B	transmembrane protein 179B	protein_coding	chr11:62787402	1504	10.20351277	8.101779695	-0.419	2.15E-06	5.64E-06
TGS1	trimethylguanosine synthase 1	protein_coding	chr8:55773142-	3869	4.703497728	3.968548168	-0.332	2.16E-06	5.67E-06
TRAF6	TNF receptor associated factor 6	protein_coding	chr11:3648376?	8129	4.01370292	3.489358117	-0.287	2.18E-06	5.72E-06
FEN1	flap structure-specific endonuclease 1	protein_coding	chr11:61792911	2198	10.965658	9.176760257	-0.343	2.20E-06	5.77E-06
SLC12A4	solute carrier family 12 member 4	protein_coding	chr16:67943474	8298	25.68352055	21.61052906	-0.333	2.27E-06	5.94E-06
VDAC1	voltage dependent anion channel 1	protein_coding	chr5:133971871	3049	152.7939344	129.8326335	-0.319	2.27E-06	5.94E-06
NGRN	neugrin, neurite outgrowth associated	protein_coding	chr15:9026565?	7694	4.244611354	3.716933461	-0.278	2.29E-06	5.99E-06
UHRF1	ubiquitin like with PHD and ring finger do	protein_coding	chr19:4903080-	5145	1.983662591	1.458984603	-0.526	2.30E-06	6.02E-06
ZNF844	zinc finger protein 844	protein_coding	chr19:12064731	6588	2.096562066	1.653373938	-0.425	2.32E-06	6.06E-06
ZSWIM4	zinc finger SWIM-type containing 4	protein_coding	chr19:1379546(	4713	1.772580136	1.264482434	-0.572	2.32E-06	6.06E-06
ASH1L	ASH1 like histone lysine methyltransferase	protein_coding	chr1:155335268	13381	8.745248219	7.146583216	-0.374	2.33E-06	6.07E-06
CPSF1	cleavage and polyadenylation specific fact	protein_coding	chr8:14439322?	6058	22.518915	19.86764351	-0.266	2.36E-06	6.14E-06
PAPOLG	poly(A) polymerase gamma	protein_coding	chr2:60756253-	8479	1.940468674	1.640205343	-0.327	2.37E-06	6.17E-06
GPR157	G protein-coupled receptor 157	protein_coding	chr1:9100305-9	5304	2.49719307	2.047744575	-0.371	2.38E-06	6.19E-06
FAM160A1	family with sequence similarity 160 meml	protein_coding	chr4:15140917(	11843	1.767684296	1.445339485	-0.374	2.40E-06	6.25E-06
RBAK	RB associated KRAB zinc finger	protein_coding	chr7:5045821-5	6959	4.919159622	4.258810402	-0.293	2.40E-06	6.25E-06
TECPR2	tectonin beta-propeller repeat containing	protein_coding	chr14:10236294	9402	5.656043352	4.60936613	-0.377	2.42E-06	6.29E-06
PDE7A	phosphodiesterase 7A	protein_coding	chr8:65717510-	7425	4.555093324	3.79629239	-0.344	2.42E-06	6.29E-06
ZNF608	zinc finger protein 608	protein_coding	chr5:12463691?	6922	6.547193525	5.312701111	-0.386	2.42E-06	6.29E-06
LARGE1	LARGE xylosyl- and glucuronyltransferase	protein_coding	chr22:33162226	10687	3.055676177	2.561588817	-0.337	2.44E-06	6.33E-06
SGSM2	small G protein signaling modulator 2	protein_coding	chr17:2337498-	9771	10.86262438	9.292096111	-0.31	2.44E-06	6.34E-06
ZNF616	zinc finger protein 616	protein_coding	chr19:52113091	4907	1.050744346	0.707122117	-0.659	2.45E-06	6.37E-06
CHRNA3	cholinergic receptor nicotinic alpha 3 sub	protein_coding	chr15:78593052	4761	2.231205147	1.597737144	-0.567	2.46E-06	6.39E-06
EXOSC8	exosome component 8	protein_coding	chr13:3699881(	3389	6.562369387	5.089398718	-0.449	2.47E-06	6.40E-06
LRRC75A	leucine rich repeat containing 75A	protein_coding	chr17:16441577	3695	4.003028037	3.006764049	-0.492	2.48E-06	6.42E-06
NSDHL	NAD(P) dependent steroid dehydrogenase	protein_coding	chrX:15283096?	1996	24.97309291	21.08420356	-0.33	2.49E-06	6.43E-06
TNIK	TRAF2 and NCK interacting kinase	protein_coding	chr3:171058414	11448	9.517385254	8.033374884	-0.329	2.51E-06	6.48E-06
ABCG1	ATP binding cassette subfamily G member	protein_coding	chr21:4219968?	6121	0.876844877	0.491910269	-0.899	2.52E-06	6.51E-06
AC009955.2	novel transcript	lncRNA	chr2:21964509(	542	2.399849783	0.704751254	-1.802	2.52E-06	6.52E-06
ATP6V0A1	ATPase H+ transporting V0 subunit a1	protein_coding	chr17:42458844	10955	5.900467857	5.188048328	-0.272	2.53E-06	6.54E-06
ITGA5	integrin subunit alpha 5	protein_coding	chr12:54395261	7383	16.95536622	14.90382281	-0.271	2.54E-06	6.55E-06
LRFN4	leucine rich repeat and fibronectin type II	protein_coding	chr11:66856647	3087	20.96623433	17.85208022	-0.317	2.55E-06	6.57E-06
MOB3C	MOB kinase activator 3C	protein_coding	chr1:46607715-	5916	1.117147099	0.740986297	-0.686	2.55E-06	6.59E-06
TIMM23	translocase of inner mitochondrial memb	protein_coding	chr10:4597248?	1190	62.54615928	53.98789059	-0.3	2.57E-06	6.62E-06
POR	cytochrome p450 oxidoreductase	protein_coding	chr7:75899200-	6042	7.232338449	6.297337739	-0.286	2.60E-06	6.71E-06
SNX2	sorting nexin 2	protein_coding	chr5:12277507?	8606	10.17719571	8.880849281	-0.282	2.62E-06	6.74E-06
CNOT1	CCR4-NOT transcription complex subunit	protein_coding	chr16:58519951	10851	14.04898481	12.00117306	-0.312	2.64E-06	6.79E-06
SHARPIN	SHANK associated RH domain interactor	protein_coding	chr8:14409863?	2783	16.34409602	14.44659646	-0.265	2.64E-06	6.79E-06
WDR47	WD repeat domain 47	protein_coding	chr1:10897021?	4423	5.661276522	4.735602648	-0.342	2.65E-06	6.82E-06
TRH	thyrotropin releasing hormone	protein_coding	chr3:12997468?	1592	14.61892509	9.927568044	-0.653	2.66E-06	6.84E-06
MOCS1	molybdenum cofactor synthesis 1	protein_coding	chr6:39899578-	5174	4.465746886	3.765231072	-0.332	2.71E-06	6.96E-06
ABL2	ABL proto-oncogene 2, non-receptor tyro	protein_coding	chr1:17909933(	16689	2.241751482	1.904573386	-0.321	2.72E-06	6.99E-06
LAMC1	laminin subunit gamma 1	protein_coding	chr1:18302342(	8446	83.38151509	71.6188835	-0.304	2.74E-06	7.01E-06
REEP4	receptor accessory protein 4	protein_coding	chr8:22138020-	2266	7.527713169	6.109037543	-0.385	2.78E-06	7.13E-06
AC026691.1	novel transcript, antisense to H2AFY	lncRNA	chr5:13539928(	2017	1.372016363	0.742310554	-0.968	2.79E-06	7.13E-06
HIST1H3E	histone cluster 1 H3 family member e	protein_coding	chr6:26224199-	2679	5.302269312	4.02200593	-0.49	2.80E-06	7.18E-06
MKKS	McKusick-Kaufman syndrome	protein_coding	chr20:10401009 7040		2.476568872	2.046592975	-0.361	2.83E-06	7.23E-06
MCM2	minichromosome maintenance complex	protein_coding	chr3:12759841(	4715	9.283966098	7.904623024	-0.317	2.83E-06	7.25E-06
TMEM80	transmembrane protein 80	protein_coding	chr11:695428-7	4071	5.453614148	4.55747815	-0.347	2.85E-06	7.29E-06
SLC25A5	solute carrier family 25 member 5	protein_coding	chrX:119468422	1555	317.781389	278.9328339	-0.273	2.85E-06	7.29E-06
WIPI1	WD repeat domain, phosphoinositide inte	protein_coding	chr17:68420948	3403	26.61868491	23.35312251	-0.276	2.87E-06	7.32E-06
ECHDC1	ethylmalonyl-CoA decarboxylase 1	protein_coding	chr6:127288712	5676	10.5901554	9.234013357	-0.285	2.89E-06	7.37E-06
ESYT1	extended synaptotagmin 1	protein_coding	chr12:5611825(	5493	8.874083257	7.714219503	-0.287	2.90E-06	7.41E-06
ZFP36	ZFP36 ring finger protein	protein_coding	chr19:39406813 2010		6.167937387	4.54603143	-0.526	2.92E-06	7.44E-06
LINC01034	long intergenic non-protein coding RNA 1	lncRNA	chr13:76013023	277	1.141998703	0.232991634	-2.202	2.93E-06	7.47E-06
CNST	consortin, connexin sorting protein	protein_coding	chr1:246566444	5933	4.052230705	3.471621836	-0.307	2.94E-06	7.50E-06
KCTD6	potassium channel tetramerization doma	protein_coding	chr3:58492096-	3086	2.40471935	1.846856758	-0.462	2.94E-06	7.50E-06
LIN52	lin-52 DREAM MuvB core complex compo	protein_coding	chr14:7408479(	3692	4.260788837	3.660585497	-0.307	2.96E-06	7.55E-06
PAQR6	progestin and adipoQ receptor family me	protein_coding	chr1:15624332(	2531	15.33860552	11.74229696	-0.469	2.98E-06	7.59E-06
ADAMTS8	ADAM metallopeptidase with thrombosp	protein_coding	chr11:13040492	4141	1.848537844	1.282952712	-0.605	3.04E-06	7.74E-06
GGH	gamma-glutamyl hydrolase	protein_coding	chr8:63015079-	2765	2.569801096	1.61865199	-0.768	3.05E-06	7.75E-06
TSPAN12	tetraspanin 12	protein_coding	chr7:12078732(	3368	14.54186358	11.5628146	-0.411	3.05E-06	7.76E-06
SMAD4	SMAD family member 4	protein_coding	chr18:51028394	13123	3.662942482	3.170376215	-0.293	3.10E-06	7.87E-06
NPC1	NPC intracellular cholesterol transporter	protein_coding	chr18:23506184	9705	2.783942548	2.273307899	-0.376	3.13E-06	7.94E-06
HOMEZ	homeobox and leucine zipper encoding	protein_coding	chr14:23272422	5534	3.871784476	3.26829528	-0.328	3.14E-06	7.96E-06
ACTR10	actin related protein 10	protein_coding	chr14:5820008(	4039	24.57093516	21.48306443	-0.278	3.22E-06	8.13E-06
NPAS1	neuronal PAS domain protein 1	protein_coding	chr19:4701982(	2582	1.127700783	0.676868123	-0.827	3.23E-06	8.15E-06
XRCC2	X-ray repair cross complementing 2	protein_coding	chr7:15264477(	4832	1.161418291	0.807308267	-0.602	3.26E-06	8.22E-06
ZCCHC4	zinc finger CCHC-type containing 4	protein_coding	chr4:25312774-	4411	2.646803402	2.158127908	-0.378	3.26E-06	8.22E-06
TMEM98	transmembrane protein 98	protein_coding	chr17:3292791(	5187	5.951679984	5.114699428	-0.305	3.26E-06	8.23E-06
TRAPPC6A	trafficking protein particle complex 6A	protein_coding	chr19:4516292?	933	27.82696988	23.58444157	-0.328	3.31E-06	8.35E-06
IWS1	interacts with SUPT6H, CTD assembly fact	protein_coding	chr2:127436207	4146	14.46999196	12.78282208	-0.264	3.32E-06	8.36E-06
KIAA0586	KIAA0586	protein_coding	chr14:58427385	15813	1.534491089	1.263672042	-0.361	3.33E-06	8.38E-06
HLA-F-AS1	HLA-F antisense RNA 1	lncRNA	chr6:29726601-	4179	0.913305755	0.54620898	-0.808	3.38E-06	8.50E-06
PRRT4	proline rich transmembrane protein 4	protein_coding	chr7:128350325	3683	2.541768934	1.741973251	-0.627	3.45E-06	8.65E-06

CSPP1	centrosome and spindle pole associated p	protein_coding	chr8:67062426-	7096	2.147489376	1.63262414	-0.48	3.45E-06	8.66E-06
USP40	ubiquitin specific peptidase 40	protein_coding	chr2:23347552(	9352	2.833145201	2.321129751	-0.371	3.45E-06	8.66E-06
NCAPH	non-SMC condensin I complex subunit H	protein_coding	chr2:96335766-	6545	1.351208778	0.909325983	-0.649	3.50E-06	8.77E-06
INSIG1	insulin induced gene 1	protein_coding	chr7:15529777(	3650	7.394848837	5.692805334	-0.456	3.53E-06	8.84E-06
CA2	carbonic anhydrase 2	protein_coding	chr8:85463968-	2007	1.240358289	0.625953173	-1.059	3.54E-06	8.86E-06
PNPLA8	patatin like phospholipase domain contai	protein_coding	chr7:108470417	5673	9.72415919	8.544188771	-0.274	3.56E-06	8.91E-06
DENND4A	DENN domain containing 4A	protein_coding	chr15:65658046	10623	2.465313562	2.056892273	-0.346	3.57E-06	8.93E-06
FBXL20	F-box and leucine rich repeat protein 20	protein_coding	chr17:39252663	11053	1.921829452	1.610075461	-0.343	3.58E-06	8.95E-06
THEM6	thioesterase superfamily member 6	protein_coding	chr8:142727223	3035	7.800049414	6.45012657	-0.36	3.60E-06	8.99E-06
TAGLN2	transgelin 2	protein_coding	chr1:159918107	2487	3.05468378	2.124944341	-0.599	3.62E-06	9.04E-06
PLXND1	plexin D1	protein_coding	chr3:129555175	9738	1.050144181	0.807993486	-0.465	3.64E-06	9.09E-06
PIGA	phosphatidylinositol glycan anchor biosy	protein_coding	chrX:15319451-	6321	1.23106453	0.924246462	-0.502	3.68E-06	9.18E-06
PKD1	polycystin 1, transient receptor potential	protein_coding	chr16:2088710-	18532	14.37985828	10.76907051	-0.496	3.73E-06	9.28E-06
MCM10	minichromosome maintenance 10 replica	protein_coding	chr10:13161554	5454	1.156699757	0.849946465	-0.524	3.74E-06	9.31E-06
CAVIN4	caveolae associated protein 4	protein_coding	chr9:100578079	2615	20.14575129	16.975806	-0.337	3.75E-06	9.33E-06
CDCA7	cell division cycle associated 7	protein_coding	chr2:17335482(	3934	1.763808629	1.276547646	-0.547	3.75E-06	9.33E-06
ZSWIM8	zinc finger SWIM-type containing 8	protein_coding	chr10:73785582	7953	10.9841526	8.923724561	-0.381	3.81E-06	9.48E-06
CKMT2	creatine kinase, mitochondrial 2	protein_coding	chr5:81233320-	2702	82.91529388	71.44442711	-0.301	3.82E-06	9.49E-06
TPRN	taperin	protein_coding	chr9:137191617	5281	5.927734602	4.995413015	-0.335	3.83E-06	9.50E-06
CDKN2AIP	CDKN2A interacting protein	protein_coding	chr4:183444635	4015	4.950611587	4.205876711	-0.319	3.83E-06	9.51E-06
TET3	tet methylcytosine dioxygenase 3	protein_coding	chr2:73986404-	12271	9.138766367	7.253028301	-0.414	3.83E-06	9.52E-06
POLR3E	RNA polymerase III subunit E	protein_coding	chr16:22297375	9486	4.935938401	4.356579192	-0.267	3.93E-06	9.75E-06
ARHGAP11A	Rho GTPase activating protein 11A	protein_coding	chr15:32615144	6406	4.024427097	3.163306687	-0.431	3.94E-06	9.75E-06
PALLD	palladin, cytoskeletal associated protein	protein_coding	chr4:168497052	10128	99.75611838	85.70376244	-0.303	3.94E-06	9.75E-06
NMB	neuromedin B	protein_coding	chr15:84655129	1022	3.209327588	1.555720261	-1.147	3.98E-06	9.86E-06
NEXN	nexilin F-actin binding protein	protein_coding	chr1:77888513-	4436	94.4886948	81.08523392	-0.304	4.00E-06	9.91E-06
NAPEPLD	N-acyl phosphatidylethanolamine phosph	protein_coding	chr7:10309977(	7561	2.713433116	2.295594451	-0.328	4.01E-06	9.92E-06
HDAC6	histone deacetylase 6	protein_coding	chrX:48801377-	10602	6.39980998	5.339690928	-0.346	4.06E-06	1.00E-05
PORCN	porcupine O-acyltransferase	protein_coding	chrX:48508962-	3011	5.095215697	4.160799652	-0.381	4.07E-06	1.01E-05
KLF10	Kruppel like factor 10	protein_coding	chr8:102648784	3821	3.919274619	3.066374974	-0.44	4.10E-06	1.01E-05
MED1	mediator complex subunit 1	protein_coding	chr17:39404285	8608	6.778061098	5.767160579	-0.317	4.10E-06	1.01E-05
MEX3B	mex-3 RNA binding family member B	protein_coding	chr15:82041778	4342	2.441308518	1.807074023	-0.521	4.18E-06	1.03E-05
REV3L	REV3 like, DNA directed polymerase zeta c	protein_coding	chr6:111299028	12425	10.01678134	8.61408621	-0.302	4.22E-06	1.04E-05
KIAA0895L	KIAA0895 like	protein_coding	chr16:67175599	4783	8.929591281	7.196595415	-0.394	4.22E-06	1.04E-05
ATL2	atlastin GTPase 2	protein_coding	chr2:38293954-	5810	6.031592985	5.288221248	-0.274	4.23E-06	1.04E-05
DERA	deoxyribose-phosphate aldolase	protein_coding	chr12:15911302	2813	7.378683484	6.269237288	-0.324	4.24E-06	1.05E-05
ARMH4	armadillo like helical domain containing 4	protein_coding	chr14:57999735	7565	2.829384915	2.322978847	-0.372	4.30E-06	1.06E-05
KIF1B	kinesin family member 1B	protein_coding	chr1:10210805-	17720	12.58435748	11.01257713	-0.277	4.35E-06	1.07E-05
ADAM11	ADAM metallopeptidase domain 11	protein_coding	chr17:44758988	4813	58.20894038	49.552046	-0.317	4.41E-06	1.09E-05
LNP1	leukemia NUP98 fusion partner 1	protein_coding	chr3:100401532	1910	4.845511146	3.885362604	-0.407	4.46E-06	1.10E-05
YIPF4	Yip1 domain family member 4	protein_coding	chr2:32277904-	12239	9.021568533	7.514600672	-0.352	4.49E-06	1.10E-05
EIF2B2	eukaryotic translation initiation factor 2B	protein_coding	chr14:75002921	5436	4.437523392	3.730330708	-0.334	4.51E-06	1.11E-05
TRPT1	tRNA phosphotransferase 1	protein_coding	chr11:64223799	2210	19.79144875	16.96277155	-0.306	4.55E-06	1.12E-05
THSD4	thrombospondin type 1 domain containi	protein_coding	chr15:71096952	15837	5.925353335	4.948422322	-0.344	4.57E-06	1.12E-05
MED13	mediator complex subunit 13	protein_coding	chr17:61942605	12673	5.304786959	4.57905967	-0.296	4.59E-06	1.13E-05
UQCRHL	ubiquinol-cytochrome c reductase hinge	protein_coding	chr1:15807169-	2180	6.681799498	5.115174008	-0.472	4.64E-06	1.13E-05
MIIP	migration and invasion inhibitory protein	protein_coding	chr1:12019466-	2821	4.146292923	3.330768309	-0.403	4.68E-06	1.14E-05
ARRDC1-AS1	ARRDC1 antisense RNA 1	lncRNA	chr9:137615332	3545	4.892367782	4.061702074	-0.354	4.69E-06	1.15E-05
PLA2G4C	phospholipase A2 group IVC	protein_coding	chr19:48047843	6613	1.128987939	0.719625398	-0.724	4.71E-06	1.15E-05
RBM15	RNA binding motif protein 15	protein_coding	chr1:110338506	4670	3.231953807	2.478866811	-0.472	4.71E-06	1.15E-05
ATP13A1	ATPase 13A1	protein_coding	chr19:19645198	8359	5.272938373	4.564746912	-0.293	4.74E-06	1.16E-05
CCDC183-AS1	CCDC183 antisense RNA 1	lncRNA	chr9:136803927	2386	1.615444918	1.014281808	-0.762	4.88E-06	1.19E-05
NGLY1	N-glycanase 1	protein_coding	chr3:25718944-	8273	5.169736074	4.354554811	-0.331	4.91E-06	1.20E-05
USPL1	ubiquitin specific peptidase like 1	protein_coding	chr13:30617693	5125	4.024961795	3.503228766	-0.288	4.92E-06	1.20E-05
PARP3	poly(ADP-ribose) polymerase family meml	protein_coding	chr3:51942345-	2928	2.419196422	1.588019944	-0.68	4.94E-06	1.20E-05
ZBTB11-AS1	ZBTB11 antisense RNA 1	lncRNA	chr3:101676475	2743	2.727009269	2.088628232	-0.473	4.97E-06	1.21E-05
LINC01128	long intergenic non-protein coding RNA 1	lncRNA	chr1:825138-86	18974	5.727907276	4.9923847	-0.282	4.98E-06	1.21E-05
ATP7B	ATPase copper transporting beta	protein_coding	chr13:51930436	9671	3.709518148	2.924156971	-0.426	5.00E-06	1.21E-05
PIMREG	PICALM interacting mitotic regulator	protein_coding	chr17:6444441-	2797	3.351272579	2.628247545	-0.435	5.05E-06	1.23E-05
AFF4	AF4/FMR2 family member 4	protein_coding	chr5:132875395	11281	16.10803082	13.97186811	-0.289	5.11E-06	1.24E-05
TOB1-AS1	TOB1 antisense RNA 1	lncRNA	chr17:50866679	3539	0.992575558	0.589588581	-0.848	5.17E-06	1.25E-05
AC074143.1	nucleolar protein 3	protein_coding	chr16:67170154	2992	7.453565342	6.224598977	-0.345	5.19E-06	1.26E-05
PTN	pleiotrophin	protein_coding	chr7:137227341	1713	10.53937681	8.391706789	-0.409	5.22E-06	1.27E-05
AC023509.3	novel transcript, antisense to TARBP2	lncRNA	chr12:53500162	775	3.380660853	2.092197484	-0.764	5.24E-06	1.27E-05
ULK2	unc-51 like autophagy activating kinase 2	protein_coding	chr17:19770829	9868	3.251942631	2.785726783	-0.309	5.27E-06	1.27E-05
CDC25B	cell division cycle 25B	protein_coding	chr20:3786772-	4314	8.09510871	6.739966792	-0.351	5.31E-06	1.28E-05
KDSR	3-ketodihydrosphingosine reductase	protein_coding	chr18:63327726	9031	2.974471835	2.624395299	-0.265	5.35E-06	1.29E-05
AL008721.2	novel transcript	lncRNA	chr22:25476218	3754	1.187557274	0.859439315	-0.548	5.43E-06	1.31E-05
MYNN	myoneurin	protein_coding	chr3:169772831	5730	3.929191027	3.392723669	-0.297	5.47E-06	1.32E-05
LRRC45	leucine rich repeat containing 45	protein_coding	chr17:82023305	3603	4.120075303	3.385561129	-0.37	5.51E-06	1.33E-05
VAT1L	vesicle amine transport 1 like	protein_coding	chr16:77788564	4103	4.435248057	3.825533642	-0.299	5.54E-06	1.33E-05
RWDD2B	RWD domain containing 2B	protein_coding	chr21:29004384	3377	9.536382497	8.059749274	-0.326	5.56E-06	1.34E-05
BBS4	Bardet-Biedl syndrome 4	protein_coding	chr15:72686179	4148	4.540182875	3.614088399	-0.42	5.62E-06	1.35E-05
ZNF597	zinc finger protein 597	protein_coding	chr16:3432414-	5483	1.118724048	0.849538156	-0.483	5.63E-06	1.35E-05
IFNLR1	interferon lambda receptor 1	protein_coding	chr1:24154168-	4940	0.966957361	0.603182529	-0.771	5.65E-06	1.36E-05
BRIP1	BRCA1 interacting protein C-terminal heli	protein_coding	chr17:61681266	7991	0.995897897	0.739849653	-0.507	5.68E-06	1.36E-05
PLEKHA4	pleckstrin homology domain containing A	protein_coding	chr19:48837097	3836	10.08051077	8.582354373	-0.317	5.76E-06	1.38E-05
MYLK3	myosin light chain kinase 3	protein_coding	chr16:46702282	10644	70.17810927	57.21377729	-0.376	5.78E-06	1.39E-05
BAG1	BCL2 associated athanogene 1	protein_coding	chr9:33247820-	7073	17.64959003	14.90481147	-0.328	5.81E-06	1.39E-05
CRADD	CASP2 and RIPK1 domain containing adap	protein_coding	chr12:93677375	4276	5.389766893	4.699875632	-0.285	5.83E-06	1.40E-05
CNPY3	canopy FGF signaling regulator 3	protein_coding	chr6:42929192-	1712	10.60512868	8.572692115	-0.397	6.08E-06	1.46E-05
RPS6KL1	ribosomal protein S6 kinase like 1	protein_coding	chr14:74903954	7617	1.542080383	1.118143277	-0.545	6.10E-06	1.46E-05
SFXN3	sideroflexin 3	protein_coding	chr10:10103123	4631	1.222786649	0.770393409	-0.746	6.10E-06	1.46E-05
RTL10	retrotransposon Gag like 10	protein_coding	chr22:19846138	6803	3.240807889	2.753282618	-0.318	6.11E-06	1.46E-05
C9orf116	chromosome 9 open reading frame 116	protein_coding	chr9:135495181	1864	2.218914199	1.462631234	-0.683	6.14E-06	1.47E-05
WDR35	WD repeat domain 35	protein_coding	chr2:19910260-	7836	3.141134106	2.568092514	-0.373	6.16E-06	1.47E-05
SLIRP	SRA stem-loop interacting RNA binding pr	protein_coding	chr14:77708071	2631	11.84166287	9.525448958	-0.398	6.19E-06	1.48E-05
FGF2	fibroblast growth factor 2	protein_coding	chr4:122826708	6775	0.923920179	0.601069066	-0.72	6.21E-06	1.48E-05
RPP14	ribonuclease P/MRP subunit p14	protein_coding	chr3:58306247-	8374	4.033324045	3.502267018	-0.288	6.22E-06	1.48E-05
SCAMP5	secretory carrier membrane protein 5	protein_coding	chr15:74957219	4862	5.836185545	5.036913468	-0.303	6.22E-06	1.49E-05
LINC00662	long intergenic non-protein coding RNA 6	lncRNA	chr19:27681072	33198	0.971463747	0.851312506	-0.276	6.29E-06	1.50E-05
INCA1	inhibitor of CDK, cyclin A1 interacting pro	protein_coding	chr17:4988130-	1553	1.766347501	1.004820521	-0.881	6.31E-06	1.50E-05
FRMPD1	FERM and PDZ domain containing 1	protein_coding	chr9:37650954-	5913	1.66406028	1.248117367	-0.494	6.41E-06	1.53E-05
AL391422.4	novel transcript, antisense to PXDC1	lncRNA	chr6:3751111-3	2761	4.843688614	3.701372098	-0.474	6.53E-06	1.55E-05
RIC8B	RIC8 guanine nucleotide exchange factor	protein_coding	chr12:10677462	7769	3.724864718	3.280470737	-0.27	6.56E-06	1.56E-05
FBXL6	F-box and leucine rich repeat protein 6	protein_coding	chr8:144355431	3375	3.441320701	2.714120221	-0.427	6.57E-06	1.56E-05
MSI1	musashi RNA binding protein 1	protein_coding	chr12:12034133	3074	8.195675563	7.028051937	-0.309	6.60E-06	1.57E-05
SNHG15	small nucleolar RNA host gene 15	lncRNA	chr7:44983023-	3939	1.64625235	1.185622056	-0.556	6.62E-06	1.57E-05
GABRE	gamma-aminobutyric acid type A recepto	protein_coding	chrX:151953124	9612	27.44952411	23.55193229	-0.305	6.64E-06	1.58E-05
CLASP1	cytoplasmic linker associated protein 1	protein_coding	chr2:121337776	11052	10.71716262	9.184573895	-0.307	6.66E-06	1.58E-05
LINC02517	long intergenic non-protein coding RNA 2	lncRNA	chr4:8320105-8	1970	1.923404252	1.150931451	-0.844	6.66E-06	1.58E-05
ARSK	arylsulfatase family member K	protein_coding	chr5:95555074-	4314	1.627469045	1.174853111	-0.565	6.66E-06	1.58E-05
ATL3	atlastin GTPase 3	protein_coding	chr11:63624087	7600	1.563719869	1.163907875	-0.513	6.70E-06	1.59E-05
SPTSSA	serine palmitoyltransferase small subunit	protein_coding	chr14:34432788	2662	12.80145018	10.68402513	-0.346	6.81E-06	1.61E-05
KIAA1217	KIAA1217	protein_coding	chr10:23669746	10807	2.937014695	2.368131958	-0.393	7.02E-06	1.66E-05
CHD8	chromodomain helicase DNA binding pro	protein_coding	chr14:21385194	20953	4.058420708	3.534798977	-0.283	7.06E-06	1.67E-05
SPC25	SPC25 component of NDC80 kinetochore	protein_coding	chr2:168834132	1829	3.361794341	2.404622757	-0.561	7.15E-06	1.69E-05
DNAJB4	DnaJ heat shock protein family (Hsp40) m	protein_coding	chr1:77979175-	3702	11.61266924	10.03978405	-0.297	7.19E-06	1.70E-05
MRPS18A	mitochondrial ribosomal protein S18A	protein_coding	chr6:43671303-	1284	19.50967995	15.78107165	-0.392	7.19E-06	1.70E-05

PPP1R7	protein phosphatase 1 regulatory subunit	protein_coding	chr2:24114957€	3781	10.71463937	9.161104553	-0.31	7.21E-06	1.70E-05
FJX1	four-jointed box kinase 1	protein_coding	chr11:3561841ℓ	2450	2.914058293	2.226337874	-0.478	7.29E-06	1.72E-05
ATPAF2	ATP synthase mitochondrial F1 complex a	protein_coding	chr17:1797740ℓ	5553	3.624668886	3.131555193	-0.3	7.30E-06	1.72E-05
ARL15	ADP ribosylation factor like GTPase 15	protein_coding	chr5:53883942-	3800	5.016155255	4.357348242	-0.29	7.36E-06	1.73E-05
C11orf96	chromosome 11 open reading frame 96	protein_coding	chr11:43925342	1918	1.191439789	0.624840996	-1.038	7.37E-06	1.74E-05
AC016355.1	novel transcript	lncRNA	chr15:67541072	1533	1.749471834	0.982809624	-0.915	7.45E-06	1.75E-05
PCOLCE	procollagen C-endopeptidase enhancer	protein_coding	chr7:10060236З	3346	1.325526475	0.887787625	-0.656	7.47E-06	1.76E-05
PCDHA12	protocadherin alpha 12	protein_coding	chr5:140875302	5457	2.64461194	2.051548206	-0.446	7.48E-06	1.76E-05
UHRF2	ubiquitin like with PHD and ring finger do	protein_coding	chr9:6413151-6	9919	3.257647781	2.8628608	-0.273	7.53E-06	1.77E-05
CREB5	cAMP responsive element binding protein	protein_coding	chr7:28299321-	11681	2.828361175	2.108778993	-0.506	7.54E-06	1.77E-05
ZC2HC1C	zinc finger C2HC-type containing 1C	protein_coding	chr14:7506417C	4516	1.618012735	1.193479698	-0.517	7.67E-06	1.80E-05
ERICH5	glutamate rich 5	protein_coding	chr8:98064522-	1550	3.344808471	2.090076308	-0.79	7.68E-06	1.80E-05
PNPLA7	patatin like phospholipase domain contai	protein_coding	chr9:137459952	9845	1.068201408	0.74989608	-0.583	7.70E-06	1.81E-05
PSMG1	proteasome assembly chaperone 1	protein_coding	chr21:39174769	2515	9.666015459	8.248201321	-0.314	7.69E-06	1.81E-05
CHEK2	checkpoint kinase 2	protein_coding	chr22:28687743	4493	2.286899709	1.816831373	-0.414	7.74E-06	1.81E-05
DNAAF2	dynein axonemal assembly factor 2	protein_coding	chr14:49625174	2963	6.586976808	5.802749422	-0.27	7.81E-06	1.83E-05
SYTL3	synaptotagmin like 3	protein_coding	chr6:158650014	2896	5.296826617	4.317222357	-0.381	7.87E-06	1.84E-05
PDCD4-AS1	PDCD4 antisense RNA 1	lncRNA	chr10:1108698€	778	1.483434347	0.6645407	-1.23	7.97E-06	1.86E-05
AC027601.4	novel transcript, antisense to SLC38A10	lncRNA	chr17:81251194	610	4.037532698	2.33985684	-0.865	8.12E-06	1.90E-05
UTP6	UTP6 small subunit processome compone	protein_coding	chr17:31860904	6388	5.241944836	4.573126655	-0.28	8.15E-06	1.90E-05
NAV2-AS2	NAV2 antisense RNA 2	lncRNA	chr11:2004384€	782	45.46050173	38.89504188	-0.312	8.19E-06	1.91E-05
MYL12A	myosin light chain 12A	protein_coding	chr18:3247481-	3245	78.15649407	68.32863429	-0.28	8.25E-06	1.92E-05
AF111167.2	novel transcript, antisense to JDP2	lncRNA	chr14:75423683	4059	1.049766121	0.722257456	-0.616	8.31E-06	1.93E-05
MBD6	methyl-CpG binding domain protein 6	protein_coding	chr12:5752071C	5566	11.96607239	10.22195336	-0.311	8.36E-06	1.95E-05
HAUS1	HAUS augmin like complex subunit 1	protein_coding	chr18:46104378	2119	5.536269012	4.355141075	-0.434	8.41E-06	1.96E-05
LRCH4	leucine rich repeats and calponin homolo	protein_coding	chr7:100574011	7117	1.818821136	1.548047328	-0.319	8.43E-06	1.96E-05
PEX6	peroxisomal biogenesis factor 6	protein_coding	chr6:42963865-	3465	9.097909228	7.981804932	-0.274	8.51E-06	1.97E-05
FXN	frataxin	protein_coding	chr9:69035563-	7859	1.535175997	1.239162007	-0.397	8.52E-06	1.98E-05
ZCCHC3	zinc finger CCHC-type containing 3	protein_coding	chr20:296968-3	3354	11.56596254	9.774230848	-0.331	8.53E-06	1.98E-05
TTI1	TELO2 interacting protein 1	protein_coding	chr20:37983007	4472	5.739484281	5.042769916	-0.274	8.69E-06	2.01E-05
MRGBP	MRG domain binding protein	protein_coding	chr20:62796473	2752	5.88836729	4.807328105	-0.381	8.76E-06	2.02E-05
ARID2	AT-rich interaction domain 2	protein_coding	chr12:4572970€	12086	4.129915773	3.524675893	-0.314	8.83E-06	2.04E-05
E2F6	E2F transcription factor 6	protein_coding	chr2:11444375-	4067	5.426448423	4.689227153	-0.295	8.83E-06	2.04E-05
DNASE1L1	deoxyribonuclease 1 like 1	protein_coding	chrX:15440123€	4132	8.438860512	7.418989325	-0.269	8.92E-06	2.06E-05
SMIM10L1	small integral membrane protein 10 like 1	protein_coding	chr12:11171222	4795	13.5345294	11.866902	-0.275	8.98E-06	2.07E-05
TLNRD1	talin rod domain containing 1	protein_coding	chr15:81000944	4845	9.85424485	8.033831863	-0.376	9.16E-06	2.11E-05
HIST1H2AG	histone cluster 1 H2A family member g	protein_coding	chr6:27133042-	2250	0.995401368	0.577150366	-0.866	9.29E-06	2.14E-05
PARP2	poly(ADP-ribose) polymerase 2	protein_coding	chr14:20343582	4096	8.053982132	6.934639676	-0.3	9.31E-06	2.15E-05
ANK2	ankyrin 2	protein_coding	chr4:11281810ℓ	18224	17.54432429	15.3278725	-0.28	9.48E-06	2.18E-05
COL4A6	collagen type IV alpha 6 chain	protein_coding	chrX:108155607	9282	20.0127335	16.80090061	-0.337	9.48E-06	2.18E-05
GPN1	GPN-loop GTPase 1	protein_coding	chr2:27628247-	3746	3.963550103	3.303298714	-0.347	9.56E-06	2.20E-05
LRRC39	leucine rich repeat containing 39	protein_coding	chr1:10014844ℓ	1991	50.64481837	42.52802587	-0.334	9.80E-06	2.25E-05
TMEM199	transmembrane protein 199	protein_coding	chr17:28357642	4559	2.546870749	2.086887215	-0.375	9.98E-06	2.29E-05
COL5A2	collagen type V alpha 2 chain	protein_coding	chr2:18903189€	7319	44.04162851	37.22177228	-0.327	1.01E-05	2.32E-05
DUSP8	dual specificity phosphatase 8	protein_coding	chr11:1554051-	4960	9.573732721	8.218674406	-0.304	1.01E-05	2.32E-05
INPP5K	inositol polyphosphate-5-phosphatase K	protein_coding	chr17:1494577-	5173	3.201390519	2.786568113	-0.288	1.04E-05	2.37E-05
PYROXD2	pyridine nucleotide-disulphide oxidoredu	protein_coding	chr10:98383565	3865	17.96954324	15.51662795	-0.295	1.04E-05	2.38E-05
AGO1	argonaute RISC component 1	protein_coding	chr1:35869808-	13890	2.648263065	2.263264018	-0.311	1.04E-05	2.38E-05
IGF2	insulin like growth factor 2	protein_coding	chr11:2129112-	6259	19.7506706	13.71704213	-0.603	1.07E-05	2.44E-05
ADCK5	aarF domain containing kinase 5	protein_coding	chr8:144373101	2742	6.814853812	5.696146019	-0.346	1.08E-05	2.46E-05
AP001160.3	novel transcript, antisense to TAF6L	lncRNA	chr11:62786023	763	6.83405245	4.764946075	-0.607	1.09E-05	2.49E-05
RSRC2	arginine and serine rich coiled-coil 2	protein_coding	chr12:12250345	8745	6.454845545	5.64452678	-0.277	1.09E-05	2.49E-05
TMED1	transmembrane p24 trafficking protein 1	protein_coding	chr19:10832067	1884	19.8848456	17.38718946	-0.282	1.09E-05	2.49E-05
AL365259.1	novel transcript, antisense to HEY2	lncRNA	chr6:125577545	4111	1.151603233	0.730487031	-0.751	1.09E-05	2.49E-05
SPHK1	sphingosine kinase 1	protein_coding	chr17:76376584	4416	1.459542755	1.029124075	-0.598	1.11E-05	2.52E-05
DHX35	DEAH-box helicase 35	protein_coding	chr20:38962299	4998	2.962404688	2.556532713	-0.299	1.13E-05	2.57E-05
NCAPD2	non-SMC condensin I complex subunit D2	protein_coding	chr12:6493356-	6470	8.411422596	7.405784911	-0.269	1.14E-05	2.59E-05
CLN8	CLN8 transmembrane ER and ERGIC prote	protein_coding	chr8:1755778-1	9459	2.129848351	1.711352041	-0.395	1.14E-05	2.59E-05
LRRC38	leucine rich repeat containing 38	protein_coding	chr1:13474973-	2168	0.846763078	0.392753424	-1.155	1.15E-05	2.60E-05
PIP4P2	phosphatidylinositol-4,5-bisphosphate 4-	protein_coding	chr8:90993802-	2868	8.012397916	6.334419804	-0.417	1.16E-05	2.63E-05
ZNF778	zinc finger protein 778	protein_coding	chr16:89217703	11273	1.603019916	1.312204747	-0.371	1.17E-05	2.64E-05
AC133552.5	novel transcript	lncRNA	chr16:25106569	534	1.782961999	0.665231518	-1.6	1.17E-05	2.65E-05
ORC6	origin recognition complex subunit 6	protein_coding	chr16:46689643	4309	3.454246883	2.659394575	-0.461	1.18E-05	2.67E-05
AP5B1	adaptor related protein complex 5 subuni	protein_coding	chr11:65775893	4811	4.142858672	3.41473498	-0.367	1.21E-05	2.72E-05
CACNA1H	calcium voltage-gated channel subunit alp	protein_coding	chr16:1153106-	8620	1.16437761	0.931577014	-0.405	1.22E-05	2.75E-05
AC105271.1	novel transcript	lncRNA	chr1:74468195-	905	3.295194373	2.133427076	-0.721	1.23E-05	2.76E-05
MAPK8IP1	mitogen-activated protein kinase 8 intera	protein_coding	chr11:45885651	3498	4.155069926	3.506217917	-0.334	1.24E-05	2.80E-05
SPC24	SPC24 component of NDC80 kinetochore	protein_coding	chr19:1113152C	2839	4.538807081	3.718868079	-0.375	1.26E-05	2.82E-05
SYNGR3	synaptogyrin 3	protein_coding	chr16:1989660-	3395	5.264297679	4.23403826	-0.392	1.26E-05	2.83E-05
C2CD3	C2 calcium dependent domain containing	protein_coding	chr11:74012714	12571	3.156525434	2.720762581	-0.299	1.26E-05	2.83E-05
APOBEC3B	apolipoprotein B mRNA editing enzyme ca	protein_coding	chr22:38982347	1896	1.027407027	0.579328127	-0.908	1.29E-05	2.89E-05
ZNF554	zinc finger protein 554	protein_coding	chr19:2819874-	4685	2.632436514	2.144174863	-0.38	1.31E-05	2.94E-05
BORA	BORA aurora kinase A activator	protein_coding	chr13:72727749	3189	1.284668822	0.862069727	-0.652	1.31E-05	2.94E-05
RPP30	ribonuclease P/MRP subunit p30	protein_coding	chr10:9087171€	4653	3.639703496	3.16505275	-0.285	1.32E-05	2.95E-05
IFT122	intraflagellar transport 122	protein_coding	chr3:12944003€	9636	2.609762787	2.233551989	-0.308	1.35E-05	3.01E-05
GALT	galactose-1-phosphate uridylyltransferase	protein_coding	chr9:34638133-	4407	5.101529505	4.205480584	-0.366	1.36E-05	3.04E-05
UBAP2	ubiquitin associated protein 2	protein_coding	chr9:33921693- 10620		4.559345508	3.894670789	-0.313	1.41E-05	3.13E-05
RPE	ribulose-5-phosphate-3-epimerase	protein_coding	chr2:210002565	3523	7.893302976	6.640768478	-0.33	1.46E-05	3.24E-05
POLE4	DNA polymerase epsilon 4, accessory subu	protein_coding	chr2:74958492-	2151	13.95585301	11.60556435	-0.352	1.47E-05	3.27E-05
POP5	POP5 homolog, ribonuclease P/MRP subu	protein_coding	chr12:12057876	2173	8.374768311	6.949082351	-0.356	1.51E-05	3.35E-05
ERCC8	ERCC excision repair 8, CSA ubiquitin ligas	protein_coding	chr5:60873831-	5448	2.059965337	1.734894337	-0.333	1.53E-05	3.38E-05
AP003486.1	novel transcript	lncRNA	chr11:13086625	3994	1.428187941	1.1026968	-0.459	1.54E-05	3.41E-05
AC019069.1	novel transcript	lncRNA	chr2:74832655-	1333	1.552208955	0.844784616	-0.942	1.59E-05	3.51E-05
CYREN	cell cycle regulator of NHEJ	protein_coding	chr7:13509236З	5321	4.835721086	4.241952026	-0.277	1.61E-05	3.55E-05
LIMD1	LIM domains containing 1	protein_coding	chr3:45555394-	12676	5.276949186	4.519517494	-0.307	1.61E-05	3.56E-05
THAP9-AS1	THAP9 antisense RNA 1	lncRNA	chr4:82893009-	4397	6.130031259	5.321690121	-0.289	1.64E-05	3.60E-05
SP1	Sp1 transcription factor	protein_coding	chr12:5338017€	8083	8.393705382	7.413334311	-0.264	1.64E-05	3.60E-05
GPATCH2L	G-patch domain containing 2 like	protein_coding	chr14:7615191€	21987	2.830693377	2.363891574	-0.346	1.66E-05	3.65E-05
ETFB	electron transfer flavoprotein subunit bet	protein_coding	chr19:51345169	6526	5.926552762	5.144340641	-0.292	1.67E-05	3.67E-05
CNOT4	CCR4-NOT transcription complex subunit	protein_coding	chr7:13536179€	6999	4.13844988	3.558662551	-0.303	1.67E-05	3.67E-05
IDUA	iduronidase alpha-L-	protein_coding	chr4:986997-10	4058	5.09359597	4.188082698	-0.365	1.70E-05	3.73E-05
TEX30	testis expressed 30	protein_coding	chr13:10276588	2256	1.909515538	1.406893169	-0.527	1.70E-05	3.73E-05
MFSD5	major facilitator superfamily domain con	protein_coding	chr12:53251251	2581	5.328361778	4.388150265	-0.364	1.71E-05	3.76E-05
TMEM205	transmembrane protein 205	protein_coding	chr19:1134277€	1606	16.5816751	14.2699755	-0.302	1.74E-05	3.81E-05
PSPH	phosphoserine phosphatase	protein_coding	chr7:56011051-	2762	7.082626578	6.202370333	-0.276	1.77E-05	3.87E-05
NIPA1	NIPA magnesium transporter 1	protein_coding	chr15:2277306З	8686	4.185651443	3.684339313	-0.27	1.83E-05	4.00E-05
C4orf48	chromosome 4 open reading frame 48	protein_coding	chr4:2041993-2	540	30.85219122	23.72014835	-0.475	1.84E-05	4.02E-05
PTOV1-AS2	PTOV1 antisense RNA 2	lncRNA	chr19:4985697C	1023	5.864642677	4.038100292	-0.612	1.84E-05	4.03E-05
UBE2C	ubiquitin conjugating enzyme E2 C	protein_coding	chr20:4581257€	1286	12.77209857	10.39826434	-0.381	1.87E-05	4.08E-05
QRICH2	glutamine rich 2	protein_coding	chr17:76274049	5775	1.436433013	1.140888773	-0.419	1.87E-05	4.08E-05
NRARP	NOTCH regulated ankyrin repeat protein	protein_coding	chr9:137299631	2641	2.747391108	2.171940987	-0.426	1.89E-05	4.11E-05
CDK5RAP2	CDK5 regulatory subunit associated prote	protein_coding	chr9:120388869 10080		4.917814055	4.293133471	-0.28	1.93E-05	4.21E-05
HDAC4	histone deacetylase 4	protein_coding	chr2:23904816€	13368	0.898161237	0.663096043	-0.525	1.94E-05	4.23E-05
RNF187	ring finger protein 187	protein_coding	chr1:228487382	3386	27.36448855	24.15055668	-0.266	1.97E-05	4.28E-05
HELLS	helicase, lymphoid specific	protein_coding	chr10:94501434 10150		1.019743578	0.770727834	-0.493	2.03E-05	4.42E-05
QSER1	glutamine and serine rich 1	protein_coding	chr11:32892820	10899	4.824760514	4.244187815	-0.27	2.07E-05	4.48E-05
MKI67	marker of proliferation Ki-67	protein_coding	chr10:12809665	12845	5.668718272	4.193432828	-0.52	2.11E-05	4.57E-05
PLEKHF1	pleckstrin homology and FYVE domain co	protein_coding	chr19:29665459	2298	1.093344159	0.71359825	-0.705	2.16E-05	4.66E-05
AHNAK	AHNAK nucleoprotein	protein_coding	chr11:62433542	19466	46.54765847	37.7986927	-0.38	2.19E-05	4.72E-05

CYB5D2	cytochrome b5 domain containing 2	protein_coding	chr17:4143168-	2182	5.74365033	4.953382985	-0.302	2.20E-05	4.74E-05
C2CD2	C2 calcium dependent domain containing	protein_coding	chr21:41885112	8730	0.947625442	0.759218976	-0.402	2.22E-05	4.79E-05
CKS1B	CDC28 protein kinase regulatory subunit	protein_coding	chr1:154974653	2340	6.447644658	5.151091682	-0.406	2.23E-05	4.80E-05
C2orf92	chromosome 2 open reading frame 92	protein_coding	chr2:97664217-	6501	1.01416771	0.774055989	-0.47	2.26E-05	4.86E-05
UMAD1	UBAP1-MVB12-associated (UMA) domain	protein_coding	chr7:7640711-7	3752	2.560418439	1.972133404	-0.462	2.27E-05	4.88E-05
LINC00235	long intergenic non-protein coding RNA 2	lncRNA	chr16:525155-5	2253	1.466691534	0.895864739	-0.795	2.30E-05	4.94E-05
CHST13	carbohydrate sulfotransferase 13	protein_coding	chr3:126524283	1789	1.644856992	1.0615915	-0.732	2.35E-05	5.03E-05
ZNF260	zinc finger protein 260	protein_coding	chr19:36510687	5571	3.934925517	3.316115605	-0.336	2.35E-05	5.04E-05
GEN1	GEN1 Holliday junction 5' flap endonucle	protein_coding	chr2:17753858-	11832	1.423938272	1.207670378	-0.323	2.35E-05	5.04E-05
UBALD2	UBA like domain containing 2	protein_coding	chr17:76265348	1650	11.42596342	9.851167268	-0.3	2.37E-05	5.08E-05
RAD51AP1	RAD51 associated protein 1	protein_coding	chr12:4538798-	2558	2.78336605	2.031944463	-0.539	2.42E-05	5.18E-05
AC091982.3	novel transcript	lncRNA	chr5:151770242	726	4.121831457	2.673784755	-0.708	2.45E-05	5.23E-05
GGT1	gamma-glutamyltransferase 1	protein_coding	chr22:24556007	3165	1.732737539	1.228958886	-0.567	2.48E-05	5.30E-05
SEMA5A	semaphorin 5A	protein_coding	chr5:9035033-9	12308	13.41572769	11.79159429	-0.27	2.49E-05	5.32E-05
POLA1	DNA polymerase alpha 1, catalytic subuni	protein_coding	chrX:24693919-	6336	4.212846988	3.618915079	-0.3	2.49E-05	5.32E-05
ARHGAP23	Rho GTPase activating protein 23	protein_coding	chr17:3841928(8080		17.71036351	15.07177129	-0.314	2.67E-05	5.69E-05
MT-CO1	mitochondrially encoded cytochrome c o	protein_coding	chrM:5904-7445	1542	15036.10768	12638.3864	-0.336	2.68E-05	5.71E-05
C1orf56	chromosome 1 open reading frame 56	protein_coding	chr1:15104774(	2331	5.426252875	4.592131598	-0.328	2.70E-05	5.73E-05
TRMT12	tRNA methyltransferase 12 homolog	protein_coding	chr8:12445082(	2331	3.777401365	3.142682399	-0.356	2.70E-05	5.73E-05
AL928654.1	novel transcript, antisense to MTA1	lncRNA	chr14:10541688	2365	3.878193831	3.108083119	-0.401	2.70E-05	5.74E-05
MAP3K1	mitogen-activated protein kinase kinase k	protein_coding	chr5:56815549-	7741	1.240022224	1.031495167	-0.353	2.80E-05	5.95E-05
ZNF584	zinc finger protein 584	protein_coding	chr19:58401504	3672	4.539677699	3.88796526	-0.309	2.82E-05	5.97E-05
C12orf43	chromosome 12 open reading frame 43	protein_coding	chr12:12100048	6140	2.089634826	1.699095522	-0.381	2.90E-05	6.14E-05
AL137847.2	novel transcript	lncRNA	chr9:83272280-	1514	0.951643262	0.466571154	-1.094	2.92E-05	6.18E-05
STK4	serine/threonine kinase 4	protein_coding	chr20:44966479	9132	1.848792207	1.611462812	-0.282	2.92E-05	6.18E-05
ZBTB34	zinc finger and BTB domain containing 34	protein_coding	chr9:126860665	6606	3.188568454	2.812379867	-0.265	2.92E-05	6.19E-05
MAFK	MAF bZIP transcription factor K	protein_coding	chr7:1530702-1	4470	24.68343503	19.74091016	-0.402	2.99E-05	6.32E-05
RAP2C-AS1	RAP2C antisense RNA 1	lncRNA	chrX:13221705З	5859	1.039164663	0.805695561	-0.458	3.01E-05	6.36E-05
CENPW	centromere protein W	protein_coding	chr6:126340174	795	2.895868826	1.88511135	-0.701	3.04E-05	6.41E-05
EOGT	EGF domain specific O-linked N-acetylgluc	protein_coding	chr3:68975217-	5365	2.854138467	2.424231681	-0.322	3.12E-05	6.57E-05
GLRX2	glutaredoxin 2	protein_coding	chr1:19309086(	6210	3.383215546	2.958714969	-0.28	3.12E-05	6.57E-05
PSMC3IP	PSMC3 interacting protein	protein_coding	chr17:42572315	2573	1.689560398	1.053275055	-0.75	3.15E-05	6.63E-05
PCED1A	PC-esterase domain containing 1A	protein_coding	chr20:2835314-	2320	11.70941993	9.870115493	-0.331	3.23E-05	6.78E-05
DERL3	derlin 3	protein_coding	chr22:23834503	3796	0.953746238	0.676807024	-0.581	3.25E-05	6.81E-05
PIM1	Pim-1 proto-oncogene, serine/threonine l	protein_coding	chr6:37170152- 3030		4.979896442	4.258866669	-0.308	3.26E-05	6.84E-05
METTL21A	methyltransferase like 21A	protein_coding	chr2:207580631	6798	1.352438729	1.025458924	-0.477	3.26E-05	6.84E-05
MRPL41	mitochondrial ribosomal protein L41	protein_coding	chr9:137551879	582	206.4432288	180.4617609	-0.278	3.27E-05	6.86E-05
PPM1B	protein phosphatase, Mg2+/Mn2+ depenc	protein_coding	chr2:44167969-	7105	3.457575025	2.924468599	-0.332	3.32E-05	6.95E-05
CCDC18	coiled-coil domain containing 18	protein_coding	chr1:93179919-	5633	1.144150787	0.851989939	-0.504	3.32E-05	6.95E-05
MOSPD1	motile sperm domain containing 1	protein_coding	chrX:134887632	2659	12.84006963	11.06347399	-0.3	3.36E-05	7.02E-05
C1orf50	chromosome 1 open reading frame 50	protein_coding	chr1:42767249-	5286	1.571860851	1.325831311	-0.334	3.37E-05	7.05E-05
PROSER3	proline and serine rich 3	protein_coding	chr19:35758143	6327	3.585581822	2.795150632	-0.441	3.40E-05	7.11E-05
CHKA	choline kinase alpha	protein_coding	chr11:68052859	3877	17.75969502	15.40301608	-0.292	3.42E-05	7.15E-05
AP003472.2	novel transcript	lncRNA	chr8:10042755S	2172	1.62843425	0.968776154	-0.855	3.43E-05	7.16E-05
KIF20B	kinesin family member 20B	protein_coding	chr10:8970161(	6851	2.893963968	2.37104942	-0.378	3.43E-05	7.16E-05
SLC31A2	solute carrier family 31 member 2	protein_coding	chr9:113150976	2473	3.999761003	3.340842225	-0.35	3.45E-05	7.19E-05
FAM174A	family with sequence similarity 174 meml	protein_coding	chr5:100535374	1347	15.10093557	13.12924676	-0.29	3.46E-05	7.22E-05
MCEE	methylmalonyl-CoA epimerase	protein_coding	chr2:71109684-	1624	7.665630169	6.647250637	-0.295	3.51E-05	7.32E-05
NDUFB1	NADH:ubiquinone oxidoreductase subuni	protein_coding	chr14:92116122	2263	35.30739346	30.26759629	-0.304	3.51E-05	7.32E-05
PIGO	phosphatidylinositol glycan anchor biosy	protein_coding	chr9:35088688-	7209	3.545058524	2.963967517	-0.339	3.53E-05	7.34E-05
CDT1	chromatin licensing and DNA replication 1	protein_coding	chr16:88803789	2760	3.351784529	2.693536379	-0.404	3.63E-05	7.55E-05
AC104463.2	novel transcript	lncRNA	chr1:20260426S	314	5.930526176	3.20347556	-0.957	3.65E-05	7.59E-05
AC021054.1	novel transcript	lncRNA	chr12:630858-6	4356	1.866013455	1.464014799	-0.436	3.66E-05	7.60E-05
CENPA	centromere protein A	protein_coding	chr2:26764289-	1949	2.302464064	1.734686607	-0.493	3.71E-05	7.70E-05
TATDN3	TatD DNase domain containing 3	protein_coding	chr1:21279182S	3992	4.289270093	3.719334602	-0.293	3.73E-05	7.74E-05
ATP5PO	ATP synthase peripheral stalk subunit OSC	protein_coding	chr21:33903453	3170	12.82330515	10.97725024	-0.307	3.74E-05	7.75E-05
AC009113.1	novel transcript, antisense to ZNF778	lncRNA	chr16:89215211	2443	1.946714598	1.415075268	-0.547	3.75E-05	7.78E-05
HS2ST1	heparan sulfate 2-O-sulfotransferase 1	protein_coding	chr1:86914635-	7426	5.929117634	5.256947813	-0.264	3.80E-05	7.86E-05
AC067930.3	novel transcript	lncRNA	chr8:143541973	498	1.009398035	0.410402798	-1.346	3.81E-05	7.88E-05
SULT1E1	sulfotransferase family 1E member 1	protein_coding	chr4:69841212-	2084	1.399885001	0.749085028	-1.026	3.81E-05	7.89E-05
ANKRD13B	ankyrin repeat domain 13B	protein_coding	chr17:29589769	4307	3.036798406	2.562437338	-0.327	3.86E-05	7.98E-05
TXLNB	taxilin beta	protein_coding	chr6:139240061	4796	8.649867797	7.643467927	-0.265	4.00E-05	8.26E-05
NEURL4	neuralized E3 ubiquitin protein ligase 4	protein_coding	chr17:7315628-	6170	8.752491342	7.332715653	-0.335	4.01E-05	8.27E-05
LIG1	DNA ligase 1	protein_coding	chr19:48115445	6576	3.686674045	3.253835206	-0.264	4.10E-05	8.44E-05
TMEM86A	transmembrane protein 86A	protein_coding	chr11:18693122	4243	1.399280991	1.061805279	-0.495	4.13E-05	8.49E-05
SIPA1L1	signal induced proliferation associated 1 l	protein_coding	chr14:71320449	12412	8.768477393	7.615629814	-0.286	4.13E-05	8.49E-05
TROAP	trophinin associated protein	protein_coding	chr12:49323236	3954	3.212023867	2.503102708	-0.442	4.22E-05	8.66E-05
NAA10	N(alpha)-acetyltransferase 10, NatA cataly	protein_coding	chrX:153929225	4616	3.914767939	3.45962163	-0.266	4.27E-05	8.77E-05
KLF12	Kruppel like factor 12	protein_coding	chr13:73686089	10923	3.617655472	3.184606709	-0.269	4.38E-05	8.99E-05
WWC1	WW and C2 domain containing 1	protein_coding	chr5:168291651	10076	1.611565403	1.393069288	-0.299	4.41E-05	9.02E-05
AC008608.2	novel transcript	lncRNA	chr5:134005147	416	3.482065352	2.111246836	-0.82	4.48E-05	9.17E-05
AF131215.3	novel transcript	lncRNA	chr8:11062647-	639	1.133960206	0.45833388	-1.36	4.50E-05	9.19E-05
AC144652.1	novel transcript	lncRNA	chr7:155295918	1624	1.157659633	0.674111376	-0.877	4.52E-05	9.23E-05
FAM131C	family with sequence similarity 131 meml	protein_coding	chr1:16057769-	1720	3.693159345	2.899106312	-0.43	4.52E-05	9.24E-05
ZNF286A	zinc finger protein 286A	protein_coding	chr17:15699577	6754	1.745623424	1.487083975	-0.323	4.61E-05	9.39E-05
ZNF35	zinc finger protein 35	protein_coding	chr3:44648732-	2658	3.784272715	3.223122381	-0.323	4.68E-05	9.53E-05
SP4	Sp4 transcription factor	protein_coding	chr7:21428043-	6225	2.547389592	2.172956014	-0.314	4.69E-05	9.55E-05
ARID4A	AT-rich interaction domain 4A	protein_coding	chr14:58298385	7568	3.403826476	2.977240325	-0.28	4.72E-05	9.60E-05
CISD1	CDGSH iron sulfur domain 1	protein_coding	chr10:58269162	2753	34.05639545	29.35546231	-0.297	4.81E-05	9.78E-05
CDCA8	cell division cycle associated 8	protein_coding	chr1:37692481-	2406	3.83565968	3.223920462	-0.341	4.94E-05	1.00E-04
LYSMD2	LysM domain containing 2	protein_coding	chr15:51723011	1874	3.030202047	2.395049117	-0.429	5.01E-05	1.02E-04
LZTS3	leucine zipper tumor suppressor family m	protein_coding	chr20:3162617-	5395	2.521121528	2.138073781	-0.323	5.02E-05	1.02E-04
VPS11	VPS11 core subunit of CORVET and HOPS c	protein_coding	chr11:11906769	5907	6.091224105	5.311678919	-0.28	5.08E-05	1.03E-04
TNNT2	troponin T2, cardiac type	protein_coding	chr1:201359008	7342	80.91511807	69.83746359	-0.297	5.11E-05	1.04E-04
HSPA6	heat shock protein family A (Hsp70) meml	protein_coding	chr1:16152454(	2355	2.435478484	1.813976055	-0.513	5.14E-05	1.04E-04
PKDCC	protein kinase domain containing, cytopl	protein_coding	chr2:42048021-	3913	9.23503662	8.030715967	-0.289	5.15E-05	1.04E-04
MELK	maternal embryonic leucine zipper kinase	protein_coding	chr9:36572862-	3087	2.422696748	1.992401284	-0.365	5.16E-05	1.04E-04
ARAP2	ArfGAP with RhoGAP domain, ankyrin rep	protein_coding	chr4:35948221-	12427	1.288168036	1.107988382	-0.307	5.28E-05	1.07E-04
ZNF700	zinc finger protein 700	protein_coding	chr19:11925068	3319	2.571977982	2.173789762	-0.326	5.29E-05	1.07E-04
LRIG1	leucine rich repeats and immunoglobulin	protein_coding	chr3:66378797-	7032	14.37860204	12.03198704	-0.338	5.30E-05	1.07E-04
RSRC1	arginine and serine rich coiled-coil 1	protein_coding	chr3:158105855	5117	2.185837963	1.807954685	-0.366	5.37E-05	1.08E-04
ACAT2	acetyl-CoA acetyltransferase 2	protein_coding	chr6:159760328	4299	4.349004452	3.565753688	-0.368	5.44E-05	1.10E-04
KIF2C	kinesin family member 2C	protein_coding	chr1:44739818-	3646	3.929113057	3.255646275	-0.358	5.54E-05	1.12E-04
NDOR1	NADPH dependent diflavin oxidoreductas	protein_coding	chr9:137205685	2836	6.308352409	4.860349492	-0.46	5.60E-05	1.13E-04
WHAMM	WASP homolog associated with actin, gol	protein_coding	chr15:82809628	5123	2.972621244	2.591663788	-0.282	5.60E-05	1.13E-04
CSNK2B	casein kinase 2 beta	protein_coding	chr6:31665236-	2933	2.522528996	1.964033242	-0.445	5.76E-05	1.16E-04
TMEM160	transmembrane protein 160	protein_coding	chr19:47045909	655	19.339837	14.88350988	-0.479	5.93E-05	1.19E-04
Z85996.3	novel transcript	lncRNA	chr6:36841430-	1022	1.02090476	0.349017453	-1.676	6.22E-05	1.25E-04
CHIC2	cysteine rich hydrophobic domain 2	protein_coding	chr4:54009789-	1233	9.623035302	7.983253498	-0.359	6.42E-05	1.28E-04
AC135050.6	novel transcript, sense intronic to KAT8	lncRNA	chr16:31122235	756	16.45594014	14.23857208	-0.293	6.42E-05	1.29E-04
CAB39L	calcium binding protein 39 like	protein_coding	chr13:4930865(	4944	4.375832354	3.580605832	-0.373	6.53E-05	1.30E-04
LRP4	LDL receptor related protein 4	protein_coding	chr11:46856717	8748	6.092451532	5.036714655	-0.353	6.54E-05	1.31E-04
NDUFS8	NADH:ubiquinone oxidoreductase core su	protein_coding	chr11:68030617	4523	29.88897973	26.0753046	-0.28	6.76E-05	1.35E-04
FUT11	fucosyltransferase 11	protein_coding	chr10:73772276	4925	6.430248571	5.318417373	-0.359	6.87E-05	1.37E-04
IMMP1L	inner mitochondrial membrane peptidase	protein_coding	chr11:31432401	3901	2.518780495	2.043414093	-0.39	6.99E-05	1.39E-04
AC068473.5	novel transcript	lncRNA	chr18:79677287	2072	2.385100964	1.813878184	-0.479	7.06E-05	1.40E-04
NABP2	nucleic acid binding protein 2	protein_coding	chr12:56222015	2201	6.581213423	5.77281359	-0.281	7.24E-05	1.44E-04
ERBB3	erb-b2 receptor tyrosine kinase 3	protein_coding	chr12:56076799	9039	1.383004985	1.102691132	-0.414	7.27E-05	1.44E-04
TNNC1	troponin C1, slow skeletal and cardiac typ	protein_coding	chr3:52451102-	1168	2324.954171	2051.366924	-0.264	7.27E-05	1.44E-04

AC022100.1	novel transcript	lncRNA	chr5:149694237	680	0.697434657	0.220577167	-1.635	7.31E-05	1.45E-04
TRIP13	thyroid hormone receptor interactor 13	protein_coding	chr5:892884-91	3319	2.403960118	1.825449842	-0.487	7.32E-05	1.45E-04
EPOP	elongin BC and polycomb repressive comp	protein_coding	chr17:38671703	3719	5.40452959	4.537831773	-0.332	7.52E-05	1.49E-04
SH3BP5-AS1	SH3BP5 antisense RNA 1	lncRNA	chr3:15254184-	6877	1.551850656	1.26961039	-0.371	7.61E-05	1.50E-04
CKAP2L	cytoskeleton associated protein 2 like	protein_coding	chr2:112736349	5464	1.910602887	1.625534959	-0.317	7.61E-05	1.50E-04
HCN3	hyperpolarization activated cyclic nucleo	protein_coding	chr1:155277463	4241	1.676400228	1.32895394	-0.425	7.62E-05	1.50E-04
SIK1	salt inducible kinase 1	protein_coding	chr21:43414483	5268	1.75496105	1.483013926	-0.323	7.63E-05	1.51E-04
WDFY3	WD repeat and FYVE domain containing 3	protein_coding	chr4:84669610-	16856	6.63894175	5.714409892	-0.298	7.67E-05	1.51E-04
FAM118B	family with sequence similarity 118 mem	protein_coding	chr11:12621172	3534	4.813110415	4.246199884	-0.272	7.70E-05	1.52E-04
MACROD1	mono-ADP ribosylhydrolase 1	protein_coding	chr11:63998558	2096	17.96921165	15.5234877	-0.294	7.80E-05	1.54E-04
PFAS	phosphoribosylformylglycinamidine synt	protein_coding	chr17:8247618-	6471	7.493724902	6.557617993	-0.276	7.92E-05	1.56E-04
AC139530.2	novel transcript, antisense to HGS	lncRNA	chr17:81701324	1977	2.927779292	2.392459682	-0.377	7.95E-05	1.56E-04
OBSL1	obscurin like 1	protein_coding	chr2:219550728	9716	67.61011133	56.97540787	-0.329	8.01E-05	1.57E-04
AC124045.1	novel transcript	lncRNA	chr3:44667412-	1953	1.170520212	0.765802063	-0.699	8.08E-05	1.59E-04
HMGN5	high mobility group nucleosome binding	protein_coding	chrX:81113701-	2416	4.871658407	3.746747421	-0.453	8.13E-05	1.59E-04
CYP11A1	cytochrome P450 family 11 subfamily A n	protein_coding	chr15:74337759	3397	0.984350622	0.720872242	-0.538	8.29E-05	1.63E-04
MMP1	matrix metallopeptidase 1	protein_coding	chr11:10278991	1971	0.923726886	0.430474057	-1.25	8.41E-05	1.65E-04
ARID1A	AT-rich interaction domain 1A	protein_coding	chr1:26693236-	15939	14.78755647	12.68009096	-0.304	8.50E-05	1.66E-04
SKA3	spindle and kinetochore associated comp	protein_coding	chr13:21153595	2998	1.689660865	1.349649173	-0.412	8.55E-05	1.67E-04
PLSCR1	phospholipid scramblase 1	protein_coding	chr3:146515180	4332	0.932451155	0.664240528	-0.568	8.59E-05	1.68E-04
NT5C3A	5'-nucleotidase, cytosolic IIIA	protein_coding	chr7:33014114-	3494	3.4509061	3.016411404	-0.28	8.69E-05	1.70E-04
ADCY1	adenylate cyclase 1	protein_coding	chr7:45574140-	13966	1.799021183	1.484799029	-0.355	8.75E-05	1.71E-04
GAL	galanin and GMAP prepropeptide	protein_coding	chr11:68683779	1300	0.7440517	0.215393161	-1.816	8.77E-05	1.71E-04
ENKD1	enkurin domain containing 1	protein_coding	chr16:67662945	2665	3.95435852	3.397918735	-0.306	8.79E-05	1.72E-04
NR4A1	nuclear receptor subfamily 4 group A mer	protein_coding	chr12:52022832	7295	1.467863302	1.212837328	-0.366	8.81E-05	1.72E-04
PIF1	PIF1 5'-to-3' DNA helicase	protein_coding	chr15:64815632	4656	1.19331778	0.832282115	-0.593	8.95E-05	1.74E-04
KIF18A	kinesin family member 18A	protein_coding	chr11:28020619	4517	2.02053837	1.643893824	-0.389	8.98E-05	1.75E-04
STARD7-AS1	STARD7 antisense RNA 1	lncRNA	chr2:96208403-	4413	3.390829667	2.972680169	-0.277	9.02E-05	1.76E-04
GNPTG	N-acetylglucosamine-1-phosphate transfe	protein_coding	chr16:1351931-	4094	9.059826107	7.911841628	-0.277	9.26E-05	1.80E-04
AURKB	aurora kinase B	protein_coding	chr17:8204733-	2241	4.382601156	3.57800003	-0.372	9.33E-05	1.81E-04
FLRT3	fibronectin leucine rich transmembrane p	protein_coding	chr20:14322985	5191	1.665600562	1.353709891	-0.388	9.33E-05	1.81E-04
AC138904.3	novel transcript	lncRNA	chr16:28258686	2681	1.530724117	1.163680394	-0.477	9.37E-05	1.82E-04
ALS2CL	ALS2 C-terminal like	protein_coding	chr3:46668995-	9059	0.872452878	0.666877314	-0.468	9.62E-05	1.86E-04
MOK	MOK protein kinase	protein_coding	chr14:10222450	10986	1.068184932	0.939309457	-0.274	9.66E-05	1.87E-04
PMF1	polyamine modulated factor 1	protein_coding	chr1:156212993	2520	7.093128643	5.661367666	-0.405	9.93E-05	1.92E-04
BUB1B	BUB1 mitotic checkpoint serine/threonin	protein_coding	chr15:40161023	4476	4.425686549	3.751692307	-0.325	9.98E-05	1.93E-04
CDC7	cell division cycle 7	protein_coding	chr1:91500851-	3599	1.542307103	1.214525825	-0.427	9.98E-05	1.93E-04
PLBD1	phospholipase B domain containing 1	protein_coding	chr12:14503661	2375	4.010695932	3.461847105	-0.293	1.01E-04	1.95E-04
BX322562.1	novel transcript	lncRNA	chr21:45403809	561	2.961953103	1.87601071	-0.757	1.01E-04	1.95E-04
CCDC78	coiled-coil domain containing 78	protein_coding	chr16:722582-7	4147	0.844468435	0.603294028	-0.562	1.02E-04	1.97E-04
VEGFB	vascular endothelial growth factor B	protein_coding	chr11:64234538	1545	81.55021703	71.79963449	-0.267	1.02E-04	1.97E-04
ZNF93	zinc finger protein 93	protein_coding	chr19:19900913	5579	1.27508799	1.006219532	-0.426	1.02E-04	1.97E-04
FOXRED2	FAD dependent oxidoreductase domain c	protein_coding	chr22:36487190	5577	5.338215593	4.642988989	-0.28	1.03E-04	1.98E-04
LKAAEAR1	LKAAEAR motif containing 1	protein_coding	chr20:64083380	868	2.188774062	1.066827538	-1.165	1.04E-04	1.99E-04
SDHB	succinate dehydrogenase complex iron su	protein_coding	chr1:17018722-	2462	48.36465876	42.57243118	-0.268	1.04E-04	2.00E-04
TMEM175	transmembrane protein 175	protein_coding	chr4:932387-95	3623	5.54503679	4.867680895	-0.269	1.04E-04	2.01E-04
EME1	essential meiotic structure-specific endor	protein_coding	chr17:50373220	3294	1.320855156	0.945149814	-0.574	1.05E-04	2.02E-04
BIK	BCL2 interacting killer	protein_coding	chr22:43110750	951	3.145282706	2.153421547	-0.634	1.06E-04	2.03E-04
LEAP2	liver enriched antimicrobial peptide 2	protein_coding	chr5:132872322	2405	1.288459106	0.901645955	-0.606	1.06E-04	2.03E-04
SVIL	supervillin	protein_coding	chr10:29457338	9937	103.8337437	89.55139498	-0.294	1.09E-04	2.08E-04
GUCA1B	guanylate cyclase activator 1B	protein_coding	chr6:42183284-	2270	1.726265893	1.180253668	-0.63	1.10E-04	2.11E-04
MT-CO2	mitochondrially encoded cytochrome c o	protein_coding	chrM:7586-8269	684	13143.22213	10728.17302	-0.373	1.11E-04	2.13E-04
DUS3L	dihydrouridine synthase 3 like	protein_coding	chr19:5784832-	3629	5.468608046	4.77267123	-0.283	1.13E-04	2.16E-04
XRCC4	X-ray repair cross complementing 4	protein_coding	chr5:83077498-	2676	3.660771099	2.980537273	-0.381	1.14E-04	2.18E-04
BX323046.1	novel transcript	lncRNA	chr1:244864738	535	1.486543365	0.690146148	-1.154	1.15E-04	2.20E-04
SLC16A5	solute carrier family 16 member 5	protein_coding	chr17:75087727	3089	4.159929525	3.27459415	-0.424	1.18E-04	2.26E-04
BACH2	BTB domain and CNC homolog 2	protein_coding	chr6:89926528-	9787	1.33056722	1.127389469	-0.326	1.19E-04	2.26E-04
AL354836.1	novel transcript	lncRNA	chr20:62305432	681	1.508838819	0.765609507	-1.065	1.19E-04	2.26E-04
GTSE1	G2 and S-phase expressed 1	protein_coding	chr22:46296870	4113	3.735967905	3.015863335	-0.392	1.19E-04	2.26E-04
MRPL34	mitochondrial ribosomal protein L34	protein_coding	chr19:17292609	1686	68.83689905	58.24933466	-0.321	1.19E-04	2.27E-04
AC092287.1	novel transcript, antisense to SMG1	lncRNA	chr16:18926863	463	0.782185521	0.265287887	-1.521	1.20E-04	2.29E-04
AL049775.1	novel transcript	lncRNA	chr14:85518871	4193	0.965027049	0.611314211	-0.754	1.21E-04	2.31E-04
CEP55	centrosomal protein 55	protein_coding	chr10:93496632	3271	3.427869436	2.622780525	-0.464	1.23E-04	2.34E-04
TAF1C	TATA-box binding protein associated facto	protein_coding	chr16:84177847	5577	6.957290927	6.139388683	-0.264	1.24E-04	2.35E-04
SLC25A28	solute carrier family 25 member 28	protein_coding	chr10:99610522	1830	7.143389059	6.091162257	-0.319	1.25E-04	2.38E-04
AC009779.2	novel transcript, antisense to CD63	lncRNA	chr12:55729104	1443	10.70014232	9.407761912	-0.272	1.30E-04	2.46E-04
OLFM2	olfactomedin 2	protein_coding	chr19:9853718-	2500	1.152604081	0.826312587	-0.582	1.33E-04	2.51E-04
SDHAF3	succinate dehydrogenase complex assemb	protein_coding	chr7:97116590-	2303	4.854135754	4.171329645	-0.304	1.34E-04	2.54E-04
TMEM185A	transmembrane protein 185A	protein_coding	chrX:149596556	5158	4.679546046	3.982449246	-0.323	1.35E-04	2.56E-04
ASPRV1	aspartic peptidase retroviral like 1	protein_coding	chr2:69960089-	2177	1.360243225	0.913135795	-0.682	1.40E-04	2.64E-04
AC107294.1	novel transcript	lncRNA	chr3:184742818	467	0.810042907	0.135824903	-2.822	1.40E-04	2.64E-04
C1QTNF1	C1q and TNF related 1	protein_coding	chr17:79022814	4755	1.103299146	0.751759611	-0.617	1.41E-04	2.66E-04
MED19	mediator complex subunit 19	protein_coding	chr11:57703714	1877	6.458800187	5.438320053	-0.331	1.42E-04	2.68E-04
DCLRE1A	DNA cross-link repair 1A	protein_coding	chr10:11383472	4582	2.235349394	1.92186326	-0.31	1.43E-04	2.69E-04
NEMP1	nuclear envelope integral membrane prot	protein_coding	chr12:57055643	5730	2.060938463	1.799794812	-0.279	1.43E-04	2.70E-04
MRPS31	mitochondrial ribosomal protein S31	protein_coding	chr13:40729135	2124	11.30735062	9.881560845	-0.277	1.46E-04	2.75E-04
PROSER2	proline and serine rich 2	protein_coding	chr10:11823339	3663	1.308852488	1.055167466	-0.398	1.55E-04	2.90E-04
LINC01621	long intergenic non-protein coding RNA 1	lncRNA	chr6:79803582-	1473	2.078009436	1.403153121	-0.651	1.56E-04	2.91E-04
TTC9	tetratricopeptide repeat domain 9	protein_coding	chr14:70641916	5094	1.694935436	1.399530838	-0.364	1.57E-04	2.93E-04
AC114271.1	novel transcript, antisense to ICAM3	lncRNA	chr19:10333436	2813	1.633415972	1.219296123	-0.512	1.57E-04	2.94E-04
VAMP5	vesicle associated membrane protein 5	protein_coding	chr2:85584431-	790	0.998599003	0.475097393	-1.145	1.58E-04	2.95E-04
OBSCN	obscurin, cytoskeletal calmodulin and titi	protein_coding	chr1:228208063	39549	20.88304688	16.95215778	-0.378	1.66E-04	3.09E-04
FRRS1	ferric chelate reductase 1	protein_coding	chr1:99708632-	3958	1.656346476	1.372474868	-0.352	1.70E-04	3.17E-04
NDUFB10	NADH:ubiquinone oxidoreductase subuni	protein_coding	chr16:1959538- 1040		148.5616062	128.1094652	-0.295	1.71E-04	3.18E-04
HERC1	HECT and RLD domain containing E3 ubiq	protein_coding	chr15:63608618	17059	9.318389337	8.103982801	-0.282	1.71E-04	3.19E-04
OCEL1	occludin/ELL domain containing 1	protein_coding	chr19:17226213	1858	6.300440542	5.382856445	-0.318	1.77E-04	3.28E-04
SLC46A1	solute carrier family 46 member 1	protein_coding	chr17:28394642	7954	1.155984598	0.963180695	-0.351	1.78E-04	3.29E-04
NDUFB11	NADH:ubiquinone oxidoreductase subuni	protein_coding	chrX:47142216-	1418	70.30967756	60.48250303	-0.297	1.78E-04	3.30E-04
ZNF324	zinc finger protein 324	protein_coding	chr19:58467045	5664	1.748276059	1.514086647	-0.291	1.78E-04	3.30E-04
IFI6	interferon alpha inducible protein 6	protein_coding	chr1:27666064-	842	21.74921286	17.24229421	-0.438	1.85E-04	3.41E-04
PGBD4	piggyBac transposable element derived 4	protein_coding	chr15:34102083	6604	1.584567483	1.396270257	-0.265	1.90E-04	3.51E-04
PRR7	proline rich 7, synaptic	protein_coding	chr5:177446445	1753	3.645057658	3.040087873	-0.345	1.91E-04	3.52E-04
ADHFE1	alcohol dehydrogenase iron containing 1	protein_coding	chr8:66432492-	3378	3.53130278	2.69417124	-0.46	1.93E-04	3.56E-04
SREBF2-AS1	SREBF2 antisense RNA 1	lncRNA	chr22:41831215	2989	4.652543733	4.109936617	-0.271	1.94E-04	3.57E-04
RFC4	replication factor C subunit 4	protein_coding	chr3:186789880	2997	3.567374106	3.064922889	-0.301	1.98E-04	3.63E-04
FAM76A	family with sequence similarity 76 memb	protein_coding	chr1:27725979-	4034	3.3080561	2.891427095	-0.281	2.02E-04	3.71E-04
AIFM2	apoptosis inducing factor mitochondria a	protein_coding	chr10:70098223	5135	2.388279054	2.016401011	-0.334	2.02E-04	3.71E-04
SNAPC1	small nuclear RNA activating complex pol	protein_coding	chr14:61762420	2593	2.614046891	2.059753552	-0.427	2.06E-04	3.77E-04
SYN1	synapsin I	protein_coding	chrX:47571901-	3733	3.117824607	2.731093613	-0.274	2.11E-04	3.86E-04
FAM216A	family with sequence similarity 216 mem	protein_coding	chr12:11046841	2702	6.815584333	6.01752065	-0.264	2.19E-04	4.01E-04
ACTR5	actin related protein 5	protein_coding	chr20:38748460	2547	2.480451372	2.043073313	-0.368	2.26E-04	4.12E-04
PDXP	pyridoxal phosphatase	protein_coding	chr22:37658723	2205	3.98212521	2.074934846	-1.022	2.29E-04	4.17E-04
ZXDB	zinc finger X-linked duplicated B	protein_coding	chrX:57591652-	5894	2.396159531	2.078051158	-0.292	2.33E-04	4.23E-04
PNPO	pyridoxamine 5'-phosphate oxidase	protein_coding	chr17:47941506	5226	1.293292269	1.06678648	-0.361	2.36E-04	4.29E-04
SRRM2	serine/arginine repetitive matrix 2	protein_coding	chr16:2752626-	12969	61.91904265	52.34146305	-0.326	2.36E-04	4.30E-04
WASHC3	WASH complex subunit 3	protein_coding	chr12:10201292	2593	4.272717635	3.530219213	-0.354	2.42E-04	4.39E-04
MFSD9	major facilitator superfamily domain con	protein_coding	chr2:102714630	5577	1.856656818	1.574727937	-0.326	2.48E-04	4.49E-04
MND1	meiotic nuclear divisions 1	protein_coding	chr4:153344649	1511	2.324839322	1.761794834	-0.482	2.50E-04	4.53E-04
TIMM23B	translocase of inner mitochondrial memb	protein_coding	chr10:49942049 3040		2.329586095	2.009043782	-0.297	2.54E-04	4.60E-04

FNTB	farnesyltransferase, CAAX box, beta	protein_coding	chr14:64986895	3773	3.336309197	2.829528319	-0.318	2.55E-04	4.61E-04
FUNDC1	FUN14 domain containing 1	protein_coding	chrX:44523639-	1257	15.72658988	13.77071346	-0.275	2.55E-04	4.61E-04
AL513534.2	novel transcript, overlapping to TET1	lncRNA	chr10:68698500	2295	2.637558961	1.997661556	-0.484	2.55E-04	4.61E-04
AL049838.1	novel transcript, overlapping C14orf37	lncRNA	chr14:57993545	981	2.405744718	1.332876606	-0.906	2.57E-04	4.64E-04
PUS7	pseudouridine synthase 7	protein_coding	chr7:105439661	4433	2.697098962	2.282911083	-0.327	2.58E-04	4.66E-04
ALDH16A1	aldehyde dehydrogenase 16 family memb	protein_coding	chr19:49453225	4589	2.849718865	2.372746387	-0.35	2.76E-04	4.97E-04
SLC25A19	solute carrier family 25 member 19	protein_coding	chr17:75272981	3316	4.374306948	3.637780911	-0.344	2.77E-04	4.98E-04
AP003068.3	novel transcript	lncRNA	chr11:65117157	175	2.209173153	1.068803434	-1.083	2.89E-04	5.19E-04
RHBDL1	rhomboid like 1	protein_coding	chr16:675666-6	1884	3.71634555	2.98132707	-0.409	2.94E-04	5.26E-04
KIAA1671	KIAA1671	protein_coding	chr22:24952730	16439	3.126109818	2.664875432	-0.309	2.96E-04	5.30E-04
MED12	mediator complex subunit 12	protein_coding	chrX:71118556-	7425	6.827645662	6.001179393	-0.267	3.07E-04	5.49E-04
COX7B	cytochrome c oxidase subunit 7B	protein_coding	chrX:77899440-	3025	75.2164535	63.57666131	-0.321	3.09E-04	5.51E-04
LCAT	lecithin-cholesterol acyltransferase	protein_coding	chr16:67939750	2625	6.702858666	5.540197827	-0.356	3.15E-04	5.63E-04
BABAM1	BRISC and BRCA1 A complex member 1	protein_coding	chr19:17267376	2581	4.537177296	4.020543957	-0.267	3.21E-04	5.73E-04
FAM110C	family with sequence similarity 110 meml	protein_coding	chr2:38814-468	4088	1.369234816	1.102261805	-0.401	3.27E-04	5.82E-04
ADGRA2	adhesion G protein-coupled receptor A2	protein_coding	chr8:37784191-	7061	1.805091746	1.510288095	-0.339	3.31E-04	5.89E-04
TGFB3	transforming growth factor beta 3	protein_coding	chr14:75958099	4914	1.275304361	1.011981345	-0.415	3.32E-04	5.90E-04
NEAT1	nuclear paraspeckle assembly transcript 1	lncRNA	chr11:65422774	22767	12.13882974	9.297737181	-0.463	3.36E-04	5.97E-04
LPXN	leupaxin	protein_coding	chr11:58526871	2467	2.356032993	1.936113948	-0.359	3.39E-04	6.03E-04
ARSG	arylsulfatase G	protein_coding	chr17:68259182	6587	1.541256971	1.354993877	-0.268	3.40E-04	6.04E-04
AC005696.1	novel transcript, antisense to CLUH	lncRNA	chr17:2712309-	443	4.198093337	2.885566425	-0.622	3.46E-04	6.14E-04
CENPJ	centromere protein J	protein_coding	chr13:24882279	5817	1.322291732	1.10215332	-0.34	3.47E-04	6.16E-04
ALPK3	alpha kinase 3	protein_coding	chr15:8481668(	10940	65.23359395	56.19774912	-0.294	3.55E-04	6.29E-04
KTN1-AS1	KTN1 antisense RNA 1	lncRNA	chr14:55496786	8113	1.146121739	0.977367918	-0.318	3.57E-04	6.33E-04
SPAG5	sperm associated antigen 5	protein_coding	chr17:28577565	5218	2.801206287	2.324912803	-0.355	3.62E-04	6.40E-04
ZBTB6	zinc finger and BTB domain containing 6	protein_coding	chr9:122908056	4099	2.8841337	2.559616896	-0.265	3.67E-04	6.49E-04
DGCR11	DiGeorge syndrome critical region gene 1	lncRNA	chr22:19046162	2214	1.308631669	0.979097655	-0.501	3.67E-04	6.49E-04
ZNF433	zinc finger protein 433	protein_coding	chr19:12014732	3079	1.633004244	1.263816668	-0.446	3.71E-04	6.54E-04
POGLUT2	protein O-glucosyltransferase 2	protein_coding	chr13:10278428	2336	3.141374929	2.468431311	-0.431	3.72E-04	6.56E-04
BOLA3	bolA family member 3	protein_coding	chr2:74135400-	3072	6.632149641	5.819472628	-0.27	3.76E-04	6.62E-04
ADSL	adenylosuccinate lyase	protein_coding	chr22:40346500	7111	2.104202055	1.833806547	-0.285	3.80E-04	6.69E-04
MPP6	membrane palmitoylated protein 6	protein_coding	chr7:24573268-	9369	1.150238387	1.013805755	-0.264	3.86E-04	6.79E-04
FAM174B	family with sequence similarity 174 meml	protein_coding	chr15:92617448	4469	2.540364989	2.239037583	-0.271	3.89E-04	6.84E-04
AL122023.1	novel transcript, sense intronic UNC79	lncRNA	chr14:93334528	530	1.213917877	0.633872925	-1.086	3.98E-04	6.97E-04
SF3B6	splicing factor 3b subunit 6	protein_coding	chr2:24067586-	1125	37.82362663	31.94198747	-0.325	4.06E-04	7.10E-04
HDAC7	histone deacetylase 7	protein_coding	chr12:47782722	9928	4.586561652	3.866023322	-0.328	4.24E-04	7.39E-04
ART5	ADP-ribosyltransferase 5	protein_coding	chr11:3638503-	1691	1.402638845	0.922653126	-0.695	4.38E-04	7.62E-04
ZIK1	zinc finger protein interacting with K prot	protein_coding	chr19:57578456	5370	0.987520262	0.798824983	-0.393	4.41E-04	7.66E-04
HCST	hematopoietic cell signal transducer	protein_coding	chr19:35902480	613	1.419942857	0.814401524	-0.847	4.46E-04	7.76E-04
CA14	carbonic anhydrase 14	protein_coding	chr1:150257251	3296	2.004939702	1.581770903	-0.419	4.51E-04	7.84E-04
FAM83H	family with sequence similarity 83 memb	protein_coding	chr8:143723933	6220	3.122880414	2.75423651	-0.265	4.57E-04	7.93E-04
NBR2	neighbor of BRCA1 lncRNA 2	lncRNA	chr17:43125551	3048	1.658668746	1.390343462	-0.344	4.62E-04	8.00E-04
SEMA3B	semaphorin 3B	protein_coding	chr3:50267558-	5768	2.170348203	1.869276651	-0.299	4.63E-04	8.02E-04
PDK1	pyruvate dehydrogenase kinase 1	protein_coding	chr2:172555373	14883	18.39452068	15.48061699	-0.338	4.69E-04	8.12E-04
HYLS1	HYLS1 centriolar and ciliogenesis associat	protein_coding	chr11:12588361	2999	1.646250706	1.387582327	-0.342	4.84E-04	8.37E-04
C12orf65	chromosome 12 open reading frame 65	protein_coding	chr12:12323291	7090	3.095578184	2.717781712	-0.269	4.84E-04	8.37E-04
EBP	EBP cholestenol delta-isomerase	protein_coding	chrX:48521799-	1768	5.527309421	4.50466815	-0.383	4.86E-04	8.39E-04
TWNK	twinkle mtDNA helicase	protein_coding	chr10:10098736	4135	2.252654963	1.945400913	-0.307	4.92E-04	8.49E-04
SLC16A12	solute carrier family 16 member 12	protein_coding	chr10:89430299	4873	7.930176957	6.99856617	-0.267	5.14E-04	8.84E-04
FAM229B	family with sequence similarity 229 meml	protein_coding	chr6:112087591	2551	6.117490001	5.401367947	-0.268	5.21E-04	8.95E-04
EDEM2	ER degradation enhancing alpha-mannosi	protein_coding	chr20:35115364	1884	6.251108023	5.451789889	-0.284	5.22E-04	8.96E-04
CDKN3	cyclin dependent kinase inhibitor 3	protein_coding	chr14:54396849	1836	4.017950415	3.107412158	-0.444	5.41E-04	9.27E-04
AL157932.1	novel transcript	lncRNA	chr13:42043727	363	1.178023206	0.53283473	-1.136	5.43E-04	9.31E-04
FUOM	fucose mutarotase	protein_coding	chr10:13335515	1270	5.296132177	4.163425702	-0.425	5.63E-04	9.62E-04
TMEM74	transmembrane protein 74	protein_coding	chr8:108606850	7419	1.334431776	1.168384214	-0.288	5.72E-04	9.78E-04
FGF12-AS3	FGF12 antisense RNA 3	lncRNA	chr3:192516831	291	1.453719029	0.740424599	-1.034	5.95E-04	1.01E-03
SAMD10	sterile alpha motif domain containing 10	protein_coding	chr20:63974116	2450	4.912178032	4.312276515	-0.27	5.95E-04	1.01E-03
UCN	urocortin	protein_coding	chr2:27307400-	795	1.935939043	1.310777008	-0.652	5.99E-04	1.02E-03
CCDC57	coiled-coil domain containing 57	protein_coding	chr17:82101460	12394	2.310852639	2.044489077	-0.264	6.06E-04	1.03E-03
HIST1H4H	histone cluster 1 H4 family member h	protein_coding	chr6:26277609-	2163	3.204832616	2.687930544	-0.337	6.09E-04	1.04E-03
AL035071.1	novel transcript	lncRNA	chr20:32856621	2131	1.023420645	0.519412634	-1.02	6.10E-04	1.04E-03
ATRIP	ATR interacting protein	protein_coding	chr3:48446710-	5431	1.365915847	1.178639146	-0.297	6.23E-04	1.06E-03
AL358472.4	novel transcript, antisense to SLC39A1	lncRNA	chr1:153964361	710	1.420569696	0.965334114	-0.647	6.34E-04	1.08E-03
TCF19	transcription factor 19	protein_coding	chr6:31158547-	3533	3.438086901	2.995882811	-0.278	6.41E-04	1.09E-03
CIAO2A	cytosolic iron-sulfur assembly componen	protein_coding	chr15:64072565	2260	27.61837377	24.08986249	-0.276	6.47E-04	1.10E-03
SLC9A3-AS1	SLC9A3 antisense RNA 1	lncRNA	chr5:473236-48	4731	8.379263736	7.254984735	-0.291	6.63E-04	1.12E-03
AC025165.5	novel transcript	lncRNA	chr12:57803838	578	5.480369213	4.377137488	-0.411	6.67E-04	1.13E-03
PPP1R14A	protein phosphatase 1 regulatory inhibito	protein_coding	chr19:38251237	1442	0.9220002	0.627083622	-0.627	6.69E-04	1.13E-03
ANO4	anoctamin 4	protein_coding	chr12:10071752	4997	1.183822796	1.00055485	-0.332	6.79E-04	1.15E-03
CCNB1	cyclin B1	protein_coding	chr5:69167135-	2433	12.23350244	10.72873257	-0.277	6.80E-04	1.15E-03
WSB1	WD repeat and SOCS box containing 1	protein_coding	chr17:27294076	8602	42.43929438	36.0598671	-0.315	6.87E-04	1.16E-03
TAS2R31	taste 2 receptor member 31	protein_coding	chr12:11030387	1021	0.97994384	0.597785938	-0.8	7.01E-04	1.18E-03
HLA-E	major histocompatibility complex, class I	protein_coding	chr6:30489509-	2922	2.893419117	2.55745427	-0.269	7.01E-04	1.18E-03
AL139260.1	novel transcript	lncRNA	chr1:38859912-	2004	1.110366198	0.750014523	-0.642	7.05E-04	1.19E-03
AC004825.2	novel transcript	lncRNA	chr14:70906657	455	1.976267192	1.079050776	-0.916	7.10E-04	1.19E-03
FAM117A	family with sequence similarity 117 meml	protein_coding	chr17:49710332	3800	2.279053157	1.946522042	-0.31	7.24E-04	1.22E-03
FBXO5	F-box protein 5	protein_coding	chr6:152970519	3856	2.272109479	1.993237697	-0.274	7.35E-04	1.24E-03
ZNF429	zinc finger protein 429	protein_coding	chr19:21496682	7571	1.48647555	1.280191733	-0.303	7.49E-04	1.26E-03
ASPM	abnormal spindle microtubule assembly	protein_coding	chr1:197084127	10888	2.485895025	2.103967689	-0.324	7.51E-04	1.26E-03
SPTBN5	spectrin beta, non-erythrocytic 5	protein_coding	chr15:41848146	12312	1.473606038	1.100222916	-0.49	7.56E-04	1.27E-03
FAM86C1	family with sequence similarity 86 memb	protein_coding	chr11:71787510	2973	2.33888359	1.97459709	-0.333	7.69E-04	1.29E-03
SDHAF2	succinate dehydrogenase complex assemb	protein_coding	chr11:61430042	2492	2.372068654	2.084905309	-0.276	7.80E-04	1.31E-03
AC006249.1	novel transcript, sense intronic to CDH2	lncRNA	chr18:28146233	471	1.028228985	0.606530994	-0.825	7.92E-04	1.33E-03
WDR90	WD repeat domain 90	protein_coding	chr16:649311-6	10534	1.751887953	1.456816225	-0.343	7.96E-04	1.33E-03
S100A6	S100 calcium binding protein A6	protein_coding	chr1:153534599	971	1.014749967	0.590139663	-0.84	8.08E-04	1.35E-03
ST7-AS1	ST7 antisense RNA 1	lncRNA	chr7:116952446	1889	3.035530754	2.611178385	-0.304	8.11E-04	1.35E-03
UBR4	ubiquitin protein ligase E3 component n-r	protein_coding	chr1:19074510-	24661	12.97773009	11.14107106	-0.298	8.14E-04	1.36E-03
SLC36A1	solute carrier family 36 member 1	protein_coding	chr5:151437046	6971	1.217696719	1.042478308	-0.306	8.27E-04	1.38E-03
AC022613.2	novel transcript, antisense to TJP1	lncRNA	chr15:29822631	1451	1.853302582	1.443844044	-0.455	8.33E-04	1.39E-03
GPD1	glycerol-3-phosphate dehydrogenase 1	protein_coding	chr12:50103982	3822	1.174519311	0.972380666	-0.351	8.36E-04	1.39E-03
NDUFAF2	NADH:ubiquinone oxidoreductase compl	protein_coding	chr5:60945205-	930	13.2518911	11.38095671	-0.299	8.45E-04	1.41E-03
S100A13	S100 calcium binding protein A13	protein_coding	chr1:153618787	2216	1.211575274	0.854425829	-0.579	8.45E-04	1.41E-03
AC009318.1	novel transcript, antisense to FAR2	lncRNA	chr12:29277955	6167	1.066552036	0.942824123	-0.268	9.03E-04	1.50E-03
HNRNPA1L2	heterogeneous nuclear ribonucleoprotein	protein_coding	chr13:52642425	1372	4.434293386	3.703088569	-0.344	9.06E-04	1.50E-03
RIPK2	receptor interacting serine/threonine kin	protein_coding	chr8:89757806-	3059	2.749491796	2.38795225	-0.288	9.23E-04	1.53E-03
AC091271.1	novel transcript	lncRNA	chr17:59618553	1162	1.808096409	1.343947159	-0.514	9.35E-04	1.55E-03
CFAP58	cilia and flagella associated protein 58	protein_coding	chr10:10435383	3491	1.093113594	0.853655946	-0.466	9.39E-04	1.55E-03
LRRCC1	leucine rich repeat and coiled-coil centro	protein_coding	chr8:85107215-	5001	1.299201739	1.094338539	-0.331	9.42E-04	1.56E-03
C9orf85	chromosome 9 open reading frame 85	protein_coding	chr9:71911510-	4973	1.697591281	1.48097148	-0.283	9.51E-04	1.57E-03
AL122058.1	novel transcript	lncRNA	chr20:57266797	1293	0.983968721	0.616943056	-0.805	9.66E-04	1.60E-03
ZSWIM1	zinc finger SWIM-type containing 1	protein_coding	chr20:45881227	2769	3.409792263	2.980279418	-0.274	9.70E-04	1.60E-03
TGFB2-AS1	TGFB2 antisense RNA 1 (head to head)	lncRNA	chr1:218344196	557	1.016461245	0.586684158	-0.893	1.02E-03	1.68E-03
GEMIN6	gem nuclear organelle associated protein	protein_coding	chr2:38751534-	4363	2.045319713	1.740276061	-0.312	1.03E-03	1.69E-03
S1PR2	sphingosine-1-phosphate receptor 2	protein_coding	chr19:10221433	3643	2.127737638	1.828281329	-0.301	1.05E-03	1.73E-03
ALG5	ALG5 dolichyl-phosphate beta-glucosyltra	protein_coding	chr13:36949738	2231	2.47156982	2.149736866	-0.29	1.08E-03	1.78E-03
MAP1LC3B2	microtubule associated protein 1 light ch	protein_coding	chr12:11654810	1916	1.871252649	1.455072164	-0.451	1.10E-03	1.80E-03
RGS19	regulator of G protein signaling 19	protein_coding	chr20:64073181	2028	2.640350239	2.287261331	-0.297	1.10E-03	1.81E-03
ZNF823	zinc finger protein 823	protein_coding	chr19:11721265	2459	1.448958721	1.083352355	-0.486	1.12E-03	1.84E-03
MRPL48	mitochondrial ribosomal protein L48	protein_coding	chr11:73787316	4386	3.05237557	2.678251071	-0.272	1.15E-03	1.88E-03

AC092139.1	novel transcript	lncRNA	chr16:81385463	754	2.364925878	1.747385626	-0.526	1.16E-03	1.90E-03
CCDC28A	coiled-coil domain containing 28A	protein_coding	chr6:13877350	1507	2.76612728	2.276674436	-0.37	1.17E-03	1.91E-03
DHRS12	dehydrogenase/reductase 12	protein_coding	chr13:51767993	3530	0.969151325	0.757986446	-0.436	1.21E-03	1.98E-03
ANKRD37	ankyrin repeat domain 37	protein_coding	chr4:185396021	2253	0.873914285	0.617294148	-0.581	1.24E-03	2.01E-03
LYAR	Ly1 antibody reactive	protein_coding	chr4:4267701-4	2304	2.340612455	2.030226866	-0.293	1.25E-03	2.03E-03
DLK2	delta like non-canonical Notch ligand 2	protein_coding	chr6:43450352-	2236	1.133408415	0.884454591	-0.45	1.26E-03	2.04E-03
AL358472.3	novel transcript, antisense to SLC39A1	lncRNA	chr1:15396651	415	1.02053643	0.542931787	-0.939	1.28E-03	2.08E-03
C17orf53	chromosome 17 open reading frame 53	protein_coding	chr17:44141906	3005	1.999057286	1.638828841	-0.363	1.30E-03	2.10E-03
AC119427.2	novel transcript	lncRNA	chr1:20135901	1694	3.177535277	2.775946638	-0.281	1.34E-03	2.17E-03
ZNF460	zinc finger protein 460	protein_coding	chr19:57280051	4005	1.714503953	1.513651496	-0.27	1.36E-03	2.20E-03
CDC25C	cell division cycle 25C	protein_coding	chr5:13828526	3338	0.992601437	0.729219429	-0.542	1.40E-03	2.26E-03
C8orf88	chromosome 8 open reading frame 88	protein_coding	chr8:90958471-	1041	7.343784301	6.240545166	-0.313	1.45E-03	2.34E-03
CHKB-DT	CHKB divergent transcript	lncRNA	chr22:50583026	4154	1.069595221	0.88752522	-0.357	1.47E-03	2.35E-03
NDUFB8	NADH:ubiquinone oxidoreductase subuni	protein_coding	chr10:10052374	1999	4.31945647	3.6676181	-0.31	1.48E-03	2.37E-03
LLPH-DT	LLPH divergent transcript	lncRNA	chr12:66130751	623	5.704216534	4.771660541	-0.345	1.50E-03	2.41E-03
AC008736.2	novel transcript	lncRNA	chr19:32687089	568	1.471045254	0.896218881	-0.754	1.57E-03	2.50E-03
AL158829.1	novel transcript	lncRNA	chr9:10976036	478	6.407278236	5.103784701	-0.419	1.57E-03	2.51E-03
PODXL	podocalyxin like	protein_coding	chr7:131500262	6893	1.279004633	1.099642367	-0.298	1.58E-03	2.53E-03
AL606469.1	novel transcript	lncRNA	chr10:23343957	892	1.378645415	0.972539497	-0.581	1.65E-03	2.64E-03
RIMKLA	ribosomal modification protein rimK like	protein_coding	chr1:42380792-	10638	3.021071402	2.53640462	-0.331	1.68E-03	2.67E-03
MICAL3	microtubule associated monooxygenase,	protein_coding	chr22:17787649	22356	6.713094625	5.844907105	-0.276	1.72E-03	2.73E-03
ATP6V1G2	ATPase H+ transporting V1 subunit G2	protein_coding	chr6:31544462-	2147	3.299934319	2.826501218	-0.308	1.82E-03	2.89E-03
PTPRU	protein tyrosine phosphatase receptor ty	protein_coding	chr1:29236516-	6682	1.345720045	1.082274098	-0.392	1.83E-03	2.90E-03
AL353138.1	novel transcript	lncRNA	chr6:48069213-	803	1.893534368	1.396488003	-0.522	1.84E-03	2.92E-03
DSCC1	DNA replication and sister chromatid coh	protein_coding	chr8:11983397	2487	1.467308764	1.167189412	-0.433	1.87E-03	2.96E-03
DBF4	DBF4 zinc finger	protein_coding	chr7:87876216-	4595	2.111358215	1.838813304	-0.284	1.95E-03	3.08E-03
METTL4	methyltransferase like 4	protein_coding	chr18:2537525-	5724	1.504564595	1.288597838	-0.296	1.96E-03	3.10E-03
AL163051.1	novel transcript, antisense to PAPOLA	lncRNA	chr14:96500810	1512	0.976270903	0.709097372	-0.546	1.99E-03	3.14E-03
KRBA2	KRAB-A domain containing 2	protein_coding	chr17:8356902-	3701	1.723800301	1.499164778	-0.291	2.01E-03	3.16E-03
GDF15	growth differentiation factor 15	protein_coding	chr19:18374731	2087	1.159628538	0.897023613	-0.463	2.05E-03	3.23E-03
CCDC134	coiled-coil domain containing 134	protein_coding	chr22:41800679	1270	3.374519211	2.695670394	-0.422	2.06E-03	3.24E-03
ABHD14A	abhydrolase domain containing 14A	protein_coding	chr3:51971426-	2378	1.247475287	0.961173337	-0.447	2.06E-03	3.24E-03
MT3	metallothionein 3	protein_coding	chr16:56589074	1837	1.496213321	1.113950582	-0.486	2.08E-03	3.27E-03
PFKFB3	6-phosphofructo-2-kinase/fructose-2,6-bi	protein_coding	chr10:6144934-	6114	2.92987899	2.402582961	-0.368	2.29E-03	3.58E-03
LRRC73	leucine rich repeat containing 73	protein_coding	chr6:43506969-	2121	2.078170961	1.795464039	-0.298	2.54E-03	3.94E-03
DOCK9-DT	DOCK9 divergent transcript	lncRNA	chr13:99087819	807	1.845795826	1.298803791	-0.616	2.55E-03	3.95E-03
ARL6IP6	ADP ribosylation factor like GTPase 6 inte	protein_coding	chr2:15271789	3349	2.066347205	1.80519637	-0.272	2.58E-03	4.00E-03
AC113189.1	novel transcript, antisense to TMEM102	lncRNA	chr17:7436557-	327	0.805815208	0.38101745	-1.178	2.62E-03	4.05E-03
DOHH	deoxyhypusine hydroxylase	protein_coding	chr19:3490822-	2031	7.872959334	6.881588267	-0.276	2.63E-03	4.07E-03
DACT3-AS1	DACT3 antisense RNA 1	lncRNA	chr19:46660364	1825	2.126639994	1.833326719	-0.298	2.76E-03	4.26E-03
AC015983.2	novel transcript	lncRNA	chr7:12214440	1888	0.831558454	0.533166702	-0.718	2.77E-03	4.27E-03
SLC25A35	solute carrier family 25 member 35	protein_coding	chr17:8287763-	3952	1.817777196	1.599507261	-0.263	2.81E-03	4.34E-03
TMEM54	transmembrane protein 54	protein_coding	chr1:32894594-	1417	3.027570376	2.419246717	-0.393	2.89E-03	4.45E-03
TMEM267	transmembrane protein 267	protein_coding	chr5:43444252-	3158	2.108814582	1.810621462	-0.298	2.90E-03	4.46E-03
AL513550.1	novel transcript	lncRNA	chr6:99424922-	2588	1.149135732	0.951340305	-0.367	2.94E-03	4.51E-03
AL591845.1	novel transcript, antisense to EVA1B	lncRNA	chr1:36323734-	1838	2.358975526	1.916451175	-0.381	3.03E-03	4.64E-03
B4GALNT4	beta-1,4-N-acetyl-galactosaminyltransfer	protein_coding	chr11:369499-3	4059	1.628580725	1.408588143	-0.293	3.07E-03	4.71E-03
CLDN9	claudin 9	protein_coding	chr16:3012923-	1583	1.084926539	0.802707734	-0.524	3.09E-03	4.74E-03
AC009041.4	novel transcript, sense intronic to LMF1	lncRNA	chr16:968375-9	638	1.765007671	1.14365283	-0.721	3.21E-03	4.92E-03
AC026803.1	novel transcript, overlapping NUCB1	lncRNA	chr19:48926171	274	0.72976091	0.258603407	-1.398	3.33E-03	5.07E-03
GPR137B	G protein-coupled receptor 137B	protein_coding	chr1:23614250	2307	1.868160907	1.529627518	-0.352	3.43E-03	5.22E-03
SLC44A3	solute carrier family 44 member 3	protein_coding	chr1:94820342-	2869	0.958149179	0.755757057	-0.443	3.49E-03	5.30E-03
AL162377.1	novel transcript	lncRNA	chr13:51803838	2469	1.060255128	0.889060678	-0.347	3.60E-03	5.47E-03
SRD5A3	steroid 5 alpha-reductase 3	protein_coding	chr4:55346242-	4291	1.164860907	1.018499866	-0.287	3.65E-03	5.53E-03
AC079610.2	novel transcript	lncRNA	chr2:21315297	690	0.99750674	0.635849537	-0.738	3.67E-03	5.55E-03
ZSWIM3	zinc finger SWIM-type containing 3	protein_coding	chr20:45857614	2776	1.709151525	1.509558895	-0.272	3.77E-03	5.69E-03
LINC01402	long intergenic non-protein coding RNA 1	lncRNA	chrX:120117642	279	0.955600482	0.400000313	-1.481	3.79E-03	5.72E-03
GAS2L3	growth arrest specific 2 like 3	protein_coding	chr12:10057368	7425	1.173841042	1.027777439	-0.277	3.85E-03	5.81E-03
KCNJ2-AS1	KCNJ2 antisense RNA 1	lncRNA	chr17:70166961	2442	1.555476566	1.293953107	-0.34	3.91E-03	5.89E-03
OAF	out at first homolog	protein_coding	chr11:12021103	2913	1.064542019	0.880131678	-0.346	4.07E-03	6.12E-03
UBAC2-AS1	UBAC2 antisense RNA 1	lncRNA	chr13:99181223	2628	0.911155753	0.724379896	-0.421	4.07E-03	6.12E-03
AL162586.1	novel transcript	lncRNA	chr9:12781606	2247	0.949956217	0.769140616	-0.389	4.12E-03	6.19E-03
AL049597.2	novel transcript, antisense to SH3GLB1	lncRNA	chr1:86703502-	961	1.286974142	1.004287766	-0.438	4.20E-03	6.30E-03
MTLN	mitoregulin	protein_coding	chr2:11021152	2051	10.40814078	8.80809156	-0.315	4.42E-03	6.62E-03
C1orf162	chromosome 1 open reading frame 162	protein_coding	chr1:111473792	2768	1.378122317	1.190055301	-0.287	4.55E-03	6.79E-03
MIR503HG	MIR503 host gene	lncRNA	chrX:13454311	2463	1.711238264	1.439230631	-0.34	4.55E-03	6.79E-03
SFR1	SWI5 dependent homologous recombina	protein_coding	chr10:10412205	1812	2.522624698	2.195625475	-0.288	4.59E-03	6.86E-03
EGLN3	egl-9 family hypoxia inducible factor 3	protein_coding	chr14:33924231	7359	4.143856125	3.096784498	-0.524	4.63E-03	6.90E-03
ZNF440	zinc finger protein 440	protein_coding	chr19:11814284	6427	1.23686979	1.063858821	-0.297	4.68E-03	6.97E-03
AC005609.4	novel transcript, antisense to PCDHA10	lncRNA	chr5:140867513	447	0.806081964	0.486095108	-0.794	4.70E-03	7.00E-03
AC124861.1	novel transcript	lncRNA	chr2:24022756	429	0.911770386	0.407088518	-1.238	4.83E-03	7.17E-03
APOM	apolipoprotein M	protein_coding	chr6:31652416-	1237	1.024997109	0.68046728	-0.63	4.87E-03	7.23E-03
AC018413.1	novel transcript, antisense to ZFN156	lncRNA	chr18:76491629	1574	0.882141697	0.658922628	-0.499	5.46E-03	8.03E-03
HIST1H3H	histone cluster 1 H3 family member h	protein_coding	chr6:27810064-	1237	1.266623962	0.969849972	-0.469	5.84E-03	8.55E-03
AC084125.4	novel transcript, antisense to RECQL4	lncRNA	chr8:144512567	338	1.035554409	0.537959772	-0.96	5.88E-03	8.61E-03
CCDC59	coiled-coil domain containing 59	protein_coding	chr12:82223681	3518	3.906101656	3.357729717	-0.294	6.08E-03	8.89E-03
NKILA	NF-kappaB interacting lncRNA	lncRNA	chr20:57710156	2625	1.457627844	1.260632333	-0.297	6.23E-03	9.09E-03
AC083880.1	novel transcript, antisense to LUC7L2	lncRNA	chr7:139359032	535	2.667161159	2.065867092	-0.454	6.43E-03	9.35E-03
TRAIP	TRAF interacting protein	protein_coding	chr3:49828601-	3261	0.880645877	0.695719121	-0.425	6.44E-03	9.36E-03
TWIST1	twist family bHLH transcription factor 1	protein_coding	chr7:19020991-	1997	1.230678121	1.016228338	-0.363	6.88E-03	9.98E-03
NBPF26	NBPF member 26	protein_coding	chr1:12072394	6782	1.639993968	1.435597973	-0.268	7.25E-03	1.05E-02
AC005609.3	novel transcript, antisense to PCDHA12	lncRNA	chr5:14087534	577	0.891455819	0.578292277	-0.717	7.28E-03	1.05E-02
SLC35A1	solute carrier family 35 member A1	protein_coding	chr6:87470623-	1994	1.852757325	1.637266681	-0.263	7.30E-03	1.05E-02
CENPQ	centromere protein Q	protein_coding	chr6:49463370-	1741	2.148686159	1.871671064	-0.282	7.32E-03	1.06E-02
AC025181.2	novel transcript	lncRNA	chr5:32174471-	802	3.088503477	2.388089482	-0.435	7.47E-03	1.08E-02
AC108047.1	novel transcript	lncRNA	chr2:19053485	2078	1.033215807	0.847034422	-0.378	7.48E-03	1.08E-02
AC132938.3	novel transcript, antisense NARF	lncRNA	chr17:8245414	1097	1.010858642	0.746498755	-0.521	7.50E-03	1.08E-02
AC039056.2	novel transcript, sense intronic to EHD4	lncRNA	chr15:41908204	511	0.766179593	0.469302766	-0.783	7.64E-03	1.10E-02
ZNF551	zinc finger protein 551	protein_coding	chr19:57681969	4703	1.252302651	1.098364179	-0.264	7.85E-03	1.13E-02
SH3D21	SH3 domain containing 21	protein_coding	chr1:36306387-	4474	3.93895659	3.132400263	-0.405	7.90E-03	1.13E-02
AC253536.3	novel transcript	lncRNA	chr22:24101689	558	4.358203663	3.399297827	-0.421	8.12E-03	1.16E-02
AC020910.4	novel transcript	lncRNA	chr19:34733298	540	1.794115132	1.257313073	-0.576	8.46E-03	1.21E-02
TBC1D8-AS1	TBC1 domain family member 8 antisense I	lncRNA	chr2:10115166	2478	0.907973741	0.733978354	-0.376	8.54E-03	1.22E-02
THAP10	THAP domain containing 10	protein_coding	chr15:70881342	2047	2.087322785	1.765926047	-0.338	8.66E-03	1.24E-02
AC095057.3	novel transcript, overlapping N4BP2	lncRNA	chr4:40166675-	1157	0.915847526	0.634611354	-0.624	9.03E-03	1.29E-02
AC107952.2	novel transcript	lncRNA	chr8:56222688-	486	0.701098626	0.238243625	-1.684	9.36E-03	1.33E-02
AC135048.3	novel transcript	lncRNA	chr16:30956872	328	1.601869297	0.955116496	-0.794	9.48E-03	1.35E-02
SYNE1-AS1	SYNE1 antisense RNA 1	lncRNA	chr6:15238054	553	1.400434162	0.926829789	-0.639	9.76E-03	1.38E-02
TRAF1	TNF receptor associated factor 1	protein_coding	chr9:120902393	4476	0.872501202	0.738010519	-0.319	9.78E-03	1.39E-02
AP001033.2	novel transcript, sense intronic to ANKRD	lncRNA	chr18:9259388-	1003	1.395075817	1.060955175	-0.482	9.97E-03	1.41E-02
LINC01449	long intergenic non-protein coding RNA 1	lncRNA	chr7:41089539-	2468	1.796834735	1.540864912	-0.315	1.01E-02	1.43E-02
AC145422.1	novel transcript	lncRNA	chr12:12158079	544	1.477551003	1.032151022	-0.575	1.01E-02	1.43E-02
SLC16A3	solute carrier family 16 member 3	protein_coding	chr17:82228397	8995	6.332542853	5.19873086	-0.371	1.03E-02	1.45E-02
AC011461.1	novel transcript, sense intronic with KEAF	lncRNA	chr19:10490339	357	1.213301498	0.818912982	-0.685	1.03E-02	1.46E-02
AC104393.1	novel transcript	lncRNA	chr8:41275115-	255	0.792785161	0.338110177	-1.337	1.07E-02	1.51E-02
UCHL3	ubiquitin C-terminal hydrolase L3	protein_coding	chr13:7554948	1905	1.326518054	1.118291459	-0.326	1.11E-02	1.55E-02
AC027277.2	novel transcript, sense intronic to EMP2	lncRNA	chr16:10576499	435	1.132219029	0.760972464	-0.64	1.12E-02	1.57E-02
AL078644.2	novel transcript	lncRNA	chr1:185317779	752	0.884276183	0.581966043	-0.735	1.14E-02	1.60E-02
GAS6-AS1	GAS6 antisense RNA 1	lncRNA	chr13:11381563	7169	1.200631595	1.01857046	-0.316	1.16E-02	1.63E-02

AL353194.1	novel transcript	lncRNA	chr20:3888239-	441	2.625842804	2.062307976	-0.447	1.18E-02	1.64E-02
LINC01290	long intergenic non-protein coding RNA 1	lncRNA	chr16:10514842	502	1.084951477	0.766587762	-0.596	1.20E-02	1.67E-02
ADNP-AS1	ADNP antisense RNA 1	lncRNA	chr20:50930984	1049	1.149624828	0.91588324	-0.447	1.39E-02	1.92E-02
AC139530.4	novel transcript, antisense to HGS	lncRNA	chr17:81697025	690	0.952105202	0.656517966	-0.605	1.39E-02	1.92E-02
CTH	cystathionine gamma-lyase	protein_coding	chr1:70411218-	2414	1.162364137	1.020942426	-0.282	1.39E-02	1.93E-02
AL118558.3	novel transcript	lncRNA	chr14:10194834	1079	3.830233004	3.101804906	-0.37	1.48E-02	2.05E-02
AC005277.1	novel transcript, antisense to ARHGAP44	lncRNA	chr17:12990149	335	0.590992534	0.243353308	-1.324	1.62E-02	2.21E-02
C10orf71-AS1	C10orf71 antisense RNA 1	lncRNA	chr10:49296112	963	2.885462452	2.208583981	-0.449	1.67E-02	2.28E-02
AC099568.2	novel transcript	lncRNA	chr1:89820174-	695	0.993063807	0.726804216	-0.553	1.68E-02	2.29E-02
FAM110A	family with sequence similarity 110 meml	protein_coding	chr20:833715-8	2722	1.642079769	1.447885206	-0.274	1.69E-02	2.31E-02
LINC02248	long intergenic non-protein coding RNA 2	lncRNA	chr15:26395037	2804	1.051555258	0.887382719	-0.319	1.73E-02	2.36E-02
CACNA1C-IT2	CACNA1C intronic transcript 2	lncRNA	chr12:2048352-	589	1.936015858	1.394781188	-0.563	1.96E-02	2.65E-02
MED30	mediator complex subunit 30	protein_coding	chr8:117520713	1440	2.619002791	2.330710209	-0.271	1.99E-02	2.69E-02
AC100793.2	novel transcript, antisense to CCR10	lncRNA	chr17:42679963	542	2.498548549	1.93675152	-0.493	2.04E-02	2.75E-02
HYAL3	hyaluronidase 3	protein_coding	chr3:50292831-	1967	1.094534561	0.879747951	-0.419	2.22E-02	2.99E-02
AP000350.5	novel transcript, antisense to SLC2A11	lncRNA	chr22:23856427	613	1.023079635	0.796743612	-0.433	2.24E-02	3.01E-02
AC115618.1	novel transcript, antisense to RBM3	lncRNA	chrX:48568014-	549	1.002203039	0.717988098	-0.549	2.25E-02	3.02E-02
AC040934.1	novel transcript	lncRNA	chr17:47409322	513	1.036571902	0.798465581	-0.454	2.28E-02	3.06E-02
AC106782.2	novel transcript	lncRNA	chr16:30355441	992	1.032605575	0.817711733	-0.417	2.34E-02	3.13E-02
LGALS1	galectin 1	protein_coding	chr22:37675636	1067	3.283907815	2.782613407	-0.31	2.45E-02	3.27E-02
GFOD1-AS1	GFOD1 antisense RNA 1	lncRNA	chr6:13486294-	332	0.810847995	0.498348662	-0.692	2.71E-02	3.58E-02
ITM2A	integral membrane protein 2A	protein_coding	chrX:79360384-	2051	1.367513949	1.203791579	-0.266	2.77E-02	3.66E-02
HES7	hes family bHLH transcription factor 7	protein_coding	chr17:8120592-	1880	1.044618038	0.875231075	-0.33	2.84E-02	3.75E-02
AP005482.3	novel transcript, sense intronic CEP76	lncRNA	chr18:12670426	720	1.495756467	1.178406426	-0.409	3.05E-02	4.00E-02
AL596094.1	novel transcript	lncRNA	chr10:5813985-	276	1.509940226	1.113495285	-0.51	3.09E-02	4.06E-02
AC005342.1	novel transcript	lncRNA	chr12:2004666-	629	1.788630858	1.367027181	-0.431	3.19E-02	4.18E-02
BEND5	BEN domain containing 5	protein_coding	chr1:48727519-	2142	1.178630909	1.023555788	-0.29	3.21E-02	4.20E-02
MTRNR2L10	MT-RNR2 like 10	protein_coding	chrX:55181391-	1530	1.969376061	1.643434164	-0.318	3.22E-02	4.21E-02
AC117402.1	novel transcript	lncRNA	chr3:110888384	1363	0.907903676	0.707359942	-0.408	3.36E-02	4.39E-02
AC009309.1	novel transcript, antisense to IMMT	lncRNA	chr2:86195590-	460	3.564570801	3.031529514	-0.31	3.38E-02	4.41E-02
AC005288.1	novel transcript, antisense to MED1 & FB)	lncRNA	chr17:39401793	1053	1.412803618	1.166262322	-0.361	3.46E-02	4.51E-02
FMC1	formation of mitochondrial complex V as:	protein_coding	chr7:139339457	1328	1.037642614	0.872086986	-0.331	3.49E-02	4.54E-02
MTRNR2L12	MT-RNR2 like 12	protein_coding	chr3:96617188-	1049	12.16922117	10.45592193	-0.268	3.49E-02	4.54E-02
AC018648.1	novel transcript	lncRNA	chr7:32845394-	668	0.730245936	0.478446879	-0.634	3.80E-02	4.93E-02

Supplemental Table 3: List of oligonucleotides used for CRISPR-Cas9 experiments

Nomencalture used in this paper	Sequence
sgRNA BRAF	TCGAGATTTCACTGTAGCTA
ssODN T599R	GGGCCAAAAATTTAATCAGTGGAAAAATAGCCTCAATTCTTACCAT<br>CCACAAAATGGATCCAGACAACTGTTCAAACTGATGGGACCCACTCC<br>ATCGAGATTTCACTCTCGCTAGACCAAAATCACCTAT
Fwd-BRAF	CCTAACACATTTCAAGCCCCA
Rev-BRAF	AACTCAGCAGCATCTCAGGG
